# Fast relaxing red and near-IR switchable azobenzenes with chalcogen and halogen substituents: periodic trends, tuneable thermal half-lives and chalcogen bonding†

**DOI:** 10.1039/d2sc04601f

**Published:** 2022-09-20

**Authors:** Aidan Kerckhoffs, Kirsten E. Christensen, Matthew J. Langton

**Affiliations:** Chemistry Research Laboratory, Department of Chemistry, University of Oxford 12 Mansfield Road Oxford OX1 3TA UK matthew.langton@chem.ox.ac.uk

## Abstract

Molecular photoswitches operating in the red to near-IR region with controllable thermal relaxation rates are attractive components for photo-regulating biological processes. Herein, we report the synthesis of red-shifted azobenzenes functionalised with the heavier chalcogens and halogens that meet these requirements for biological application; namely fatigue-resistant photo-switching with red and near IR light and functional handles for further functionalisation for application. We report robust periodic trends for the chalcogen and halogen azobenzene series, and exploit intramolecular chalcogen bonding to tune and redshift the absorption maxima, supported by photo-physical measurements and solid-state structural analysis. Remarkably, the rate of the *Z* → *E* thermal isomerisation can be tuned over timescales spanning 10^7^ s by judicious choice of chalcogen and halogen substituents.

## Abstract 2

The synthesis of red-shifted azobenzenes functionalised with heavy chalcogen and halogen substituents is reported, enabling photo-switching with red and near IR light and *Z* → *E* thermal isomerization rates spanning seven orders of magnitude.

## Graphical abstract

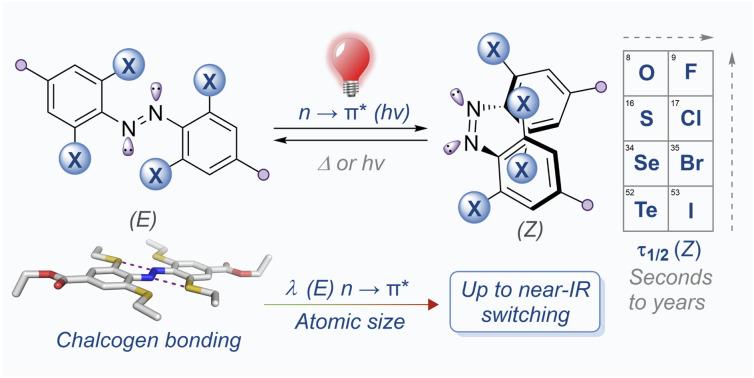

## Introduction

Azobenzenes (ABs) are attractive photoswitches due to their robust photo-switching, large conformational changes, low rate of photobleaching, and ease of synthesis and derivatization.^1^ Azobenzene motifs have thus been successfully employed in a plethora of applications, including molecular machines,^2,3^ smart materials,^4–6^ crystal engineering,^7^ transmembrane ion transport^8–10^ and non-invasive manipulation of biological systems, in particular within the emerging field of photo-pharmacology.^11–13^ Within the last decade, there have been significant efforts towards developing ABs,^14–17^ as well as related photoswitches such as diazocine,^18^ azonium^19^ and azo-BF_2_ derivatives,^20^ that can be isomerised using light in the biocompatible red to near-infrared region with controlled thermal relaxation rates. This is primarily driven by potential applications within biological contexts: longer wavelengths of light (>600 nm) exhibit significantly higher tissue penetrance,^21^ are absorbed less by hemoglobin,^22^ and do not display any toxic or mutagenic effects observed with high energy UV light that are typically required to isomerise ABs and other photoswitches such as stilbene.^23,24^

**Fig. 1:**
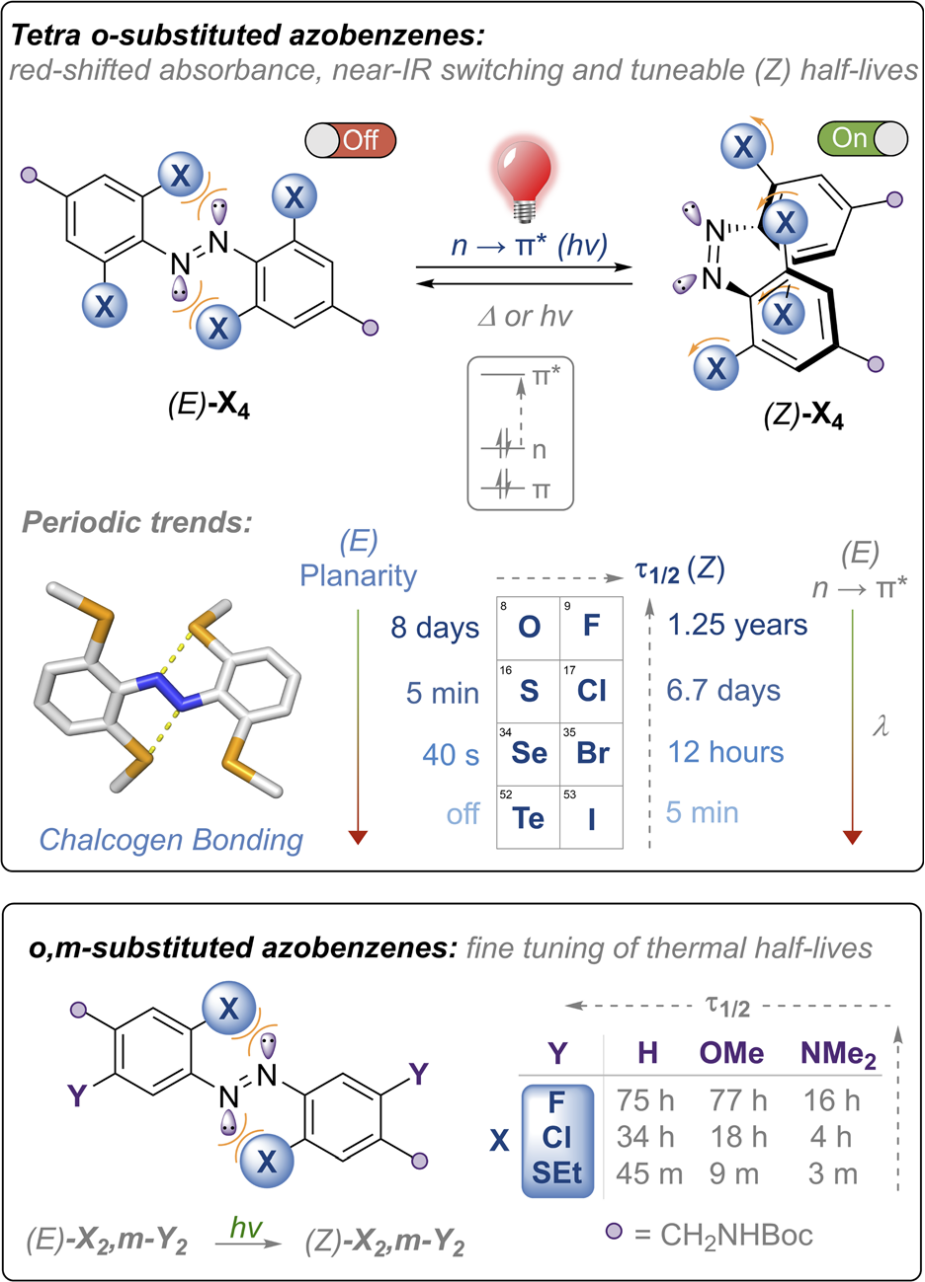
Heavy chalcogen and halogen substituted azobenzenes described in this article: periodic trends, thermal half-life tuning and near-IR photo-switching. No photo-switching was detected at room temperature for the tetra-telluride Te_4_.

Tetra *ortho*-substitution of azobenzenes with the lighter chalcogens and halogens (Fig. 1) is an established approach for red-shifting *trans*-ABs, pioneered by Woolley (X = Cl, Br, OMe, SEt)^15,25,26^ and Hecht (X = F).^14^ In these systems *E* → *Z* isomerisation is triggered using green or red light, exciting into the red-shifted n → π* transitions, whereas *Z* → *E* isomerisation occurs using higher energy blue or violet LEDs, with photo-stationary states (PSS) ranging from ∼85–95%. Thermal relaxation of the *Z* isomer of these derivatives is generally slow (half-lives of hours to years). Developing faster relaxing derivatives of these systems, whilst maintaining the efficient switching with visible light (and ideally with red/near IR wavelengths), is an attractive goal, because generation of the *Z* isomer can be achieved using bio-compatible wavelengths of light whilst relying only on thermal relaxation to quantitatively regenerate the *E* isomer. Typically, it is more convenient for the *Z* isomer to be the active ‘on’ form of the photoswitch, because this allows for the activation of systems that switch ‘off’ over time in the absence of light, without requiring a second excitation wavelength.^11^

Access to a tuneable arsenal of structurally similar azobenzenes activated with biocompatible wavelengths of light, and exhibiting varying thermal relaxation rates, is also desirable to match the timescales of various biological processes. For example, *Z* → *E* thermal relaxation kinetics in the timeframe of minutes is suitable for photocontrol of transcription factors,^25^ whereas photochemical manipulation of ion channels has been demonstrated using azobenzenes that relax within seconds.^27^ It is therefore appealing to design redshifted photoswitches that thermally relax in the timescale of seconds to minutes, where relaxation does not outcompete the influx of light and photoisomerisation.^28,29^ To date, only a handful of studies guiding the design of redshifted azobenzenes exist, and scope for further functionalisation is limited.^28–30^ Furthermore, the vast majority of fast-relaxing azobenzenes are asymmetric push–pull systems, and not structurally analogous to the highly red-shifted and thermally stable *ortho*-substituted azobenzenes, preventing interchange of the photo-switch in applications where systematic modulation of thermal half-lives are desired.^31^

Herein, we report the first examples of the heavy chalcogen and halogen tetra-*ortho* functionalised azobenzenes (where X = SeMe, TeMe and I), and demonstrate how steric clash and intramolecular chalcogen bonding may be used to tune and red-shift the absorption maxima for azobenzene isomerisation, achieving photo-switching in the unprecedented near IR region for the tetra-*ortho*-iodo derivative (Fig. 1, top). We comprehensively examine the substituent effects and periodic trends down the chalcogen and halogen series, and show that thermal half-lives may be tuned over seven orders of magnitude within the family of structurally related ABs, ranging from essentially bistable derivatives (half-lives of days to years) through to those that relax on the timescale of seconds, all photo-switched using visible or near-IR light. We also explore the impact of di-*ortho*/di-*meta* substituted scaffolds on photo-switching properties and show that this substitution pattern allows for fine-tuning of the thermal half-lives (Fig. 1, bottom). Importantly, all scaffolds are appended with functional handles for further post-synthetic modification that is required for downstream application.

## Results and discussion

### Approach

The nature of the *ortho*-substituent directly influences the n orbital energies of ABs. In the case of (*E*)-Cl_4_ and (*E*)-(OMe)_4_, repulsive interactions between the diazo lone pairs and *ortho* substituent has been shown to destabilise the n molecular orbital, leading to red-shifted n → π* transitions in the *E* isomer (Fig. 1, top).^26,29^ The tetra-chloro derivative (*E*)-Cl_4_ has been proposed to be conformationally flexible, exhibiting n → π* (S_0_ → S_1_) transitions with extended red-shifted tails, because the minor populations of planar conformations raise the n orbital energy.^29^ Upon photo-switching to the *Z*-isomer, this repulsion is reduced, leading to large separations of the n → π* bands of the *E* and *Z* isomers, enabling high photo-stationary state ratios to be accessed. It is desirable to photo-isomerise ABs by exciting n → π* bands, as these typically proceed with higher quantum yields and longer wavelengths than by exciting the π → π* transitions.^16^

With these previous results in mind, our initial strategy to access highly red-shifted, fast relaxing ABs was to extend the known tetra-*ortho* azobenzene derivatives X_4_ to the unknown heavier congeners (Fig. 1, top). We anticipated that the larger, heavier atoms (Se, Te, I) would lead to more redshifted n → π* tails due to increased destabilising clash with the diazo N lone pairs. Furthermore, we sought to explore whether, in the case of the chalcogens, intramolecular chalcogen bonds between the diazo lone pair and the chalcogen atom sigma hole would lead to higher populations of planar (and thus more red-shifted) conformations. Analogous to the well-known halogen bonds,^32^ chalcogen bonds are potent non-covalent interactions between a polarised chalcogen atom and a Lewis base, with applications in crystal engineering,^33^ construction of complex hierarchal supramolecular assemblies,^34^ anion recognition^35–37^ and transport.^38–40^

Our second strategy was to explore the di-*ortho*-substituted analogues (*E*)-X_2_,*m*-Y_2_, anticipating that with only two *ortho* heteroatoms the azobenzenes would retain a planar conformation whilst red-shifting the n → π* transition.^26^ We reasoned that a *meta* electron-donating substituent would shift absorbances to higher wavelengths due to the rule of auxochromes, whilst *meta* electron donating groups have previously been shown to significantly reduce the half-life of *ortho*-substituted azobenzenes to the (milli)second range when X = OMe.^41^ We therefore systematically explored combinations of X = F, Cl, OMe, SEt, pyrrolidine and phosphonate ester, and Y = H, OMe, NMe_2_, whilst retaining functional handles for derivatisation (see ESI† for full experimental procedures and characterisation).

**Fig. 2:**
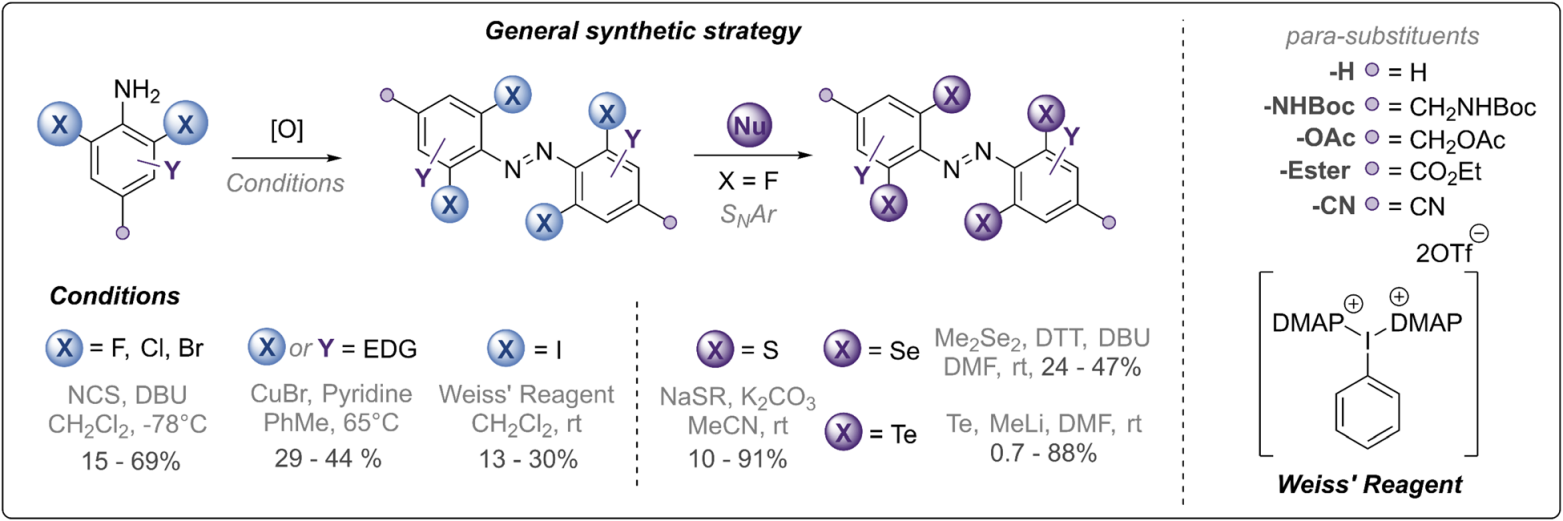
Synthetic strategy towards chalcogen and halogen substituted azobenzene derivatives (see Fig. 1 and Table 1 for structures).

Our synthetic strategy is summarised in Fig. 2 and all synthesised derivatives are tabulated in Table 1 (see ESI† for full experimental details and optimisation of synthetic routes). In general, *ortho*-halogenated and methoxy derivatives were accessed *via* oxidative dimerisation of the corresponding aniline, with yields typically decreasing with increasing steric bulk. Preparation of tetra-thio derivatives *via* this strategy generally proceeded with extremely low yields (∼2%),^25^ presumably due to competitive oxidation of the thioether units and the inherent challenges of forming the extremely sterically hindered bond. Thus, a strategy using nucleophilic aromatic substitution with *ortho*-fluoro derivatives was employed, providing yields up to 90%. This strategy was also exploited to access the unprecedented *ortho*-seleno and telluro derivatives. The photo-switches are equipped with functional handles (*p*-CH_2_NHBoc, *p*-CO_2_Et, *p*-CH_2_N_3_, *p*-CH_2_OH) for further derivatisation.

**Table 1:** List of photo-switches studied in this article

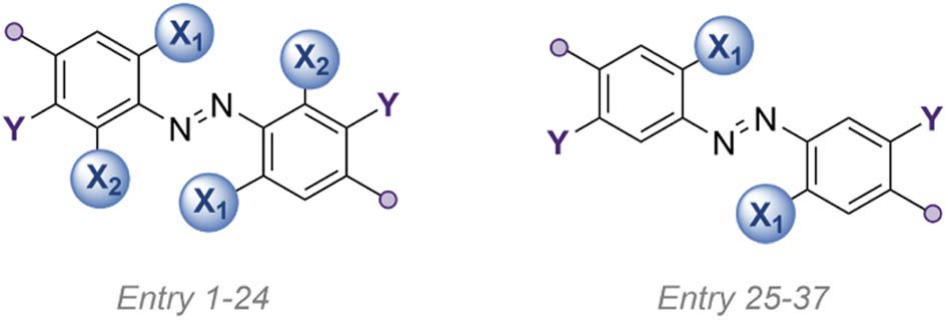										
	AB	X_1_, X_2_^a^	Y	*Para*	Conditions	Yield	*τ*_1/2_	*λ*_excite_ e (nm)	*λ*_max_ f (nm)	PSS
1	F_4_-H	F	H	H	KMnO_4_/FeSO_4_	28%	b	b	b	b
2	F_4_-NHBoc	F	H	CH_2_NHBoc	NCS/DBU	69%	1.25*y*	530	454	84%
3	F_4_-Ester	F	H	CO_2_Et	NCS/DBU	92%	b	b	b	b
4	Cl_4_-NHBoc	Cl	H	CH_2_NHBoc	NCS/DBU	43%	160 h	625	462.6	77%
5	Br_4_-NHBoc	Br	H	CH_2_NHBoc	NCS/DBU	15%	12 h	625	465.9	66%
6	I_4_-OAc	I	H	CH_2_OAc	Weiss' Reagent	13%	2 m	625	501.3	b
7	I_4_-OH	I	H	CH_2_OH	From entry 6	—	b	b	500.3	b
8	I_4_-N_3_	I	H	CH_2_N_3_	From entry 6	—	3 m	625	514.4	b
9	I_4_-NPhth	I	H	CH_2_NPhth	From *entry 6*	—	3 m	625^g^	511.8	50%
10	I_4_-NHBoc	I	H	CH_2_NHBoc	From *entry 6*	—	5 m	660	514.3	b
11	I_4_-CN	I	H	CN	Weiss' Reagent	26%	20 s	660	520.8	b
12	I_4_-ester	I	H	CO_2_Et	Weiss' Reagent	30%	46 s	660^h^	519.6	i
13	(OMe)_4_-NHBoc	OMe	H	CH_2_NHBoc	CuBr/pyridine	38%	8.2 d	625	459.8	95%
14	(OMe)_2_F_2_,*m*-OMe_2_-NHBoc	OMe, F	OMe	CH_2_NHBoc	CuBr/pyridine	3%	c	530	425.4	b
15	(SMe)_4_-H	SMe	H	H	S_N_Ar from *1*	91%	5 m	625	514.2	b
16	(SEt)_4_-NHBoc	SEt	H	CH_2_NHBoc	S_N_Ar from *2*	72%	3 m	625	514.5	46%
17	(SEt)_4_-ester	SEt	H	CO_2_Et	S_N_Ar from *3*	44%	52 s	660	590.0	b
18	(S^i^Pr)_4_-NHBoc	S^i^Pr	H	CH_2_NHBoc	S_N_Ar from *2*	35%	3 m	590	500.4	b
19	(SEt)_4_,*m*-OMe_2_-NHBoc	SEt	OMe	CH_2_NHBoc	S_N_Ar	36%	c	590	512.9	b
20	(SeMe)_4_-H	SeMe	H	H	S_N_Ar from *1*	47%	80 s	625	533.9	i
21	(SeMe)_4_-NHBoc	SeMe	H	CH_2_NHBoc	S_N_Ar from *2*	24%	34 s	625	534.3	i
22	(SeMe)_4_-ester	SeMe	H	CO_2_Et	S_N_Ar from *3*	34%	2.5 s	660	588.8	b
23	Te_2_F_2_-H	TeMe, F	H	H	S_N_Ar from *1*	88%	d	n/a	543	b
24	(TeMe)_4_-H	TeMe	H	H	S_N_Ar from *23*	0.7%	d	n/a	518.5	b
25	F_2_,*m*-H_2_	F	H	CH_2_NHBoc	NCS/DBU	36%	75 h	530	452.5	50%
26	F_2_,*m*-OMe_2_	F	OMe	CH_2_NHBoc	CuBr/pyridine	14%	77 h	530	387.1	50%
27	F_2_,*m*-(NMe_2_)_2_	F	NMe_2_	CH_2_NHBoc	CuBr/pyridine	38%	16 h	530	405.5	48%
28	Cl_2_,*m*-H_2_	Cl	H	CH_2_NHBoc	NCS/DBU	10%	34 h	530	471.0	50%
29	Cl_2_,*m*-OMe_2_	Cl	OMe	CH_2_NHBoc	CuBr/pyridine	14%	18 h	530	475.0	77%
30	Cl_2_,*m*-(NMe_2_)_2_	Cl	NMe_2_	CH_2_NHBoc	CuBr/pyridine	44%	4 h	530	432.1	50%
31	OMe_2_,*m*-OMe_2_	OMe	OMe	CH_2_NHBoc	CuBr/pyridine	17%	c	530	426.6	b
33	(SEt)_2_,*m*-H_2_	SEt	H	CH_2_NHBoc	S_N_Ar from *25*	10%	44 m	590	431.3	50%
34	(SEt)_2_,*m*-OMe_2_	SEt	OMe	CH_2_NHBoc	S_N_Ar from *26*	21%	9 m	590	464.1	b
35	(SEt)_2_,*m*-(NMe_2_)_2_	SEt	NMe_2_	CH_2_NHBoc	S_N_Ar from *27*	10%	140 s	590	543.0	b
36	(N(CH_2_)_4_)_2_,*m*-H	N(CH_2_)_4_	H	CH_2_NHBoc	CuBr/pyridine	29%	c	530	530.2	b
37	(PO(OEt)_2_)_2_,*m*-H	PO(OEt)_2_	H	H	Cross coupling	51%	c	530	458.2	b

a X_1_ = X_2_ if one entry.

b Not determined.

c Decomposed under irradiation with light.

d No photo-switching observed.

e Emission maximum of the LED used for photo-switching.

f *λ*_max_ of n → π* transition.

g Switching possible up to 660 nm (43% *Z* PSS).

h Switching possible up to 730 nm.

i Thermal half-life too fast to determine the PSS distribution. All half-life and photo-switching experiments conducted in DMSO at 298 K.

### Optical, electronic and structural analysis

**Fig. 3:**
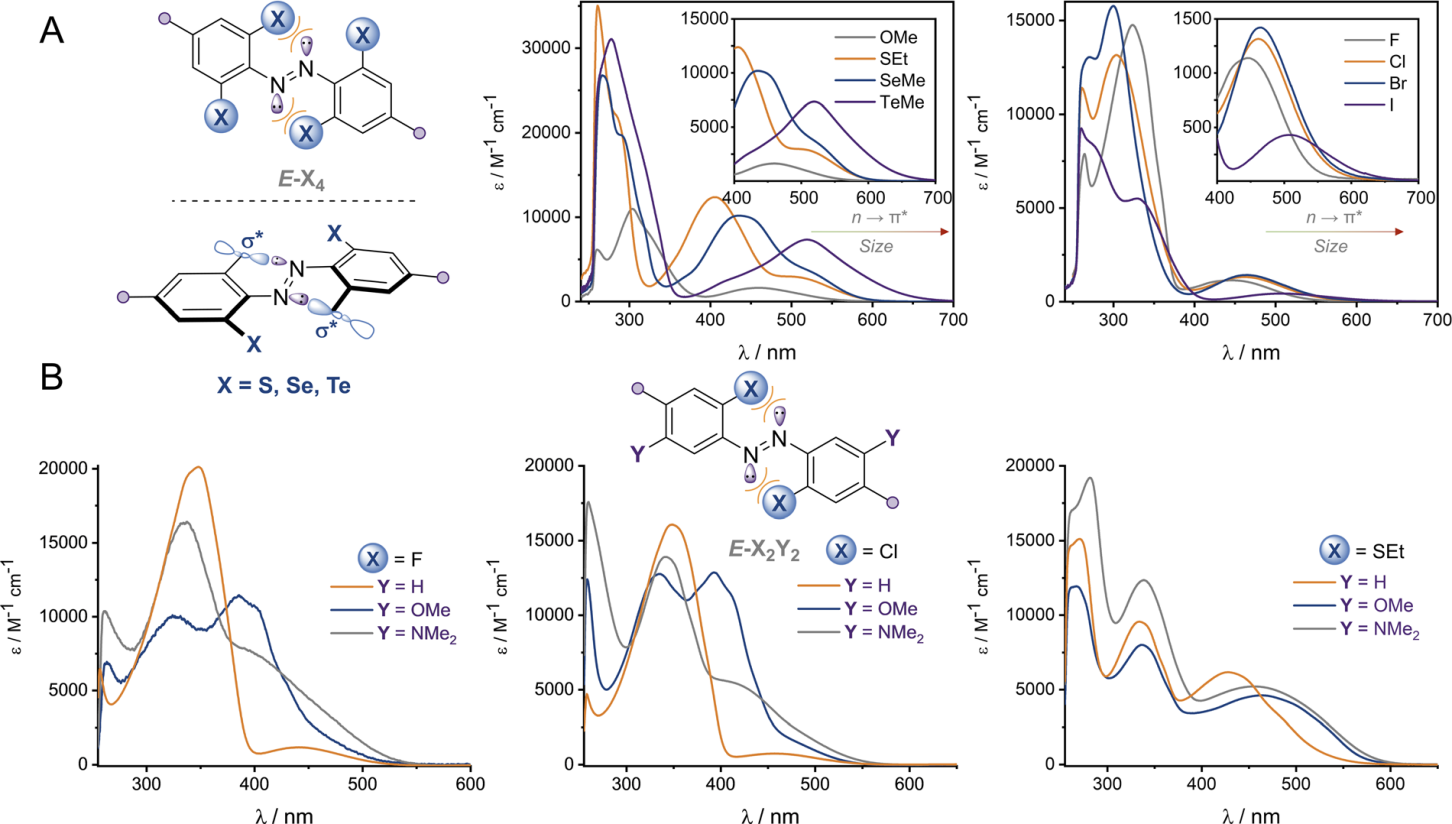
UV-vis spectra of (A) (*E*)-X_4_ derivatives and (B) (*E*)-X_2_,*m*-Y_2_ in DMSO at 25 °C.

The UV-vis spectra of the (*E*)-X_4_ derivatives and (*E*)-X_2_,*m*-Y_2_ are shown in Fig. 3 (analogous spectra for the *Z* isomers, and deconvoluted spectra are available in the ESI†). Tetra-*ortho* substitution proved to be a reliable method of red-shifting the n → π* absorbance: within each group, increasing atomic size leads to red-shifting of the n → π* absorption maximum. The heavier atoms exhibit deeply redshifted tails (>650 nm for X = I and >700 nm for X = Te), attributed to increased steric clash between the larger element with the n orbital in planar conformations (Fig. 3b). In contrast, for (*E*)-X_2_,*m*-Y_2_, (*E*)-Cl_2_,*m*-Y_2_ and (*E*)-(SEt)_2_,*m*-Y_2_ derivatives, it was found that although the *meta* auxochrome (Y = OMe and NMe_2_) broadened and increased the absorbance of the n → π* transition, the tail did not generally extend much further than the R = H derivatives; with absorptions not exceeding 600 nm (Fig. 3a). The n → π* transition maximum, *λ*_max_, typically followed the order *meta*-H > –NMe_2_ > –OMe and *ortho*-S > Cl > F. It is clear from these experiments that the four *ortho* X substituents in the X_4_ derivatives are essential for achieving the deeply red-shifted tails which allows for red/near-IR triggered photo-isomerisation.

**Fig. 4:**
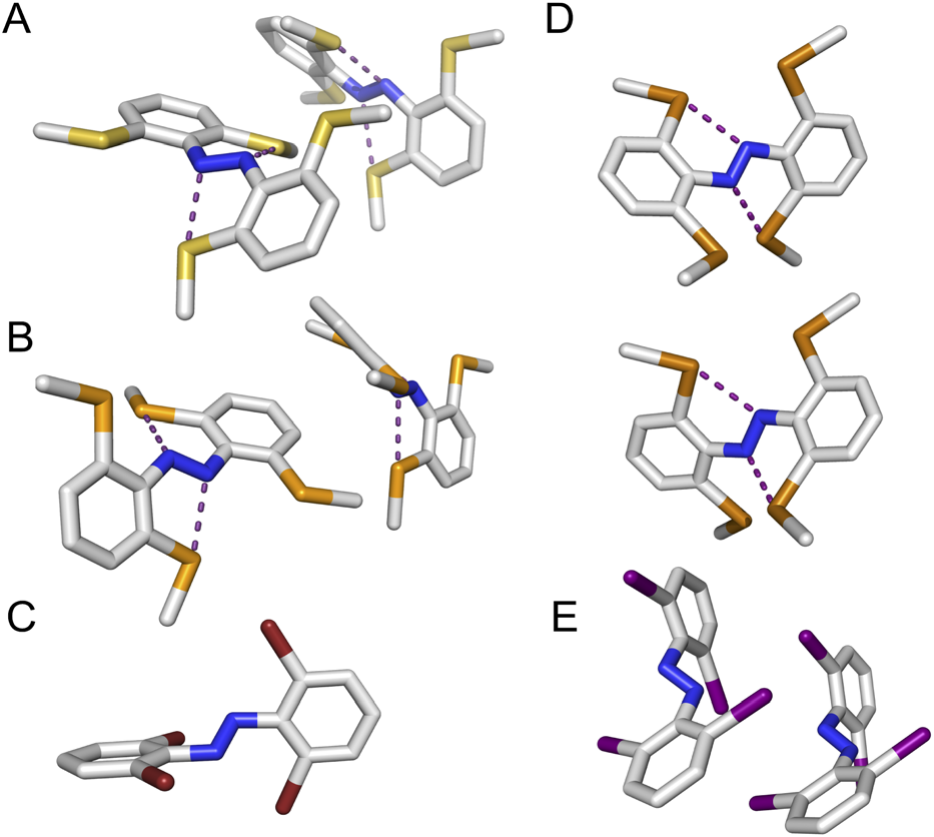
Crystal structures of novel tetra-*ortho* substituted azobenzene scaffolds (*para* substituents not shown for clarity). (A) (SMe)_4_-H (B) (SeMe)_4_-H (C) Br_4_-NHBoc (D) (TeMe)_4_-H (E) I_4_-N_3_. Intramolecular chalcogen bonds forming 5-membered rings between the chalcogen and azo N atoms represented with purple dotted lines. For A, B, D and E two conformations are present within the unit cell.

To explore the role of the *ortho*-substituents on the azobenzene structure, we grew single crystals of the novel tetra-*ortho* derivatives and analysed their structures by X-ray crystallography (Fig. 4 – see ESI† for experimental procedures and diffraction data). Structures for the known F_4_,^42^ Cl_4_ ^29^ and (OMe)_4_ ^15^ derivatives were accessed from the Cambridge Crystallographic Data Centre (CCDC).

**Fig. 5:**
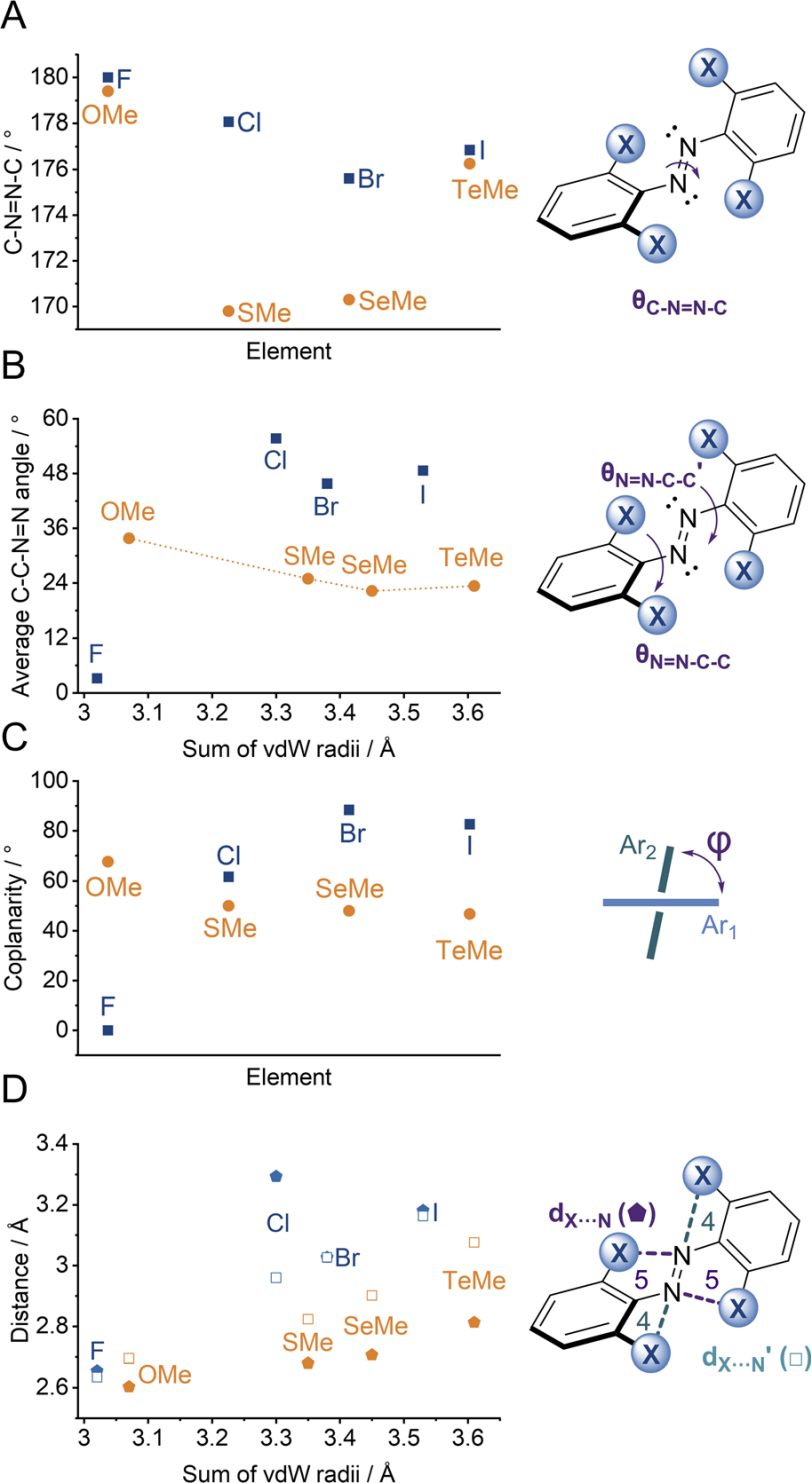
Geometric parameters from single-crystal data for (*E*)-X_4_ (A) C–N=N–C dihedral, (B) average N=N–C–C dihedral, (C) coplanarity of rings, (D) distances between diazo lone pair and X-atom (formation of intramolecular 5 ( <svg xmlns="http://www.w3.org/2000/svg" version="1.0" width="18.545455pt" height="16.000000pt" viewBox="0 0 18.545455 16.000000" preserveAspectRatio="xMidYMid meet"><metadata> Created by potrace 1.16, written by Peter Selinger 2001-2019 </metadata><g transform="translate(1.000000,15.000000) scale(0.015909,-0.015909)" fill="currentColor" stroke="none"><path d="M400 760 l0 -40 -40 0 -40 0 0 -40 0 -40 -80 0 -80 0 0 -40 0 -40 -40 0 -40 0 0 -40 0 -40 40 0 40 0 0 -40 0 -40 -40 0 -40 0 0 -40 0 -40 40 0 40 0 0 -80 0 -80 40 0 40 0 0 -80 0 -80 280 0 280 0 0 40 0 40 40 0 40 0 0 160 0 160 40 0 40 0 0 80 0 80 -40 0 -40 0 0 40 0 40 -40 0 -40 0 0 40 0 40 -80 0 -80 0 0 40 0 40 -120 0 -120 0 0 -40z"/></g></svg> ) and 4 (□) membered rings).

We observed multiple conformations within the same unit cell for tetra -thio, -seleno, -telluro and -iodo derivatives, as has been observed previously by Trauner and co-workers for the tetra chloro azobenzenes.^29^ This provides evidence for the high flexibility of the *E* isomers, which are able to sample a range of conformations including the more planar, redshifted geometries. The geometries of the various structures across the tetra-*ortho* series are compared in Fig. 5. In general, the diazo bond is more twisted for the (heavier) chalcogens (Fig. 5a), perhaps to accommodate the intra-molecular 5-membered ring chalcogen bond. Increased N=N distortion and longer N–N bonds leads to more redshifted wavelengths for the tetra-chloro derivatives, based on previous theoretical calculations,^29^ which presumably contribute here to the high absorbance at longer wavelengths of the chalcogen derivatives.

Generally, each aryl ring is independently twisted away from the diazo bond (dihedral N=N–C–C ≠ N=N–C–C′), in line with the proposed conformational flexibility of the *E* isomers. The average N=N–C–C dihedral and coplanarity of the aryl rings generally decreases down the chalcogen group despite the increasing atomic size, whereas in contrast, it broadly increases down the halogen group (Fig. 5b and c). This is consistent with the chalcogens enforcing planar character *via* an intramolecular chalcogen bond. Further evidence for this X⋯N chalcogen bonding interaction can be inferred from the inherent preference for 5-membered intramolecular X⋯N interactions over 4-membered (*d*_X⋯N_ < *d*_X⋯N′_), where the highly directional chalcogen bond overlap is more feasible (Fig. 5d).^43^ Conversely, the halogens have no preference between 4- or 5-membered interactions because the halogen centred σ-hole is on the pole of the halogen atom and therefore cannot interact with the azo N *via* a linear halogen bond. Conformations where steric clash is minimised are therefore favoured (*d*_X⋯N_ ≈ *d*_X⋯N′_).

**Fig. 6:**
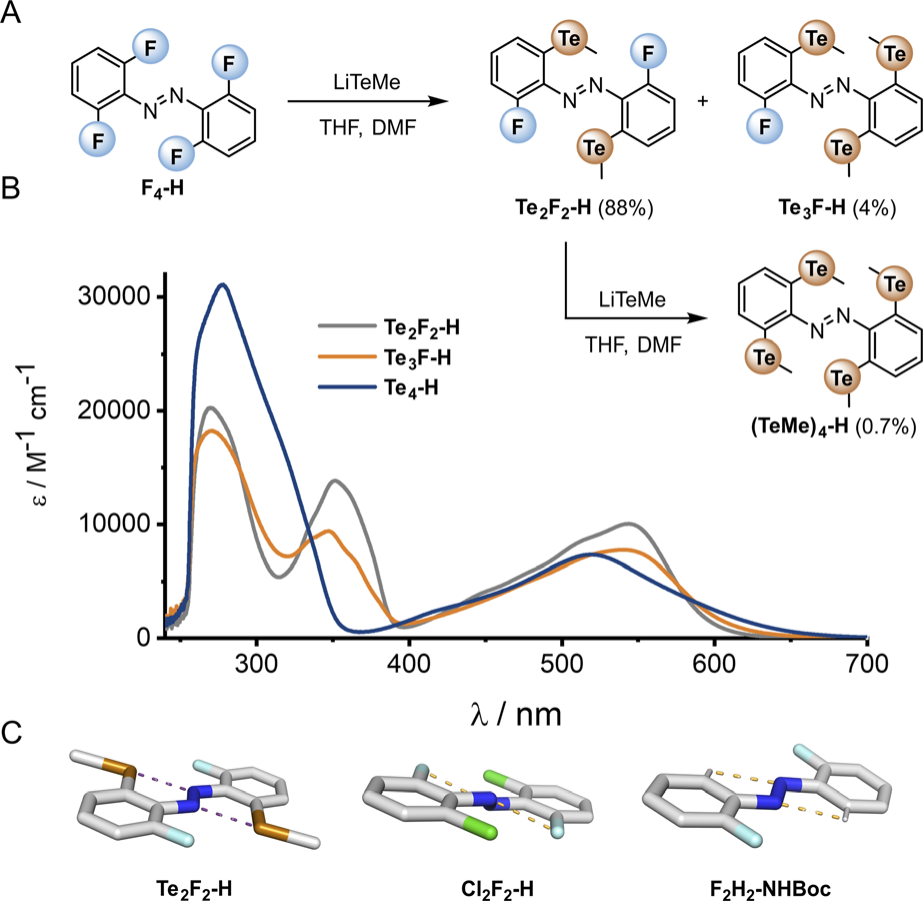
(A) Preparation of tellurium-substituted azobenzenes *via* an S_N_Ar reaction of the corresponding fluoro-derivative. (B) UV-vis spectra of Te derivatives. (C) Crystal structures: Cl_2_F_2_ structure accessed from CCDC,^29^F_2_H_2_-NHBoc determined in-house (*para* substituent omitted for clarity).

The di-, tri- and tetra-telluro derivatives (Te_2_F_2_-H, Te_3_F-H and Te_4_-H) were prepared *via* an unprecedented nucleophilic aromatic substitution of the methyl-telluride on the corresponding fluoro-azobenzene derivative (Fig. 6A). UV-vis spectra of di, tri and tetra ortho tellurides reveal that with increasing number of *ortho*-TeMe substituents, the n → π* *λ*_max_ is blue-shifted, but the tail of the absorption extended further into the red/near IR region (Fig. 6B). The solid-state structure of Te_2_F_2_-H (Fig. 6C), revealed that this di-telluride is completely planar, owing to the strong 5-membered Te⋯N chalcogen bond (2.72 Å), well within the sum of the N–Te van der Waals radii (3.61 Å; 75%). When Te is exchanged to Cl (Cl_2_F_2_-H)^26^ or H (F_2_H_2_-NHBoc), the 5-membered interaction between the azo-N atoms and the *ortho*-substituents is biased to the smallest substituent to minimise repulsion with the lone pairs. Notably, Cl_2_F_2_-H adopts a more twisted geometry than Te_2_F_2_-H despite the smaller size of Cl relative to Te (Fig. 6C); serving as further evidence for strong intramolecular chalcogen bonding within the *ortho*-telluro ABs.

Analysis of the UV-vis spectra and data from the crystal structures suggests that the di-telluride Te_2_F_2_-H exhibits an optimal balance of intra-molecular chalcogen bonds and steric bulk to enforce planarity, because it can form two strong chalcogen bonds without any destabilising steric interactions arising from extra tellurium atoms. This leads to Te_2_F_2_-H exhibiting the most red-shifted n → π* *λ*_max_. However, as with the other heavy *ortho* main group ABs, the derivatives with additional Te atoms also appear to have minor populations of planar conformations, leading to a deeply red-shifted tail of the n → π* transition.

### Photo-isomerisation and glutathione stability

**Fig. 7:**
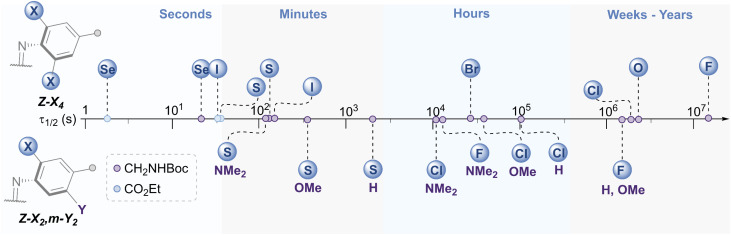
Schematic timeline showing the range of half-lives for the *Z* → *E* thermal isomerisation of the azobenzene derivatives.

The *E* → *Z* photo-isomerisation and half-life for the *Z* → *E* thermal isomerisation was evaluated for all derivatives (Fig. 7 and Table 1, see ESI† for original data). To our surprise, the half-lives for (*Z*)-X_2_,*m*-Y_2_ derivatives were in the order of hours to days for X = F and Cl: the effect of the *meta* group was not as drastic as anticipated. We typically observed a ∼4 fold decrease in half-life by substituting H with OMe, and a further 3-fold drop from OMe to NMe_2_ for X = F, Cl and SEt. The nature of the *ortho*-substituent appeared to be the main influence on *τ*_1/2_ (F > Cl ≫ SEt). However, these predictable trends observed for the *meta*-substitution allow for precise tuning of thermal half-lives. The photo-stationary states (PSSs) of these derivatives ranged from 48–77% *Z* isomer (Table 1). We also prepared the di-*ortho*, mono-*meta*-methoxy derivative, OMe_2_,*m*-OMe_2_, however this was found to be unstable to light (ESI Fig. S359†).

**Fig. 8:**
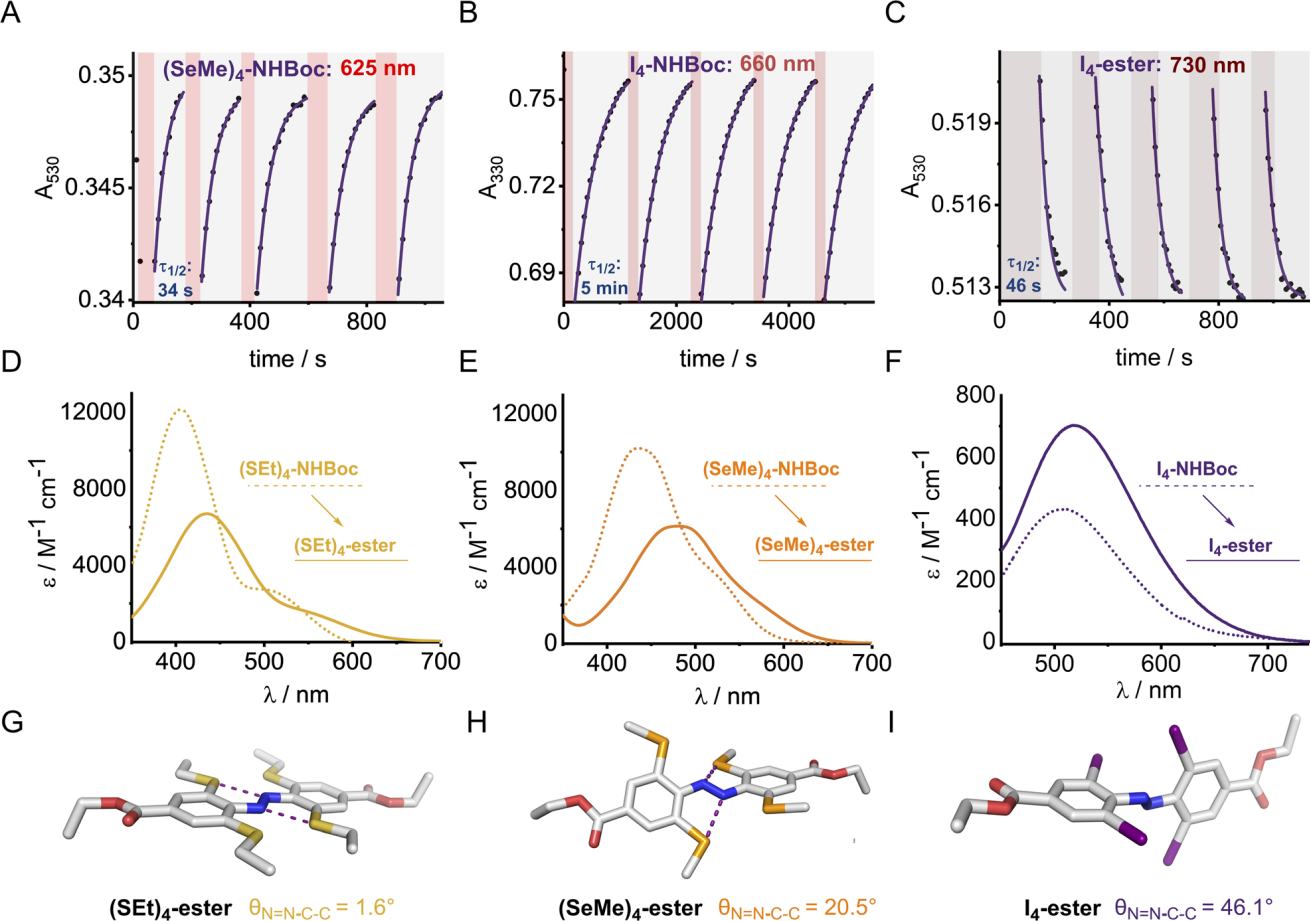
Reversible photo-switching for tetra *ortho*-substituted azobenzene derivatives: (A) (SeMe)_4_-NHBoc with 625 nm, (B) I_4_-NHBoc with 660 nm and (C) I_4_-ester with 730 nm irradiation. Data point: absorbance at the indicated wavelength, solid line: 1^st^ order exponential fit. Red shaded region indicates time period of irradiation with an LED of the indicated wavelength. Samples of ∼100 μM were irradiated with LEDs (625 nm, ∼0.9 W; 660 nm, ∼1.1 W, 730 nm ∼0.7 W). (D–F) Stacked UV-vis spectra of S, Se and I derivatives (G–I) Solid-state structures of *para*-ester derivatives with average aryl-N=N dihedral angles.

The X_4_ derivatives exhibited more interesting photo-switching properties, with heavier elements displaying robust, reversible switching behaviour using solely red light and thermal decay, with half-lives for the *Z* → *E* thermal isomerisation of the order of seconds to minutes (Fig. 8A and B). Remarkably, it was possible to photo-switch the tetra-iodo derivative I_4_-ester using near-IR light (730 nm) (Fig. 8C). Notably, these thermal half-lives can be tuned over seven orders of magnitude within this isostructural series and each derivative is amenable to further functionalisation. Photo-isomerisation was not detected for the *ortho*-telluro derivatives. This is consistent with findings by Haberhauer and co-workers, who propose that the strong chalcogen bonds in the *E* isomer of a PhTe-substituted azobenzene lead to dramatic energy differences between the *E* and *Z* isomers, which suppresses photoswitching.^43^ The photo-stationary states of the halogen X_4_ derivatives range from 50–84% (Table 1) and could also be determined for the slower relaxing chalcogen derivatives (PSS 46–95%, see Fig. S308–S323† for original data).

The half-lives of the (*Z*)-X_4_ derivatives decrease down each group (Table 1, entries 2, 4, 5, 10 and 13, 16, 21, 24), consistent with electronegative substituents relieving the N=N lone pair repulsion and stabilising the overall *Z* isomer energy.^14^ For X = F, anomalously high *Z* isomer stability has been previously suggested to arise from intramolecular C⋯F interactions.^44^ Increasing the steric bulk of the alkyl group appended to sulfur from ethyl to iso-propyl blueshifts the switching transition, presumably due to steric clash that distorts from planarity outweighing the chalcogen bonding interactions (entry 16 → 18). This structural change does not affect the thermal half-life of the *Z*-isomer.

In general, switching from a *para*-H substituent to a methylene group lowers the half-life around 2-fold, most likely due to increased electron density of the azobenzene.^45^ Switching to an ester group significantly lowers the half-life for each derivative (∼4–10-fold), consistent with previously reported systems, and rationalised by π-acceptors stabilising the linear Ar–N–N–Ar transition state during thermal isomerisation.^16^ Furthermore, the ester group typically allows switching with more deeply red-shifted wavelengths and acts as a potential functional handle for further derivatisation (Fig. 8D–F). These deeply red-shifted tails may be attributed to increased average planarity to maximise conjugation between the diazo bond and ester. Indeed, for the S_4_ derivatives, the ester motif has a dramatic effect on planarity relative to (SMe_4_)-H in the solid state (Fig. 8G), with an essentially planar structure observed. For the seleno and iodo derivatives, the decrease in planarity is less dramatic in the solid-state crystal structures, presumably due to competing steric effects with the larger atoms (Fig. 8H–I). Combining tetra-*ortho* substitution with a *meta*-methoxy substituent lead to dyes that were unstable to light (entry 14, 19 see ESI S68 and S363†). ABs with *ortho*-phospho and amino groups were also found to decompose in the presence of light, and thus are not suitable candidates for photo-switching applications (entry 36–37, S364–S365).†

The (SeMe)_4_, (SEt)_4_, and I_4_ derivatives display thermal half-lives in the order useful for spatial targeting applications (second to minute range). Crucially, these photo-switches may be reversibly switched between both isomers using only red wavelengths of light (*E* → *Z*) and thermal isomerisation (*Z* → *E*). Furthermore, there is scope for selectively switching one scaffold in the presence of another with orthogonal wavelengths. For instance, (SeMe)_4_-NHBoc does not switch with 660 nm light, whereas I_4_-NHBoc or I_4_-CO_2_Et derivatives readily isomerise under these conditions.

Finally, the stability of the X_4_ derivatives were assessed by exposing them to the highest expected intracellular concentration of glutathione (GSH, 10 mM).^28^ This was achieved by treating the Boc-protected amino-methyl derivatives X_4_-NHBoc (X = F, Cl, Br, I, OMe, SMe and SeMe) with TFA to remove the protecting groups. The resulting ammonium-appended AB derivative was dissolved in N_2_-degassed 100 mM phosphate buffer at pH 7 containing 10 mM GSH, and the changes in absorbance were monitored over an extended period. Generally, the larger, less electron withdrawing derivatives are more stable under these conditions, which is expected based on the substituent's ability to polarise the diazo bond towards nucleophilic attack, and steric arguments. Strong σ-donating substituents are also proposed to aid diazo lone pair protonation, and subsequent GSH attack.^25^ Notably, the seleno derivatives exhibit stability for days, advantageous for applications in cellular environments, whereas the lighter elements generally decay in the order of hours (Table 2). The I_4_ derivative displays similar stability to the F_4_ analogue.

**Table 2:** Stability to glutathione (GSH) of X_4_ structures

X^a^	*τ*_1/2_ (10 mM GSH)
F	0.7 h
Cl	2.7 h
Br	5 h
I	0.7 h
OMe	2 h
SMe	6.1 h
SeMe	168 h

a *Para* substituent = CH_2_NH_2_·TFA. Conditions: 100 mM phosphate buffer containing 10 mM GSH, degassed with N_2_. *T* = 298 K.

## Conclusion

We report the synthesis of heavy main group element substituted azobenzenes, revealing robust periodic trends in absorption and thermal half-lives within the tetra-*ortho*-chalcogen and halogen substituted series. We observe that the unprecedented azobenzenes substituted with the heavier (less electronegative) elements exhibit shorter half-lives for the thermal *Z* to *E* isomerisation and more redshifted n → π* bands. We provide a uniquely comprehensive study on the *ortho*-substituent effects in these systems, and demonstrate that introduction of *meta*-electron donating groups could be used to fine-tune the thermal half-lives, whilst *para*-electron withdrawing groups further red-shift the absorption, and decrease the thermal stability of the *Z* isomer. In particular, the sulfur, seleno and iodo derivatives display short (second to minute) half-lives, and are reversibly switched using red light, with no evidence of photobleaching. The corresponding tetra-*ortho*-iodo, *para*-ester derivative could be reversibly switched using near-IR irradiation and thermal relaxation. The red-shifted seleno derivative also exhibits excellent stability to the cellular reducing agent glutathione. This work provides a comprehensive point of reference for red-shifted azobenzene design, and we anticipate these tuneable, robust and biocompatible photo-switches will find utility within the field of photo-pharmacology, and other applications in which the precise tuning of photo-switching and thermal relaxation, within otherwise isostructural molecular switches, is desired.

## Author contributions

A. K. carried out the experimental work. K. E. C. performed the X-ray crystallography experiments. M. J. L. conceived and directed the project. All authors contributed to the discussion and prepared the manuscript.

## Conflicts of interest

There are no conflicts to declare.

## Supplementary Material

SC-013-D2SC04601F-s001

SC-013-D2SC04601F-s002
