# Supplementary material for: Fast relaxing red and near-IR switchable azobenzenes with chalcogen and halogen substituents: periodic trends, tuneable thermal half-lives and chalcogen bonding
Source: Chem Sci. 2022 Sep 20;13(39):11551–9. doi: 10.1039/d2sc04601f (PMC9555560; doi:10.1039/d2sc04601f)
Supplement: SC-013-D2SC04601F-s001 [file SC-013-D2SC04601F-s001.pdf]

Electronic Supplementary Material (ESI) for Chemical Science.
This journal is © The Royal Society of Chemistry 2022

Supporting Information for: Fast relaxing red and near-IR switchable azobenzenes with heavy chalcogen and halogen substituents: periodic trends, tuneable thermal half-lives and chalcogen bonding

Aidan Kerckhoffs, Kirsten E. Christensen and Matthew J. Langton*

Contents

1. Materials and methods 2
2. Synthesis and characterization 3
3. UV-Vis spectra and Photo-switching experiments 151
4. Photoswitching: PSS and Half-Life Determination 187
5. GSH stability studies 215
6. Single crystal X-ray diffraction experiments 219
7. References 234

1. Materials and methods

All reagents and solvents were purchased from commercial sources and used without further purification. Where necessary, solvents were dried by passing through an MBraun MPSP-800 column and degassed with nitrogen. Triethylamine was distilled from and stored over potassium hydroxide. Column chromatography was carried out on Merck® silica gel 60 under a positive pressure of nitrogen. Where mixtures of solvents were used, ratios are reported by volume. NMR spectra were recorded on a Bruker AVIII 400, Bruker AVII 500 (with cryoprobe), Bruker NEO 600 with broadband helium cryoprobe and Bruker AVIII 500 spectrometers. Chemical shifts are reported as δ values in ppm. Mass spectra were carried out on a Waters Micromass LCT and Bruker microTOF spectrometers. UV-Vis spectra were recorded on a V-770 UV-Visible/NIR Spectrophotometer equipped with Peltier temperature controller and stirrer using quartz cuvettes of 1 cm path length. Experiments were conducted at 25°C unless otherwise stated.

Abbreviations

Boc: tert-butyloxycarbonyl; BINAP: (2,2′-bis(diphenylphosphino)-1,1′-binaphthyl); DBDMH: 1,3-Dibromo-5,5-Dimethylhydantoin; DBU: 1,8-Diazabicyclo[5.4.0]undec-7-ene; DCM: Dichloromethane; DMAP: 4-Dimethylaminopyridine; DMF: *N*,*N*-Dimethylformamide; DMSO: Dimethylsulfoxide; DPPA; Diphenylphosphoryl azide; HRMS: High resolution mass spectrometry; MeCN: Acetonitrile; MeOH: Methanol; NBS: *N*-Bromosuccinimide; NCS: *N*-Chlorosuccinimide; NMP: N-Methyl-2-pyrrolidone; PIDA: phenyliodine(III) diacetate; Phth: Phthaloyl; PSS: Photo-stationary state; rt: Room temperature; TFA: Trifluoroacetic acid; THF: Tetrahydrofuran; TMS: Trimethylsilyl; µW: Microwave

2. Synthesis and characterization

General comments.

All novel compounds were characterised by ^{1}H and ^{13}C NMR, and high-resolution mass spectrometry. Azobenzene derivatives were formed as a mixture of *E* and *Z* isomers. Peaks for the *E* isomer are reported (major product).

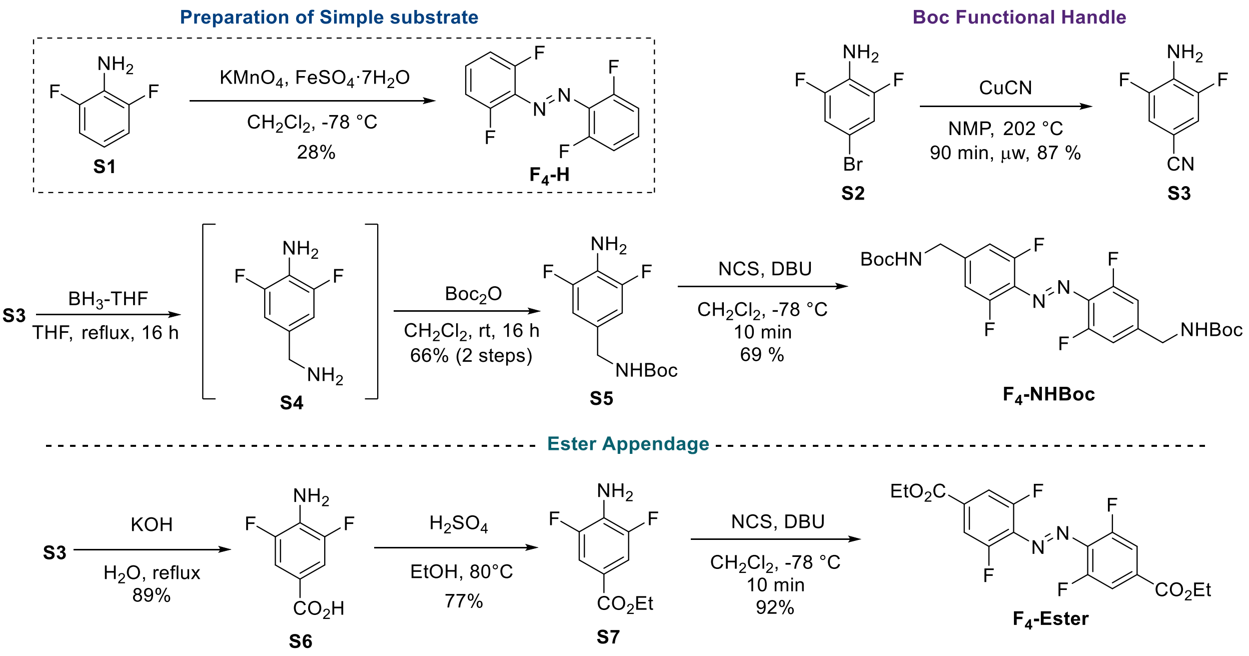

Scheme S1. Synthesis of tetra-*ortho*-fluoro azobenzene derivatives [[1]]

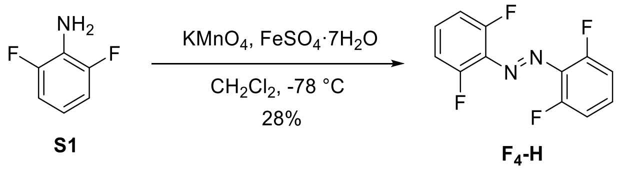

F_4-H. Prepared according to a modified literature procedure.[[2]] 2,6-difluoroaniline **S1** (8 g, 62.0 mmol) was dissolved in CH_2Cl_2 (300 mL) and a freshly ground mixture of $KMnO_4$ (32 g, 202.5 mmol, 3.27 eq) and $FeSO_4{\cdot}7H_2O$ (32 g, 115 mmol, 1.86 eq) were added. The solution was refluxed for 3 days, filtered through celite, dried over $MgSO_4$, filtered, and concentrated under reduced pressure. The crude residue was purified by column chromatography (dry load, 20% CH_2Cl_2 in hexane) to give **F_4-H** as an orange solid (2.2 g, 8.66 mmol, 28%). ^{1}H NMR (400 MHz, $CDCl_3$) δ 7.38 (tt, J = 8.4, 5.8 Hz, 2H), 7.11 – 7.03 (m, 4H). Data consistent with that given in the literature. [[2]]

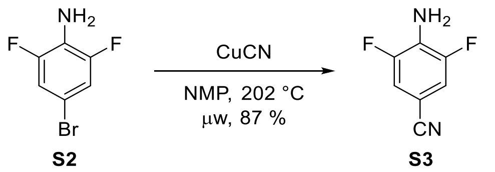

4-amino-3,5-difluorobenzonitrile S3 A suspension of 4-bromo-2,6-difluoroaniline **S2** (6.5 g, 31.1 mmol) and copper(I) cyanide (4.2 g, 146.6 mmol, 1.5 eq) in dry NMP (15 mL) was heated to 202 °C under microwave irradiation for 90 minutes under N_2. The reaction was poured onto 15% ammonia solution (300 mL), then extracted with 50:50 Hexane:EtOAc (3x 150 mL). The combined organic layers were washed with water (5x 150 mL), 5% LiCl solution, then were concentrated in vacuo. The resulting residue was purified by silica gel column chromatography (50% CH_2Cl_2 in hexane) to obtain the title compound as a white crystalline solid (4.16 g, 27 mmol, 87%). ^{1}H NMR (400 MHz, Chloroform-d) δ 7.17 (dd, J = 6.0, 2.3 Hz, 2H), 4.29 (s, 2H). Data consistent with that given in the literature.[2]

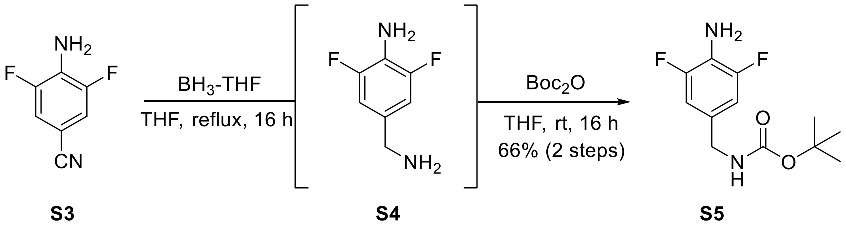

tert-butyl (4-amino-3,5-difluorobenzyl)carbamate S5. To a solution at 0°C of 4-amino-3,5-difluorobezonitrile **S3** (3.85 g, 25 mmol) in THF (20 mL) was added dropwise 1M BH_3-THF solution (100 ml, 100 mmol, 4 equiv). The mixture was stirred under reflux for 16 h. 100 mL of MeOH were then added, and the resulting mixture was refluxed for 1 h. The solvent was removed *in vacuo*. The resulting residue was re-dissolved in EtOAc (300 mL) and washed with water (3 x 50mL) and brine (1 x 50 mL). The organic layers were concentrated *in vacuo* to afford crude amine **S4**. ^{1}H NMR (400 MHz, Acetone) δ 6.97 – 6.77 (m, 1H), 4.29 (s, 1H). This was immediately dissolved in CH_2Cl_2 (60 mL). Boc_2O (5.45g, 25 mmol, 1 equiv) in CH_2Cl_2 (30 mL) was added dropwise to the solution of **7**. The reaction was stirred for 16 h at rt. The solvent was concentrated and the residue was purified by silica gel flash column chromatography (6:1 hexane-acetone) to afford the title compound as a white solid (4.26 g, 66%). ^{1}H NMR (400 MHz, $CDCl_3$) δ 6.82 – 6.69 (m, 2H), 4.80 (s, 1H), 4.17 (d, *J* = 6.1 Hz, 2H), 3.82 – 3.54 (br s, 2H), 1.46 (s, 9H). ^{13}C NMR (101 MHz, $CDCl_3$) δ 155.85, 151.92 (dd, *J* = 240.8, 8.2 Hz), 128.40 (t, *J* = 7.83 Hz), 122.83 (t, *J* = 16.5 Hz), 109.92 (dd, *J* = 15.1, 7.5 Hz), 79.66, 43.67, 28.35. HRMS-ESI (m/z) Calculated for $C_{12}H_{16}O_2N_2F_2$ $[M+Na]^+$, 281.1072; found 281.1072.

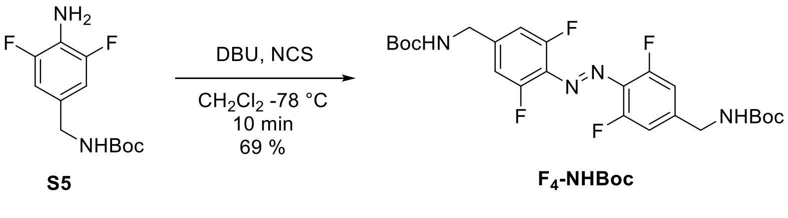

F_4-NHBoc. To a solution of **S5** (303 mg, 1.17 mmol) in CH_2Cl_2 (17 mL) was added DBU (350 mg, 2.34 mmol, 2 eq). The solution was stirred at room temperature for 5 min before being cooled down to −78 °C. NCS (313 mg, 2.34 mmol, 2 eq) was added. The orange solution was stirred for 10 min at −78 °C before quenching with saturated bicarbonate solution (15 mL). The organic layer was separated, washed sequentially with 15 mL of water (5 x 15 2mL) and 1N HCl (15 mL), dried over anhydrous sodium sulfate, and concentrated to dryness in vacuo. The residue was purified by silica gel flash chromatography (5% EtOAc in CH_2Cl_2) to afford the title compound as an orange solid as a mixture of isomers (205 mg, 402 µmol, 69%). *(E)*-**F_4-NHBoc:** ^{1}H NMR (400 MHz, Chloroform-*d*) δ 6.99 (d, *J* = 10.1 Hz, 4H), 4.99 (s, 2H), 4.35 (d, *J* = 6.4 Hz, 4H), 1.47 (s, 18H). ^{13}C NMR (101 MHz, Chloroform-

d) δ 155.95, 155.04 (dd, J = 261.7, 4.53 Hz), 144.60 (m), 130.69 (t, J = 9.8 Hz), 111.14 (d, J = 21 Hz), 80.40, 44.02, 28.50. HRMS-ESI (m/z) Calculated for $C_{24}H_{28}F_4N_4O_4$ $[M+H]^+$, 513.2119; found 513.2119.

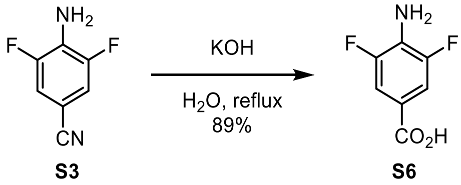

4-amino-3,5-difluorobenzoic acid S6. **S3** (2.5 g, 16.2 mmol) was suspended in 25% KOH in water (50 mL) and was refluxed for 16 hours. Then 2N HCl was added until pH 2 was reached, and the precipitate was extracted with EtOAc. The organic layers were washed with brine, then concentrated to afford the title compound as a salmon-pink solid (2.48 g, 14.4 mmol, 89%). ^{1}H NMR (400 MHz, DMSO) δ 12.51 (s, 1H), 7.40 (d, *J* = 7.2, 2.5Hz, 2H), 6.06 (s, 2H). Data consistent with that given in the literature [[3]]

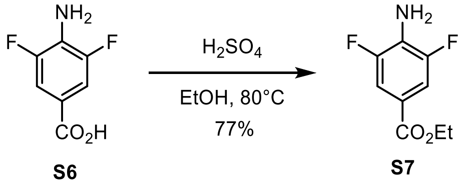

Ester S7. S6 (1 g, 5.78 mmol) was dissolved in EtOH (20 mL). Then sulphuric acid (0.3 mL) was added and the reaction was stirred at 80 °C bath temperature for 16 hours. The reaction was diluted with CH_2Cl_2 and the organic layer washed with sat. bicarbonate solution. The organic layer was dried then concentrated to afford the title compound as an off-white solid (889 mg, 4.42 mmol, 77%). ^{1}H NMR (400 MHz, $CDCl_3$) δ 7.58 – 7.48 (m, 2H), 4.33 (q, *J* = 7.1 Hz, 2H), 3.72 (q, *J* = 7.0 Hz, 2H), 1.37 (t, *J* = 7.1 Hz, 3H). Data consistent with that given in the literature. [2]

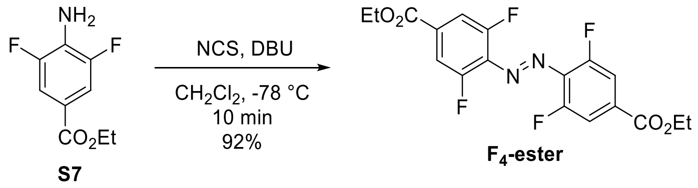

F_4-ester. To a solution of **S7** (1.0 mg, 5.0 mmol) in CH_2Cl_2 (75 mL) was added DBU 1.48 mL, 10.0, 2 eq). The solution was stirred at room temperature for 5 min before being cooled down to −78 °C. NCS (1.33 g, 10.0 mmol, 2 eq) was added. The orange solution was stirred for 10 min at −78 °C before quenching with saturated bicarbonate solution (75 mL). The organic layer was separated, washed sequentially with 75 mL of water and 1N HCl (75 mL), dried over anhydrous sodium sulfate, and concentrated to dryness in vacuo. The residue was purified by silica gel flash chromatography (50% Hexane in CH_2Cl_2) to afford the title compound as an orange solid as a mixture of isomers (905 mg, 2.49 mmol, 92%. *(E)*-**F_4-Ester**: ^{1}H NMR (400 MHz, $CDCl_3$) δ 7.79 – 7.71 (m, 4H), 4.43 (q, *J* = 7.1 Hz, 4H), 1.43 (t, *J* = 7.1 Hz, 6H). Data consistent with that given in the literature. [[2]]

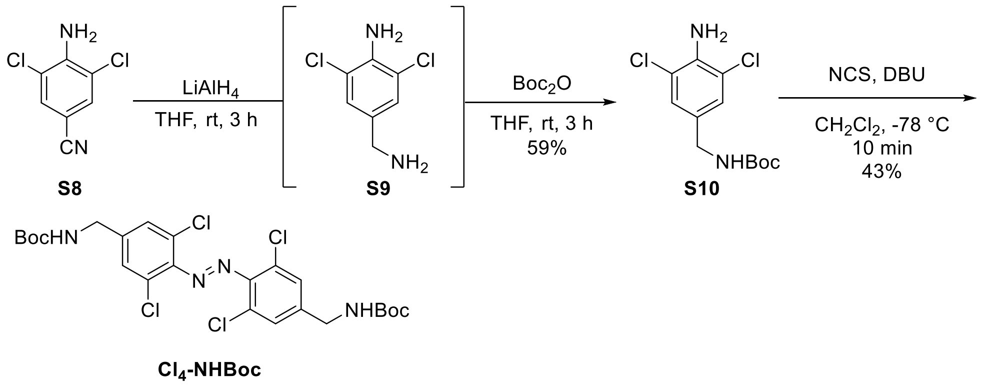

Scheme S2. Synthesis of tetra-*ortho*-chloro azobenzene derivatives [[4]]

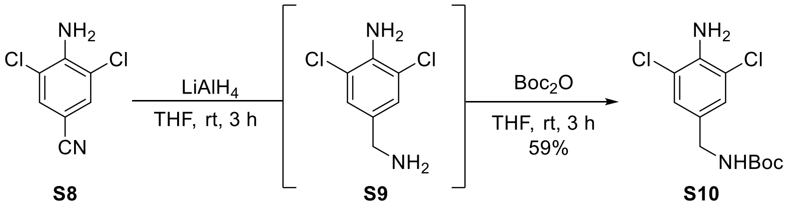

Aniline S10. To a suspension of $LiAlH_4$ (3.62 g, 95.3 mmol, 2.2 eq) in dry THF (90 mL) was added dropwise 3,5-dichloro-4-aminobenzonitrile **S8** (8.1 g, 43.31 mmol, 1.0 eq) in THF (130 mL) at rt. THF (100 mL) was added to break up the thick solids. The suspension was stirred at rt for 3 hours. The reaction mixture was diluted in Et_2O (350 mL), then water (2 mL), 2.5M NaOH (4 mL), and water (8 mL) was added dropwise. The suspension was stirred for 15 minutes, and then anhydrous $MgSO_4$ was added. The solution was filtered, and filtrate concentrated. The off-white solid **S9** was redissolved in THF (75 mL). Boc_2O (8.4 g, 38.5 mmol, 1.05 eq) was added and the reaction was stirred for 3 hours. The solution was concentrated and purified by silica gel flash chromatography (18% Acetone in hexane), then recrystallized from Et_2O and petroleum ether to afford the title compound (7.4 g, 3.37 mmol, 59% over two steps). ^1H NMR (400 MHz, Chloroform-*d*) δ 7.11 (s, 2H), 4.78 (s, 1H), 4.51 – 4.28 (br s, 2H), 4.15 (d, J = 6.0 Hz, 2H), 1.46 (s, 9H). ^{13}C NMR (126 MHz, $CDCl_3$) δ 155.89, 139.37, 129.40, 127.13, 119.70, 79.88, 43.64, 28.35. HRMS-ESI (m/z) Calculated for $C_{12}H_{16}Cl_2N_2O_2$ $[M+H]^+$, 291.0661; found 291.0662.

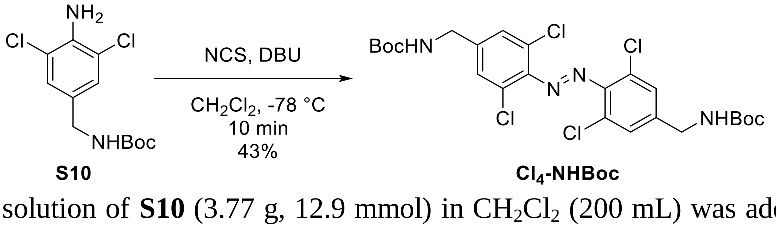

Cl_4-NHBoc. To a solution of **S10** (3.77 g, 12.9 mmol) in CH_2Cl_2 (200 mL) was added DBU (3.95 g, 25.9 mmol, 2 eq). The solution was stirred at room temperature for 5 min before being cooled down to −78 °C. N-Chlorosuccinimide (3.45 g, 25.9 mmol, 2 eq) was added. The dark solution was stirred for 10 min at −78 °C before quenching by addition of a saturated bicarbonate solution (200 mL). The organic layer was separated, washed sequentially with water (3 x 200 mL) and 1M HCl (200 mL) and concentrated to dryness in vacuo. The residue was purified by silica gel flash chromatography (1 to 3% EtOAc in CH_2Cl_2) to afford the title compound as a brown solid as a mixture of isomers (1.61 g, 5.55 mmol, 43%. (*E*)**-Cl_4-NHBoc:** ^1H NMR (400 MHz, Chloroform-d) δ 7.37 (s, 4H), 4.99 (br s, 2H), 4.32 (d, J = 6.2 Hz, 4H), 1.48 (s, 18H) ^{13}C NMR (126 MHz, DMSO-d_6) δ 155.80, 144.97, 144.32, 128.11, 126.13, 78.34, 42.36, 28.16. HRMS-ESI (m/z) Calculated for $C_{24}H_{28}Cl_4N_4O_4$ $[M+H]^+$, 577.0937; found 577.0936.

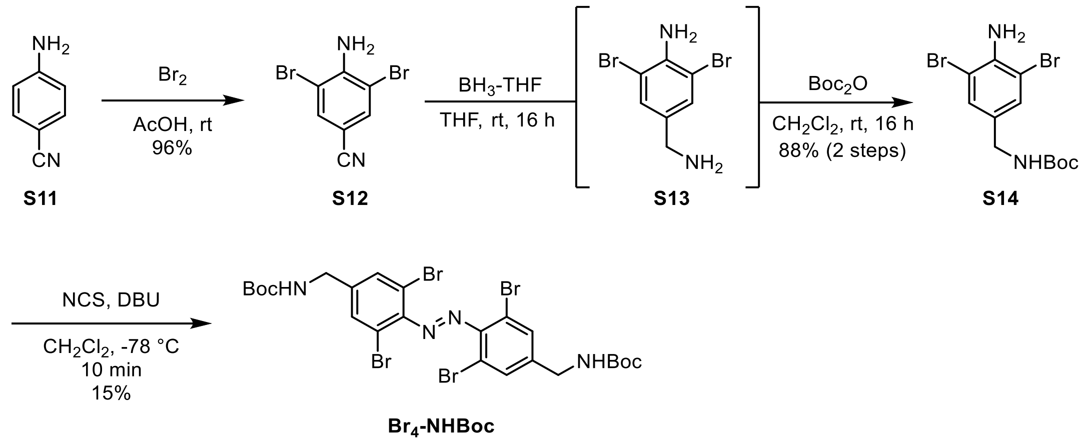

Scheme S3. Synthesis of tetra-*ortho*-bromo azobenzene derivative **Br_4-NHBoc**.

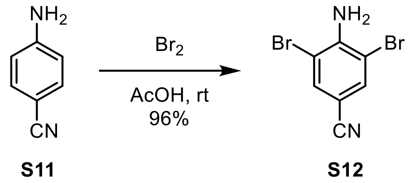

Dibromoaniline S12. To a solution of **S11** (2 g, 16.9 mmol) in AcOH (140 mL) was added Br_2 (1.91 mL, 37.2 mmol, 2.2 eq) dropwise. The reaction was stirred for 30 minutes at room temperature, then poured onto ice water (350 mL). The solid was collected via filtration, washed with sodium thiosulfate, then water. The filter cake was dried in vacuo to afford the title compound as an off-white solid (4.47 g, 16.2 mmol, 96%). ^1H NMR (400 MHz, $CDCl_3$) δ 7.66 (s, 2H), 5.11 (s, 2H). Data consistent with that given in the literature. [[5]]

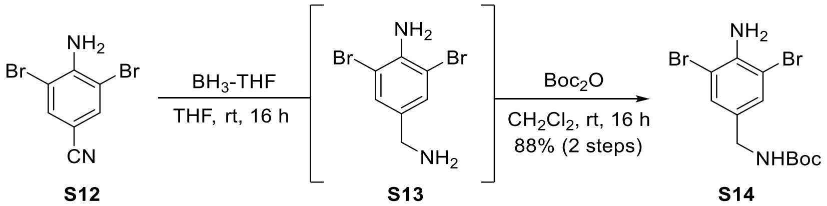

S14. S13 (1 g, 3.6 mmol) was dissolved in THF (50 mL). Then, Borane-THF (1M, 14.5 mmol, 4 eq) was added at 0 °C, which was allowed to warm up to room temperature. The reaction was stirred at this temperature for 16 hours, then 10% HCl (3 mL) was added slowly, forming a precipitate. The reaction was heated to 50 °C for 30 min, then cooled. The mixture was basified with 10% NaOH, then extracted with EtOAc (3x). The reaction was concentrated to afford the intermediate amine **S13**. ^1H NMR (400 MHz, $CDCl_3$) δ 7.34 (s, 2H), 4.48 (s, 2H), 3.72 (s, 2H). The residue was dissolved in CH_2Cl_2 (30 mL), and Boc_2O (790 mg, 3.62 mmol, 1 eq) in CH_2Cl_2 (10 mL) was added dropwise. The reaction was stirred at room temperature for 16 hours, then concentrated. The resulting residue was purified by silica gel flash chromatography (CH_2Cl_2) to afford the title compound a white solid (1.21 g, 3.17 mmol, 88%). ^1H NMR (400 MHz, $CDCl_3$) δ 7.29 (s, 2H), 4.85 (s, 1H), 4.48 (s, 2H), 4.13 (d, *J* = 5.4 Hz, 2H), 1.45 (s, 9H). ^{13}C NMR (101 MHz, $CDCl_3$) δ 155.85, 141.25, 131.03, 130.55, 108.80, 79.85, 43.37, 28.51. HRMS-EI (m/z) Calculated for $C_{12}H_{16}O_2N_2Br_2Na$ $[M+Na]^+$, 402.9450; found 402.9449.

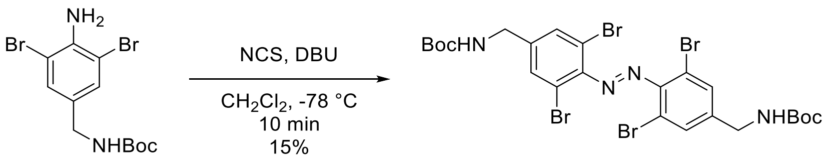

S14 **Br_4-NHBoc**

Br_4-NHBoc. To a solution of **S14** (211 mg, 555 µmol) in CH_2Cl_2 (8 mL) was added DBU (167 µL, 1.11 mmol, 2 eq). The solution was stirred at room temperature for 5 min before being cooled down to −78 °C. NCS (148 mg, 1.11 mmol, 2 eq) was added. The orange solution was stirred for 30 min at −78 °C before quenching by addition of a saturated bicarbonate solution (10 mL). The organic layer was separated, washed sequentially with 10 mL of water (3 x) and 10 mL of 1 N HCl, and concentrated in vacuo. The residue was purified by silica gel flash chromatography (0 to 1% acetone in CH_2Cl_2) to afford the title compound as an orange solid (31.2 mg, 41.0 µmol, 15%). (*E*)-**Br_4-NHBoc:** ^{1}H NMR (600 MHz, $CDCl_3$) δ 7.63 (s, 4H), 4.98 (s, 2H), 4.36 (d, *J* = 6.2 Hz, 4H), 1.51 (s, 18H). ^{13}C NMR (151 MHz, $CDCl_3$) δ 155.76, 147.80, 142.29, 132.04, 116.28, 80.24, 43.26, 28.38. HRMS-EI (m/z) Calculated for $C_{24}H_{29}O_4N_4Br_4$ $[M+H]^+$, 756.8878; found 756.8870.

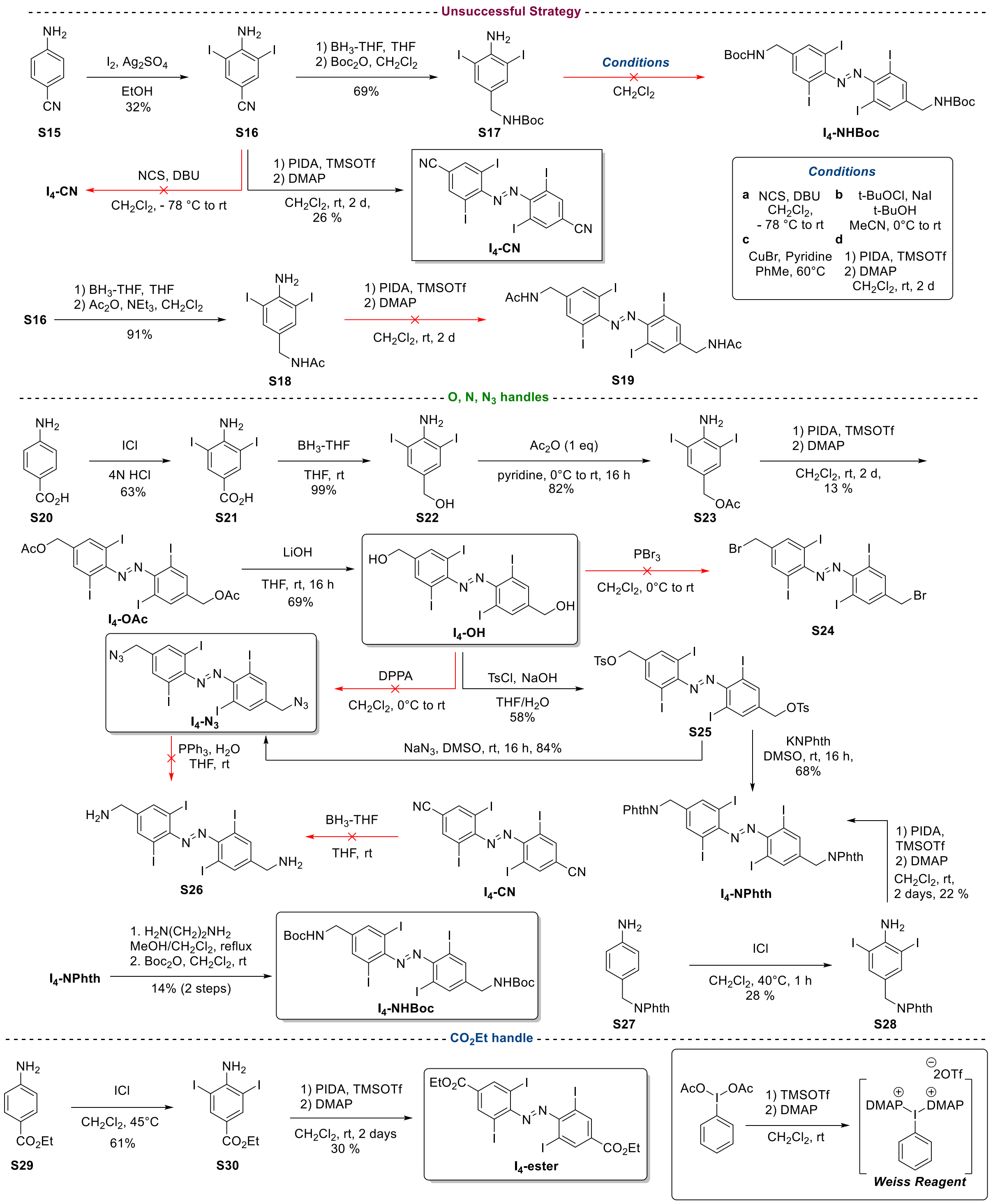

Scheme S3. Synthesis of I_4 derivatives.

S17 was prepared from iodination of **S15** followed by reduction and boc protection. A variety of azo coupling conditions were screened to dimerise **S17**: NCS/DBU and CuBr/Pyridine[6] resulted in recovery of **S17**. Reaction with *in-situ* generated tert-butyl hypoiodate[7] or Weiss' Reagent[8] did not result in any product formation. It was necessary to use different protecting groups for successful dimerization using Weiss' reagent, as this was sensitive to the para substituent (CN, CO_2Et, CH_2OAc and CH_2NPhth are tolerated, yet those containing an NH group were not tolerated; CH_2NHAc **S18** and CH_2NHBoc)**. I_4-OAc** (derived from **S20-S23**) was used to access alcohol **I_4-OH**, azide **I_4-N_3** and ultimately **I_4-NHBoc** and **I_4-NPhth**. **I_4-NPhth** can alternatively be accessed via **S27** and **S28**. It was

not possible to reduce **I_4-N_3** or **I_4-CN** to generate **S26**. **I_4-OH** did not tolerate PBr_3; this resulted in C-I bond reduction. **I_4-ester** can conveniently be accessed from **S29-S30**.

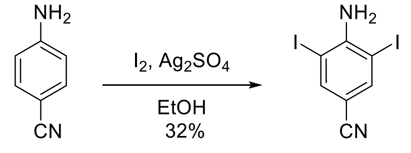

S16. Nitrile **S15** (25.39 mmol) was added to a mixture of Iodine (12.89 g, 50.8 mmol, 2 eq) and silver (I) sulfate (15.84 g, 50.8 mmol, 2 eq) in EtOH (210 mL). The reaction was stirred vigorously at room temperature. The reaction was filtered over a celite pad, then washed with EtOAc. The filtrate was concentrated, then redissolved in CH_2Cl_2 and subsequently washed with 1N NaOH, then water and dried over magnesium sulfate. The dark brown mixture was dissolved in hot EtOH (120 mL), then filtered. The filtrate was cooled to room temperature. The formed crystals were collected and washed with cold ethanol and pentane to afford the title compound as brown/purple crystals (3 g, 8.1 mmol, 32%). ^{1}H NMR (400 MHz, $CDCl_3$) δ 7.88 (s, 2H), 5.17 (s, 2H). Data consistent with that given in the literature. [[9]]

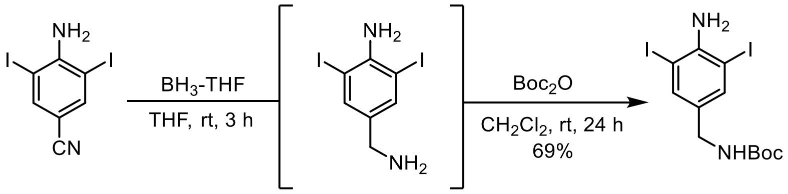

Building Block S17. **S16** (1 g, 2.7 mmol) was dissolved in THF (20 mL). Borane-THF (1M, 10.8 mL, 10.8 mmol, 4 eq) was added at 0 °C under N_2 atmosphere, and the reaction was stirred for 16 hours at rt. 5% HCl (5 mL) was added dropwise at this temperature until effervescence stopped, and the reaction was refluxed for 30 minutes. The reaction was basified with NaOH (1M), and the mixture was extracted with EtOAc (3x), then concentrated to afford the crude amine. ^{1}H NMR (400 MHz, $CDCl_3$) δ 7.59 (s, 2H), 4.54 (s, 2H). The off-white solid was redissolved in CH_2Cl_2 (20 mL). Boc_2O (590 mg, 2.7 mmol, 1.0 eq) in CH_2Cl_2 (10 mL) was added dropwise, and the reaction was stirred for 16 hours at room temperature. The solution was concentrated and purified by silica gel flash chromatography (70% CH_2Cl_2 in hexane) to afford the title compound as a white solid (818 mg, 1.86 mmol, 69%). ^{1}NMR (600 MHz, $CDCl_3$) δ 7.55 (s, 2H), 4.76 (s, 1H), 4.58 (br s, 2H), 4.11 (d, *J* = 5.8 Hz, 2H), 1.46 (s, 9H). ^{13}C NMR (151 MHz, $CDCl_3$) δ 155.84, 145.54, 138.74, 132.09, 81.37, 79.90, 42.89, 28.54. HRMS-EI (m/z) Calculated for $C_{12}H_{16}N_2O_6I_2$ $[M+H]^+$, 496.9193; found 496.9193.

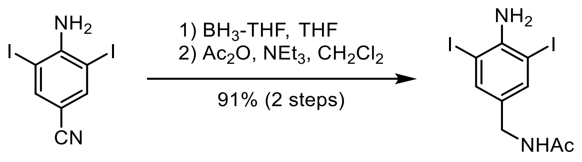

S16 **S18**

S18. 4-amino-3,5-diiodobenzonitrile (600 mg, 1.62 mmol) was dissolved in THF (20 mL). Borane-THF (1M, 6.5 mL, 6.5 mmol, 4 eq) was added at 0 °C under N_2 atmosphere, and the reaction was stirred for 16 hours at rt. 10% HCl (1.2 mL) was added dropwise at this temperature until effervescence stopped, and the reaction was refluxed for 30 minutes. The reaction was basified with NaOH (1M), and the mixture was extracted with EtOAc (3x), then concentrated to afford the crude amine. ^{1}H NMR (400 MHz, $CDCl_3$) δ 7.59 (s, 2H), 4.54 (s, 2H). The off-white solid was redissolved in CH_2Cl_2 (35 mL). Ac_2O (145 µL, 1.54 mmol, 0.95 eq) in CH_2Cl_2 (10 mL) was added dropwise, and the reaction was stirred for 16 hours at room temperature. The reaction was diluted in water, then extracted with CH_2Cl_2. The combined organic layers were concentrated and purified by silica gel flash chromatography (10% Acetone in CH_2Cl_2) to afford the title compound as a white solid (617 mg, 1.48 mmol, 91%). ^{1}H NMR (600 MHz, DMSO) δ 8.22 (t, *J* = 6.0 Hz, 1H), 7.52 (s, 2H), 5.00 (s, 2H), 4.03 (d, *J* = 5.9 Hz, 2H), 1.83 (d, *J* = 3.1 Hz, 3H). ^{13}C NMR (151 MHz, DMSO) δ 169.49, 146.28, 138.76, 132.43, 81.88, 40.59, 23.05. HRMS-EI (m/z) Calculated for $C_9H_{11}N_2OI_2$ $[M+H]^+$, 416.8955; found 416.8953.

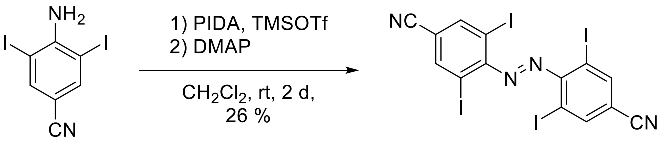

S16 **I$_4$-CN**

I$_4$-CN. (Diacetoxyiodo)benzene (633 mg, 2.0 mmol, 1.5 eq) was dissolved in dry degassed CH_2Cl_2 (10 mL) under nitrogen. Trimethylsilyl trifluoromethanesulfonate (712 µL, 3.9 mmol, 3 eq) was added. DMAP (480 mg, 3.9 mmol, 3 eq) in dry degassed CH_2Cl_2 (5 mL) was added and the yellow solution was stirred for 5 minutes, after which a white precipitate had formed (Weiss's reagent). Nitrile **S16** was added in dry degassed CH_2Cl_2 (5 mL) and the reaction was refluxed under protection from light for 30 minutes, then stirred at room temperature under protection from light for 48 hours. The solution was concentrated and purified by silica gel flash chromatography (40% CH_2Cl_2 in hexane) to afford the title compound as a green solid (124 mg, 168.2 µmol, 26 %). ^{1}H NMR (600 MHz, $CDCl_3$) δ 8.29 (d, *J* = 0.9 Hz, 4H). ^{13}C NMR (151 MHz, $CDCl_3$) δ 153.11, 143.99, 116.03, 115.13, 89.57. HRMS-EI (m/z) Calculated for $C_{12}H_{16}N_2O_6I_2$ $[M-H]^-$, 735.6620; found 735.6617.

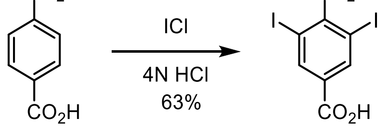

S20 **S21**

S21. Prepared according to a modified literature procedure.[[10]] p-aminobenzoic acid **S20** (3.5 g, 25.5 mmol) was dissolved in 12.5 % HCl (150 mL) at 75 °C. Iodine monochloride (15.6 g, 96 mmol, 4 eq) in 12% HCl (20 mL) was added. The reaction was stirred at 75 °C for one minute after which a light brown precipitate had formed. Water (300 mL) was added and a precipitate formed. The solution was allowed to cool to room temperature, and the solid was obtained by filtration. The filter cake was triturated with EtOH (~300 mL until the yellow washings were colourless) to afford the title compound

as an off-white solid (6.3 g, 16.2 mmol, 63%). ^{1}H NMR (400 MHz, DMSO) δ 12.66 (s, 1H), 8.12 (s, 2H), 5.80 – 5.70 (m, 2H). Data consistent with that given in the literature. [[11]]

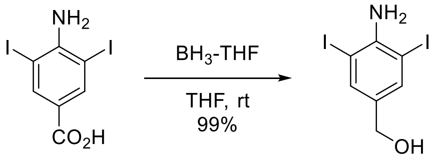

S21 **S22**

Alcohol **S22**. Acid **S21** (6.3 g, 16.2 mmol) was dissolved in THF (50 mL). Then Borane-THF (49 mL, 49 mmol, 3 eq) was added at room temperature. The reaction was stirred at ambient temperature for 16 hours. MeOH was added dropwise until effervescence stopped, then the solution was poured onto EtOAc (200 mL). The organic layer was washed with 1N HCl, then water, then sat. bicarbonate solution. The organic layer was dried and concentrated to afford the title compound as a white solid (6.0 g, 16 mmol, 99%). ^{1}H NMR (600 MHz, $CDCl_3$) δ 7.65 (s, 2H), 4.62 (s, 2H), 4.49 (d, J = 5.7 Hz, 2H), 1.55 (t, J = 5.89 Hz). ^{13}C NMR (151 MHz, $CDCl_3$) δ 145.82, 138.59, 134.02, 81.32, 63.60. HRMS-EI (m/z) Calculated for $C_7H_8NOI_2$ $[M+H]^+$, 375.8690; found 375.8689.

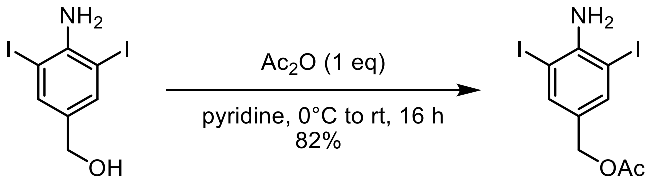

S22 **S23**

Protected Alcohol **S23**. Alcohol **S22** (6.0 g, 16 mmol) was dissolved in pyridine (50 mL). Ac_2O (1.5 mL, 16 mmol, 1 eq) was added dropwise at 0 °C. The reaction was stirred at rt overnight. The solution was concentrated and purified by silica gel flash chromatography (75% CH_2Cl_2 in hexane) to afford the title compound as a white solid (5.5 g, 13.2 mmol, 82%). ^{1}H NMR (600 MHz, $CDCl_3$) δ 7.64 (s, 2H), 4.88 (s, 2H), 4.68 (s, 2H), 2.08 (s, 3H). ^{13}C NMR (151 MHz, $CDCl_3$) δ 170.90, 146.39, 140.02, 128.90, 80.95, 64.45, 21.19. HRMS-EI (m/z) Calculated for $C_{17}H_6I_2$ $[M\text{-}OAc^-]^+$, 357.8584; found 357.8583.

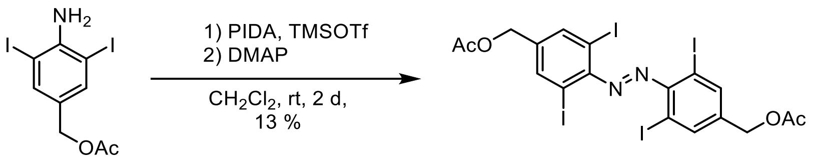

S23 **I$_4$-OAc**

I$_4$-OAc. (Diacetoxyiodo)benzene (4.66 g, 14.5 mmol, 1.1 eq) was dissolved in dry degassed CH_2Cl_2 (200 mL) under Argon. Trimethylsilyl trifluoromethanesulfonate (5 mL, 27.7 mmol, 2.1 eq) was added. DMAP (3.38 g, 27.7 mmol, 2.1 eq) in dry degassed CH_2Cl_2 (100 mL) was added and the yellow solution was stirred for 5 minutes, after which a white precipitate had formed. Aniline **S23** was added in dry degassed CH_2Cl_2 (100 mL) and the reaction was refluxed under protection from light for 30 minutes, then stirred at room temperature under protection from light for 48 hours. The solution was concentrated and purified by silica gel flash chromatography (70% CH_2Cl_2 in hexane) to afford the title compound as a purple solid (691 mg, 832.6 µmol, 13 % [purity ca 90%]). ^{1}H NMR (600 MHz, $CDCl_3$) δ 8.00 (s, 4H), 5.07 (s, 4H), 2.16 (s, 6H). ^{13}C NMR (151 MHz, $CDCl_3$) δ 170.67, 149.88, 140.57, 140.06, 90.15, 63.75, 21.06. HRMS-EI (m/z) Calculated for $C_{18}H_{15}N_2O_4I_4$ $[M+H]^+$, 830.7205; found 830.7206.

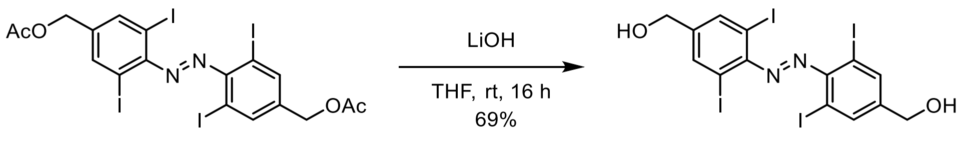

I$_4$-OAc **I$_4$-OH**

I$_4$-OH. I$_4$-OAc was dissolved in THF (20 mL) under N_2. LiOH (160 mg, 8 eq) in water (2 mL) was added and the reaction was stirred under ambient conditions for 16 hours. The reaction was diluted with CH_2Cl_2 and the organic layer separated and concentrated, then purified by silica gel flash chromatography (5% EtOAc in CH_2Cl_2) to afford the title compound as a purple solid (432 mg, 579 µmol, 69%). ^{1}H NMR (600 MHz, $CDCl_3$) δ 8.03 (s, 4H), 4.71 (s, 4H), 1.81 (br s, 2H). ^{13}C NMR (151 MHz, $CDCl_3$) δ 149.34, 144.85, 139.23, 90.33, 63.06. HRMS-EI (m/z) Calculated for $C_{14}H_{11}N_2O_2I_4$ $[M+H]^+$, 746.6994; found 746.6992.

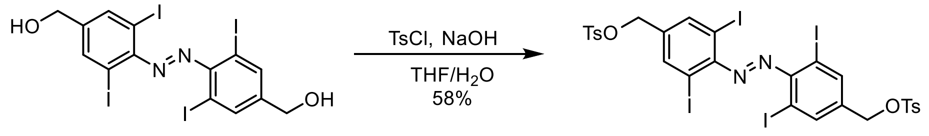

I$_4$-OH **S25**

Tosylate S25. I$_4$-OH (40 mg, 53.6 µmol) was dissolved in 7:3 THF/H_2O (2.5 mL). NaOH (6.5 mg, 161 µmol, 3 eq) was added. Then TsCl (31 mg, 161 µmol, 3 eq) was added. The reaction was stirred at room temperature for 40 minutes under protection from light. The reaction was diluted with CH_2Cl_2, then washed with H_2O (3x). The organic layer was concentrated, then purified by silica gel flash chromatography (dry load, 30% to 70% CH_2Cl_2 in hexane) to afford the title compound as a purple solid (33 mg, 31.3 µmol, 58%). ^{1}H NMR (600 MHz, $CDCl_3$) δ 7.82 (d, *J* = 1.0 Hz, 4H), 7.80 – 7.76 (m, 4H), 7.37 – 7.33 (m, 4H), 5.02 (s, 4H), 2.46 (s, 6H). ^{13}C NMR (151 MHz, $CDCl_3$) δ 150.30, 145.54, 140.64, 137.34, 133.12, 130.19, 128.13, 89.85, 68.97, 22.02. HRMS-EI (m/z) Calculated for $C_{28}H_{23}N_2O_6S_2I_4$ $[M+H]^+$, 1054.7171; found 1054.7173.

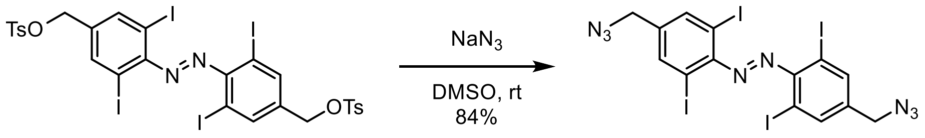

S25 **I$_4$-N$_3$**

I$_4$-N$_3$. 25 (33 mg, 31 µmol) was dissolved in dry DMSO (0.5 mL) under N_2. Sodium azide (10.2 mg, 156 µmol, 5 eq) was added and the reaction was stirred at room temperature for 16 hours. Water was added (5 mL) and the reaction was extracted with Et_2O (3x 10 mL). The combined organic layers were washed with water (3x 10 mL). The organic layer was concentrated, then purified by silica gel flash chromatography (dry load, 30% CH_2Cl_2 in hexane) to afford the title compound as a purple solid (21 mg, 26.4 µmol, 84%). ^{1}H NMR (600 MHz, $CDCl_3$) δ 7.98 (s, 4H), 4.38 (s, 4H). ^{13}C NMR (151 MHz, $CDCl_3$) δ 149.77, 140.26, 139.55, 90.25, 52.50. HRMS-EI (m/z) Calculated for $C_{14}H_9N_8I_4$ $[M+H]^+$, 796.7123; found 796.7122.

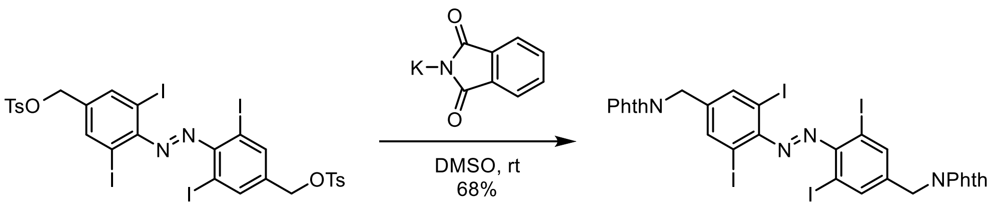

S25 **I_4-NPhth**

I_4-NPhth. 25 (80 mg, 76 µmol) was dissolved in DMSO (1 mL) under N_2. Potassium phthalimide (56 mg, 303.5 µmol, 4 eq) was added, and the reaction was stirred at room temperature for 16 hours. Water was added, and the solid was collected by filtration, washing with water and then Et_2O. The solid was dried to afford the title compound as a pink solid (52 mg, 51.8 µmol, 68%). ^{1}H NMR (600 MHz, $CDCl_3$) δ 8.06 (s, 4H), 7.92 – 7.87 (m, 4H), 7.78 – 7.73 (m, 4H), 4.79 (s, 4H). ^{13}C NMR (151 MHz, $CDCl_3$) δ 167.74, 149.60, 141.04, 139.94, 134.31, 131.94, 123.66, 90.15, 39.59. HRMS-EI (m/z) Calculated for $C_{30}H_{17}N_4O_4I_4$ $[M+H]^+$, 1004.7423; found 1003.2421.

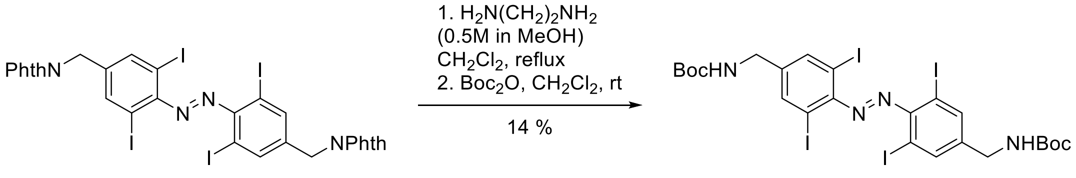

I_4-NPhth **I_4-NHBoc**

I_4-NHBoc. I_4-NPhth (71.7 µmol) was dissolved in CH_2Cl_2 (3 mL). Ethylene diamine (0.5M in MeOH) (1.72 mL, 860 µmol, 12 eq) was added and the reaction was stirred at 40 °C for 3 days. The reaction was concentrated, then redissolved in 1:1 CH_2Cl_2 (5 mL), and Boc_2O (60 mg, 285 µmol, 4 eq) was added. The reaction was stirred for 16 hours at room temperature, then concentrated. The residue was purified by silica gel flash chromatography (dry load, 80% CH_2Cl_2 in hexane) to recover starting material **I_4-NPhth** (22 mg, 22 µmol, 30%). The column was flushed with 2% MeCN in CH_2Cl_2 to afford the title compound as a purple solid (9.7 mg, 10.27 µmol, 14%). ^{1}H NMR (400 MHz, $CDCl_3$) δ 7.93 (s, 4H), 4.94 (s, 2H), 4.30 (d, $J = 6.3$ Hz, 4H), 1.49 (s, 18H). ^{13}C NMR (151 MHz, $CDCl_3$) δ 155.91, 149.25, 143.30, 139.90, 90.44, 80.36, 42.93, 28.54. HRMS-EI (m/z) Calculated for $C_{24}H_{29}N_4O_4I_4$ $[M+H]^+$, 944.8362; found 944.8364.

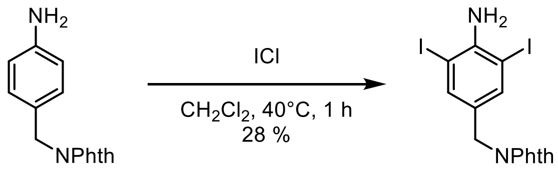

S27 **S28**

Aniline S28. Phthalimide **S27** (880 mg, 3.49 mmol) was dissolved in CH_2Cl_2 (15 mL). Iodine monochloride (1.2 g, 7.33 mmol, 2.1 eq) in CH_2Cl_2 (10 mL) was added. The reaction was refluxed for 1 hour. The mixture was cooled to room temperature and then washed with sodium thiosulfate. The organic layer was dried and concentrated. The crude was purified by silica gel flash chromatography (60 to 80% CH_2Cl_2 in hexane) to afford the title compound as a white solid. (500 mg, 0.99 mmol, 28%). ^{1}H NMR (600 MHz, DMSO) δ 7.89 – 7.86 (m, 2H), 7.86 – 7.82 (m, 2H), 7.61 (s, 2H), 5.09 (s, 2H), 4.56 (s, 2H). ^{13}C NMR (151 MHz, DMSO) δ 167.65, 146.49, 138.69, 134.50, 131.56, 128.69, 123.20, 81.32, 38.87. HRMS-EI (m/z) Calculated for $C_{15}H_{11}N_2O_2I_2$ $[M+H]^+$, 504.8094; found 503.3567.

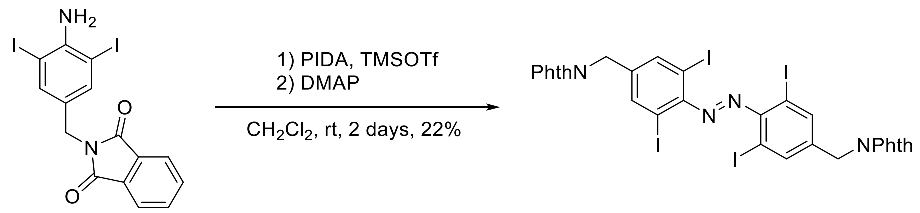

S28 **I_4-NPhth**

I_4-NPhth. (Diacetoxyiodo)benzene (351 mg, 1.09 mmol, 1.1 eq) was dissolved in dry degassed CH_2Cl_2 (25 mL) under nitrogen. Trimethylsilyl trifluoromethnanesulfonate (376 µL, 2.08 mmol, 2.1 eq) was added. DMAP (254 mg, 2.08 mmol, 2.1 eq) in dry degassed CH_2Cl_2 (10 mL) was added and the yellow solution was stirred for 5 minutes, after which a white precipitate had formed. Aniline **S28** was added in dry degassed CH_2Cl_2 (25 mL) and the reaction was refluxed under protection from light for 30 minutes, then stirred at room temperature under protection from light for 48 hours. The solution was concentrated and purified by silica gel flash chromatography (40% to 80% CH_2Cl_2 in hexane) to afford the title compound as a purple solid (108 mg, 107 µmol, 22 %). ^{1}H NMR (600 MHz, $CDCl_3$) δ 8.06 (s, 4H), 7.92 – 7.87 (m, 4H), 7.78 – 7.73 (m, 4H), 4.79 (s, 4H). ^{13}C NMR (151 MHz, $CDCl_3$) δ 167.74, 149.60, 141.04, 139.94, 134.31, 131.94, 123.66, 90.15, 39.59. HRMS-EI (m/z) Calculated for $C_{30}H_{17}N_4O_4I_4$ $[M+H]^+$, 1004.7423; found 1003.2421.

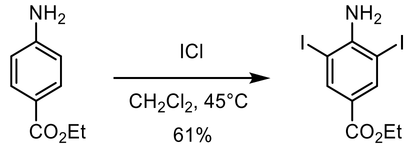

S29 **S30**

Aniline S30. Benzocaine **S29** (2.0 g, 12.11 mmol) was dissolved in CH_2Cl_2 (30 mL). Iodine monochloride (4.91 g, 30.3 mmol, 2.5 eq) in CH_2Cl_2 (20 mL) was added. The reaction was refluxed for 1 hour. The mixture was cooled to room temperature and then washed with sodium thiosulfate. The organic layer was dried and concentrated. The residue was triturated with Et_2O until the purple washings were colourless. The solid was dried to afford the title compound as a white solid (3.1 g, 7.43 mmol, 61 %). ^{1}H NMR (400 MHz, $CDCl_3$) δ 8.30 (s, 2H), 5.05 (s, 2H), 4.32 (q, J = 7.1 Hz, 2H), 1.37 (t, J = 7.1 Hz, 3H). Data consistent with that given in the literature. [12]

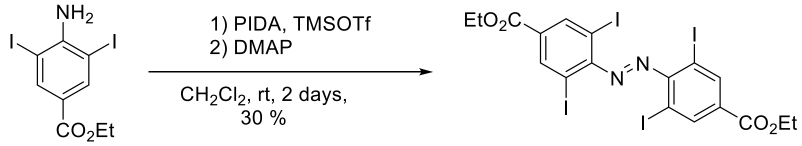

S30 **I_4-ester**

I_4-Ester. PIDA (3g, 7.2 mmol, 1.0 eq) was dissolved in dry degassed CH_2Cl_2 (100 mL) under nitrogen. Trimethylsilyl trifluoromethanesulfonate (2.73 mL, 15.1 mmol, 2.1 eq) was added. DMAP (1.85 g, 15.1 mmol, 2.1 eq) in dry degassed CH_2Cl_2 (50 mL) was added and the yellow solution was stirred for 5 minutes, after which a white precipitate had formed. Ester **S30** was added in dry degassed CH_2Cl_2 (50 mL) and the reaction was refluxed under protection from light for 30 minutes, then stirred at room temperature under protection from light for 72 hours. The solution was concentrated and purified by silica gel flash chromatography (40% to 50% CH_2Cl_2 in hexane) to afford the title compound as a purple solid (900 mg, 1.08 mmol, 30 %). ^{1}H NMR (600 MHz, $CDCl_3$) δ 8.65 (s, 4H), 4.42 (q, J = 7.1 Hz, 4H), 1.43 (t, J = 7.1 Hz, 6H). ^{13}C NMR (151 MHz, $CDCl_3$) δ 163.33, 152.98, 142.06, 133.14, 89.16, 62.16, 14.45. HRMS-EI (m/z) Calculated for $C_{18}H_{15}N_2O_4I_4$ $[M+H]^+$, 830.7205; found 830.7203.

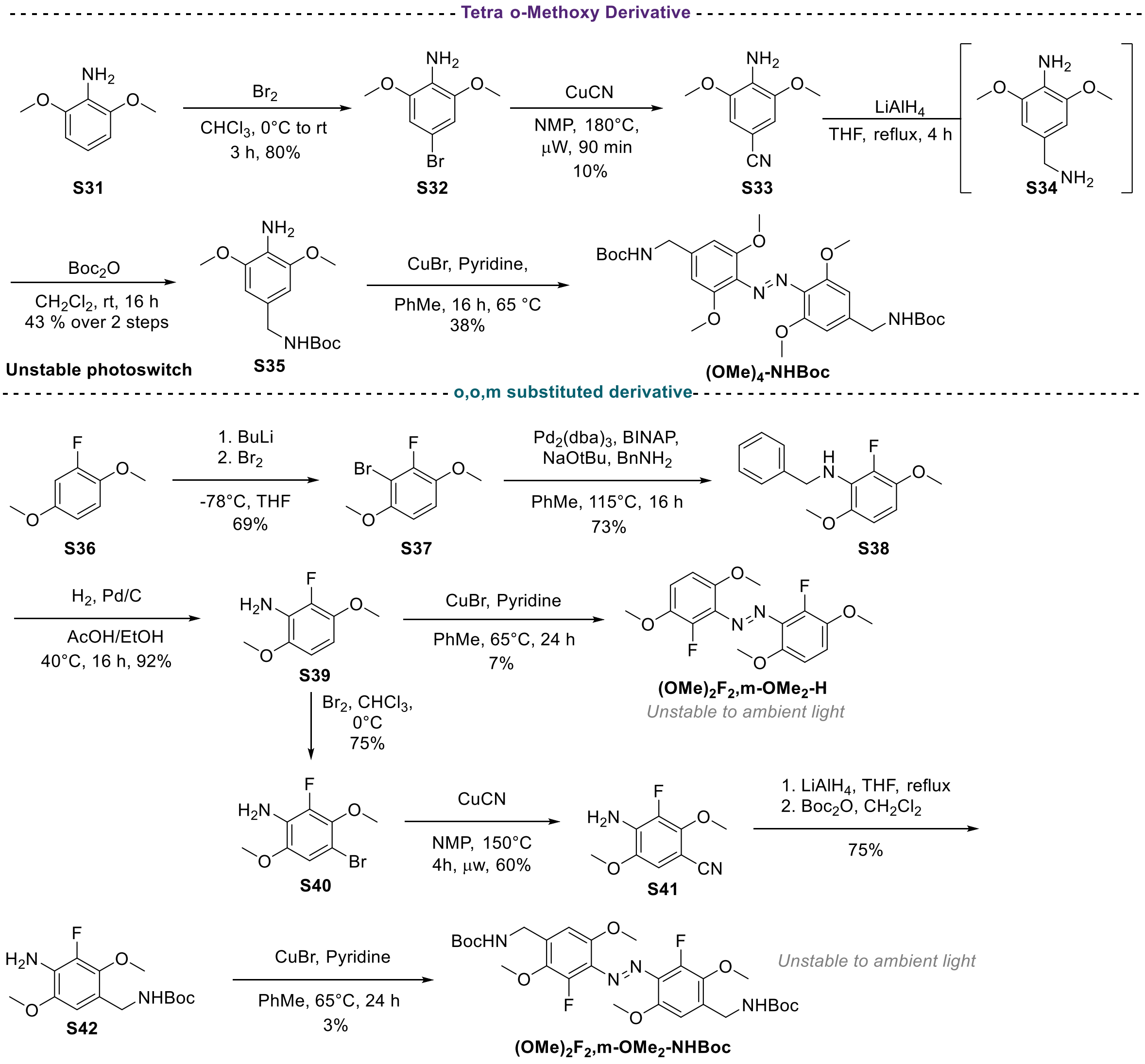

Scheme S4. Synthesis of tetra-methoxy azobenzene derivatives.

S32 was prepared via regioselective bromination of **S31**. Cyanation to **S33** proceeded in poor yields due to heavy emulsion formation. The nitrile was reduced and protected to **S35**, which was dimerised using CuBr/Pyridine in air to afford the $(OMe)_4$ photoswitch. **$(OMe)_2F_2$,*m*-OMe_2-NHBoc** was accessed via an *o*-lithiation strategy followed by cross-coupling using benzylamine as the ammonia equivalent to afford **S39**. The functional handle was installed as usual: bromination/Cyanation/reduction/protection/dimerization to afford the photoswitch, which was unstable to both ambient light and LEDs.

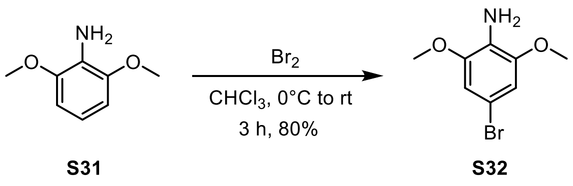

S32. Prepared according to a literature procedure. [[13]] 2,6-dimethoxyaniline **S31** (722 mg, 4.71 mmol, 1.03 eq) was dissolved in $CHCl_3$ (12 mL). Bromine (731 mg, 4.58 mmol, 1 eq) in $CHCl_3$ (12 mL) was added dropwise at 0 °C via a dropping funnel, then allowed to warm to room temperature over 3 hours. The reaction was quenched with aqueous sodium thiosulfate. The organic layer was separated and concentrated. The residue was purified by silica gel flash chromatography (90% CH_2Cl_2 in hexane) to afford the title compound as a white solid (849 mg, 3.67 mmol, 80%). ^{1}H NMR (400 MHz, $CDCl_3$) δ 6.66 (s, 2H), 4.34 – 3.06 (br s, 2H), 3.84 (s, 6H). Data consistent with that given in the literature. [[13]]

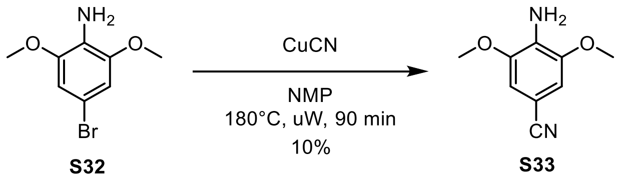

Nitrile S33. In a microwave vial was added **S33** (740 mg, 3.19 mmol) and CuCN (571 mg, 6.38 mmol, 2 eq). The vial was purged with N_2 for 10 minutes. Then, 6.5 mL NMP was added, the tube was sealed and reaction was heated to 180 °C under microwave irradiation for 90 min. The reaction was cooled, poured over ice water with 15% ammonia solution and extracted with 50:50 hexane : EtOAc 3x. *Warning: terrible emulsion formed: recommend filtering the contents of separating funnel before each extraction.* The combined organic layers were sequentially washed with water and 5% LiCl solution, then concentrated. The resulting residue purified by silica gel flash chromatography (20% Acetone in hexane) to afford the title compound as a white solid (55 mg, 0.31 mmol, 10%). ^{1}H NMR (600 MHz, $CDCl_3$) δ 6.79 (d, *J* = 1.4 Hz, 2H), 3.87 (s, 6H). ^{13}C NMR (151 MHz, $CDCl_3$) δ 146.28, 131.08, 120.68, 98.14, 56.20. HRMS-EI (m/z) Calculated for $C_9H_{11}O_2N_2$ $[M+H]^+$, 179.0815; found 179.0815.

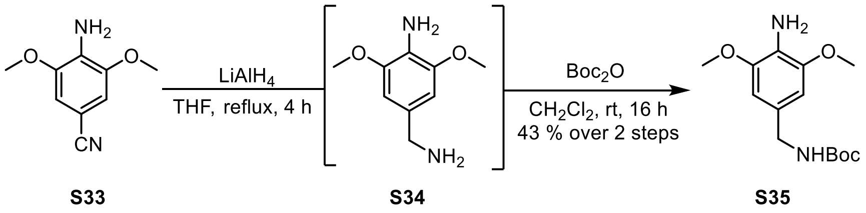

Aniline S35. To a suspension of $LiAlH_4$ (75 mg, 2.04 mmol, 7 eq) in dry THF (3 mL) was added **S33** (52 mg, 292 µmol, 1.0 eq) in THF (2 mL). The suspension was refluxed for 16 hours. The reaction mixture was cooled, diluted in Et_2O (5 mL), then water (100 µL), 2.5M NaOH (100 µL), and water (0.3 mL) was added dropwise (*Fieser Workup*). The suspension was stirred for 15 minutes, and then anhydrous $MgSO_4$ was added. The solution was filtered, filtrate concentrated to afford **S34** without further purification. ^{1}H NMR (400 MHz, $CDCl_3$) δ 6.49 (s, 2H), 3.85 (s, 6H), 3.78 (s, 2H), 3.74 (br s, 2H). The amine was dissolved in CH_2Cl_2 (10 mL), then Boc_2O (60.5 mg, 6.93 mmol, 0.9 eq) in CH_2Cl_2 (15 mL) was added dropwise. The reaction was stirred at room temperature for 16 h. The reaction was concentrated, then purified by silica gel flash chromatography (2% to 4% Acetone in CH_2Cl_2) to afford the title compound as a white solid (45.5 mg, 159 µmol, 55%). ^{1}H NMR (600 MHz, $CDCl_3$) δ 6.47 (s, 2H), 4.74 (s, 1H), 4.21 (d, *J* = 5.5 Hz, 2H), 3.84 (s, 6H), 1.47 (s, 9H). ^{13}C NMR (151 MHz, $CDCl_3$) δ 156.03, 147.56, 127.95, 124.67, 103.91, 79.49, 56.01, 45.38, 28.58. HRMS-EI (m/z) Calculated for $C_{14}H_{23}O_4N_2$ $[M+H]^+$, 283.1652; found 283.1648.

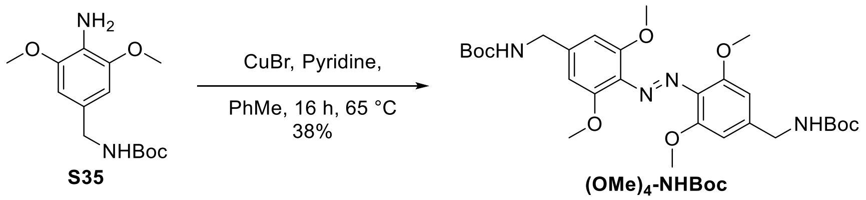

(OMe)$_4$-NHBoc. Aniline **S35** (43 mg, 152 mmol) was dissolved in toluene (2.5 mL). Pyridine (25 µL, 304 mmol, 2 eq) and CuBr (22 mg, 152 µmol, 1 eq) was added and the reaction was stirred under atmospheric air at 65 °C for 24 hours under protection from light. The reaction was concentrated, then purified by silica gel flash chromatography (6% - 14% Acetone in CH_2Cl_2), and the pure fractions were combined to afford the title compound as an orange solid (16 mg, 29 µmol, 38%). ^{1}H NMR (600 MHz, $CDCl_3$) δ 6.58 (s, 4H), 4.90 (s, 2H), 4.30 (d, *J* = 6.0 Hz, 4H), 3.83 (s, 13H), 1.47 (s, 18H). ^{13}C NMR (151 MHz, $CDCl_3$) δ 155.99, 152.60, 140.97, 133.38, 104.30, 79.69, 56.61, 45.12, 28.41. HRMS-EI (m/z) Calculated for $C_{14}H_{23}O_4N_2$ $[M+H]^+$, 561.2919; found 561.2918.

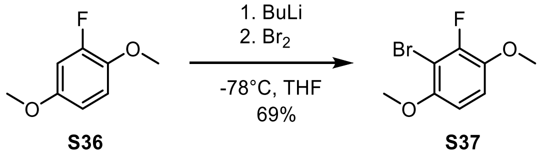

S37. A flame-dried 250 mL round-bottom flask equipped with a magnetic stir-bar and a septum was charged with **S36** (4.2 g, 25 mmol) and THF (100 mL). The reaction mixture was cooled to –78 °C in a dry ice/acetone bath and n-butyllithium (2.5 M in hexane, 11.3 mL, 26.5 mmol, 1.06 eq) was added dropwise via syringe. The reaction mixture was allowed to stir at –78 °C for 1 h, after which time bromine (1.46 mL, 25.5 mmol, 1.02 eq) was added dropwise via syringe and the reaction mixture was allowed to slowly warm up to room temperature over the course of 6 h. The reaction was then quenched with 100 mL of a saturated solution of Na_2SO_3 in water and the resulting mixture was transferred to a separatory funnel. The aqueous and organic phases were separated and the aqueous phase was extracted with EtOAc (2 x 60 mL). The combined organic layers were dried over Na_2SO_4, filtered and concentrated. The crude residue was dissolved in CH_2Cl_2 and filtered through a short plug of silica gel, eluting with 20% CH_2Cl_2 in hexanes. The filtrate was concentrated and the residue was purified via column chromatography on silica gel (5% EtOAc in hexanes) to afford the title compound as white crystals (4.32 g, 17.12 mmol, 68%). ^{1}H NMR (500 MHz, CD_2Cl_2) δ 6.92 (t, *J* = 9.2 Hz, 1H), 6.66 (dd, *J* = 9.1, 2.2 Hz, 1H), 3.84 (s, 3H), 3.84 (s, 3H). Data consistent with that given in the literature.[[14]]

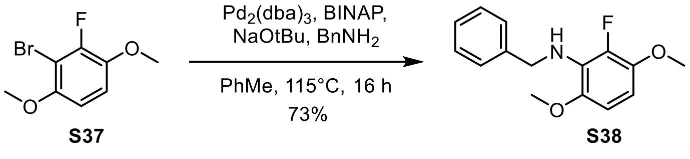

Protected amine S38. Aryl bromide **S37** (1.5 g, 6.38 mmol), Pd_2dba_3 (292 mg, 0.32 mmol, 0.05 eq), rac-BINAP (238 mg, 0.383 mmol, 0.06 eq), benzylamine (2.74 g, 25.53 mmol, 4 eq) and sodium *tert*-butoxide (859 mg, 8.93 mmol, 1.4 eq) were added to a 20 mL microwave vial. The solids were purged with N_2 for 30 minutes. Degassed dry toluene (20 mL) was added, the tube was sealed and the reaction was refluxed for 16 hours. The solution was cooled and diluted with CH_2Cl_2 (100 mL). The organic layer was washed with 1M HCl (100 mL). The organic layer was collected and concentrated in *vacuo*. The residue was purified by silica gel flash chromatography (85% CH_2Cl_2 in hexane), to afford the title compound as a yellow oil (1.21 g, 6.38 mmol, 73%). ^{1}H NMR (500 MHz, $CDCl_3$) δ 7.41 – 7.20 (m,

5H), 6.48 (dd, J = 8.9, 2.0 Hz, 1H), 6.33 (t, J = 8.9 Hz, 1H), 4.51 (d, J = 1.8 Hz, 2H), 4.36 (br s, 1H), 3.82 (s, 3H), 3.78 (s, 3H). ^{13}C NMR (126 MHz, $CDCl_3$) δ 144.38 (d, J = 7.0 Hz), 153.57 (d, J = 238.4 Hz) 143.51 (d, J = 10.4 Hz), 128.59, 127.85, 127.70 (d, J = 9.5 Hz), 127.22, 104.97 (d, J = 3.2 Hz), 101.80 (d, J = 1.7 Hz), 56.77, 56.47, 50.74 (d, J = 7.8 Hz). HRMS-EI (m/z) Calculated for $C_{15}H_{17}O_2NF$ $[M+H]^+$, 262.1238; found 262.1239

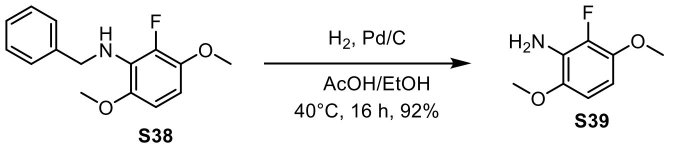

Aniline 39. Benzylamine **S38** (600 mg, 2.30 mmol) was dissolved in 1:1 AcOH:EtOH (20 mL). The reaction vessel was evacuated and filled with nitrogen 3 x. Palladium 10% on carbon (570 mg) was added and the reaction was stirred under a hydrogen atmosphere for 16 hours at 40 °C. The reaction was filtered over celite, the filtrate concentrated to afford the title compound as a grey oil (360 mg, 2.1 mmol, 92%). ^{1}H NMR (400 MHz, $CDCl_3$) δ 6.54 – 6.40 (m, 1H), 6.28 (t, J = 9.0 Hz, 1H), 3.82 (m, 8H). ^{13}C NMR (101 MHz, $CDCl_3$) δ 142.85 (d, J = 6.2 Hz) 142.77 (d, J = 9.8 Hz), 141.93 (d, J = 236.4 Hz), 125.94 (d, J = 12.5 Hz), 104.89 (d, J = 3.2 Hz), 100.71, 56.77, 56.27. HRMS-EI (m/z) Calculated for $C_8H_{11}O_2NF$ $[M+H]^+$, 172.0768; found 172.0770

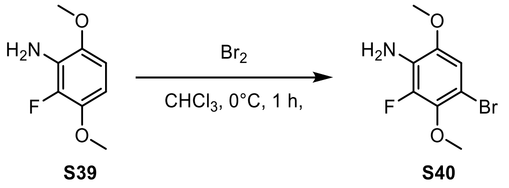

Aryl bromide S40. Aniline **S39** (520 mg, 3.04 mmol) was dissolved in $CHCl_3$ (60 mL) and the solution was cooled to 0°C. Bromine (157 µL) in 5 mL $CHCl_3$ was added dropwise and the reaction was stirred at this temperature for 1 hour. Saturated sodium thiosulfate was added and the organic layer was separated and concentrated in vacuo. The residue was purified by silica gel flash chromatography (85% CH_2Cl_2 in hexane) to afford the title compound as a grey oil which solidified over time (620 mg, 2.48 mmol, 82%). ^{1}H NMR (400 MHz, $CDCl_3$) δ 6.71 (d, J = 2.2 Hz, 1H), 3.86 (d, J = 0.8 Hz, 3H), 3.82 (s, 3H), 3.79 (s, 2H). ^{13}C NMR (126 MHz, $CDCl_3$) δ 146.36, 145.08 – 143.83 (m), 140.12 (d, J = 12.0 Hz), 125.62 (d, J = 13.7 Hz), 109.39 (d, J = 2.6 Hz), 102.75 (d, J = 3.9 Hz), 61.75 (d, J = 3.7 Hz), 56.34. HRMS-EI (m/z) Calculated for $C_8H_{10}O_2NBrF$ $[M+H]^+$, 249.9873; found 249.9875.

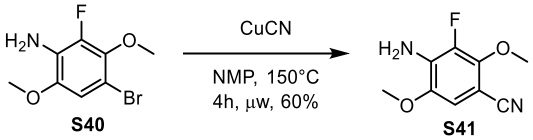

Nitrile S41. Bromide **S40** (589 mg, 2.36 mmol) was dissolved in NMP (6 mL) under N_2 in a microwave tube. The tube was sealed and reaction was heated to 160 °C under microwave irradiation for 3h. The reaction mixture was cooled, poured onto 15% ammonia solution, then extracted with 50:50 hexane/EtOAc 3x. The combined organic layers were washed with water 5x, then concentrated. The residue was purified by silica gel flash chromatography (75% CH_2Cl_2 in hexane) to afford the title compound as a grey oil which solidified over time into a white solid (620 mg, 2.48 mmol, 82%). ^{1}H NMR (400 MHz, $CDCl_3$) δ 6.66 (d, J = 1.9 Hz, 1H), 4.30 (s, 2H), 4.02 (d, J = 1.7 Hz, 3H), 3.85 (s, 3H).

^{13}C NMR (126 MHz, $CDCl_3$) δ 145.82 (d, *J* = 10.9 Hz), 142.92 (d, *J* = 7.2 Hz), 142.82, (d, *J* = 7.2 Hz), 131.65 (d, *J* = 238.2 Hz), 116.99 (d, *J* = 4.8 Hz), 108.35 (d, *J* = 2.3 Hz), 91.73 (d, *J* = 4.9 Hz), 62.16 (d, *J* = 5.1 Hz), 56.26. HRMS-EI (m/z) Calculated for $C_9H_{10}O_2N_2F$ [M+H]$^+$, 197.0721; found 197.0722.

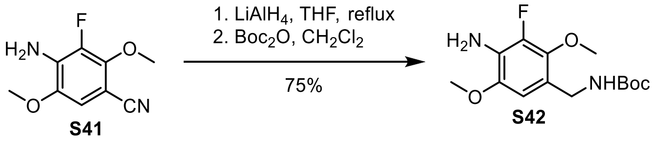

Aniline **S42**. $LiAlH_4$ (78 mg, 2.1 mmol, 3 eq) was suspended in dry THF (4 mL). Then, nitrile **S41** (135 mg, 0.69 mmol) in dry THF (4 mL) was added dropwise. The reaction was refluxed for 16 hours, then allowed to cool to room temperature. The reaction was diluted with Et_2O (10 mL), then quenched with 80 µL water, then 80 µL 10% NaOH, followed by 240 µL water (*Fieser workup).* This was stirred for 15 minutes, after which $MgSO_4$ was added, and the suspension was stirred a further 15 minutes. The solids were filtered off, and the filtrate concentrated to afford the intermediate amine. ^{1}H NMR (400 MHz, $CDCl_3$) δ 6.47 (d, *J* = 2.0 Hz, 1H), 3.84 (d, *J* = 1.3 Hz, 3H), 3.80 (s, 3H), 3.73 (s, 2H). This was immediately dissolved in CH_2Cl_2 (10 mL), to which Boc_2O (135 mg, 0.9 eq) in CH_2Cl_2 (5 mL) was added dropwise at room temperature. The reaction was stirred at this temperature for 16 hours, then concentrated. The residue was purified by silica gel flash chromatography (4% acetone in CH_2Cl_2) to afford the title compound as a white solid (156 mg, 0.52 mmol, 75%). ^{1}H NMR (400 MHz, $CDCl_3$) δ 6.50 (s, 1H), 4.91 (br s, 1H), 4.21 (d, *J* = 6.0 Hz, 2H), 3.84 (d, *J* = 1.3 Hz, 3H), 3.80 (s, 3H), 3.76 (br s, 2H), 1.43 (s, 9H). ^{13}C NMR (101 MHz, $CDCl_3$) δ 155.99, 144.93 (d, *J* = 238.44 Hz), 143.85 (d, *J* = 6.9 Hz), 140.34 (d, *J* = 9.7 Hz), 125.26 (d, *J* = 13.2 Hz), 119.74 (d, *J* = 2.1 Hz), 106.09, 79.38, 61.75 (d, *J* = 5.2 Hz), 56.17, 39.82, 28.53. HRMS-ESI (m/z) Calculated for $C_{14}H_{21}N_2O_4FNa$ [M+Na]$^+$, 323.1378; found 323.1382.

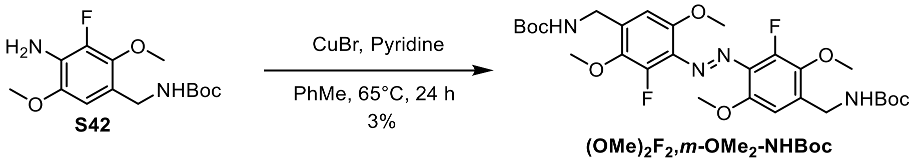

$(OMe)_2F_2$,*m*-OMe_2-NHBoc. Aniline S42 (154 mg, 520 µmol) was dissolved in toluene (10 mL). Pyridine (125 µL, 1.56 mmol, 3 eq) and CuBr (74 mg, 520 µmol, 1 eq) was added and the reaction was stirred under atmospheric air at 65 °C for 24 hours protected from light. The reaction was concentrated, then purified by silica gel flash chromatography (5% EtOAc, 1 % MeOH in CH_2Cl_2). The fractions containing product were concentrated and further purified by silica gel flash chromatography (50% EtOAc in in hexane) to afford the title compound as an orange solid (5 mg, 8 µmol, 3%). ^{1}H NMR (400 MHz, Acetone) δ 6.99 (d, *J* = 1.8 Hz, 1H), 6.52 (t, *J* = 6.2 Hz, 1H), 4.38 (d, *J* = 6.2 Hz, 2H), 3.90 (s, 3H), 3.85 (s, 3H), 1.44 (s, 9H). ^{13}C NMR (126 MHz, Acetone) δ 156.86, 150.68, 147.27 (d, *J* = 260 Hz), 140.44, 136.66, 133.58, 107.98, 79.11, 62.04, 57.16, 39.52, 28.63. HRMS-ESI (m/z) Calculated for $C_{28}H_{39}N_4O_8F_2Na$ [M+H]$^+$, 597.2730; found 597.2728.

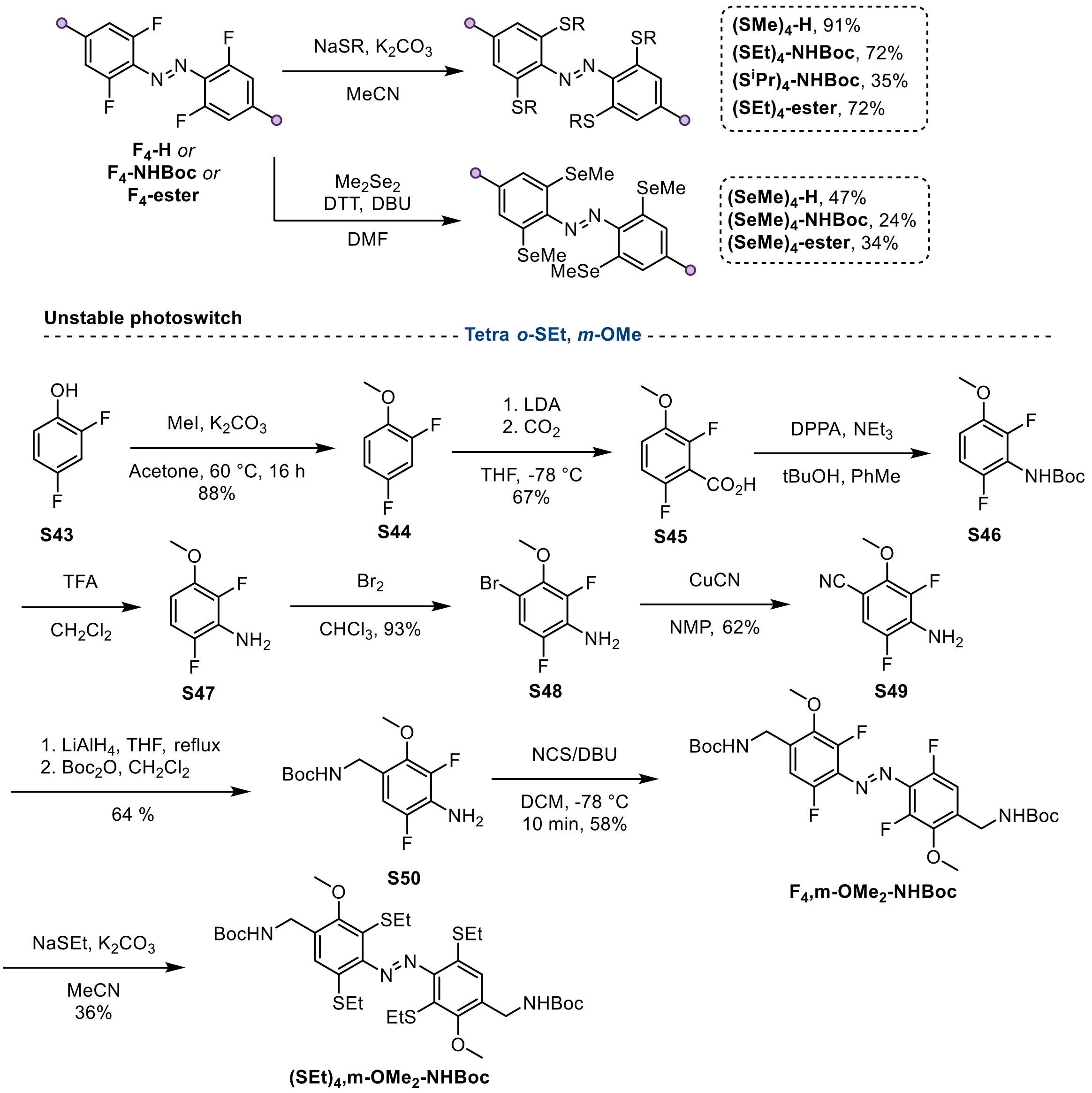

Scheme S4. Synthesis of tetra-*ortho*-thio and seleno azobenzene derivatives.

Thio and seleno derivatives were prepared via an S_NAr reaction with either commercially available thiolates or in-situ generated selenolate nucleophiles[15]. **F$_4$,*m*-OMe$_2$-NHBoc** was prepared *via* an ortho lithiaton/curtius rearrangement strategy, followed by bromoination/Cyanation/reduction/protection/azo coupling.

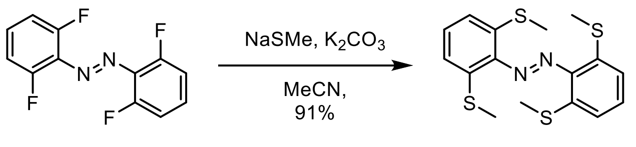

F_4-H **(SMe)$_4$-H**

(SMe$_4$)-H. **F_4-H** (100 mg, 393 µmol), Sodium methanethiolate (200 mg, 3.15 mmol, 8 eq) and potassium carbonate (200 mg, 1.57 mmol, 4 eq) were suspended in MeCN (2 mL) and stirred at room temperature for 16 hours under N_2 in a sealed tube. The mixture was diluted in CH_2Cl_2, then washed with water. The organic layer was concentrated, then purified by silica gel flash chromatography (0 to 60% hexane in CH_2Cl_2) to afford the title compound as an orange solid (131 mg, 357 µmol, 91%). ^{1}H NMR (400 MHz, $CDCl_3$) δ 7.27 (t, *J* = 7.9 Hz, 2H), 7.09 (d, *J* = 8.0 Hz, 4H), 2.43 (s, 12H). ^{13}C NMR (101 MHz, $CDCl_3$) δ 145.69, 138.18, 129.67, 120.74, 16.46. HRMS-EI (m/z) Calculated for $C_{16}H_{19}N_2S_4$ $[M+H]^+$, 367.0426; found 367.0424

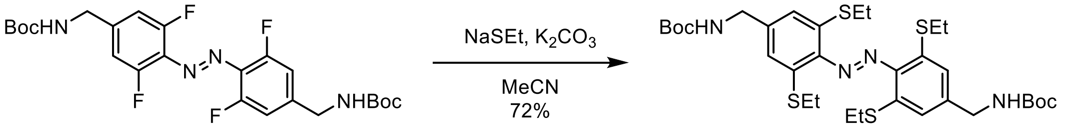

F_4-NHBoc **(SEt)$_4$-NHBoc**

(SEt)$_4$-NHBoc. **F_4-NHBoc** (100 mg, 209 µmol), Sodium ethanethiolate (80%, 164 mg, 1.68 mmol, 8 eq) and potassium carbonate (107 mg, 836 µmol, 4 eq) were suspended in MeCN (2 mL) and stirred at room temperature for 16 hours under N_2 in a sealed tube. The mixture was diluted in CH_2Cl_2, then washed with water. The organic layer was concentrated, then purified by silica gel flash chromatography (0 to 2% Acetone in CH_2Cl_2) to afford the title compound as an orange solid (96 mg, 141 µmol, 72%). ^{1}H NMR (400 MHz, $CDCl_3$) δ 7.03 (s, 4H), 4.88 (br s, 2H), 4.30 (d, *J* = 6.2 Hz, 4H), 2.91 (q, *J* = 7.4 Hz, 8H), 1.47 (s, 18H), 1.32 (t, *J* = 7.3 Hz, 12H). ^{13}C NMR (126 MHz, $CDCl_3$) δ 155.92, 145.85, 140.17, 137.10, 121.02, 79.74, 28.41, 26.76, 13.40. HRMS-EI (m/z) Calculated for $C_{32}H_{49}O_4N_4S_4$ $[M+H]^+$, 681.2631; found 681.2623

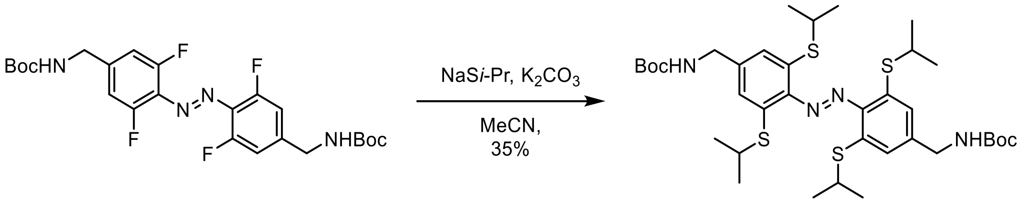

F_4-NHBoc **(SiPr)$_4$-NHBoc**

(S^{i}Pr)$_4$-NHBoc. **F_4-NHBoc** (100 mg, 195 µmol), Sodium 2-propanethiolate (90%, 164 mg, 1.56 mmol, 8 eq) and potassium carbonate (100 mg, 780 µmol, 4 eq) were suspended in MeCN (3.5 mL) and stirred at room temperature for 16 hours under N_2 in a sealed tube. The mixture was diluted in CH_2Cl_2, then washed with water. The organic layer was concentrated, then purified by silica gel flash chromatography (0 to 1% Acetone in CH_2Cl_2) to afford the title compound as an orange solid (950 mg, 68 µmol, 35%). ^{1}H NMR (500 MHz, $CDCl_3$) δ 7.17 (s, 4H), 4.88 (s, 2H), 4.31 (d, *J* = 6.1 Hz, 4H), 3.48 (hept, *J* = 6.7 Hz, 4H), 1.48 (s, 18H), 1.27 (d, *J* = 6.6 Hz, 24H). ^{13}C NMR (126 MHz, $CDCl_3$) δ 155.88, 148.52, 139.51, 135.28, 124.44, 79.71, 44.50, 28.40, 22.77. HRMS-EI (m/z) Calculated for $C_{36}H_{57}O_4N_4S_4$ $[M+H]^+$, 737.3251; found 737.3257

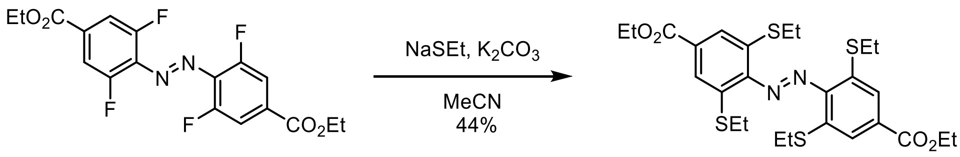

F_4-ester **$(SEt)_4$-ester**

$(SEt)_4$-ester. **F_4-ester** (66 mg, 165 µmol), Sodium ethanethiolate (80%, 142 mg, 1.23 mmol, 7.5 eq) and potassium carbonate (150 mg, 0.99 mmol, 6 eq) were suspended in MeCN (3 mL) and stirred at room temperature for 16 hours under N_2 in a sealed tube. The mixture was diluted in CH_2Cl_2, then washed with water. The organic layer was concentrated, then purified by silica gel flash chromatography (50 to 70% CH_2Cl_2 in hexane) to afford title compound as a brown solid (41.7 mg, 73.5 µmol, 44%).^{1}H NMR (600 MHz, $CDCl_3$) δ 7.81 (s, 4H), 4.42 (q, *J* = 7.1 Hz, 4H), 3.00 (q, *J* = 7.4 Hz, 8H), 1.42 (t, *J* = 7.1 Hz, 6H), 1.35 (t, *J* = 7.4 Hz, 12H).^{13}C NMR (151 MHz, $CDCl_3$) δ 165.92, 148.84, 137.41, 130.66, 123.30, 61.63, 27.11, 14.47, 13.42. HRMS-EI (m/z) Calculated for $C_{26}H_{35}O_4N_2S_4$ $[M+H]^+$, 567.1474; found 567.1473

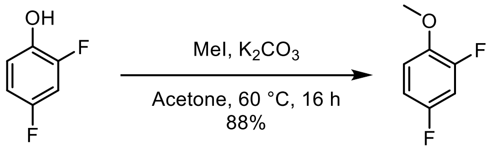

S43 **S44**

Anisole **S44**. Phenol **S43** (5.15 mL, 53.81 mmol) was dissolved in Acetone (50 mL). Potassium carbonate (29.8 g, 215.23 mmol, 4 eq) and 1methyl iodide (7.37 mL, 118.4 mmol, 2.2 eq) was added and the reaction was stirred at 60 °C for 16 hours. The mixture was filtered, then solvent removed in vacuo. The residue was dissolved in CH_2Cl_2, then washed with water. The organic layer was dried and concentrated to afford the title compound as a clear oil (6.85 g, 47.5 mmol, 88%). ^{1}H NMR (400 MHz, $CDCl_3$) δ 6.94 – 6.72 (m, 3H), 3.85 (s, 3H). ^{13}C NMR (101 MHz, $CDCl_3$) δ 156.43 (dd, *J* = 240.5, 10.4 Hz), 152.32 (dd, *J* = 248.3, 10.4 Hz), 144.39 (dd, *J* = 10.7, 3.4 Hz), 114.04 (dd, *J* = 9.5, 2.9 Hz), 110.34 (dd, *J* = 22.4, 4.1 Hz), 104.89 (dd, *J* = 27.0, 21.9 Hz). Data consistent with that given in the literature. [16]

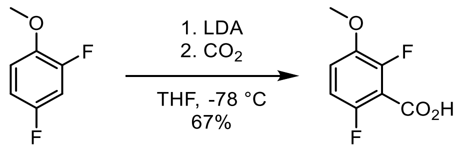

S44 **S45**

Acid S45. Difluoride **S44** (6.85 g, 47.5 mmol) was dissolved in dry THF (50 mL). In a separate flask, BuLi (22.8 mL, 57.04 mmol, 1.2 eq) was added to DIPA (8.72 mL, 61.8 mmol, 1.3 eq) in dry THF (30 mL) at -78 °C. The solution was stirred at 0 °C for 15 minutes, then added dropwise to the solution of **S44** at -78 °C. The solution was stirred at this temperature for 90 minutes. Then CO_2 (Dry ice, dried over $CaSO_4$) was bubbled through the mixture for 15 minutes, after which a solid precipitate had formed. The reaction was warmed to 0 °C, and 8M HCl was added slowly until pH 1 was reached. The solution was extracted with ethyl acetate (3 × 100 mL). The organic layers were extracted with 5% aq. NaOH (3 × 100 mL). The NaOH extracts were adjusted to pH 1 with HCl and the white solid was filtered, washed with water then dried in vacuo to afford the title compound as a white solid (6 g, 31.9 mmol, 67%).^{1}H NMR (400 MHz, $CDCl_3$) δ 10.18 (s, 1H), 7.09 (td, *J* = 9.1, 4.9 Hz, 1H), 6.91 (td, *J* = 9.2, 2.1 Hz, 1H), 3.90 (s, 3H). ^{13}C NMR (101 MHz, $CDCl_3$) δ 166.51, 154.45 (dd, *J* = 251.7, 4.8 Hz),

151.02 (dd, J = 259.4, 5.9 Hz), 144.79 (dd, J = 10.9, 3.5 Hz), 118.72 – 116.02 (m), 111.23 (dd, J = 23.3, 4.5 Hz), 110.40, 57.27. HRMS-ESI (m/z) Calculated for $C_8H_5O_3F_2$ $[M-H]^-$, 187.0212; found 187.0202.

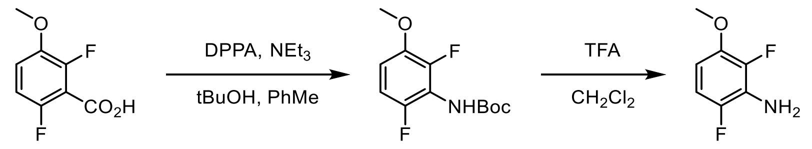

S45 **S46** **S47**

Aniline S47. To a mixture of **S45** (4 g, 21.3 mmol), triethyl amine (3.26 mL, 23.4 mmol, 1.1 eq) and tert-butanol (2.22 mL, 1.73 g, 23.4 mmol, 1.1) in toluene (68 mL) was added diphenylphosphorylazide (4.25 mL, 22.32 mmol, 1.05 eq). The reaction was heated at 65 °C for 2 hours. The reaction mixture was evaporated and the residue was dissolved in ethyl acetate (70 mL), washed with sat KH_2PO_4, $NaHCO_3$ and brine, then dried over Na_2SO_4, filtered and evaporated under vacuum to give **S46** as white solid. The residue was dissolved in CH_2Cl_2 (70 mL), then trifluoroacetic acid (15 mL) was added, and the reaction was stirred at room temperature for 2 hours. The reaction was dried under a stream of nitrogen, then concentrated in vacuo. The pH of the residue was adjusted to pH 8 with a saturated aqueous solution of sodium bicarbonate. The organic layer was separated and the aqueous layer is extracted with CH_2Cl_2 3x. The organic extracts were combined, then purified by silica gel flash chromatography (50% CH_2Cl_2 in hexane) to afford the title compound as a clear oil (1.54 g, 9.68 mmol, 46% [2 steps]. ^{1}H NMR (400 MHz, $CDCl_3$) δ 6.71 (ddd, J = 10.2, 9.2, 2.3 Hz, 1H), 6.26 (td, J = 9.1, 4.8 Hz, 1H), 3.83 (s, 3H), 3.81 – 3.64 (m, 2H). ^{13}C NMR (101 MHz, $CDCl_3$) δ 146.71 (dd, J = 233.4, 5.5 Hz), 144.52 (dd, J = 8.9, 2.6 Hz), 141.88 (dd, J = 238.7, 6.8 Hz), 124.88 (dd, J = 17.6, 13.7 Hz), 109.09 (dd, J = 19.7, 3.7 Hz), 100.58 (d, J = 8.7 Hz), 56.61. HRMS-APCI (m/z) Calculated for $C_7H_8ONF_2$ $[M+H]^+$, 160.0568; found 160.0565.

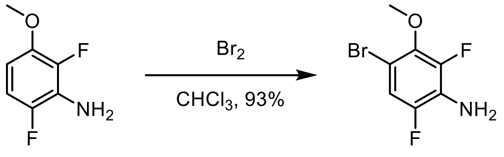

S47 **S48**

Bromide S48. Aniline **S47** (1.44 g, 9.05 mmol) was dissolved in $CHCl_3$ (150 mL). Then bromine (1.45 g, 463.6 µL, 9.05 mmol, 1 eq) in chloroform (30 mL) was added slowly at 0°C. The reaction was allowed to warm up to room temperature over 1 hour, then was quenched with sodium thiosulfate solution. The organic layer was separated, then dried and concentrated to afford the title compound, which was left without further purification as a beige solid (2 g, 8.4 mmol, 93%). ^{1}H NMR (600 MHz, $CDCl_3$) δ 7.01 (m, 1H), 3.89 (t, J = 1.1 Hz, 3H), 3.77 (t, J = 1.0 Hz, 2H). ^{13}C NMR (151 MHz, $CDCl_3$) δ 147.73 (dd, J = 239.6, 7.2 Hz), 145.79 (dd, J = 243.1, 8.2 Hz), 142.47 (dd, J = 11.5, 3.8 Hz), 124.94 (dd, J = 17.3, 14.8 Hz), 114.16 (dd, J = 22.4, 3.3 Hz), 102.60 (dd, J = 11.1, 3.5 Hz), 61.75 (d, J = 4.1 Hz). HRMS-APCI (m/z) Calculated for $C_7H_6ONF_2Br$ $[M+H]^+$, 239.9653; found 240.0649.

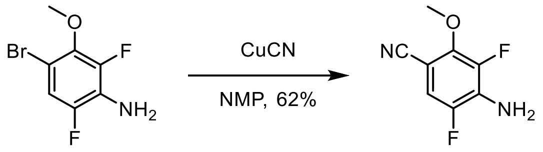

S48 S49

Nitrile S49. In a microwave vial was added **S48** (2.0 g, 8.4 mmol) and CuCN (978 mg, 10.9 mmol, 1.3 eq). The vial was purged with N_2 for 10 minutes. Then, 15 mL degassed NMP was added, the tube was sealed and reaction was heated to 180 °C under microwave irradiation for 90 minutes. The reaction was cooled, poured over ice water ammonia solution and extracted with 50:50 hexane:EtOAc 3x. The combined organic layers were sequentially washed with water, then concentrated. The resulting residue was purified by silica gel flash chromatography (50% CH_2Cl_2 in hexane) to afford the title compound as a white solid (962 mg, 5.22 mmol, 62%). ^{1}H NMR (400 MHz, $CDCl_3$) δ 6.98 (dd, *J* = 9.9, 2.1 Hz, 1H), 4.32 (s, 2H), 4.04 (d, *J* = 2.1 Hz, 3H). ^{13}C NMR (101 MHz, $CDCl_3$) δ 147.51 (dd, *J* = 10.4, 2.6 Hz), 145.98 (dd, *J* = 238.2, 6.8 Hz), 143.32 (dd, *J* = 241.4, 7.8 Hz), 131.08 (dd, *J* = 16.9, 13.8 Hz), 115.83 (t, *J* = 3.9 Hz), 114.05 (dd, *J* = 22.1, 3.1 Hz), 92.09 (dd, *J* = 10.7, 4.9 Hz), 62.18 (d, *J* = 5.7 Hz). HRMS-APCI (m/z) Calculated for $C_8H_7ONF_2$ $[M+H]^+$, 185.0521; found 185.0517.

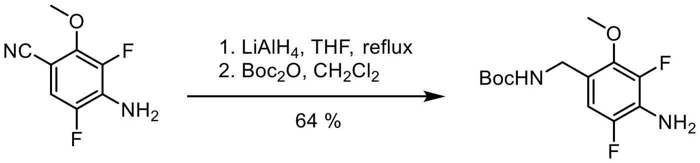

S49 S50

Aniline S50. $LiAlH_4$ (705 mg, 15.6 mmol, 3 eq) was suspended in dry THF (30 mL). Then, nitrile **S49** (960 mg, 5.21 mmol) in dry THF (40 mL) was added dropwise. The reaction was refluxed for 16 hours, then allowed to cool to room temperature. The reaction was diluted with Et_2O (30 mL), then quenched with 700 µL water, then 700 µL 10% NaOH, followed by 2.1 mL water (*Fieser workup*). This was stirred for 15 minutes, after which $MgSO_4$ was added, and the suspension was stirred a further 15 minutes. The solids were filtered off, and the filtrate concentrated to afford the intermediate amine. ^{1}H NMR (400 MHz, $CDCl_3$) δ 6.75 (dd, *J* = 10.8, 2.3 Hz, 1H), 3.90 (d, *J* = 1.6 Hz, 3H), 3.75 (s, 2H), 3.69 (s, 2H). This was immediately dissolved in CH_2Cl_2 (40 mL), to which Boc_2O (970 mg, 4.43 mmol, 0.85 eq) in CH_2Cl_2 (15 mL) was added dropwise at room temperature. The reaction was stirred at this temperature for 16 hours, then concentrated. The residue was purified by silica gel flash chromatography (1% acetone in CH_2Cl_2) to afford the title compound as a white solid (966 mg, 3.35 mmol, 64%). ^{1}H NMR (400 MHz, $CDCl_3$) δ 6.74 (d, *J* = 10.7 Hz, 1H), 4.88 (s, 1H), 4.19 (d, *J* = 4.6 Hz, 2H), 3.88 (s, 3H), 3.42 (s, 2H), 1.43 (s, 9H). ^{13}C NMR (151 MHz, $CDCl_3$) δ 155.92, 147.48 (dd, *J* = 236.5, 6.6 Hz), 145.27 (dd, *J* = 241.0, 7.5 Hz), 142.32 (dd, *J* = 9.3, 3.2 Hz), 124.38 (dd, *J* = 17.6, 14.4 Hz), 120.42 (d, *J* = 7.7 Hz), 110.60 – 109.53 (m), 79.61, 61.66, 39.32, 28.54. HRMS-EI (m/z) Calculated for $C_{13}H_{18}F_2N_2O_2Na$ $[M+Na]^+$, 311.1178; found 311.1178.

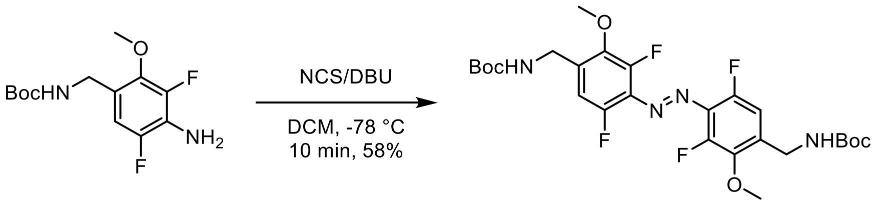

S50 → F$_4$,m-OMe$_2$-NHBoc

F$_4$,*m*-OMe$_2$-NHBoc. To a solution of **S50** (966 mg, 3.35 mmol) in CH_2Cl_2 (52 mL) was added DBU (1 mL, 6.70 mmol, 2 eq). The solution was stirred at room temperature for 5 min before being cooled down to −78 °C. NCS (902 mg, 6.70 mmol, 2 eq) was added. The orange solution was stirred for 10 min at −78 °C before quenching by addition of a saturated bicarbonate solution (50 mL). The organic layer was separated, washed sequentially with 50 mL of water (3 x) and 50 mL of 1 N HCl, and concentrated to dryness in vacuo. The residue was purified by silica gel flash chromatography (1 to 5% acetone in CH_2Cl_2) to afford the title compound as an orange solid (520 mg, 1.94 mmol, 58%). ^{1}H NMR (600 MHz, $CDCl_3$) δ 7.00 (d, *J* = 10.7 Hz, 1H), 4.99 (s, 1H), 4.37 (d, *J* = 6.2 Hz, 2H), 3.99 (d, *J* = 1.5 Hz, 3H), 1.46 (s, 9H). ^{13}C NMR (151 MHz, $CDCl_3$) δ 155.79, 150.75 (d, *J* = 258.0 Hz), 148.50 (d, *J* = 262.2 Hz), 142.31, 136.18, 131.41 (d, *J* = 9.9 Hz), 111.45 – 110.68 (m), 80.04, 61.67 (d, *J* = 6.0 Hz), 39.59, 28.38. HRMS-EI (m/z) Calculated for $C_{26}H_{32}F_4N_4O_6Na$ $[M+Na]^+$, 595.2150; found 595.2139.

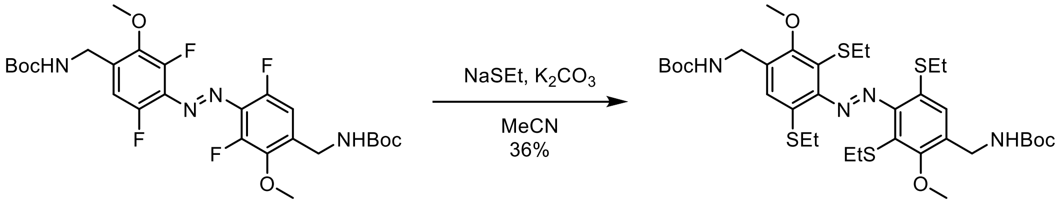

F$_4$,m-OMe$_2$-NHBoc → (SEt)$_4$,m-OMe$_2$-NHBoc

(SEt)$_4$,*m*-OMe$_2$-NHBoc. F$_4$,*m*-OMe$_2$-NHBoc (100 mg, 174.7 µmol), Sodium ethanethiolate (80%, 147 mg, 1.40 mmol, 8 eq) and potassium carbonate (97 mg, 699 µmol, 4 eq) were suspended in MeCN (2 mL) and stirred at room temperature for 16 hours under N_2 in a sealed tube. The mixture was diluted in CH_2Cl_2, then washed with water. The organic layer was concentrated, then purified by silica gel flash chromatography (0 to 1% Acetone in CH_2Cl_2) to afford the title compound as a red solid (36 mg, 63 µmol, 36%).^{1}H NMR (600 MHz, $CDCl_3$) δ 7.35 (s, 2H), 5.03 (t, *J* = 6.3 Hz, 2H), 4.41 (d, *J* = 6.3 Hz, 4H), 3.97 (s, 6H), 2.89 (m, 8H), 1.49 (s, 18H), 1.26 (t, *J* = 7.3 Hz, 6H), 1.13 (t, *J* = 7.4 Hz, 6H). ^{13}C NMR (151 MHz, $CDCl_3$) δ 156.60, 156.05, 152.25, 133.27, 128.78, 127.38, 126.90, 79.77, 60.76, 40.41, 30.03, 28.57, 28.10, 14.95, 13.75. HRMS-EI (m/z) Calculated for $C_{34}H_{53}S_4N_4O_6$ $[M+H]^+$, 741.2842; found 741.2832.

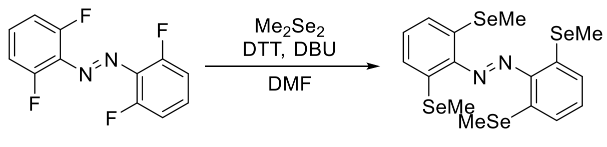

F$_4$-H → (SeMe)$_4$-H

(SeMe$_4$)-H. Dry Dithiothreitol (61 mg, 393 µmol, 2.5 eq) was dissolved in dry DMF (2mL) under N_2 in a sealed tube. Dimethyldiselenide (45 µL, 472 µmol, 3 eq) and degassed DBU (211 uL, 1.42 mmol, 9 eq) was added via syringe. The solution was stirred at room temperature for 30 minutes. The solution was transferred into a separate vial containing **F$_4$-H** (40 mg, 157 µmol, 1 eq) in Dry DMF (1 mL) under N_2. The reaction was stirred for 3 hours at room temperature, after which water was added. The organic precipitate was extracted with EtOAc (2x). The combined organic layers were washed with water (2x), then concentrated. The resulting residue was purified by silica gel flash chromatography (40% CH_2Cl_2 in hexane) to afford the title compound as a sparkly red solid (41 mg, 73 µmol, 47%). ^{1}H NMR (600

MHz, $CDCl_3$) δ 7.24 (d, *J* = 1.4 Hz, 4H), 7.18 (dd, *J* = 8.7, 6.7 Hz, 2H), 2.23 (s, 12H). ^{13}C NMR (151 MHz, $CDCl_3$) δ 148.23, 134.28, 130.02, 124.72, 7.09. HRMS-EI (m/z) Calculated for $C_{16}H_{19}N_2Se_4$ $[M+H]^+$, 554.8219; found 554.8227.

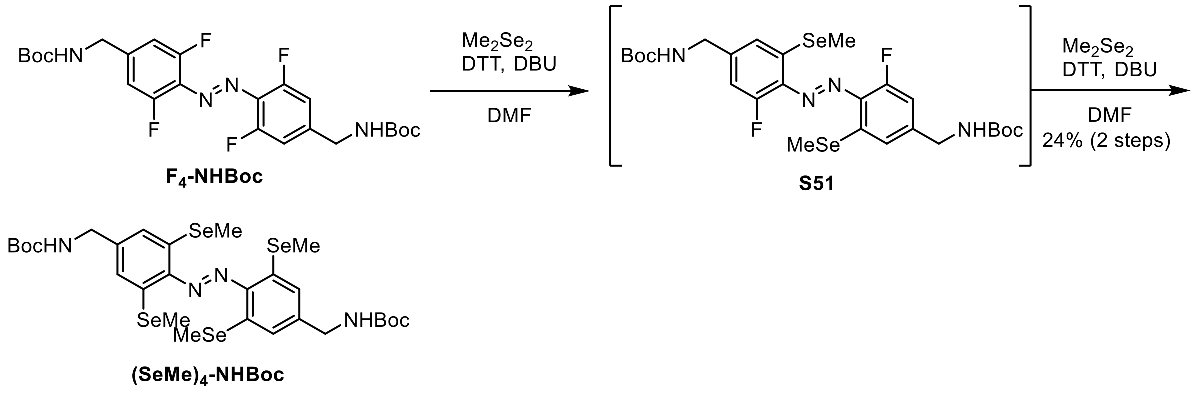

(SeMe₄)-NHBoc. Dry Dithiothreitol (53 mg, 341 µmol, 2.5 eq) was dissolved in dry DMF (1.5 mL) under N_2 in a sealed tube. Dimethyldiselenide (33 µL, 472 µmol, 3 eq) and degassed DBU (211 uL, 683 µmol, 5 eq) was added via syringe. The solution was stirred at room temperature for 30 minutes. The solution was transferred into a separate vial containing **F₄-NHBoc** (70 mg, 136 µmol, 1 eq) in Dry DMF (1 mL) under N_2. The reaction was stirred for 3 hours at room temperature, after which water was added. The organic precipitate was extracted with EtOAc (2x). The combined organic layers were washed with water (2x), then concentrated. The resulting residue purified by silica gel flash chromatography (100% CH_2Cl_2) to afford the title compound as an orange film (30 mg, 45 µmol, 33%). ^1H NMR (400 MHz, $CDCl_3$) δ 7.16 (s, 2H), 7.07 – 7.03 (d, *J* = 10.8, 1.1 Hz, 2H), 4.97 (s, 2H), 4.38 (d, *J* = 6.6 Hz, 4H), 2.22 (s, 6H), 1.48 (s, 18H). Then, dry Dithiothreitol (21 mg, 136 µmol, 3 eq) was dissolved in dry DMF (1 mL) under N_2 in a sealed tube. Dimethyldiselenide (13 µL, 136 µmol, 3 eq) and degassed DBU (27 uL, 181 µmol, 4 eq) was added via syringe. The solution was stirred at room temperature for 30 minutes. The solution was transferred into a separate vial containing **S51** (30 mg, 45 µmol, 1 eq) in Dry DMF (1 mL) under N_2. The reaction was stirred for 16 hours at 30 °C, after which water was added. The organic precipitate was extracted with EtOAc (2x). The combined organic layers were washed with water (2x), then concentrated. The resulting residue purified by silica gel flash chromatography (3% MeCN in CH_2Cl_2) to afford the title compound as an orange solid (27 mg, 45 µmol, 73%) [24% over 2 steps]. ^1H NMR (600 MHz, $CDCl_3$) δ 7.12 (s, 4H), 4.90 (s, 2H), 4.33 (d, *J* = 6.2 Hz, 4H), 2.22 (s, 12H), 1.48 (s, 18H).^{13}C NMR (151 MHz, $CDCl_3$) δ 156.08, 147.50, 141.19, 134.58, 123.73, 79.95, 28.56, 7.13. HRMS-EI (m/z) Calculated for $C_{28}H_{40}N_4O_4Se_4$ $[M+H]^+$, 814.9791; found 814.9798.

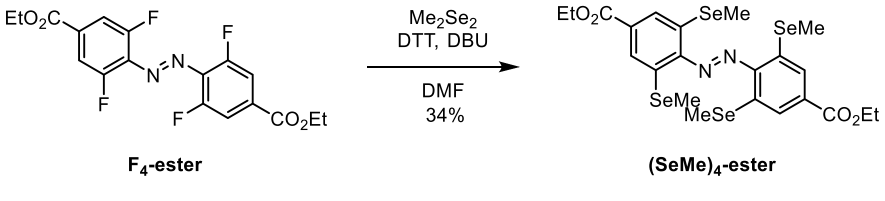

(SeMe₄)-ester. Dry Dithiothreitol (125 mg, 810 µmol, 3 eq) was dissolved in dry DMF (2 mL) under N_2 in a sealed tube. Dimethyldiselenide (81 µL, 472 µmol, 3 eq) and degassed DBU (322 uL, 1.42 mmol, 8 eq) was added via syringe. The solution was stirred at room temperature for 30 minutes. The solution was transferred into a separate vial containing **F₄-ester** (100 mg, 270 µmol, 1 eq) in Dry DMF (1 mL) under N_2. The reaction was stirred for 16 hours at room temperature after which a dark purple solution had formed. The mixture was poured into a separating funnel containing Et_2O (20 mL) and

water (20 mL). The mixture was shook gently (which turned brown/orange), then the water layer removed. The organic layer was washed with water (2x), then concentrated. The resulting residue was purified by silica gel flash chromatography (10 to 100% CH_2Cl_2 in hexane) to afford the title compound as a brown solid (62 mg, 93 μmol, 34%). ^{1}H NMR (600 MHz, $CDCl_3$) δ 7.89 (s, 4H), 4.43 (q, J = 7.1 Hz, 4H), 2.31 (s, 12H), 1.43 (t, J = 7.1 Hz, 6H).^{13}C NMR (151 MHz, $CDCl_3$) δ 165.73, 150.35, 134.83, 131.18, 125.89, 61.75, 14.49, 7.50. HRMS-EI (m/z) Calculated for $C_{22}H_{27}N_2O_4Se_4$ $[M+H]^+$, 700.8634; found 700.8637.

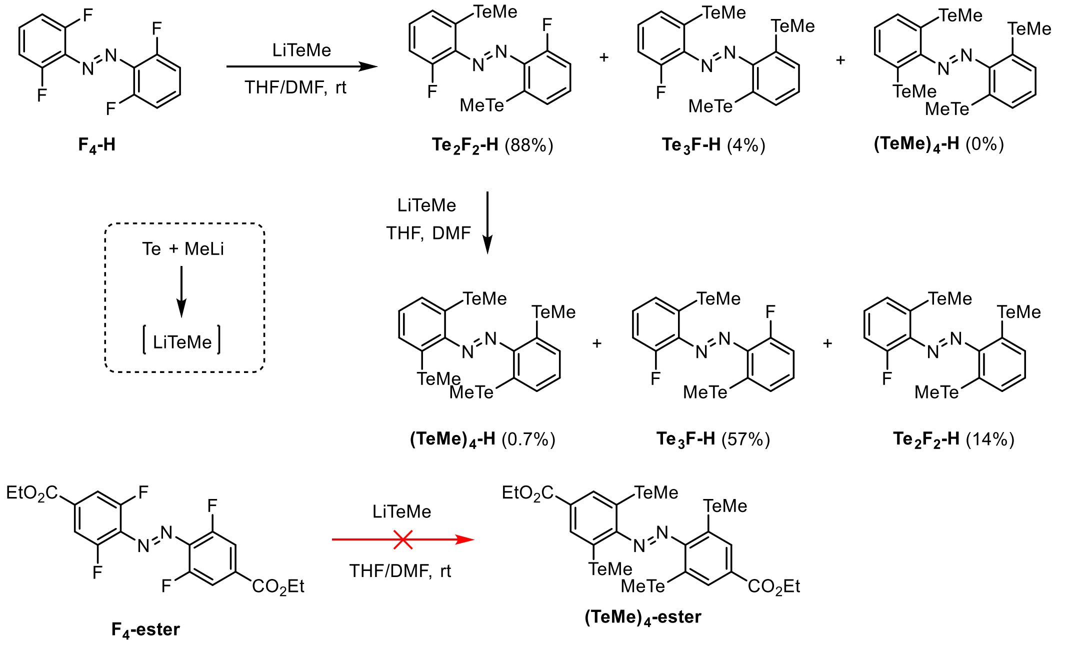

Scheme S4. Synthesis telluro azobenzene derivatives. The telluride nucleophile was prepared using MeLi and elemental tellurium. It was necessary to prepare the the **Te₄** derivative step-wise via **Te_2F_2-H**. A mixture of mono and di-substituted telluride was obtained with 20 eq LiTeMe for the p-Ester derivative (**$TeMe_4$)-ester**. Optimisation table is found overleaf

- Optimisation -

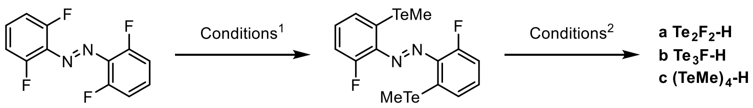

F_4-H **Te_2F_2-H**

	**Conditions[1]**	Yield
1	Me_2Te_2 (2.5 eq), DTT (2.5 eq), DBU (5 eq), DMF 0.1M, rt, 16 h	**a** 1.4%
2	Me_2Te_2 (2.5 eq), DTT (2.5 eq), DBU (5 eq), DMF 0.1M, **40 °C**, 16 h	No conv.
3	Me_2Te_2 (2.5 eq), DTT (2.5 eq), DBU (5 eq), DMF 0.1M, **30 °C 1 day, 35 °C 1 day**	No conv.
4	Me_2Te_2 (2.5 eq), $NaBH_4$ (5 eq), DMF 0.1M, rt, 16 h	(Decomp)
5	Me_2Te_2 (2.5 eq), DTT (2.5 eq), DBU (5 eq), DMF 0.1M, rt, overnight, **Et_2O/water workup**	**a** 10%
6	Te (5 eq), MeLi (5 eq), THF/DMF, 16 h, Et_2O/water workup	**a** 88% **b** 4%
7	Te (10 eq), MeLi (10 eq), THF/DMF, 16 h , Et_2O/water workup	**a** 49% **b** 10%
	**Conditions[2]**	**Yield**
8	Me_2Te_2 (3 eq), DTT (3 eq), DBU (4 eq), DMF 0.1M, rt to 40 to 50 to 60 °C	No conv.
9	Te (10 eq), MeLi (10 eq), THF/DMF, 40 °C, 2 days, then Te (10 eq), MeLi (10 eq), 40 °C, 1 more day.	**a** 57% **b** 14% **c** 0.7%

Table S1. Optimisation table for telluro azobenzene derivatives. It was necessary to prepare the tetra telluride step-wise via the ditelluride (conditions 2). Conditions 1 (analogous to Selenolate chemistry) [15] were less successful in generating products

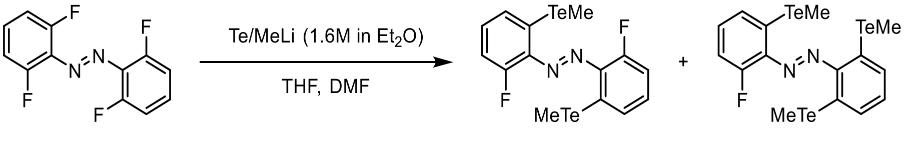

F_4-H **Te_2F_2-H** (88%) **Te_3F-H** (4%)

Te_2F_2-H and **Te_3F-H.** Te powder (500 mg, 3.9 mmol) was added to dry THF (10 mL). MeLi (1.6M in Et_2O, 2.4 mL, 4 mmol, 1.2 eq) was added and the solution was stirred at room temperature for 3 hours. The green/grey suspension was transferred into a separate vial containing **F_4-H** (200 mg, 786 µmol, 0.2 eq) in Dry DMF (3 mL) under N_2. The reaction was stirred for 16 hours at room temperature. The mixture was poured into a separating funnel containing Et_2O (100mL) and water (50 mL). The mixture was shook gently, then the water layer removed. The organic layer was dry loaded onto silica gel, and purified by silica gel flash chromatography (5% Hexane to 10% Hexane in CH_2Cl_2) to afford **Te_2F_2-H** as a sparkly dark purple solid. **Te_3F-H** was obtained with 15% hexane in CH_2Cl_2, then purified further using preparative TLC (15% hexane in CH_2Cl_2). **Te_2F_2-H**: ^{1}H NMR (600 MHz, $CDCl_3$) δ 7.48 (d, J = 7.9 Hz, 2H), 7.32 (td, J = 7.9, 5.2 Hz, 2H), 7.22 (ddd, J = 10.5, 8.0, 1.2 Hz, 2H), 1.96 (s, 6H). ^{13}C NMR (151 MHz, $CDCl_3$) δ 162.58 (d, J = 263.7 Hz), 139.50 (d, J = 9.9 Hz), 130.82 (d, J = 8.4 Hz), 128.40 (d, J = 3.8 Hz), 117.43 (d, J = 3.6 Hz), 113.13 (d, J = 19.8 Hz), -11.09. ^{19}F NMR (377 MHz, $CDCl_3$) δ -113.50. HRMS-EI (m/z) Calculated for $C_{14}H_{12}N_2F_2Te_2OH$ $[M+O+H]^+$, 520.9097; found 520.9094. **Te_3F-H**: ^{1}H NMR (600 MHz, $CDCl_3$) δ 7.47 (d, J = 7.6 Hz, 2H), 7.43 (d, J = 7.9 Hz, 1H), 7.25 (dd, J = 8.0, 5.1 Hz, 1H), 7.15 (t, J = 9.1 Hz, 1H), 7.08 (t, J = 7.6 Hz, 1H), 2.04 (s, 5H), 1.99 (s, 3H). ^{13}C NMR (151 MHz, $CDCl_3$) δ 160.77 (d, J = 261.5 Hz), 153.09, 140.91 (d, J = 8.9 Hz), 131.03 (d, J = 8.3 Hz), 130.98, 130.09, 129.58 (d, J = 8.9 Hz), 121.75, 115.02 (d, J = 2.1 Hz), 113.78 (d, J = 19.8 Hz), -12.00, -13.99. HRMS-EI (m/z) Calculated for $C_{15}H_{15}N_2FTe_3OH$ $[M+O+H]^+$, 642.8381; found 642.8379.

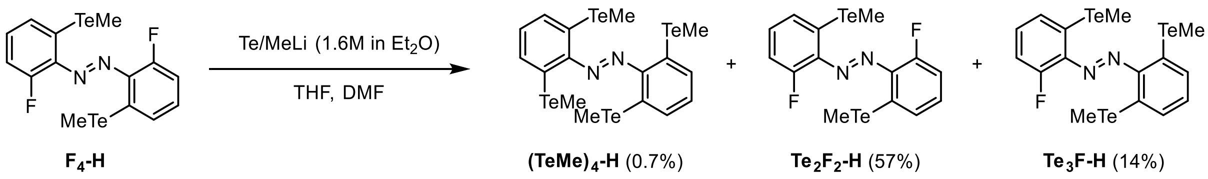

(TeMe)$_4$-H. Te powder (500 mg, 3.9 mmol) was added to dry THF (10 mL). MeLi (1.6M in Et_2O, 2.4 mL, 4 mmol, 1.2 eq) was added and the solution was stirred at room temperature for 3 hours. The green/grey suspension was transferred into a separate vial containing **Te$_2$F$_2$-H** (190 mg, 379 µmol, 1 eq) in Dry DMF (1 mL) under N_2. The reaction was stirred for 48 hours at 40 °C. In a separate flask, Te powder (500 mg, 3.9 mmol) was added to dry THF (10 mL). MeLi (1.6M in Et_2O, 2.4 mL, 4 mmol, 1.2 eq) was added and the solution was stirred at room temperature for 3 hours. This mixture was added to the solution of **Te$_2$F$_2$-H**, and this was stirred for a further 48 hours at 40 °C. The mixture was poured into a separating funnel containing Et_2O (50 mL) and water (30 mL). The mixture was shook gently, then the water layer removed. The organic layer was dry loaded onto silica gel, and purified by silica gel flash chromatography (5% Hexane to 10% Hexane in CH_2Cl_2) to afford **Te$_2$F$_2$-H** as a sparkly dark purple solid (108 mg, 215 µmol, 57%). The trisubstituted compound was obtained by silica gel flash chromatography (15% hexane in CH_2Cl_2; 33 mg, 53 µmol, 14%). The tetra substituted compound was isolated using 20% Hexane in CH_2Cl_2, then purified further using preparative TLC (20% Hexane in CH_2Cl_2) to afford **(TeMe)$_4$-H** (2 mg, 2.7 µmol, 0.7%). ^{1}H NMR (400 MHz, CD_2Cl_2) δ 7.43 (d, J = 7.7 Hz, 4H), 7.06 (t, J = 7.6 Hz, 2H), 2.02 (s, 12H). ^{13}C NMR (151 MHz, CD_2Cl_2) δ 154.04, 131.92, 130.57, 119.75, -14.68. HRMS-EI (m/z) Calculated for $C_{16}H_{18}N_2Te_4OH$ $[M+H+O]^+$, 766.7676; found 766.7682.

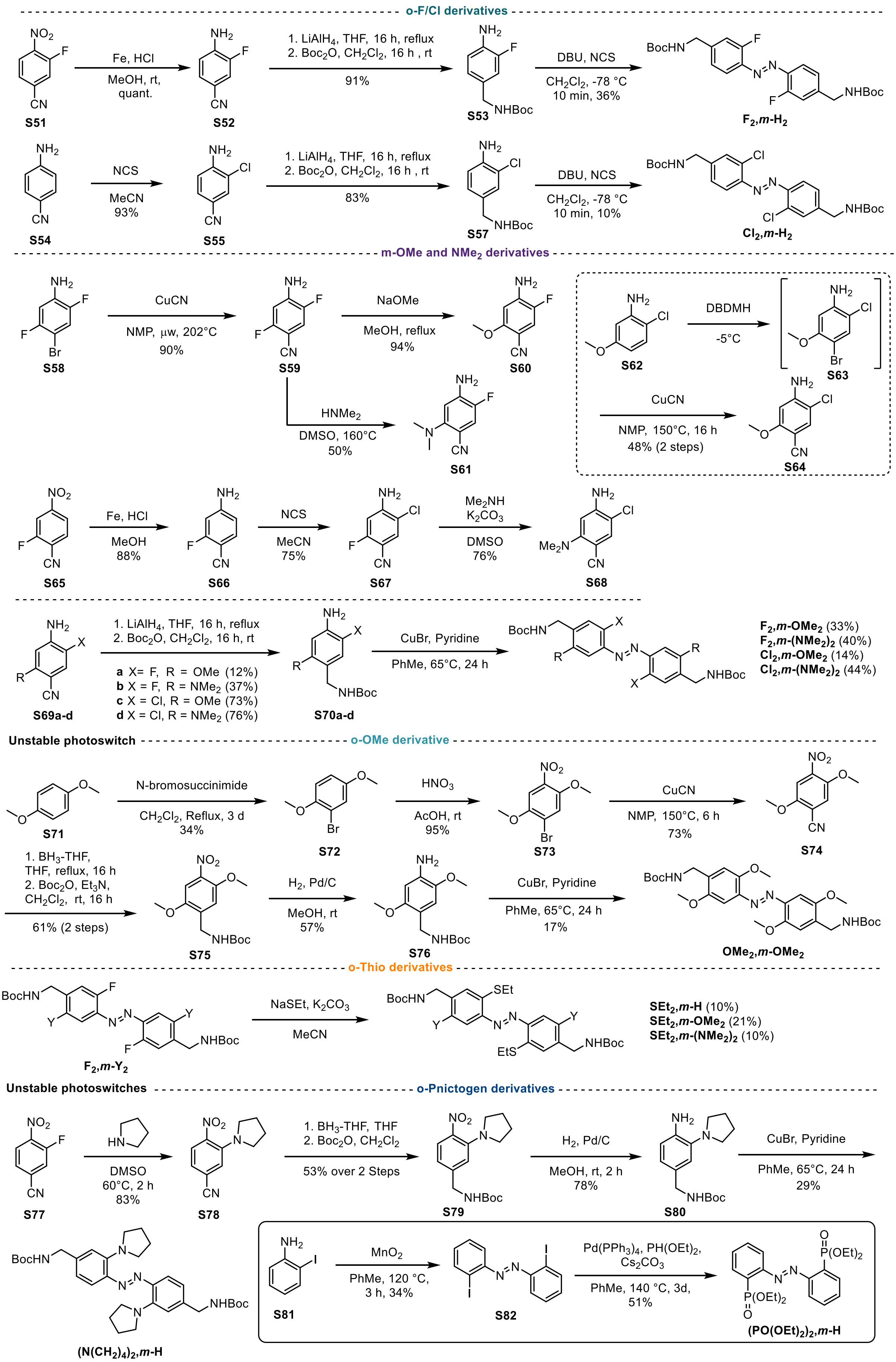

Scheme 1. Preparation of di ortho and meta substituted azobenzene scaffolds.

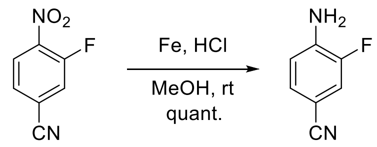

S51 **S52**

Aniline S52. Prepared according to a modified literature procedure.[17] Nitro compound **S51** (1 g, 6 mmol) was dissolved in MeOH (10 mL), then iron powder (1 g, 18 mmol, 3 eq) was added. HCl (4 mL) was added carefully and the reaction was stirred at room temperature for 2h. The solution was filtered over celite, then concentrated. The residue was extracted with EtOAc and basified with 10% $NaHCO_3$. The combined organic layer was dried and filtered to afford the title compound (820 mg, 6 mmol, quant). ^1H NMR (400 MHz, DMSO) δ 7.50 (dd, J = 11.7, 1.9 Hz, 1H), 7.30 (dd, J = 8.3, 1.9 Hz, 1H), 6.80 (t, J = 8.7 Hz, 1H), 6.24 (s, 2H). Data consistent with that given in the literature. [17]

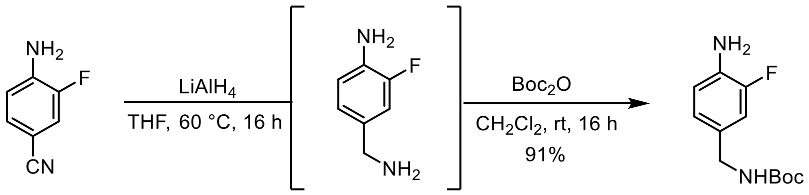

S52 **S53**

Aniline S53. To a suspension of $LiAlH_4$ (686 mg, 18.1 mmol, 3 eq) in dry THF (25 mL) was added **S52** (820 mg, 6 mmol, 1.0 eq) in THF (20 mL). The suspension was stirred at 60 °C for 16 hours. The reaction mixture was cooled, diluted in Et_2O (10 mL), then water (685 µL), 2.5M NaOH (685 µL), and water (2 mL) was added dropwise. The suspension was stirred for 15 minutes, and then anhydrous $MgSO_4$ was added. The solution was filtered. The filtrate was concentrated to afford the crude amine intermediate. The residue was dissolved in CH_2Cl_2 (35 mL) and Boc_2O (1.25 g, 5.7 mmol, 0.95 eq) was added. The reaction was stirred overnight. The solution was concentrated, then purified by silica gel flash chromatography (0 to 2% Acetone in CH_2Cl_2) to afford the title compound as a yellow oil (1.32 g, 5.5 mmol, 91%). ^1H NMR (400 MHz, $CDCl_3$) δ 6.92 (dd, J = 11.6, 1.9 Hz, 1H), 6.87 – 6.79 (m, 1H), 6.71 (dd, J = 9.0, 8.1 Hz, 1H), 4.77 (s, 1H), 4.18 (d, J = 5.9 Hz, 2H), 3.69 (br s, 2H), 1.45 (s, 9H). ^{13}C NMR (101 MHz, $CDCl_3$) δ 155.94, 151.69 (d, J =240.1 Hz) 133.74 (d, J =12.9 Hz), 129.83 (d, J = 6.4 Hz), 123.73 (d, J = 3.7 Hz), 116.98 (d, J = 3.79 Hz), 114.73 (d, J = 18.7 Hz), 79.49, 43.98, 28.41. HRMS-ESI (m/z) Calculated for $C_{12}H_{18}FN_2O_2$ $[M+H]^+$, 241.1347; found 241.1349.

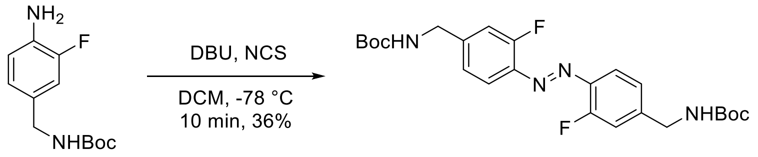

S53 **F_2,*m*-H_2**

F_2,*m*-H_2. To a solution of **S53** (415 mg, 1.17 mmol) in CH_2Cl_2 (26 mL) was added DBU (516 µL, 3.45 mmol, 2 eq). The solution was stirred at room temperature for 5 min before being cooled down to −78 °C. NCS (526 mg, 3.45 mmol, 2 eq) was added. The orange solution was stirred for 10 min at −78 °C before quenching with saturated bicarbonate solution (65 mL). The organic layer was separated, washed sequentially with water (5 x 65mL), dried over anhydrous sodium sulfate, and concentrated to dryness in vacuo. The residue was purified by silica gel flash chromatography (0 to 2% Acetone in CH_2Cl_2) to afford the title compound as an orange solid as a mixture of isomers (150 mg, 315 µmol, 36%). *(E)*-**F_2,*m*-H_2**: ^1H NMR (400 MHz, $CDCl_3$) δ 6.96 (dd, J = 8.2, 1.7 Hz, 2H), 6.92 – 6.79 (m, 4H), 4.90 (s, 2H), 4.25 (d, J = 6.2 Hz, 4H), 1.44 (s, 18H). ^{13}C NMR (151 MHz, $CDCl_3$) δ 160.57 (d, J = 258.9 Hz),

156.02, 145.33, 140.01 (d, J = 6.8 Hz), 123.14, 118.17, 115.72 (d, J = 20.5 Hz), 80.13, 44.17, 28.52. HRMS-ESI (m/z) Calculated for $C_{24}H_{30}F_2N_4O_4$ $[M+H]^+$,477.2308; found 477.2307.

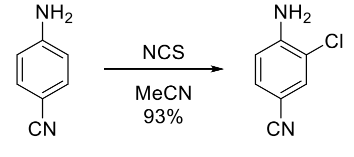

S54 **S55**

Aniline S55. Nitrile **S54** (1 g, 8.5 mmol), then NCS (1.24 g, 9.3 mmol, 1.1 eq) in MeCN (10 mL) were stirred at 90°C for 2 h. The mixture was cooled, concentrated, then dissolved in 50 mL CH_2Cl_2. The organic layer was washed with 5% NaOH, then dried and concentrated to afford the title compound as a beige solid (1.2 g, 7.86 mmol, 93%). ^1H NMR (400 MHz, $CDCl_3$) δ 7.53 (d, J = 1.8 Hz, 1H), 7.34 (dd, J = 8.4, 1.9 Hz, 1H), 6.75 (d, J = 8.4 Hz, 1H), 4.55 (s, 2H). Data consistent with that given in the literature. [18]

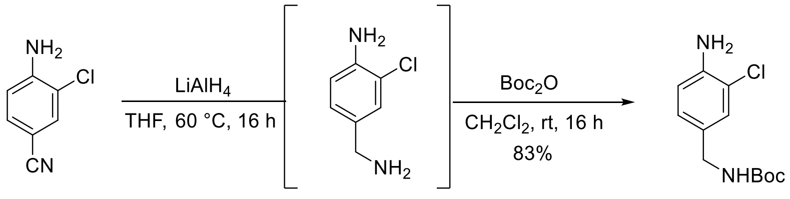

S55 **S57**

Aniline S57. To a suspension of $LiAlH_4$ (746 mg, 19.66 mmol, 3 eq) in dry THF (30 mL) was added **S55** (1 g, 6.55 mmol, 1.0 eq) in THF (20 mL). The suspension was refluxed for 16 hours. The reaction mixture was cooled, diluted in Et_2O (30 mL), then water (750 µL), 2.5M NaOH (750 µL), and water (2.25 mL) was added dropwise. The suspension was stirred for 15 minutes, and then anhydrous $MgSO_4$ was added. The solution was filtered, filtrate concentrated to afford the crude amine. The residue was dissolved in CH_2Cl_2 (40 mL) and Boc_2O (1.36 g 6.23 mmol, 0.95 eq) was added. The reaction was stirred overnight. The solution was concentrated, then purified by silica gel flash chromatography (3% acetone in CH_2Cl_2), and the pure fractions were combined to afford the title compound as a white solid (1.4 g, 5.45 mmol, 83%).^1H NMR (400 MHz, $CDCl_3$) δ 7.20 (d, J = 2.0 Hz, 1H), 7.04 – 6.99 (m, 1H), 6.77 (d, J = 8.2 Hz, 1H), 4.77 (s, 1H), 4.47 – 4.18, 4.20 (d, J = 5.8 Hz, 2H), 1.48 (s, 9H). Data consistent with that given in the literature. [19]

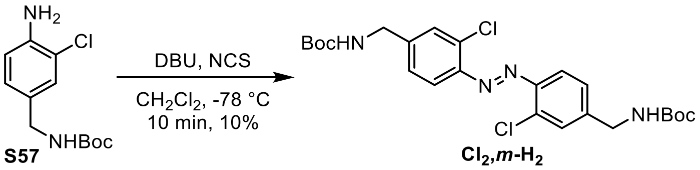

S57 **Cl_2,*m*-H_2**

Cl_2,*m*-H_2: To a solution of **S57** (1.4 g, 5.45 mmol) in CH_2Cl_2 (80 mL) was added DBU (1.63 mL, 10.9 mmol, 2 eq). The solution was stirred at room temperature for 5 min before being cooled down to −78 °C. NCS (1.46 g, 10.9 mmol, 2 eq) was added. The orange solution was stirred for 10 min at −78 °C before quenching with saturated bicarbonate solution (50 mL). The organic layer was separated, washed sequentially with 15 mL of water (5 x 50 mL) and 1N HCl (50 mL), dried over anhydrous sodium sulfate, and concentrated to dryness in vacuo. The residue was purified by silica gel flash chromatography (1% EtOAc in CH_2Cl_2) to afford the title compound as an orange solid as a mixture of isomers (141 mg, 277 µmol, 10%). *(E)*-**Cl_2,*m*-H_2**: ^1H NMR (400 MHz, $CDCl_3$) δ 7.74 (d, J = 8.3 Hz, 2H), 7.48 (d, J = 1.8 Hz, 2H), 7.25 (d, J = 8.3 Hz, 2H), 4.97 (s, 2H), 4.36 (d, J = 6.2 Hz, 4H), 1.48 (s, 18H). ^{13}C NMR (151 MHz, $CDCl_3$) δ 155.99, 148.04, 144.14, 136.28, 129.40, 126.40, 118.38, 80.16, 44.05, 28.53. HRMS-EI (m/z) Calculated for $C_{24}H_{31}O_4N_4Cl_2$ $[M+H]^+$, 509.1717; found 509.1717

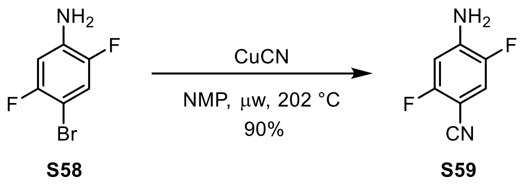

Nitrile S59. Aryl bromide **S58** (2.5 g, 12.02 mmol) was dissolved in NMP (12.5 mL) under N_2 in a microwave vial. CuCN (1.3 g, 13.2 mmol, 1.2 eq) was added and the reaction was stirred for 90 minutes at 202°C under microwave irradiation. The reaction was cooled to room temperature, then 15% ammonium hydroxide solution was added. The reaction was extracted with 50:50 EtOAC:Hexane 3x. The combined organic layers were washed with water 3x, then 5% LiCl solution. The organic layer was concentrated to afford a crude residue that was left without further purification (1.67g, 10.84 mmol, 90%). ^{1}H NMR (400 MHz, $CDCl_3$) δ 7.17 (dd, *J* = 10.2, 5.7 Hz, 1H), 6.51 (dd, *J* = 9.9, 7.1 Hz, 1H), 4.41 (s, 2H). Data consistent with that given in the literature. [[20]]

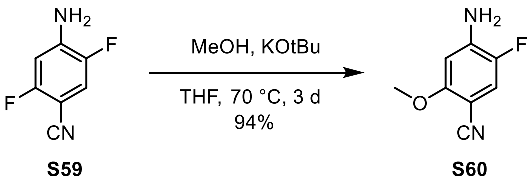

Aniline S60. To a cooled (0°C) solution of methanol (43 mL) in anhydrous THF (25 ml) under N_2 was added KOtBu (4.8 g, 42.8 mmol, 2 eq). Then **S59** (1.65 g, 10.7 mmol) was added to the solution. The reaction mixture was heated to 70°C and stirred for 3 days. The solvent was removed, then diethyl ether was added. The organic phase was washed with saturated bicarbonate solution, dried, then concentrated to afford the title compound as a white solid (1.68 g, 10.1 mmol, 94%). ^{1}H NMR (400 MHz, $CDCl_3$) δ 7.12 (d, *J* = 10.3 Hz, 1H), 6.27 (d, *J* = 7.2 Hz, 1H), 4.28 (s, 2H), 3.84 (s, 3H). Data consistent with that given in the literature. [[21]]

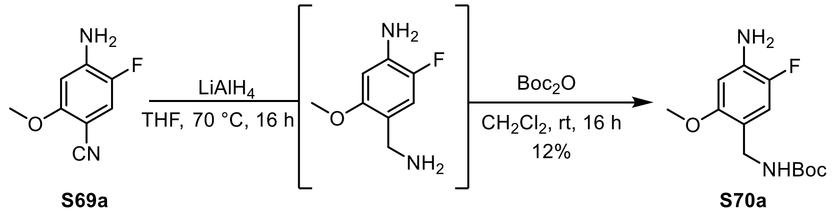

Aniline S70b. To a suspension of $LiAlH_4$ (136 mg, 3.58 mmol, 2.5 eq) in dry THF (10 mL) was added **S69a** (238 mg, 2.38 mmol, 1.0 eq) in THF (7.5 mL). The suspension was refluxed for 16 hours. The reaction mixture was cooled, diluted in Et_2O (10 mL), then water (136 µL), 2.5M NaOH (136 µL), and water (0.4 mL) was added dropwise. The suspension was stirred for 15 minutes, and then anhydrous $MgSO_4$ was added. The solution was filtered, filtrate concentrated to afford the crude amine and imine intermediates. The residue was dissolved in CH_2Cl_2 (15 mL) and Boc_2O (312 mg, 1.43 mmol, 1.0 eq) was added. The reaction was stirred overnight. The solution was concentrated, then purified by silica gel flash chromatography (100% CH_2Cl_2), and the pure fractions were combined to afford the title compound as a white solid (45 mg, 0.17 mmol, 12%). ^{1}H NMR (400 MHz, $CDCl_3$) δ 6.89 (d, *J* = 11.0 Hz, 1H), 6.27 (d, *J* = 7.4 Hz, 1H), 4.94 (s, 1H), 4.15 (d, *J* = 6.0 Hz, 2H), 3.73 (m, 5H), 1.43 (s, 9H). ^{13}C NMR (101 MHz, $CDCl_3$) δ 155.98, 153.99 (d, *J* = 1.9 Hz), 145.61 (d, *J* = 231.2 Hz), 134.10 (d, *J* = 14.2 Hz), 116.95 (d, *J* = 5.2 Hz), 116.24 (d, *J* = 20.4 Hz), 99.97 (d, *J* = 3.2 Hz), 79.30, 55.84, 39.75, 28.55. HRMS-EI (m/z) Calculated for $C_{13}H_{19}O_3N_2FNa$ $[M+Na]^+$, 293.1272; found 293.1271

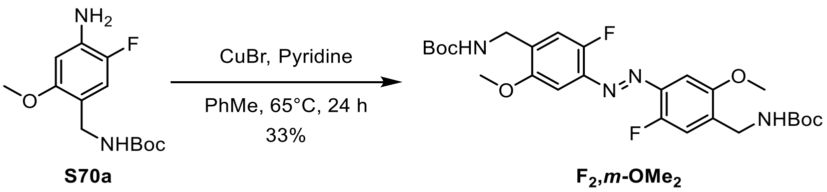

F_2,*m*-OMe$_2$. Aniline **S70a** (39 mg, 138 µmol) was dissolved in toluene (2 mL). Pyridine (22µL, 0.28 mmol, 2 eq) and CuBr (20 mg, 137 µmol, 1 eq) was added and the reaction was stirred under atmospheric air at 65 °C for 24 hours. The reaction was concentrated, then purified by silica gel flash chromatography (5% EtOAc in $CHCl_3$), and the pure fractions were combined to afford the title compound as an orange solid as a mixture of isomers (12.5 mg, 45 µmol, 33%). *(E)*-**F_2,*m*-OMe$_2$:** ^{1}H NMR (400 MHz, $CDCl_3$) δ 7.21 (d, *J* = 5.9 Hz, 1H), 7.14 (d, *J* = 10.5 Hz, 1H), 4.97 (s, 1H), 4.27 (d, *J* = 6.3 Hz, 2H), 3.82 (s, 3H), 1.40 (s, 9H). ^{13}C NMR (101 MHz, $CDCl_3$) δ 155.87, 155.17 (d, *J* = 228.69 Hz), 153.61 (d, *J* = 1.9 Hz), 139.55 (d, *J* = 7.4 Hz), 133.34 (d, *J* = 7.2 Hz), 117.00 (d, *J* = 22.8 Hz), 98.10, 79.73, 55.96, 39.98, 28.42. ^{19}F NMR (377 MHz, $CDCl_3$) δ -132.84 (s). HRMS-EI (m/z) Calculated for $C_{26}H_{35}O_6N_4F_2$ $[M+H]^+$, 537.2519; found 537.2519

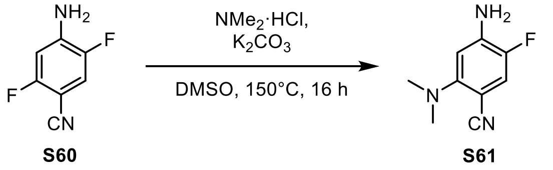

Amine S61. Nitrile **S60** (700 mg, 4.54 mmol) was dissolved in DMSO (15 mL) and, potassium carbonate (1.26 g, 9.08 mmol, 2 eq) and dimethylamine hydrochloride (1.46 g, 18.2 mmol, 4 eq) was added. The reaction was stirred at 80 °C for 16 hours, then for a further 4 hours at 150 °C after which the reaction was 60% complete. The solution was poured onto ice and extracted with Et_2O 5 x. The combined organic layers were washed with water, then concentrated. The residue was purified by silica gel flash chromatography (100% CH_2Cl_2) to afford the title compound as a white solid (456 mg, 2.54 mmol, 56 %). ^{1}H NMR (400 MHz, $CDCl_3$) δ 7.07 (d, *J* = 10.6 Hz, 1H), 6.22 (d, *J* = 7.7 Hz, 1H), 4.23 (s, 2H), 2.89 (s, 6H). ^{13}C NMR (101 MHz, $CDCl_3$) δ 154.28, 144.52 (d, *J* = 234.6 Hz), 140.11 (d, *J* = 13.4 Hz), 120.00 (d, *J* = 21.4 Hz), 119.56 (d, *J* = 2.1 Hz), 103.87 (d, *J* = 3.6 Hz), 90.19 (d, *J* = 8.1 Hz), 43.53. HRMS-EI (m/z) Calculated for $C_9H_{11}FN_3$ $[M+H]^+$, 180.0937; found 180.0932

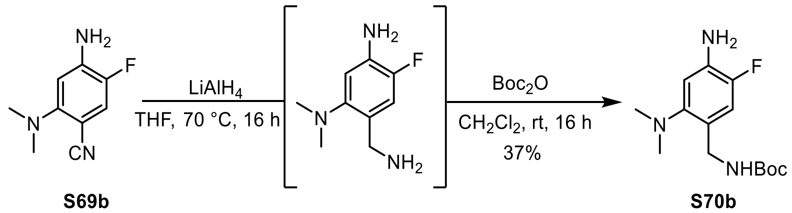

Aniline S70b. To a suspension of $LiAlH_4$ (160 mg, 4.18 mmol, 2.5 eq) in dry THF (10 mL) was added **S69b** (300 mg, 4.18 mmol, 1.0 eq) in THF (10 mL). The suspension was refluxed for 16 hours. The reaction mixture was cooled, diluted in Et_2O (10 mL), then water (160 µL), 2.5M NaOH (160 µL), and water (0.5 mL) was added dropwise. The suspension was stirred for 15 minutes, and then anhydrous $MgSO_4$ was added. The solution was filtered, filtrate concentrated to afford the crude amine and imine intermediate. The residue was dissolved in CH_2Cl_2 (15 mL) and Boc_2O (183 mg, 0.84 mmol, 0.5 eq) was added. The reaction was stirred overnight. The solution was concentrated, then purified by silica gel flash chromatography (10% EtOAc in CH_2Cl_2), and the pure fractions were combined to afford the title compound as a yellow oil (176 mg, 0.62 mmol, 37%). ^{1}H NMR (400 MHz, $CDCl_3$) δ 6.84 (dd, *J* = 11.6, 2.2 Hz, 1H), 6.50 (dd, *J* = 8.3, 1.6 Hz, 1H), 5.24 (s, 1H), 4.23 (d, *J* = 5.9 Hz, 2H), 3.72 (s, 2H), 2.54 (d, *J* = 2.0 Hz, 6H), 1.42 (s, 9H). ^{13}C NMR (101 MHz, $CDCl_3$) δ 156.15, 148.76 (d, *J* = 2.6 Hz), 147.77 (d, *J* = 235.01 Hz), 133.69 (d, *J* = 13.8 Hz), 123.73, 115.37 (d, *J* = 20.0 Hz), 108.49 (d, *J* = 3.9

Hz), 79.16, 40.34, 28.43. HRMS-EI (m/z) Calculated for $C_{14}H_{23}O_2N_3F$ $[M+H]^+$, 284.1769; found 284.1769

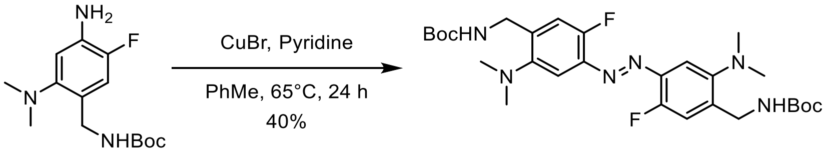

S70b **F2,*m*-(NMe2)2**

F_2,*m*-(NMe_2)$_2$. Aniline **S70b** (176 mg, 621 µmol) was dissolved in toluene (8 mL). Pyridine (100 µL, 1.24 mmol, 2 eq) and CuBr (89mg, 621 µmol, 1 eq) was added and the reaction was stirred under atmospheric air at 65 °C for 24 hours. The reaction was concentrated, then purified by silica gel flash chromatography (5% Acetone in CH_2Cl_2), and the pure fractions were combined to afford the title compound as an orange solid (70 mg, 123 µmol, 40%). (*E*)-**F_2,*m*-(NMe_2)$_2$:** ^{1}H NMR (500 MHz, $CDCl_3$) δ 7.51 (d, *J* = 7.0 Hz, 2H), 7.21 (d, *J* = 11.2 Hz, 2H), 5.18 (s, 2H), 4.46 (d, *J* = 7.4 Hz, 4H), 2.69 (s, 12H) 1.45, (s, 18H). ^{13}C NMR (126 MHz, $CDCl_3$) δ 157.05 (d, *J* = 218.6 Hz), 155.89, 148.68 (d, *J* = 2.5 Hz), 139.77, 139.61 (d, *J* = 7.5 Hz), 116.43 (d, *J* = 21.6 Hz), 108.33, 79.78, 45.03, 40.72, 28.45. HRMS-EI (m/z) Calculated for $C_{28}H_{41}O_4N_6F_2$ $[M+H]^+$, 563.3152; found 563.3148

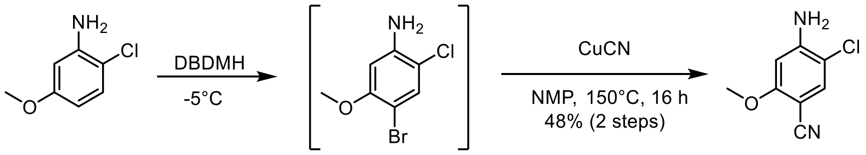

S62 **S63** **S64**

Nitrile S64. Aniline **S62** (3 g, 19.04 mmol) was dissolved in EtOAc (50 mL). The reaction was cooled to -5 °C and DBDMH was added portionwise over 20 minutes at this temperature. The reaction was stirred at -5 °C for a further hour. Then, the organic layer was washed with K_2CO_3 (2 g) in water (13 mL), then water (20 mL). The organic later was concentrated to afford the intermediate bromide **S63** without further purification. The residue was redissolved in NMP (30 mL) and N_2 was bubbled through the reaction mixture for 5 minutes, then CuCN (2.56 g, 28.55 mmol, 1.5 eq) was added and the reaction was stirred at 150°C under N_2 for 16 hours. The reaction was cooled, poured over ice water 15% ammonia solution and extracted with 50:50 hexane:EtOAc 3x. The combined organic layers were sequentially washed with water 3x and 5% LiCl solution, then concentrated. The resulting residue purified by silica gel flash chromatography (70% CH_2Cl_2 in hexane) to afford the title compound as a white solid (1.67 g, 9.15 mmol, 48%). ^{1}H NMR (400 MHz, DMSO) δ 7.54 (s, 1H), 6.46 (s, 1H), 6.40 (s, 2H), 3.79 (s, 3H). Data consistent with that found in the literature. [[22]]

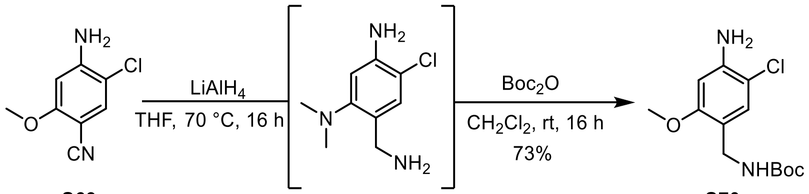

S69c **S70c**

Aniline S70c. To a suspension of $LiAlH_4$ (624 mg, 16.43 mmol, 3 eq) in dry THF (30mL) was added **S69c** (1 g, 5.48 mmol, 1.0 eq) in THF (30mL). The suspension was refluxed for 16 hours. The reaction mixture was cooled, diluted in Et_2O (30 mL), then water (625 µL), 2.5M NaOH (625 µL), and water (2 mL) was added dropwise. The suspension was stirred for 15 minutes, and then anhydrous $MgSO_4$ was added. The solution was filtered, filtrate concentrated to afford the crude amine. The residue was dissolved in CH_2Cl_2 (30 mL) and Boc_2O (1.11 mg, 5.09 mmol, 0.93 eq) was added. The reaction was stirred overnight. The solution was concentrated, then purified by silica gel flash chromatography (3% EtOAc in CH_2Cl_2), and the pure fractions were combined to afford the title compound as a white solid

(1.14 g, 3.98 mmol, 73%). ^{1}H NMR (400 MHz, $CDCl_3$) δ 7.11 (s, 1H), 6.27 (s, 1H), 4.89 (s, 1H), 4.14 (d, J = 5.44 Hz, 2H), 4.02 (s, 2H), 3.76 (s, 3H), 1.44 (s, 9H). ^{13}C NMR (126 MHz, $CDCl_3$) δ 157.09, 155.80, 142.98, 129.93, 118.26, 110.18, 98.64, 79.22, 55.51, 39.66, 28.46. HRMS-ESI (m/z) Calculated for $C_{13}H_{19}ClN_2O_3Na$ $[M+Na]^+$, 309.0975; found 309.0978.

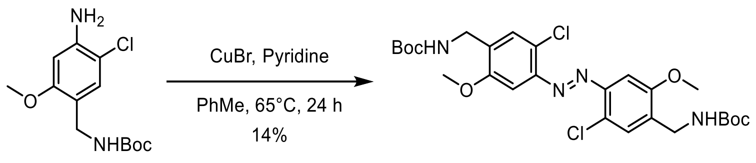

S70c **Cl_2,*m*-(OMe)$_2$**

Cl_2,*m*-OMe$_2$. Aniline **S70c** (100 mg, 348 µmol) was dissolved in toluene (4 mL). Pyridine (56 µL, 0.7 mmol, 2 eq) and CuBr (50 mg, 348 µmol, 1 eq) was added and the reaction was stirred under atmospheric air at 65 °C for 24 hours. The reaction was concentrated, then purified by silica gel flash chromatography (3% MeCN in $CHCl_3$). The fractions containing product were concentrated and further purified by silica gel flash chromatography (2% MeOH in CH_2Cl_2) to afford the title compound as an orange solid (14 mg, 24.6 µmol, 14%). *(E)*-**Cl_2,*m*-OMe$_2$:** ^{1}H NMR (400 MHz, $CDCl_3$) δ 7.45 (s, 2H), 7.33 (s, 2H), 5.02 (s, 2H), 4.34 (d, J = 6.3 Hz, 4H), 3.90 (s, 6H), 1.47 (s, 18H).^{13}C NMR (126 MHz, $CDCl_3$) δ 156.59, 155.96, 148.24, 132.80, 130.39, 128.50, 99.39, 79.87, 55.89, 40.05, 28.53. HRMS-ESI (m/z) Calculated for $C_{26}H_{35}Cl_2N_4O_6$ $[M+H]^+$, 569.1928; found 569.1928.

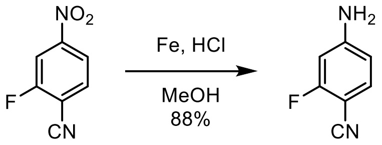

S65 **S66**

Aniline S66. To a solution of nitro compound **S65** (2 g, 12 mmol) in MeOH (20 mL) was added Fe powder (2 g, 36 mmol, 3 eq). Then, concentrated HCl (8 mL) was added dropwise, and the reaction was stirred for 2 hours at room temperature. The reaction was filtered over celite and washed with MeOH. The filtrate was diluted with H_2O, then extracted with EtOAc. The combined organic layer was washed with saturated bicarbonate solution. The organic layer was dried with magnesium sulfate, filtered, then concentrated to afford the title compound as an off-white solid (1.45 g, 10.7 mmol, 88%). ^{1}H NMR (400 MHz, $CDCl_3$) δ 7.34 (dd, J = 8.4, 7.2 Hz, 1H), 6.49 – 6.32 (m, 2H), 4.60 – 3.50 (br s, 2H). Data consistent with that given in the literature. [23]

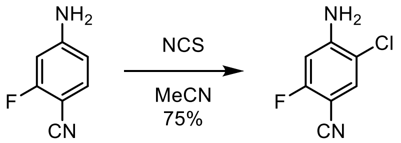

S66 **S67**

Nitrile S67. Aniline **S66** (1.25 g, 9.18 mmol) was dissolved in MeCN (63 mL). NCS (1.23 g, 9.18 mmol, 1 eq) was added and the reaction was stirred at 65 °C for 4 hours. The reaction was concentrated, then purified by silica gel flash chromatography (7:2:1 Hexane:CH_2Cl_2:EtOAc) to afford the title compound as a white solid (1.18 g, 6.92 mmol, 75%). ^{1}H NMR (400 MHz, $CDCl_3$) δ 7.46 (d, J = 6.4 Hz, 1H), 6.51 (d, J = 10.3 Hz, 1H), 4.71 (s, 2H). ^{13}C NMR (101 MHz, $CDCl_3$) δ 163.20 (d, J = 255.5 Hz), 148.84 (d, J = 12.0 Hz), 133.38 (d, J = 2.6 Hz), 117.31 – 109.78 (m), 101.71 (d, J = 24.5 Hz), 89.80 (d, J = 17.6 Hz). HRMS-EI (m/z) Calculated for $C_7H_3N_2FCl$ $[M-H]^-$, 168.9974; found 168.9966

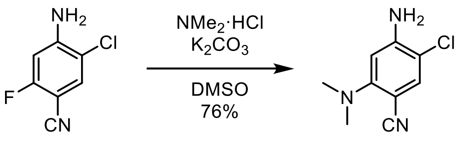

S67 **S68**

Aniline S68. Aryl fluoride **S67** (130 mg, 0.76 mmol) was dissolved in DMSO (4 mL). Then, dimethylamine hydrochloride (316 mg, 2.29 mmol, 3 eq) and potassium carbonate (615 mg, 7.6 mmol) was added. The reaction was stirred at 150 °C for 16 hours. The mixture was poured onto ice, then extracted with Et_2O 3x. The combined organic layer was washed with water, then concentrated. The residue was purified by silica gel flash chromatography (70% CH_2Cl_2 in hexane to 100% CH_2Cl_2) to afford the title compound as a white solid (114 mg, 0.58 mmol, 76%).^{1}H NMR (400 MHz, $CDCl_3$) δ 7.33 (s, 1H), 6.16 (s, 1H), 4.59 – 4.37 (m, 2H), 2.95 (s, 6H). ^{13}C NMR (101 MHz, $CDCl_3$) δ 155.64, 147.48, 135.11, 119.68, 109.66, 102.12, 91.48, 91.45, 43.08. HRMS-EI (m/z) Calculated for $C_9H_{11}N_3Cl$ $[M+H]^+$, 196.0636; found 196.0636

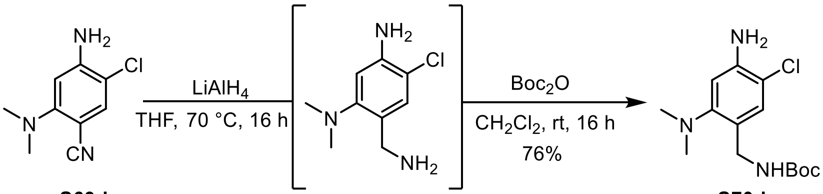

S69d **S70d**

Aniline S70d. To a suspension of $LiAlH_4$ (66 mg, 1.75 mmol, 3 eq) in dry THF (4 mL) was added **S69d** (114 mg, 0.58 mmol, 1.0 eq) in THF (4 mL). The suspension was refluxed for 16 hours. The reaction mixture was cooled, diluted in Et_2O (5 mL), then water (70 µL), 2.5M NaOH (70 µL), and water (0.2 mL) was added dropwise. The suspension was stirred for 15 minutes, and then anhydrous $MgSO_4$ was added. The solution was filtered, filtrate concentrated to afford the crude amine and imine intermediate. The residue was dissolved in CH_2Cl_2 (5 mL) and Boc_2O (64 mg, 0.29 mmol, 0.5 eq) was added. The reaction was stirred overnight. The solution was concentrated, then purified by silica gel flash chromatography (CH_2Cl_2 to 10% EtOAc in CH_2Cl_2), and the pure fractions were combined to afford the title compound as a yellow oil (56 mg, 0.19 mmol, 32%). ^{1}H NMR (400 MHz, $CDCl_3$) δ 7.12 (s, 1H), 6.49 (s, 1H), 5.10 (s, 1H), 4.26 (d, *J* = 5.8 Hz, 2H), 3.99 (s, 2H), 2.61 (s, 6H), 1.45 (s, 9H). ^{13}C NMR (101 MHz, $CDCl_3$) δ 156.18, 152.21, 142.50, 129.72, 124.21, 113.78, 107.20, 79.39, 44.90, 40.59, 28.56. HRMS-EI (m/z) Calculated for $C_{14}H_{23}O_2N_3Cl$ $[M+H]^+$, 300.1473; found 300.1472

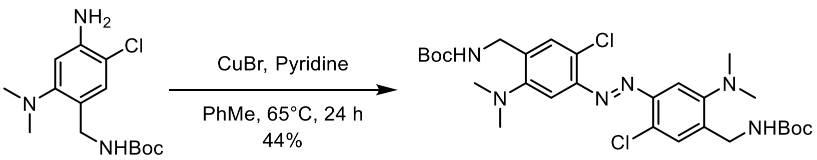

S70d **Cl$_2$,*m*-(NMe$_2$)$_2$.**

Cl$_2$,*m*-(NMe$_2$)$_2$. Aniline **S70d** (56 mg, 187 µmol) was dissolved in toluene (3.5 mL). Pyridine (45 µL, 0.56 mmol, 3 eq) and CuBr (27 mg, 187 µmol, 1 eq) was added and the reaction was stirred under atmospheric air at 65 °C for 24 hours. The reaction was concentrated, then purified by silica gel flash chromatography (2.5% Acetone in CH_2Cl_2), and the pure fractions were combined to afford the title compound as an orange solid (24.5 mg, 41 µmol, 44%). *(E)*-**Cl$_2$,*m*-(NMe$_2$)$_2$:** ^{1}H NMR (400 MHz, $CDCl_3$) δ 7.52 (s, 1H), 7.48 (s, 1H), 5.16 (s, 1H), 4.46 (d, *J* = 5.9 Hz, 2H), 2.72 (s, 6H), 1.49 (s, 9H). ^{13}C NMR (151 MHz, $CDCl_3$) δ 156.30, 151.74, 147.98, 138.37, 130.68, 130.22, 108.58, 79.96, 44.77, 40.86, 28.54. HRMS-EI (m/z) Calculated for $C_{28}H_{41}O_4N_6Cl_2$ $[M+H]^+$, 595.2561; found 595.2559

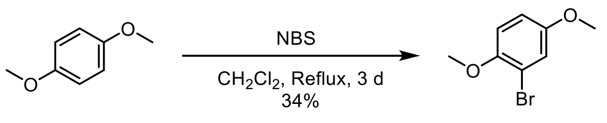

Bromide S71. Dimethylhydroquinone **S71** (8.28 g, 60 mmol) was dissolved in dry CH_2Cl_2 (50 mL). N-bromosuccinimide (11 g, 1.02 eq, 61.2 mmol) was added and the reaction was stirred at 35°C for 3 days. The reaction was concentrated, then purified by silica gel flash chromatography (20% CH_2Cl_2 in hexane). The pure fractions were combined to afford the title compound as a white solid (4.4 g, 20.2 mmol, 34%). ^{1}H NMR (400 MHz, $CDCl_3$) δ 7.13 (dd, *J* = 2.7, 0.6 Hz, 1H), 6.85 – 6.80 (m, 2H), 3.85 (s, 3H), 3.76 (s, 3H). Data matches that give in the literature. [[24]]

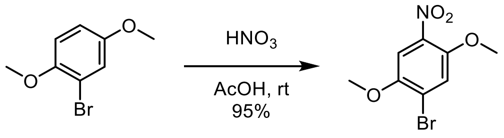

Nitro compound S73. S72 (4.4 g, 20.2 mmol) was suspended in AcOH (10 mL), then nitric acid (1.8 mL) was added. The solution immediately turned yellow and was stirred for a further 15 minutes at room temperature. H_2O (40 mL) was added and the suspension was stirred for 10 minutes. The precipitated solid was collected by filtration, then dried to afford the title compound as a yellow solid (5.03 g, 9.2 mmol, 95%). ^{1}H NMR (400 MHz, $CDCl_3$) δ 7.47 (s, 1H), 7.35 (s, 1H), 3.94 (s, 3H), 3.91 (s, 3H). Data consistent with that given in the literature[[25]]

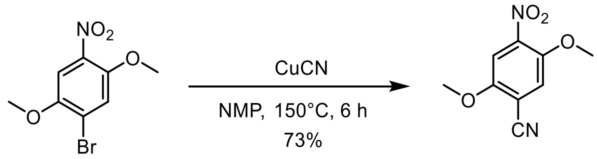

Nitrile S74. Bromide **S73** (2.5 g, 9.54 mmol) and CuCN (1.71 g, 19.1 mmol, 2 eq) were added to a flask under N_2. NMP (20 mL) was added and the reaction was stirred for 6 hours at 150 °C under inert atmosphere. The reaction was cooled, poured over ice water/15% ammonia solution and extracted with 50:50 hexane:EtOAc 3x. The combined organic layers were sequentially washed with water and 5% LiCl solution, then concentrated. The resulting residue purified by silica gel flash chromatography (60% CH_2Cl_2 in hexane) to afford the title compound as a yellow solid (1.44 g, 7.70 mmol, 73%). ^{1}H NMR (400 MHz, $CDCl_3$) δ 7.41 (s, 1H), 7.29 (s, 1H), 3.96 (s, 3H), 3.94 (s, 3H). ^{13}C NMR (126 MHz, $CDCl_3$) δ ^{13}C NMR (126 MHz, $CDCl_3$) δ 154.85, 146.49, 142.72, 118.98, 114.59, 108.75, 106.51, 57.51, 57.12. HRMS-EI (m/z) Calculated for $C_9H_8O_4N_2Na$ $[M+Na]^+$, 231.0376; found 231.0377.

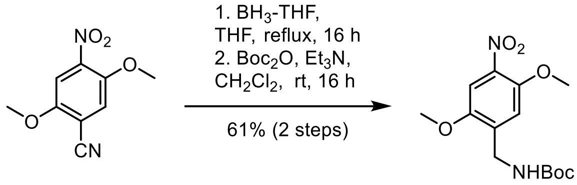

Aniline S75. Nitrile **S74** (1.4 g, 6.73 mmol) was dissolved in THF (10 mL). Borane-THF (1M, 27.6 mL, 27.6 mmol, 6 eq) was added at 0 °C under N_2 atmosphere, and the reaction was refluxed for 16 hours. The solution was quenched with MeOH (5 mL) and stirred for 1 hour at 50 °C. The solution was concentrated in vacuo, then redissolved in CH_2Cl_2. This was washed with 100 mL water, then the organic layer was concentrated. The residue was suspended in CH_2Cl_2 (15 mL) and then Boc_2O (1.47g,

6.73 mmol, 1 eq) in CH_2Cl_2 (15 mL) was added. Then Et_3N (1 mL) was added and the reaction was stirred overnight at room temperature. The suspension was concentrated, the purified by silica gel flash chromatography (80% CH_2Cl_2 to 100% CH_2Cl_2) to afford the title compound as a yellow oil which eventually crystallised to a yellow solid. (1.29 g, 4.13 mmol, 61% over two steps). ^{1}H NMR (400 MHz, $CDCl_3$) δ 7.40 (s, 1H), 7.07 (s, 1H), 5.05 (s, 1H), 4.31 (d, *J* = 5.9 Hz, 2H), 3.93 (s, 3H), 3.86 (s, 3H), 1.45 (s, 9H). ^{13}C NMR (126 MHz, $CDCl_3$) δ 155.99, 150.48, 147.89, 138.00, 134.90, 115.10, 107.61, 80.00, 57.27, 56.24, 40.18, 28.52. HRMS-EI (m/z) Calculated for $C_{14}H_{20}O_6N_2Na$ [M+Na]$^+$, 335.1214; found 335.1213

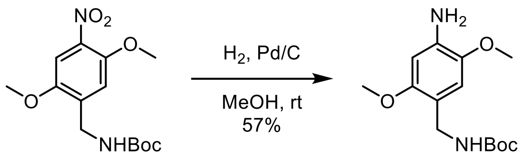

S75 S76

Aniline S76. nitro compound **S75** (1.28 g, 4.10 mmol) was dissolved in MeOH (40 mL). The reaction vessel was evacuated and filled with nitrogen 3 x. Palladium 10% on carbon (800 mg) was added and the reaction was stirred under a hydrogen atmosphere for 16 hours. The reaction mixture was filtered over celite, the filtrate was concentrated, then purified by silica gel flash chromatography (5% acetone in CH_2Cl_2) to afford the title compound as light brown solid (665 mg, 2.36 mmol, 57%). ^{1}H NMR (400 MHz, $CDCl_3$) δ 6.76 (s, 1H), 6.39 (s, 1H), 4.95 (s, 1H), 4.20 (d, *J* = 5.8 Hz, 2H), 3.80 (s, 3H), 3.75 (s, 3H), 1.44 (s, 9H). ^{13}C NMR (126 MHz, $CDCl_3$) δ 156.06, 152.24, 141.02, 136.28, 116.27, 113.30, 99.49, 79.14, 56.42, 55.99, 40.41, 28.61. HRMS-EI (m/z) Calculated for $C_{14}H_{22}O_4N_2Na$ [M+Na]$^+$, 305.1472; found 305.1472

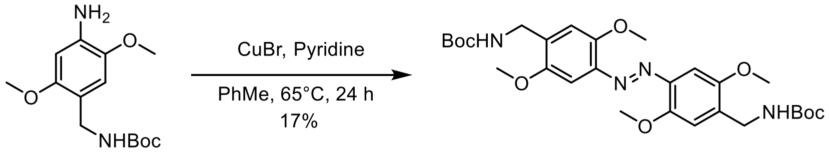

S76 OMe$_2$,*m*-OMe$_2$

OMe₂,*m*-OMe. Aniline **S76** (214 mg, 758 µmol) was dissolved in toluene (10 mL). Pyridine (122µL, 1.52 mmol, 2 eq) and CuBr (109 mg, 758 µmol, 1 eq) was added and the reaction was stirred under atmospheric air at 60 °C for 24 hours. The reaction was concentrated, then purified by silica gel flash chromatography (0.5% MeOH in $CHCl_3$), and the pure fractions were combined to afford the title compound as an orange solid (36 mg, 64 µmol, 17%). *(E)*-**OMe₂,*m*-OMe₂:** ^{1}H NMR (500 MHz, CD_2Cl_2) δ 7.19 (s, 1H), 7.05 (s, 1H), 5.13 (s, 1H), 4.31 (d, *J* = 6.2 Hz, 2H), 3.98 (s, 3H), 3.86 (s, 3H), 1.45 (s, 9H). ^{13}C NMR (126 MHz, CD_2Cl_2) δ 156.20, 152.18, 152.14, 142.18, 132.51, 114.81, 98.94, 79.51, 57.59, 56.17, 28.53. HRMS-EI (m/z) Calculated for $C_{28}H_{41}O_8N_4$ [M+H]$^+$, 561.2919; found 561.2916

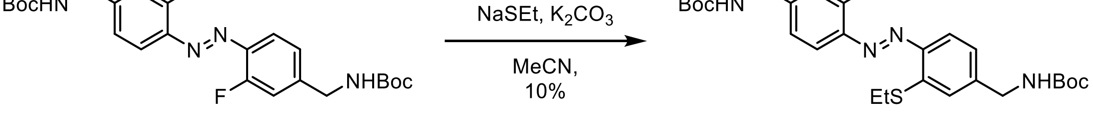

F$_2$,*m*-Y$_2$ (SEt$_2$),*m*-H

(SEt)₂,*m*-H₂. Difluoride **F₂,*m*-H₂** (55 mg, 115 µmol), Sodium ethanethiolate (80%, 49 mg, 461 µmol, 4 eq) and potassium carbonate (32 mg, 230 µmol, 2 eq) were suspended in MeCN (1.5 mL) and stirred at room temperature for 16 hours under N_2 in a sealed microwave vial. The mixture was diluted in CH_2Cl_2, then washed with water. The organic layer was concentrated, then purified by silica gel flash chromatography (0 to 0.5% Acetone in CH_2Cl_2) to afford the title compound as an orange solid (6.2

mg, 11 µmol, 10%). *(E)*-**SEt$_2$,*m*-H$_2$**: ^{1}H NMR (500 MHz, DMSO) δ 7.51 (d, *J* = 8.2 Hz, 2H), 7.40 (s, 2H), 7.15 (d, *J* = 8.4 Hz, 2H), 7.04 (s, 2H), 4.21 (d, *J* = 6.2 Hz, 4H), 3.04 (q, *J* = 7.3 Hz, 4H), 1.43 (s, 18H), 1.35 (t, *J* = 7.3 Hz, 6H). ^{13}C NMR (151 MHz, DMSO) δ 155.88, 147.36, 144.42, 139.54, 124.10, 123.56, 116.66, 78.02, 43.24, 28.22, 24.45, 13.65. HRMS-EI (m/z) Calculated for $C_{28}H_{41}O_4N_4S_2$ $[M+H]^+$, 561.2564; found 561.2560

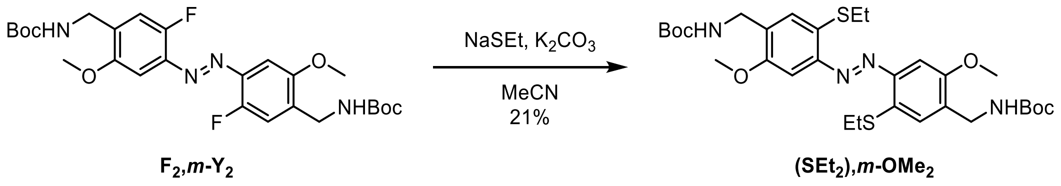

(SEt)$_2$,*m*-OMe$_2$. Difluoride **F$_2$,*m*-H$_2$** (70 mg, 130 µmol), Sodium ethanethiolate (80%, 55 mg, 522 µmol, 4 eq) and potassium carbonate (36 mg, 261 µmol, 2 eq) were suspended in MeCN (2 mL) and stirred at room temperature for 16 hours under N_2 in a sealed microwave vial. The mixture was diluted in CH_2Cl_2, then washed with water. The organic layer was concentrated, then purified by silica gel flash chromatography (5% EtOAc in CH_2Cl_2) to afford the title compound as an orange solid (21 mg, 27 µmol, 21%). ^{1}H NMR (400 MHz, $CDCl_3$) δ 7.32 (m, 4H), 5.04 (s, 2H), 4.33 (d, *J* = 6.3 Hz, 4H), 3.90 (s, 6H), 3.00 (q, *J* = 7.4 Hz, 4H), 1.46 (s, 18H), 1.36 (t, *J* = 7.4 Hz, 6H). ^{13}C NMR (126 MHz, $CDCl_3$) δ 156.17, 156.01, 150.36, 131.42, 131.29, 128.92, 99.31, 79.62, 55.71, 40.45, 28.57, 27.32, 14.26. HRMS-EI (m/z) Calculated for $C_{30}H_{45}O_6N_4S_2$ $[M+H]^+$, 625.2775; found 625.2774

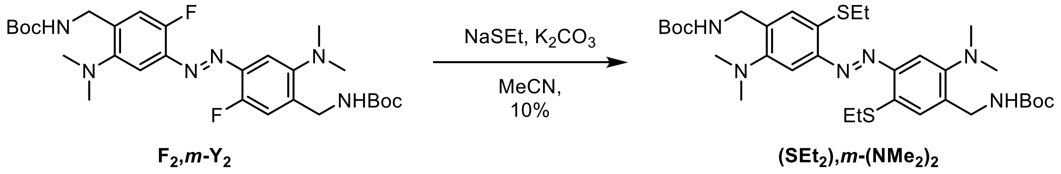

(SEt$_2$),*m*-(NMe$_2$)$_2$. Difluoride **F$_2$,*m*-H$_2$** (41.5 mg, 73 µmol), Sodium ethanethiolate (80%, 31 mg, 295 µmol, 4 eq) and potassium carbonate (20.4 mg, 148 µmol, 2 eq) were suspended in MeCN (2 mL) and stirred at room temperature for 16 hours under N_2 in a sealed microwave vial. The mixture was diluted in CH_2Cl_2, then washed with water. The organic layer was concentrated, then purified by silica gel flash chromatography (5 to 8% EtOAc in CH_2Cl_2) to afford the title compound as an orange solid (6.7 mg, 10 µmol, 14%). ^{1}H NMR (500 MHz, $CDCl_3$) δ 7.52 (s, 2H), 7.33 (s, 2H), 5.24 (s, 2H), 4.45 (d, *J* = 6.4 Hz, 2H), 3.01 (q, *J* = 7.4 Hz, 4H), 2.71 (s, 12H), 1.38 (t, *J* = 7.4 Hz, 6H). ^{13}C NMR (126 MHz, $CDCl_3$) δ 156.24, 150.37, 149.58, 136.87, 133.57, 127.68, 108.69, 79.53, 44.78, 41.12, 28.45, 26.69, 14.05. HRMS-EI (m/z) Calculated for $C_{32}H_{51}O_4N_6S_2$ $[M+H]^+$, 647.3408; found 647.3405

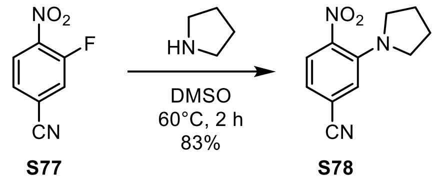

Nitrile S78. To a solution of 3-fluoro-4-nitrobenzonitrile **S77** (3 g, 18 mmol) in DMSO (20 mL) was added pyrrolidine (3 mL, 36 mmol, 2 eq); the solution went from colourless to yellow. The yellow solution was stirred at 60 °C for 2 hours. Ice and brine were added to the mixture, and the solid precipitate was extracted with CH_2Cl_2 3x. The organic layer was washed with brine. The solution was concentrated and recrystallized from boiling EtOH to afford the title compound as yellow needle-like crystals (3.26 g, 15 mmol, 83%). ^{1}H NMR (400 MHz, $CDCl_3$) δ 7.73 (d, J = 8.4 Hz, 1H), 7.16 (d, J = 1.6 Hz, 1H), 6.92 (dd, Jz = 8.4, 1.6 Hz, 1H), 3.29 – 3.14 (m, 4H), 2.10 – 1.95 (m, 4H). ^{13}C NMR (101 MHz, $CDCl_3$) δ 141.99, 138.81, 127.47, 120.20, 117.96, 117.65, 116.36, 50.51, 25.79. HRMS-ESI (m/z) Calculated for $C_{11}H_{11}N_3O_2$ $[M+H]^+$, 218.0924; found 218.0926.

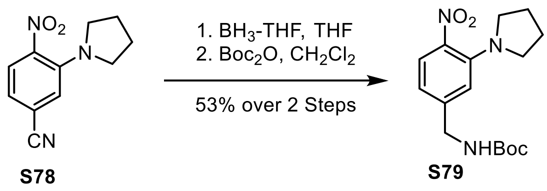

Carbamate S79. Nitrile S78 (1g, 4.6 mmol) was dissolved in THF (25 mL). Borane-THF (1M, 13.8 mL, 13.8 mmol, 3 eq) was added at 0 °C under N_2 atmosphere, and the reaction was stirred at room temperature for 16 hours. The solution was quenched with MeOH (25 mL) and stirred for 1 hour at 50 °C. The solution was concentrated in vacuo, redissolved in EtOAc, and washed with water 1x. The aqueous phase was back-extracted with CH_2Cl_2 3x. The combined organic layers were dried and concentrated in vacuo. The residue was redissolved in CH_2Cl_2 (10 mL) and Boc_2O (1.11g, 5 mmol) in CH_2Cl_2 (10mL) was added dropwise. The reaction was stirred at rt for 2 hours. The reaction was concentrated, purified by silica gel flash chromatography (50:50 Hexane:CH_2Cl_2 and 6% acetone) to afford the title compound as an orange gum (844 mg, 53% over two steps, 2.4 mmol). ^1H NMR (400 MHz, $CDCl_3$) δ 7.70 (d, J = 8.4 Hz, 1H), 6.84 – 6.74 (s, J =1.7 Hz, 1H), 6.60 (dd, J = 8.4, 1.7 Hz, 1H), 4.93 (br s, 1H), 4.28 (d, J = 6.2 Hz, 2H), 3.28 – 3.13 (m, 4H), 2.03 – 1.89 (m, 4H), 1.46 (s, 9H). ^{13}C NMR (101 MHz, $CDCl_3$) δ 156.04, 144.71, 143.10, 136.21, 127.38, 114.41, 114.34, 79.97, 50.56, 44.56, 28.50, 25.85. HRMS-ESI (m/z) Calculated for $C_{16}H_{23}N_3O_4$ $[M+H]^+$, 322.1761; found 322.1762.

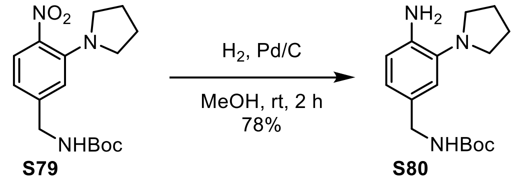

Aniline S80. Carbamate **S79** (820 mg, 2.55 mmol) was dissolved in MeOH (25 mL). The reaction vessel was evacuated and filled with nitrogen 3 x. Palladium 10% on carbon (500 mg) was added and the reaction was stirred under a hydrogen atmosphere for 2 hours. The reaction mixture was filtered over celite, the filtrate was concentrated and purified by silica gel flash chromatography (10% EtOAc in CH_2Cl_2) to afford the title compound as a white solid (580 mg, 2 mmol, 78%). ^1H NMR (400 MHz, $CDCl_3$) δ 6.90 (d, J = 2.0 Hz, 1H), 6.78 (dd, J = 7.9, 2.0 Hz, 1H), 6.67 (d, J = 7.9 Hz, 1H), 4.72 (s, 1H), 4.18 (d, J = 5.6 Hz, 2H), 3.81 (s, 2H), 3.03 (h, J = 2.8 Hz, 4H), 1.92 (h, J = 2.8 Hz, 4H), 1.46 (s, 9H). 13C NMR (101 MHz, $CDCl_3$) δ 155.99, 140.56, 137.98, 129.04, 122.81, 118.28, 115.47, 79.33, 50.90, 44.91, 28.57, 24.21. HRMS-ESI (m/z) Calculated for $C_{16}H_{25}N_3O_2$ $[M+H]^+$, 292.2020; found 292.2020.

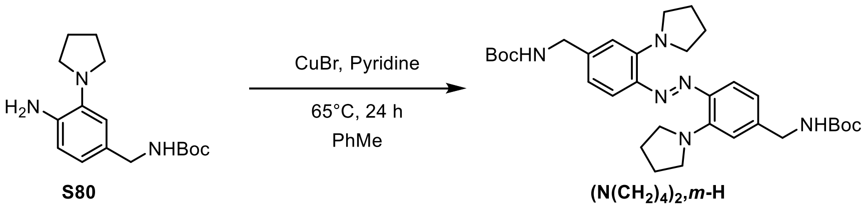

$(N(CH_2)_4)_2$,*m*-H. Aniline **S80** (138 mg, 628 µmol) was dissolved in toluene (12 mL). Pyridine (101 µL, 1.26 mmol, 2 eq) and CuBr (90 mg, 628 µmol, 1 eq) was added and the reaction was stirred under atmospheric air at 65 °C for 24 hours under protection from light. The reaction was concentrated, then purified by silica gel flash chromatography under protection from light (3% Acetone in CH_2Cl_2), and the pure fractions were combined to afford the title compound as a dark red solid (53 mg, 91 µmol, 29%). ^1H NMR (400 MHz, $CDCl_3$) δ 7.52 (s, 2H), 6.72 (s, 2H), 6.60 (s, 2H), 4.84 (s, 2H), 4.28 (s, 4H), 3.65 (s, 8H), 1.98 (s, 8H), 1.47 (s, 18H). ^{13}C NMR (101 MHz, $CDCl_3$) δ 155.94, 147.90, 146.71, 141.25, 140.31, 117.35, 115.51, 114.42, 79.45, 52.46, 44.99, 28.46, 25.96. HRMS-EI (m/z) Calculated for $C_{32}H_{47}O_4N_6$ $[M+H]^+$, 579.3653; found 579.3657

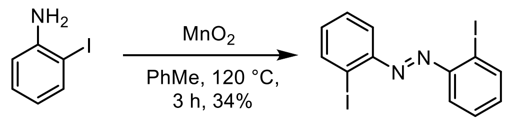

S81 **S82**

Azobenzene **S82**. Aniline **S81** (1 g, 4.57 mmol) was dissolved in toluene (100 mL). Manganese dioxide (technical grade 70%) (11.34 g, 91.31 mmol, 20 eq) was added and the suspension was refluxed for 3 hours. The mixture was filtered over celite then concentrated. The crude was dry loaded onto silica and purified by silica gel flash chromatography (100% pentane) to afford the title compound as an orange solid (336 mg, 0.77 mmol, 34%). ^{1}H NMR (400 MHz, $CDCl_3$) δ 8.04 (dd, *J* = 7.9, 1.3 Hz, 2H), 7.76 (dd, *J* = 8.0, 1.6 Hz, 2H), 7.46 (ddd, *J* = 8.2, 7.2, 1.3 Hz, 2H), 7.19 (td, *J* = 7.5, 1.6 Hz, 2H). Data consistent with that given in the literature. [[26]]

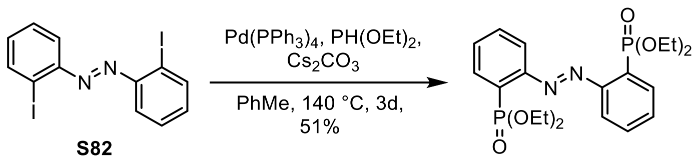

S82 **$(PO(OEt)_2)_2$,*m*-H**

$(PO(OEt)_2)_2$,*m*-H Diiodide **S82** (100 mg, 230 µmol), caesium carbonate (83 mg, 230 µmol, 1.1 eq), Tetrakis(triphenylphosphine)palladium(0) (14 mg, 11.5 µmol, 0.05 eq) were degassed with N_2 in a microwave vial for 15 minutes. Degassed toluene (1 mL) was added, then diethylphosphite (65 µL, 507 µmol, 2.2 eq) was added and the vial was sealed. The reaction was stirred at 140 °C for 3 days, then concentrated. The crude mixture was purified by silica gel flash chromatography (2% MeOH in CH_2Cl_2) to afford the product as an orange solid (53 mg, 117 µmol, 51%). ^{1}H NMR (600 MHz, $CDCl_3$) δ 8.09 (ddd, *J* = 13.9, 7.6, 1.5 Hz, 2H), 7.93 (ddd, *J* = 8.1, 5.4, 1.2 Hz, 2H), 7.66 (td, *J* = 7.6, 1.3 Hz, 2H), 7.55 (tdd, *J* = 7.5, 3.5, 1.2 Hz, 2H), 4.26 – 4.19 (m, 4H), (4.19 – 4.12 (m, 4H), 1.29 (t, *J* = 7.1 Hz, 12H). ^{13}C NMR (151 MHz, $CDCl_3$) δ 153.89 (d, *J* = 5.2 Hz), 134.35 (d, *J* = 7.4 Hz), 133.55 (d, *J* = 2.6 Hz), 130.88 (d, *J* = 14.1 Hz), 128.85 (d, *J* = 184.3 Hz), 116.67 (d, *J* = 10.4 Hz), 62.39 (d, *J* = 5.6 Hz), 16.50 (d, *J* = 6.5 Hz). HRMS-EI (m/z) Calculated for $C_{20}H_{29}N_2O_6P_2$ $[M+H]^+$, 455.1495; found 455.1497.

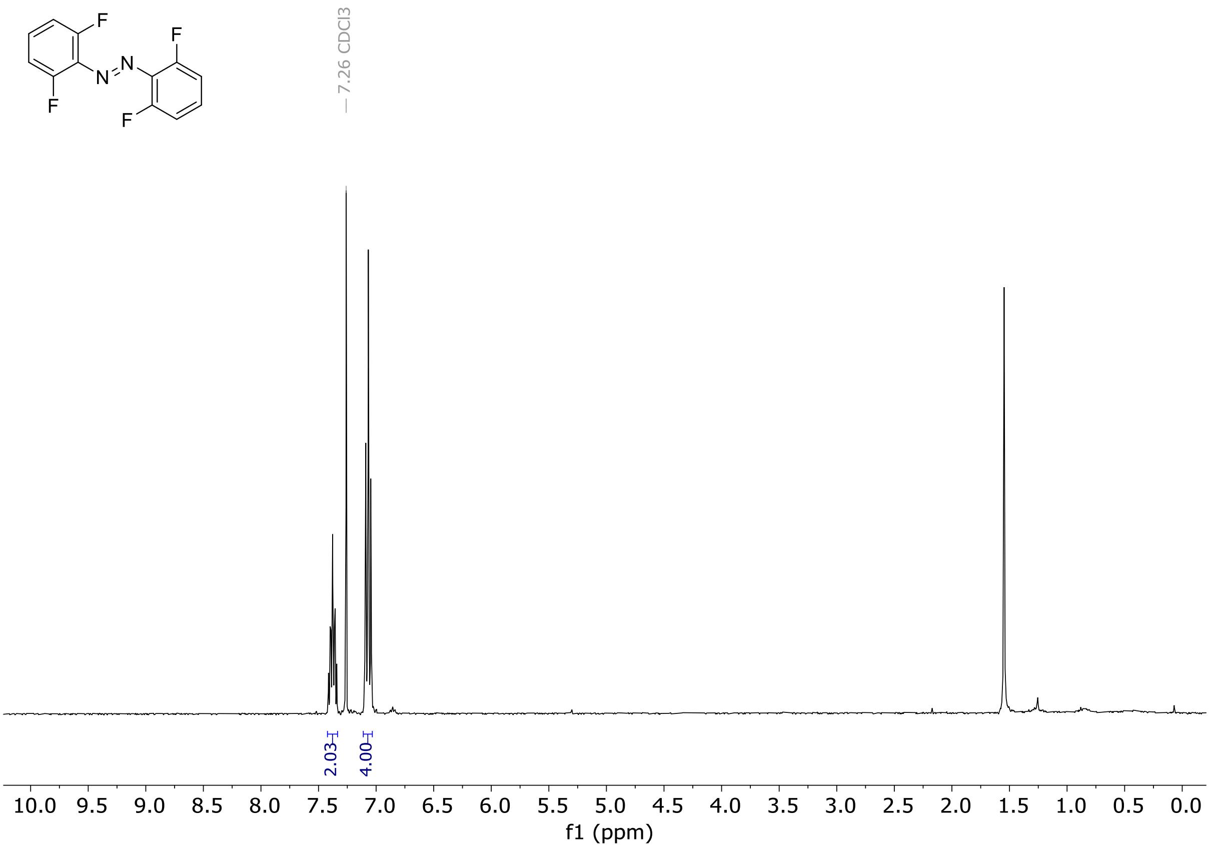

Figure S1. ^{1}H NMR spectrum of **F_4-H** (Chloroform-*d*, 298 K).

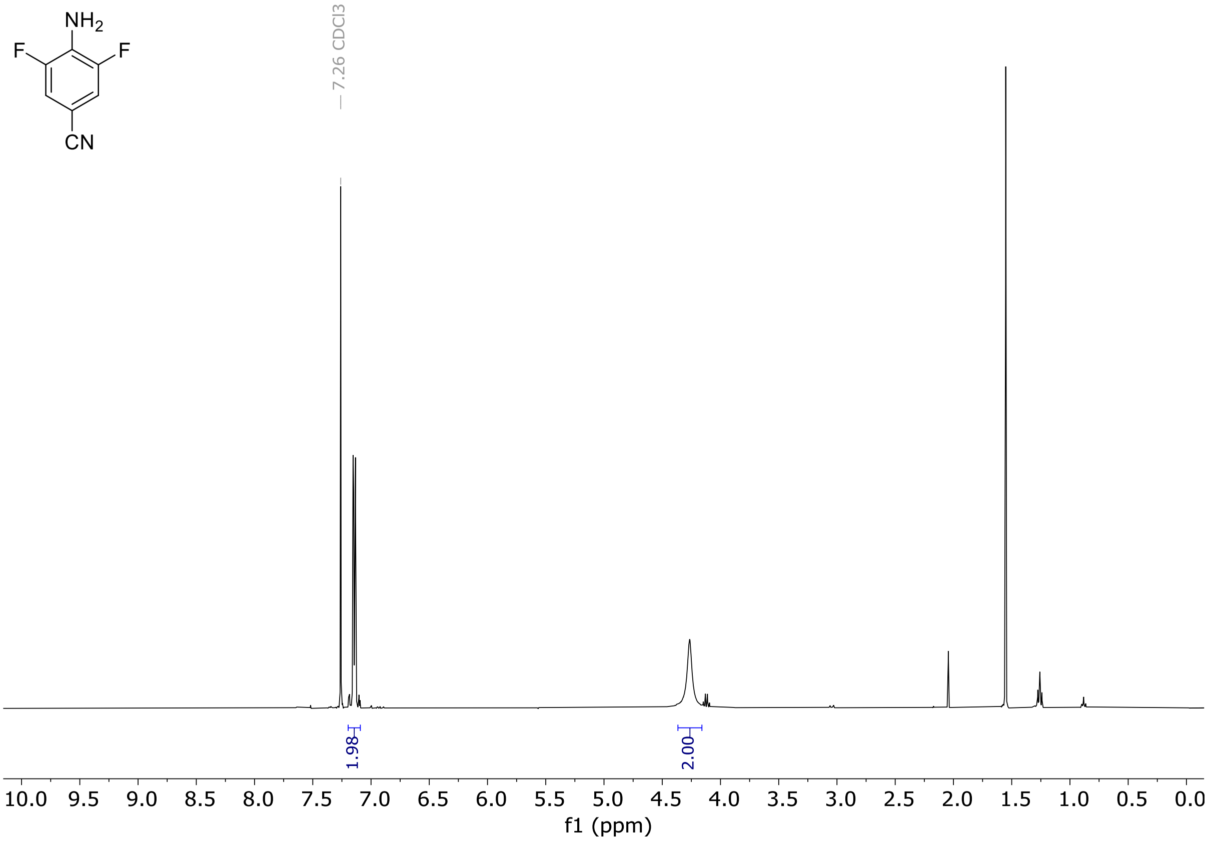

Figure S2. ^{1}H NMR spectrum of **S3** (Chloroform-*d*, 298 K).

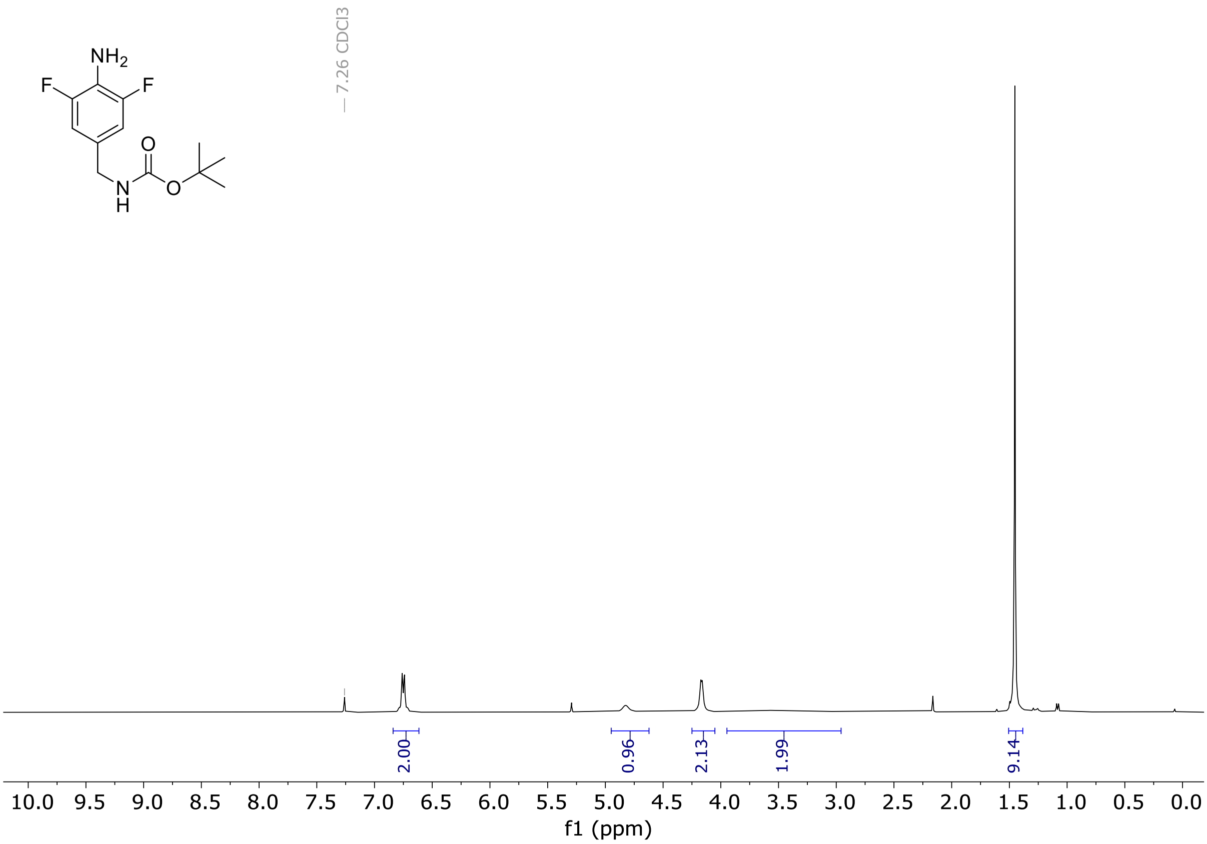

Figure S3. ^{1}H NMR spectrum of **S5** (Chloroform-*d*, 298 K).

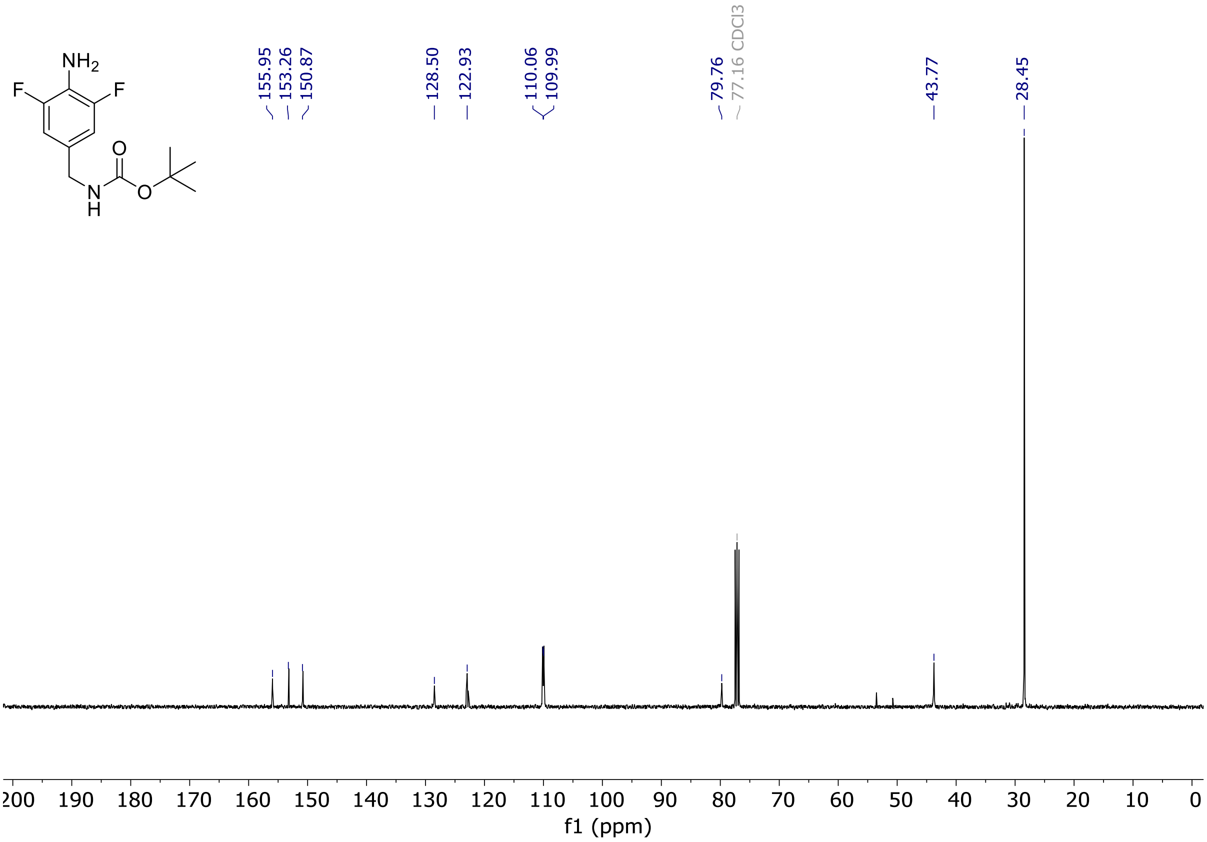

Figure S4. ^{13}C NMR spectrum of **S5**. (Chloroform-*d*, 298 K).

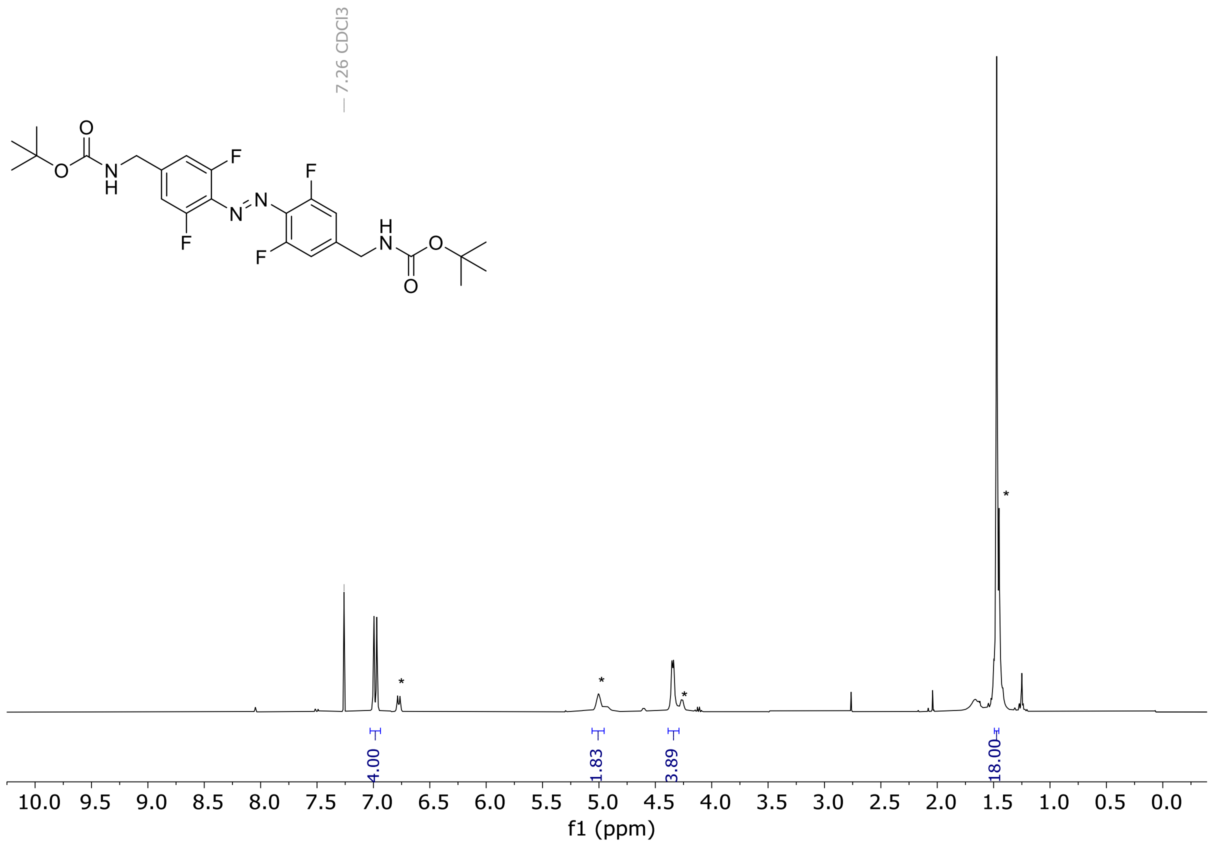

Figure S5. ^{1}H NMR spectrum of *(E)*-**F$_4$-NHBoc** (Chloroform-*d*, 298 K). ***Z*-9** signals labelled as *.

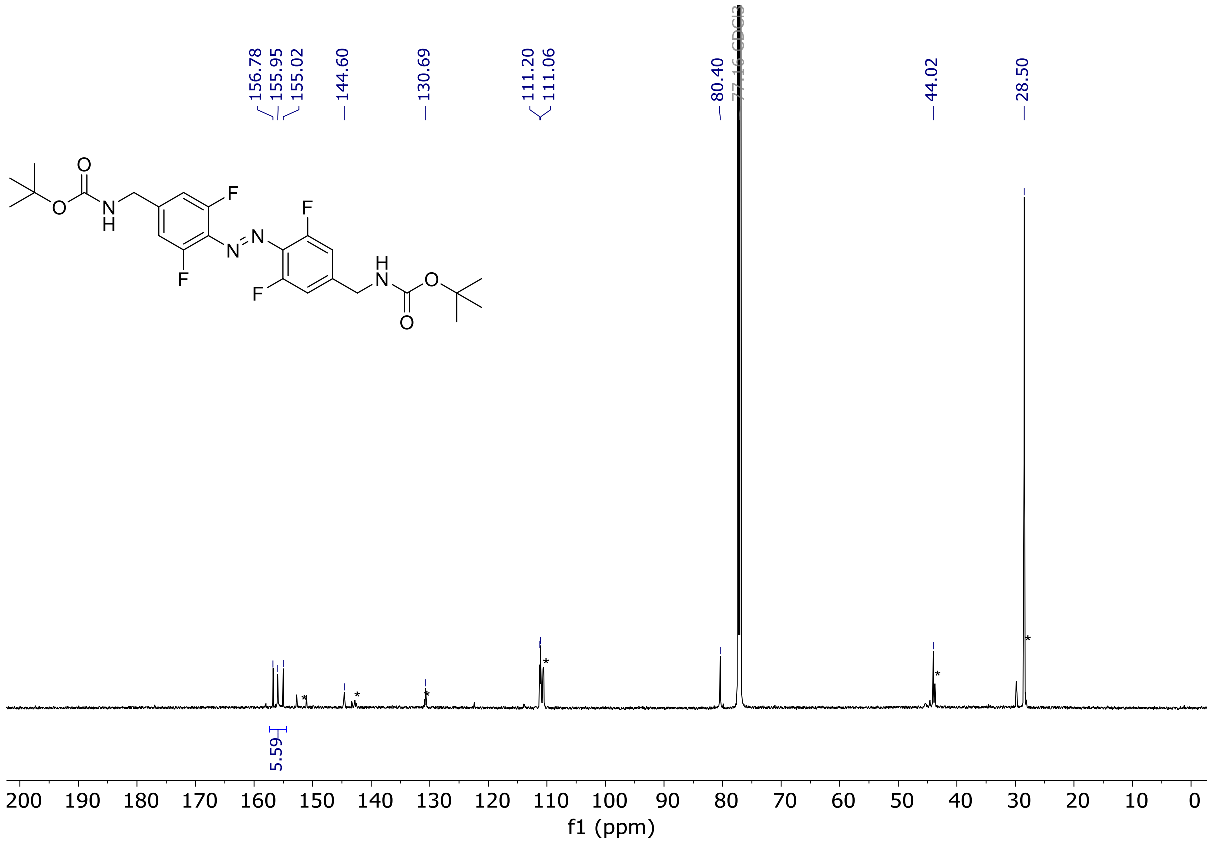

Figure S6. ^{13}C NMR spectrum of *(E)*-**F$_4$-NHBoc**. (Chloroform-*d*, 298 K). (***Z)*** signals labelled as *.

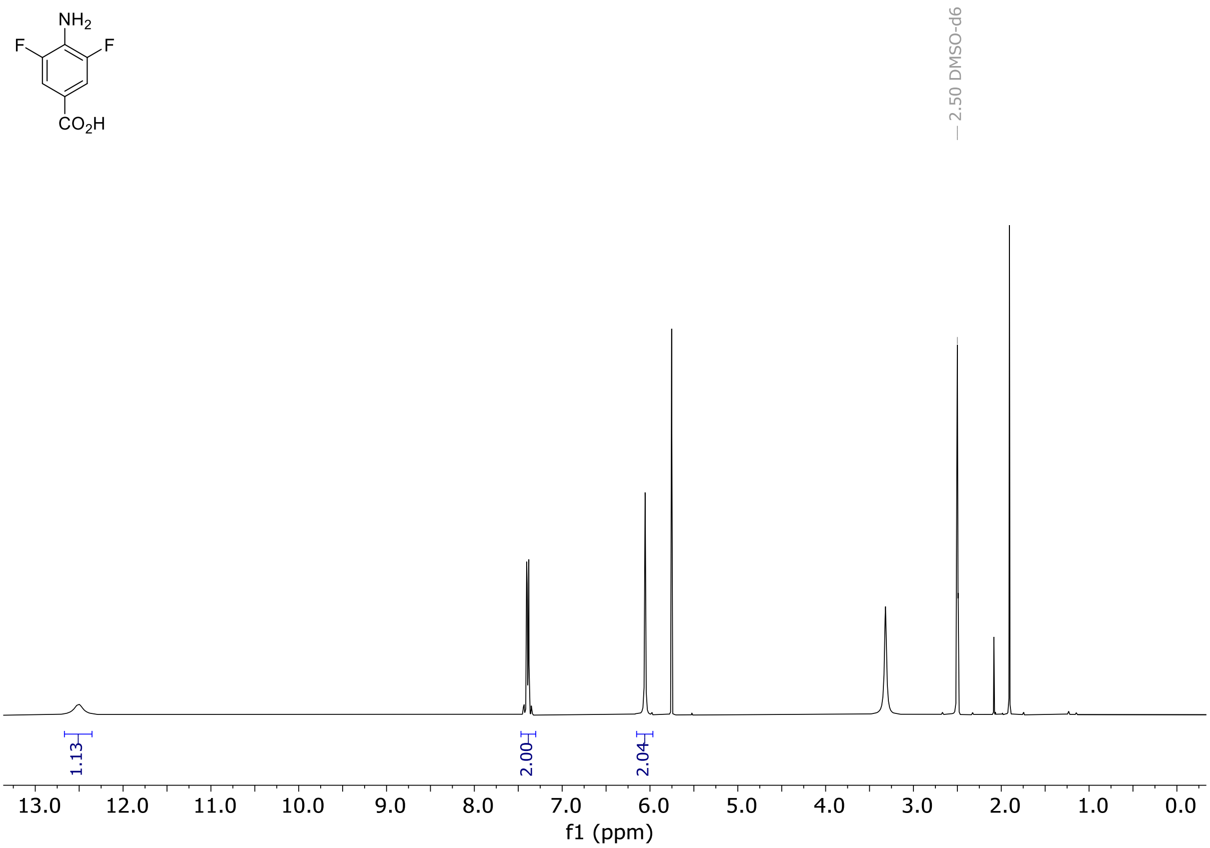

Figure S7. ^{1}H NMR spectrum of **S6** (DMSO-d_6, 298 K).

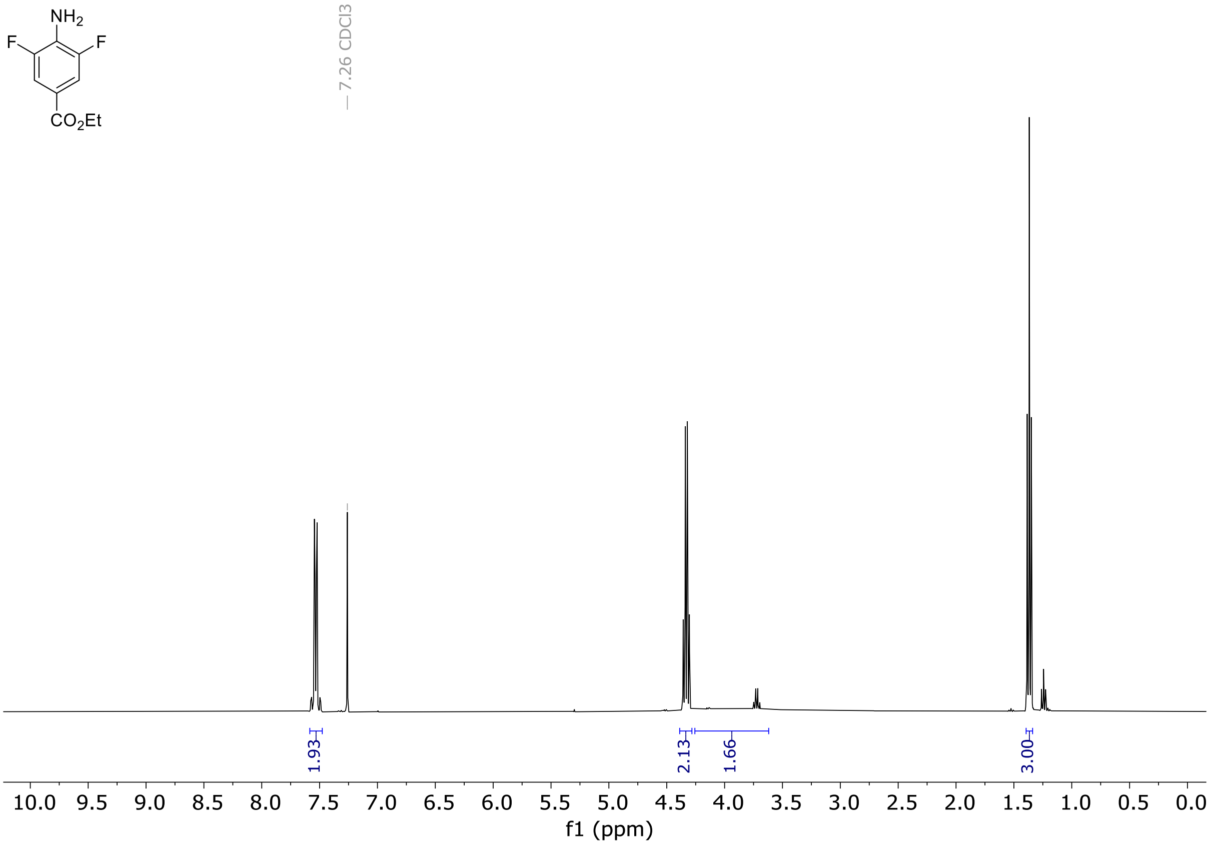

Figure S8. ^{1}H NMR spectrum of **S7** (Chloroform-d, 298 K).

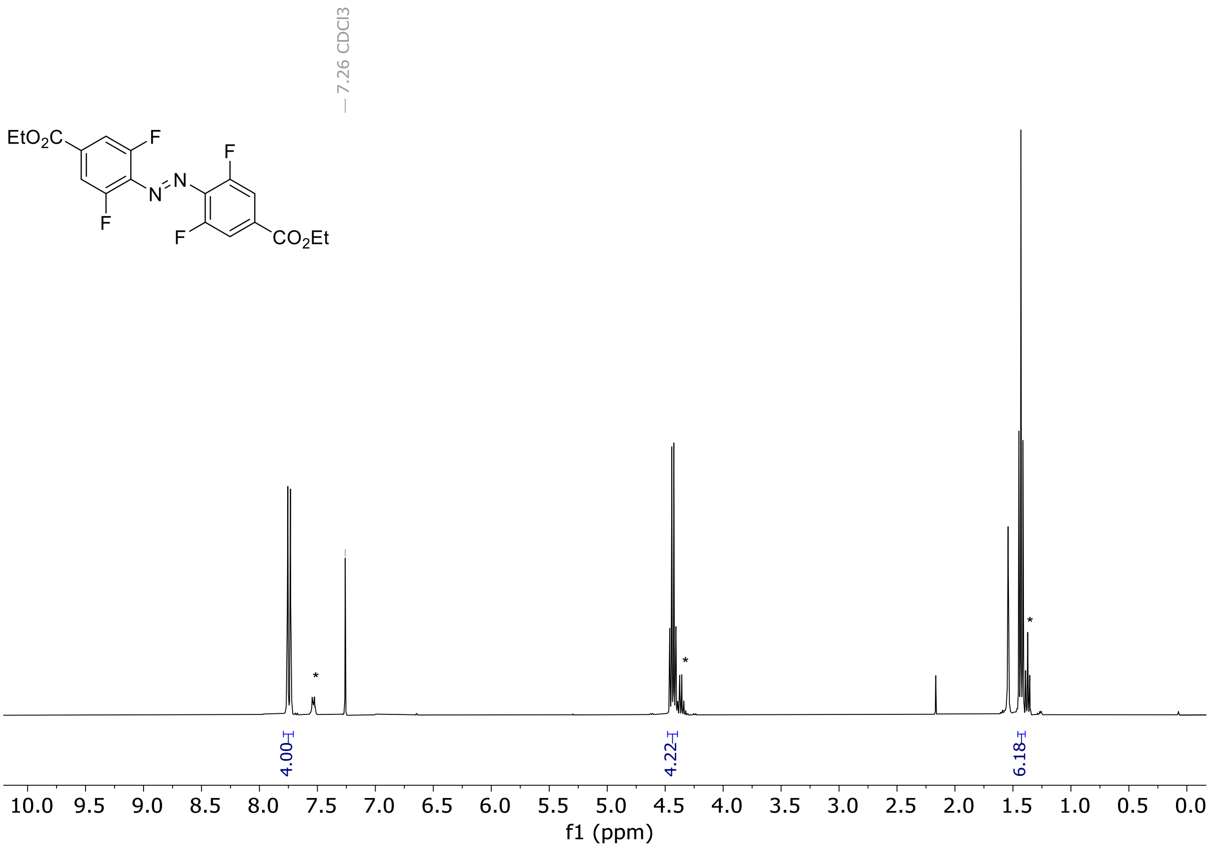

Figure S9. ^{1}H NMR spectrum of **F_4-ester** (Chloroform-*d*, 298 K). (***Z***) signals labelled as *.

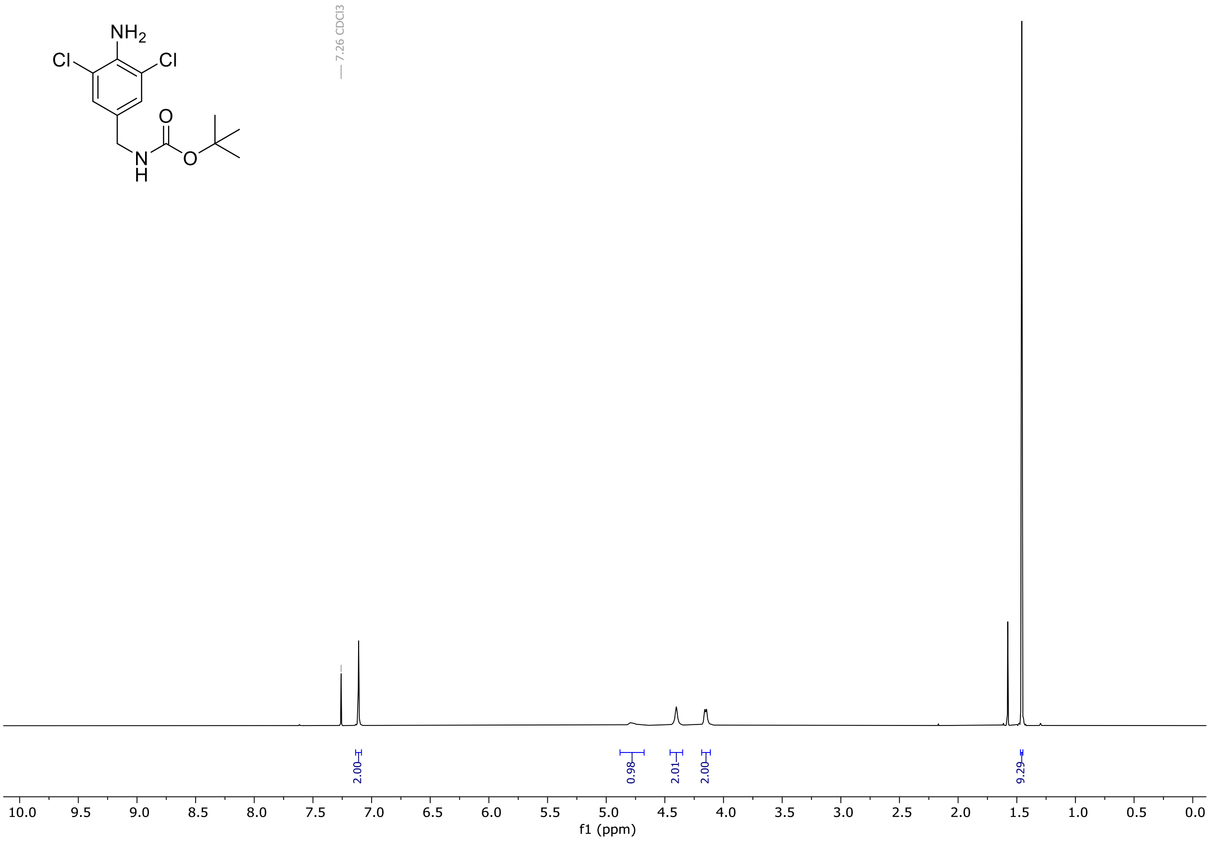

Figure S10. ^{1}H NMR Spectrum of **S10** (Chloroform-*d*, 298 K).

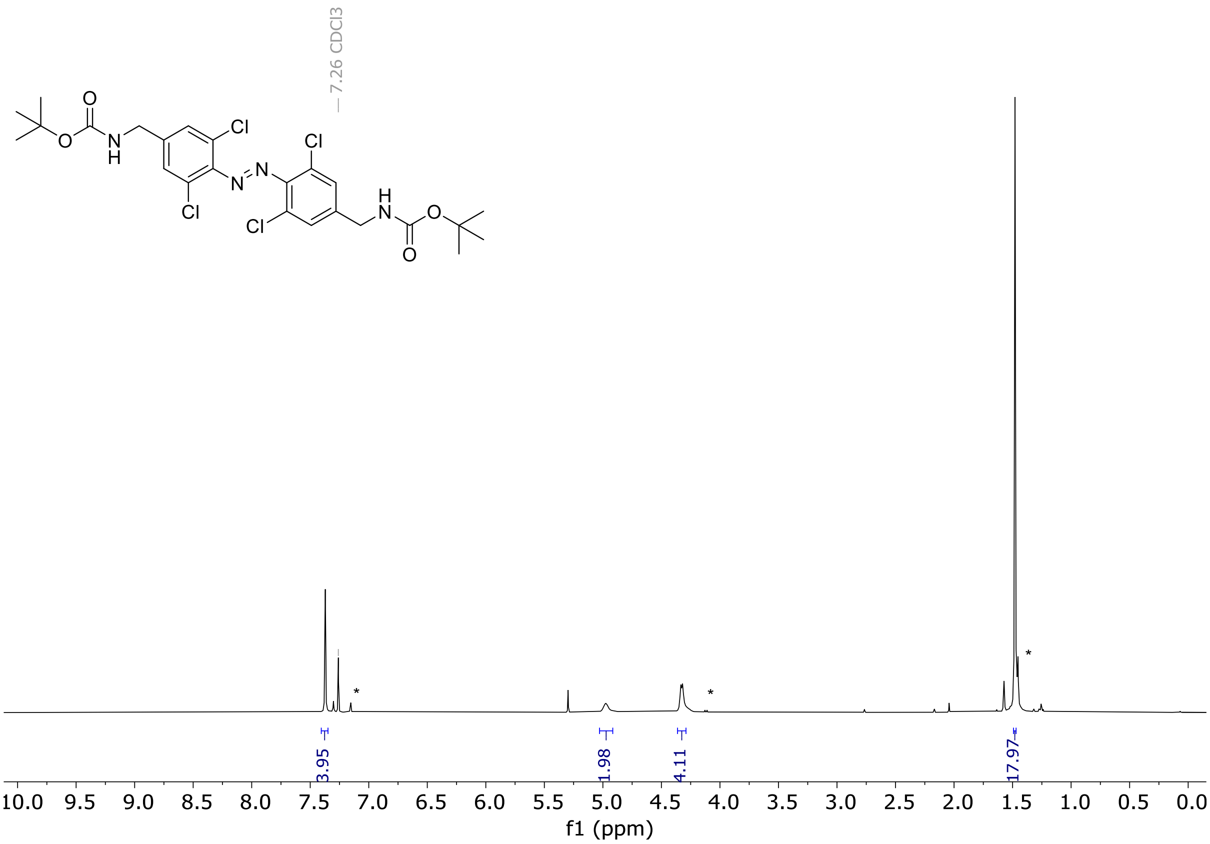

Figure S11. ^{1}H NMR Spectrum of (*E)*-**Cl_4-NHBoc** (Chloroform-*d*, 298 K). (*Z*) signals labelled as *.

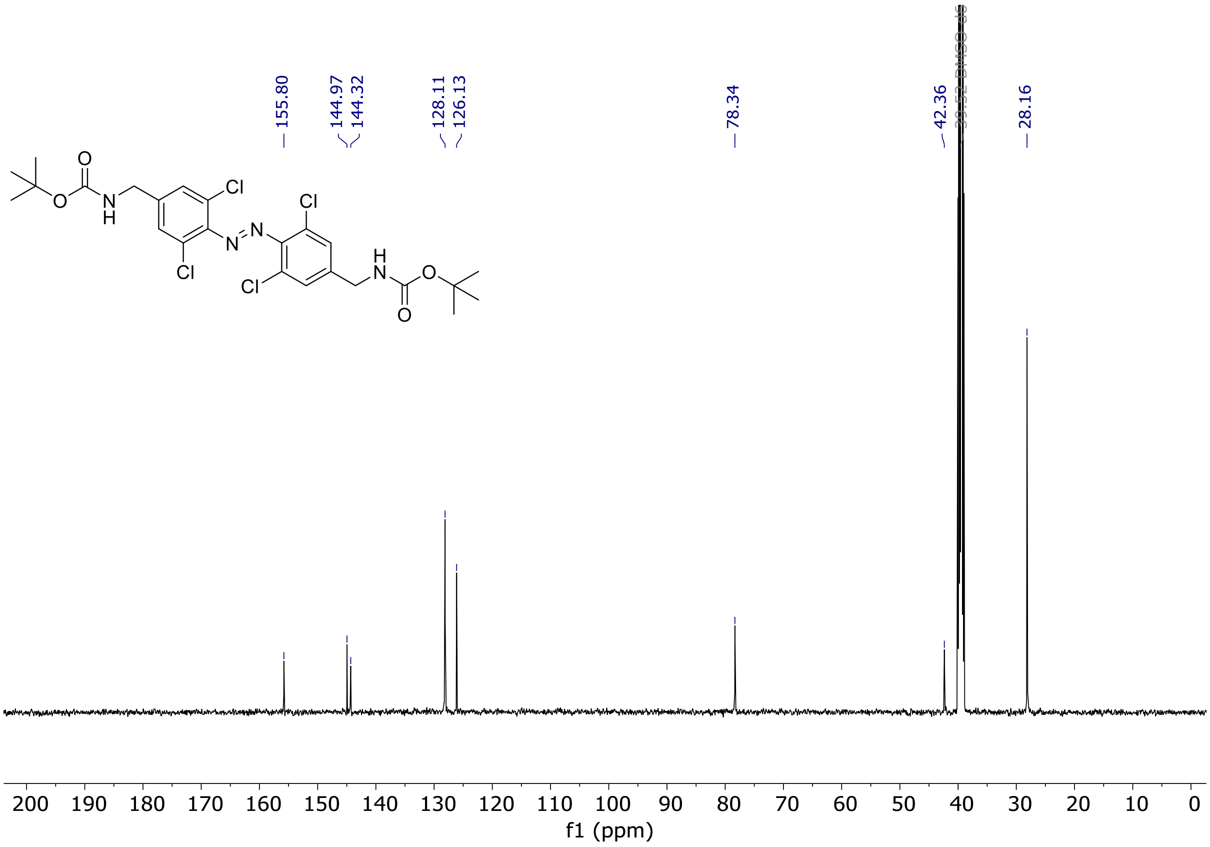

Figure S12. ^{13}C NMR Spectrum of (*E)*-**Cl_4-NHBoc** (Chloroform-*d*, 298 K).

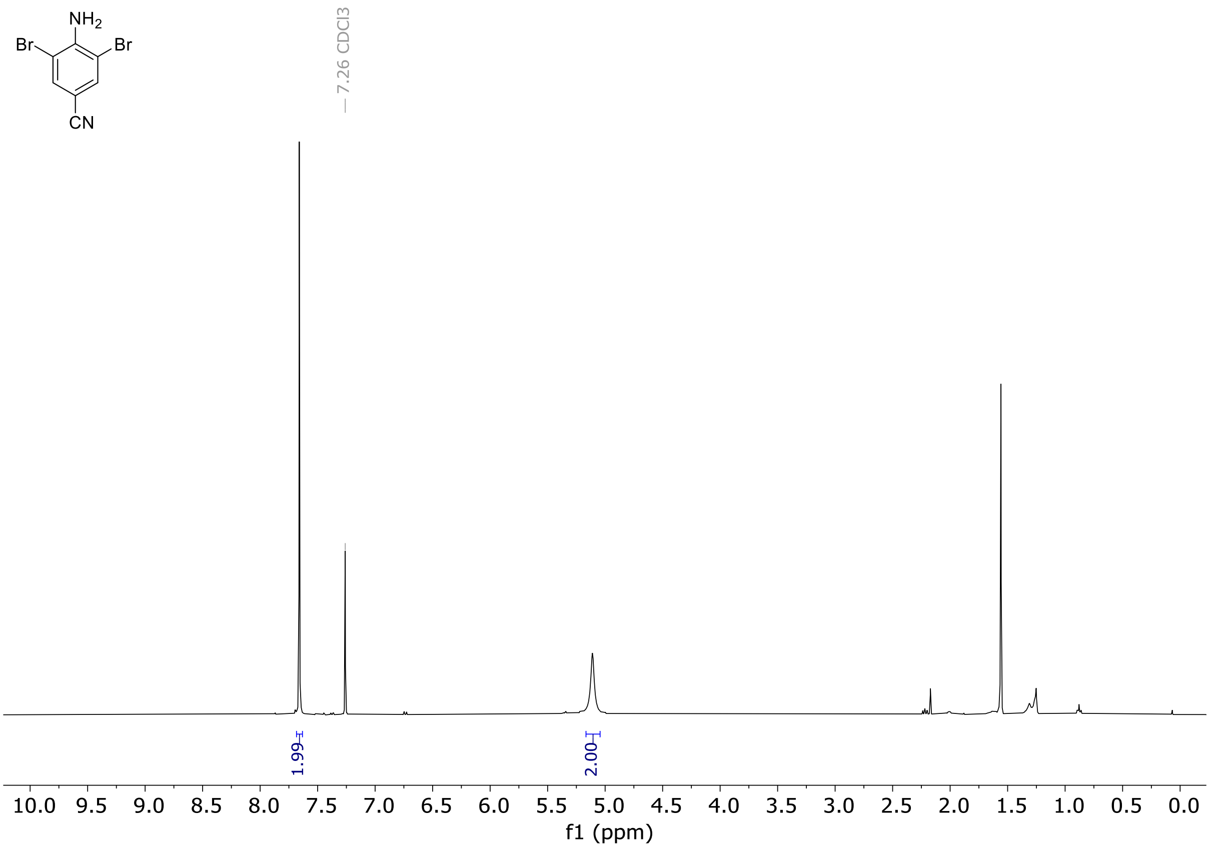

Figure S13. ^{1}H NMR Spectrum of **12** (Chloroform-*d*, 298 K).

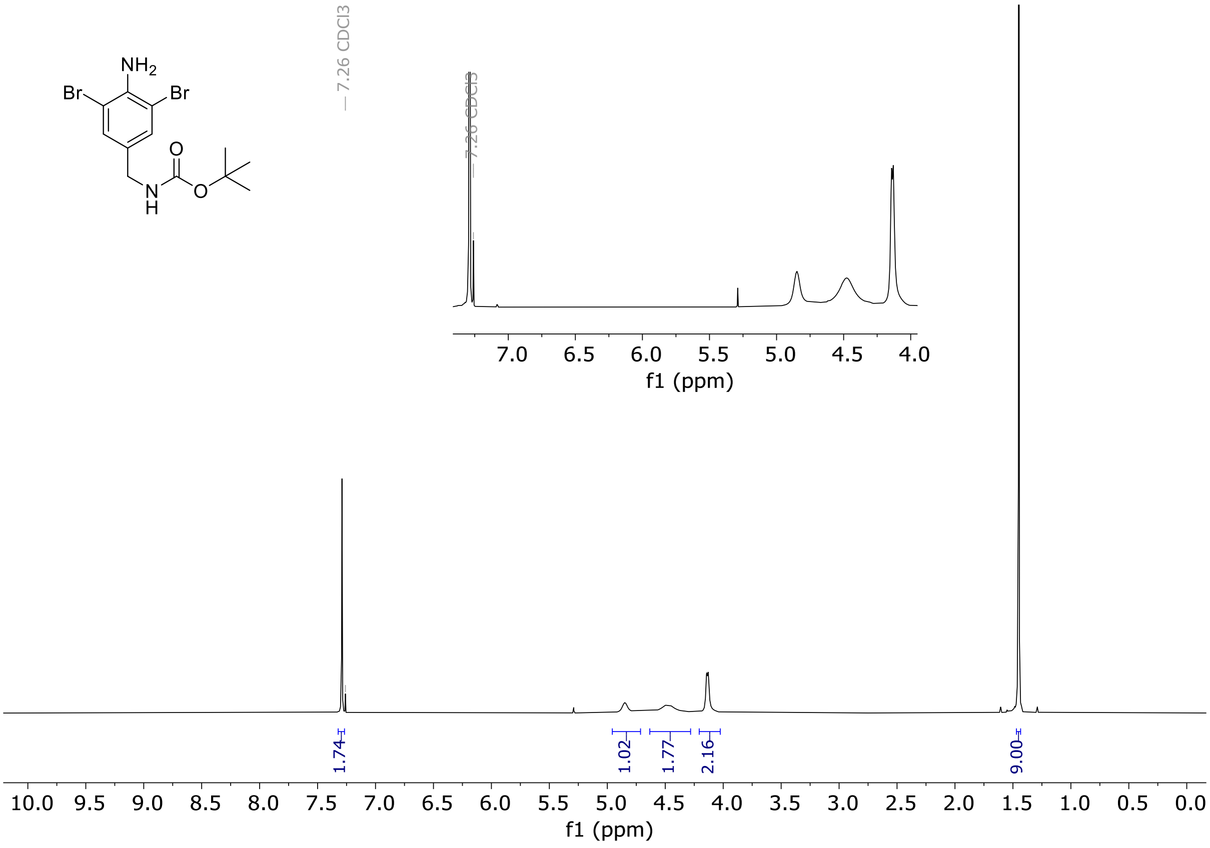

Figure S14. ^{1}H NMR Spectrum of **S14** (Chloroform-*d*, 298 K).

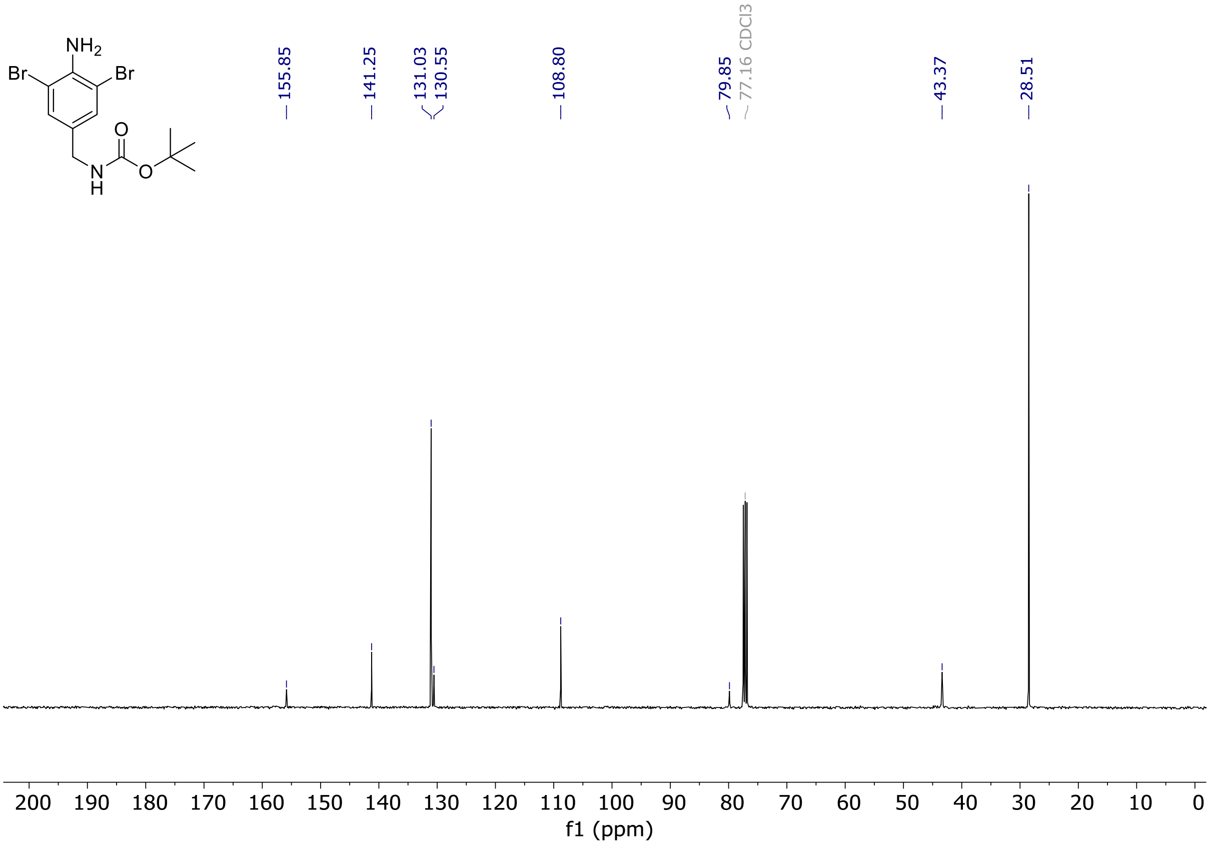

Figure S15. ^{13}C NMR Spectrum of **S14** (Chloroform-*d*, 298 K).

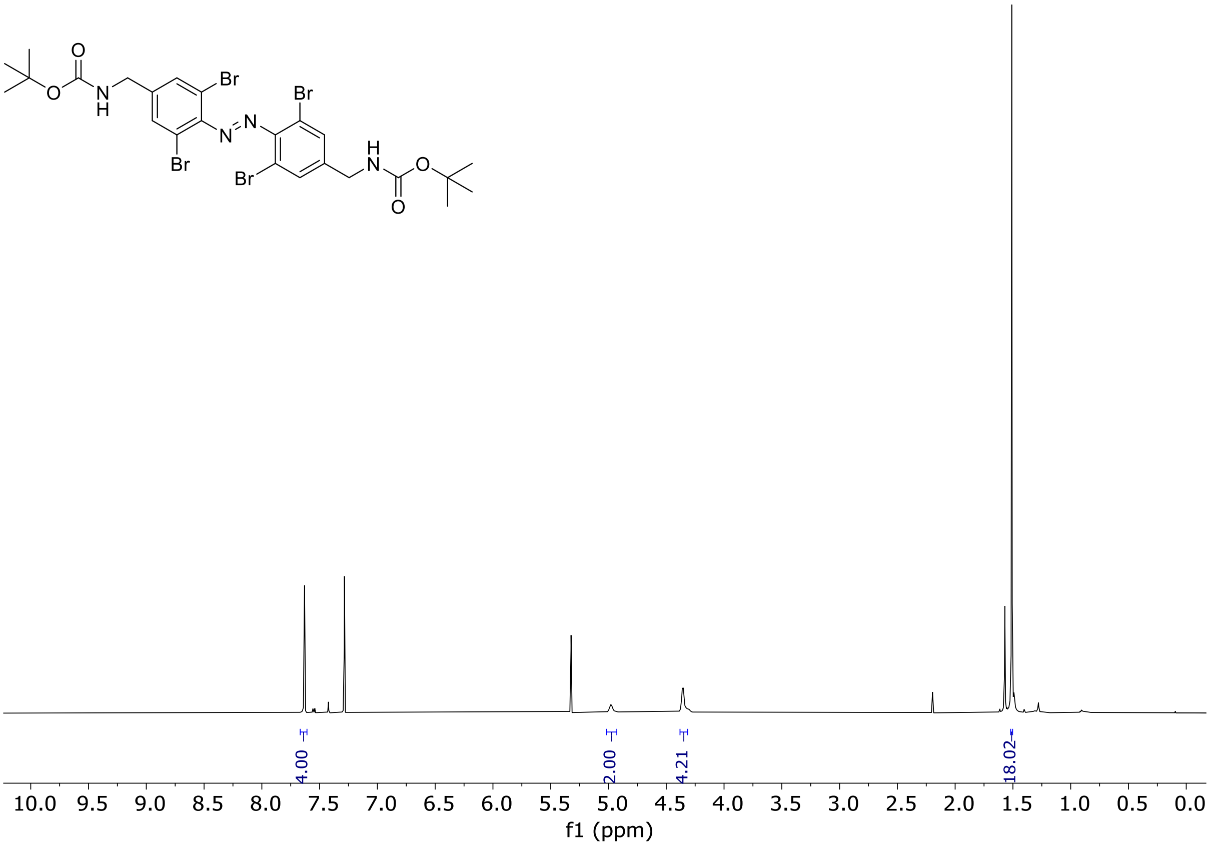

Figure S16. ^{1}H NMR Spectrum of (*E*)-**Br_4-NHBoc** (Chloroform-*d*, 298 K).

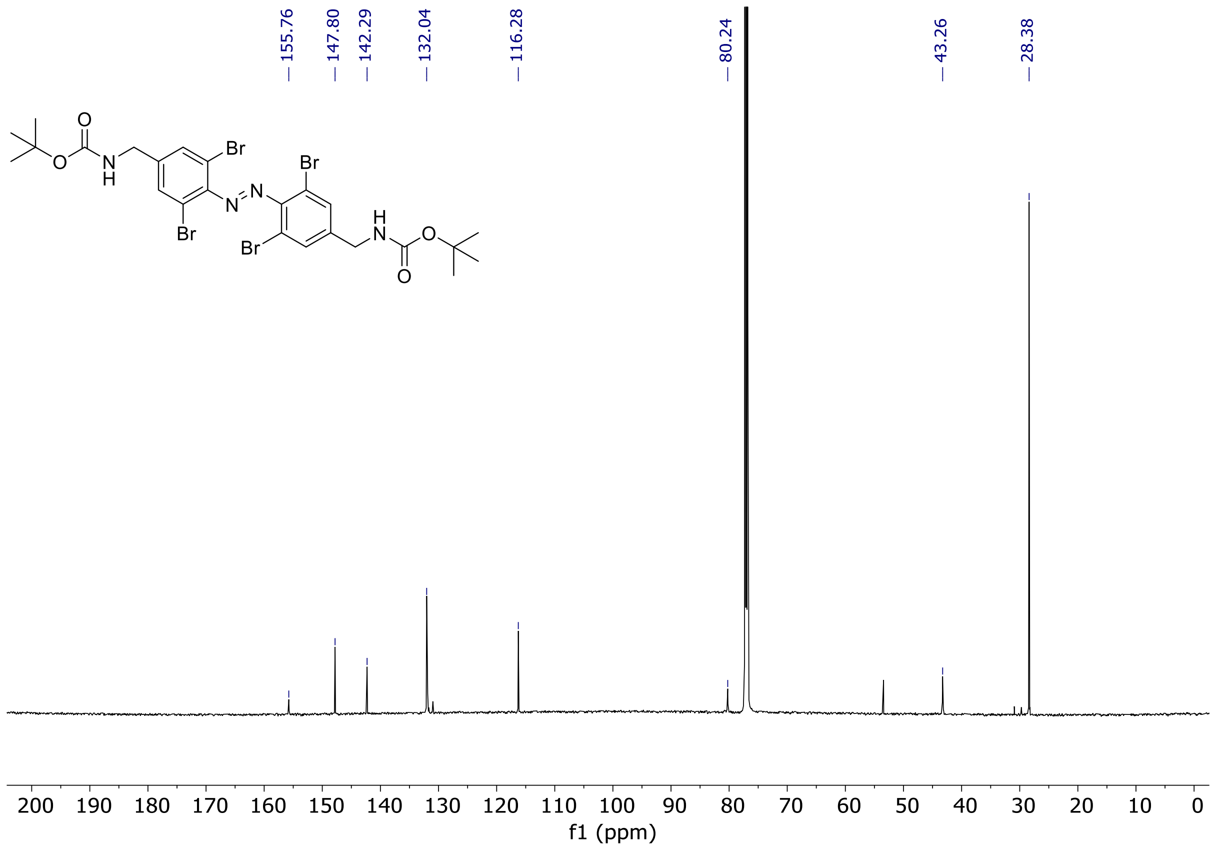

Figure S17. ^{13}C NMR Spectrum of (*E*)-**Br$_4$-NHBoc** (Chloroform-*d*, 298 K).

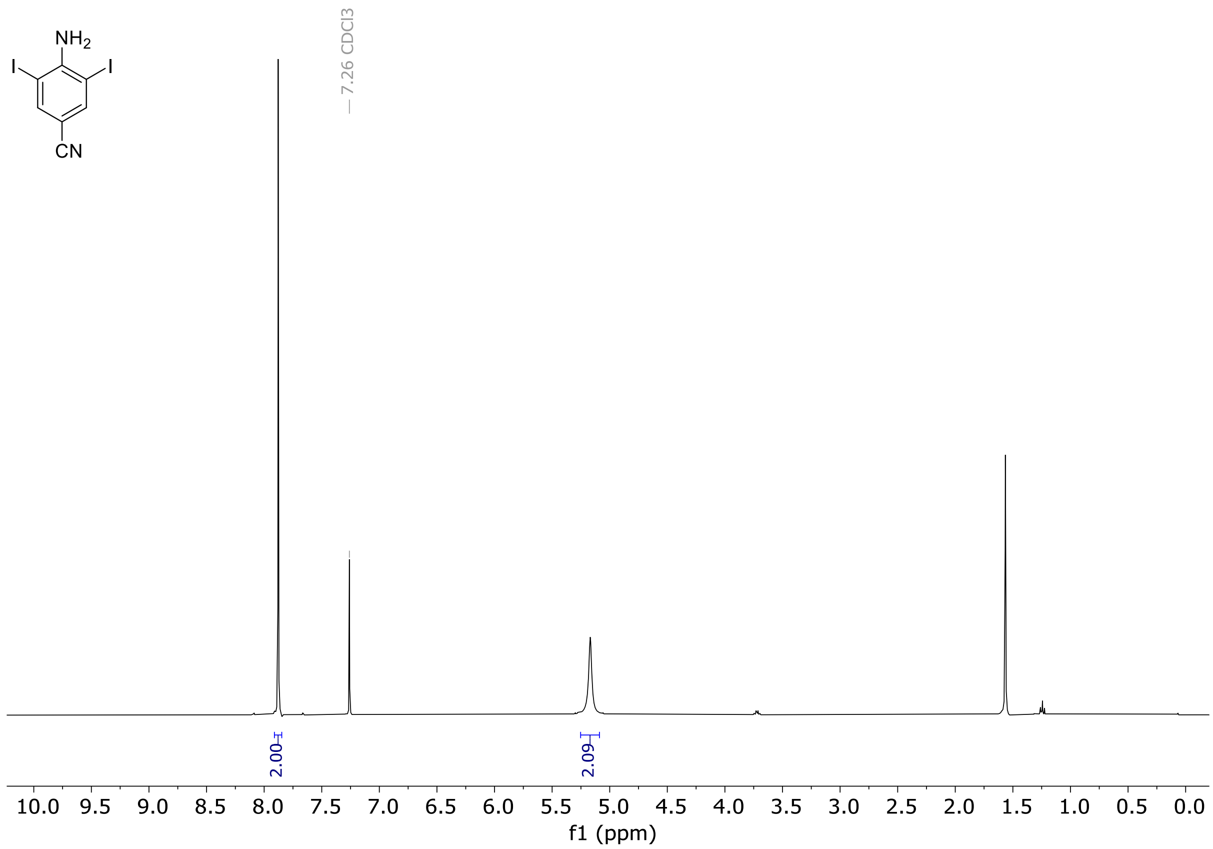

Figure S18. ^{1}H NMR Spectrum of **S16** (Chloroform-*d*, 298 K).

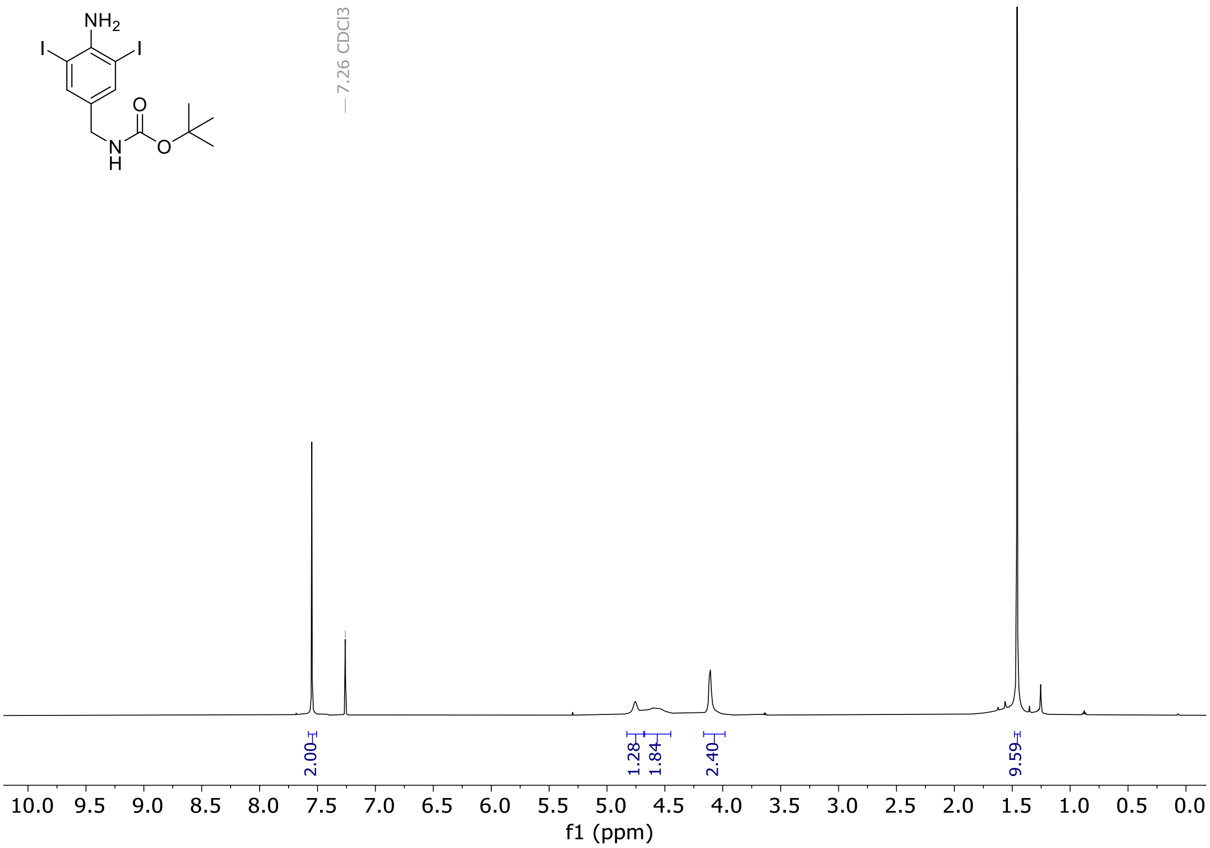

Figure S19. ^{1}H NMR Spectrum of **S17** (Chloroform-*d*, 298 K).

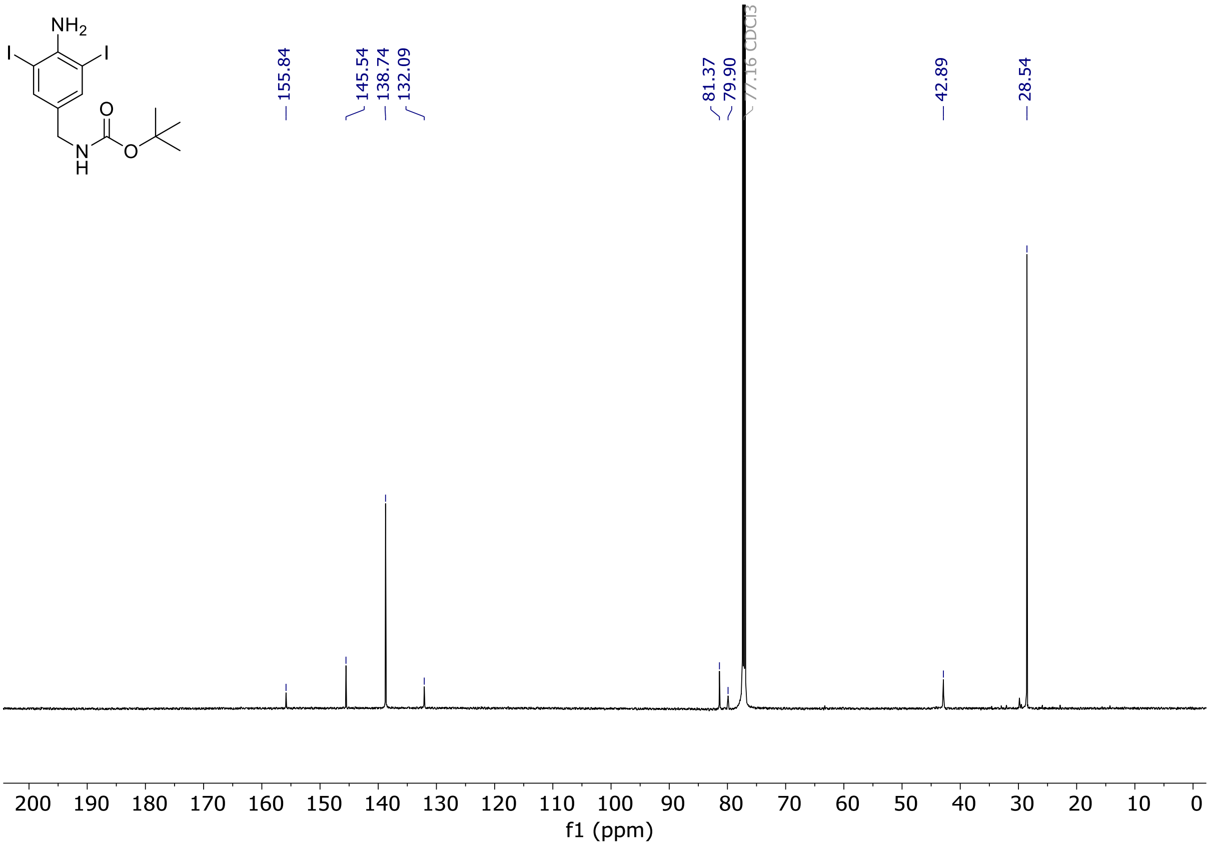

Figure S20. ^{13}C NMR Spectrum of **S17** (Chloroform-*d*, 298 K).

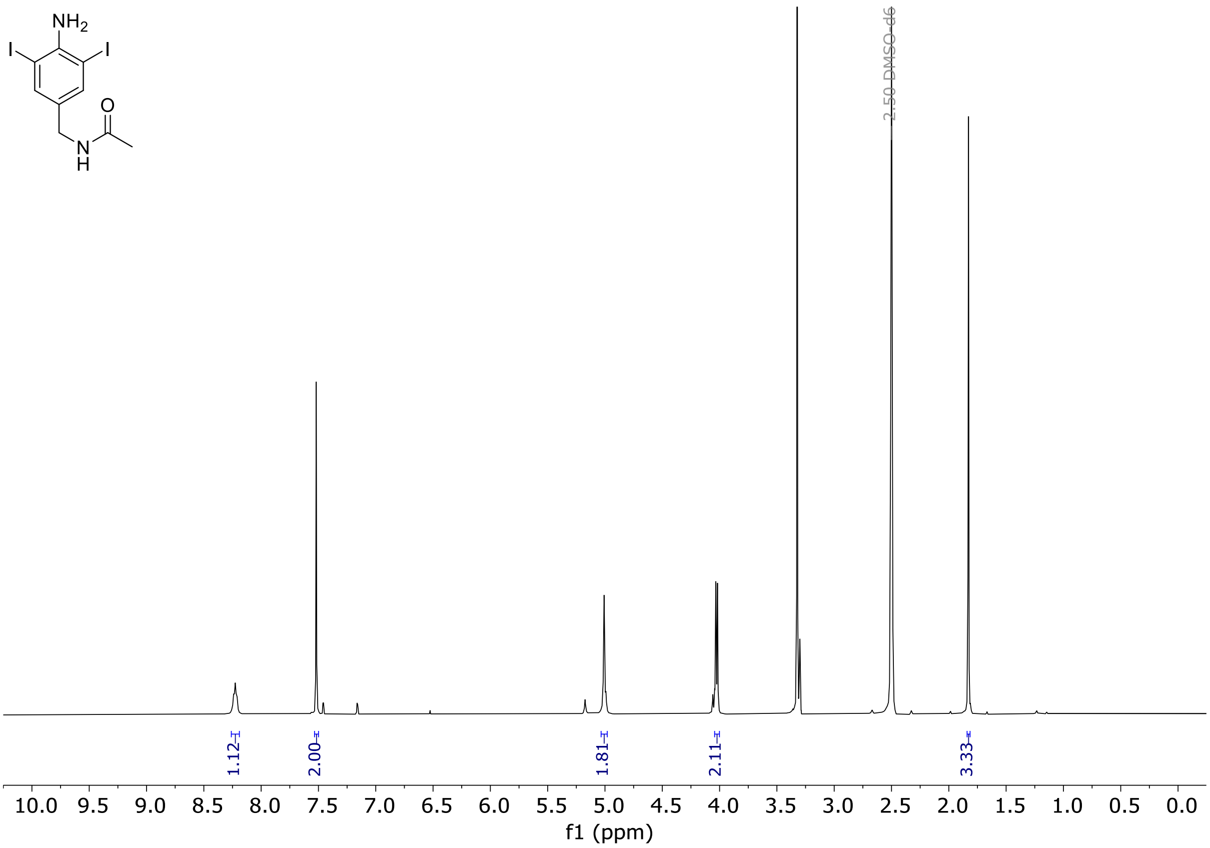

Figure S21. ^{1}H NMR Spectrum of **S18** (Chloroform-*d*, 298 K).

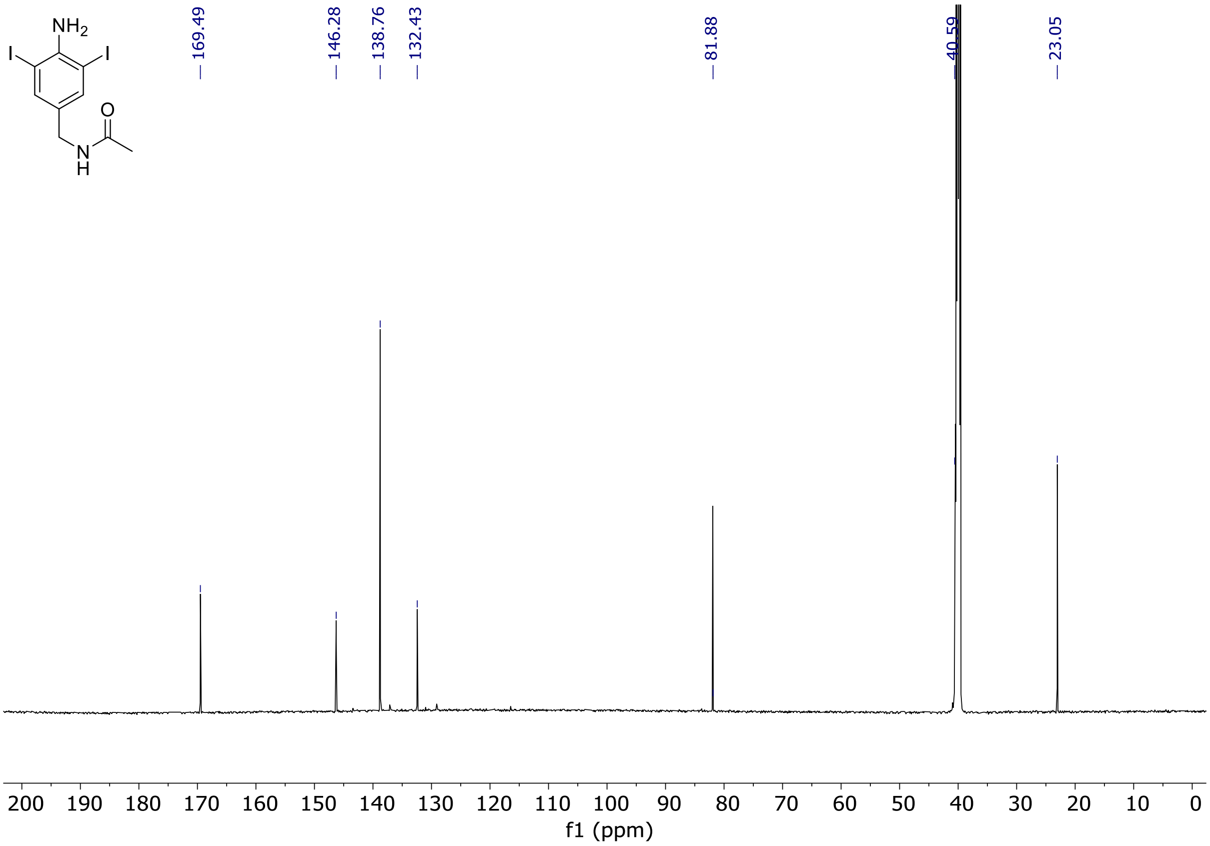

Figure S22. ^{13}C NMR Spectrum of **19** (Chloroform-*d*, 298 K).

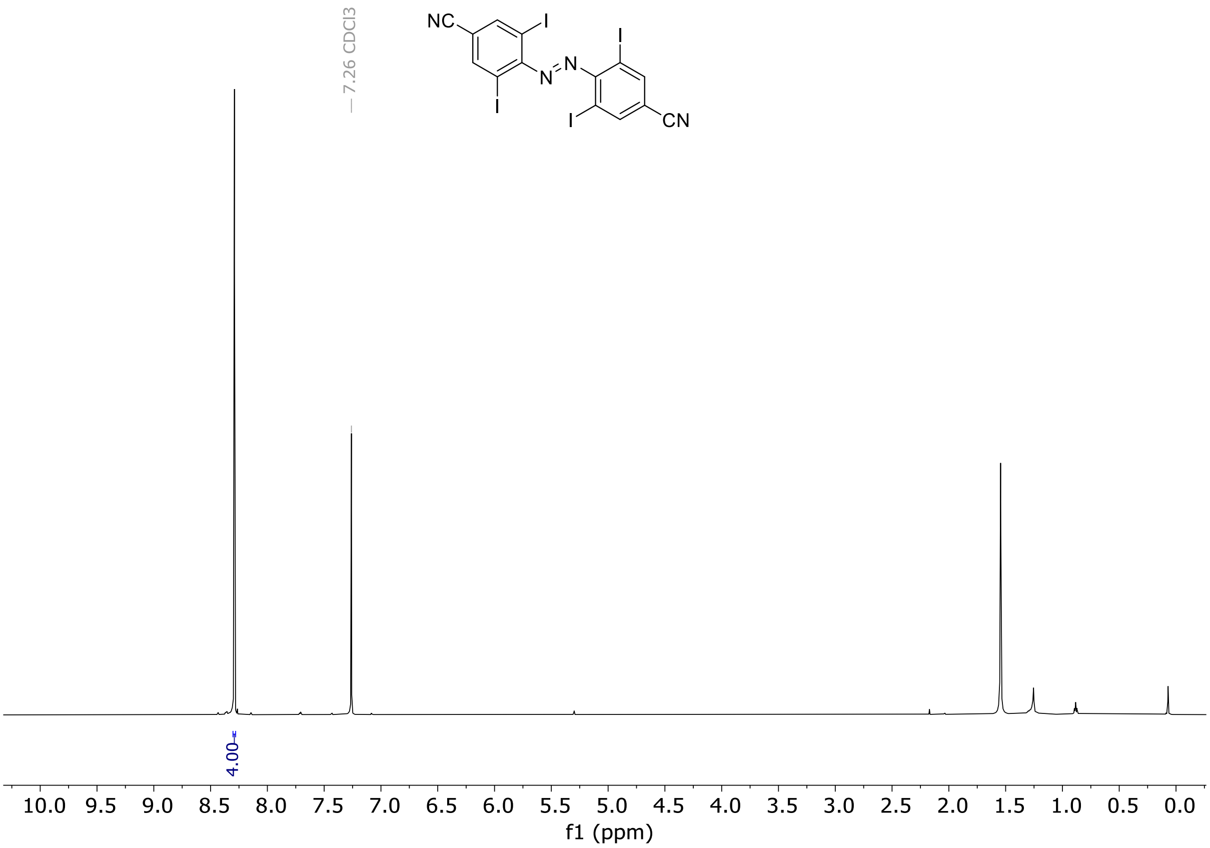

Figure S23. ^{1}H NMR Spectrum of **I$_4$-CN** (Chloroform-*d*, 298 K).

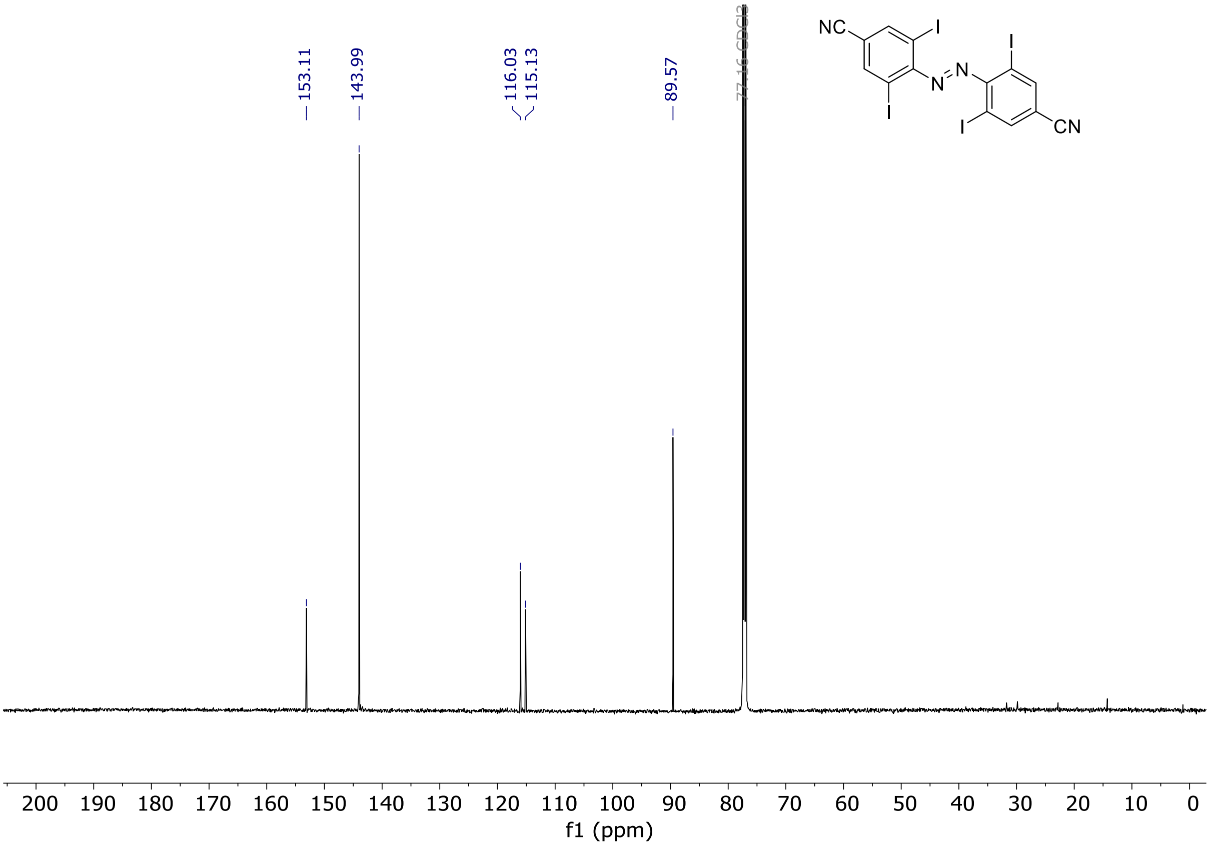

Figure S24. ^{13}C NMR Spectrum of **I$_4$-CN** (Chloroform-*d*, 298 K).

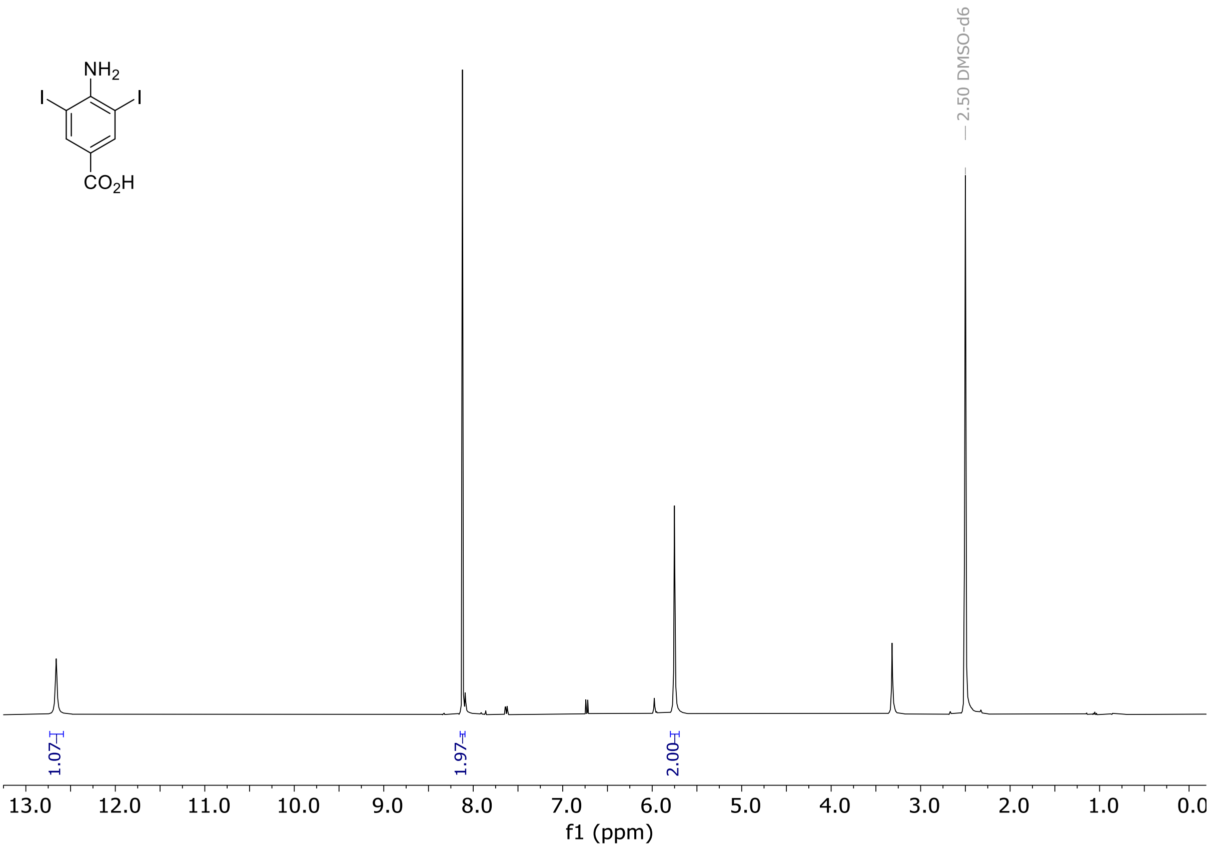

Figure S25. ^{1}H NMR spectrum of **S21** (DMSO-d_6, 298 K).

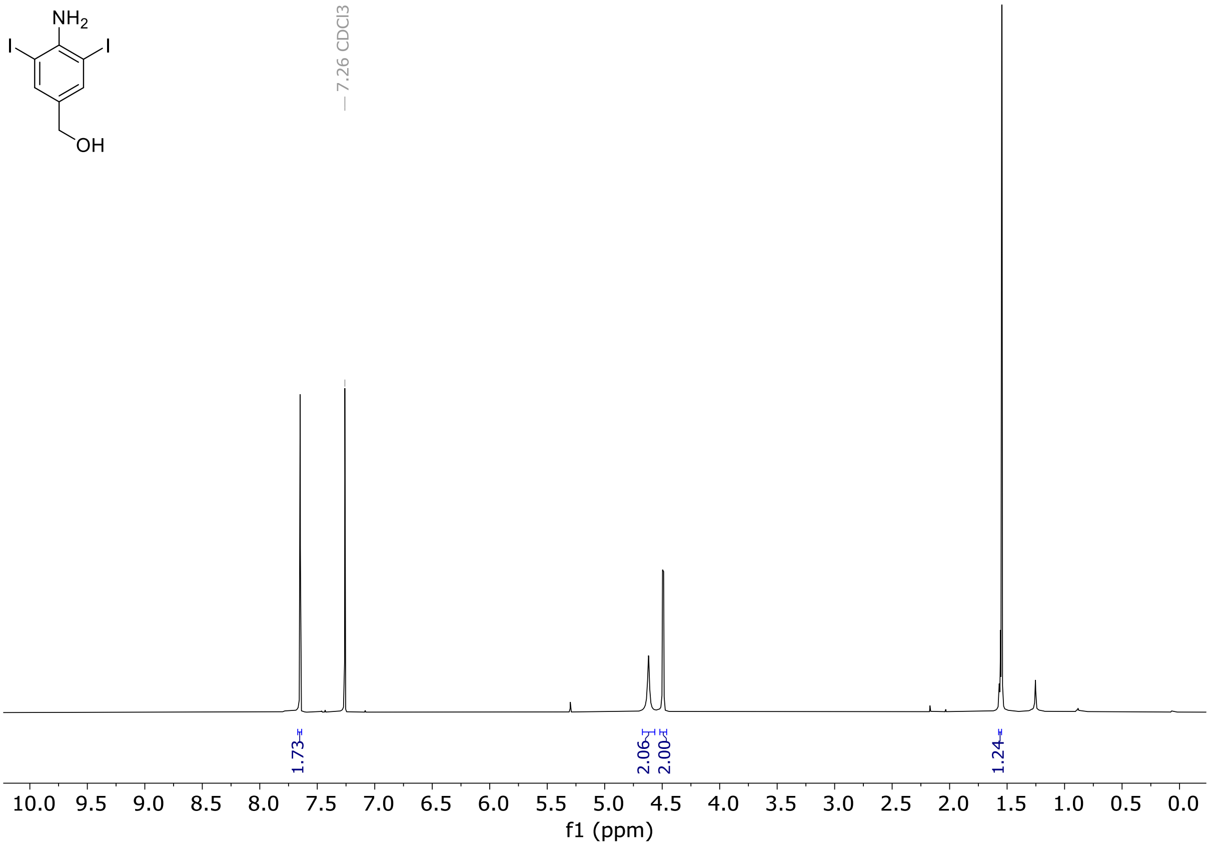

Figure S26. ^{1}H NMR Spectrum of **S22** (Chloroform-d, 298 K).

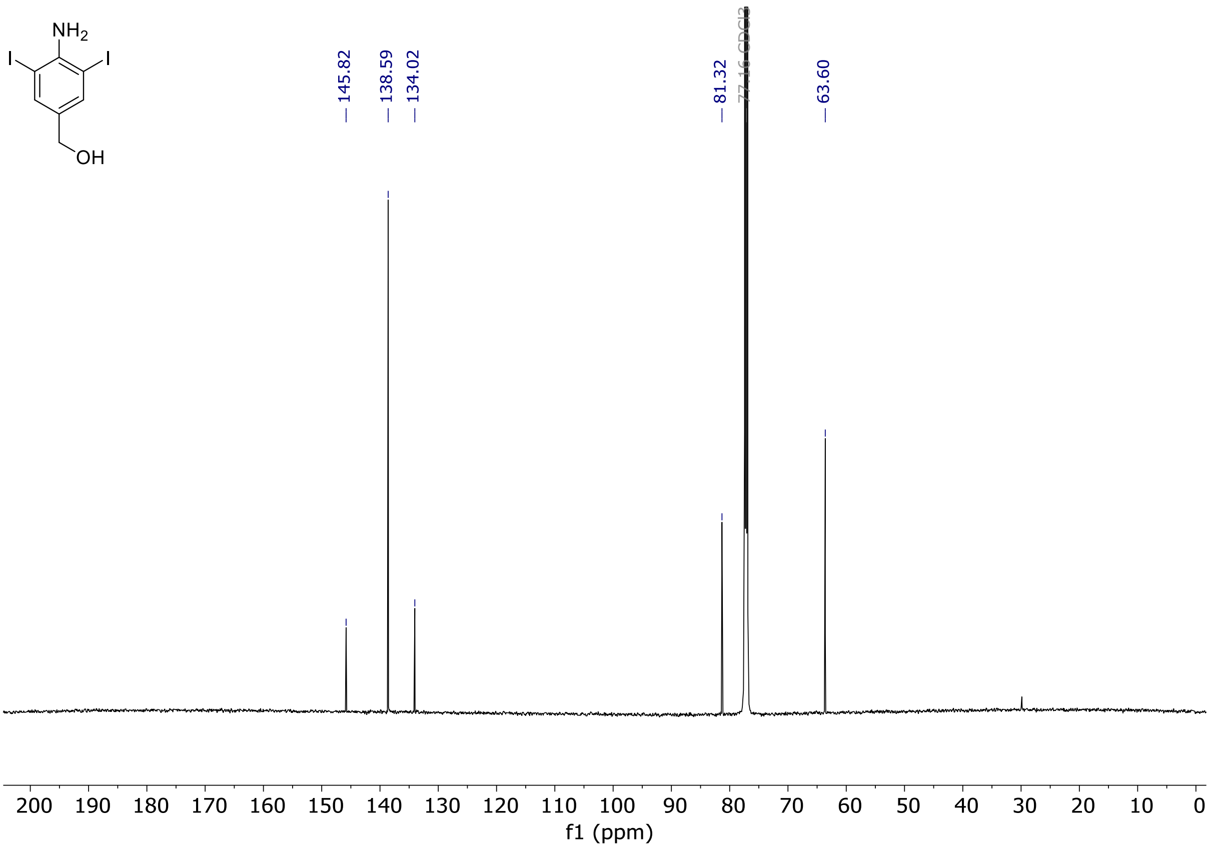

Figure S27. ^{13}C NMR Spectrum of **S22** (Chloroform-*d*, 298 K).

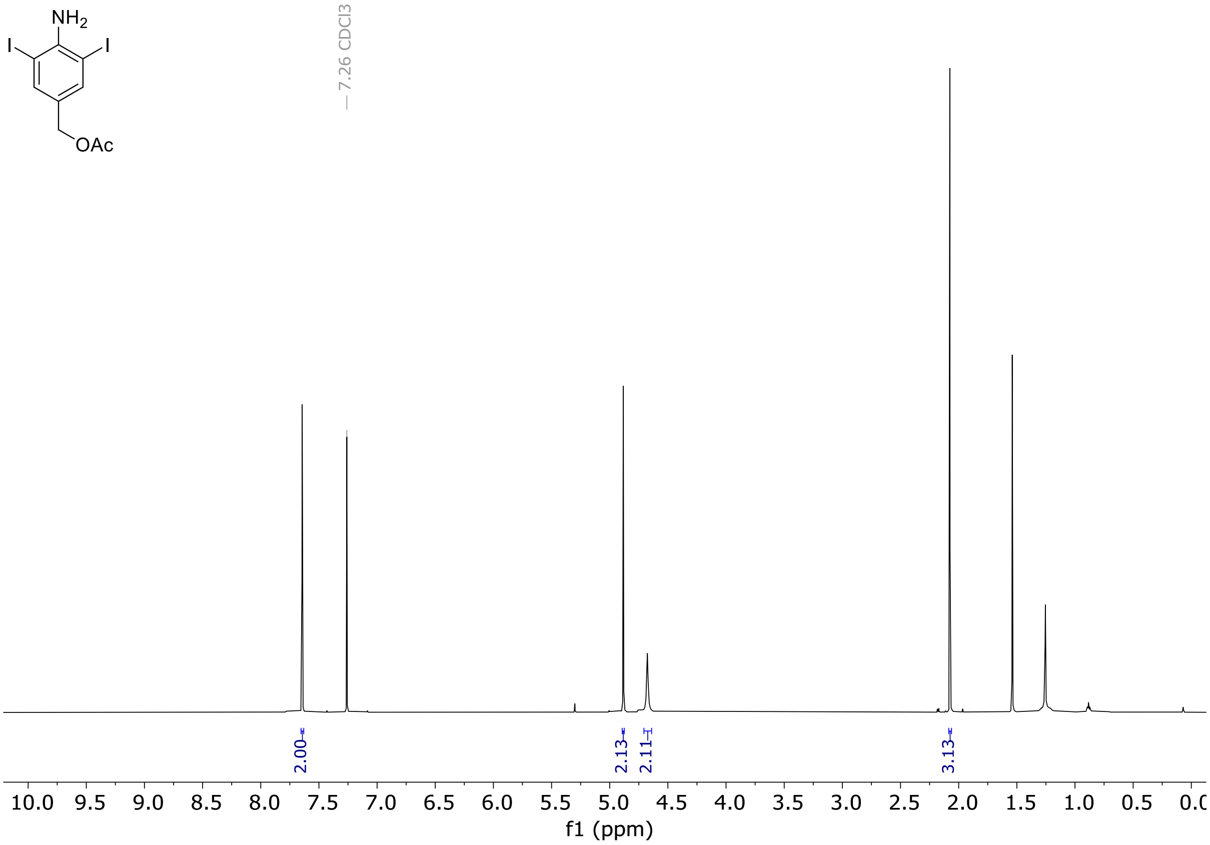

Figure S28. ^{1}H NMR Spectrum of **S23** (Chloroform-*d*, 298 K).

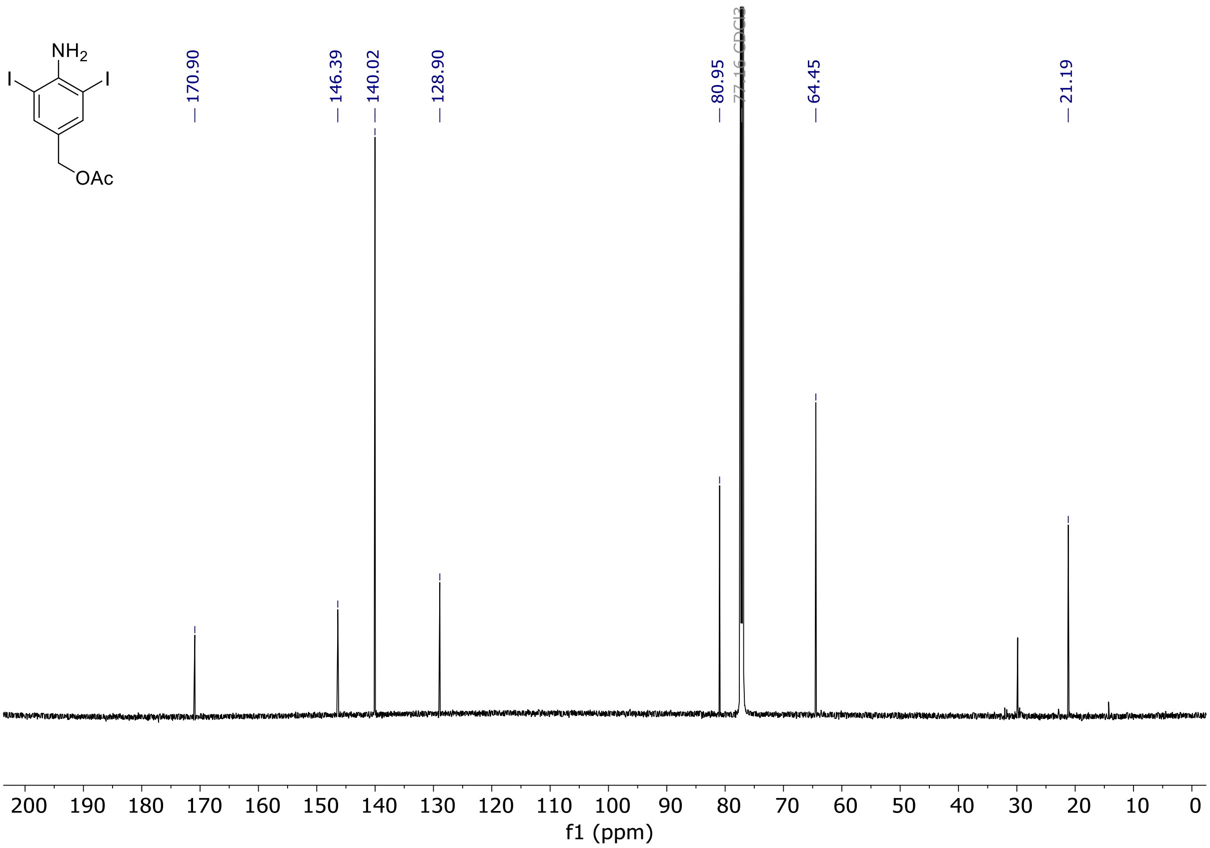

Figure S29. ^{13}C NMR Spectrum of **S23** (Chloroform-*d*, 298 K).

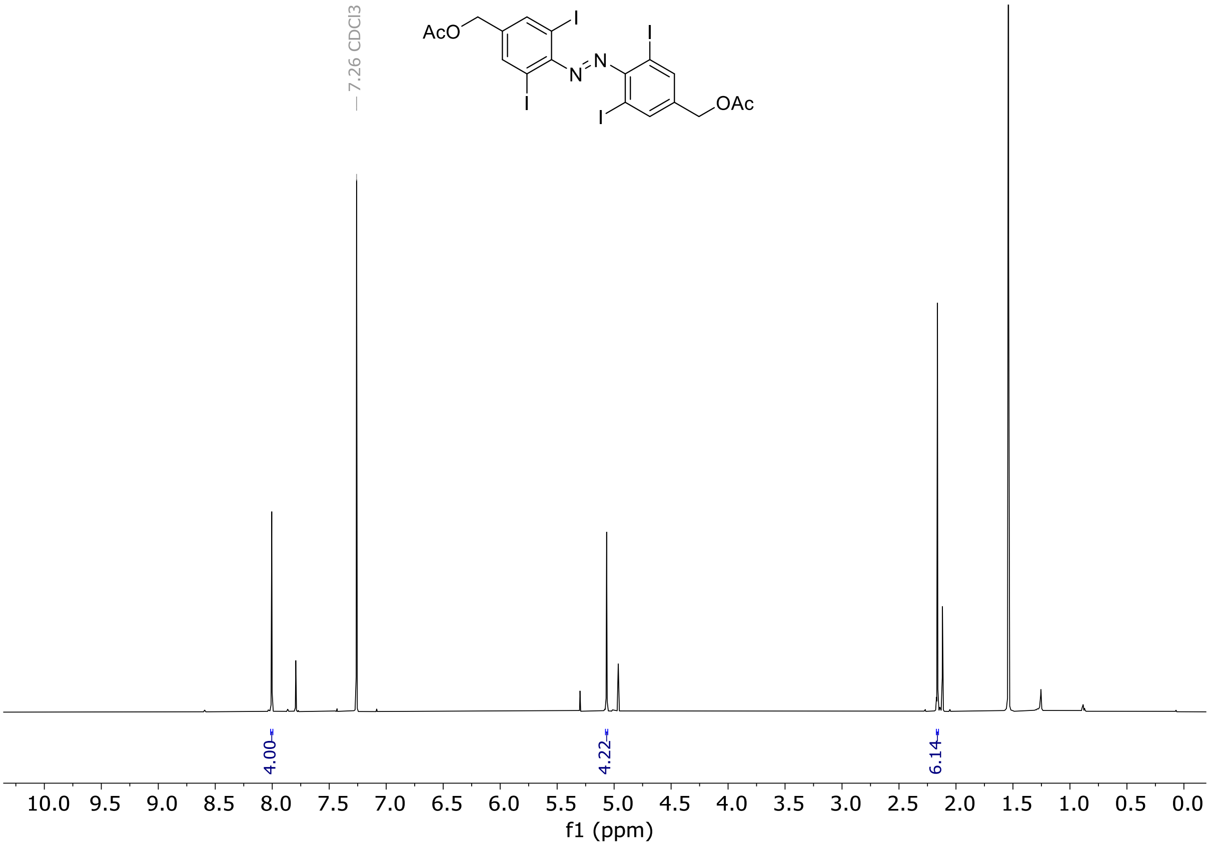

Figure S30. ^{1}H NMR Spectrum of **I_4-OAc** (Chloroform-*d*, 298 K).

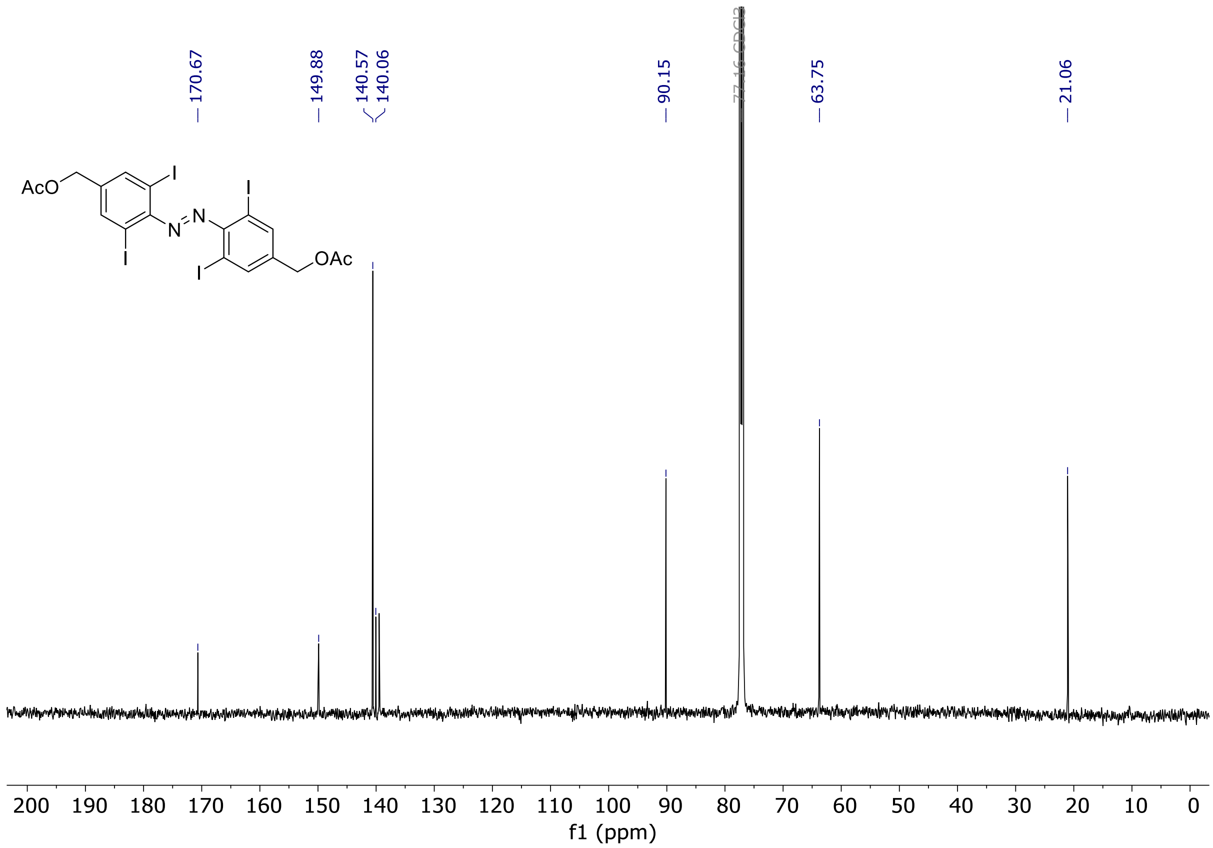

Figure S31. ^{13}C NMR Spectrum of **I$_4$-OAc** (Chloroform-*d*, 298 K).

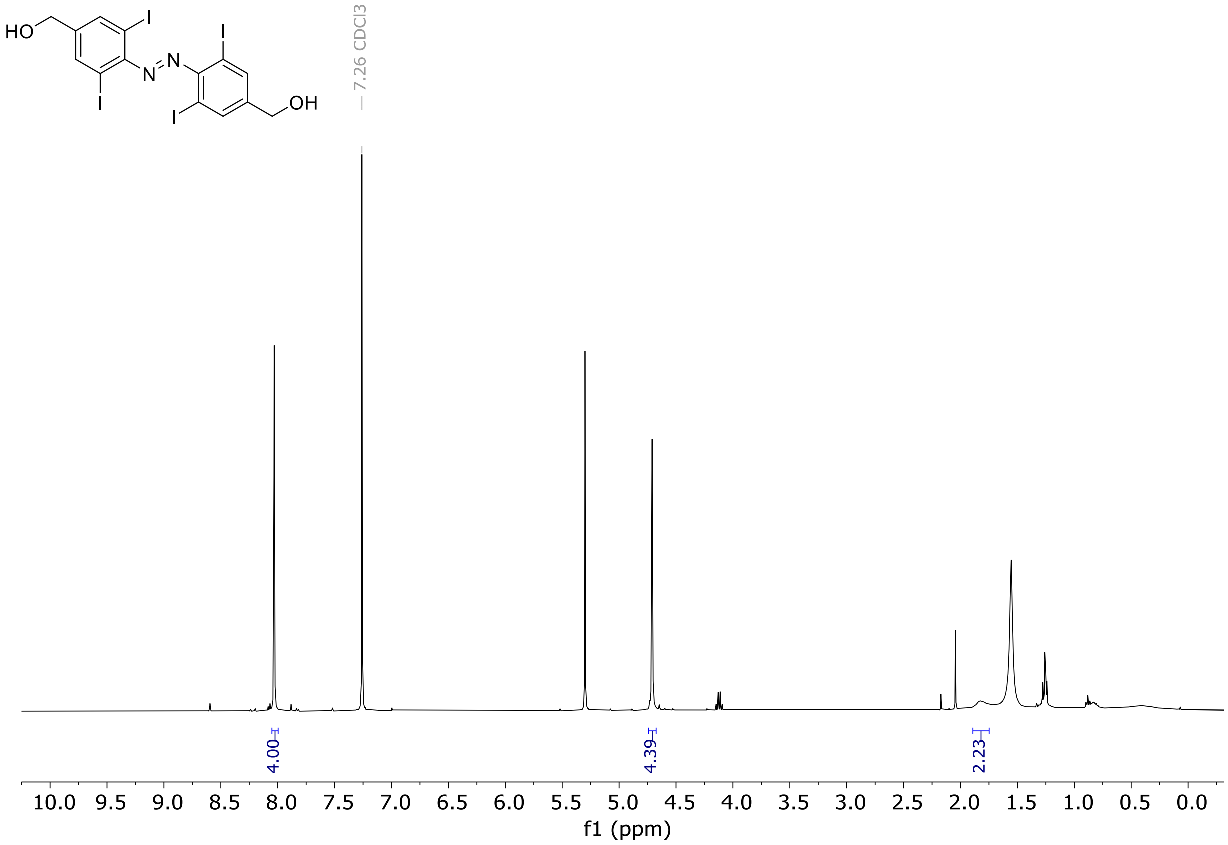

Figure S32. ^{1}H NMR Spectrum of **I$_4$-OH** (Chloroform-*d*, 298 K).

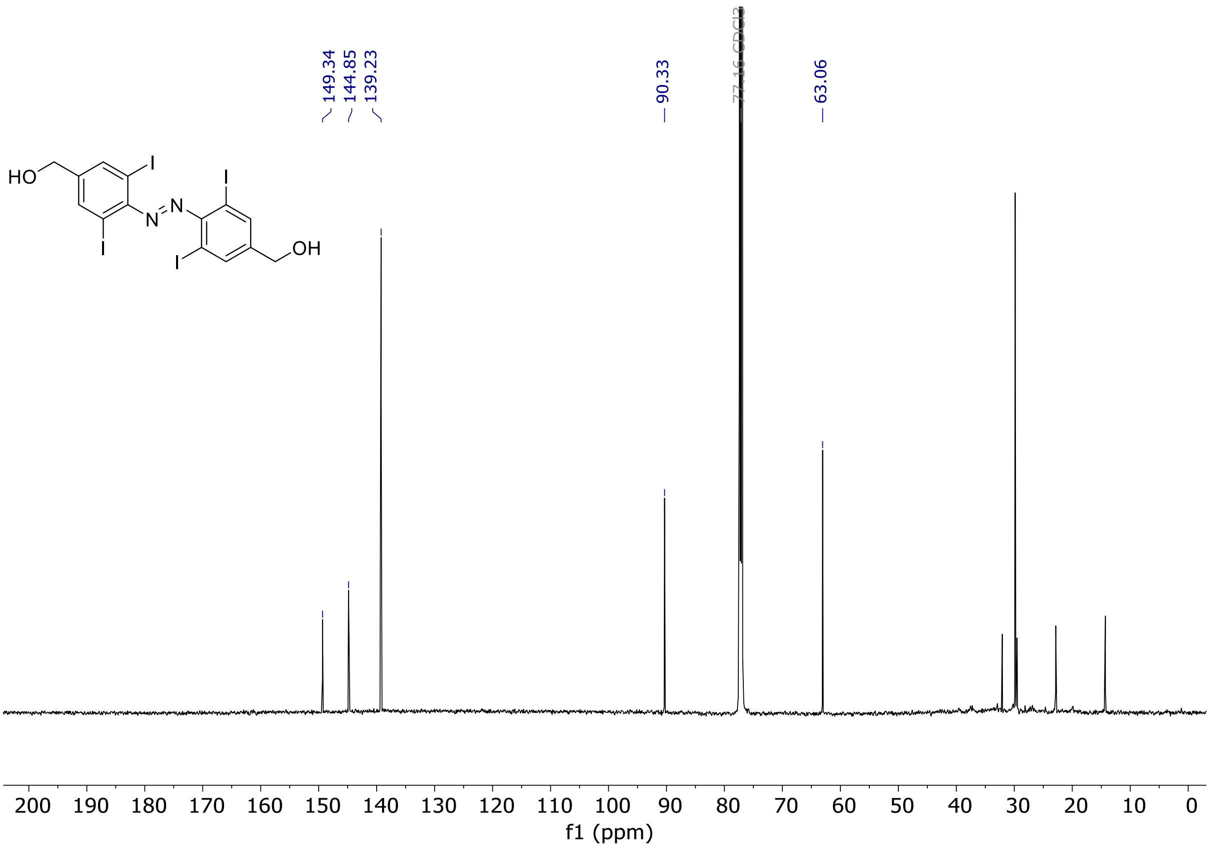

Figure S33. ^{13}C NMR Spectrum of **I$_4$-OH** (Chloroform-*d*, 298 K).

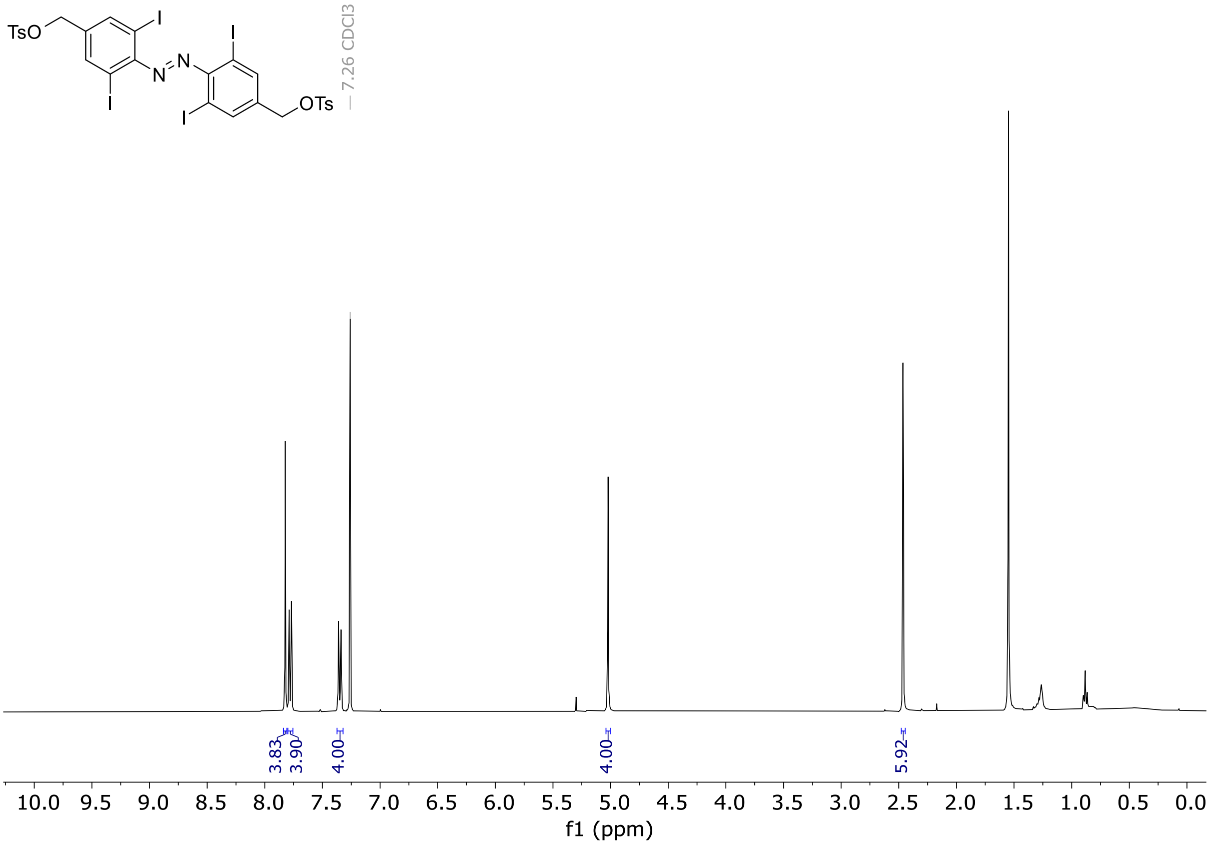

Figure S34. ^{1}H NMR Spectrum of **S25** (Chloroform-*d*, 298 K).

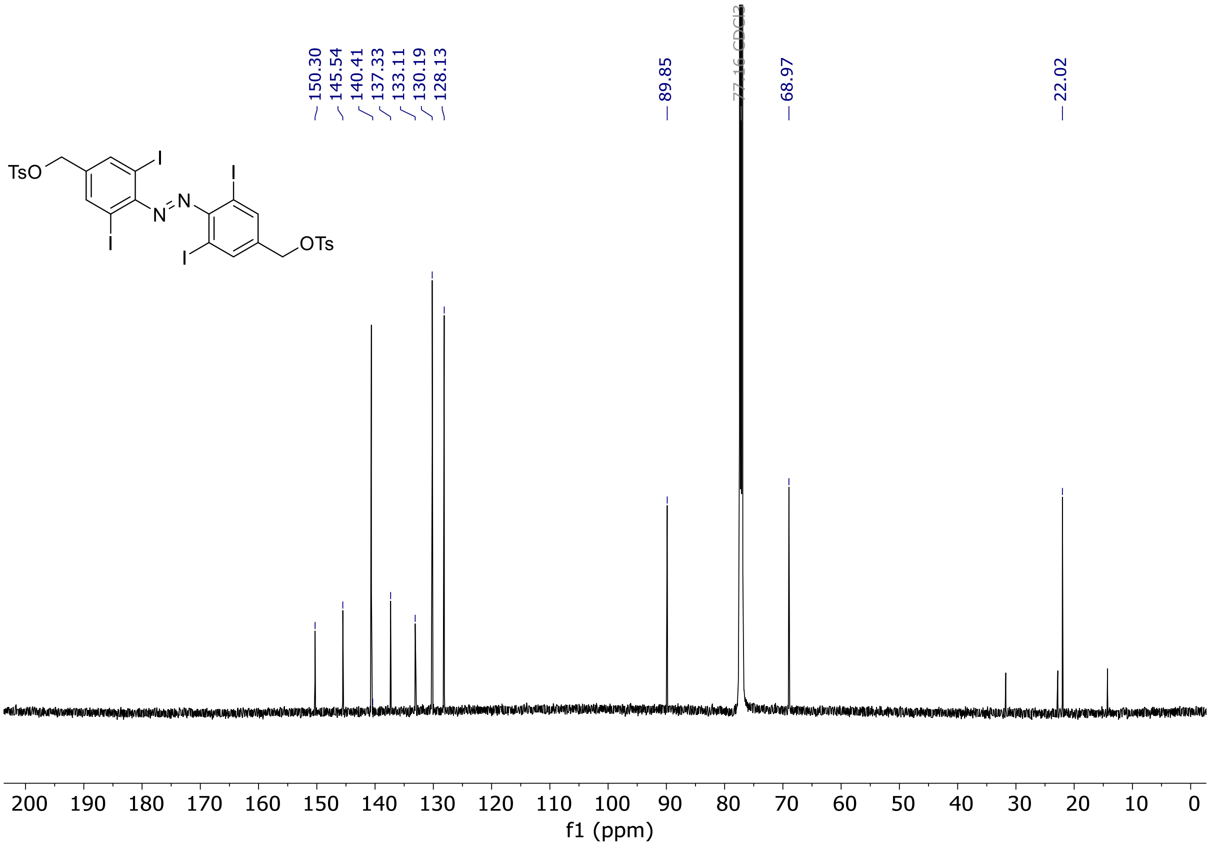

Figure S35. ^{13}C NMR Spectrum of **S25** (Chloroform-*d*, 298 K).

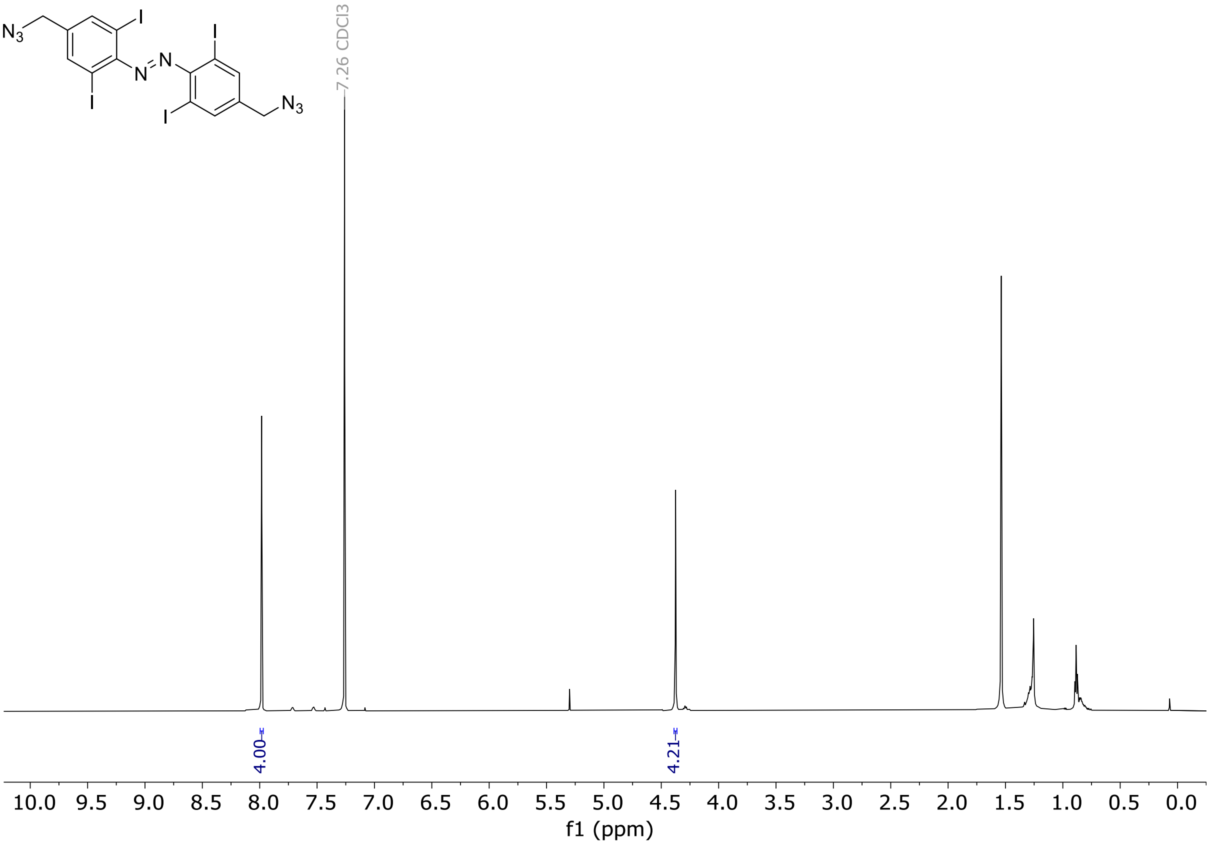

Figure S36. ^{1}H NMR Spectrum of **I_4-N_3** (Chloroform-*d*, 298 K).

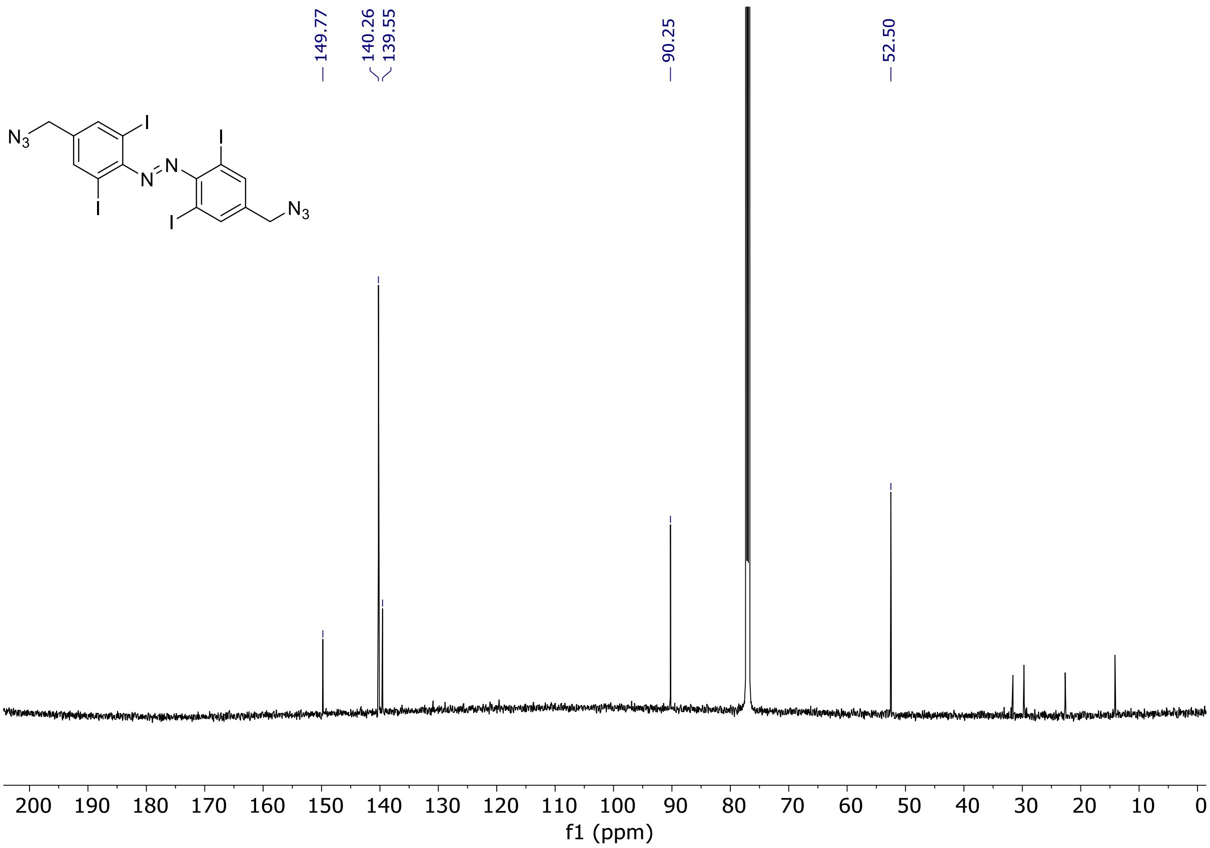

Figure S37. ^{13}C NMR Spectrum of **I_4-N_3** (Chloroform-*d*, 298 K).

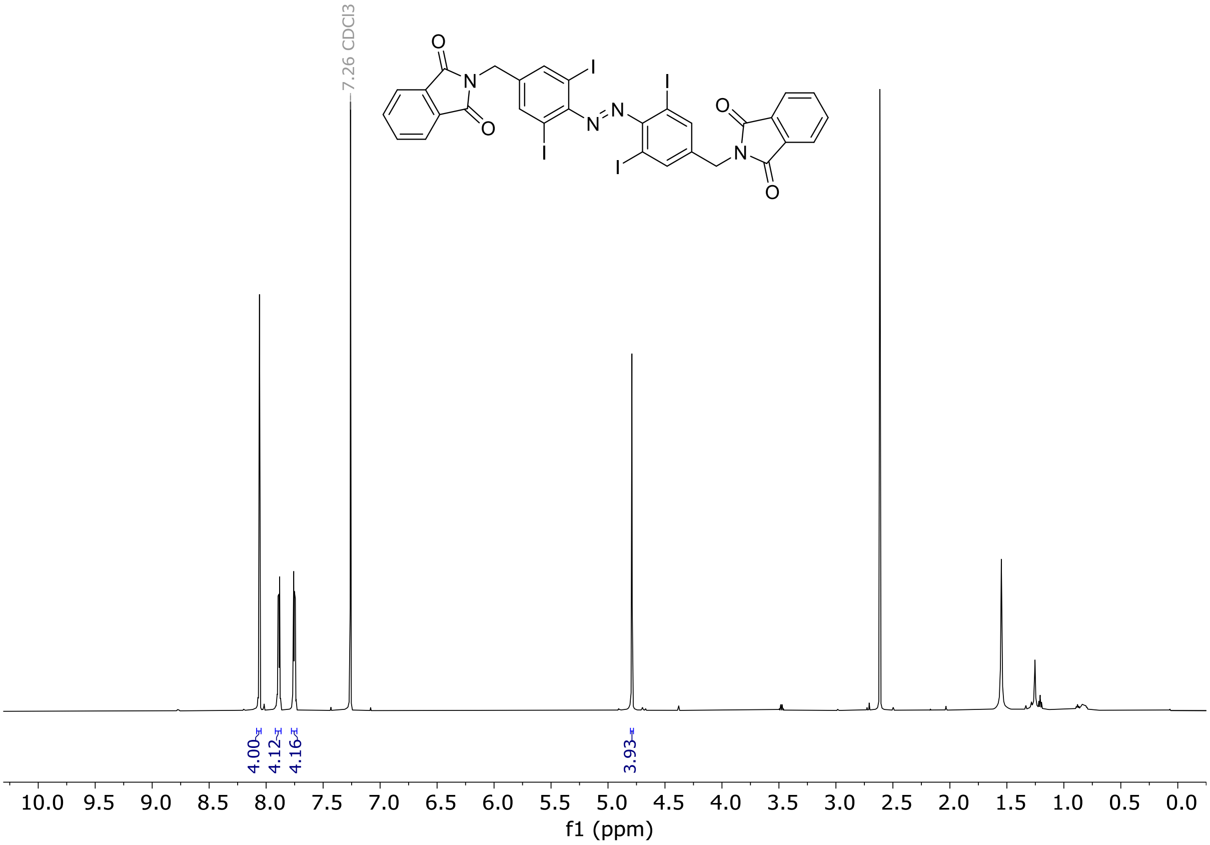

Figure S38. ^{1}H NMR Spectrum of **I_4-NPhth** (Chloroform-*d*, 298 K).

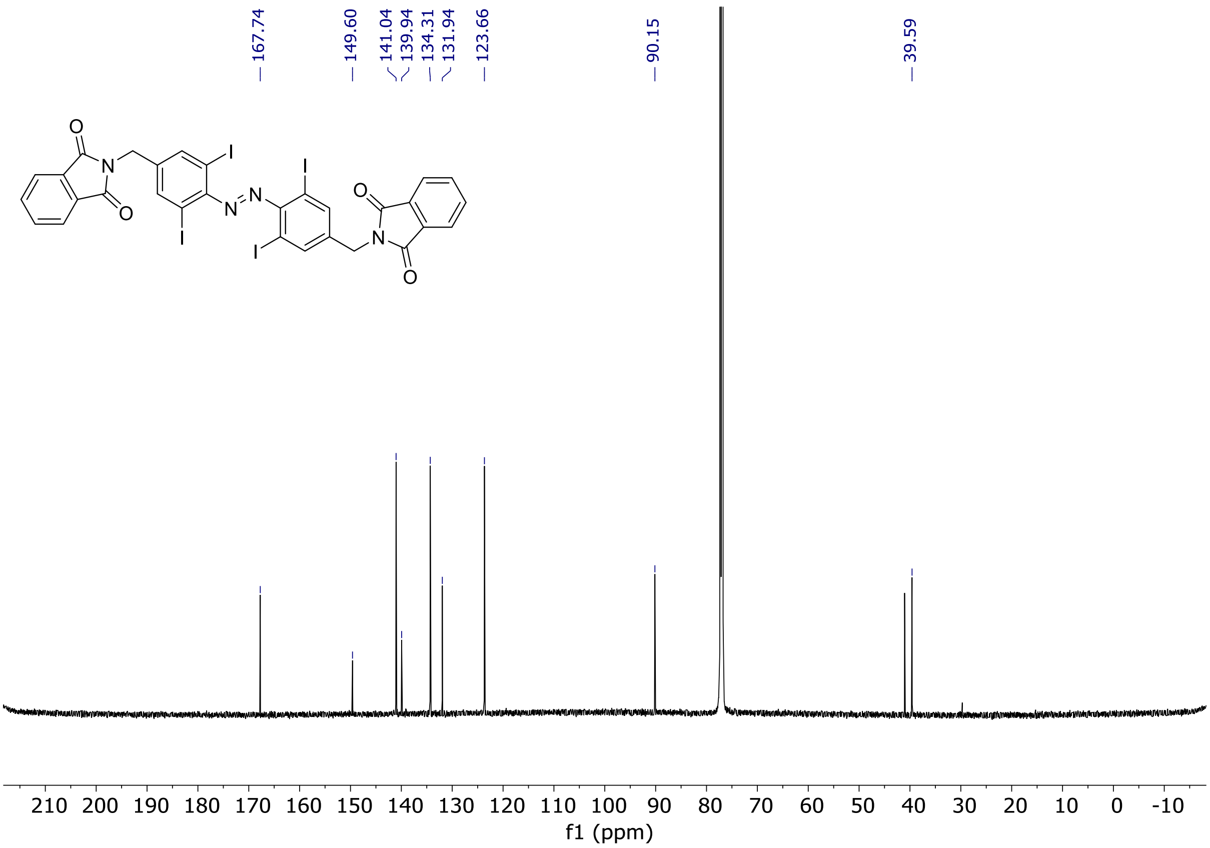

Figure S39. ^{13}C NMR Spectrum of **I$_4$-NPhth** (Chloroform-*d*, 298 K).

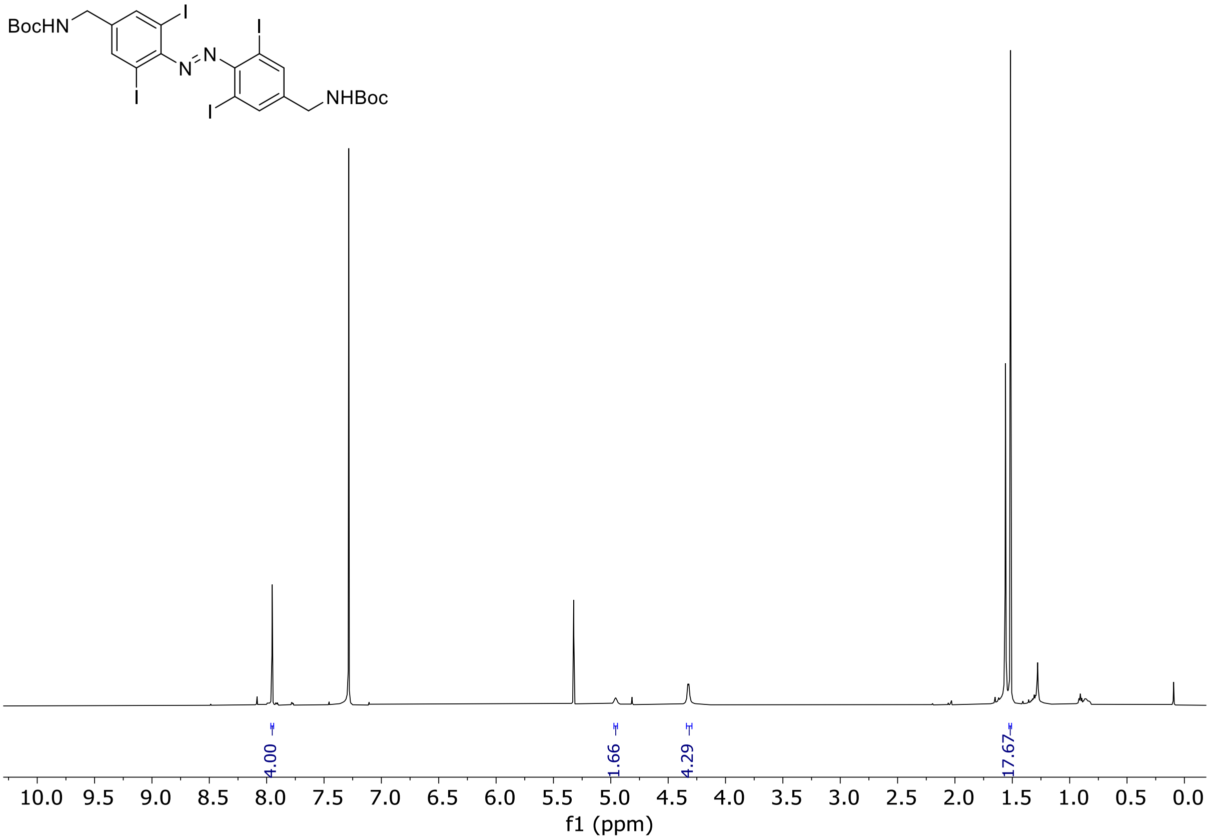

Figure S40. ^{1}H NMR Spectrum of **I$_4$-NHBoc** (Chloroform-*d*, 298 K).

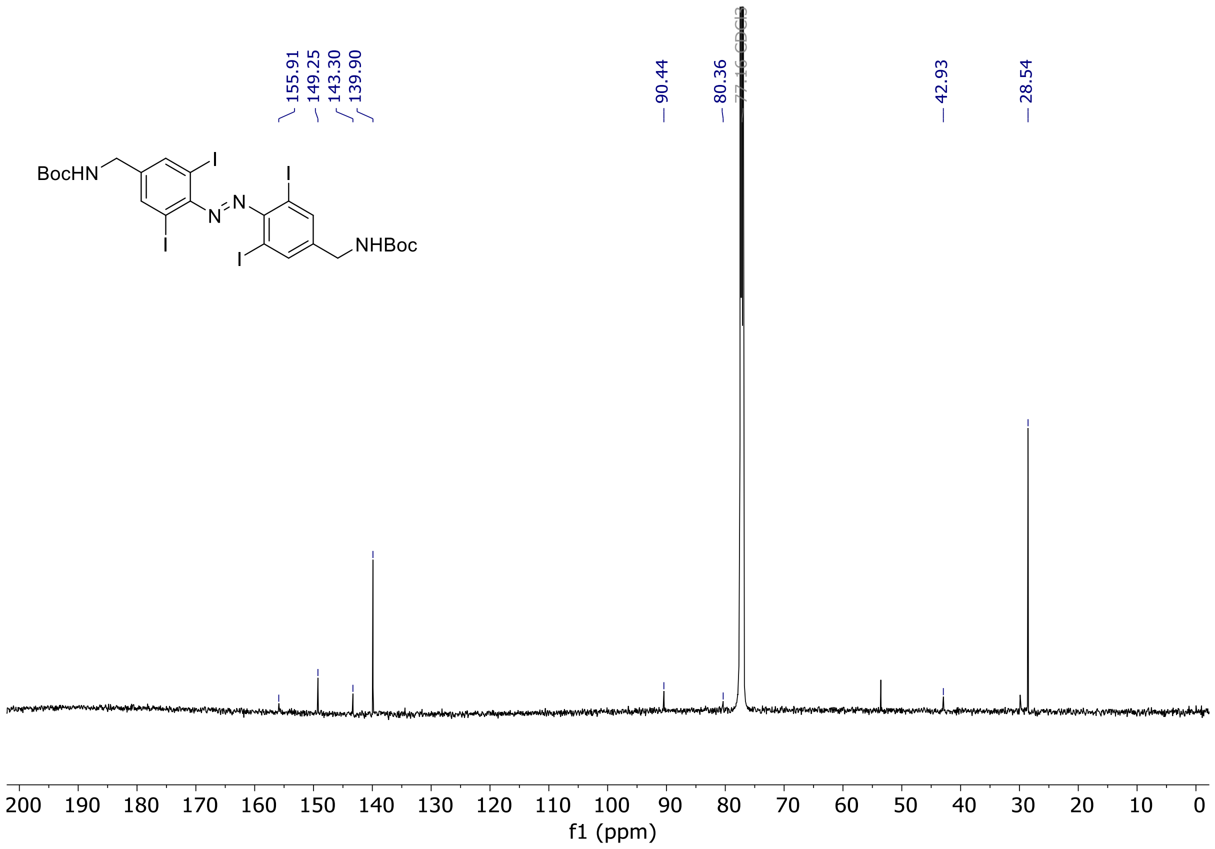

Figure S41. ^{13}C NMR Spectrum of **I_4-NHBoc** (Chloroform-*d*, 298 K).

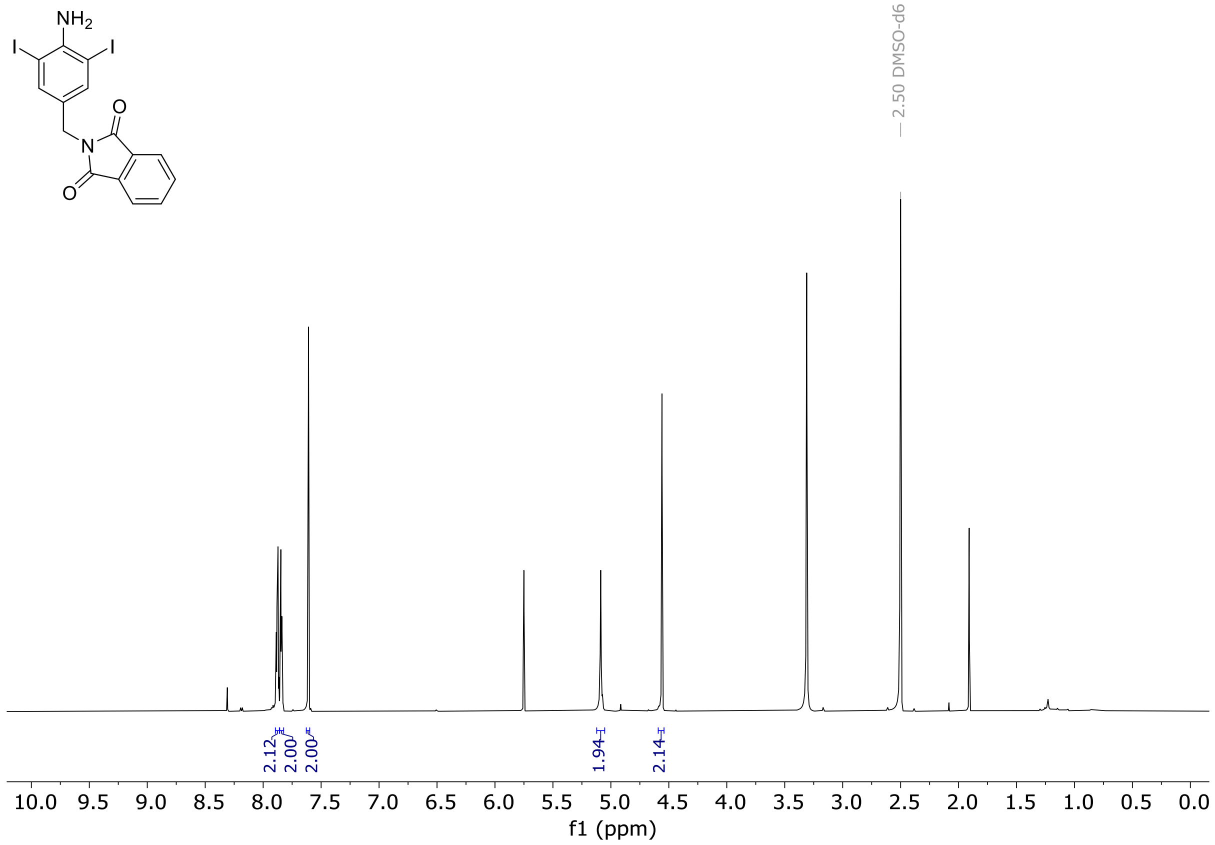

Figure S42. ^{1}H NMR spectrum of **S28** (DMSO-d_6, 298 K).

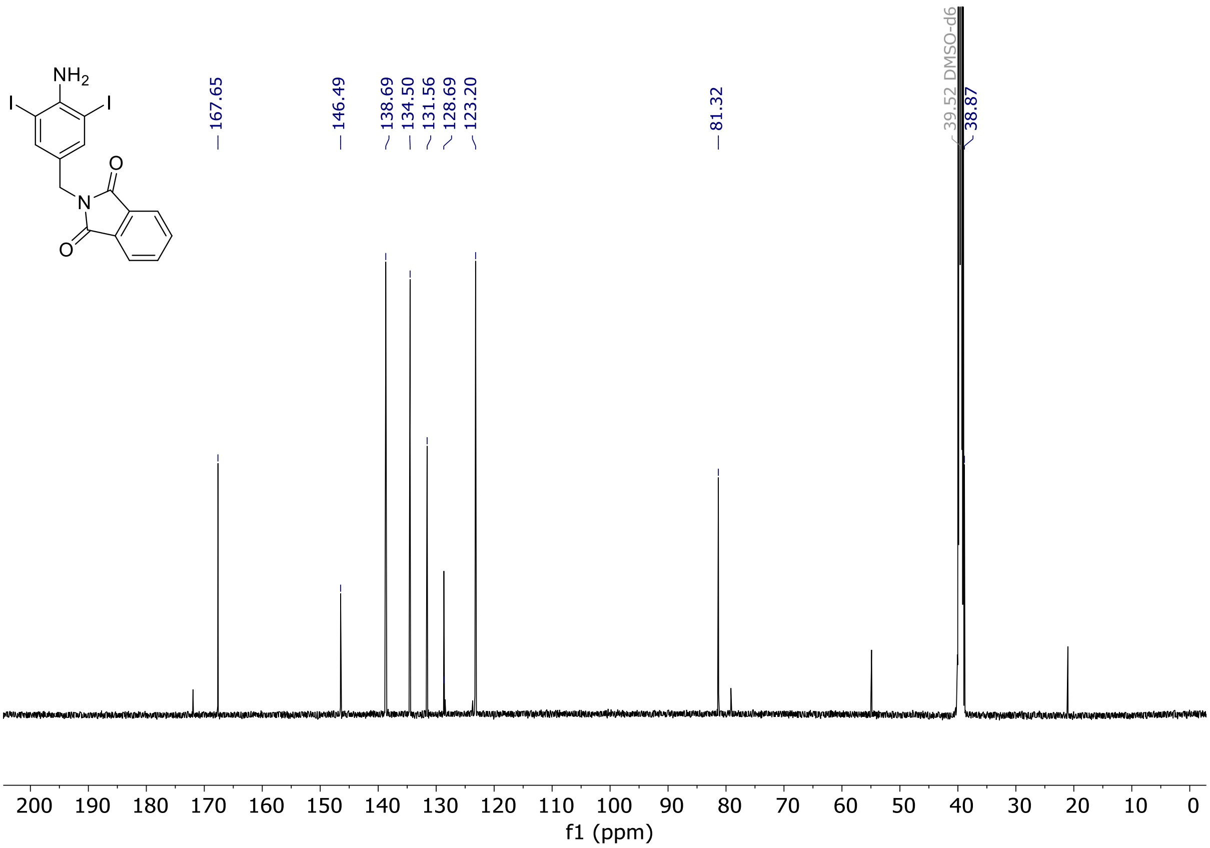

Figure S43. ^{1}H NMR spectrum of **S28** (DMSO-d_6, 298 K).

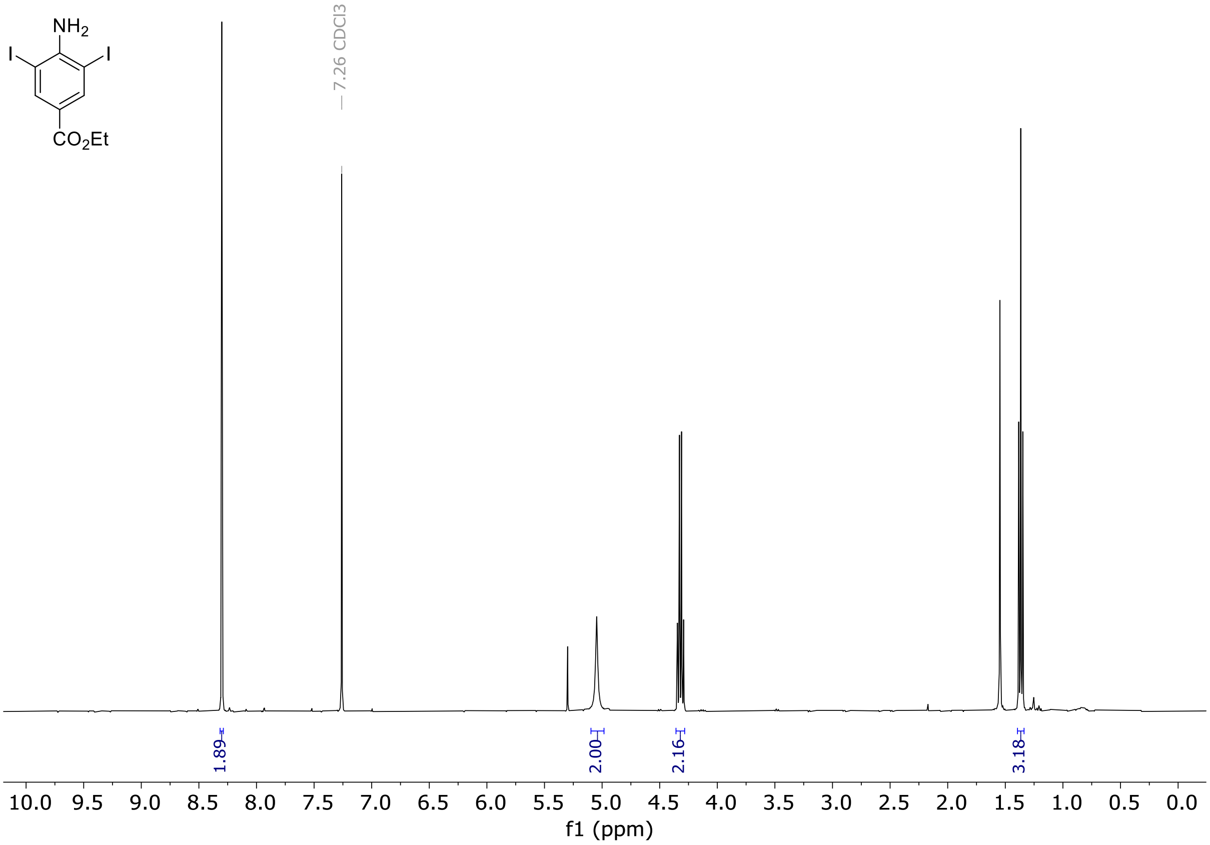

Figure S44. ^{1}H NMR Spectrum of **S30** (Chloroform-d, 298 K).

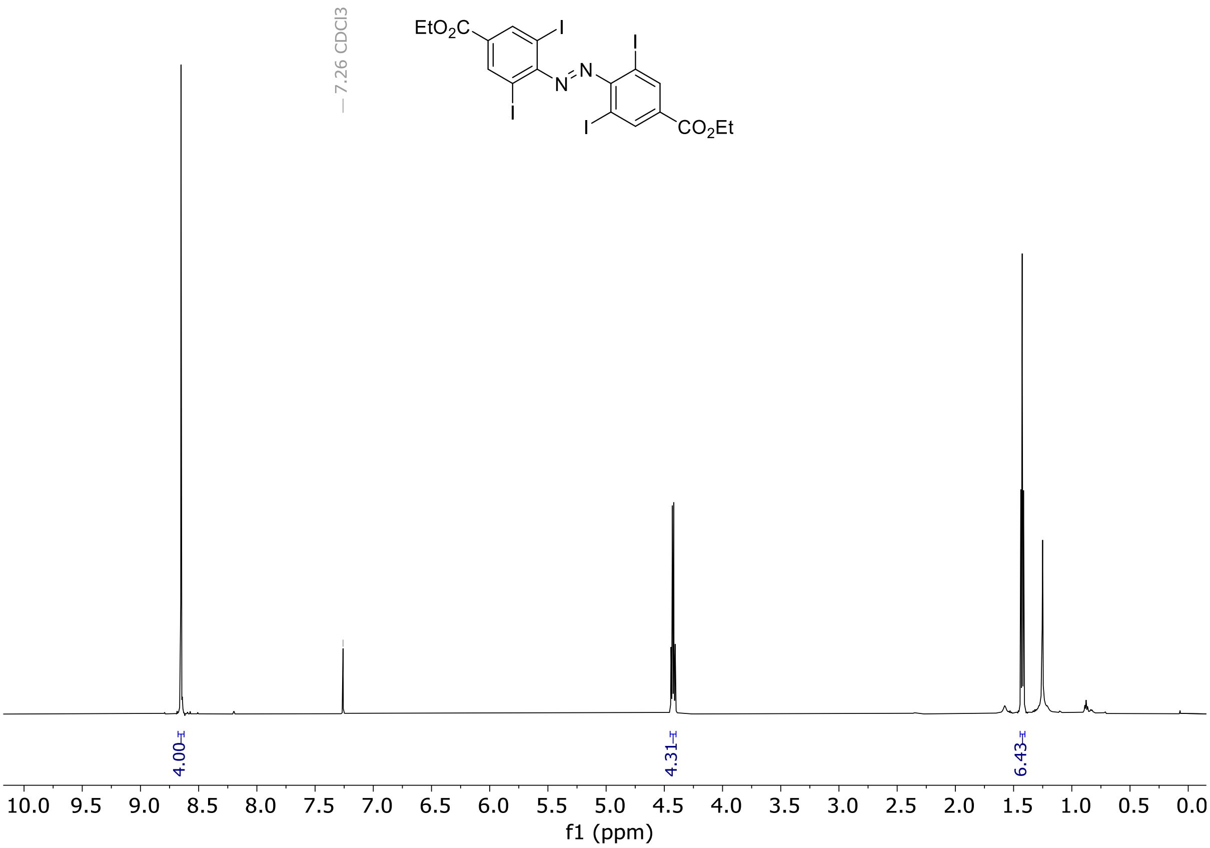

Figure S45. ^{1}H NMR Spectrum of **I$_4$-ester** (Chloroform-*d*, 298 K).

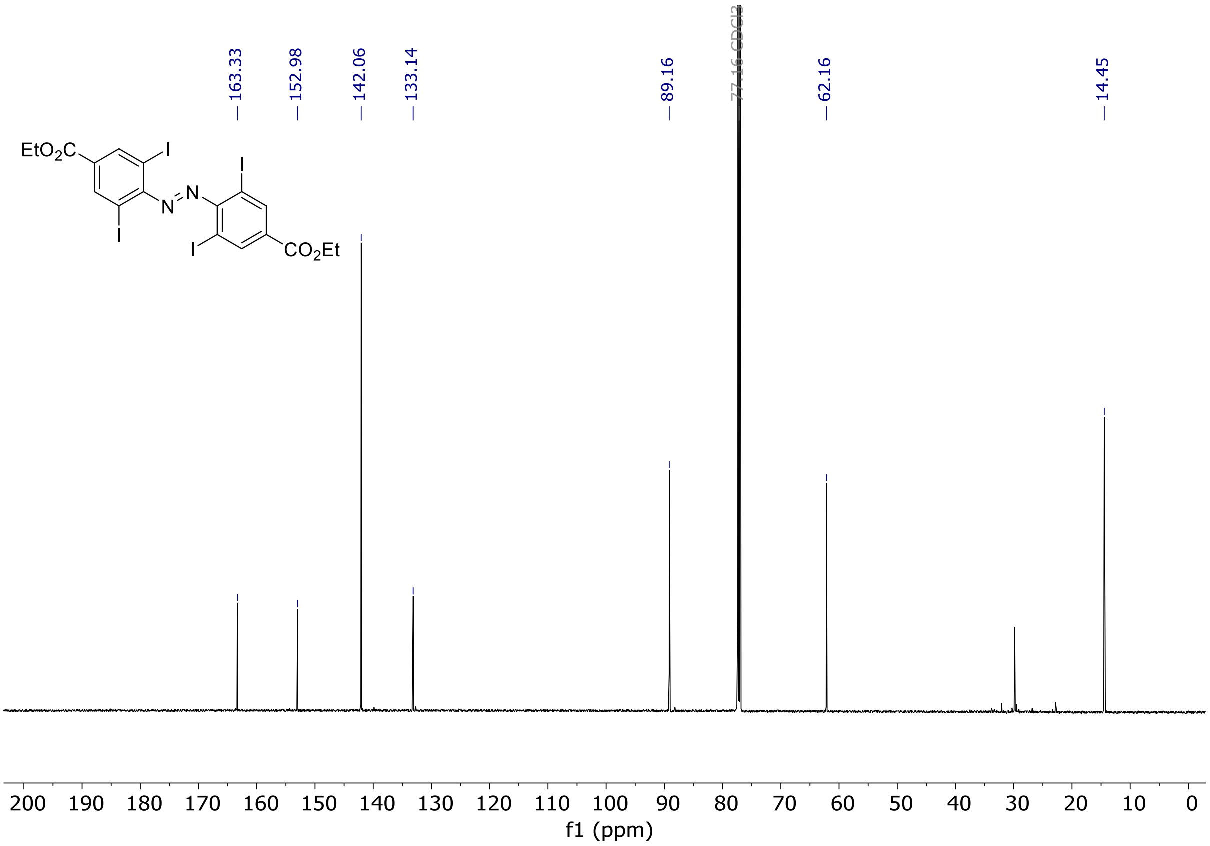

Figure S46. ^{13}C NMR Spectrum of **I$_4$-ester** (Chloroform-*d*, 298 K).

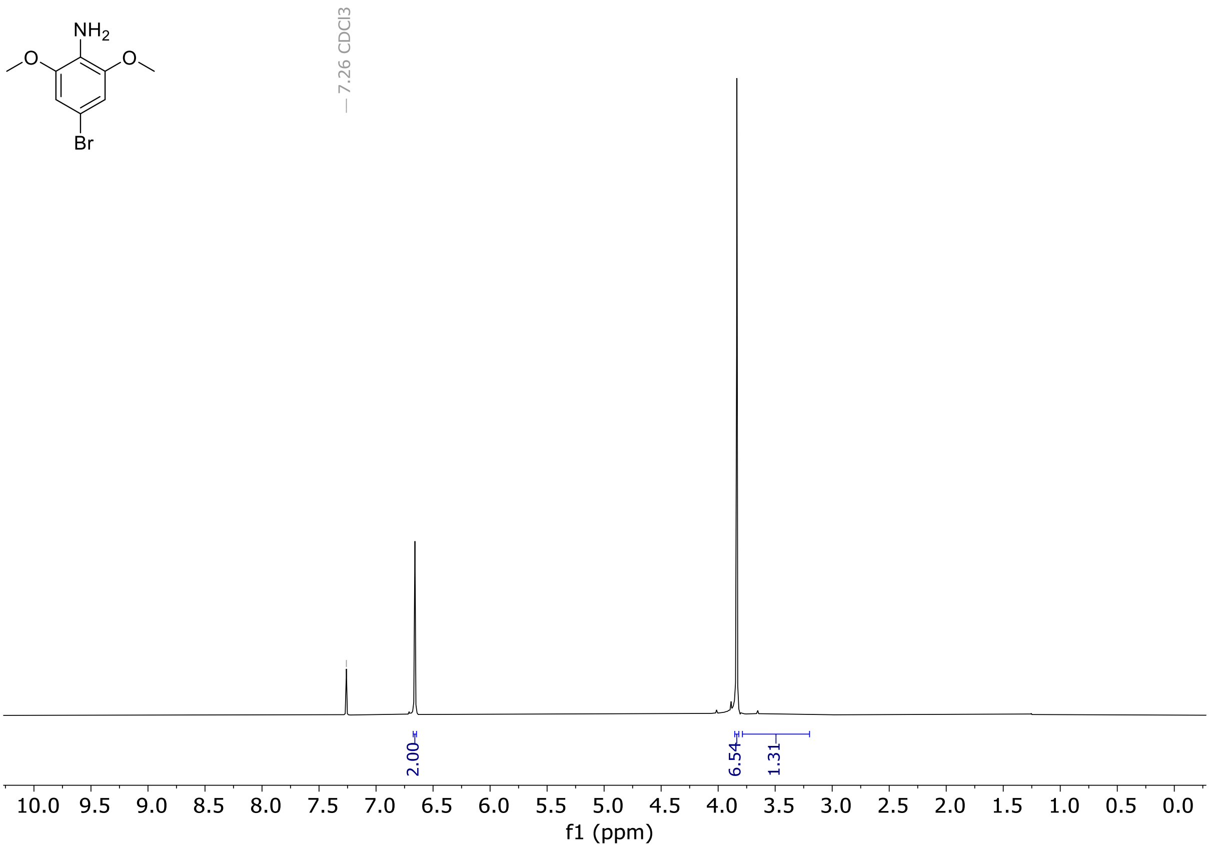

Figure S47. ^{1}H NMR Spectrum of **S32** (Chloroform-*d*, 298 K).

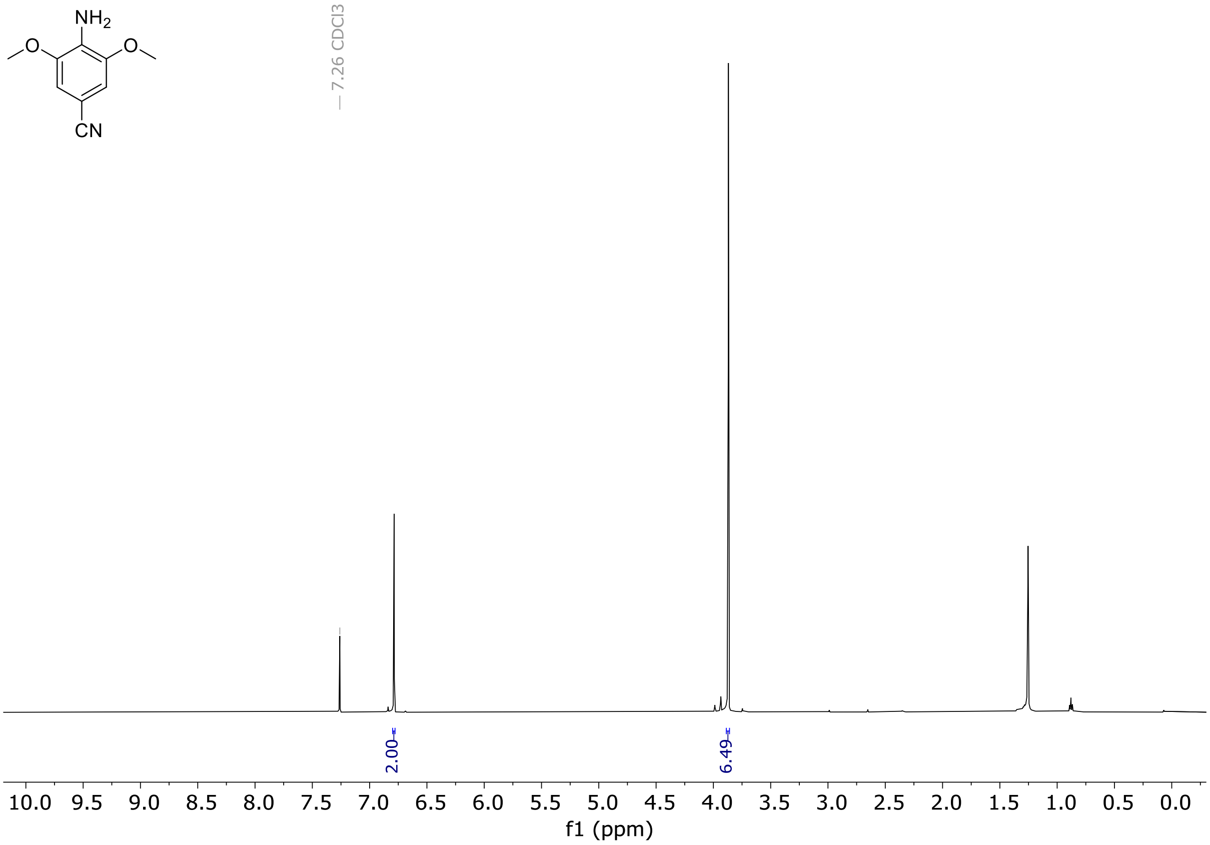

Figure S48. ^{1}H NMR Spectrum of **S33** (Chloroform-*d*, 298 K).

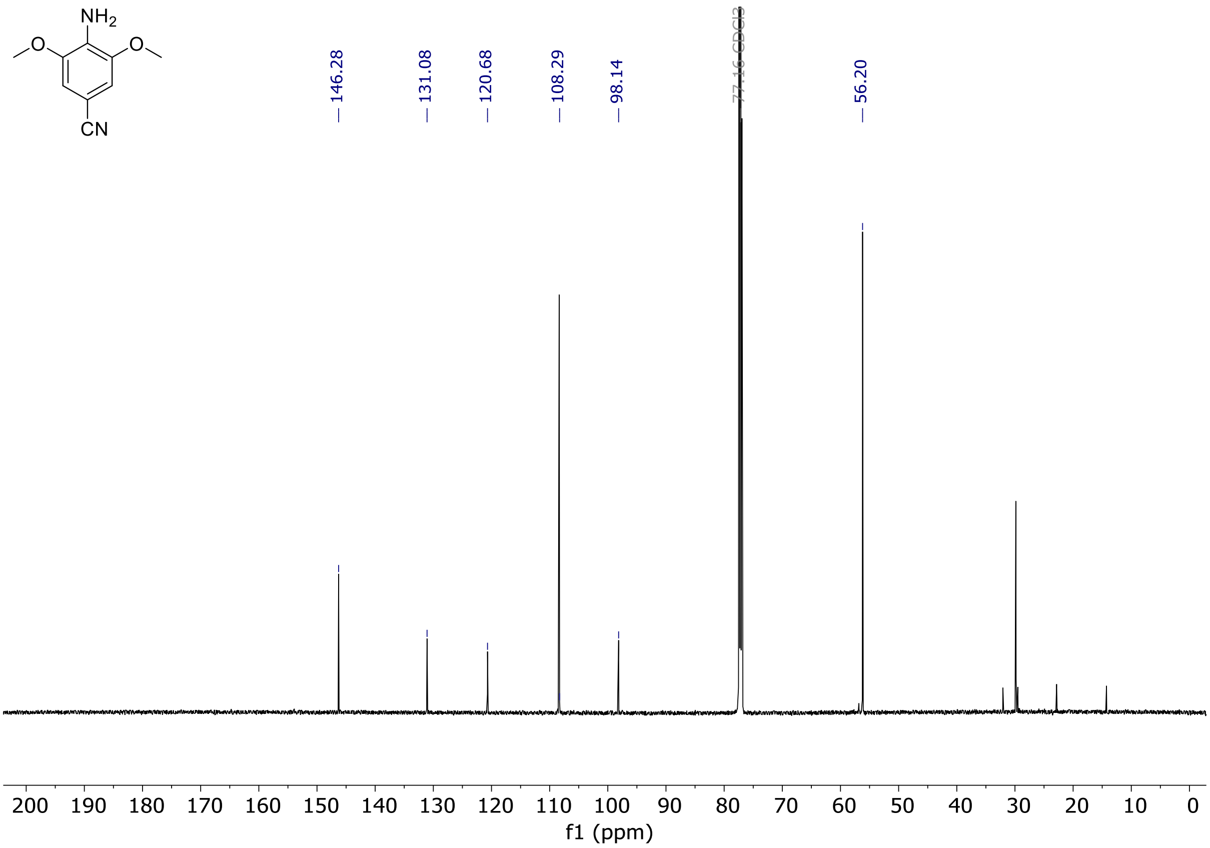

Figure S49. ^{13}C NMR Spectrum of **S33** (Chloroform-*d*, 298 K).

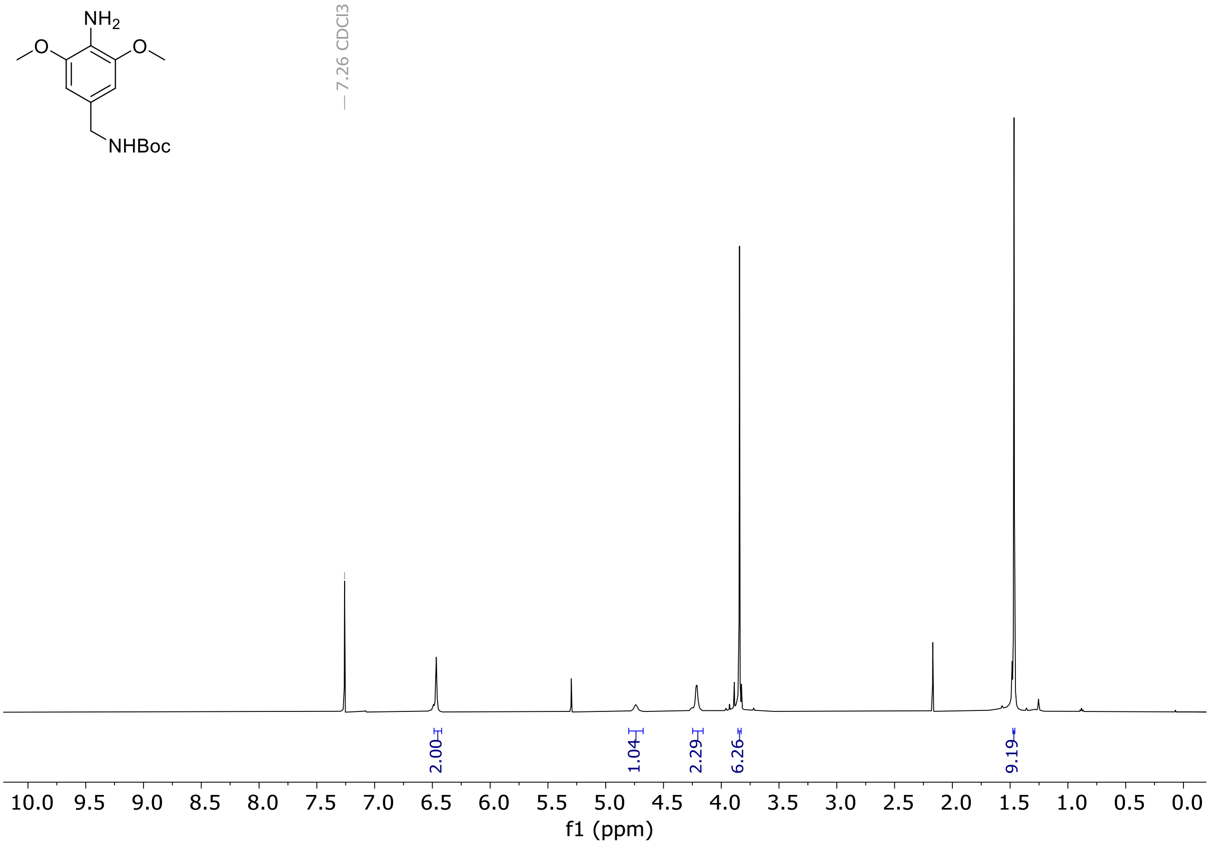

Figure S50. ^{1}H NMR Spectrum of **S35** (Chloroform-*d*, 298 K).

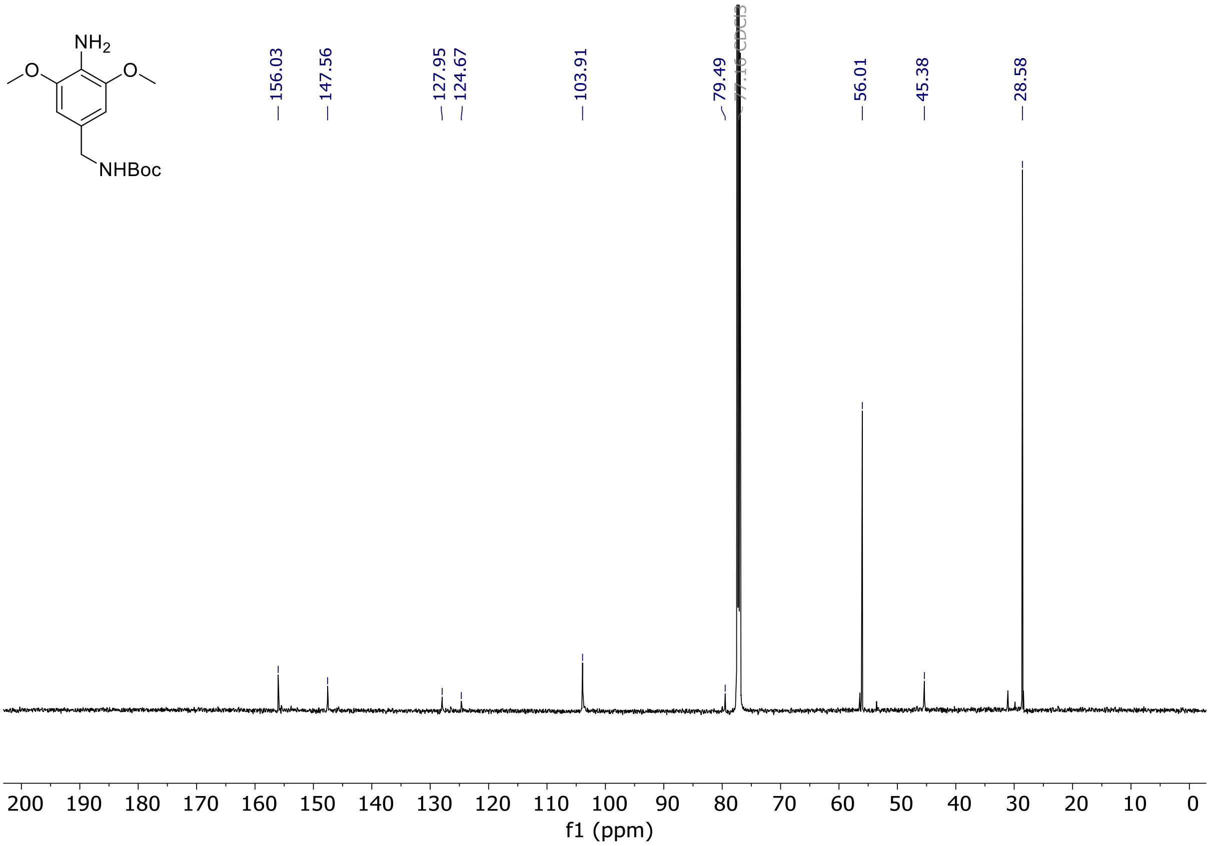

Figure S51. ^{13}C NMR Spectrum of **S35** (Chloroform-*d*, 298 K).

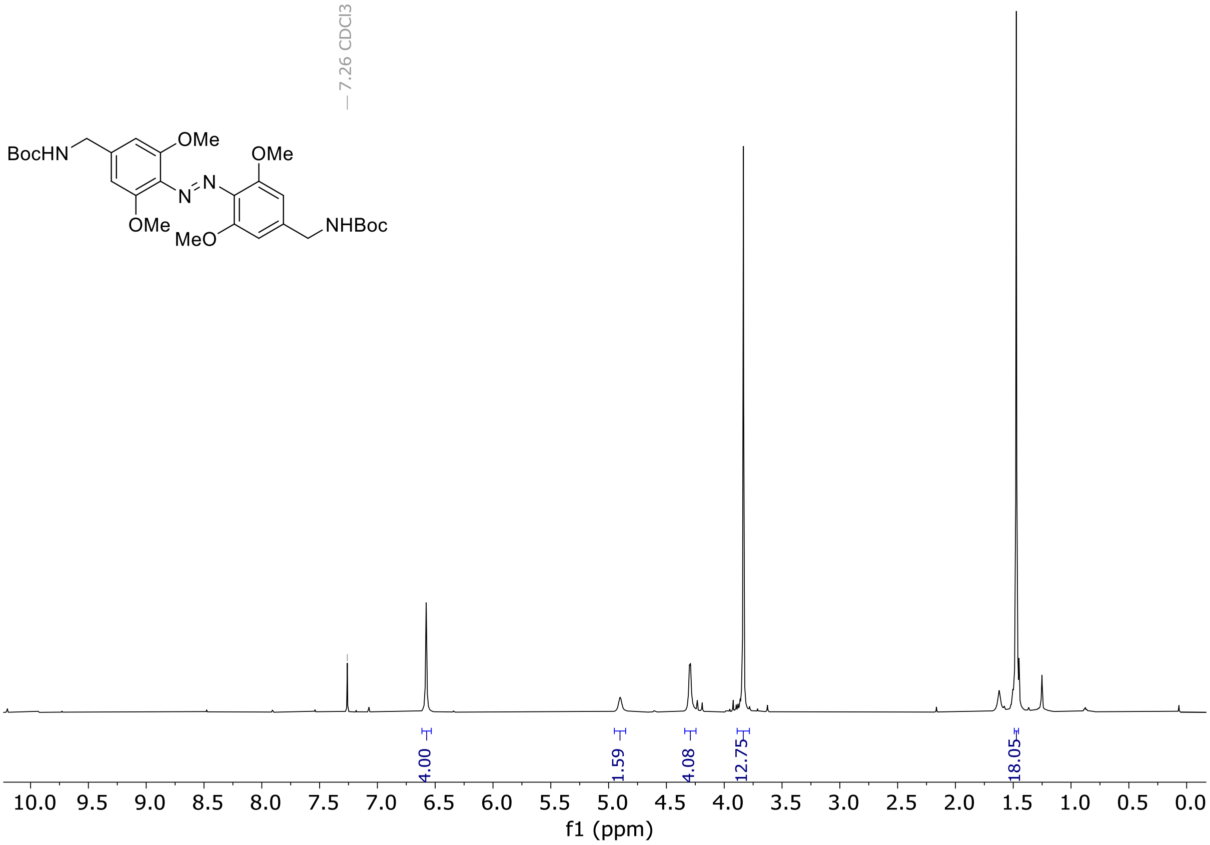

Figure S52. ^{1}H NMR Spectrum of **(OMe)$_4$-NHBoc** (Chloroform-*d*, 298 K).

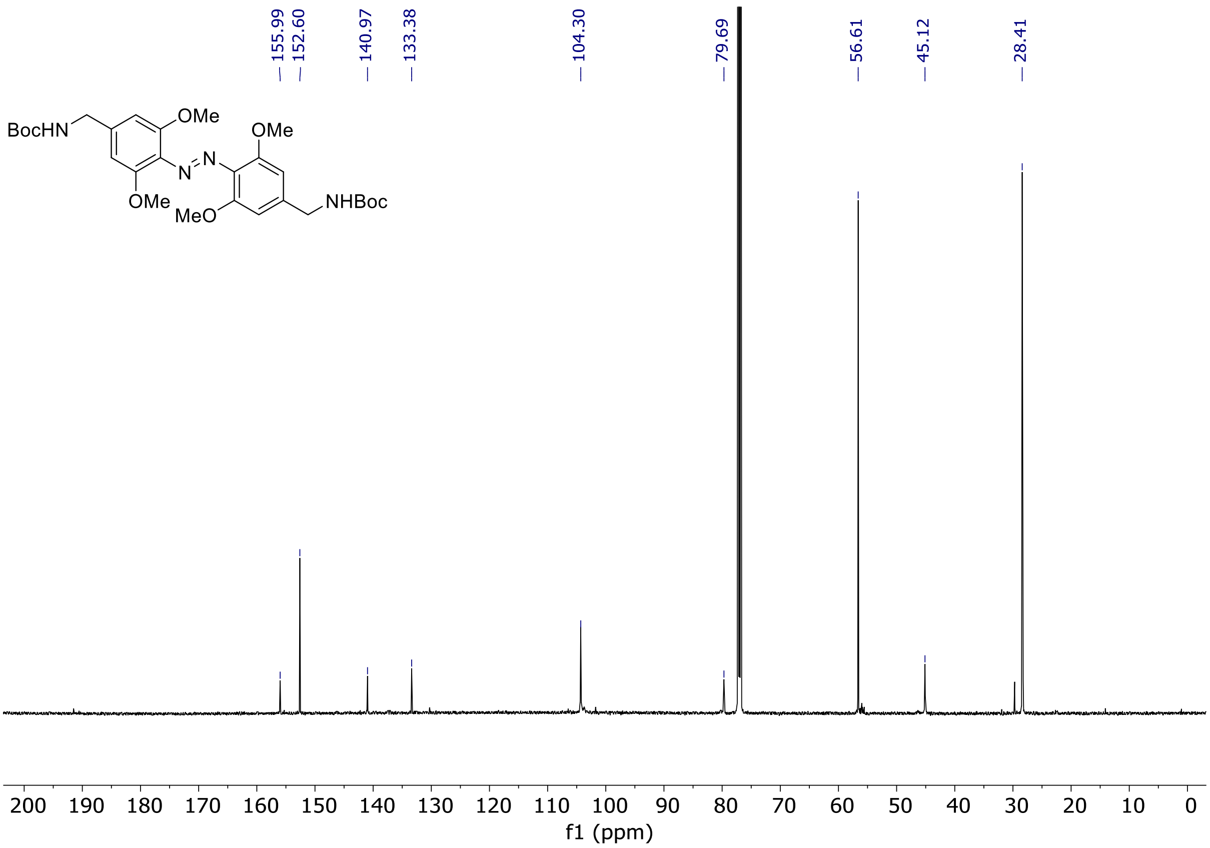

Figure S53. ^{13}C NMR Spectrum of **(OMe)$_4$-NHBoc** (Chloroform-*d*, 298 K).

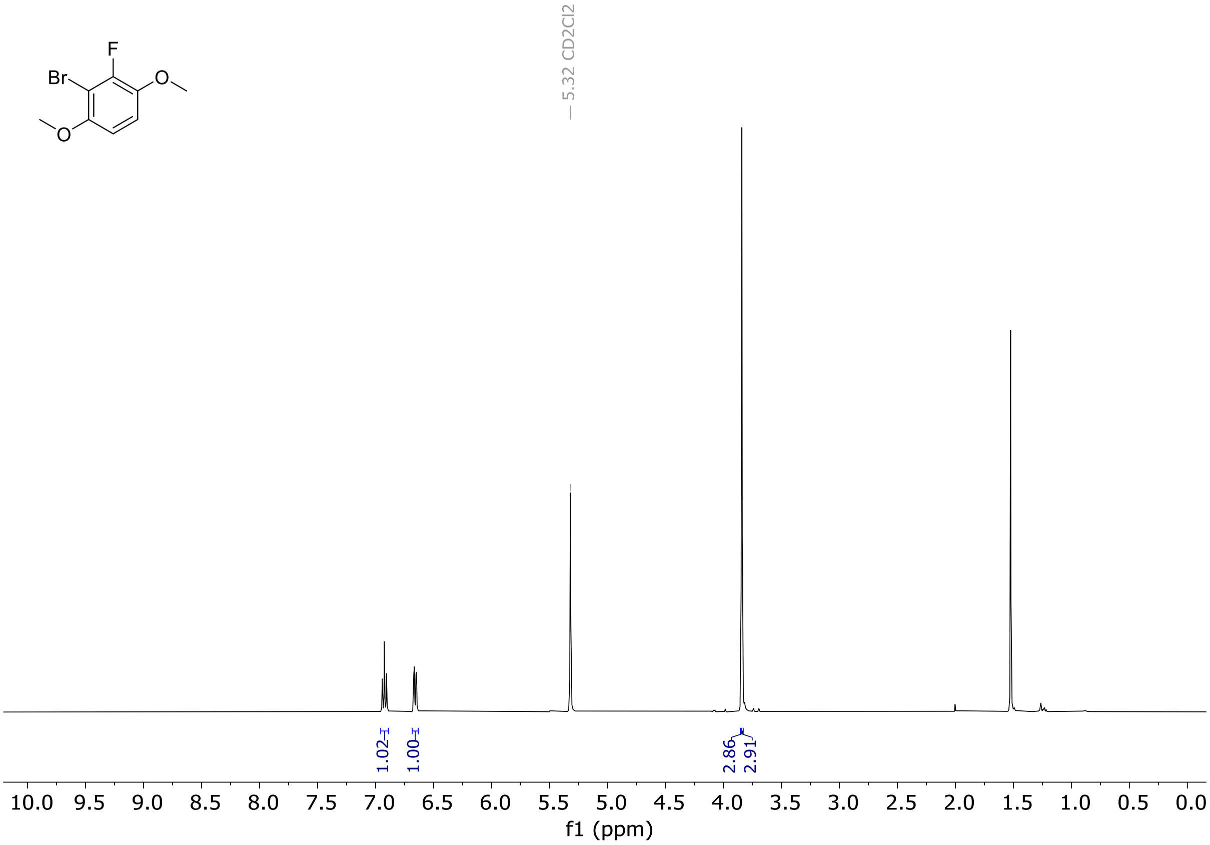

Figure S54. ^{1}H NMR Spectrum of **S37** (CD_2Cl_2, 298 K).

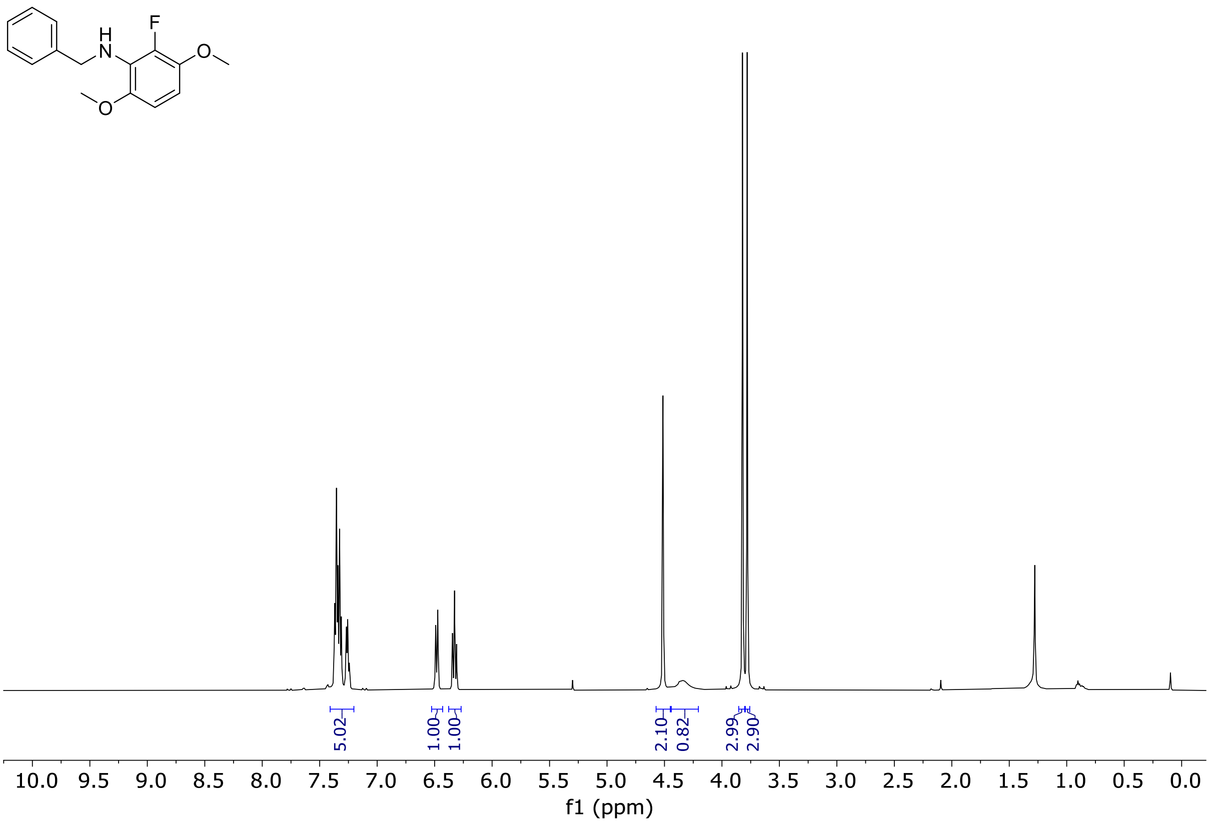

Figure S55. ^{1}H NMR Spectrum of **S38** (Chloroform-*d*, 298 K).

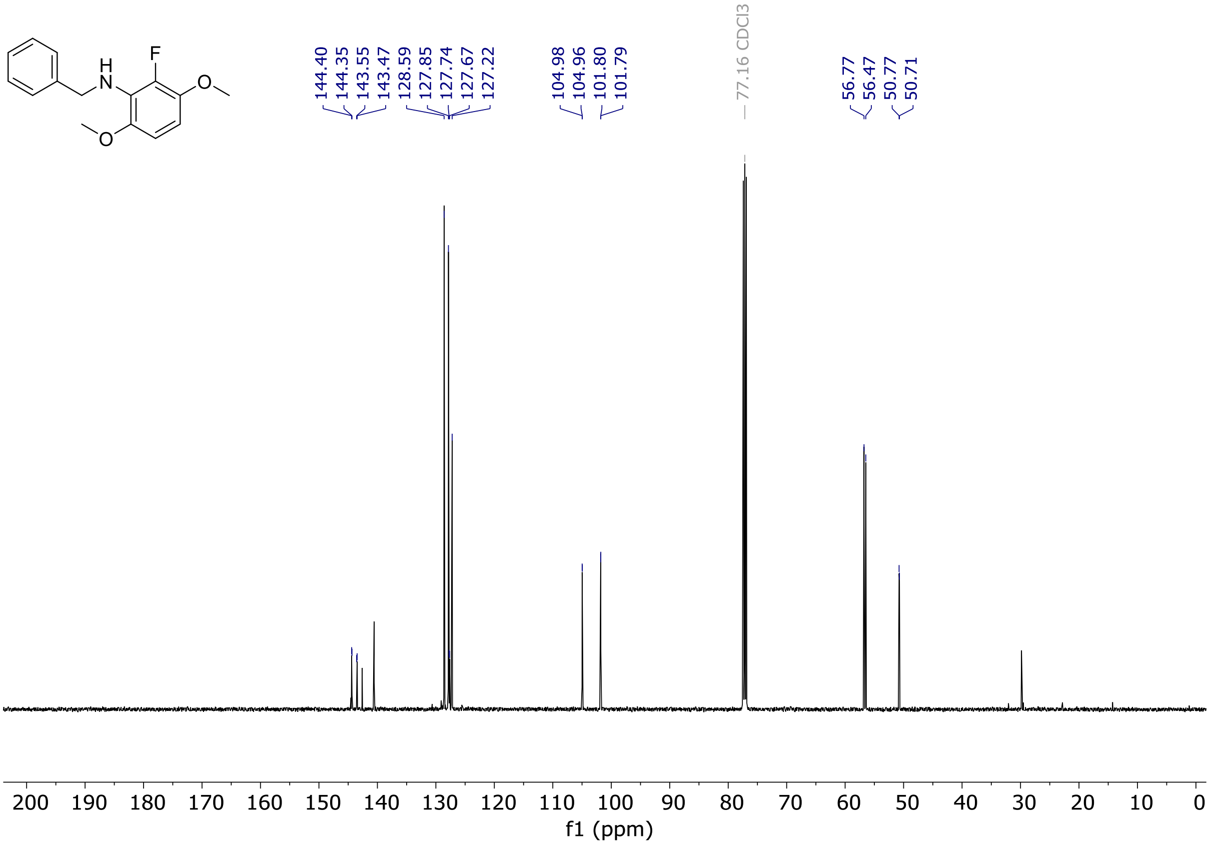

Figure S56. ^{13}C NMR Spectrum of **S38** (Chloroform-*d*, 298 K).

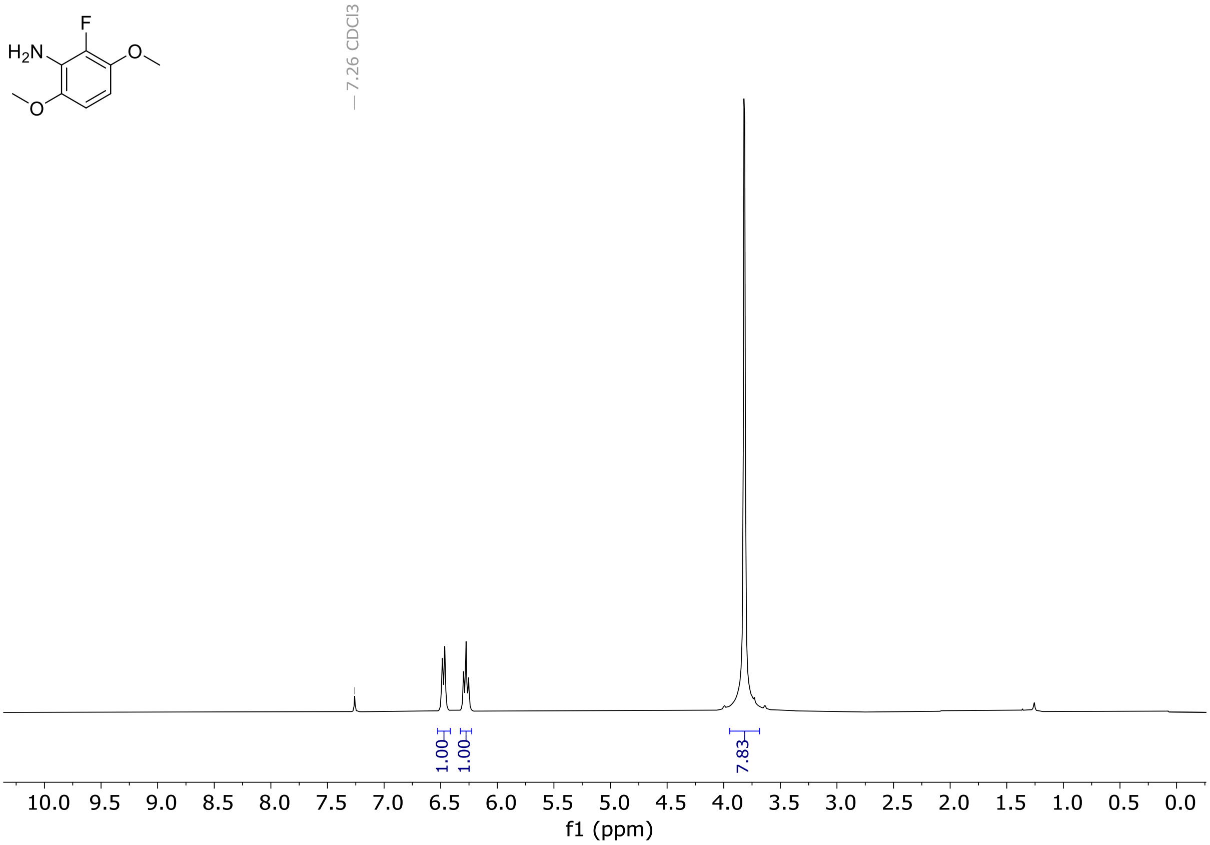

Figure S57. ^{1}H NMR Spectrum of **S39** (Chloroform-*d*, 298 K).

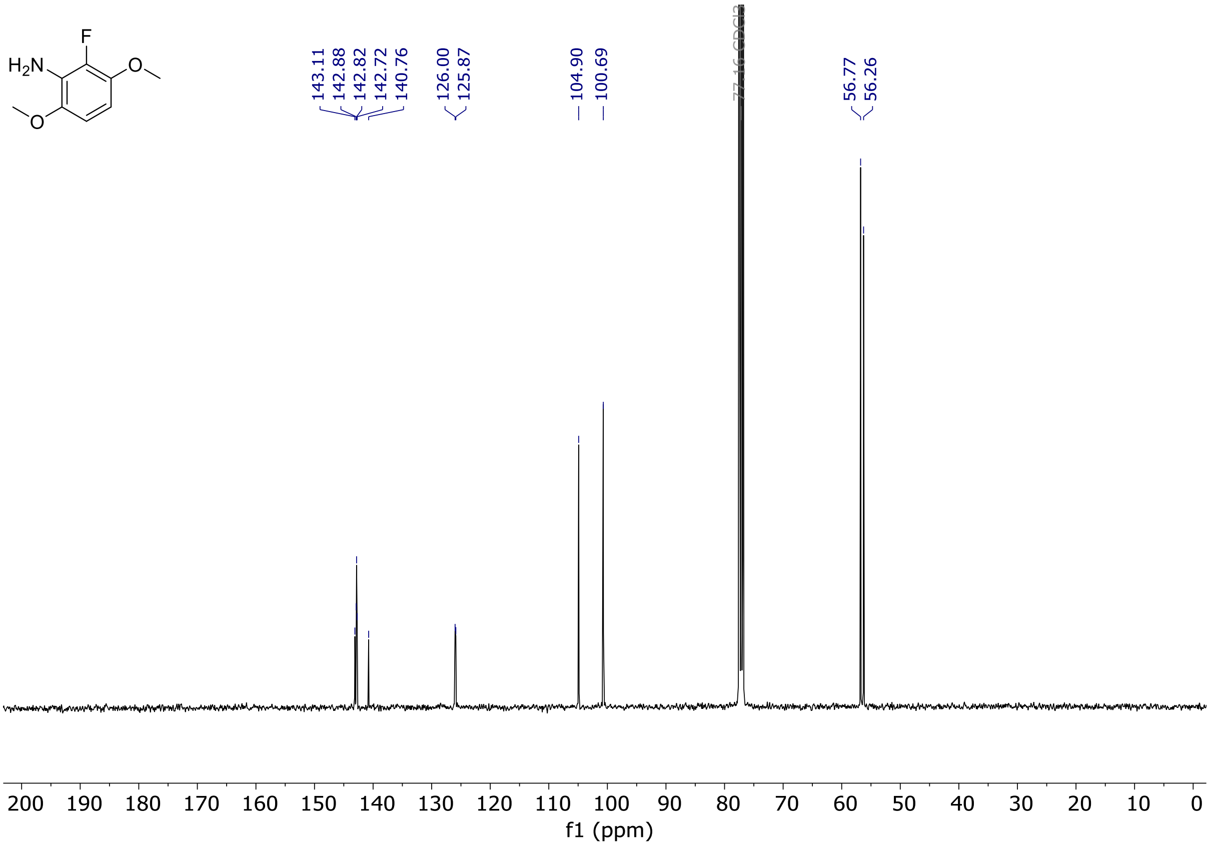

Figure S58. ^{13}C NMR Spectrum of **S39** (Chloroform-*d*, 298 K).

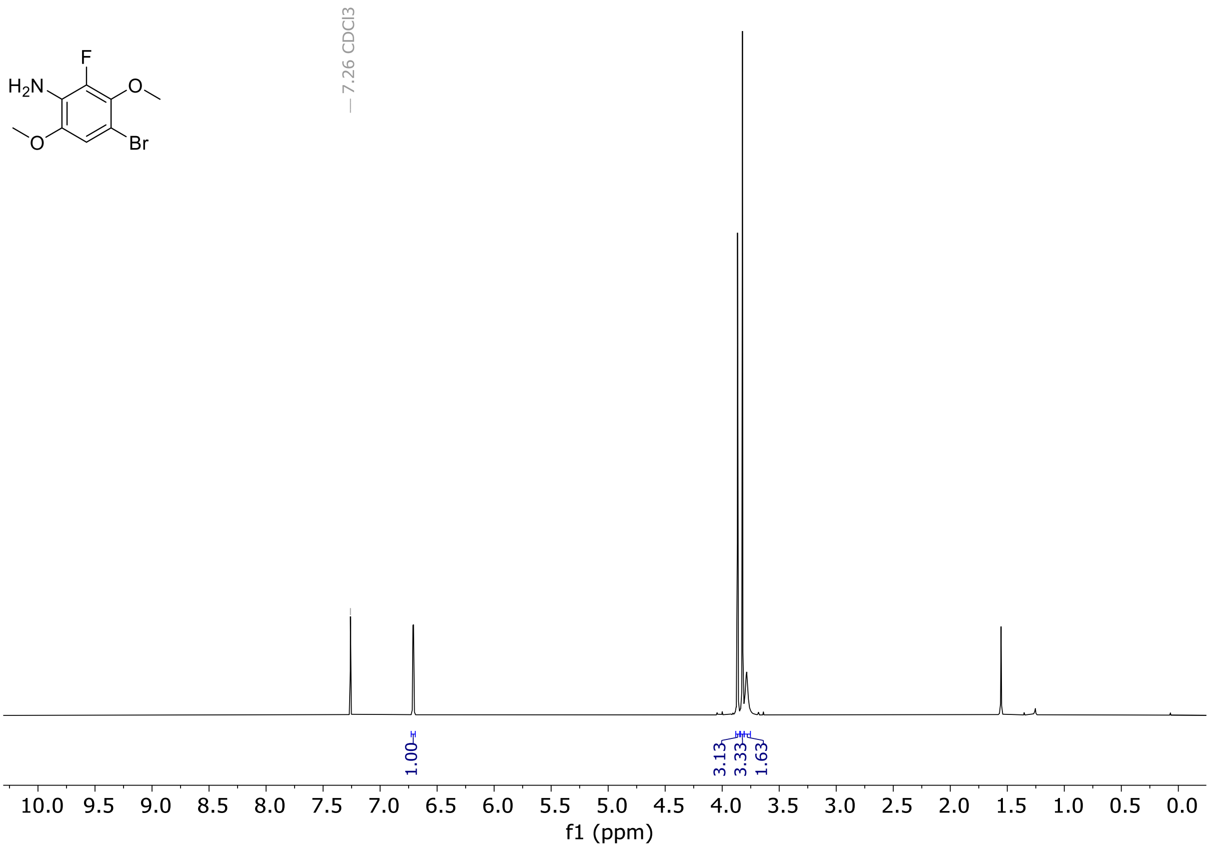

Figure S59. ^{1}H NMR Spectrum of **S40** (Chloroform-*d*, 298 K).

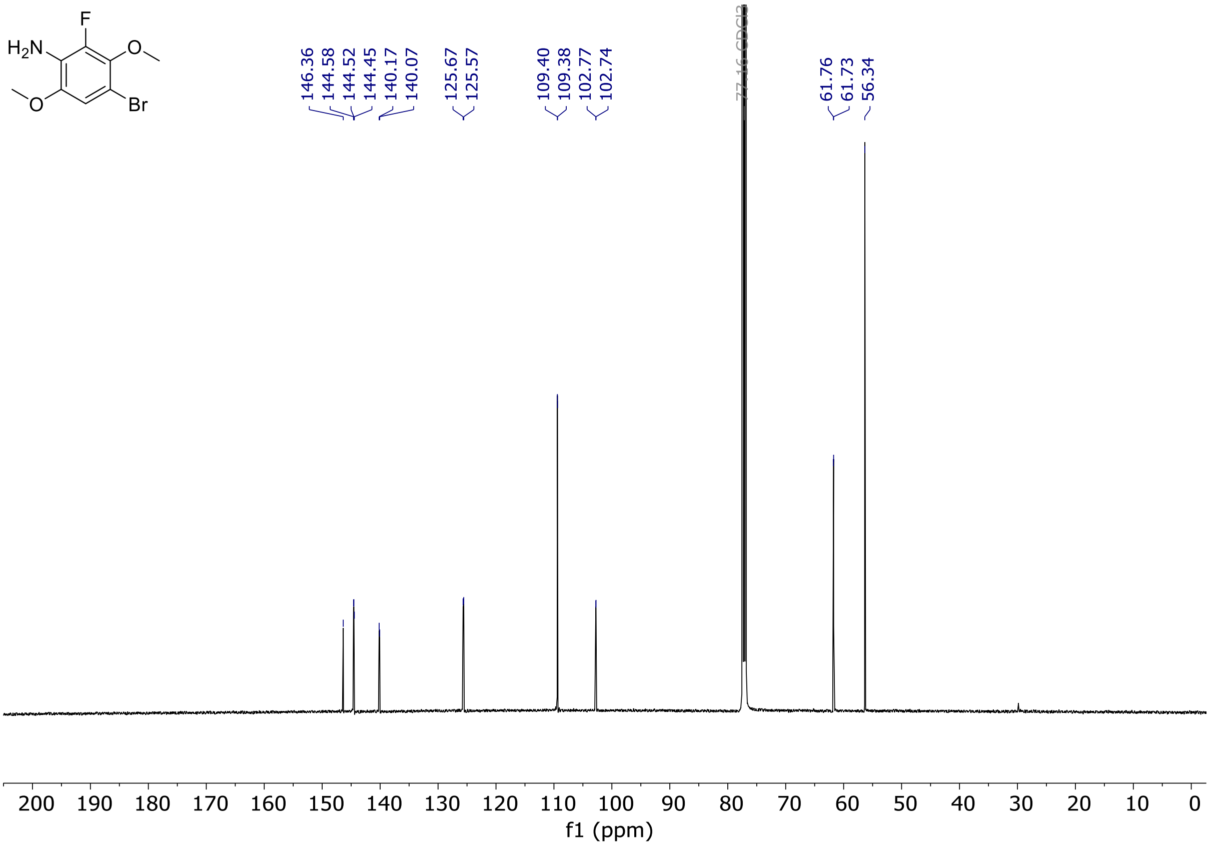

Figure S59. ^{13}C NMR Spectrum of **S40** (Chloroform-*d*, 298 K).

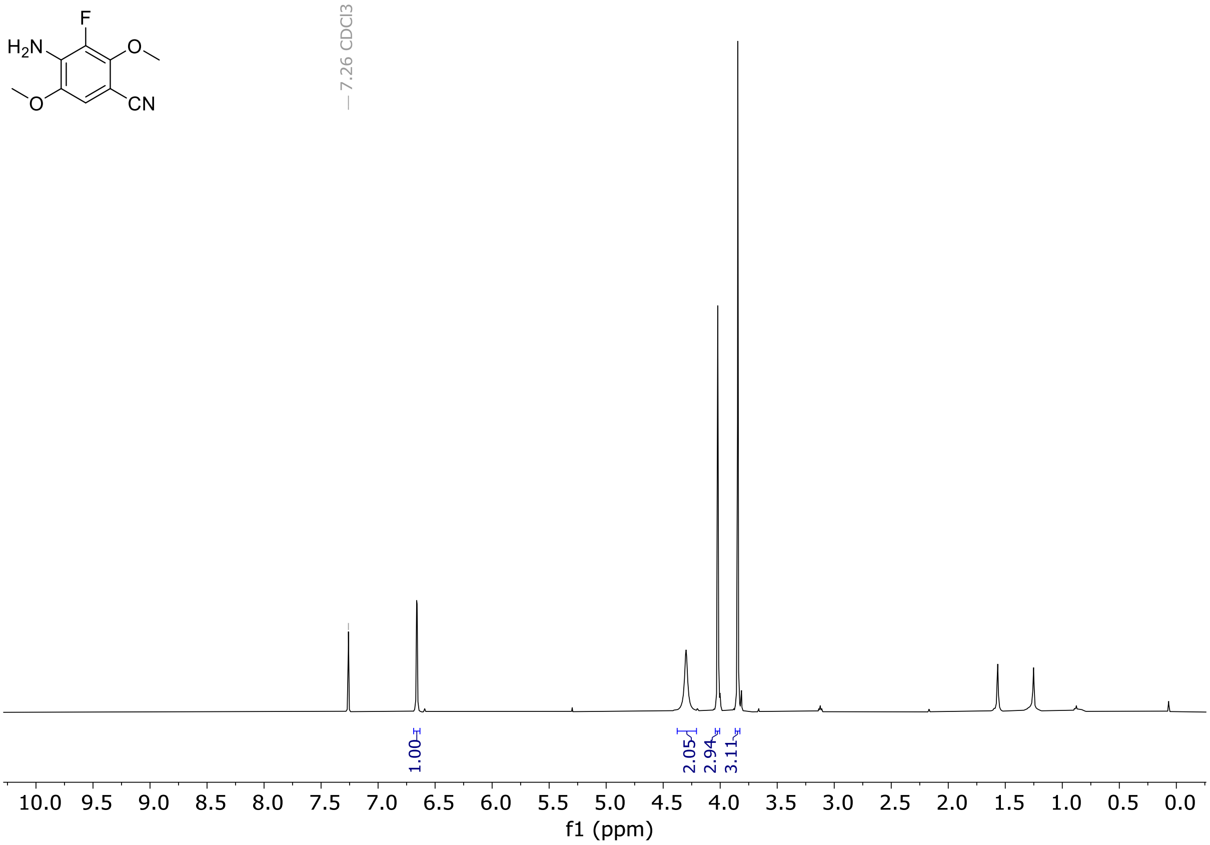

Figure S61. ^{1}H NMR Spectrum of **S41** (Chloroform-*d*, 298 K).

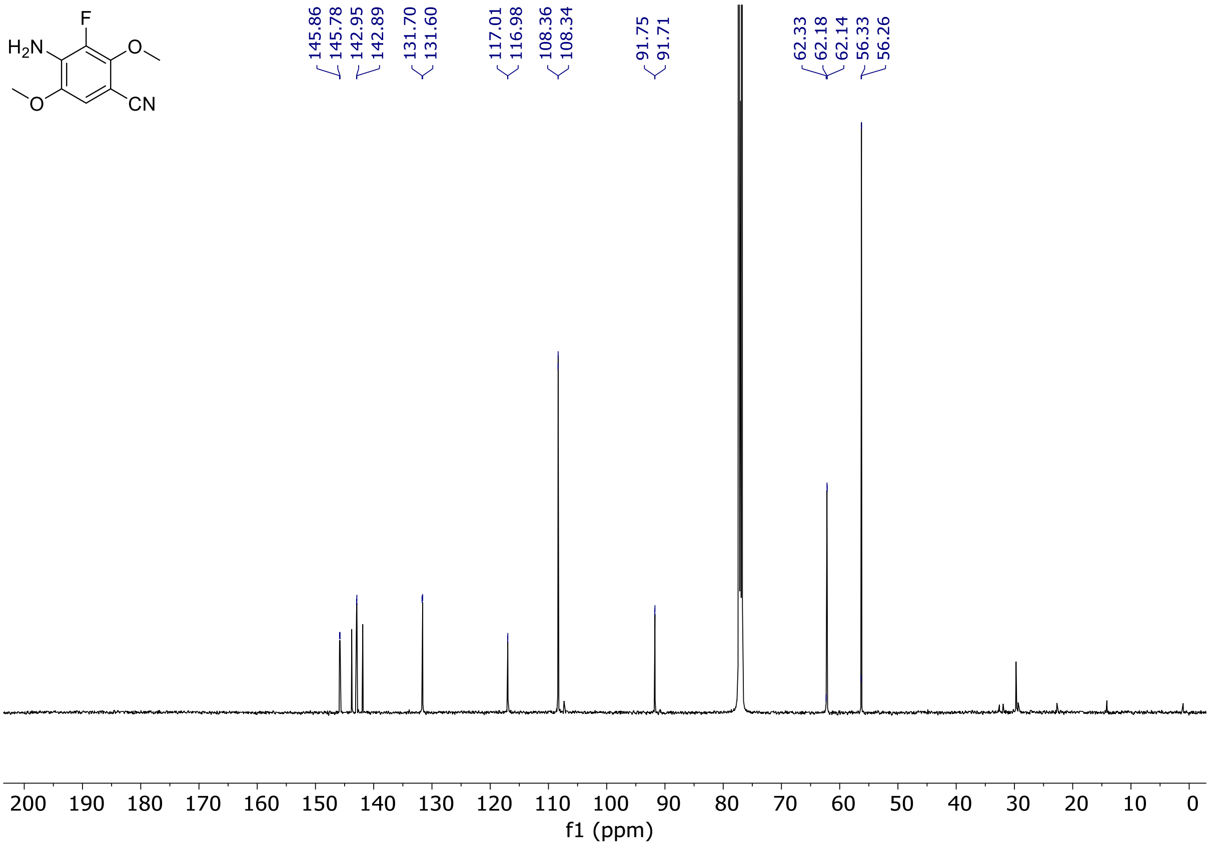

Figure S62. ^{13}C NMR Spectrum of **S41** (Chloroform-*d*, 298 K).

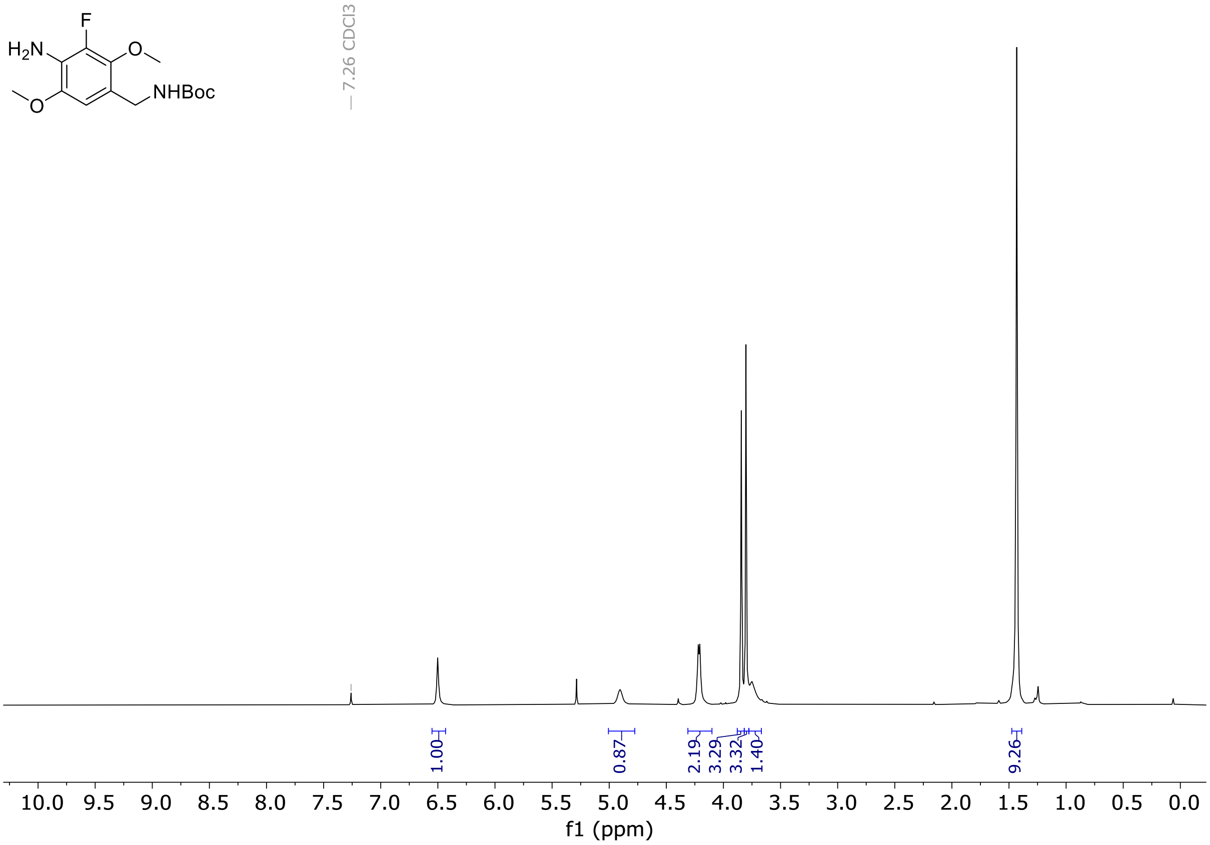

Figure S63. ^{1}H NMR Spectrum of **S42** (Chloroform-*d*, 298 K).

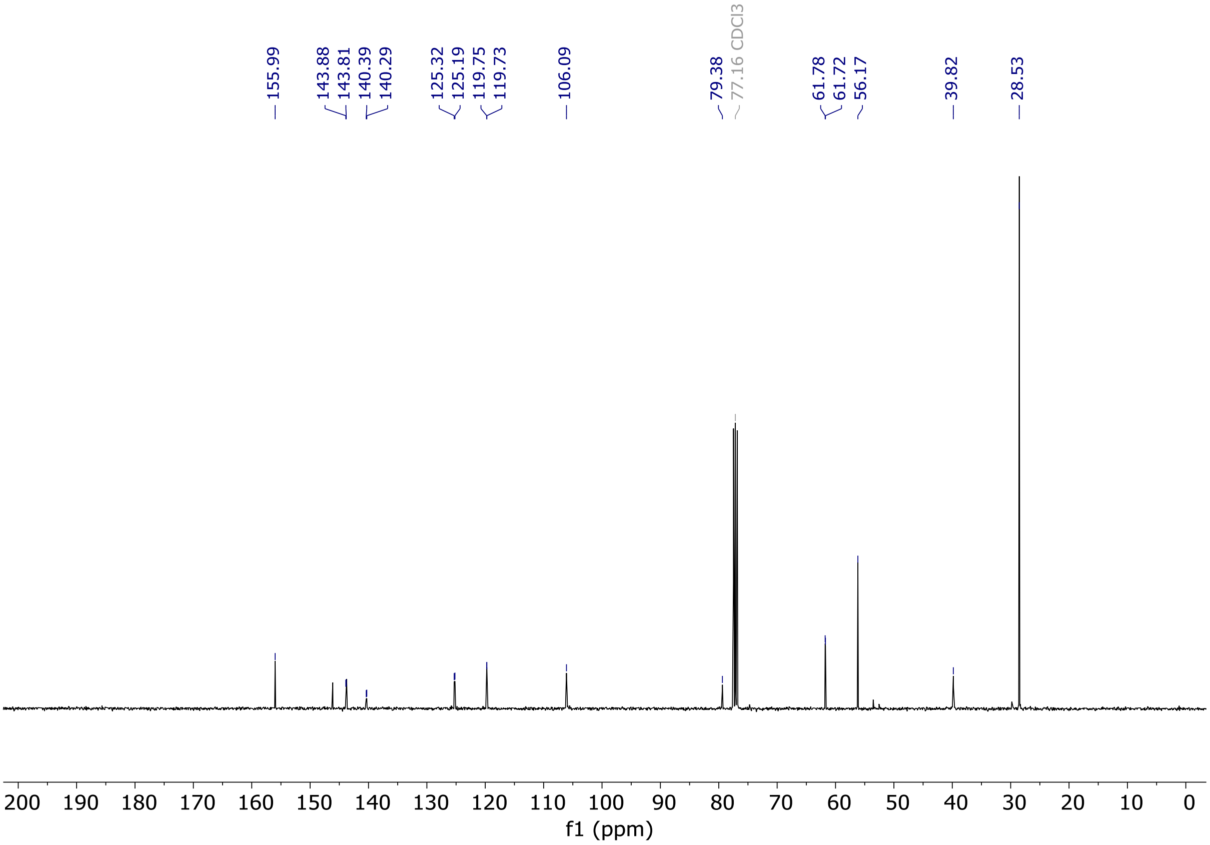

Figure S64. ^{13}C NMR Spectrum of **S42** (Chloroform-*d*, 298 K).

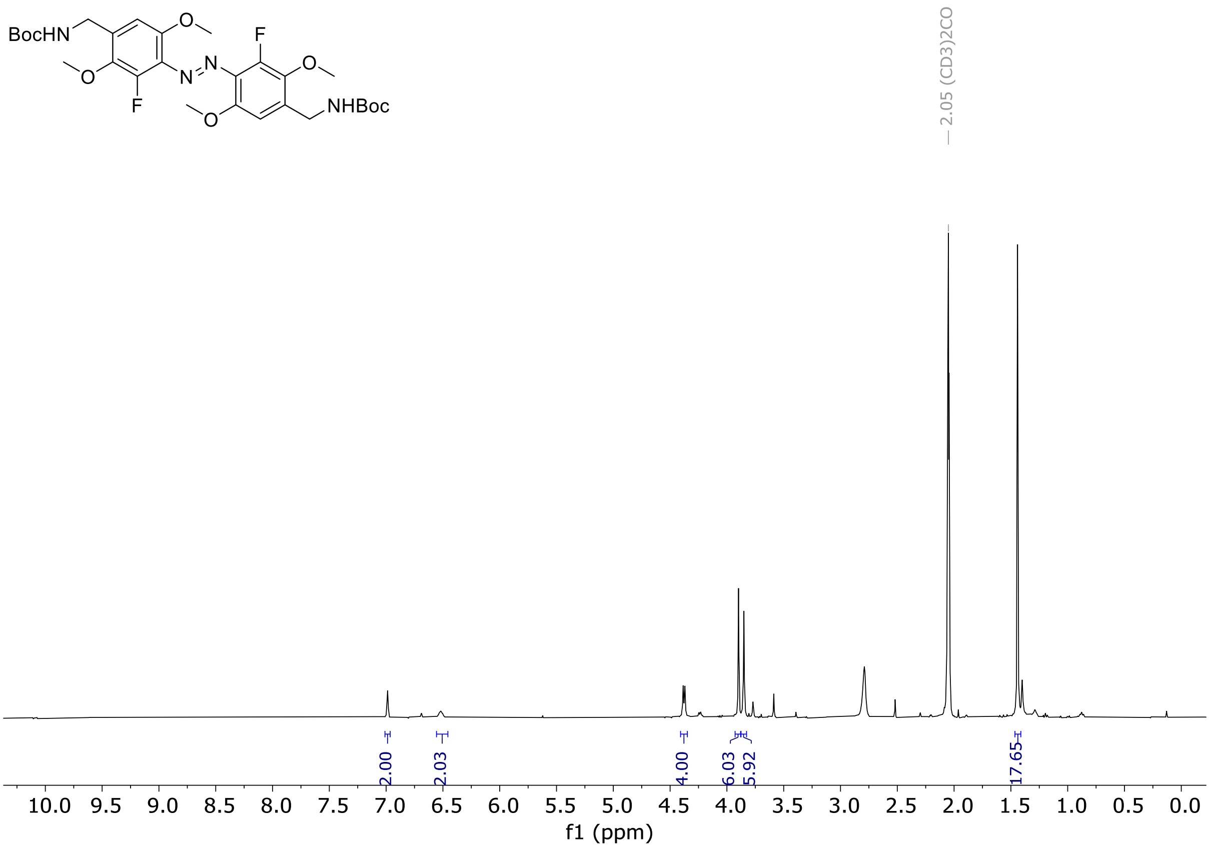

Figure S65. ^{1}H NMR Spectrum of **(OMe)$_2$F$_2$,*m*-OMe$_2$-NHBoc** (Acetone-*d*$_6$, 298 K).

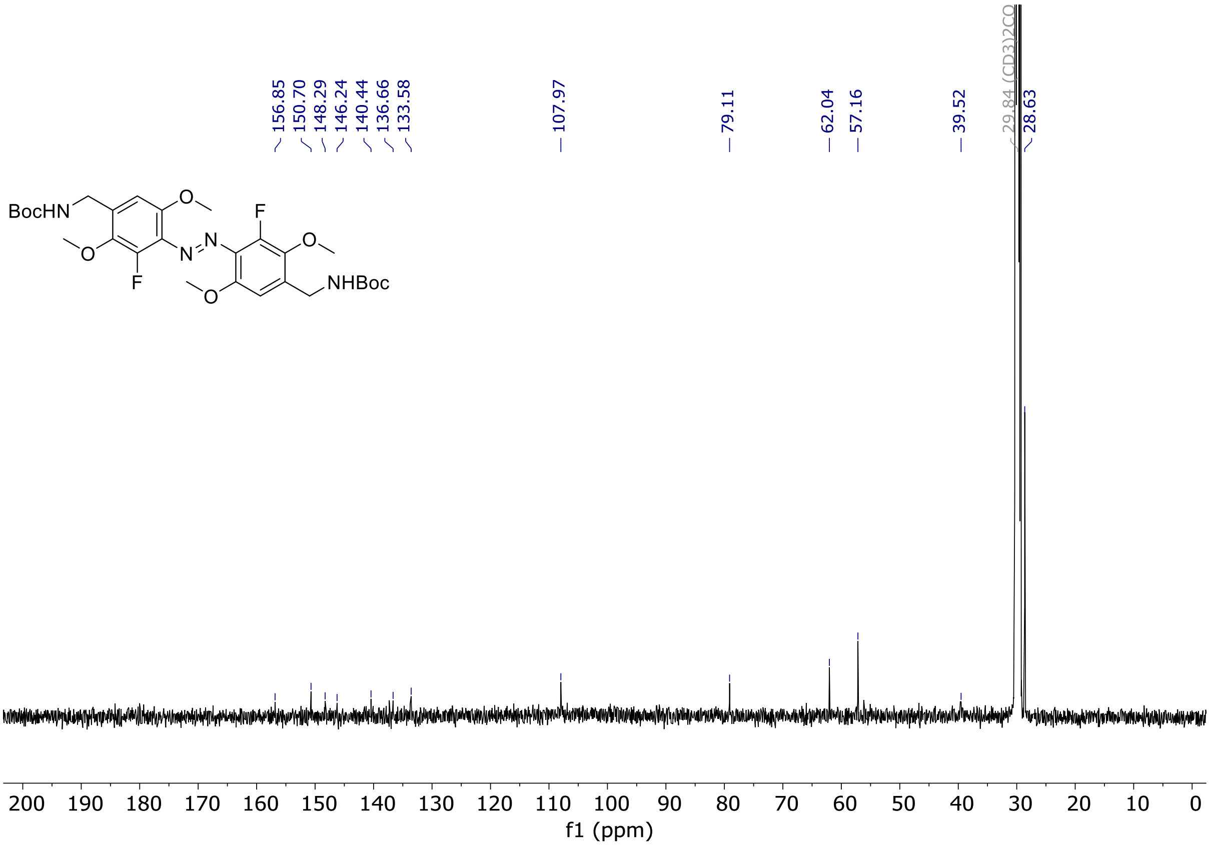

Figure S66. ^{13}C NMR Spectrum of **(OMe)$_2$F$_2$,*m*-OMe$_2$-NHBoc** (Acetone-*d*$_6$, 298 K).

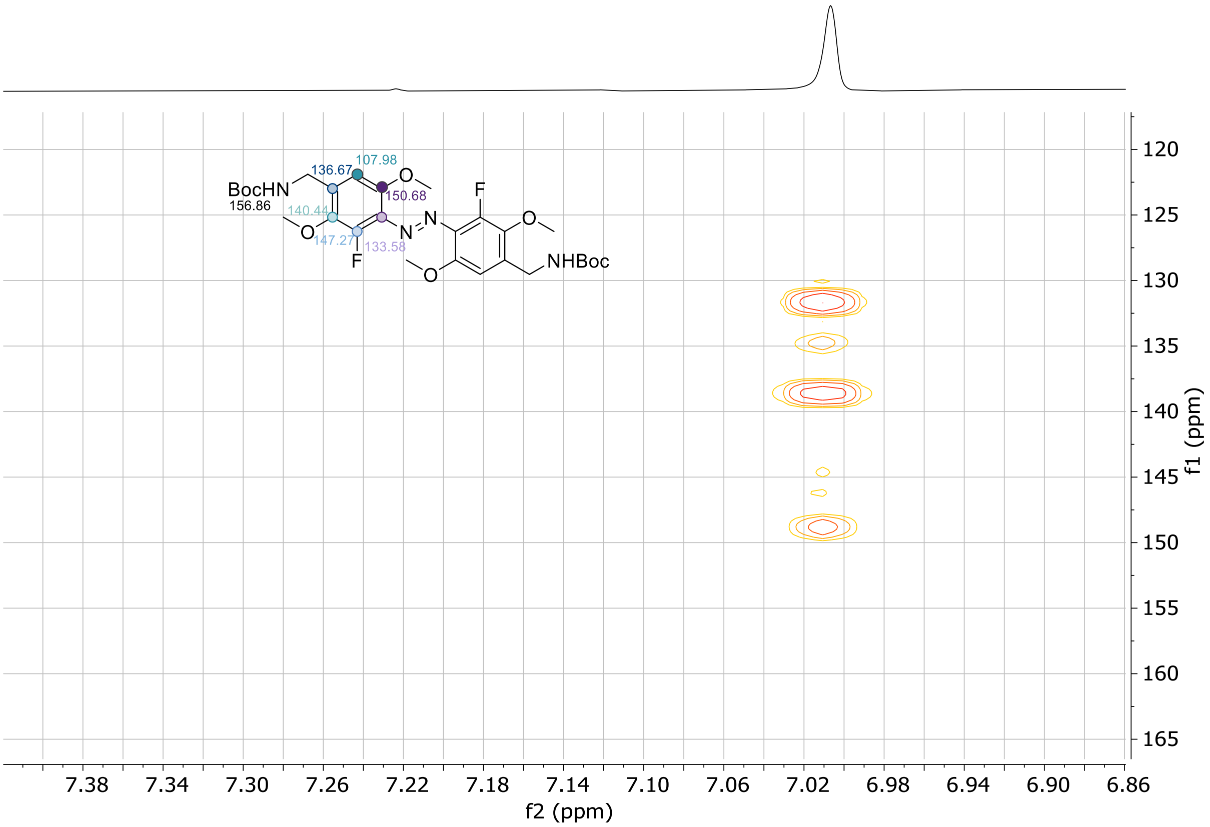

Figure S67. Partial HMBC spectrum of **(OMe)$_2$F$_2$,*m*-OMe$_2$-NHBoc** (Acetone-d_6, 298 K).

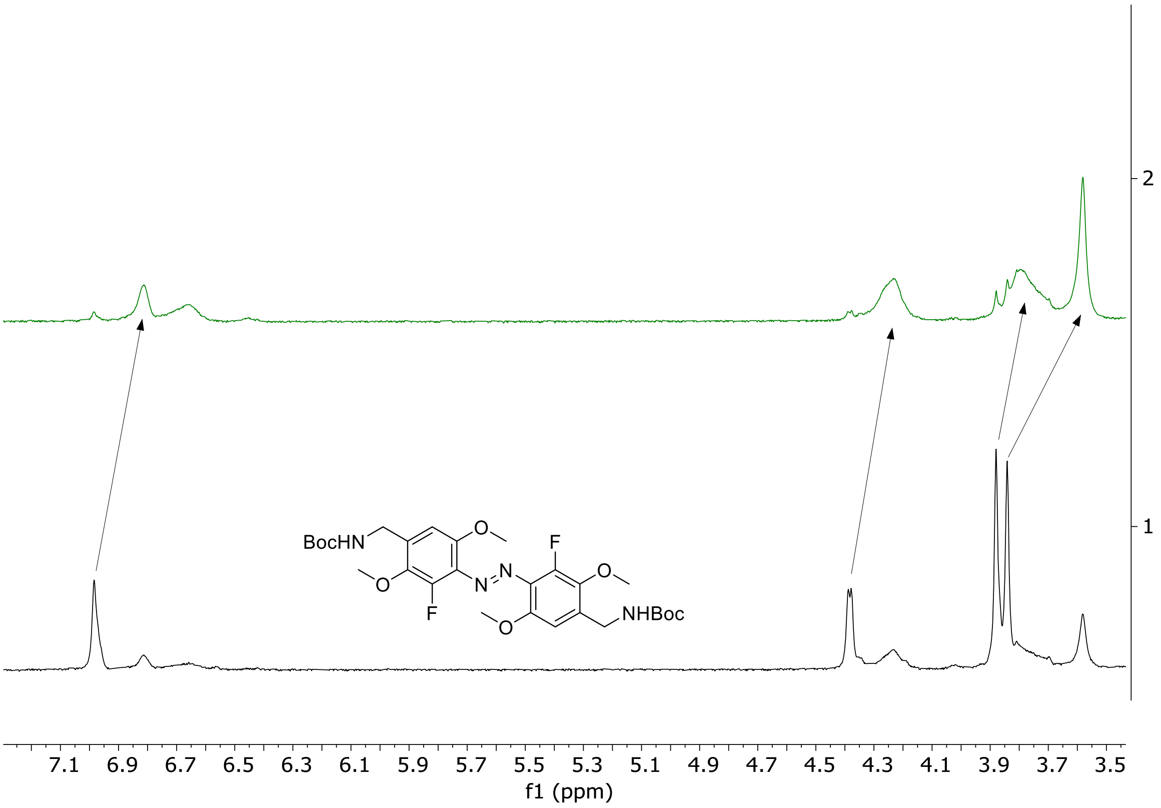

Figure S68. Stability test for **(OMe)$_2$F$_2$,*m*-OMe$_2$-NHBoc**. Bottom: Initial. Top: after irradiation with 530 nm (Acetone-d_6, 238 K)

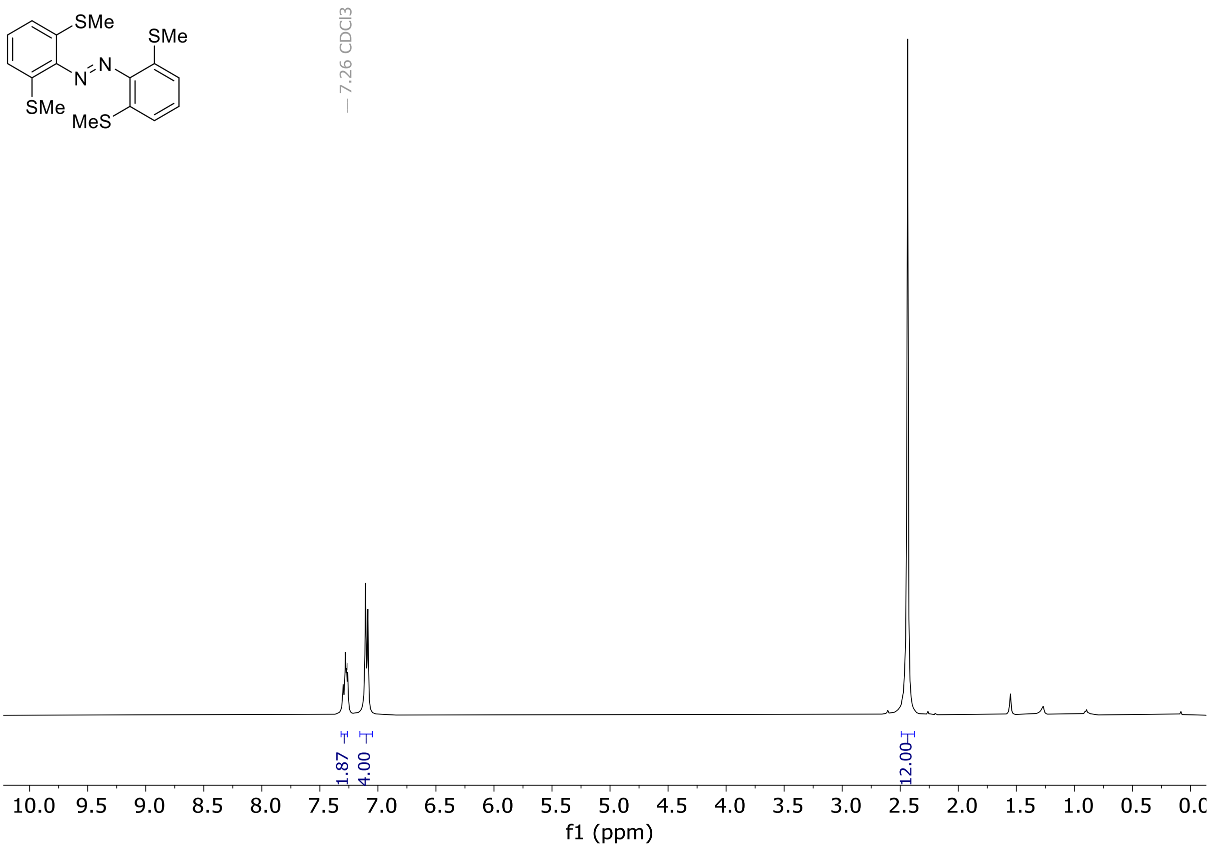

Figure S69. ^{1}H NMR Spectrum of **(SMe$_4$)-H** (Chloroform-*d*, 298 K).

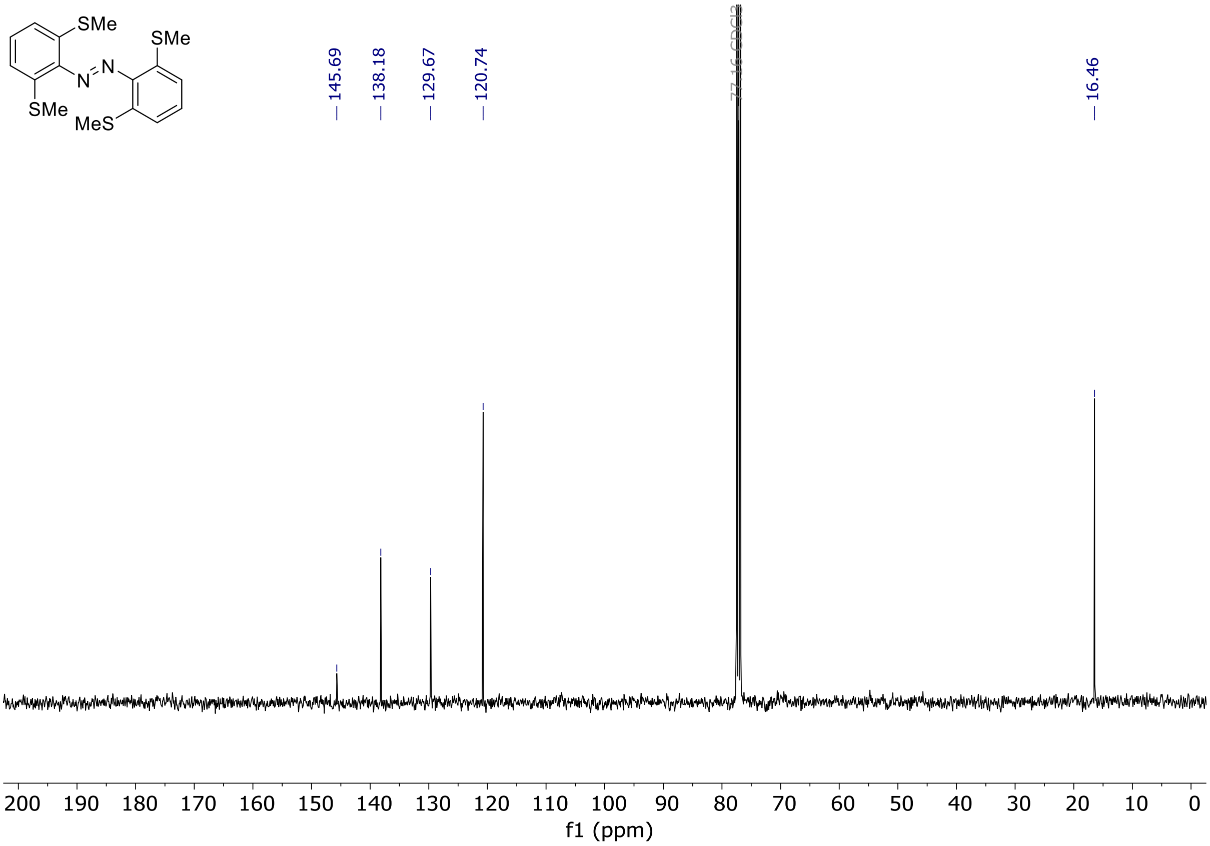

Figure S70. ^{13}C NMR Spectrum of **(SMe$_4$)-H** (Chloroform-*d*, 298 K).

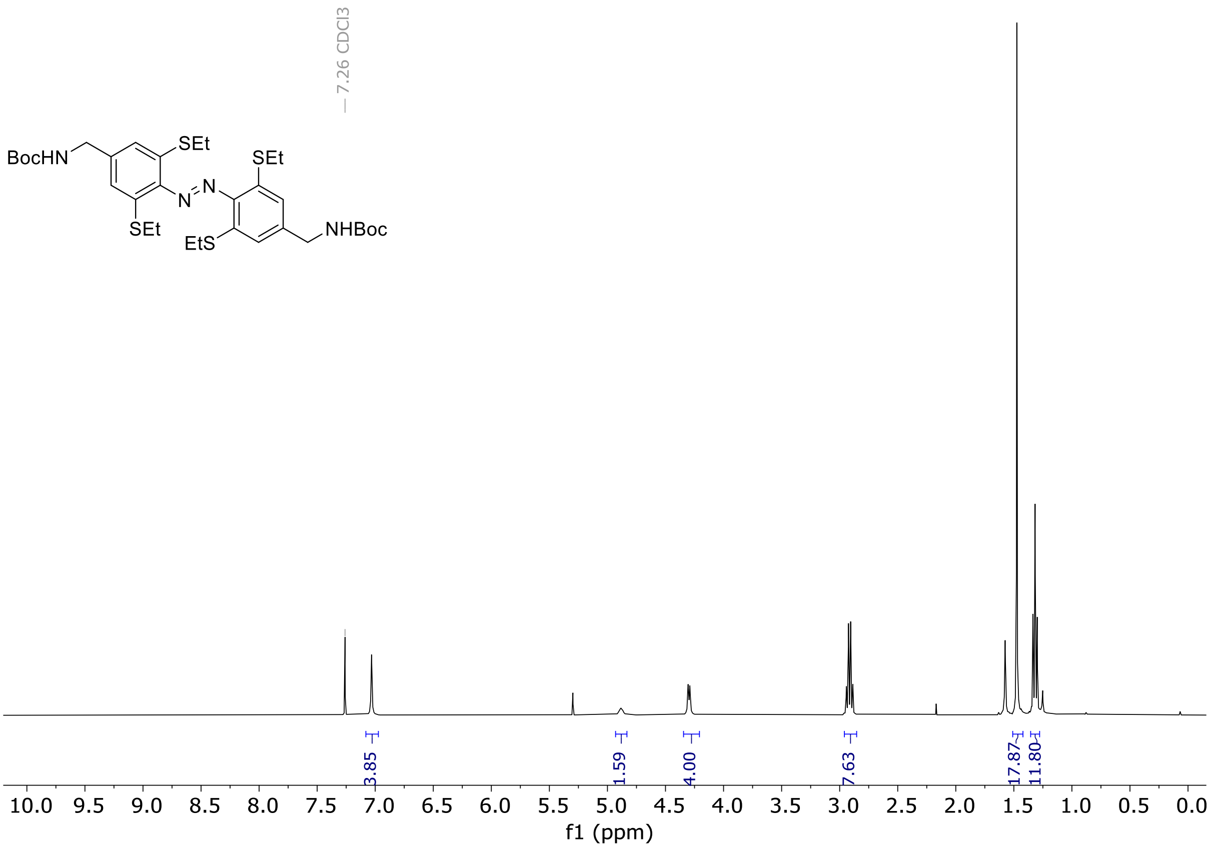

Figure S71. ^{1}H NMR Spectrum of **(SEt)$_4$-NHBoc** (Chloroform-*d*, 298 K).

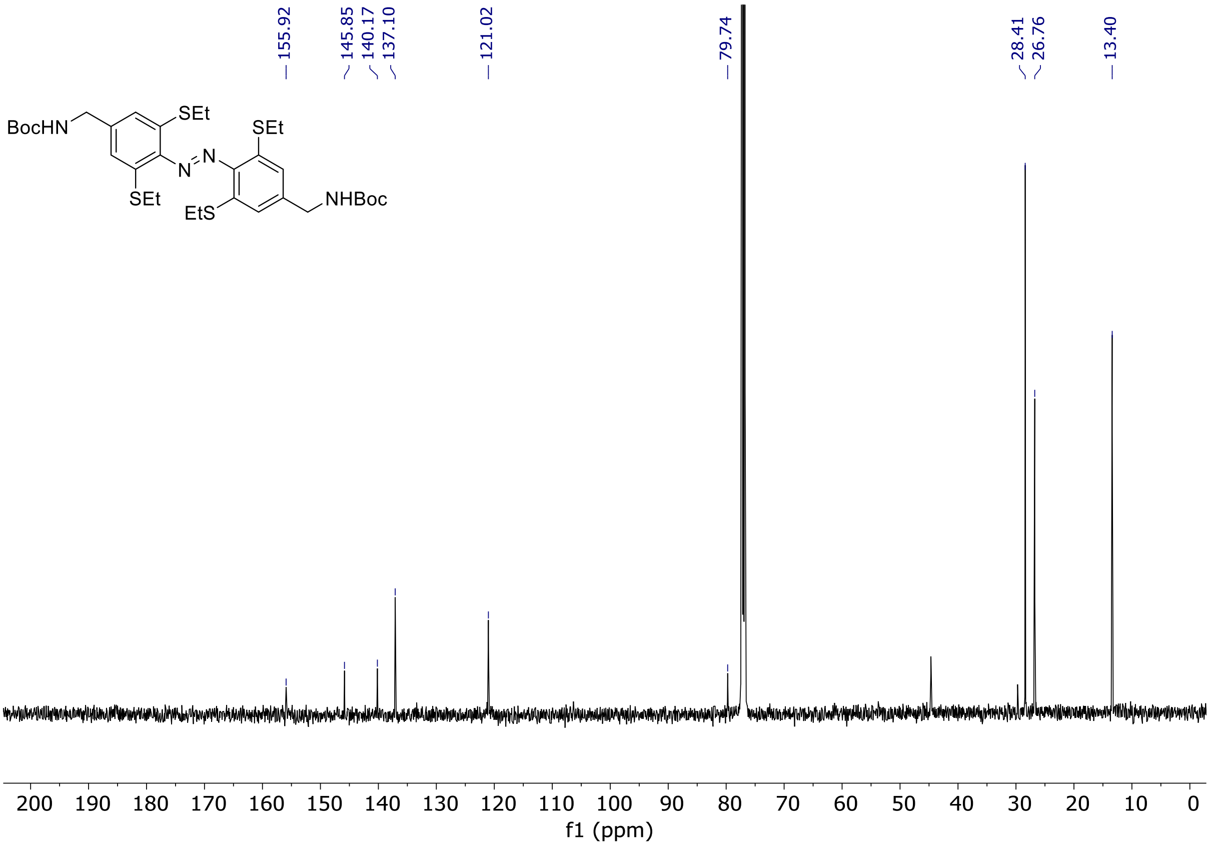

Figure S72. ^{13}C NMR Spectrum of **(SEt)$_4$-NHBoc** (Chloroform-*d*, 298 K).

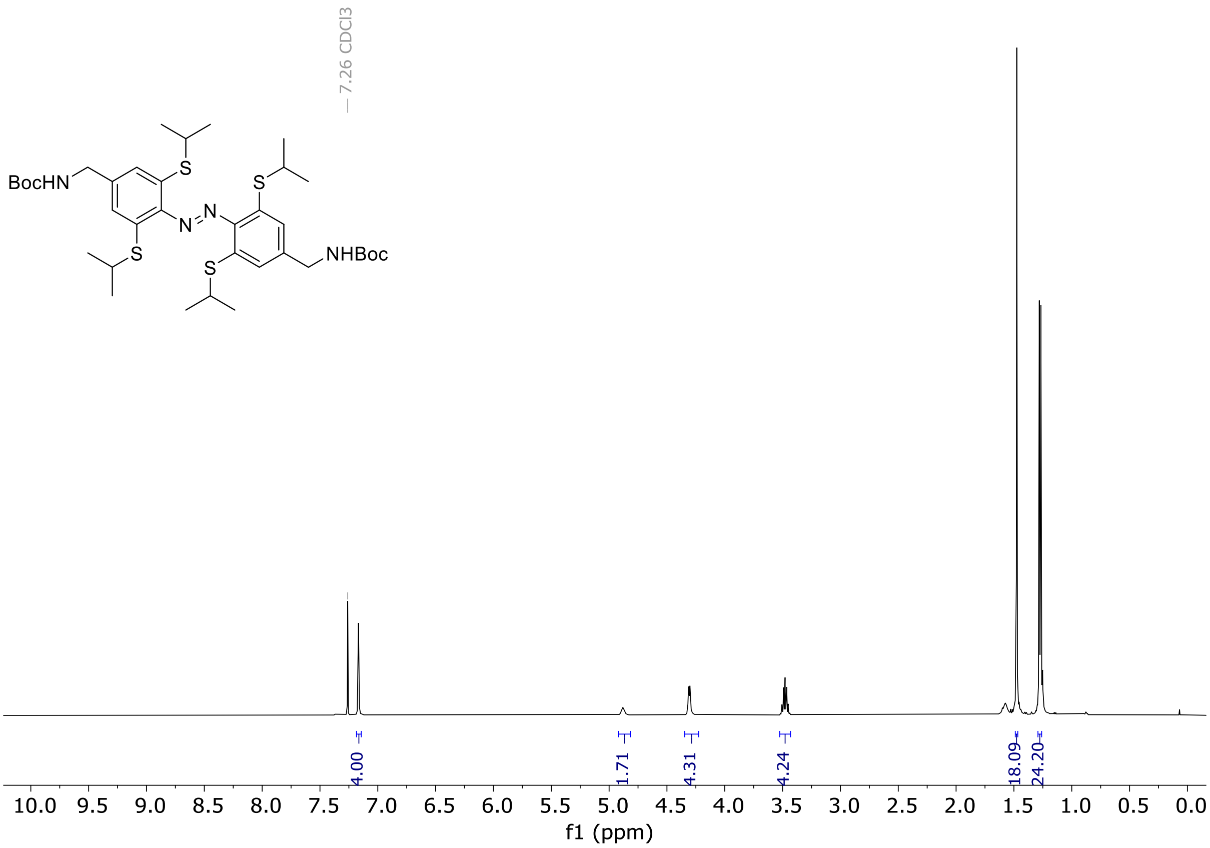

Figure S73. ^{1}H NMR Spectrum of **(S^{i}Pr)$_4$-NHBoc** (Chloroform-*d*, 298 K).

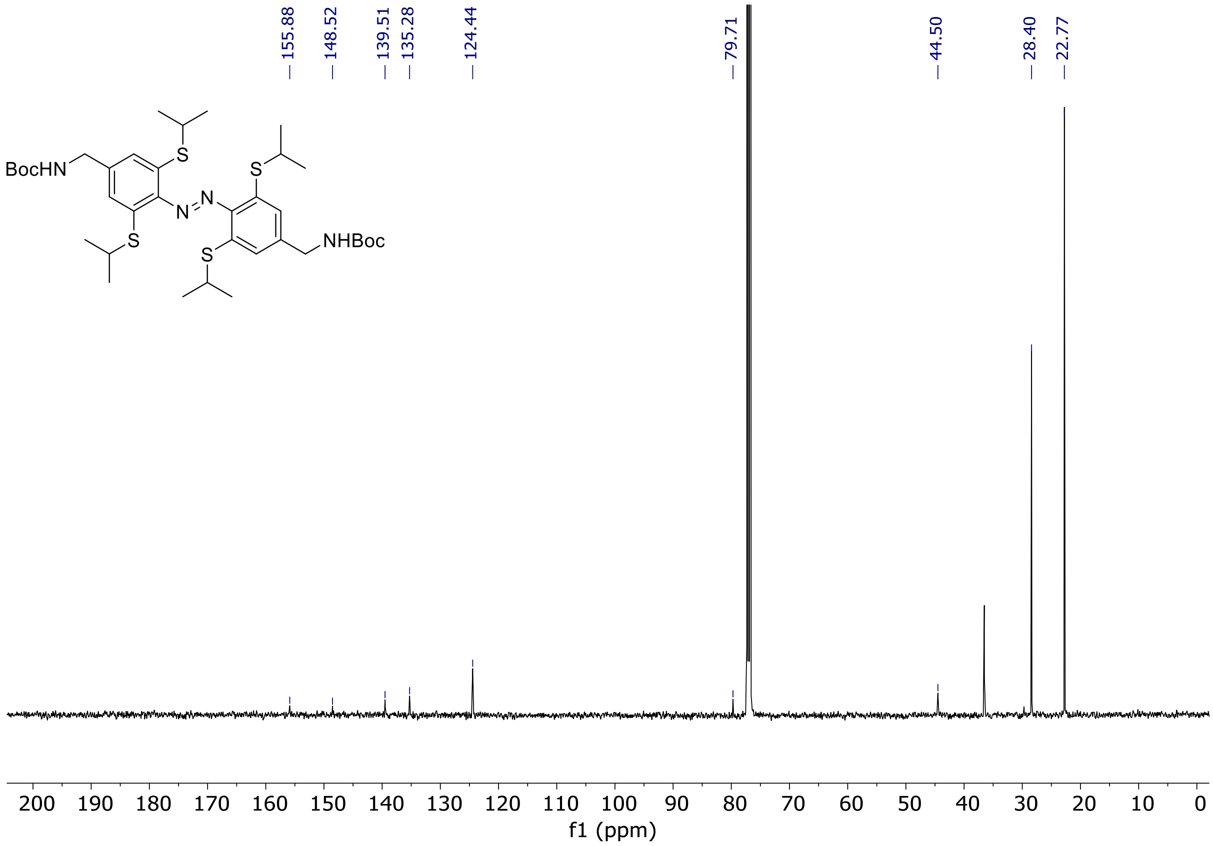

Figure S74. ^{13}C NMR Spectrum of **(S^{i}Pr)$_4$-NHBoc** (Chloroform-*d*, 298 K).

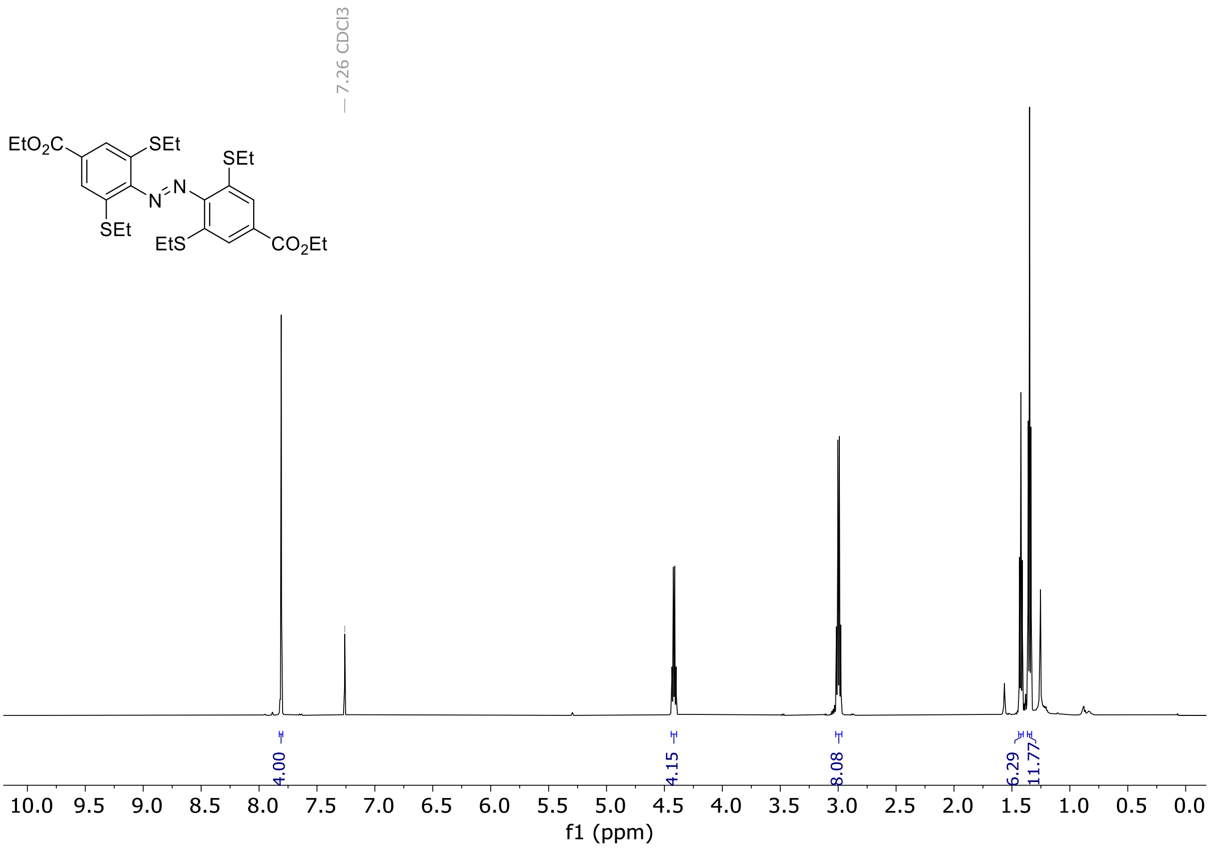

Figure S75. ^{1}H NMR Spectrum of **(SEt)$_4$-ester** (Chloroform-*d*, 298 K).

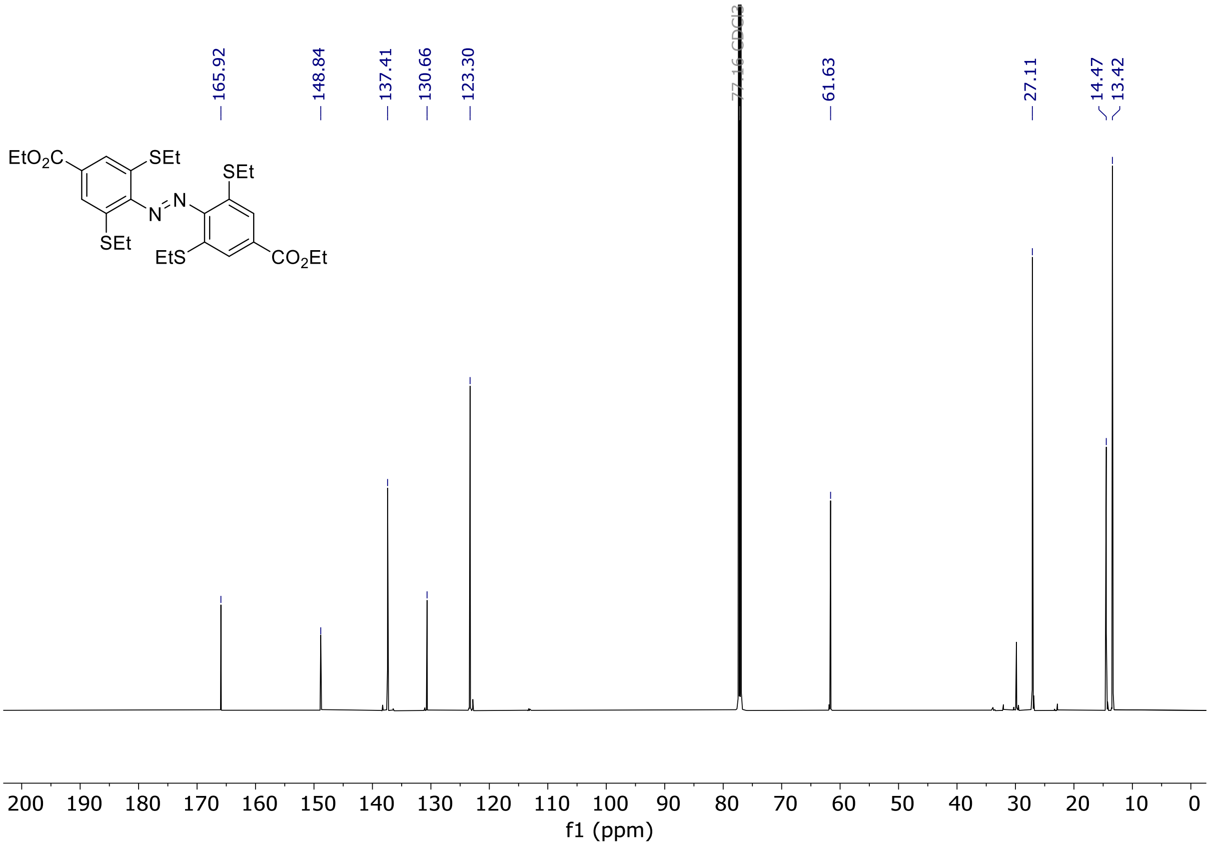

Figure S76. ^{13}C NMR Spectrum of **(SEt)$_4$-ester** (Chloroform-*d*, 298 K).

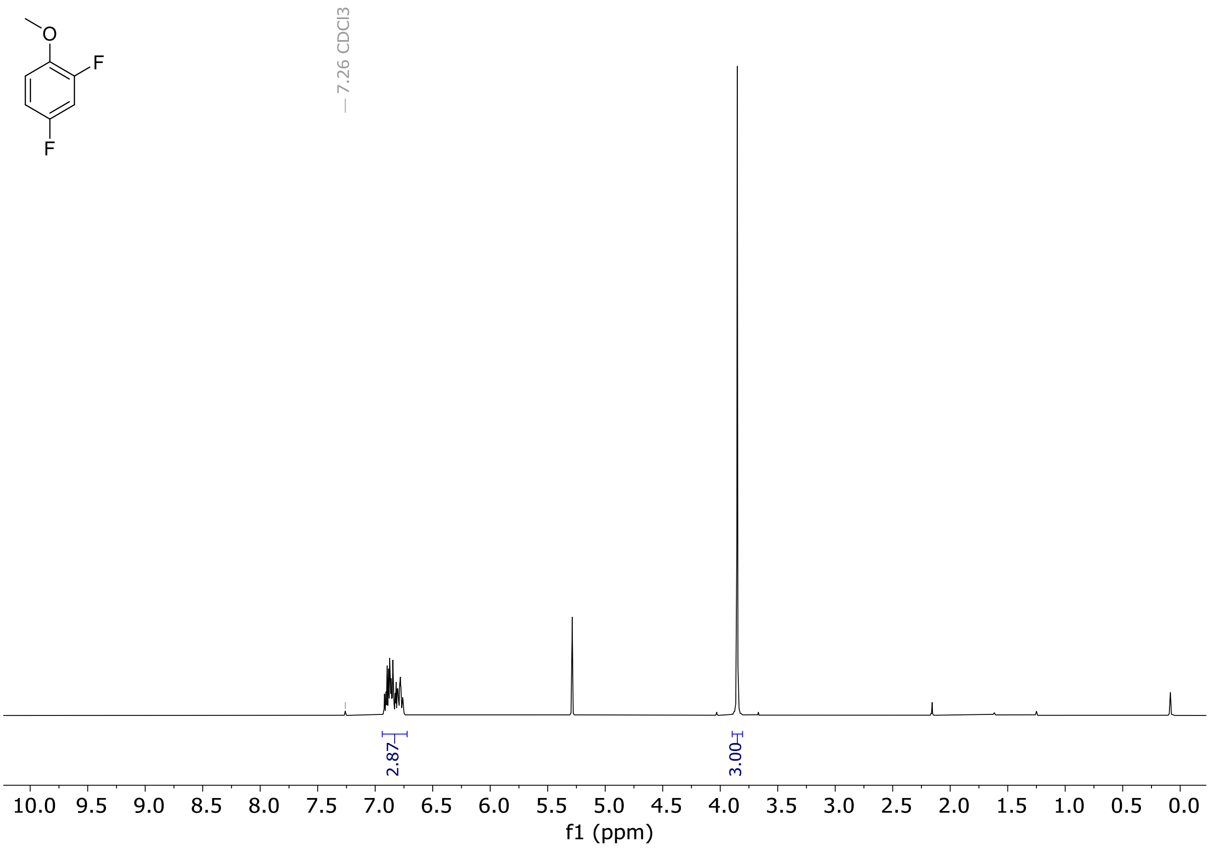

Figure S77. ^{1}H NMR Spectrum of **S43** (Chloroform-*d*, 298 K).

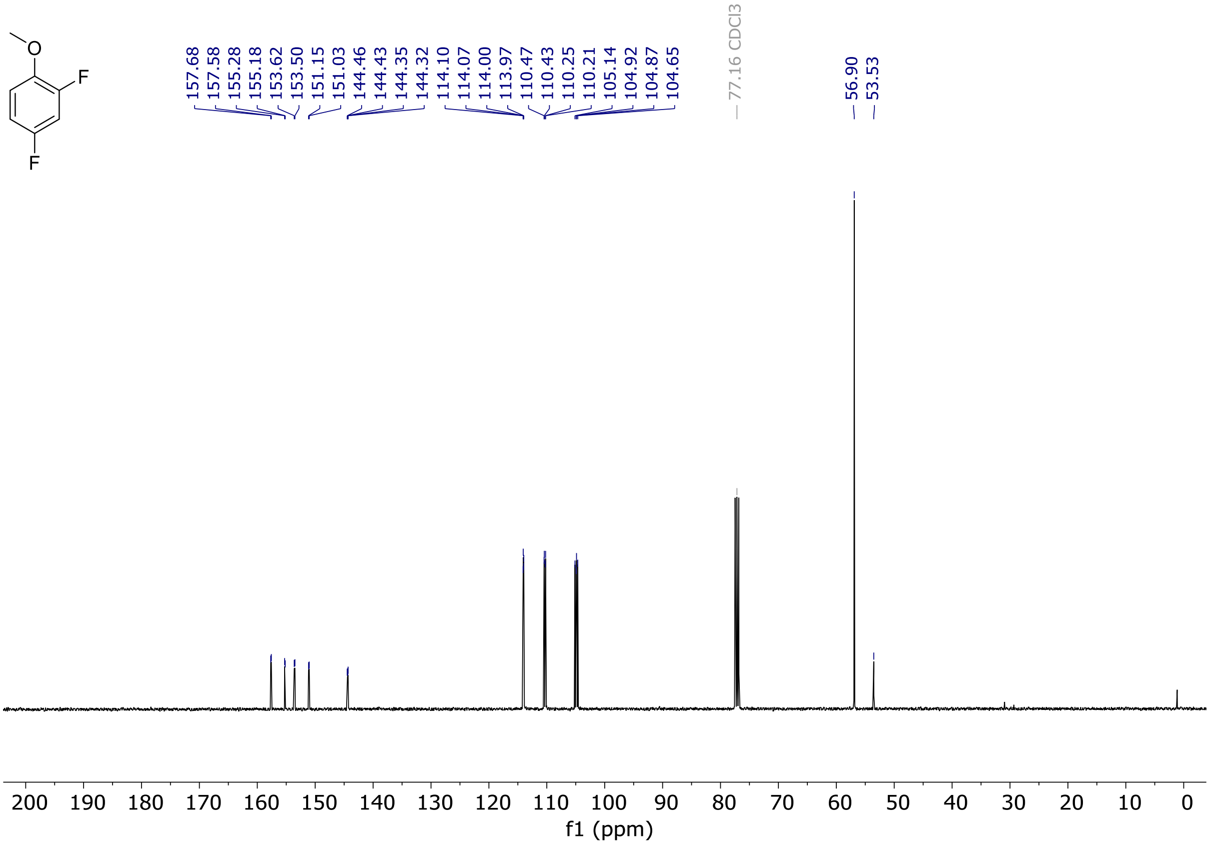

Figure S78. ^{13}C NMR Spectrum of **S44** (Chloroform-*d*, 298 K).

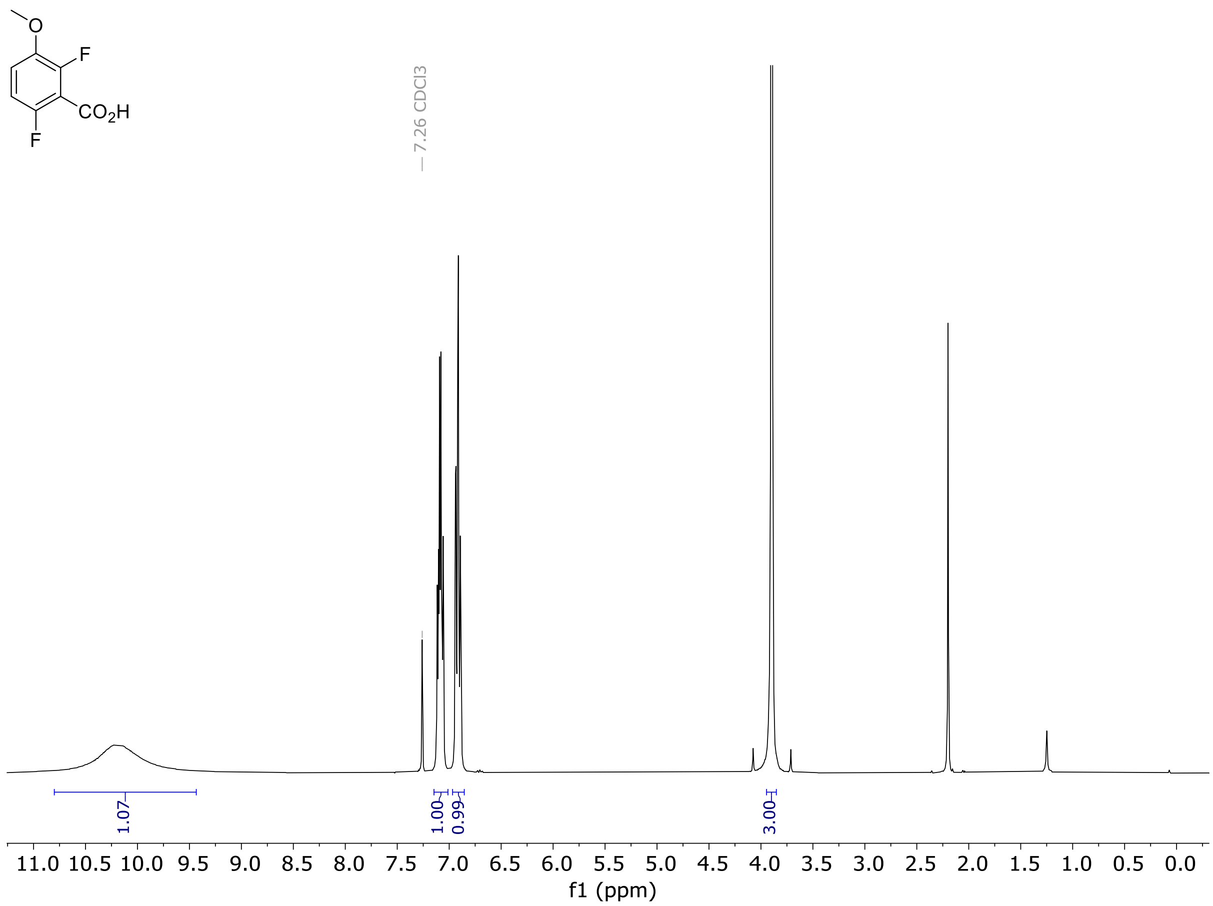

Figure S79. ^{1}H NMR Spectrum of **S45** (Chloroform-*d*, 298 K).

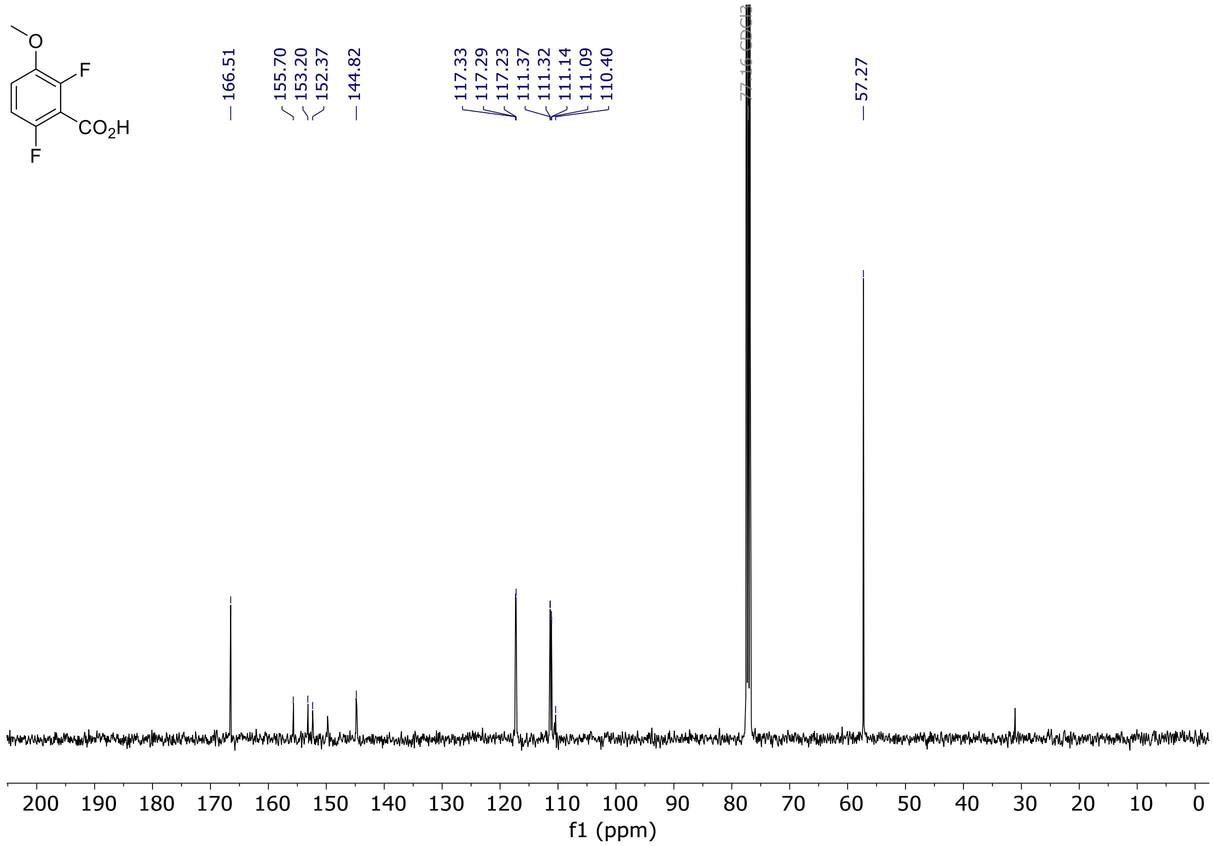

Figure S80. ^{13}C NMR Spectrum of **S45** (Chloroform-*d*, 298 K).

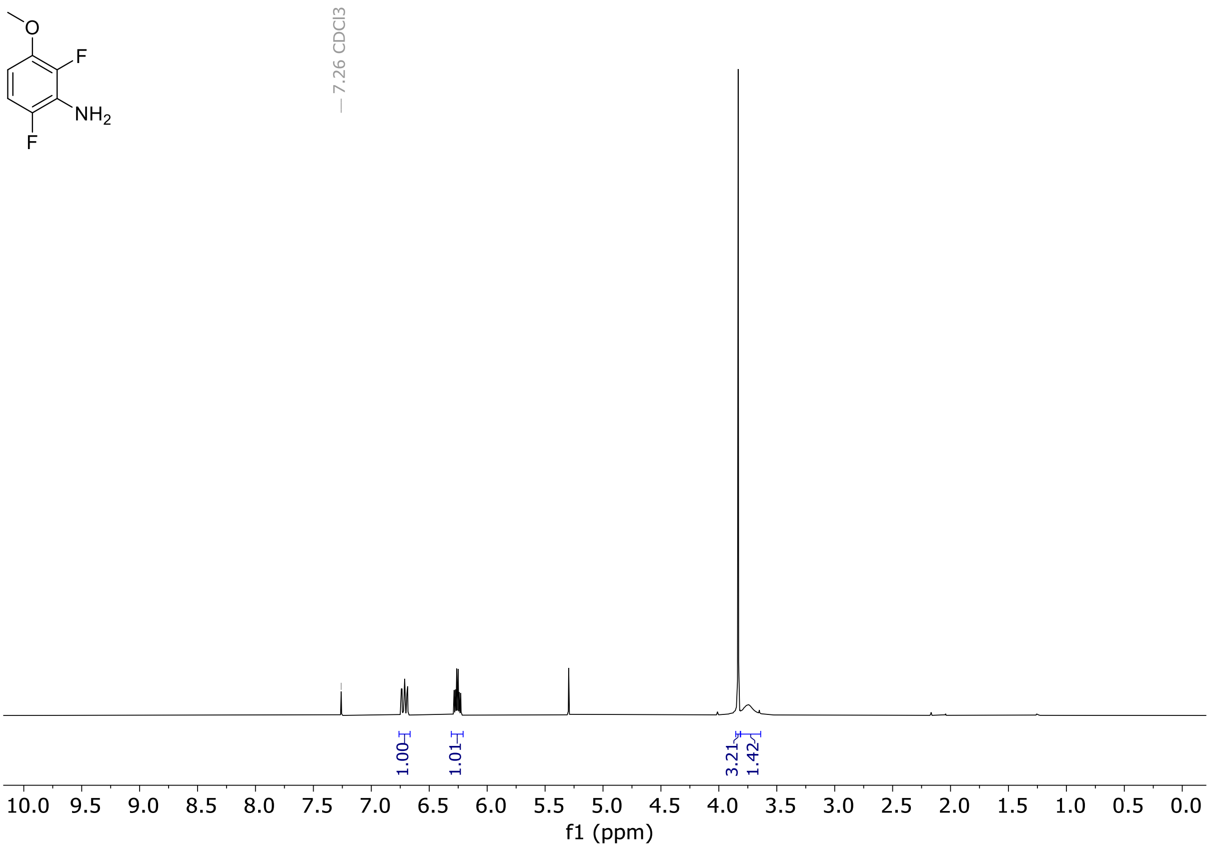

Figure S81. ^{1}H NMR Spectrum of **S47** (Chloroform-*d*, 298 K).

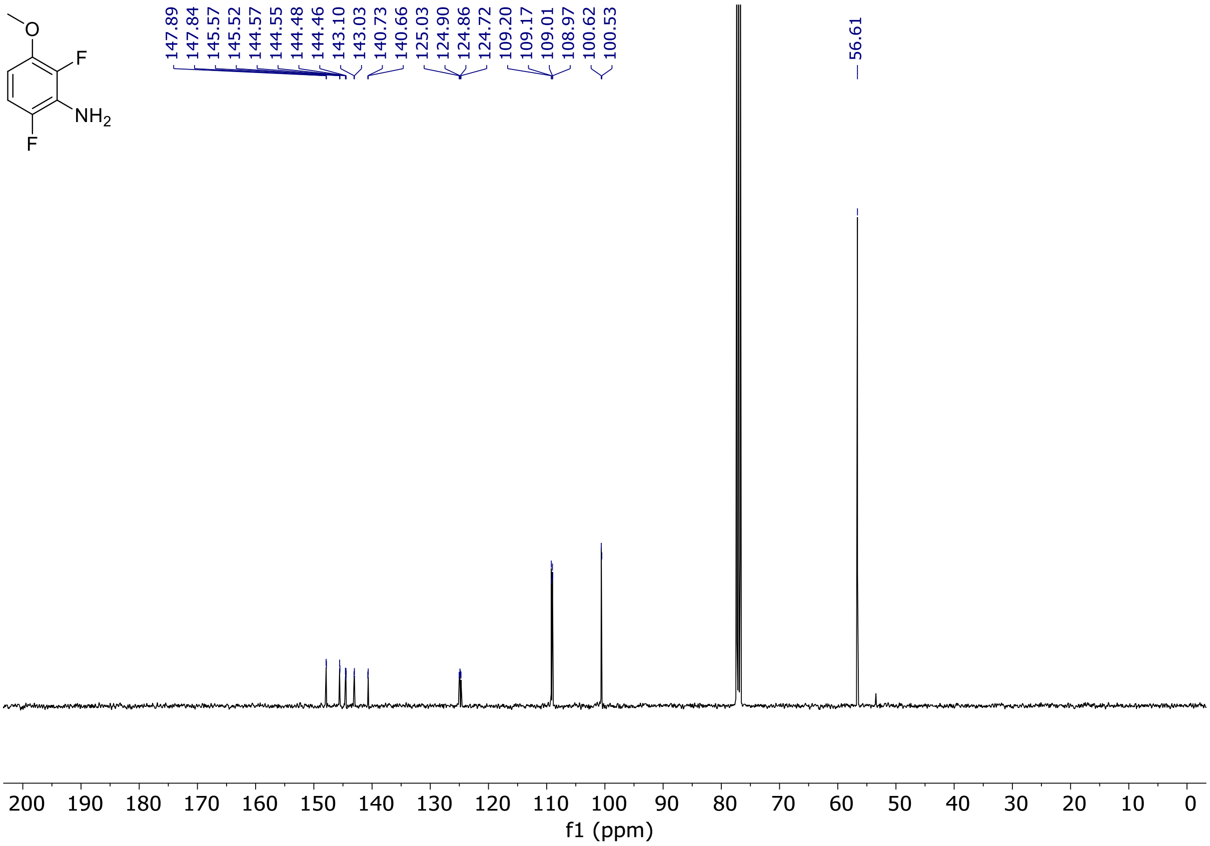

Figure S82. ^{13}C NMR Spectrum of **S47** (Chloroform-*d*, 298 K).

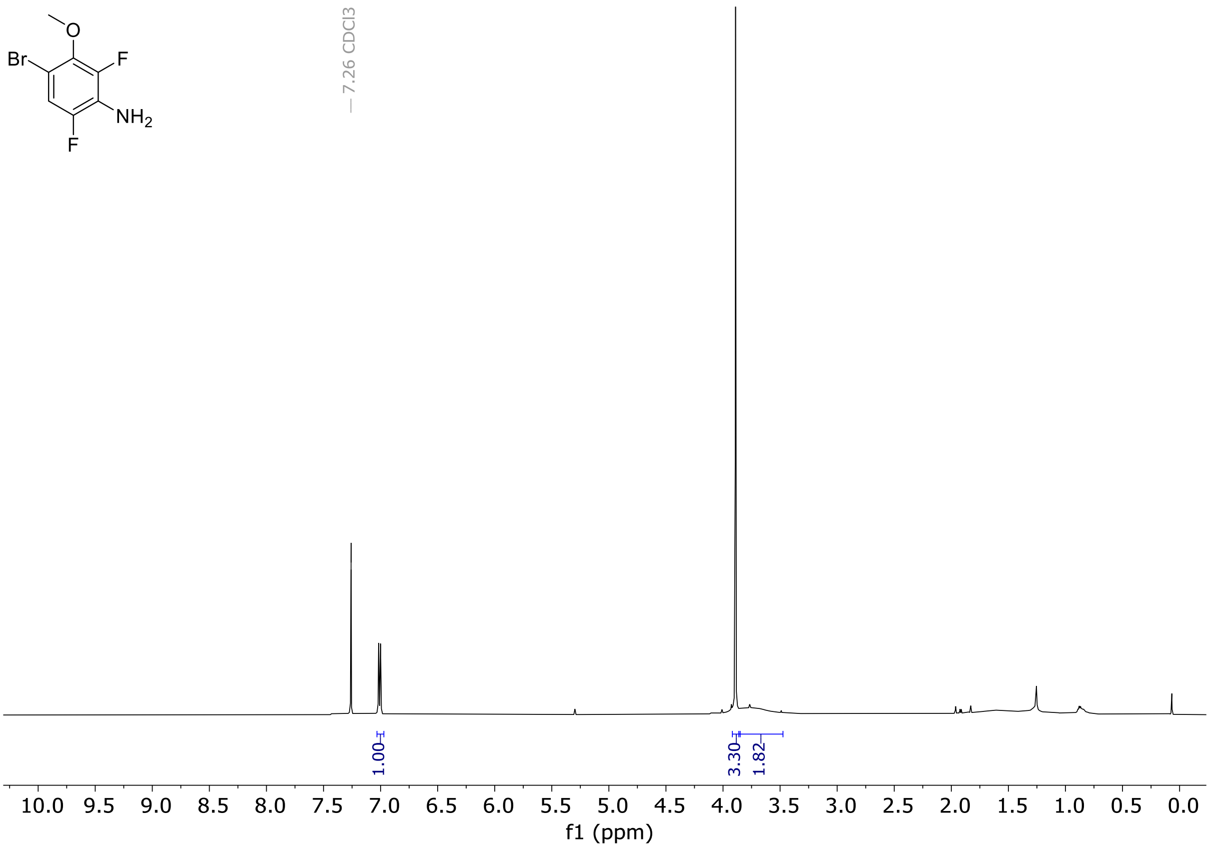

Figure S83. ^{1}H NMR Spectrum of **S48** (Chloroform-*d*, 298 K).

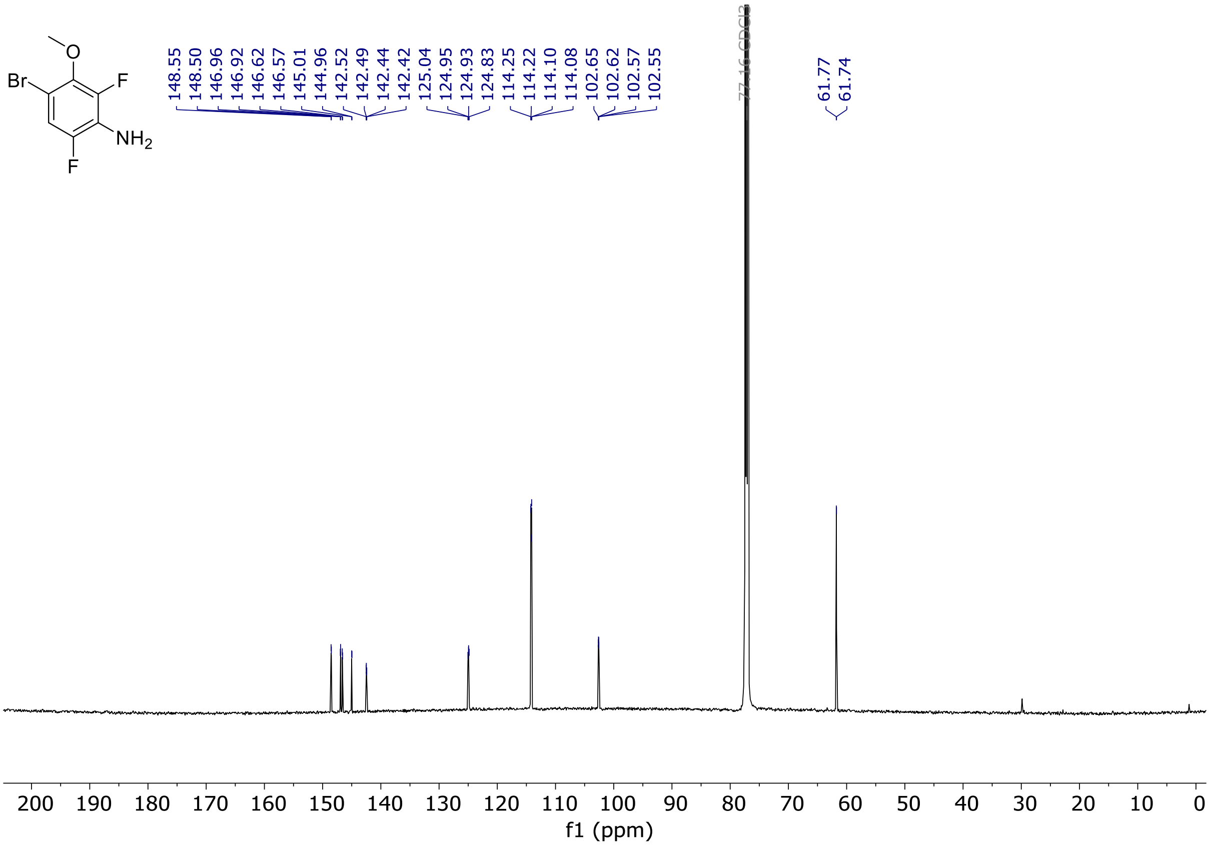

Figure S84. ^{13}C NMR Spectrum of **S48** (Chloroform-*d*, 298 K).

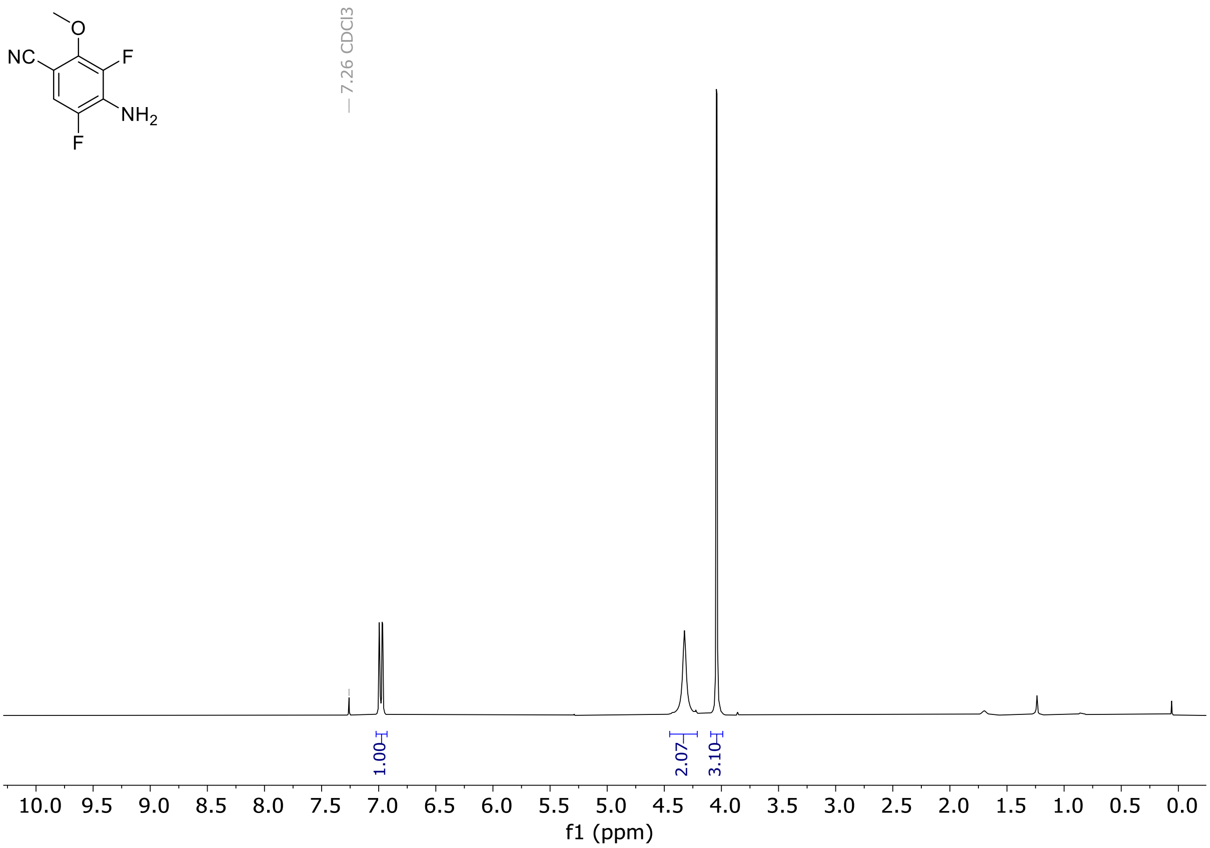

Figure S85. ^{1}H NMR Spectrum of **S49** (Chloroform-*d*, 298 K).

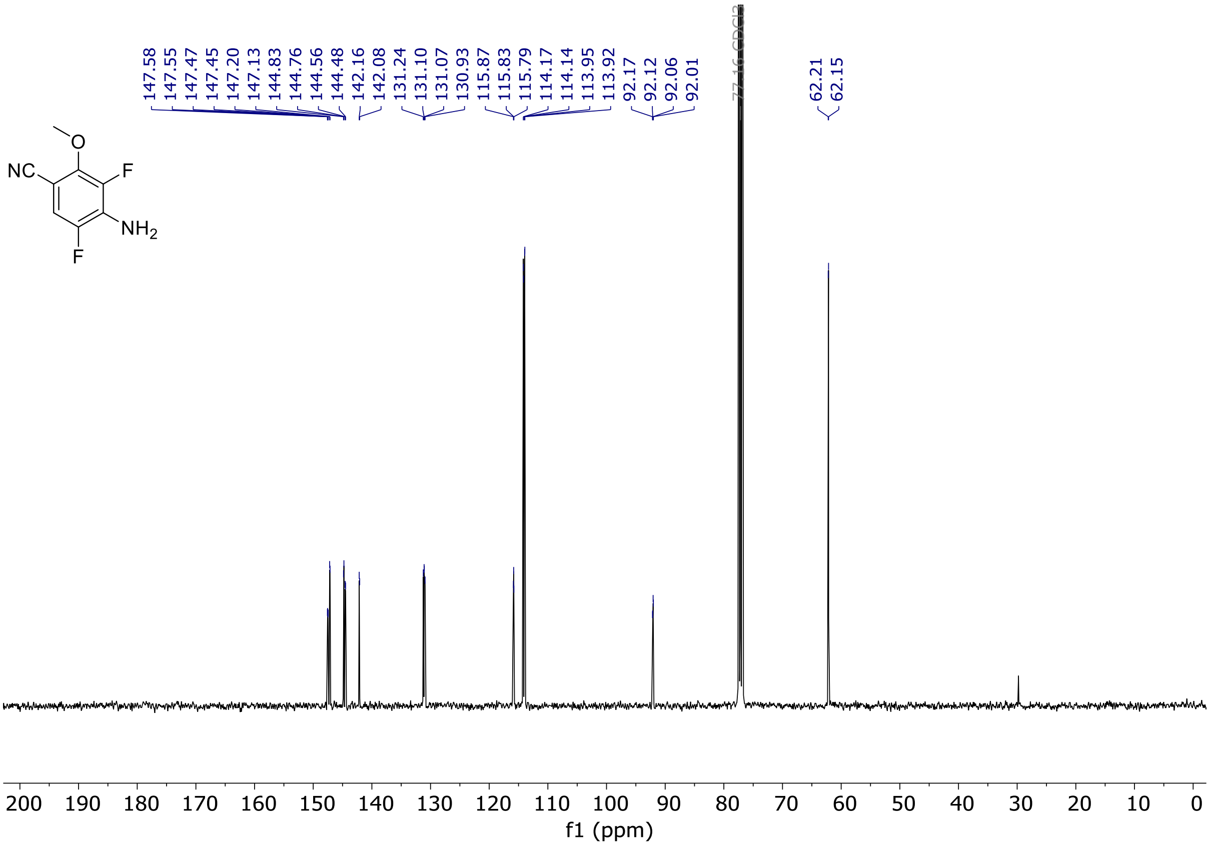

Figure S86. ^{13}C NMR Spectrum of **S49** (Chloroform-*d*, 298 K).

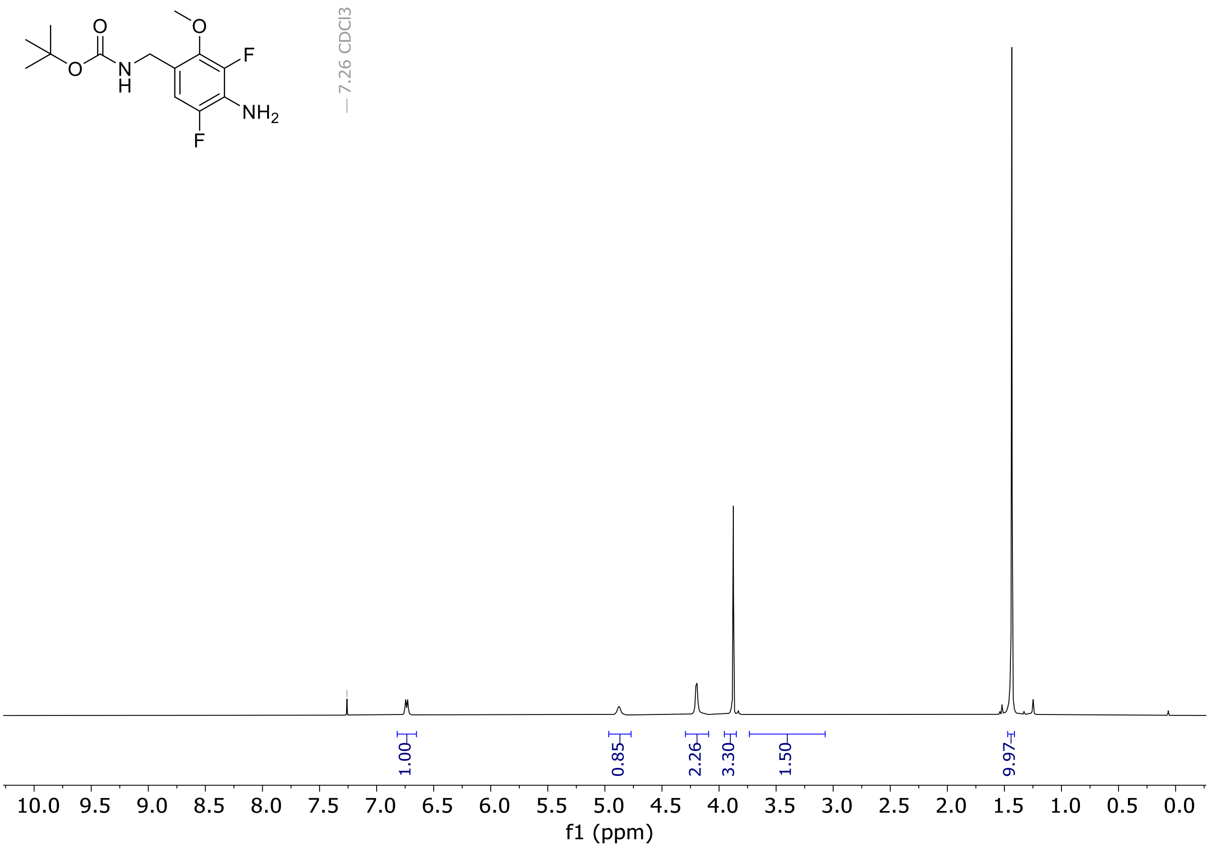

Figure S87. ^{1}H NMR Spectrum of **S50** (Chloroform-*d*, 298 K).

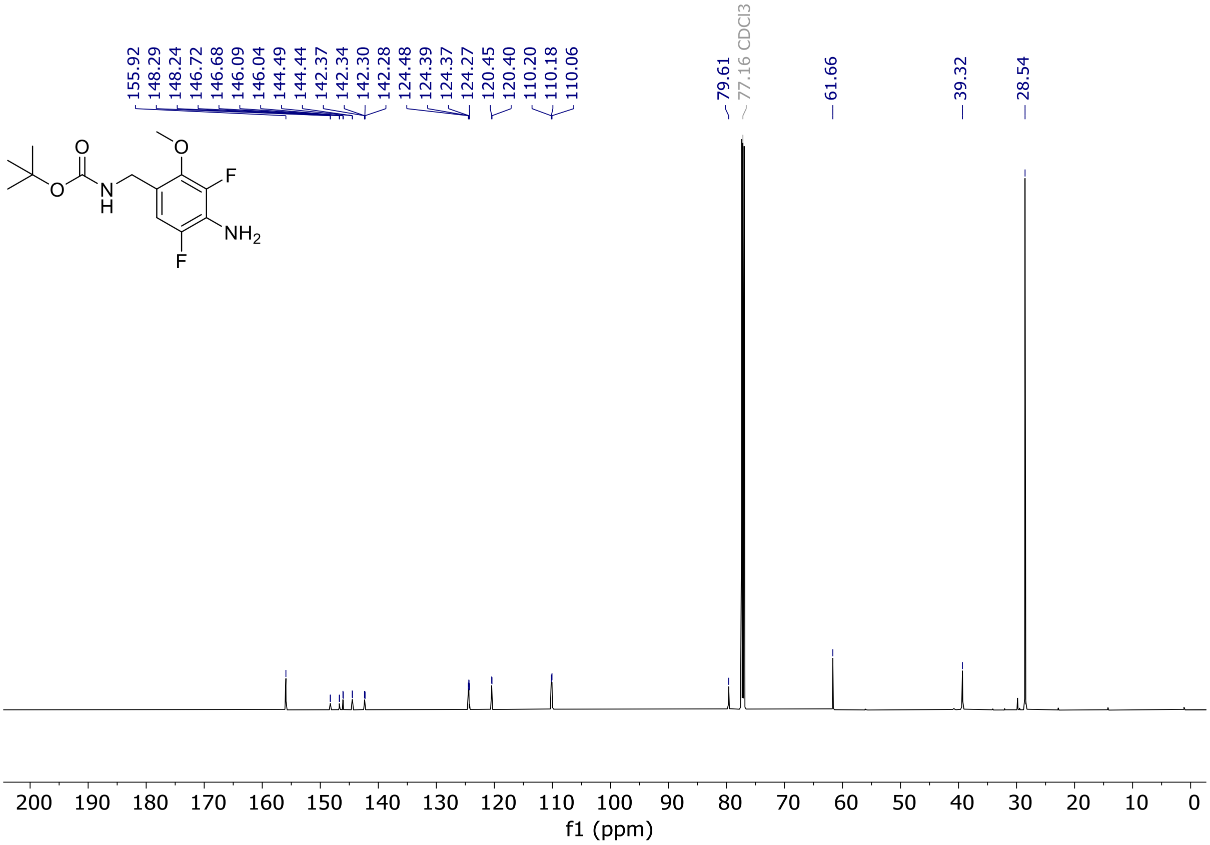

Figure S88. ^{13}C NMR Spectrum of **S50** (Chloroform-*d*, 298 K).

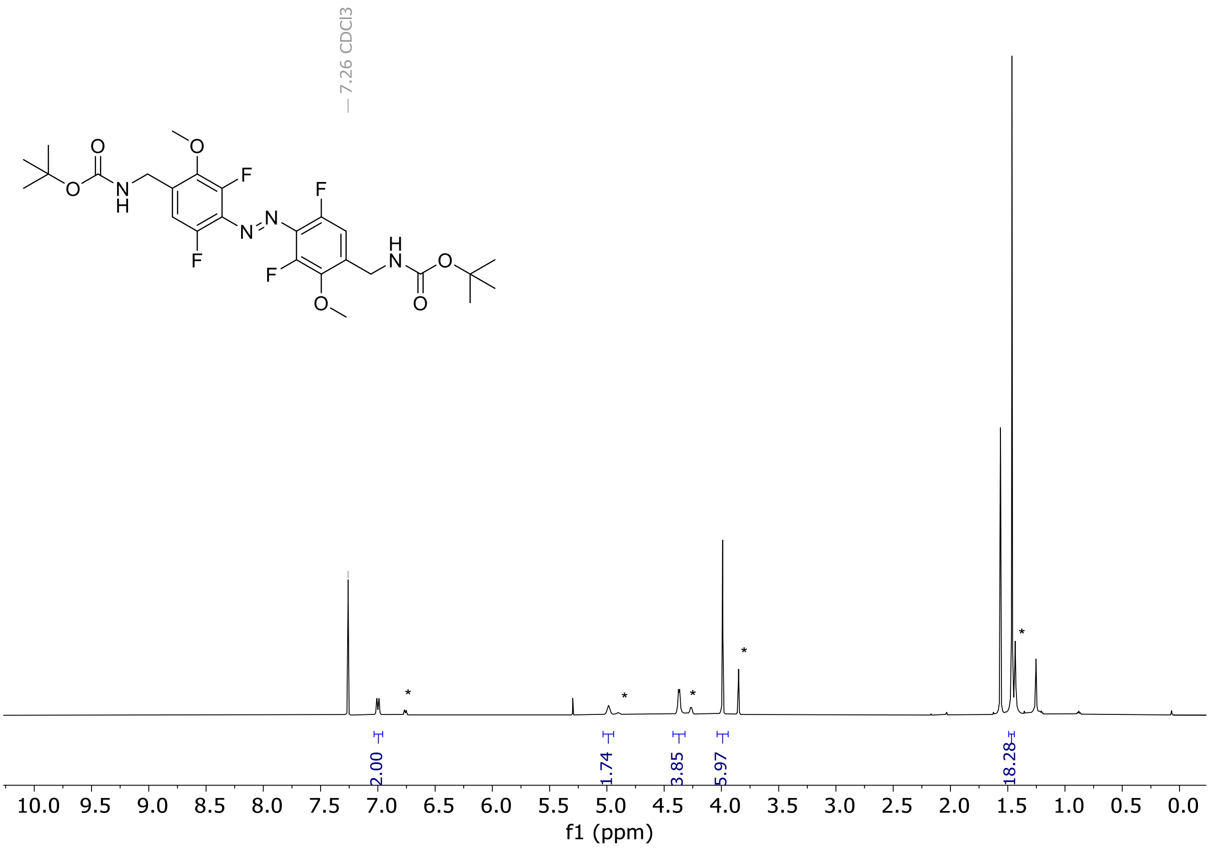

Figure S89. ^{1}H NMR spectrum of **F$_4$,*m*-OMe$_2$-NHBoc** (Chloroform-*d*, 298 K). *Z* signals labelled as *.

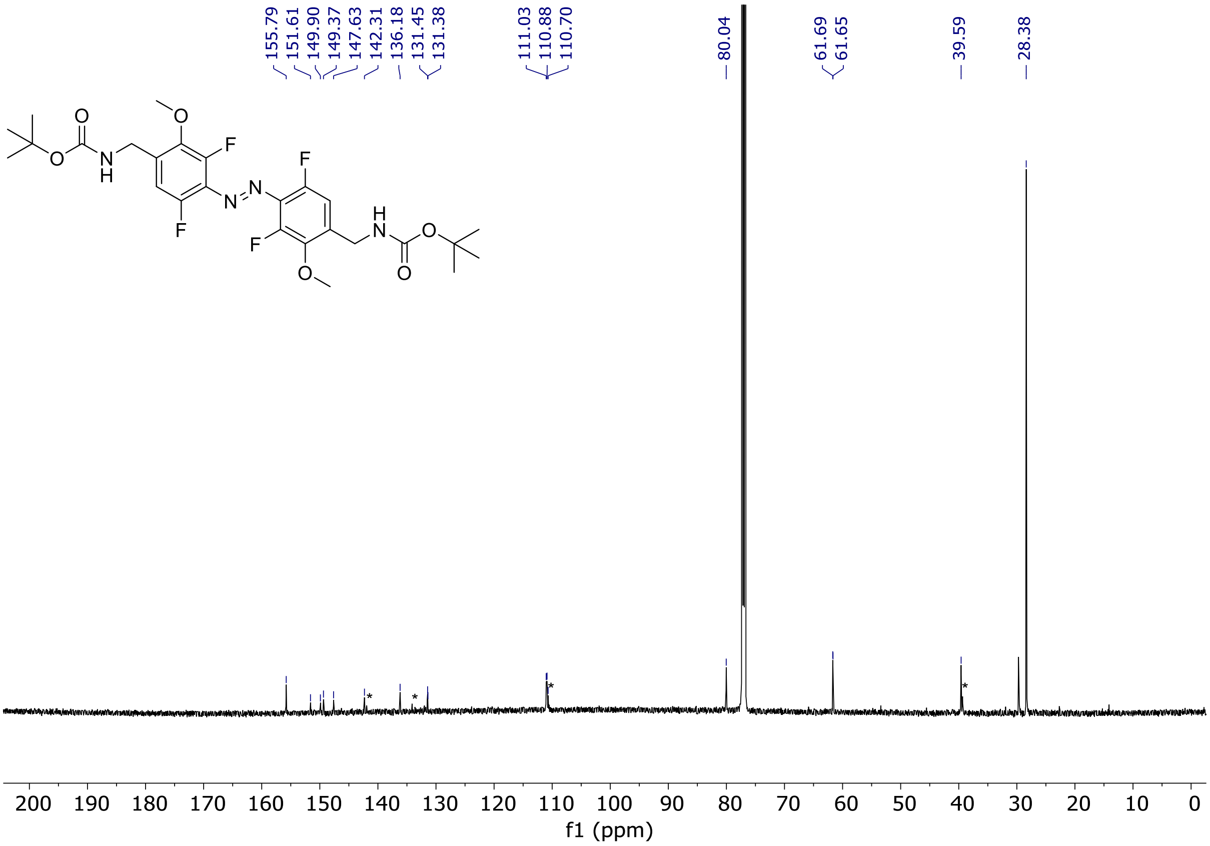

Figure S90. ^{13}C NMR Spectrum of **F$_4$,*m*-OMe$_2$-NHBoc** (Chloroform-*d*, 298 K). *Z* signals labelled as *.

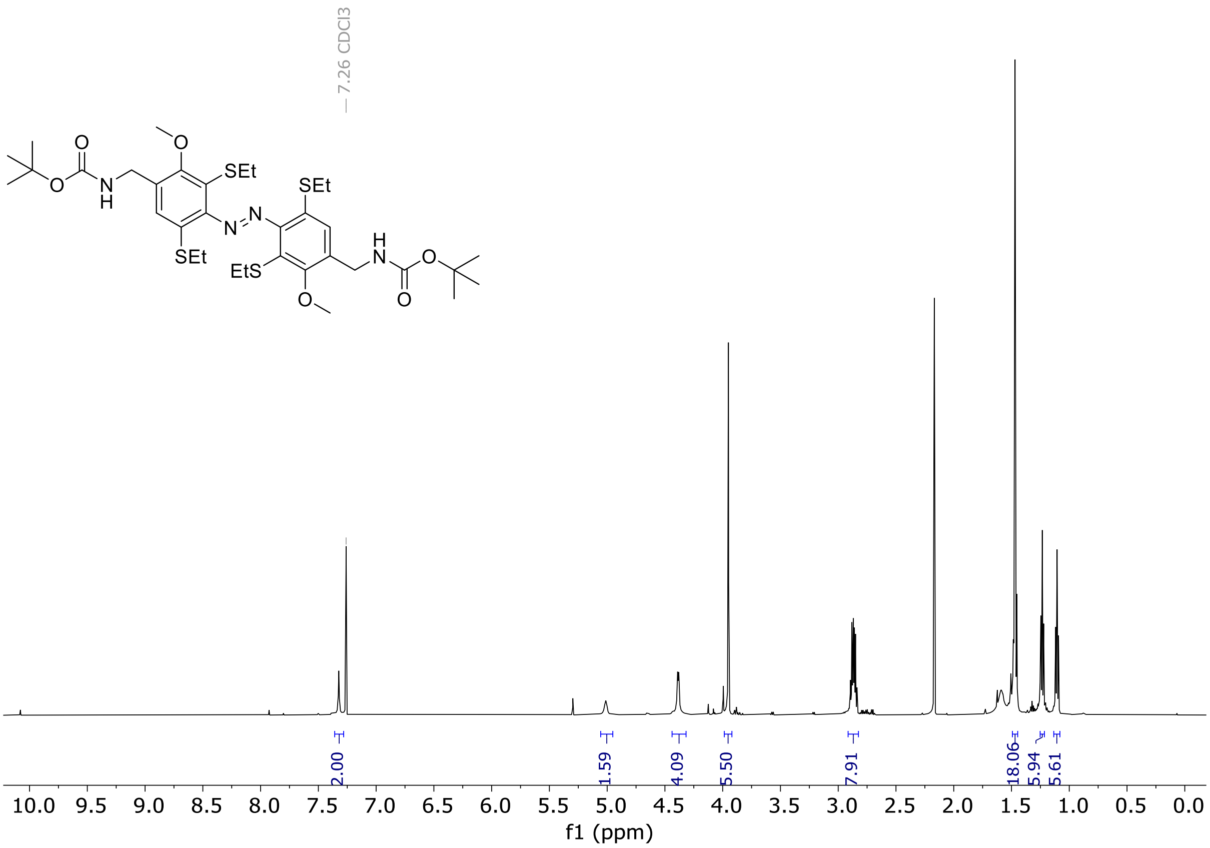

Figure S91. ^{1}H NMR spectrum of **(SEt)$_4$,*m*-OMe$_2$-NHBoc.** (Chloroform-*d*, 298 K).

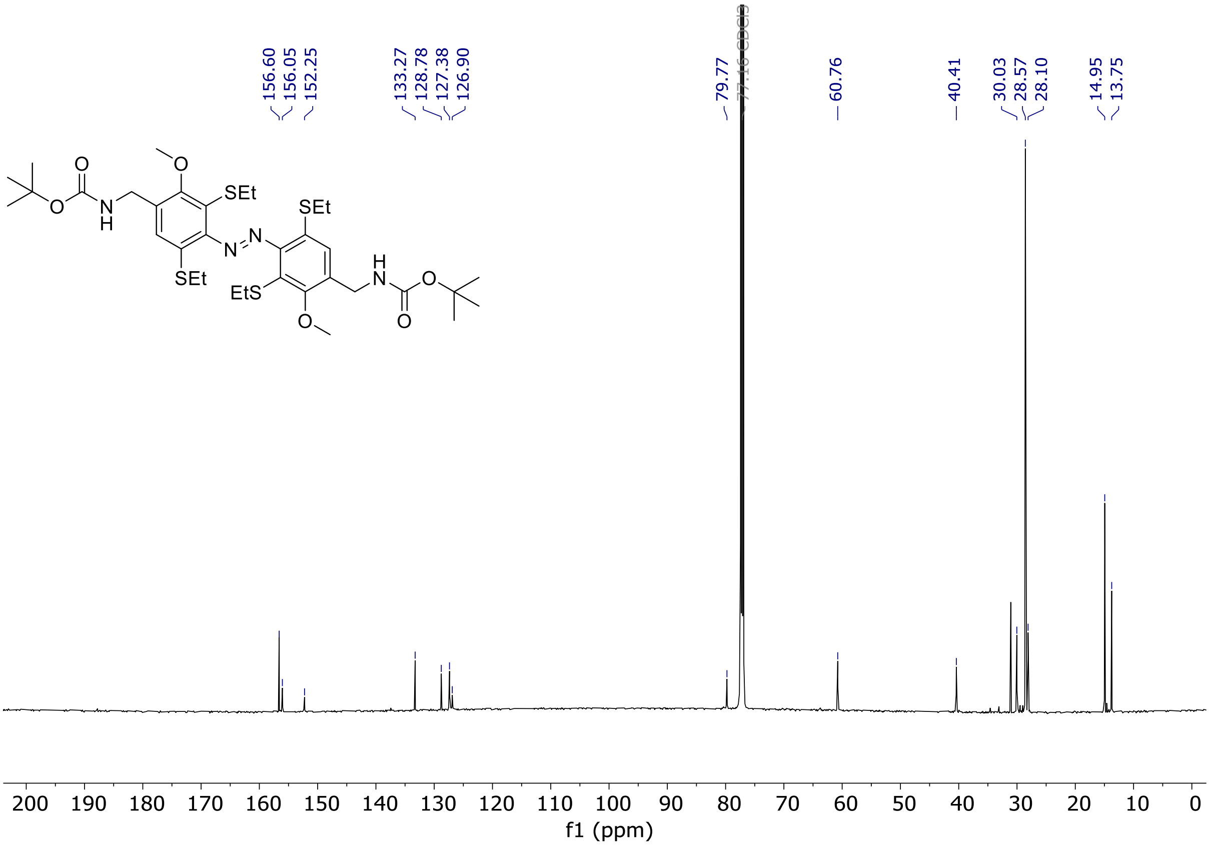

Figure S92. ^{13}C NMR spectrum of **(SEt)$_4$,*m*-OMe$_2$-NHBoc** (Chloroform-*d*, 298 K).

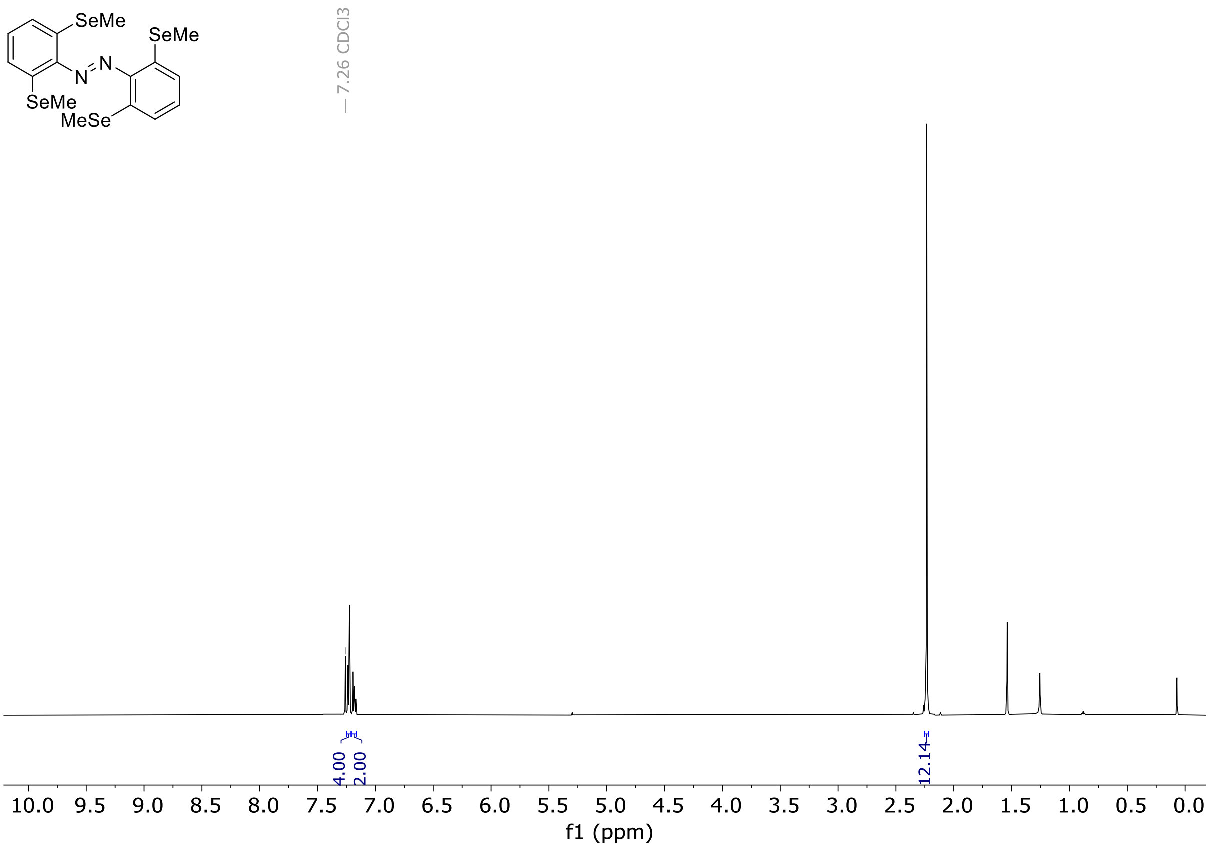

Figure S93. ^{1}H NMR Spectrum of **(SeMe$_4$)-H** (Chloroform-*d*, 298 K).

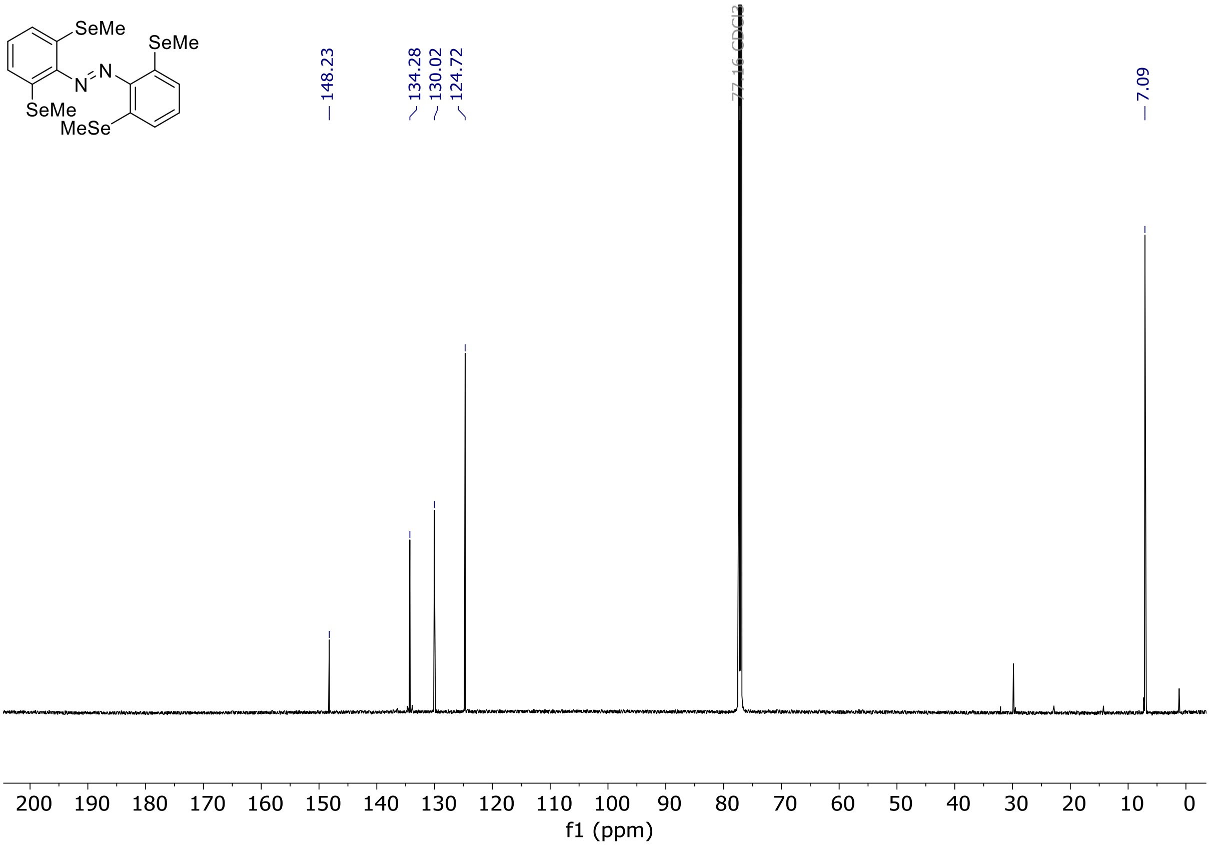

Figure S94. ^{13}C NMR Spectrum of **(SeMe$_4$)-H** (Chloroform-*d*, 298 K).

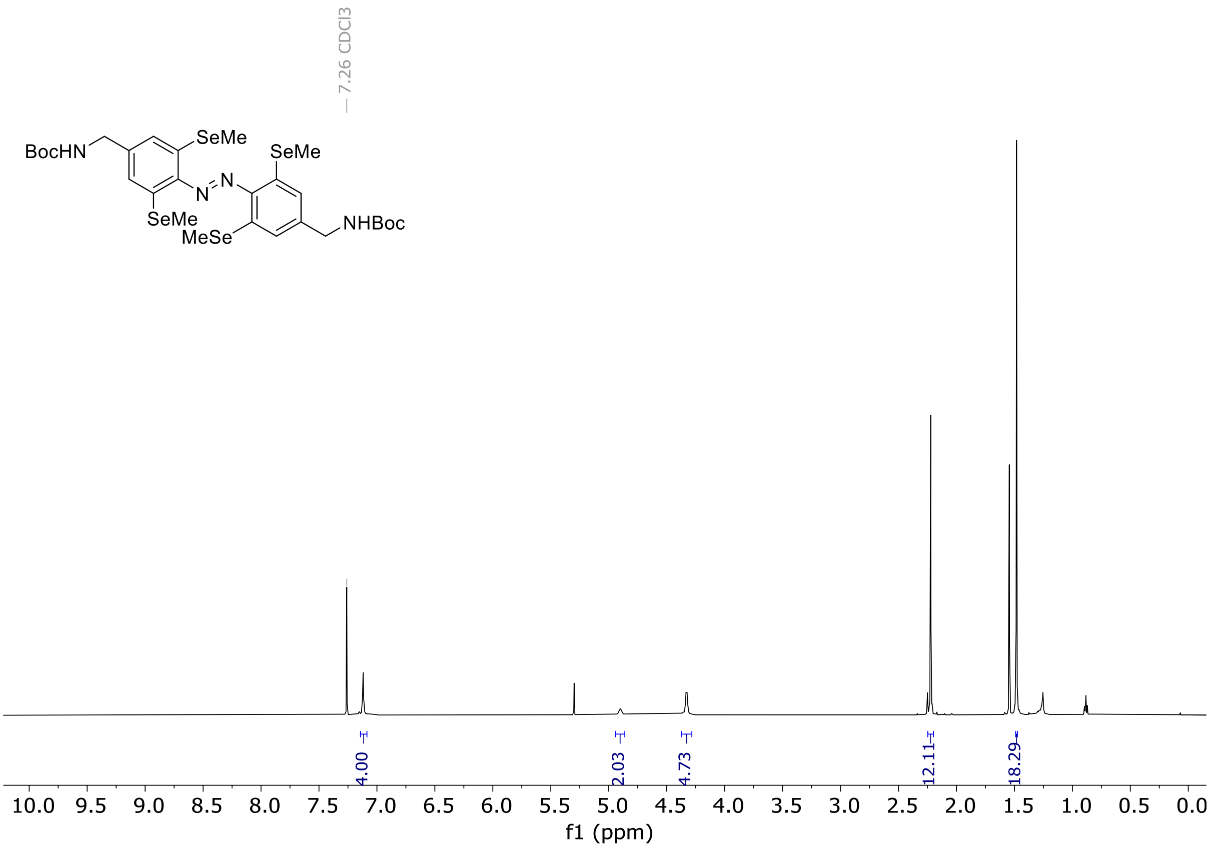

Figure S95. ^{1}H NMR Spectrum of **(SeMe$_4$)-NHBoc** (Chloroform-*d*, 298 K).

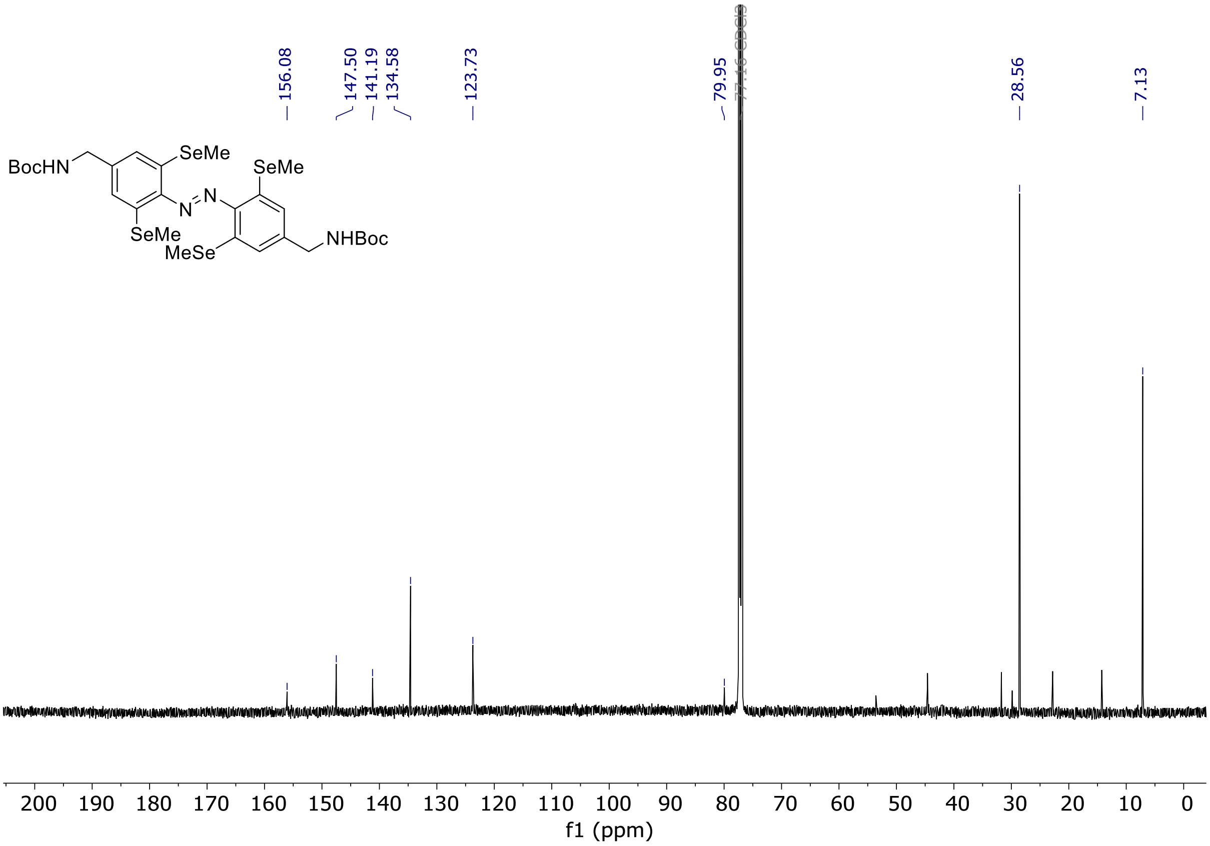

Figure S96. ^{13}C NMR Spectrum of **(SeMe$_4$)-NHBoc** (Chloroform-*d*, 298 K).

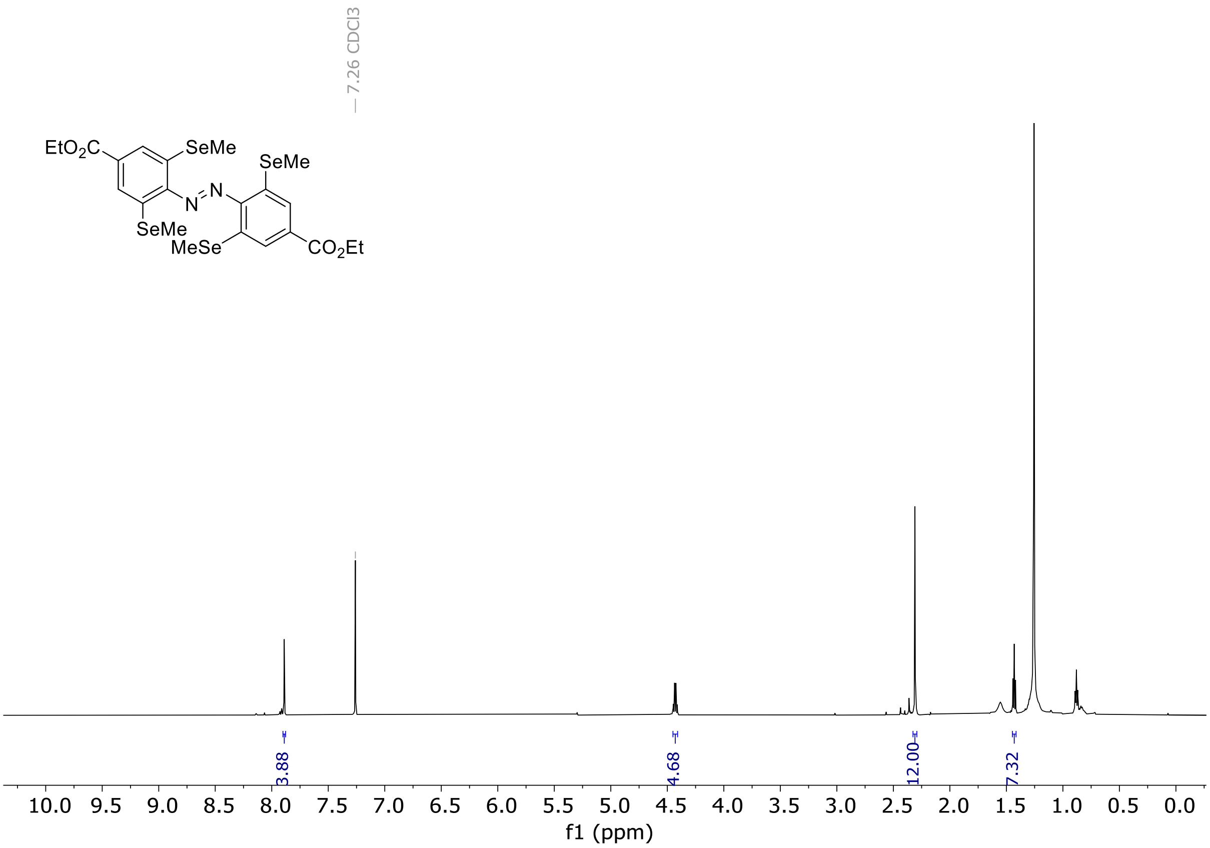

Figure S97. ^{1}H NMR Spectrum of **(SeMe$_4$)-ester** (Chloroform-*d*, 298 K).

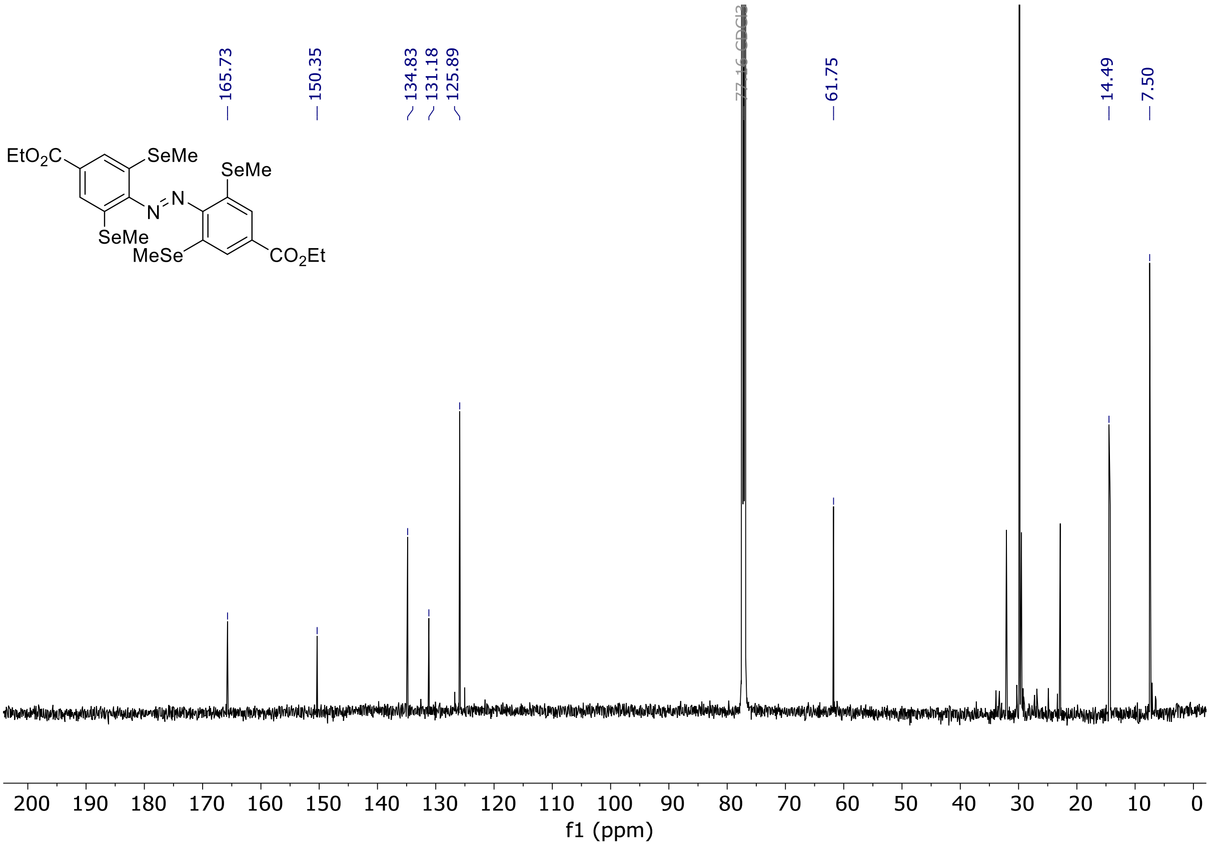

Figure S98. ^{13}C NMR Spectrum of **(SeMe$_4$)-ester** (Chloroform-*d*, 298 K).

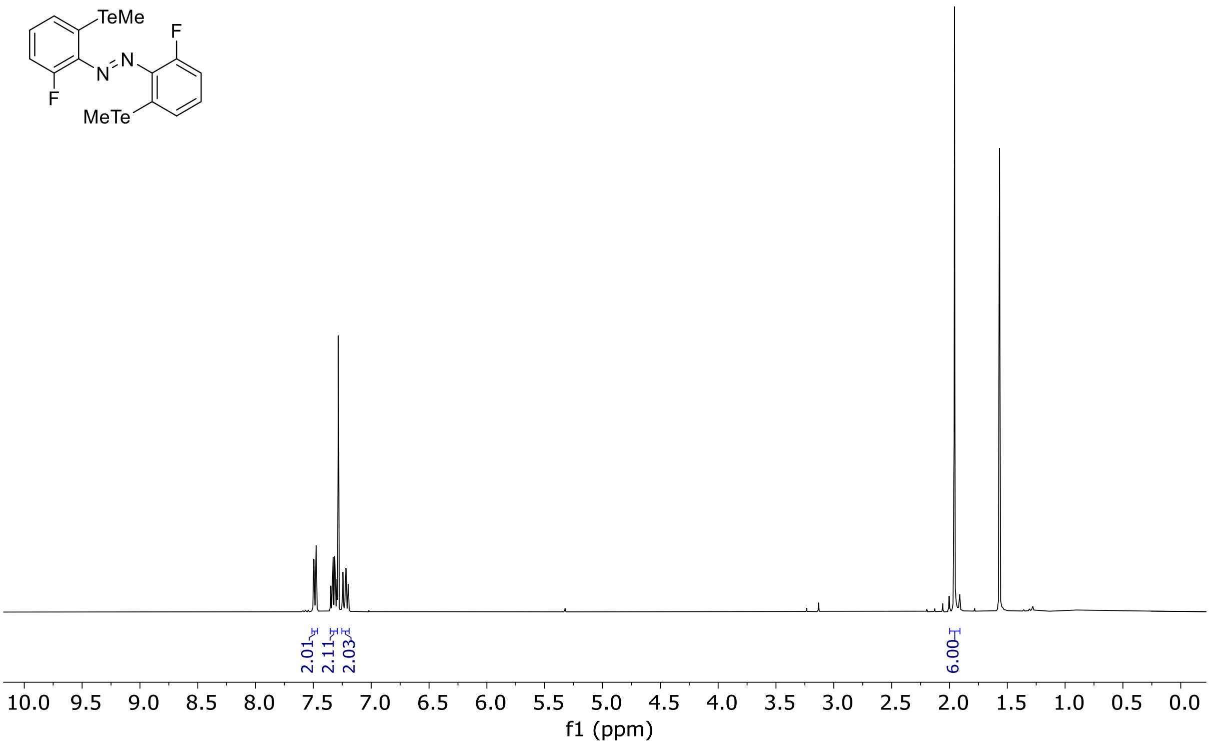

Figure S99. ^{1}H NMR Spectrum of **Te$_2$F$_2$-H** (Chloroform-*d*, 298 K).

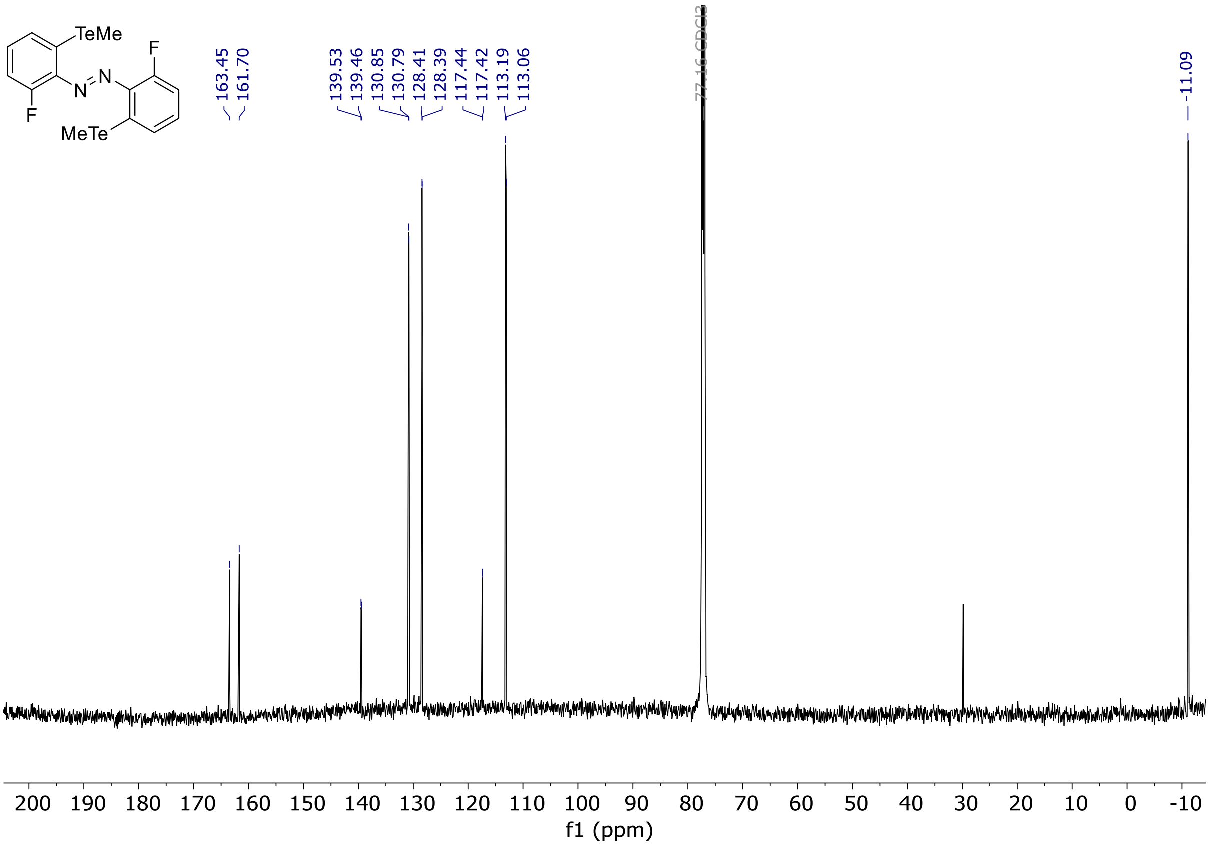

Figure S100. ^{13}C NMR Spectrum of **Te$_2$F$_2$-H** (Chloroform-*d*, 298 K).

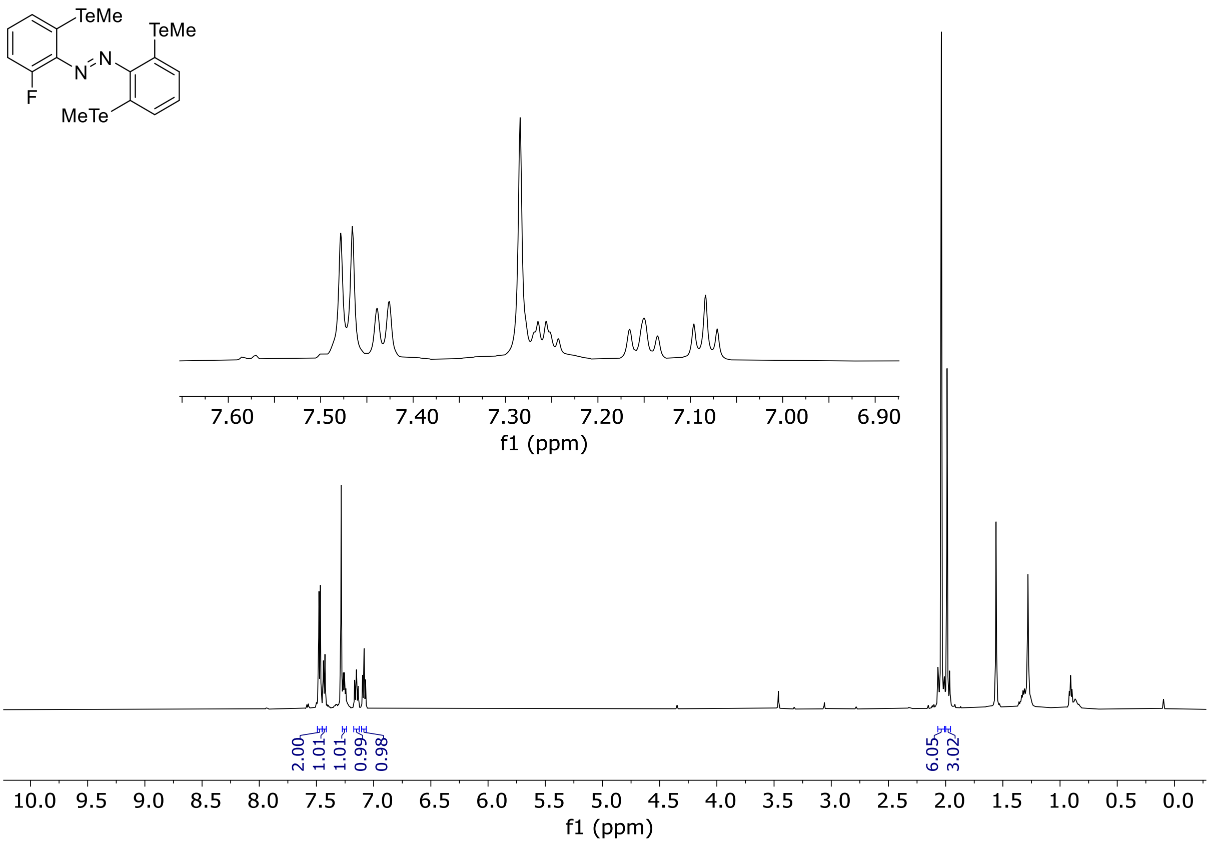

Figure S101. ^{1}H NMR Spectrum of **Te$_3$F-H** (Chloroform-*d*, 298 K).

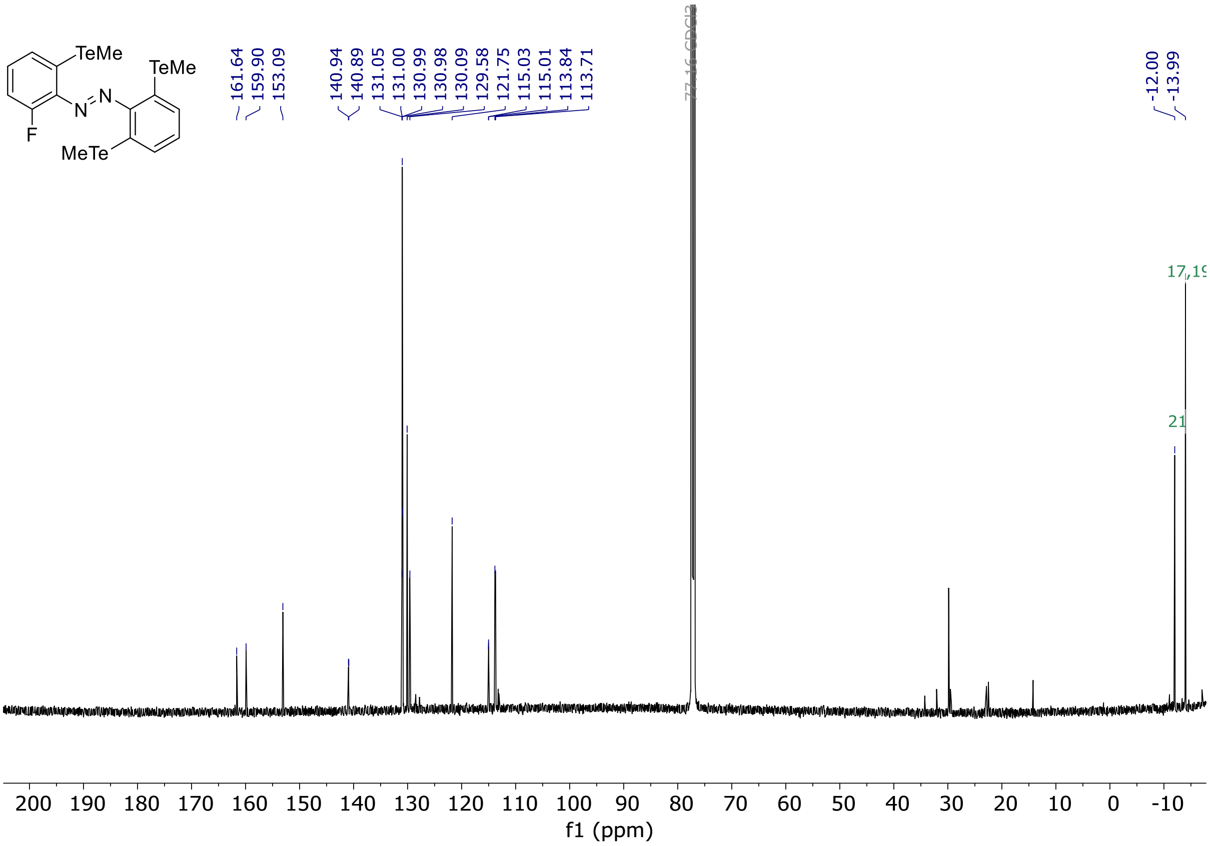

Figure S102. ^{13}C NMR Spectrum of **Te$_3$F-H** (Chloroform-*d*, 298 K).

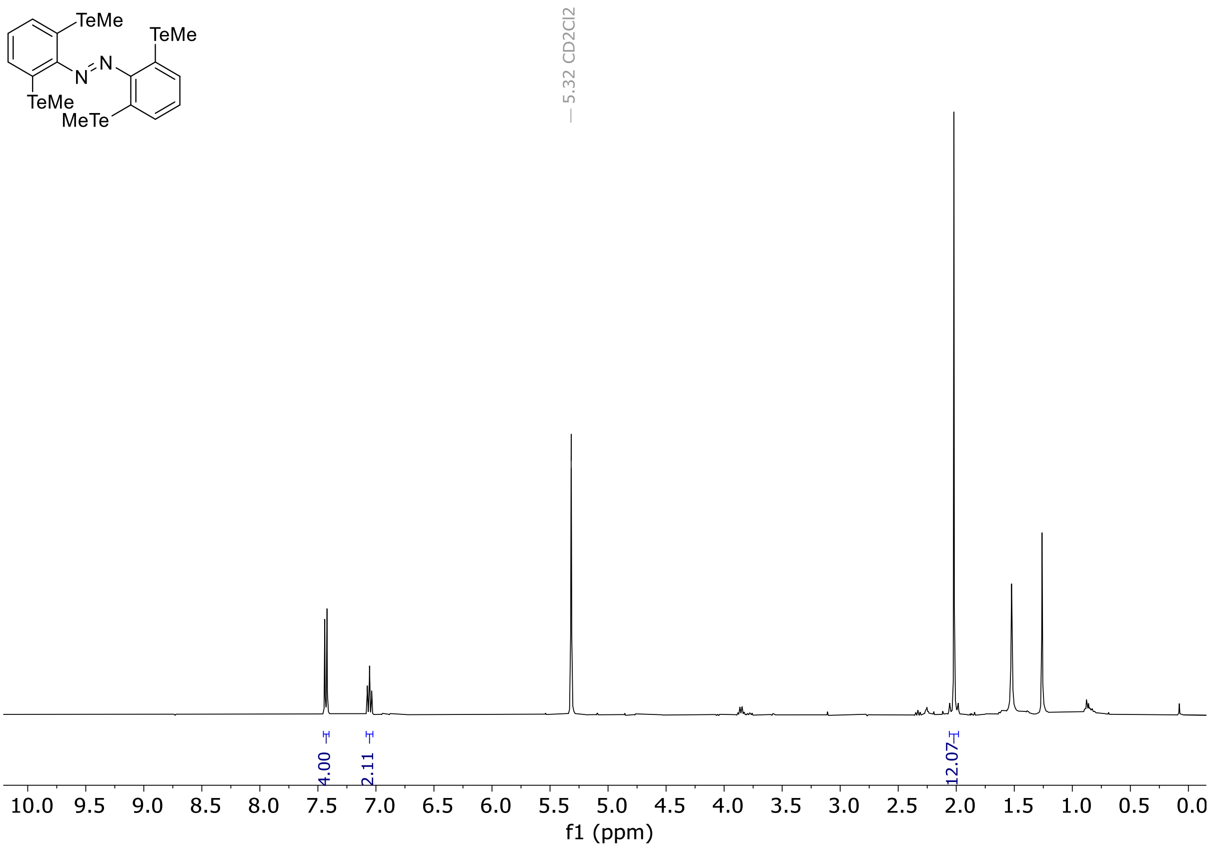

Figure S103. ^{1}H NMR Spectrum of **(TeMe)$_4$-H** (CD_2Cl_2, 298 K).

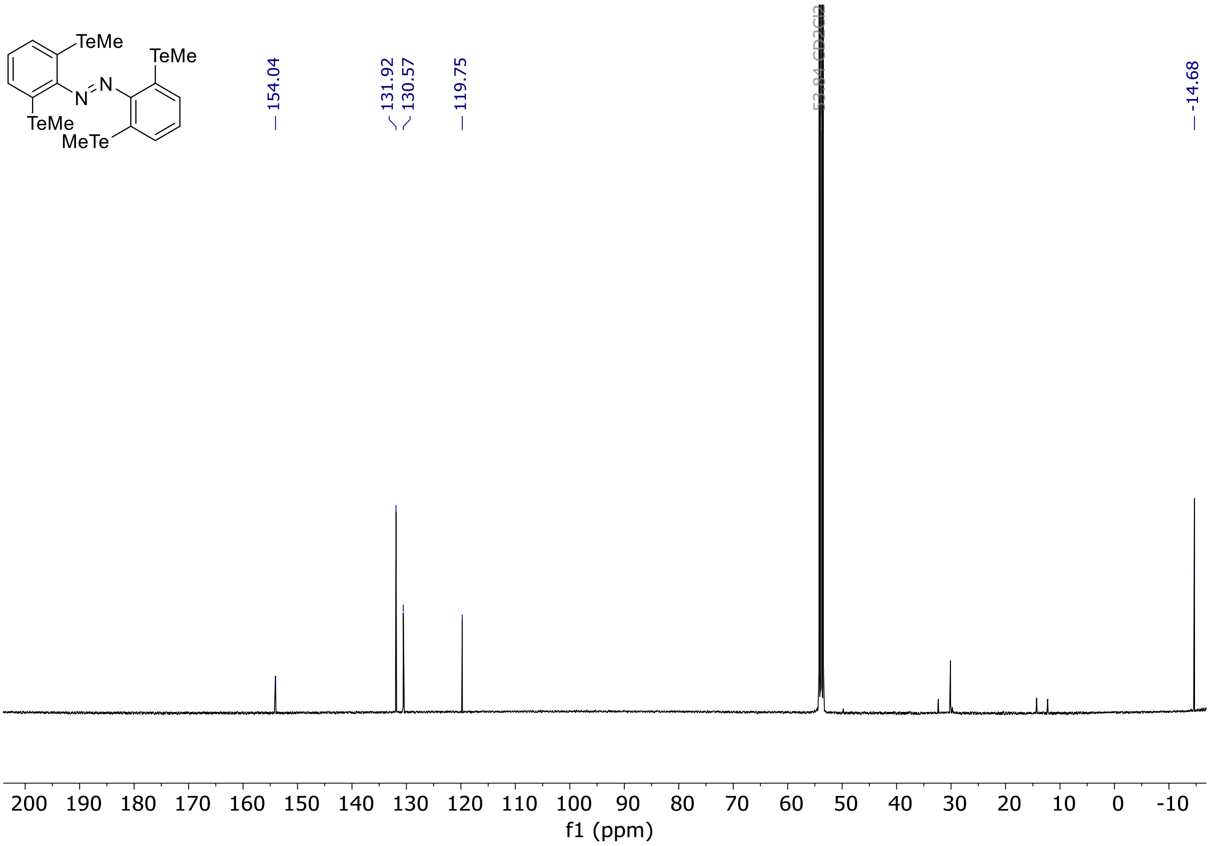

Figure S104. ^{13}C NMR Spectrum of **(TeMe)$_4$-H** (CD_2Cl_2, 298 K).

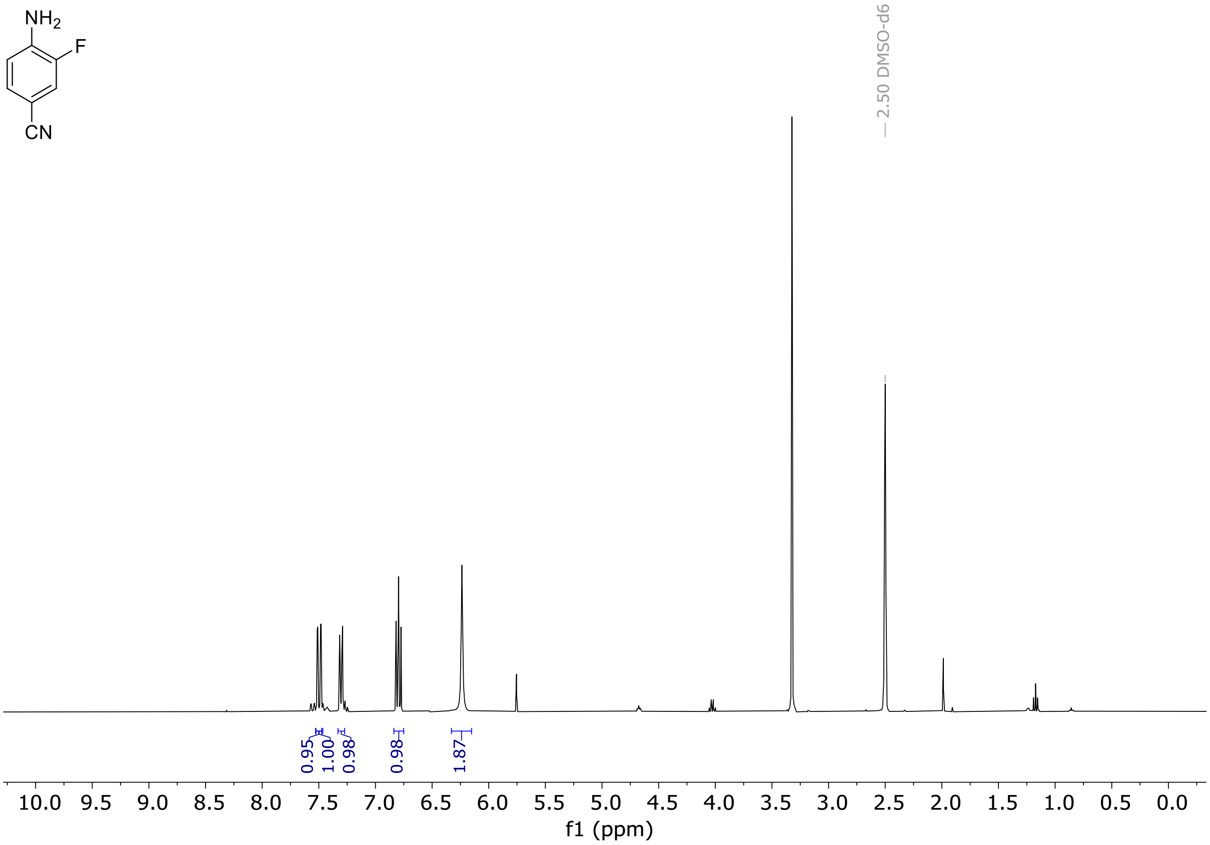

Figure S105. ^{1}H NMR spectrum of **S52** (DMSO-d_6, 298 K).

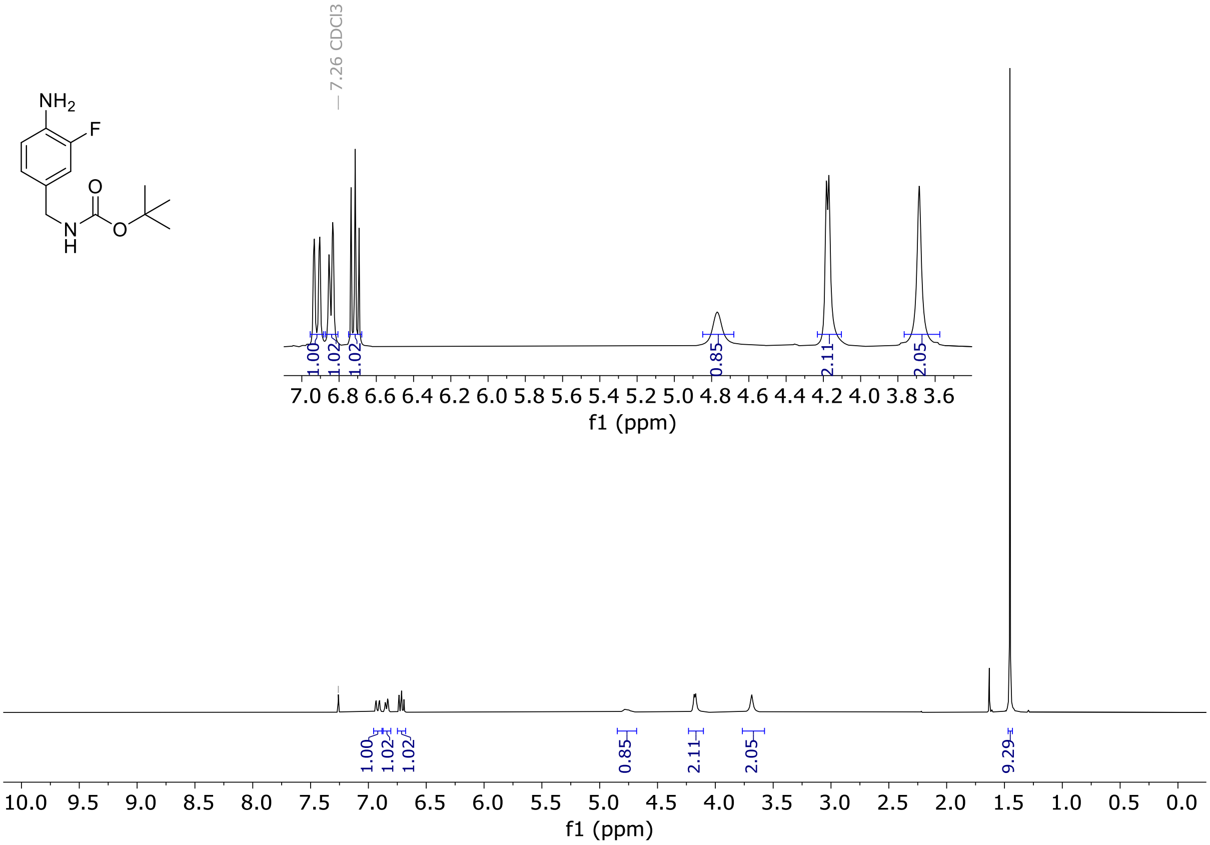

Figure S106. ^{1}H NMR spectrum of **S53** (Chloroform-d, 298 K).

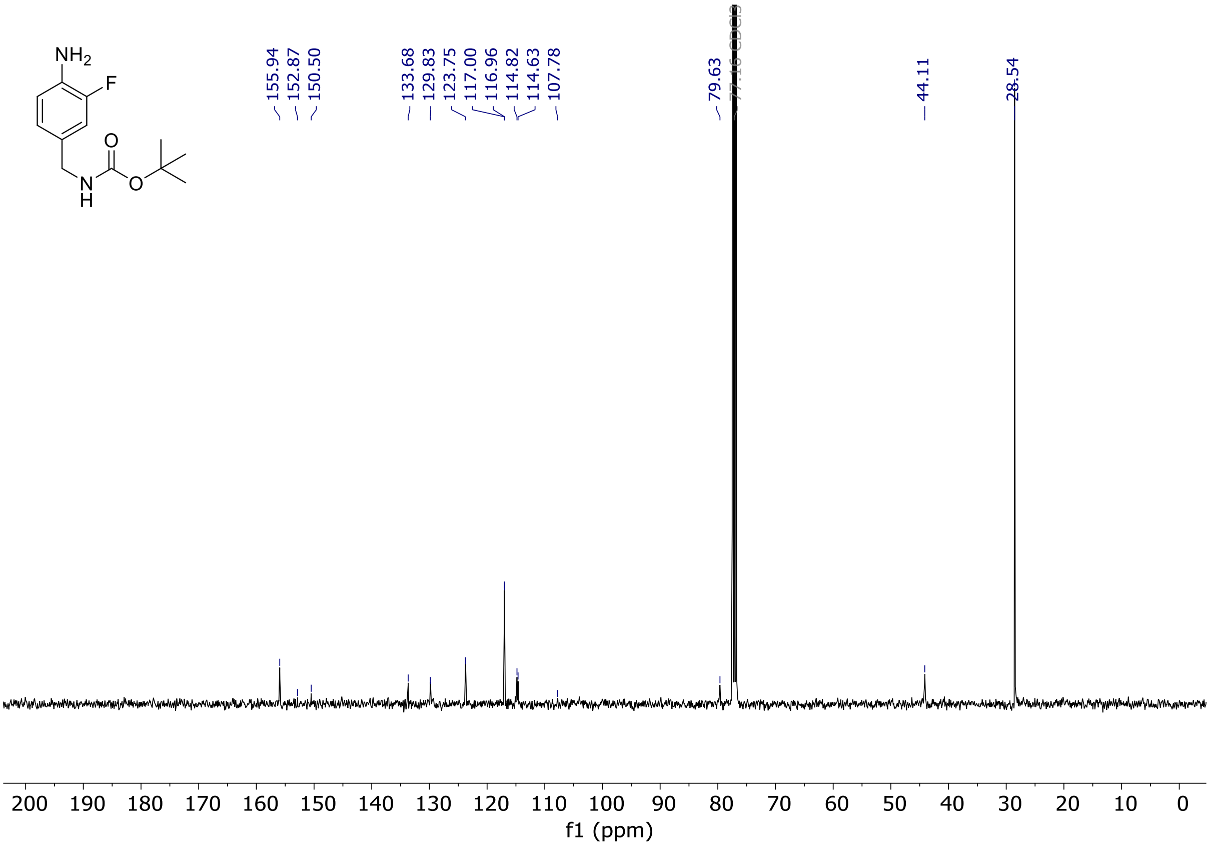

Figure S107. ^{13}C NMR spectrum of **S53**. (Chloroform-*d*, 298 K).

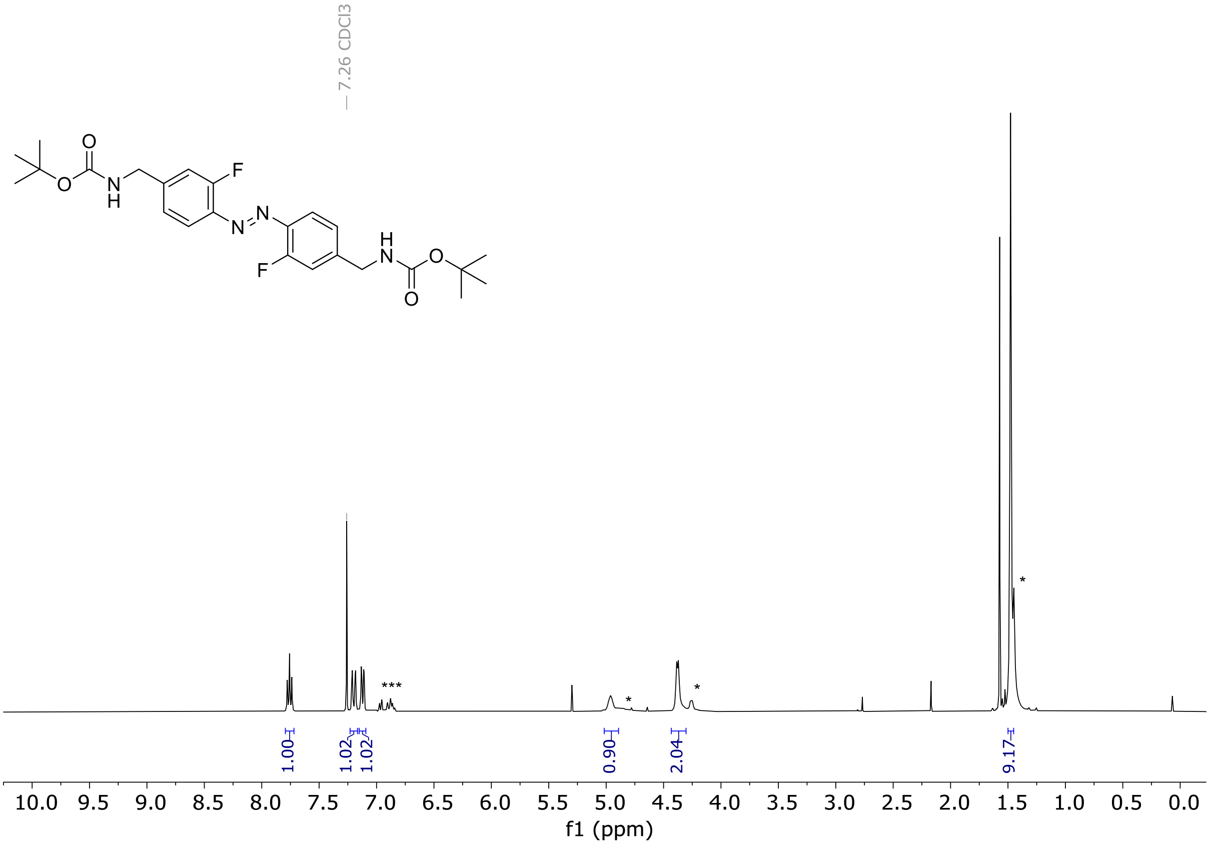

Figure S108. ^{1}H NMR spectrum of **F_2,*m*-H_2** (Chloroform-*d*, 298 K). *Z* signals labelled as *.

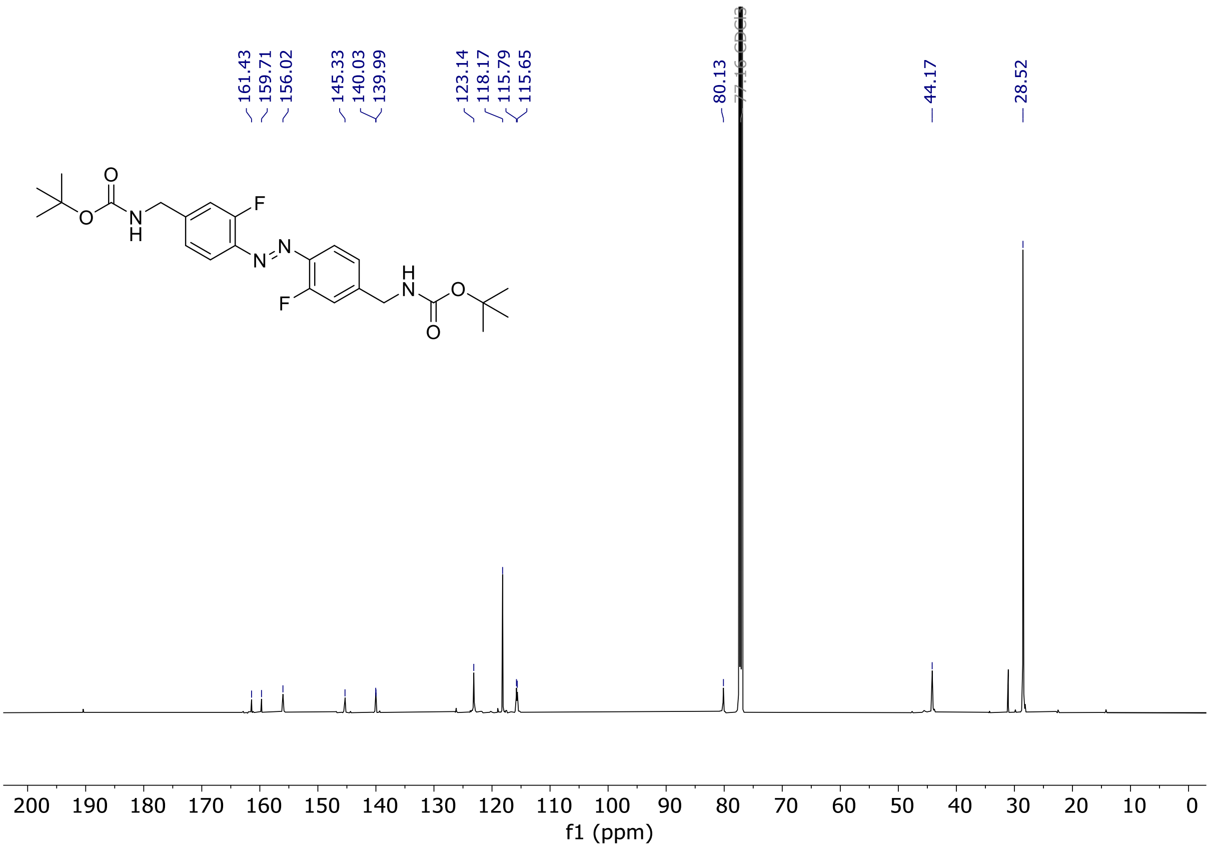

Figure S109. ^{13}C NMR spectrum of **F$_2$,*m*-H$_2$**. (Chloroform-*d*, 298 K).

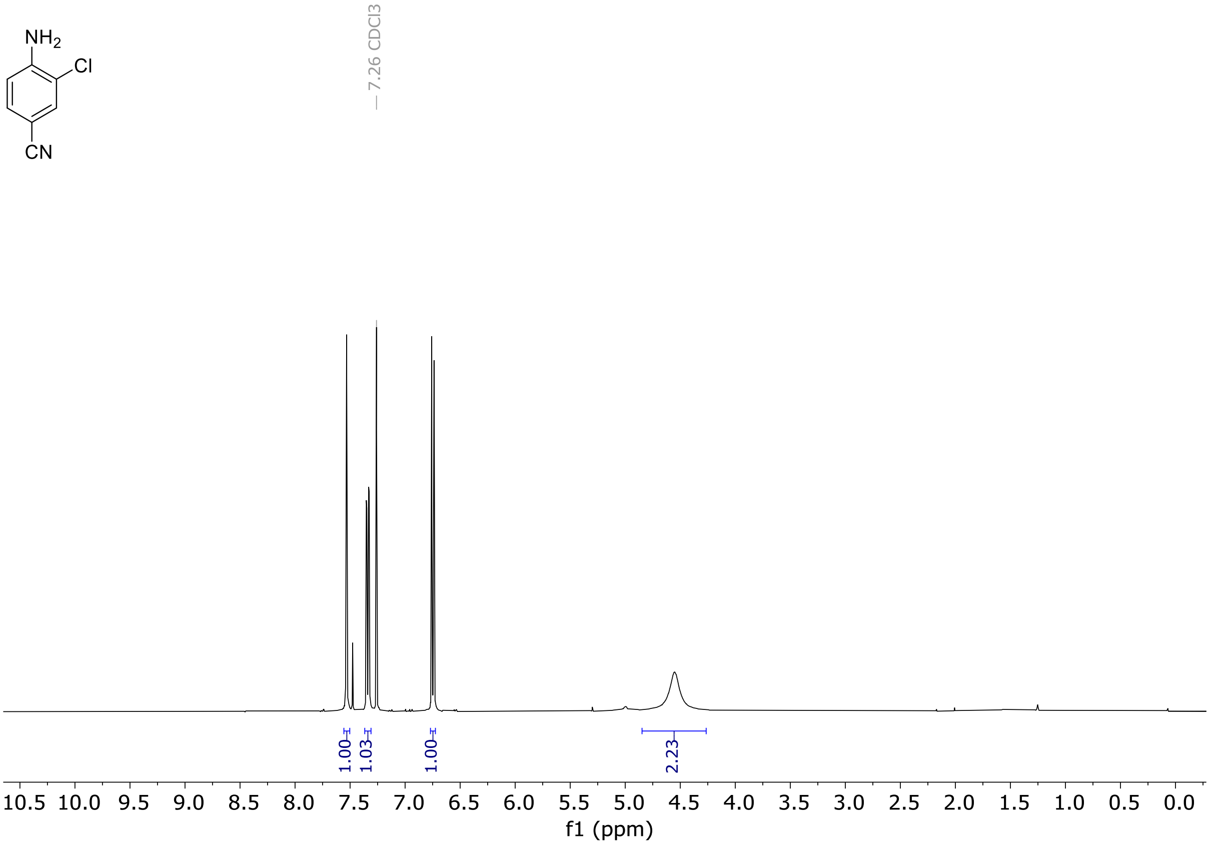

Figure S110. ^{1}H NMR spectrum of **S55** (Chloroform-*d*, 298 K).

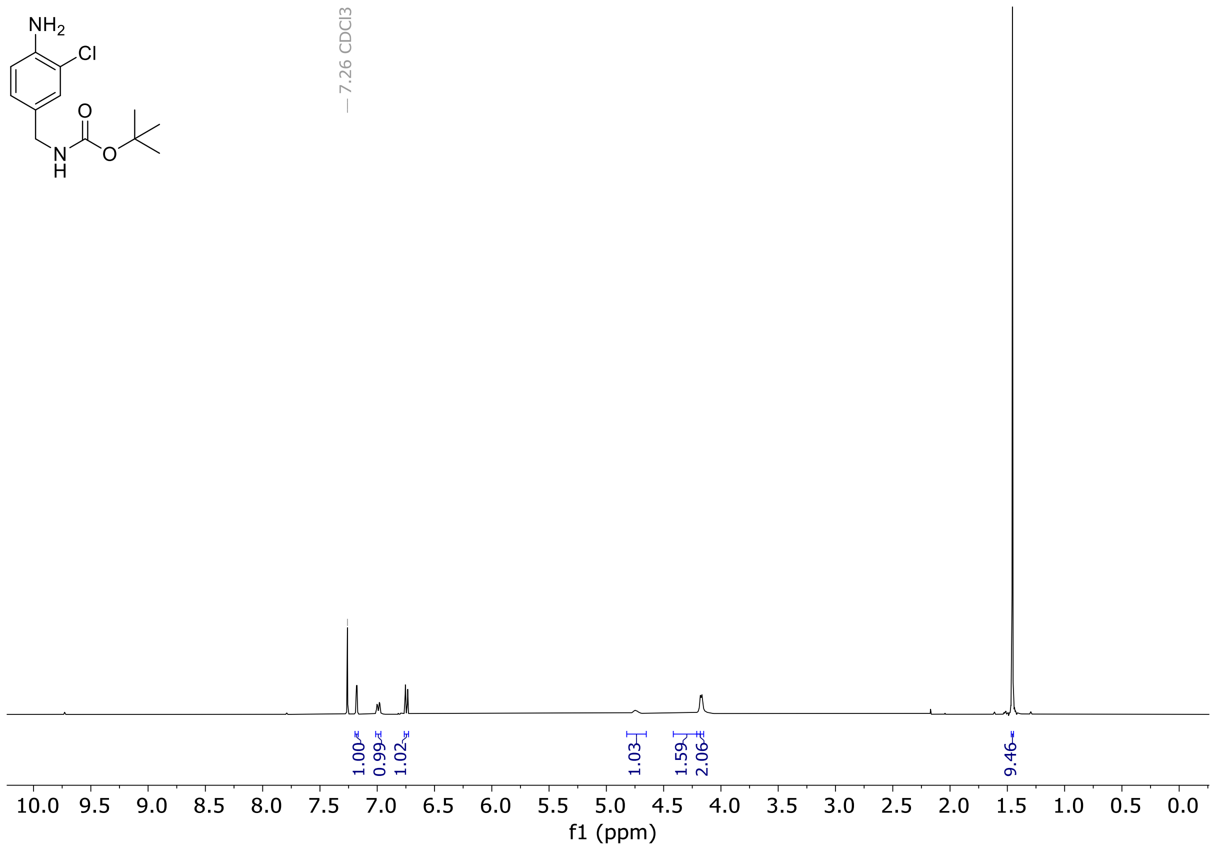

Figure S111. ^{1}H NMR spectrum of **S57** (Chloroform-*d*, 298 K).

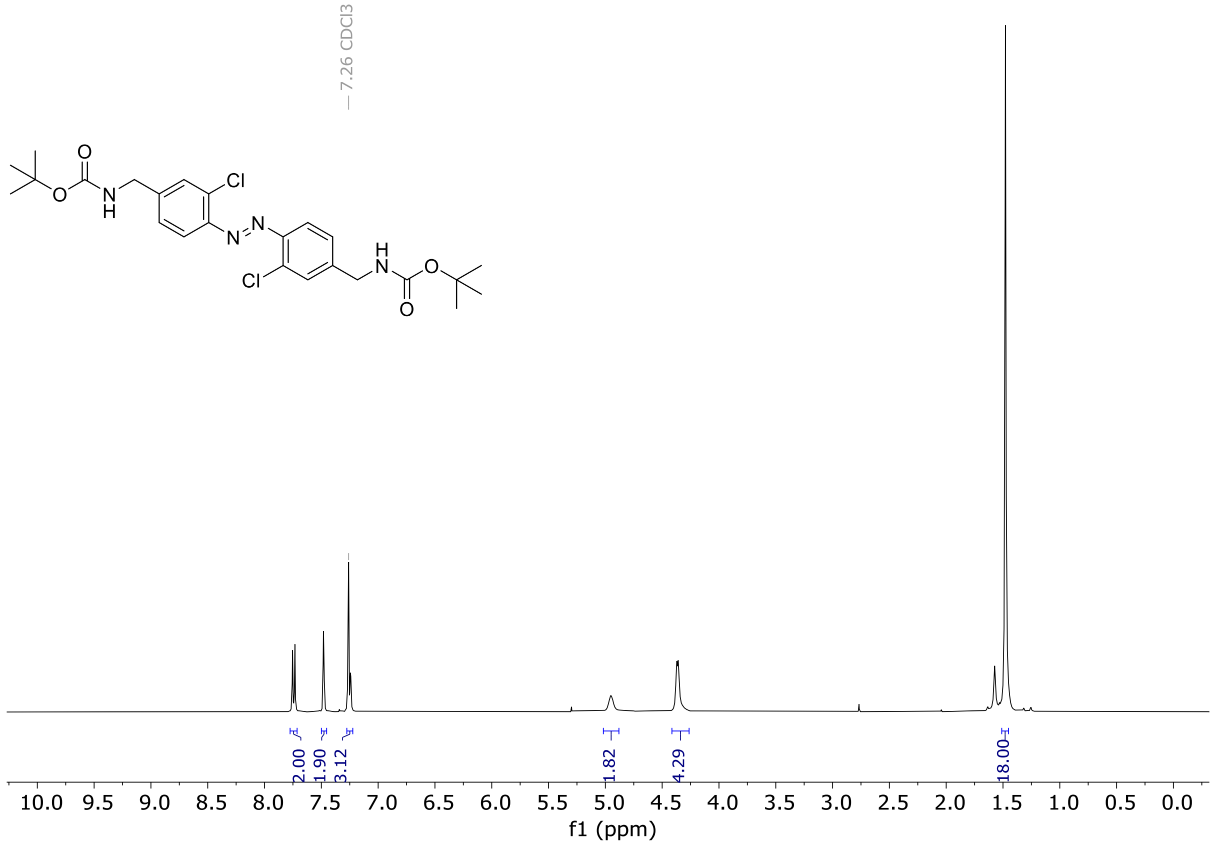

Figure S112. ^{1}H NMR spectrum of **Cl$_2$,*m*-H$_2$** (Chloroform-*d*, 298 K).

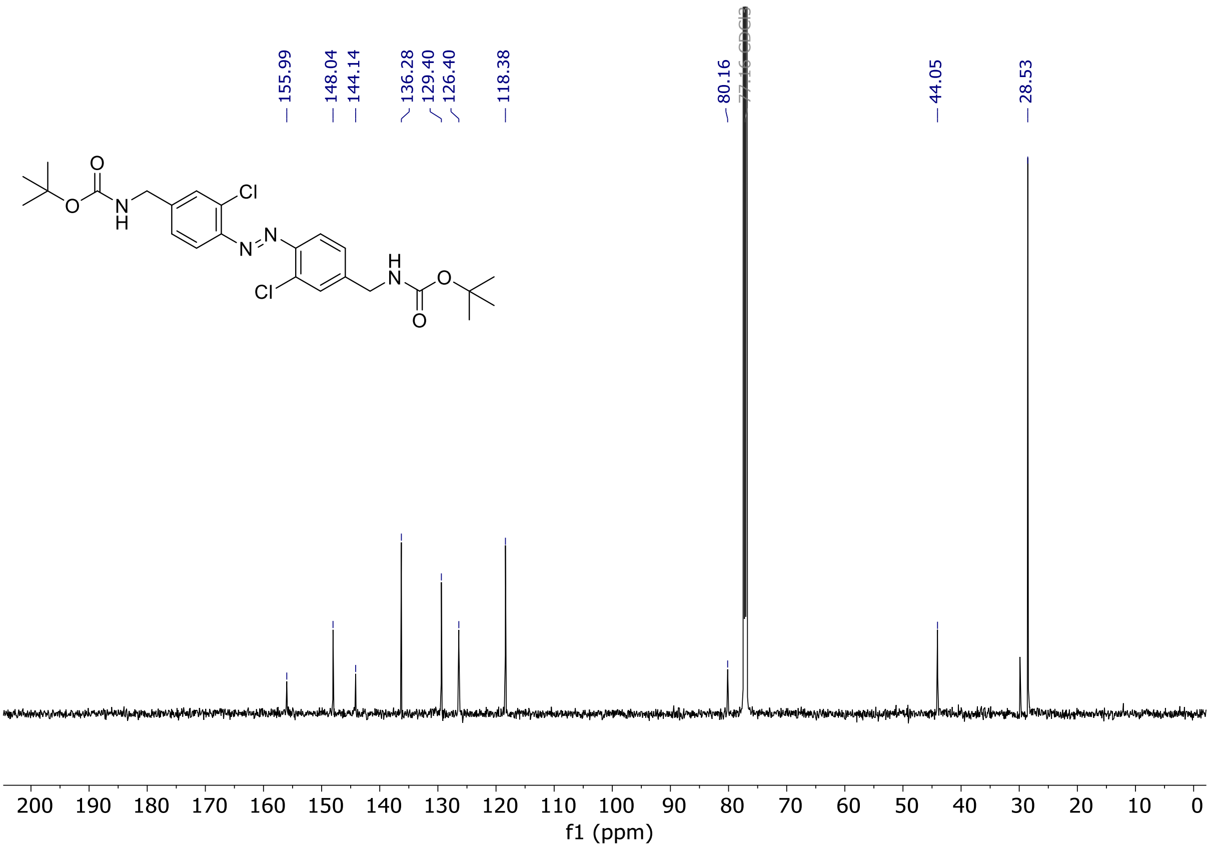

Figure S113. ^{13}C NMR spectrum of **Cl_2,*m*-H_2**. (Chloroform-*d*, 298 K).

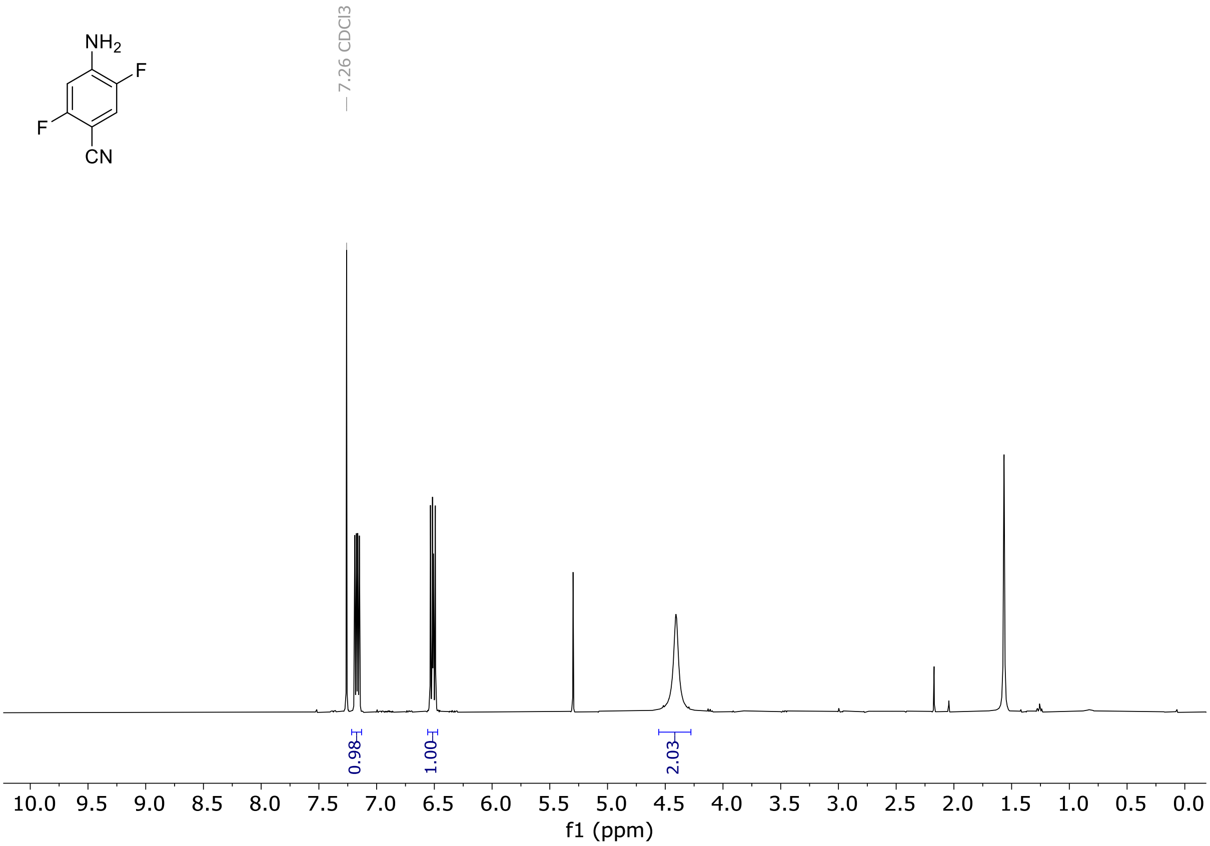

Figure S114. ^{1}H NMR spectrum of **S59** (Chloroform-*d*, 298 K).

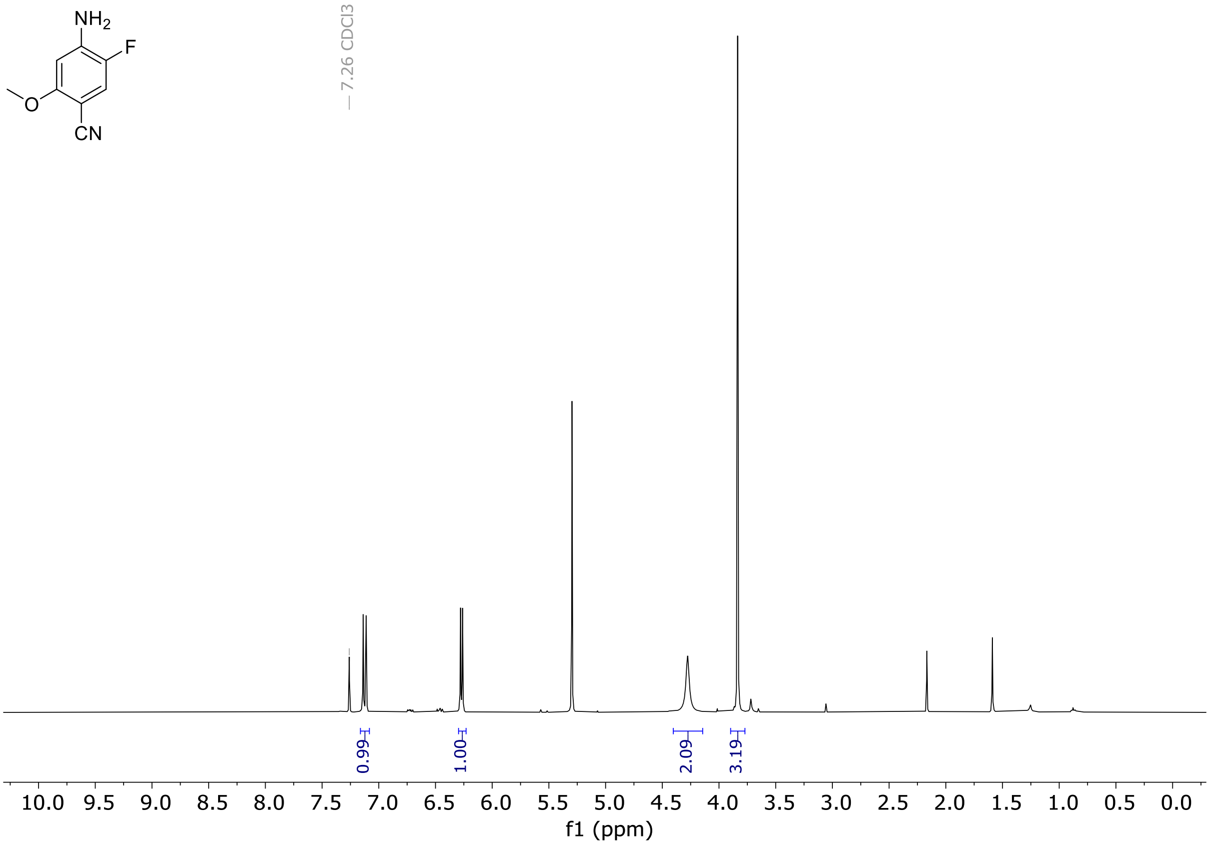

Figure S115. ^{1}H NMR spectrum of **S60** (Chloroform-*d*, 298 K).

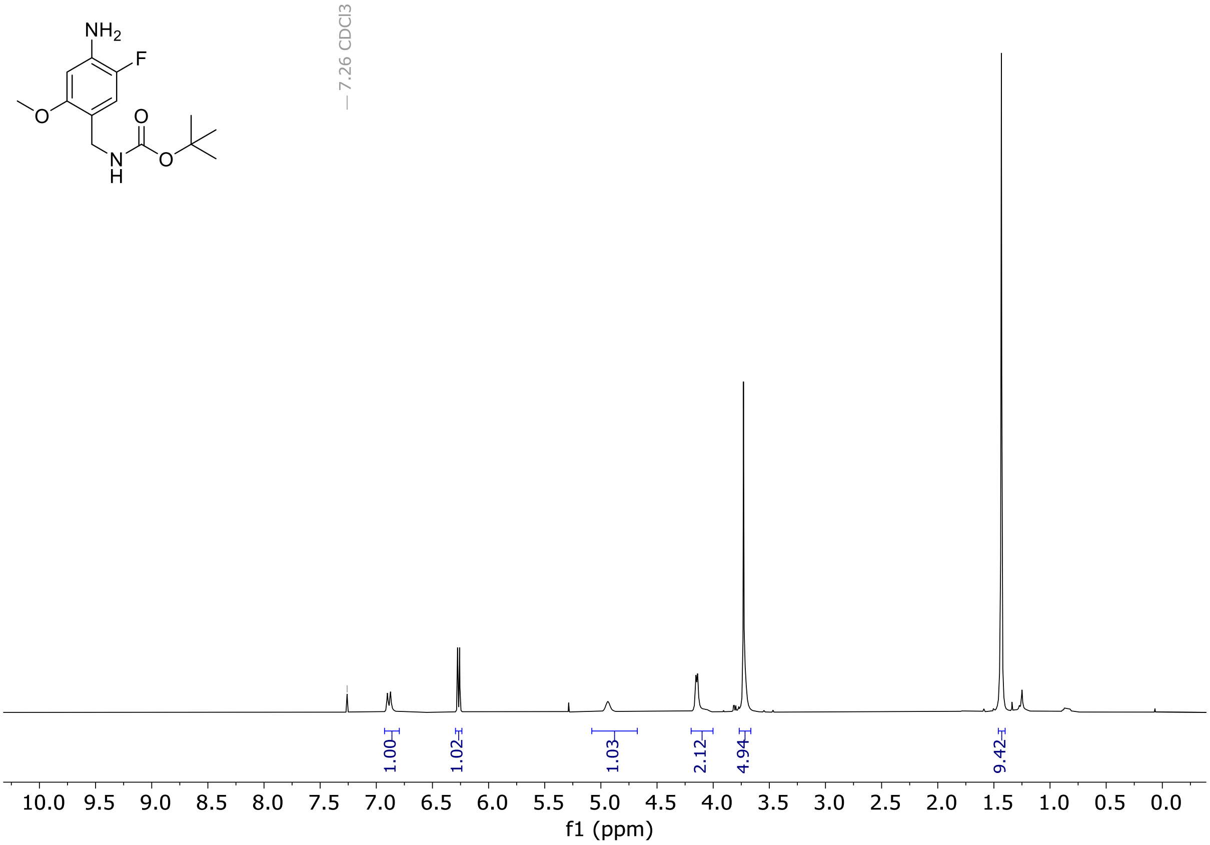

Figure S116. ^{1}H NMR spectrum of **S70a** (Chloroform-*d*, 298 K).

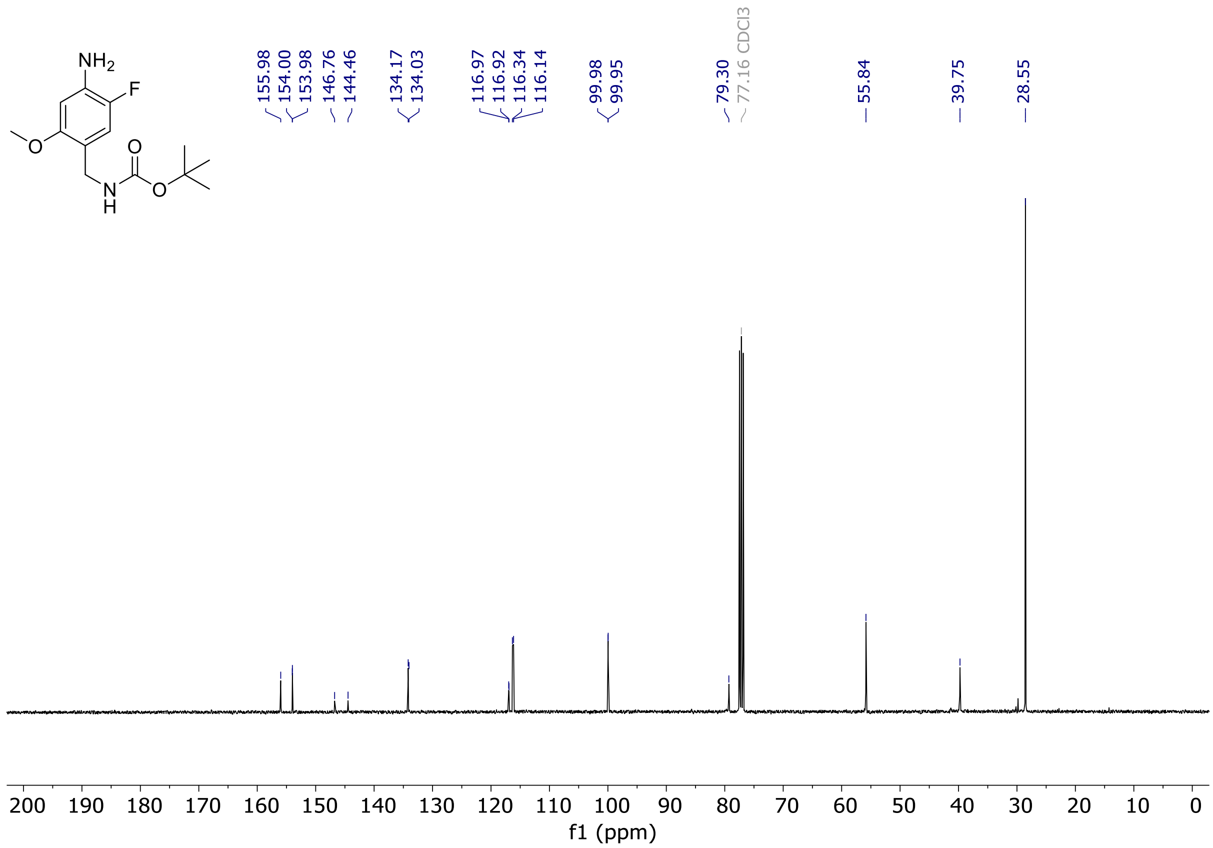

Figure S117. ^{13}C NMR spectrum of **s70a** (Chloroform-*d*, 298 K).

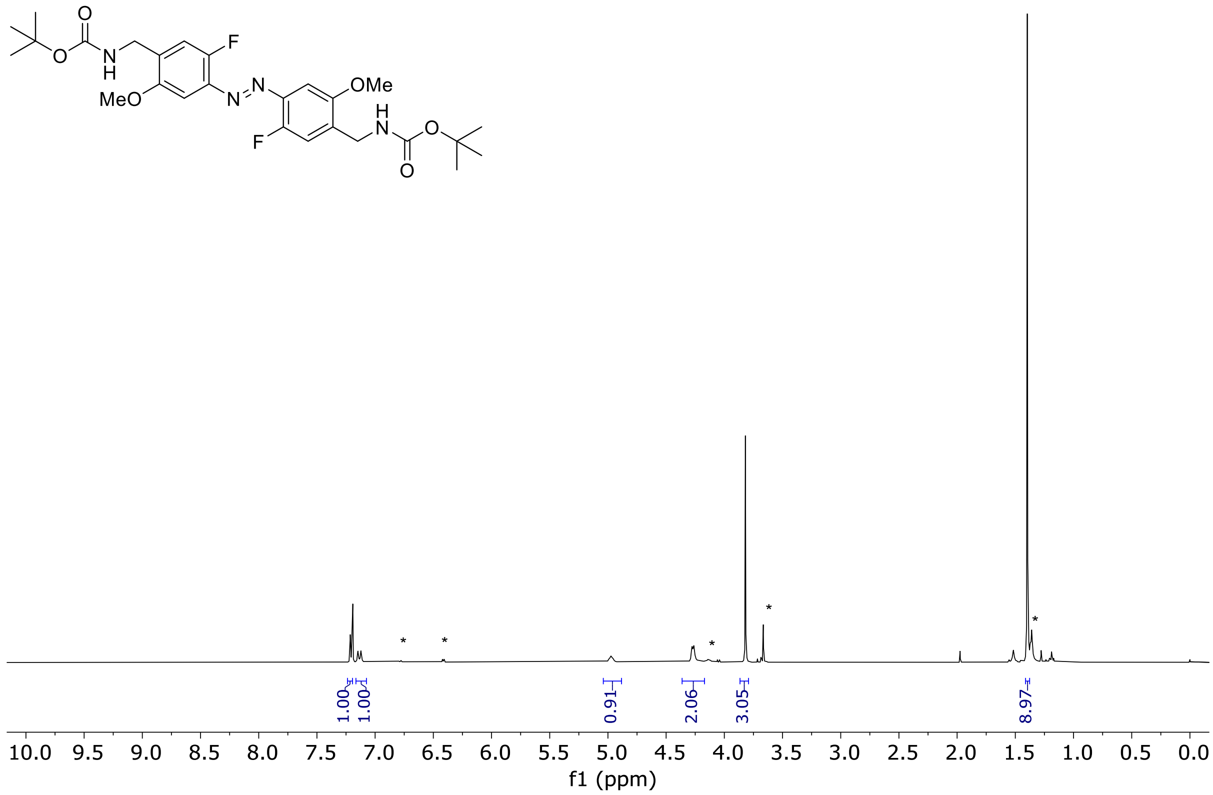

Figure S118. ^{1}H NMR spectrum of **F$_2$,*m*-OMe$_2$** (Chloroform-*d*, 298 K).

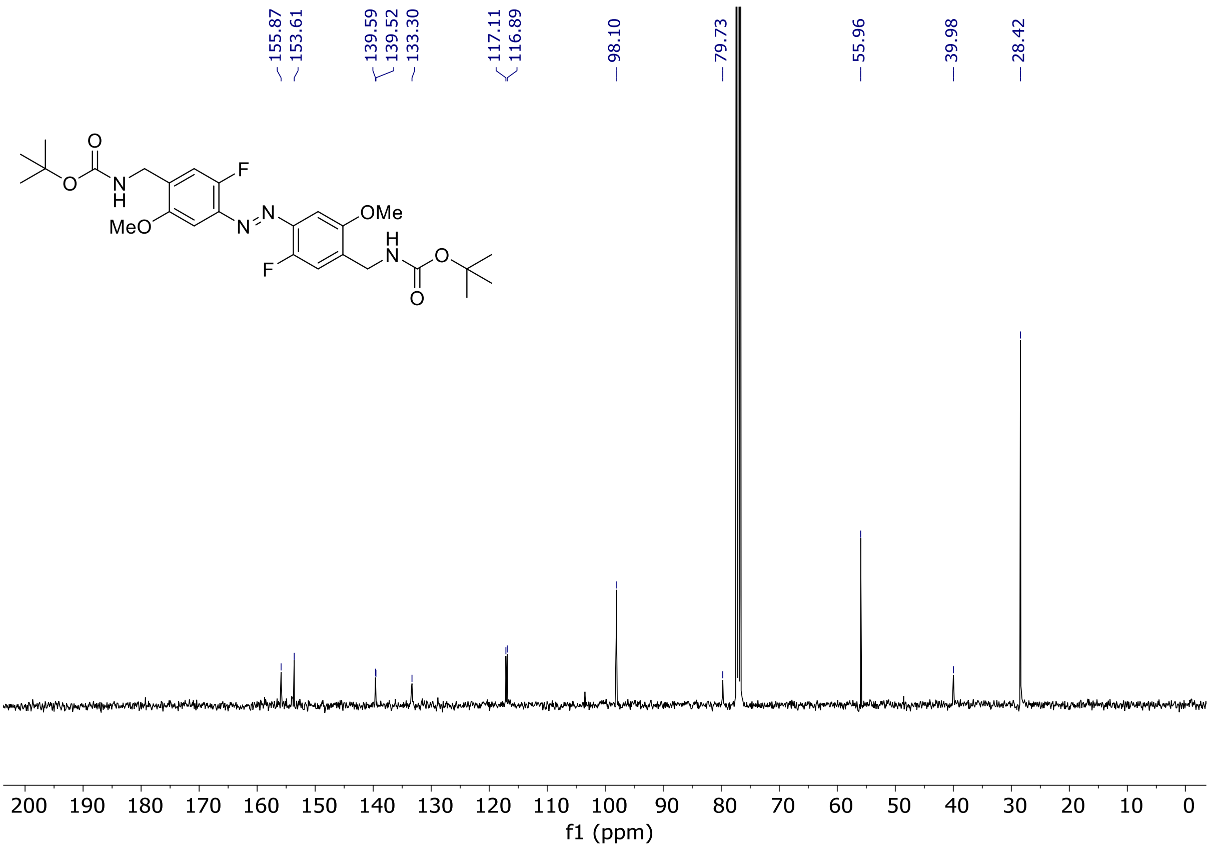

Figure S119. ^{13}C NMR spectrum of **F_2,*m*-OMe$_2$** (Chloroform-*d*, 298 K).

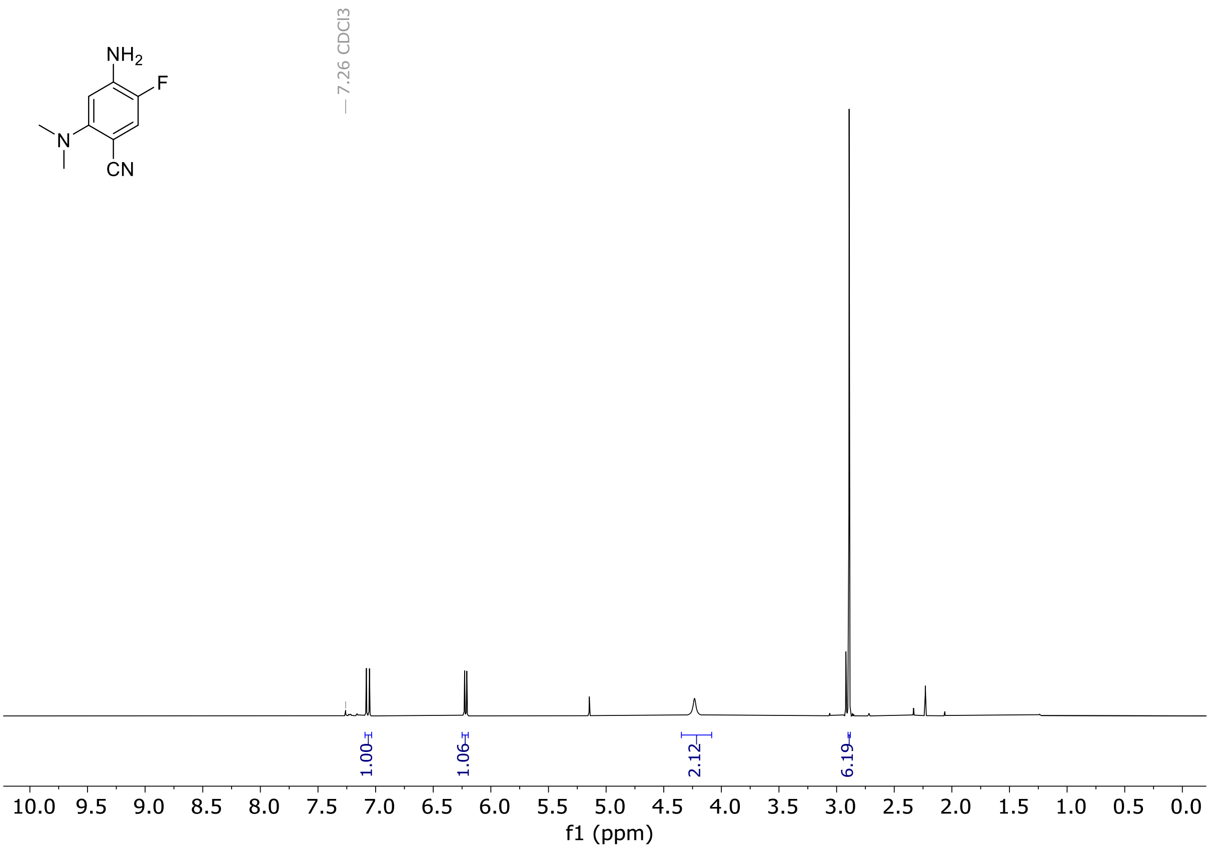

Figure S120. ^{1}H NMR spectrum of **S61** (Chloroform-*d*, 298 K).

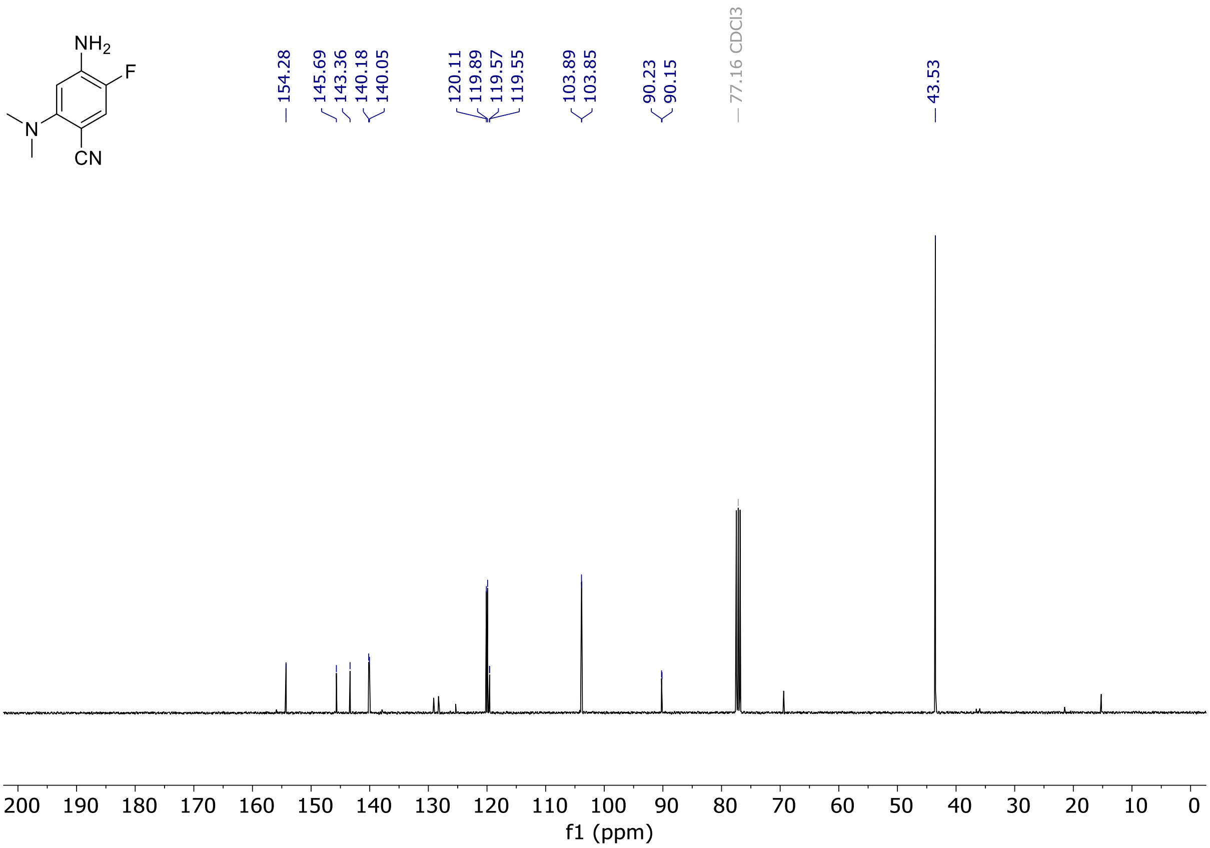

Figure S121. ^{13}C NMR spectrum of **S61** (Chloroform-*d*, 298 K).

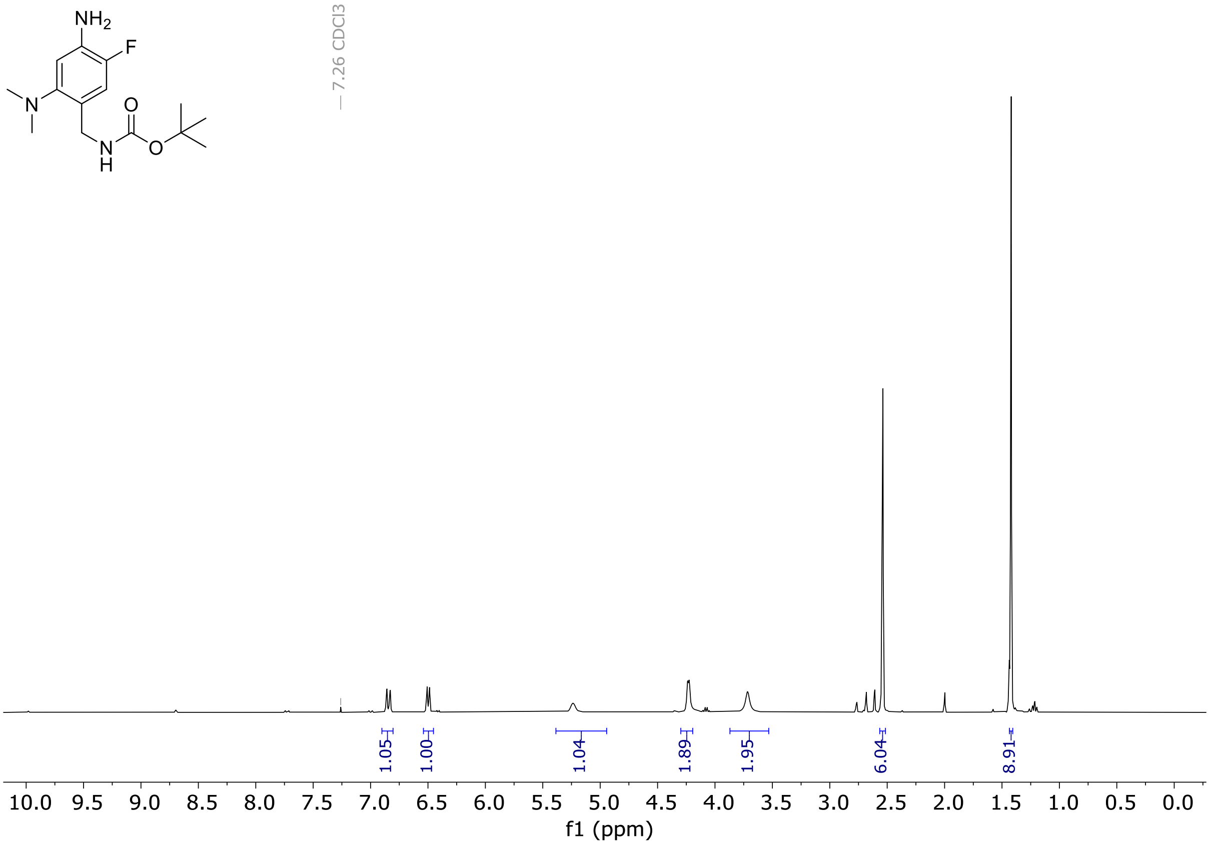

Figure S122. ^{1}H NMR spectrum of **S70b** (Chloroform-*d*, 298 K).

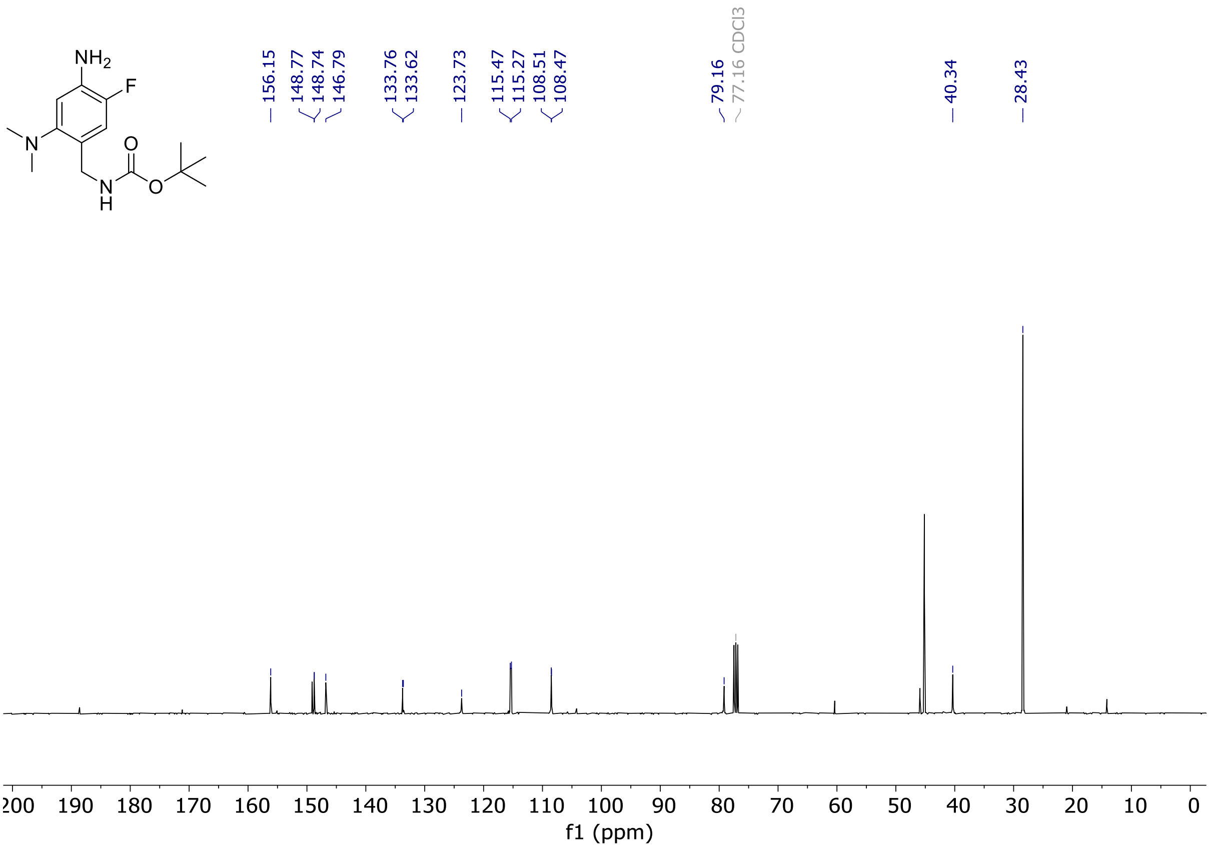

Figure S123. ^{13}C NMR spectrum of **S70b** (Chloroform-*d*, 298 K).

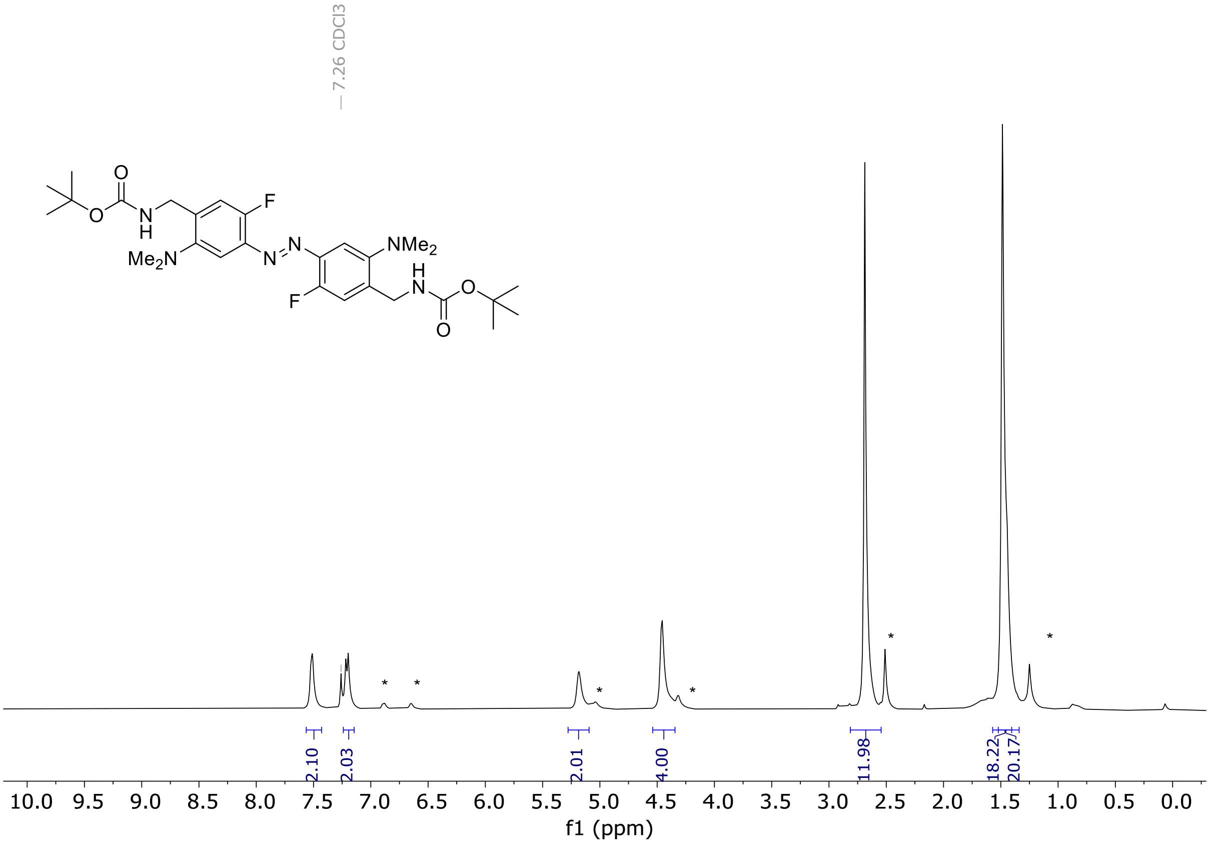

Figure S124. ^{1}H NMR spectrum of **F$_2$,*m*-(NMe$_2$)$_2$** (Chloroform-*d*, 298 K). (*Z)* signals labelled as *.

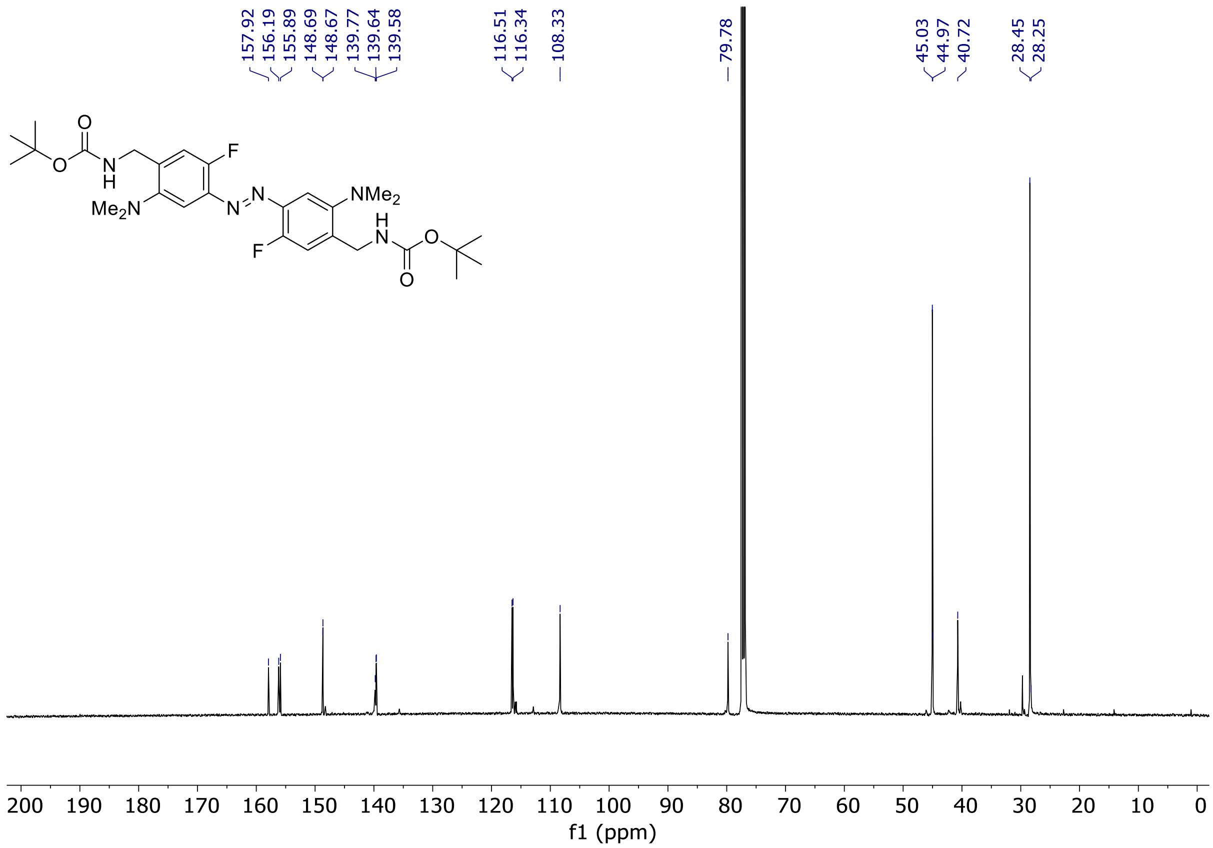

Figure S125. ^{13}C NMR spectrum of **F$_2$,*m*-(NMe$_2$)$_2$**. (Chloroform-*d*, 298 K).

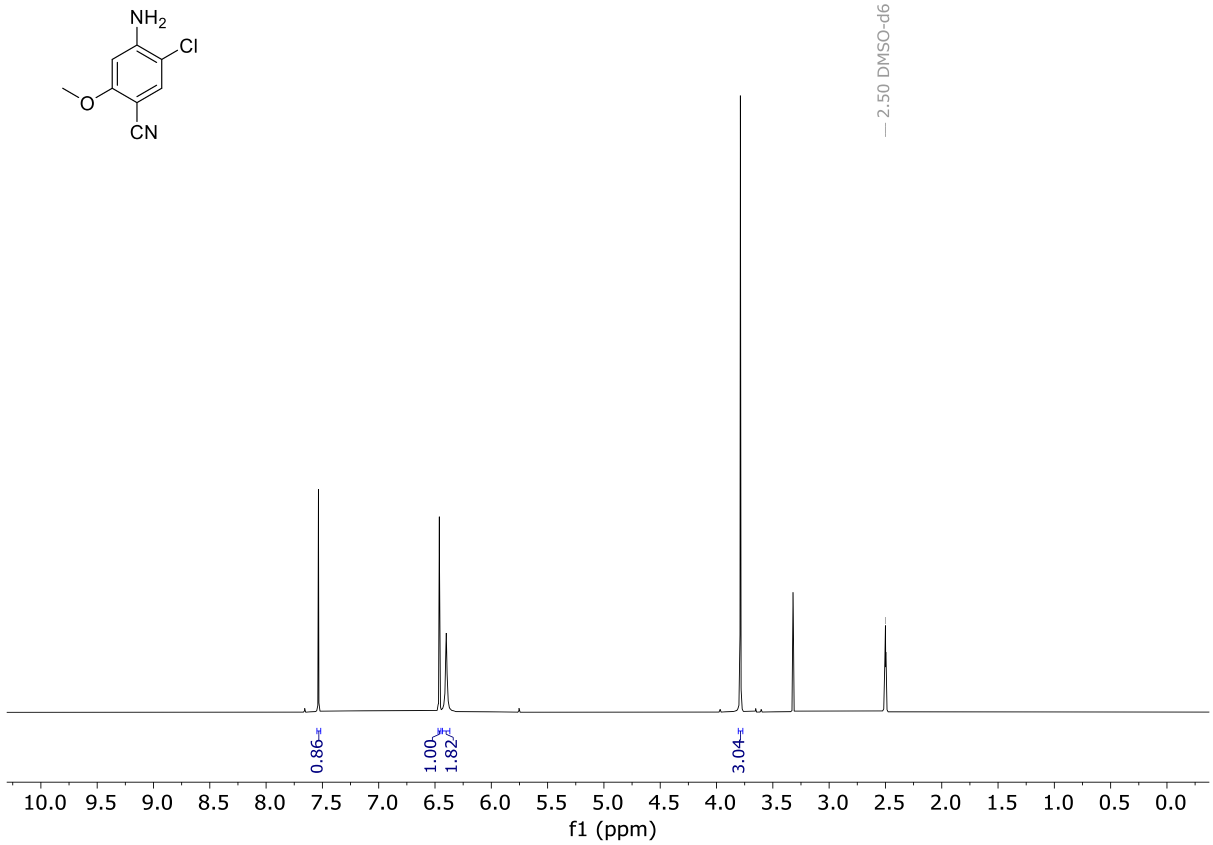

Figure S126. ^{1}H NMR spectrum of **S64** (DMSO-*d$_6$*, 298 K).

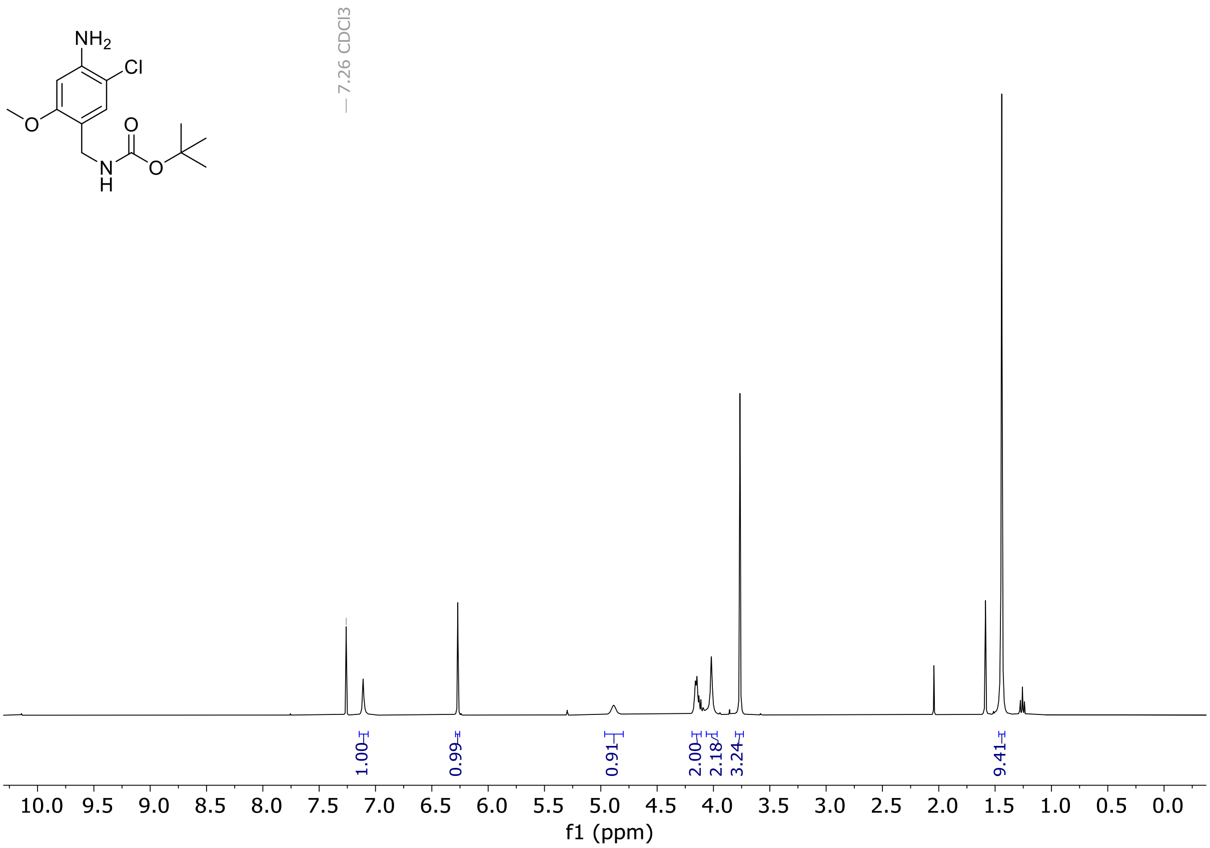

Figure S127. ^{1}H NMR spectrum of **S70c** (Chloroform-*d*, 298 K).

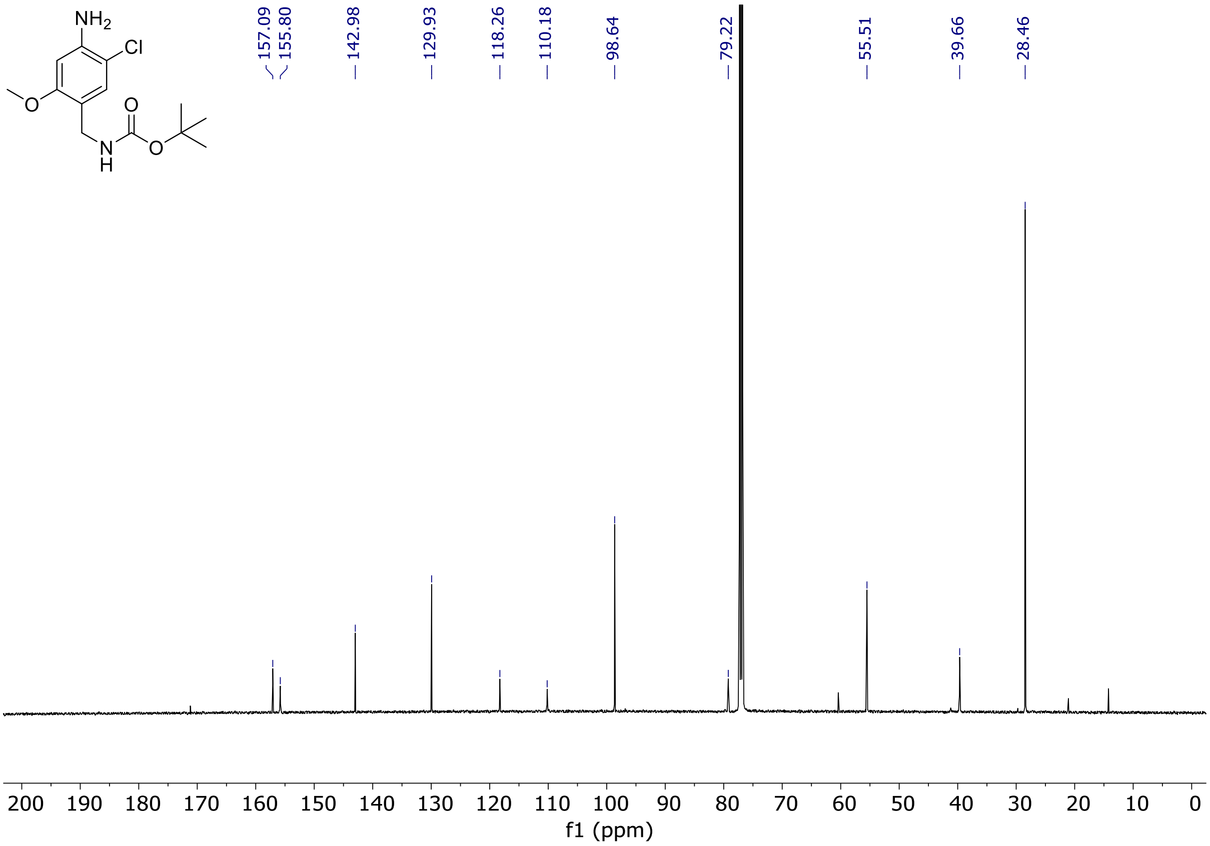

Figure S128. ^{13}C NMR spectrum of **S70c**. (Chloroform-*d*, 298 K).

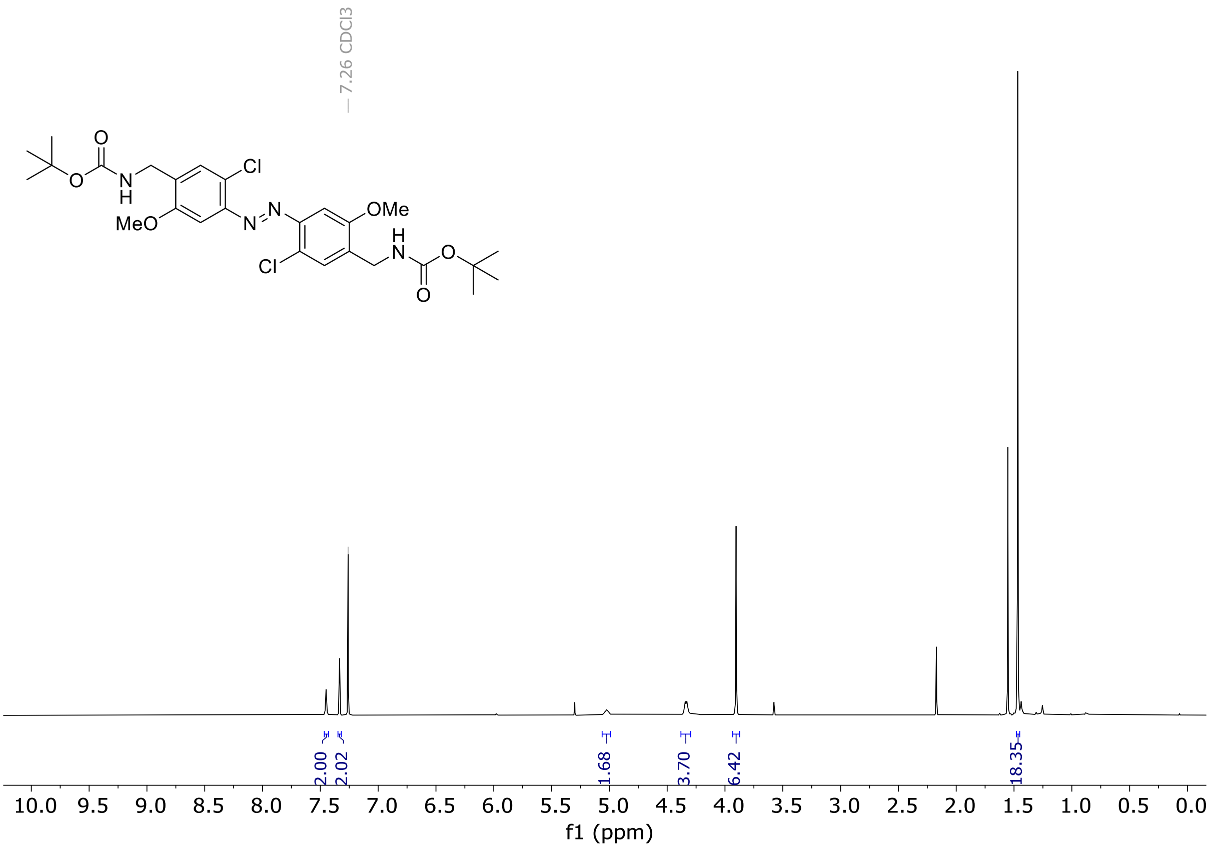

Figure S129. ^{1}H NMR spectrum of **Cl$_2$,*m*-OMe$_2$** (Chloroform-*d*, 298 K).

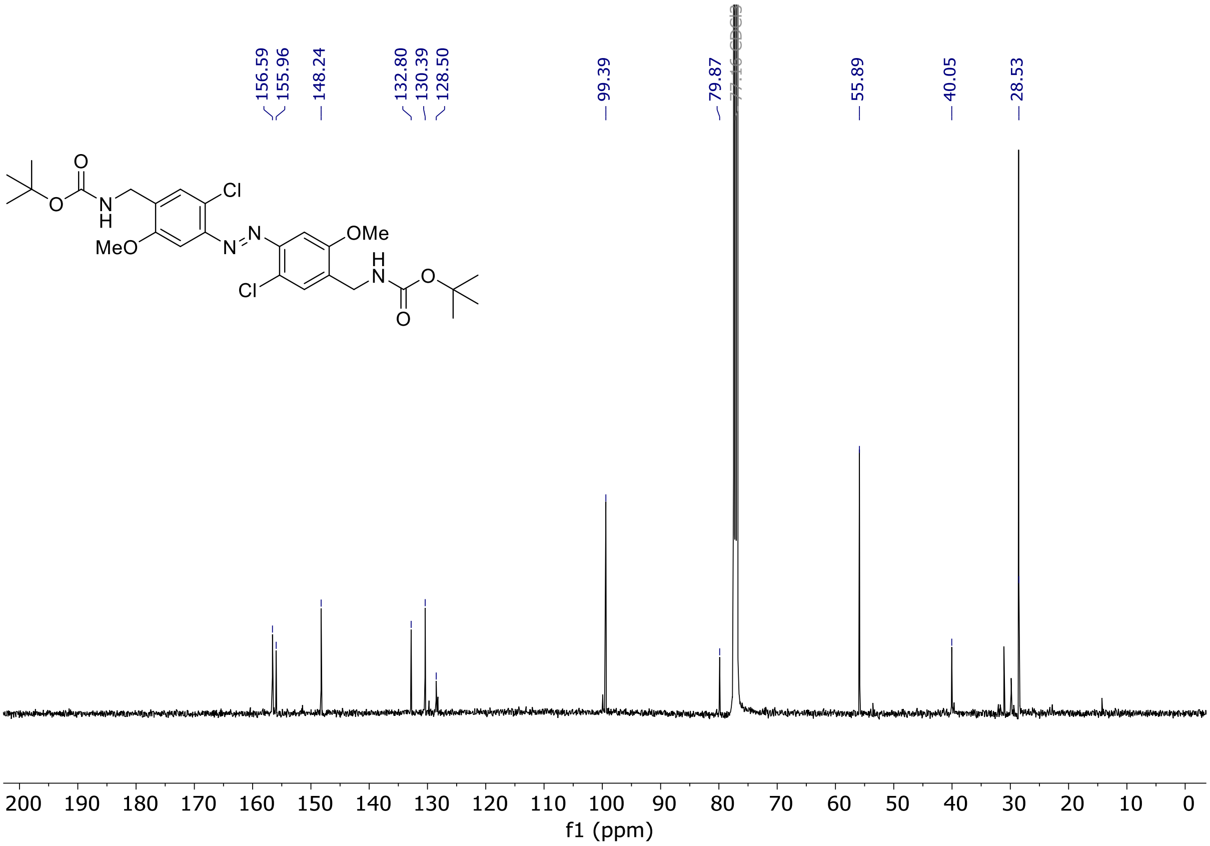

Figure S130. ^{13}C NMR spectrum of **Cl$_2$,*m*-OMe$_2$**. (Chloroform-*d*, 298 K).

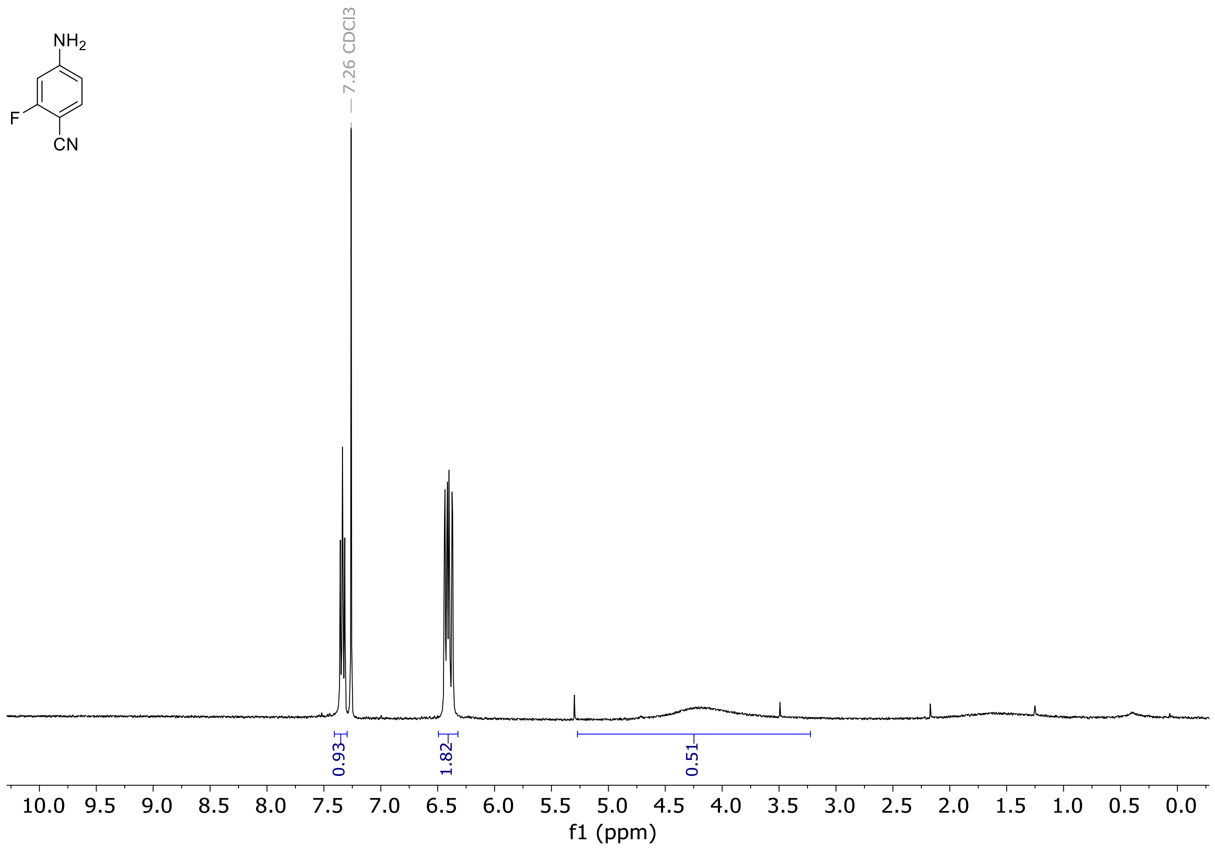

Figure S131. ^{1}H NMR spectrum of **S66** (Chloroform-*d*, 298 K).

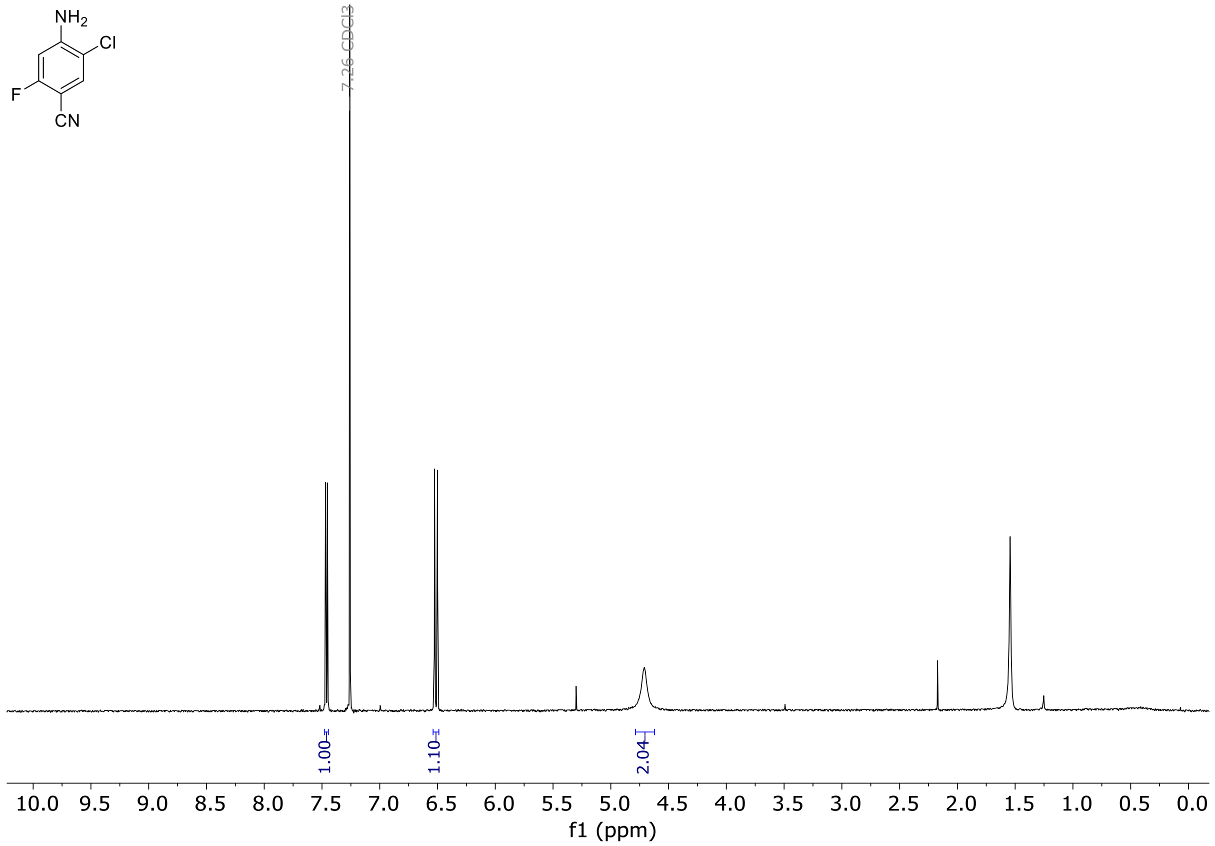

Figure S132. ^{1}H NMR spectrum of **S67** (Chloroform-*d*, 298 K).

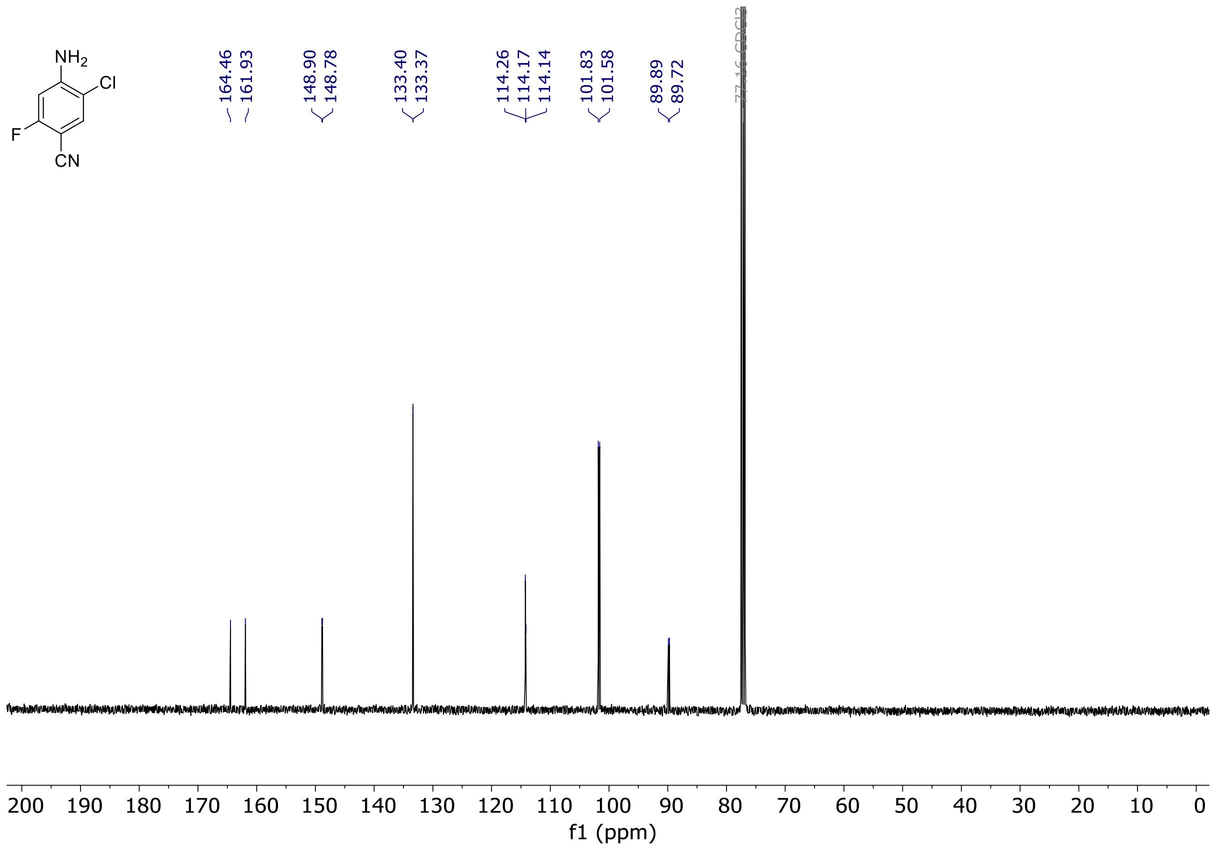

Figure S133. ^{13}C NMR spectrum of **S67**. (Chloroform-*d*, 298 K).

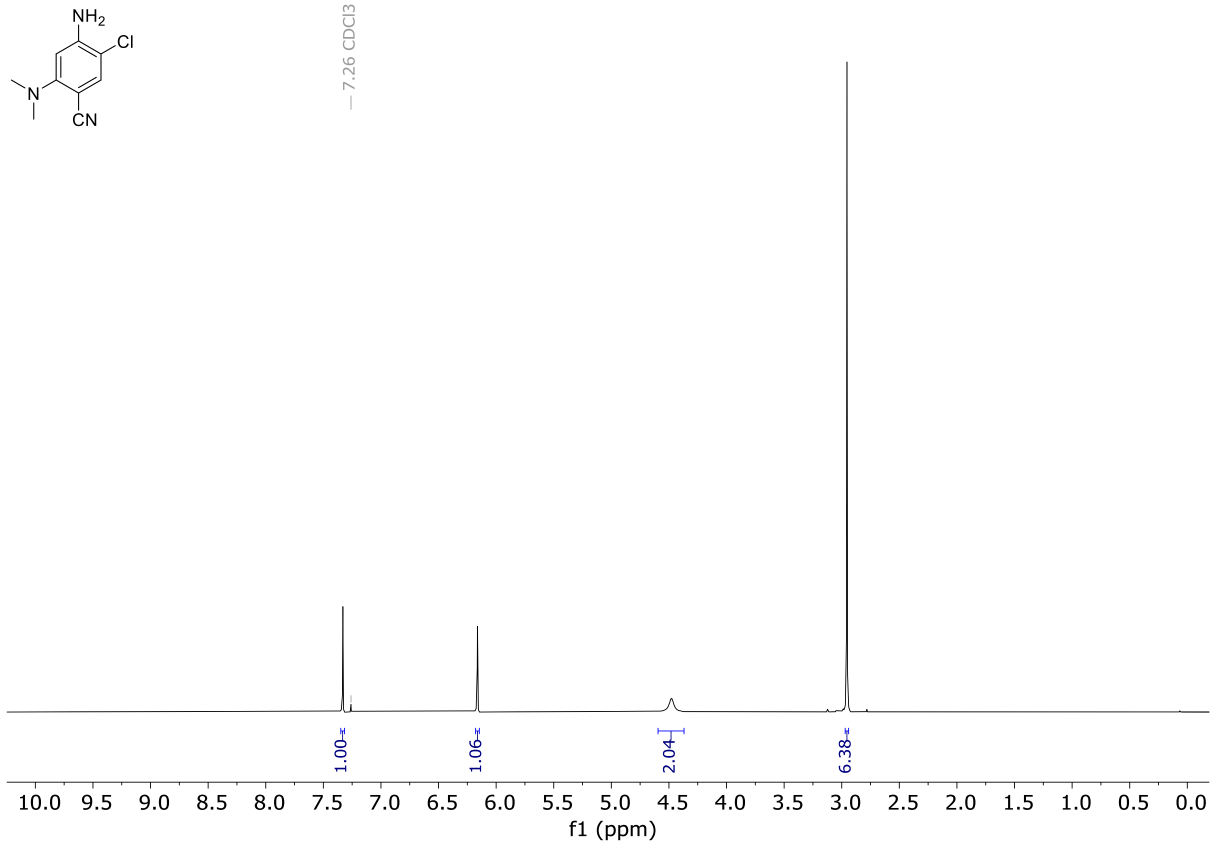

Figure S134. ^{1}H NMR spectrum of **S68** (Chloroform-*d*, 298 K).

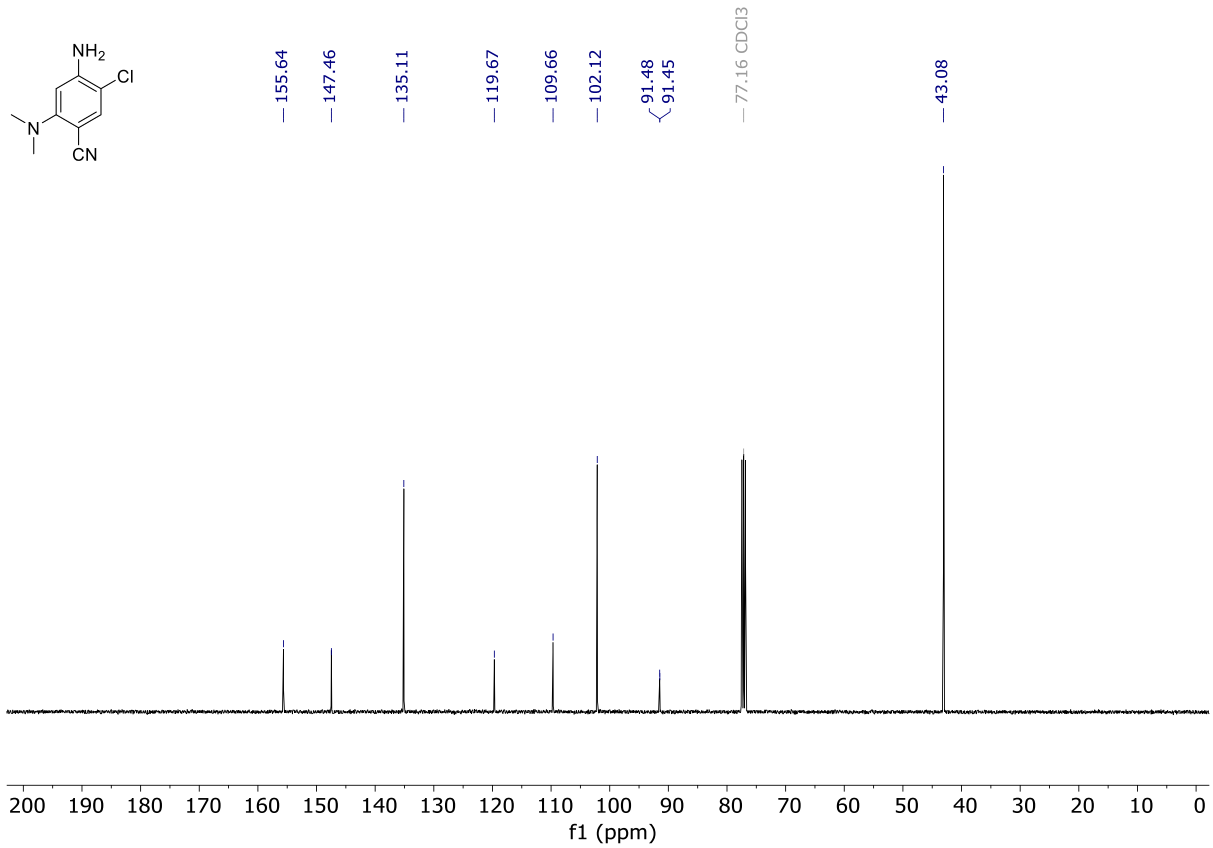

Figure S135. ^{13}C NMR spectrum of **S68**. (Chloroform-*d*, 298 K).

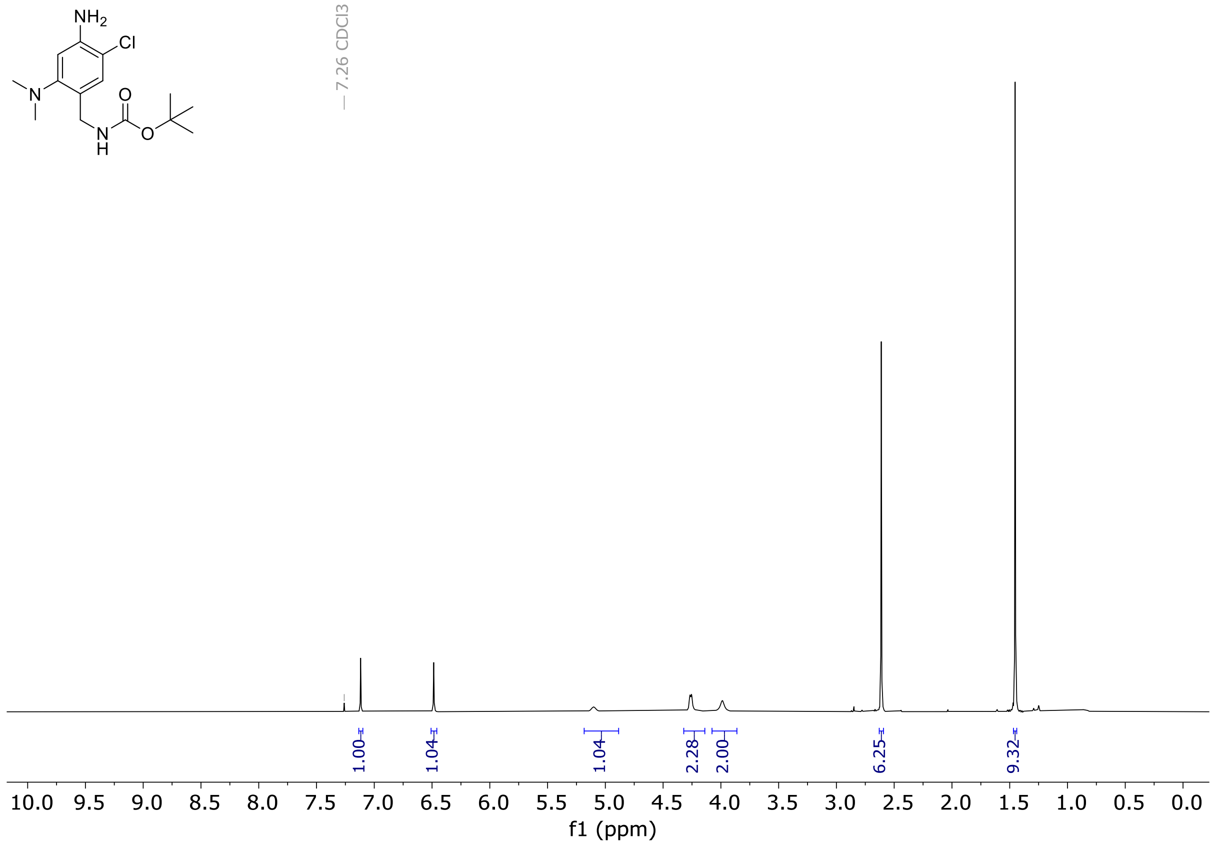

Figure S136. ^{1}H NMR spectrum of **S70d** (Chloroform-*d*, 298 K).

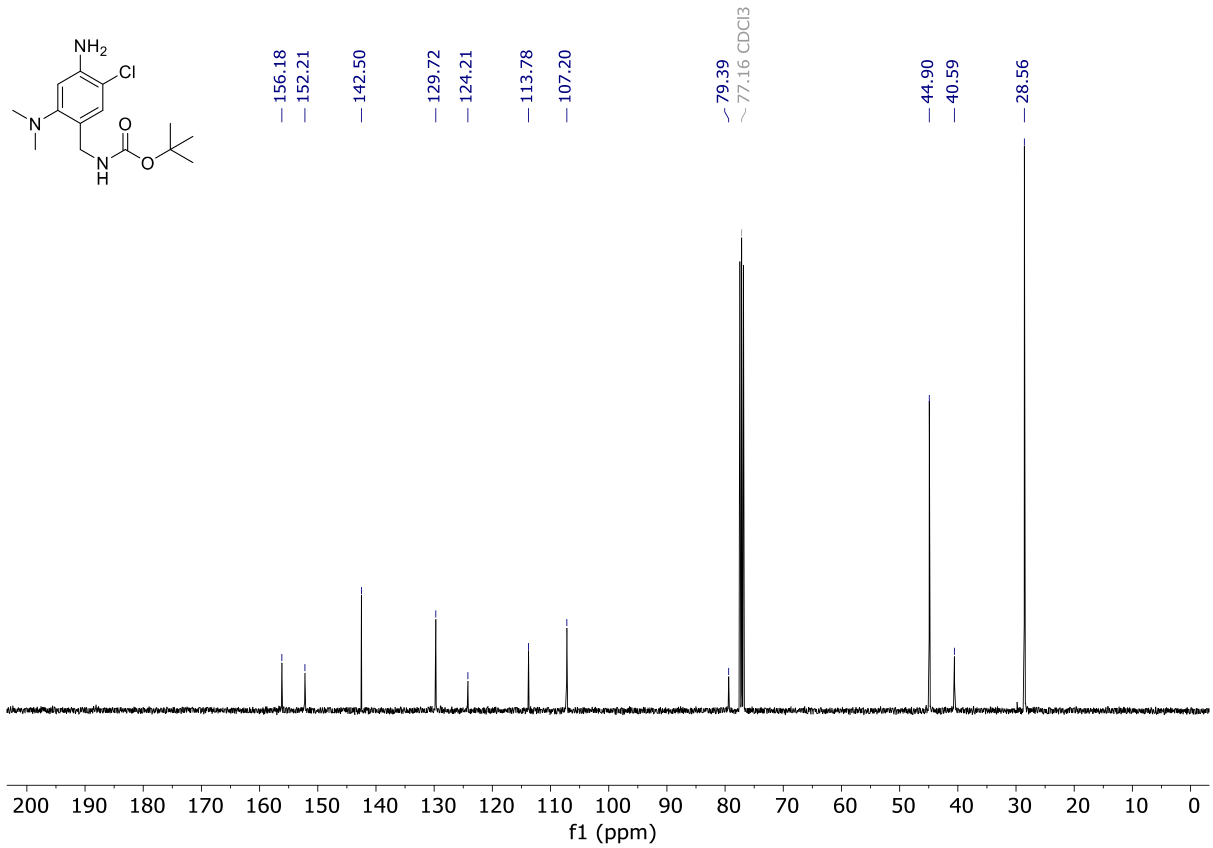

Figure S137. ^{13}C NMR spectrum of **S70d**. (Chloroform-*d*, 298 K).

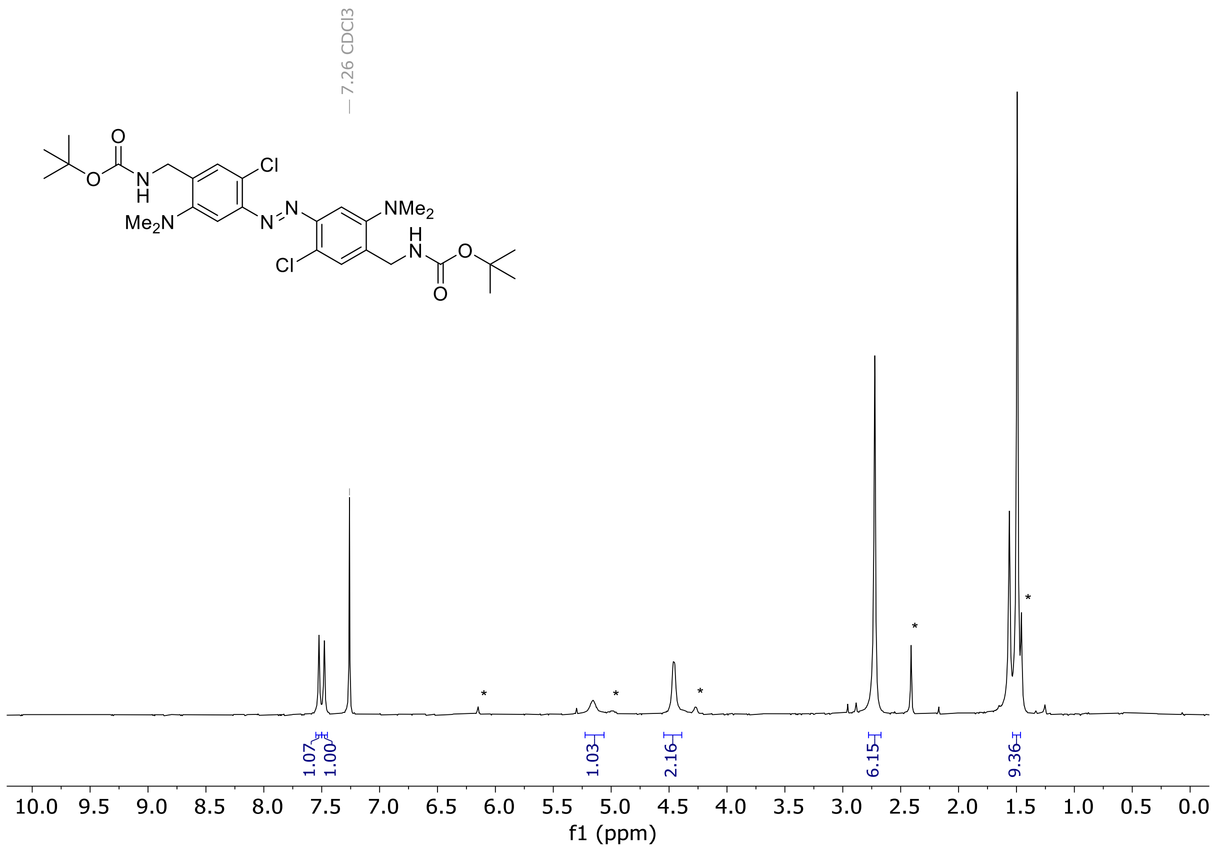

Figure S138. ^{1}H NMR spectrum of **Cl_2,*m*-(NMe_2)$_2$** (Chloroform-*d*, 298 K). (*Z*) signals labelled as *.

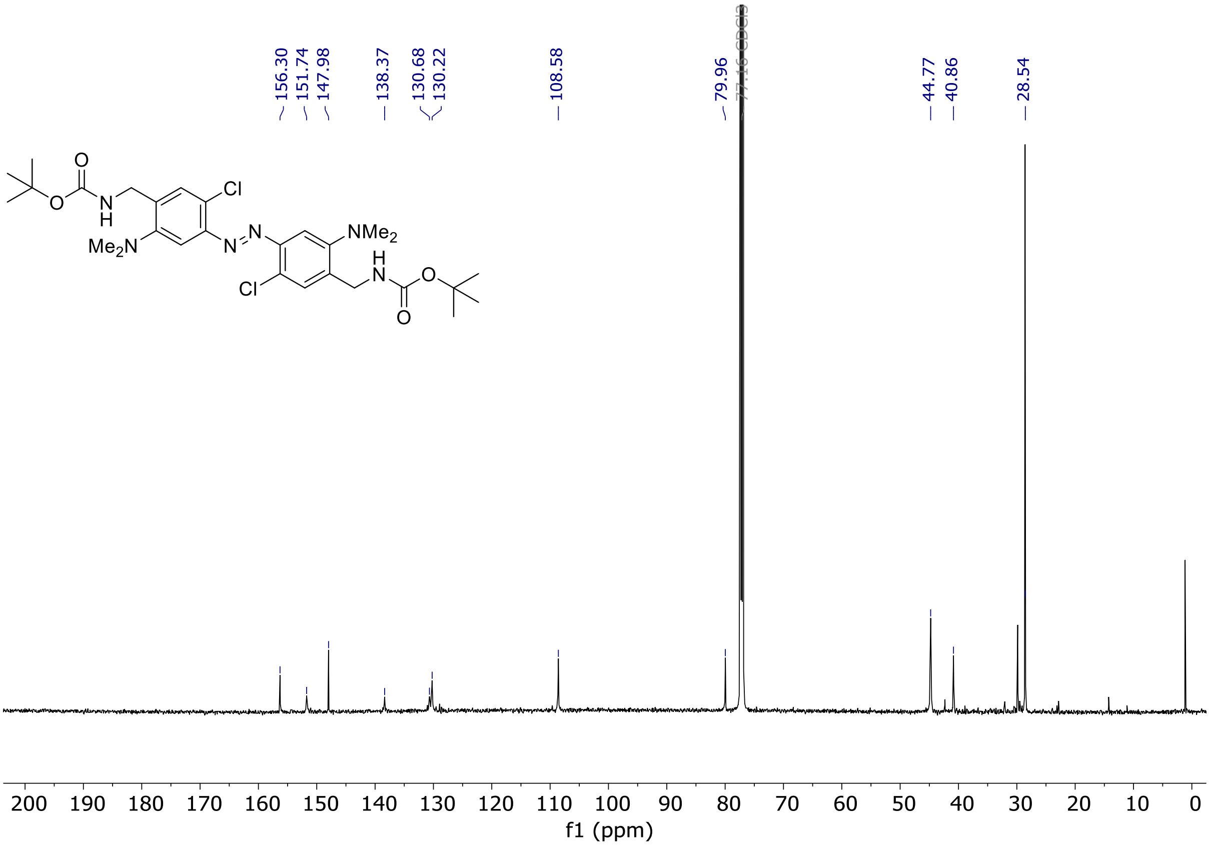

Figure S139. ^{13}C NMR spectrum of **Cl_2,*m*-(NMe_2)$_2$**. (Chloroform-*d*, 298 K).

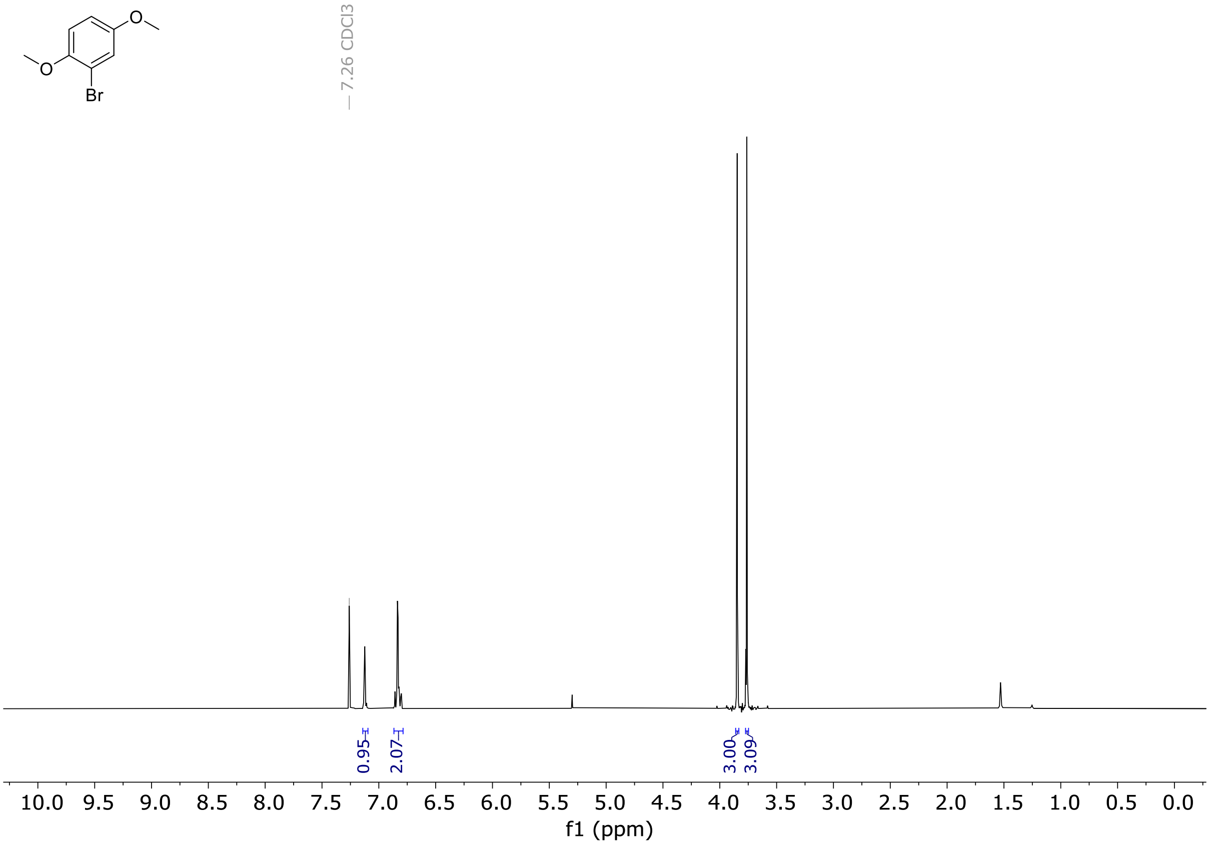

Figure S140. ^{1}H NMR spectrum of **S72** (Chloroform-*d*, 298 K).

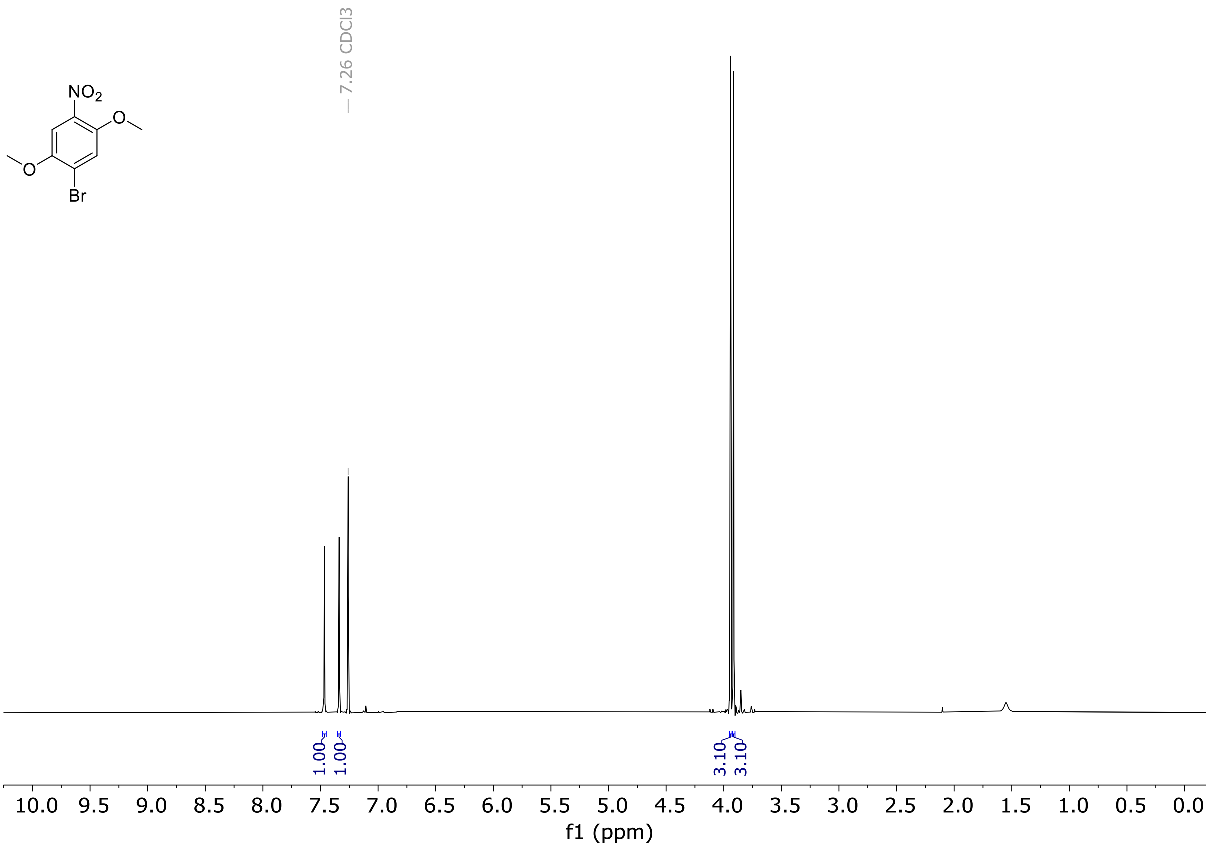

Figure S141. ^{1}H NMR spectrum of **S73** (Chloroform-*d*, 298 K).

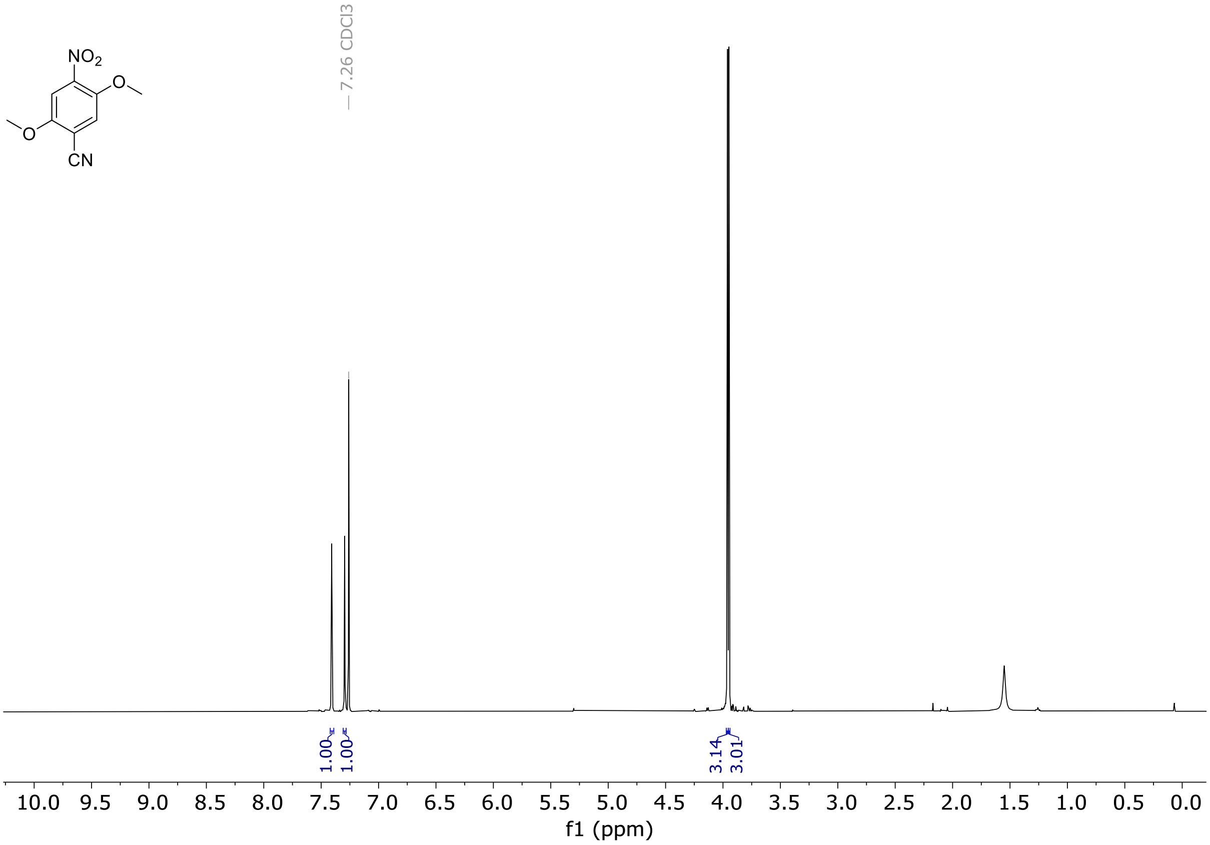

Figure S142. ^{1}H NMR spectrum of **S73** (Chloroform-*d*, 298 K).

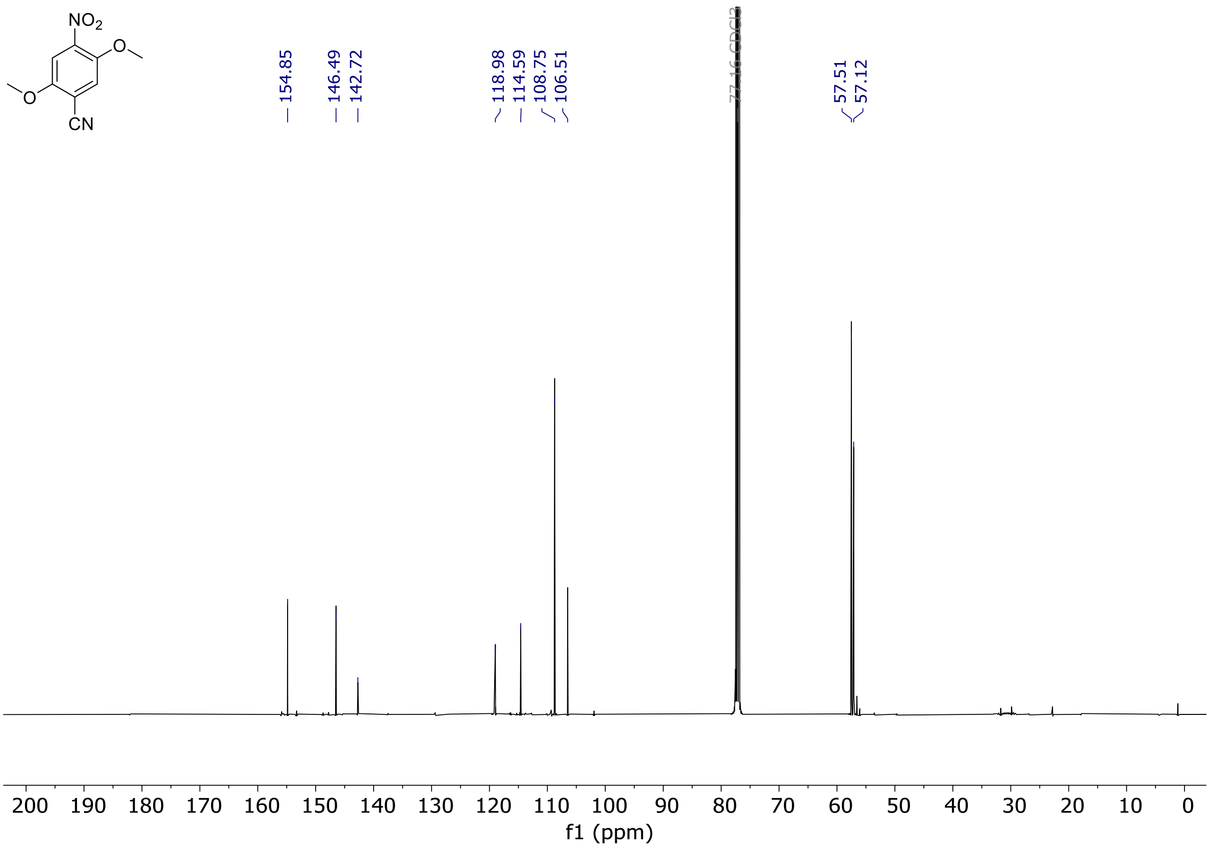

Figure S143. ^{13}C NMR spectrum of **S74**. (Chloroform-*d*, 298 K).

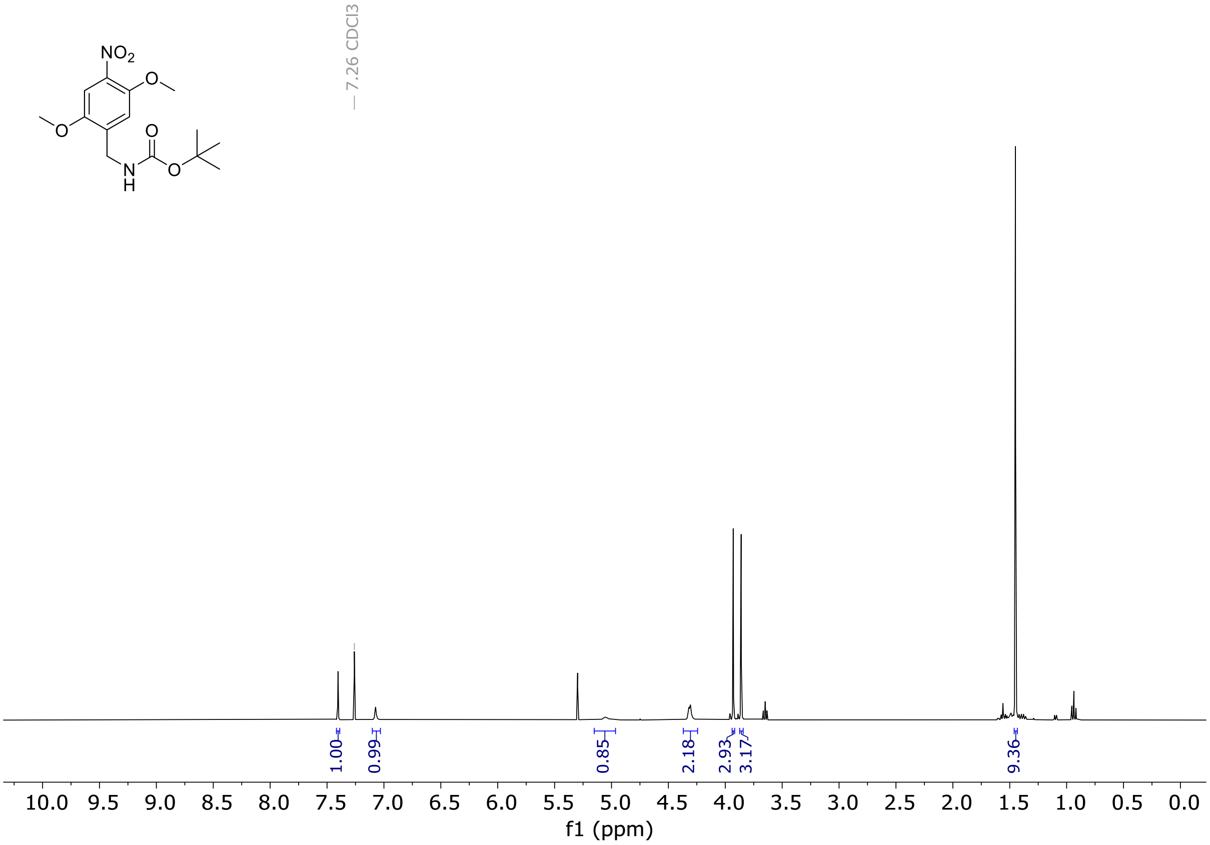

Figure S144. ^{1}H NMR spectrum of **S75** (Chloroform-*d*, 298 K).

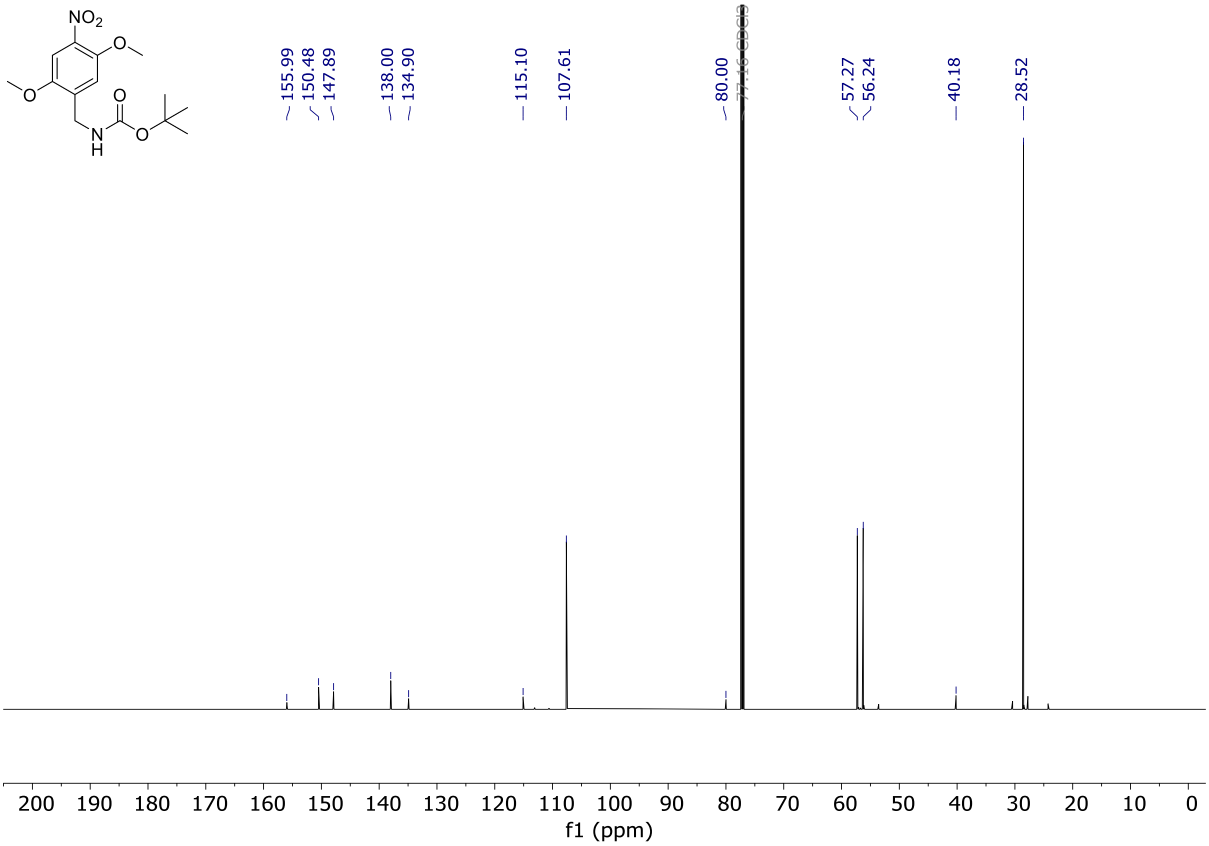

Figure S145. ^{13}C NMR spectrum of **S75**. (Chloroform-*d*, 298 K).

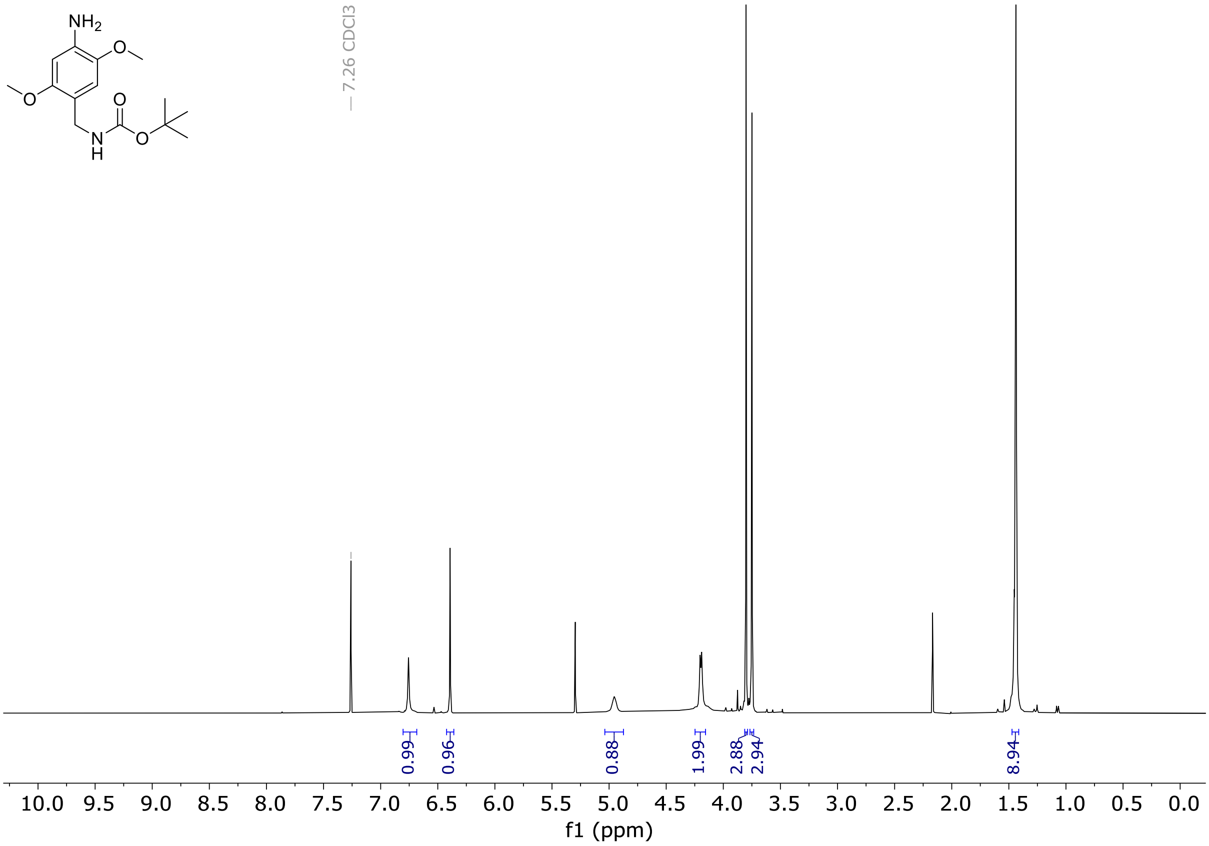

Figure S146. ^{1}H NMR spectrum of **S76** (Chloroform-*d*, 298 K).

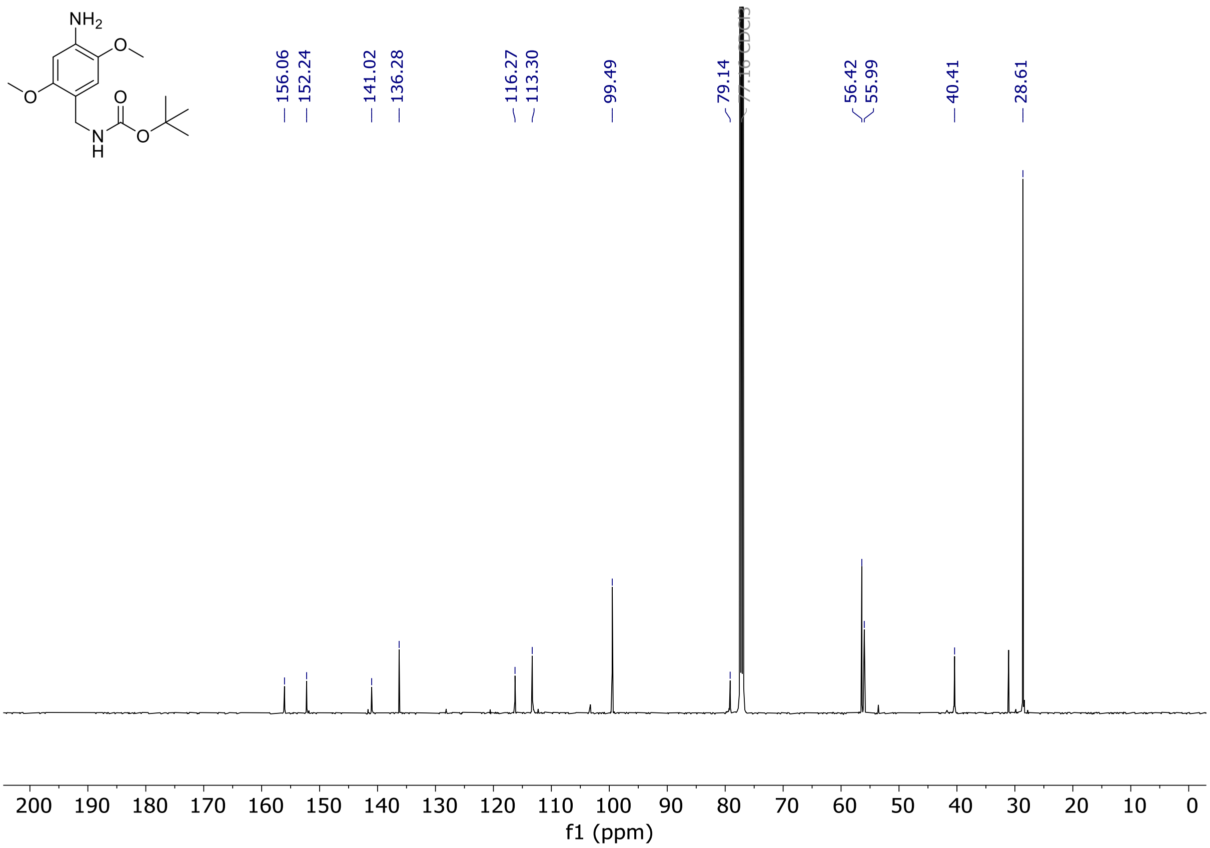

Figure S147. ^{13}C NMR spectrum of **S76**. (Chloroform-*d*, 298 K).

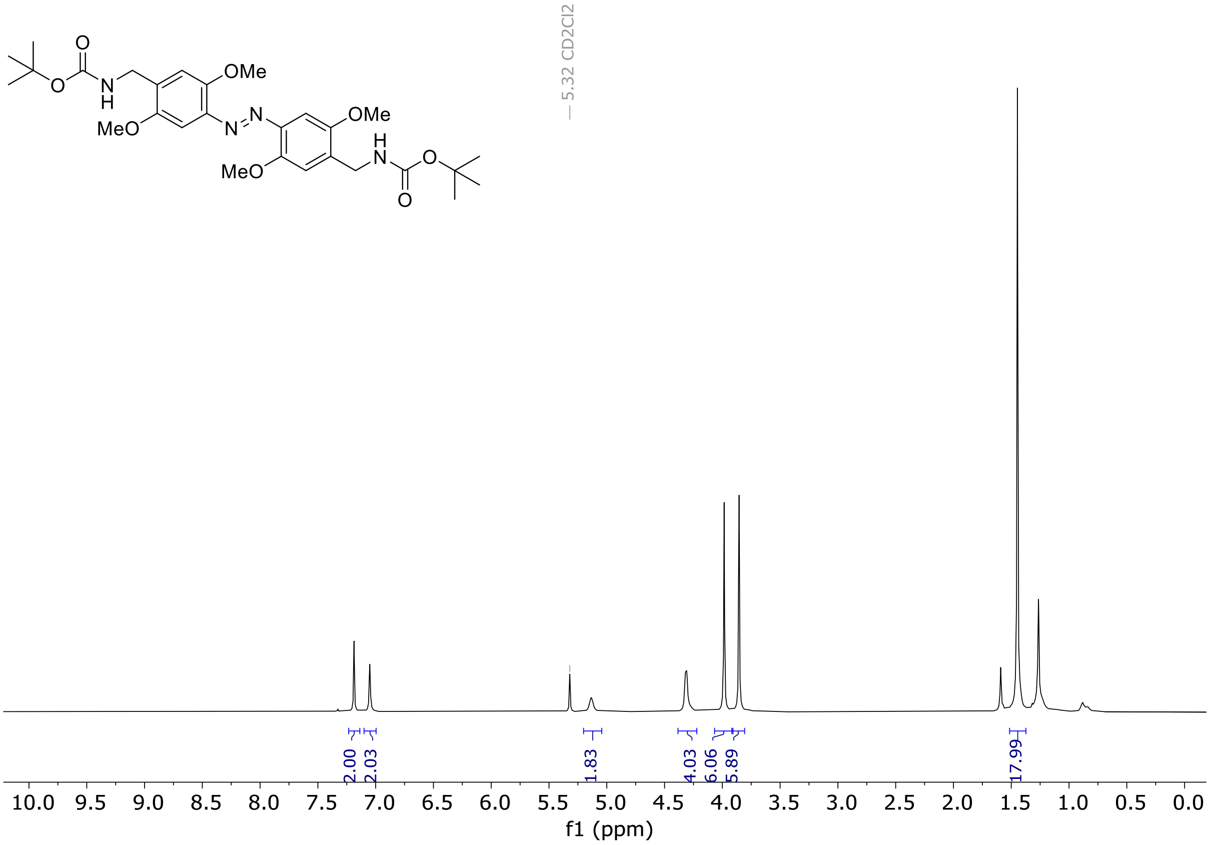

Figure S148. ^{1}H NMR Spectrum of *(E)*-**OMe$_2$,*m*-OMe$_2$** (CD_2Cl_2, 298 K).

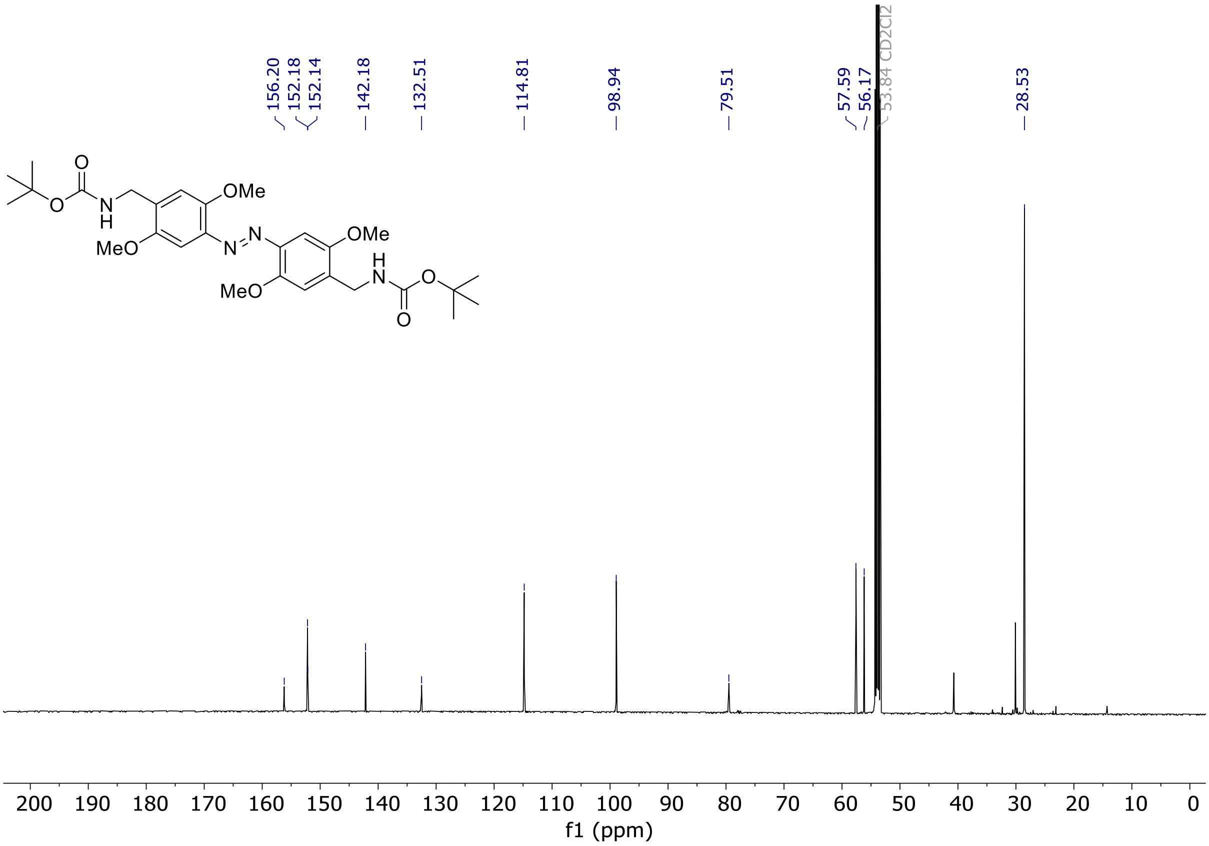

Figure S149. ^{13}C NMR spectrum of *(E)*-**OMe$_2$,*m*-OMe$_2$**. (CD_2Cl_2, 298 K).

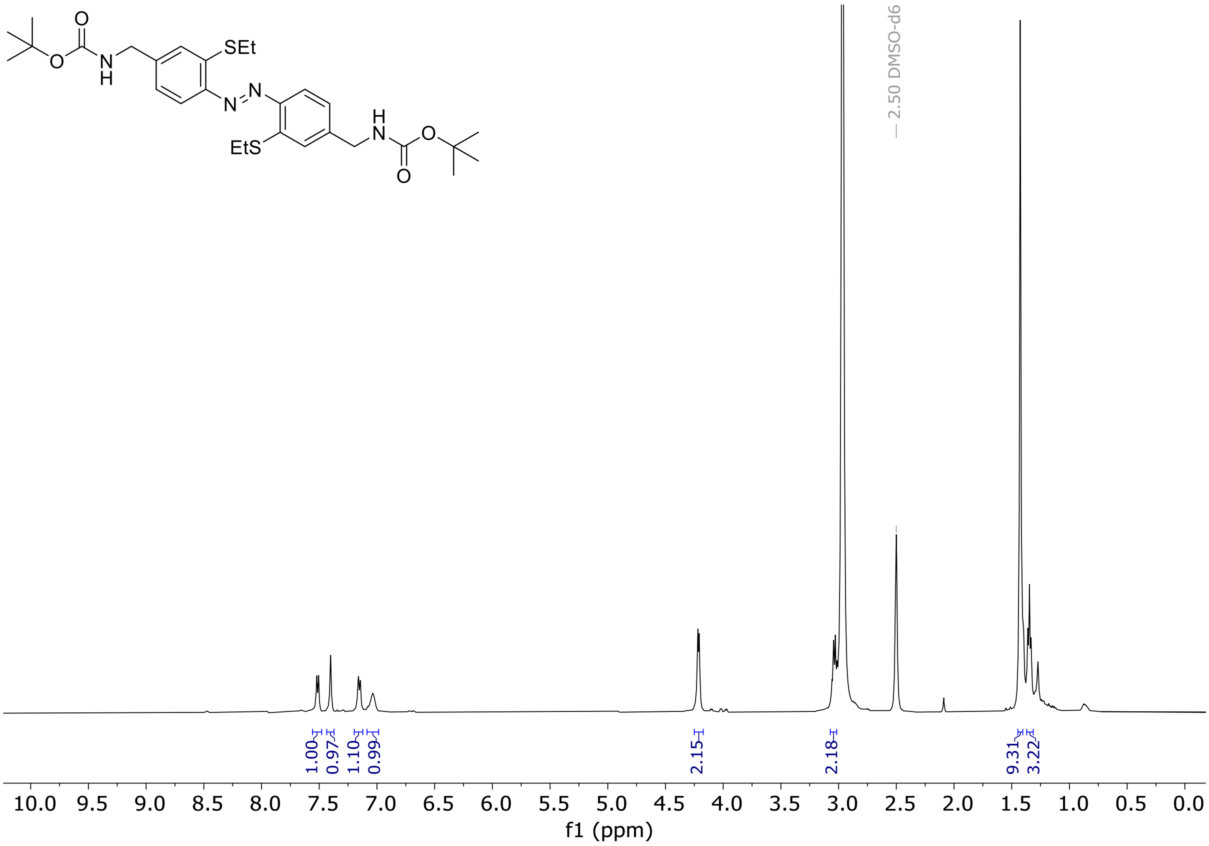

Figure S150. ^{1}H NMR spectrum of **(SEt)$_2$,*m*-H$_2$** (DMSO-d_6, 298 K).

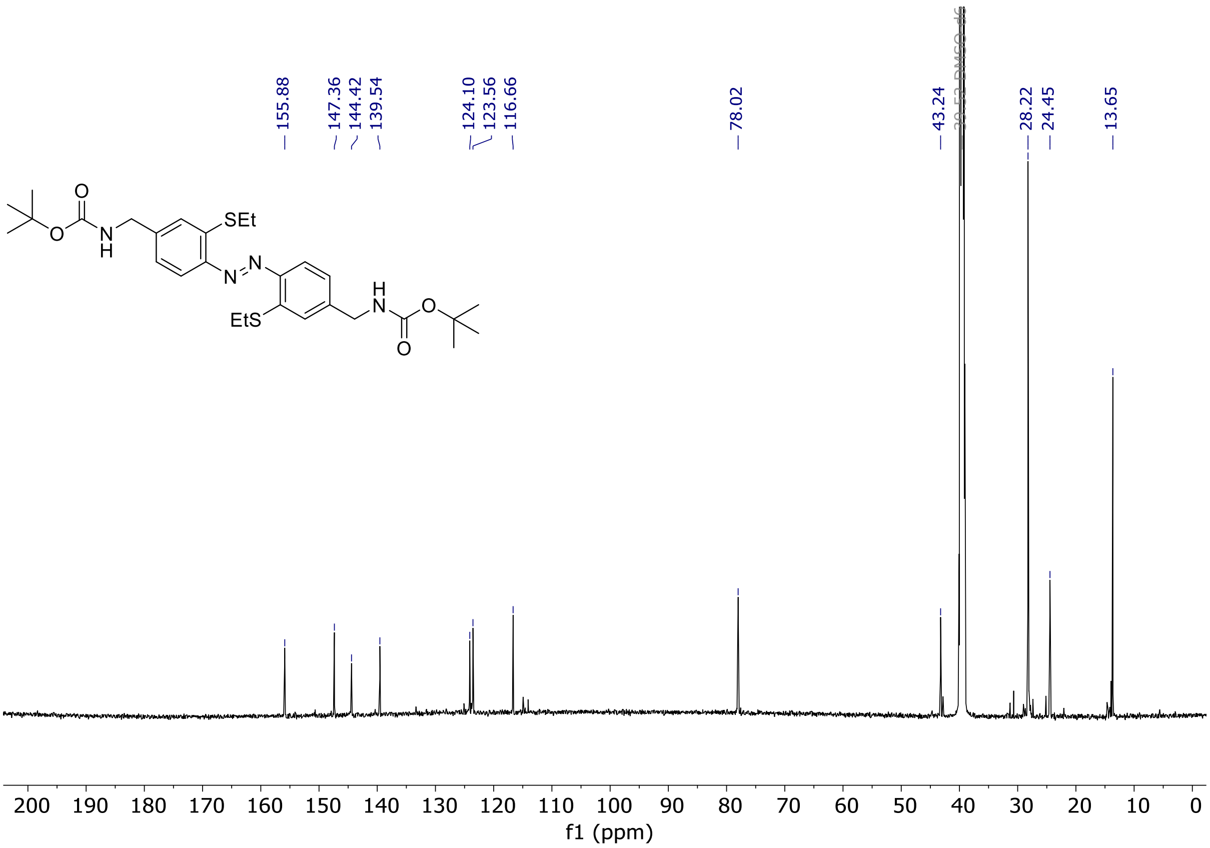

Figure S151. ^{13}C NMR spectrum of **(SEt)$_2$,*m*-H$_2$** (DMSO-d_6, 298 K).

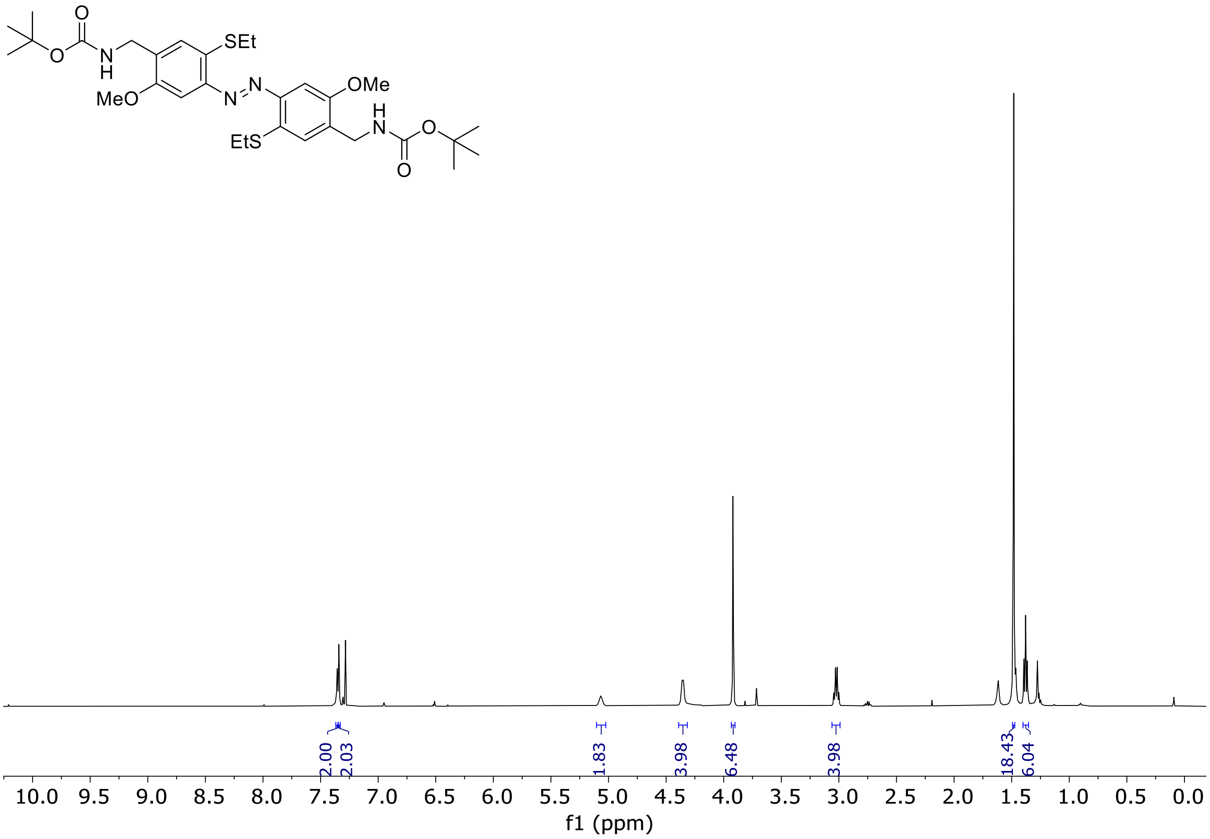

Figure S152. ^{1}H NMR spectrum of **(SEt)$_2$,*m*-OMe$_2$** (Chloroform-d, 298 K).

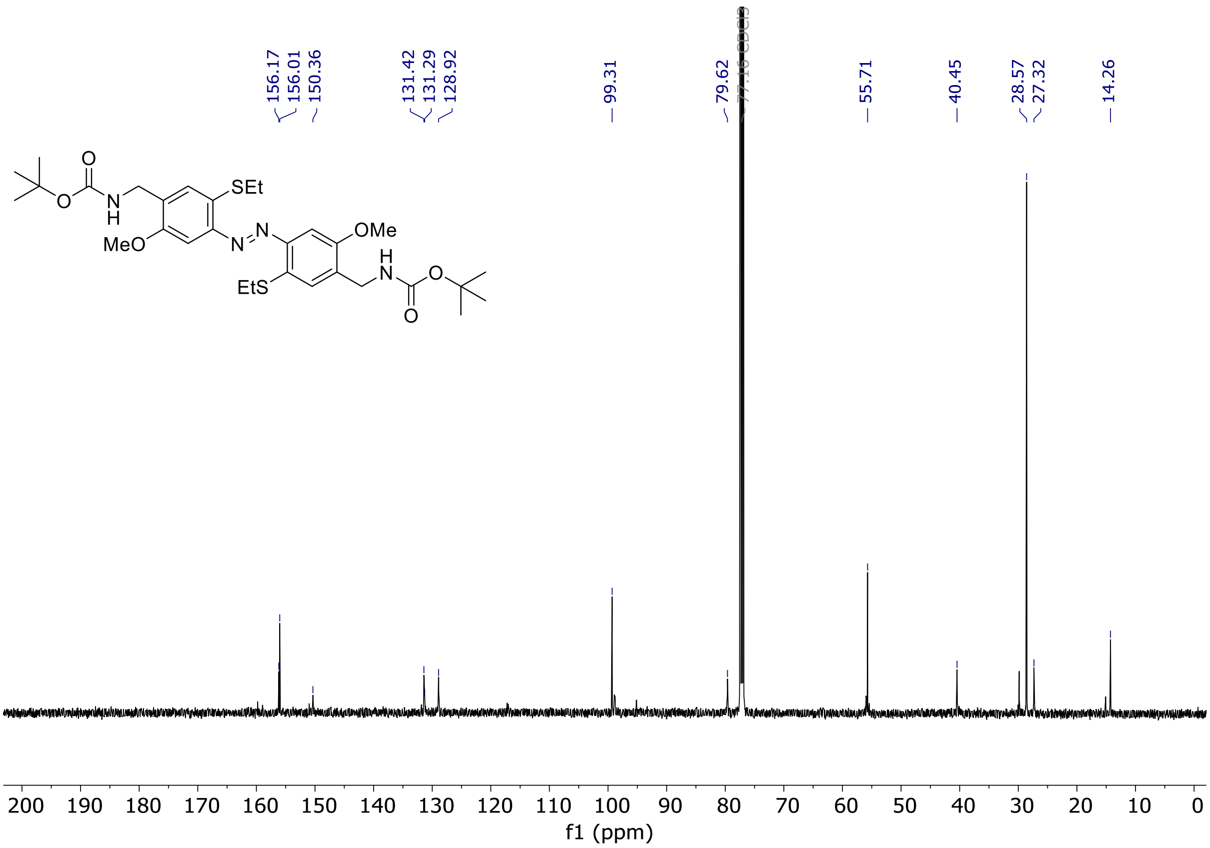

Figure S153. ^{13}C NMR spectrum of **(SEt)$_2$,*m*-OMe$_2$**. (Chloroform-*d*, 298 K).

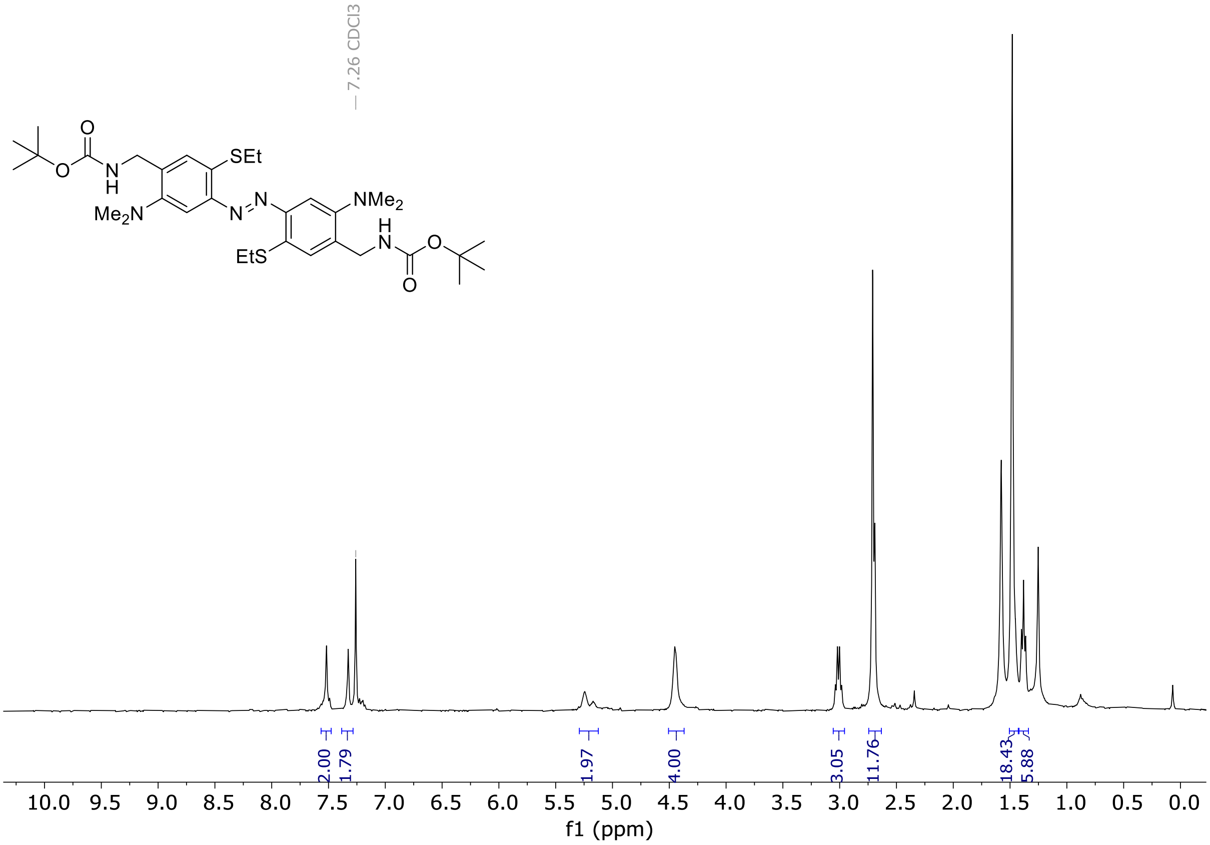

Figure S154. ^{1}H NMR spectrum of **(SEt$_2$),*m*-(NMe$_2$)$_2$** (Chloroform-*d*, 298 K).

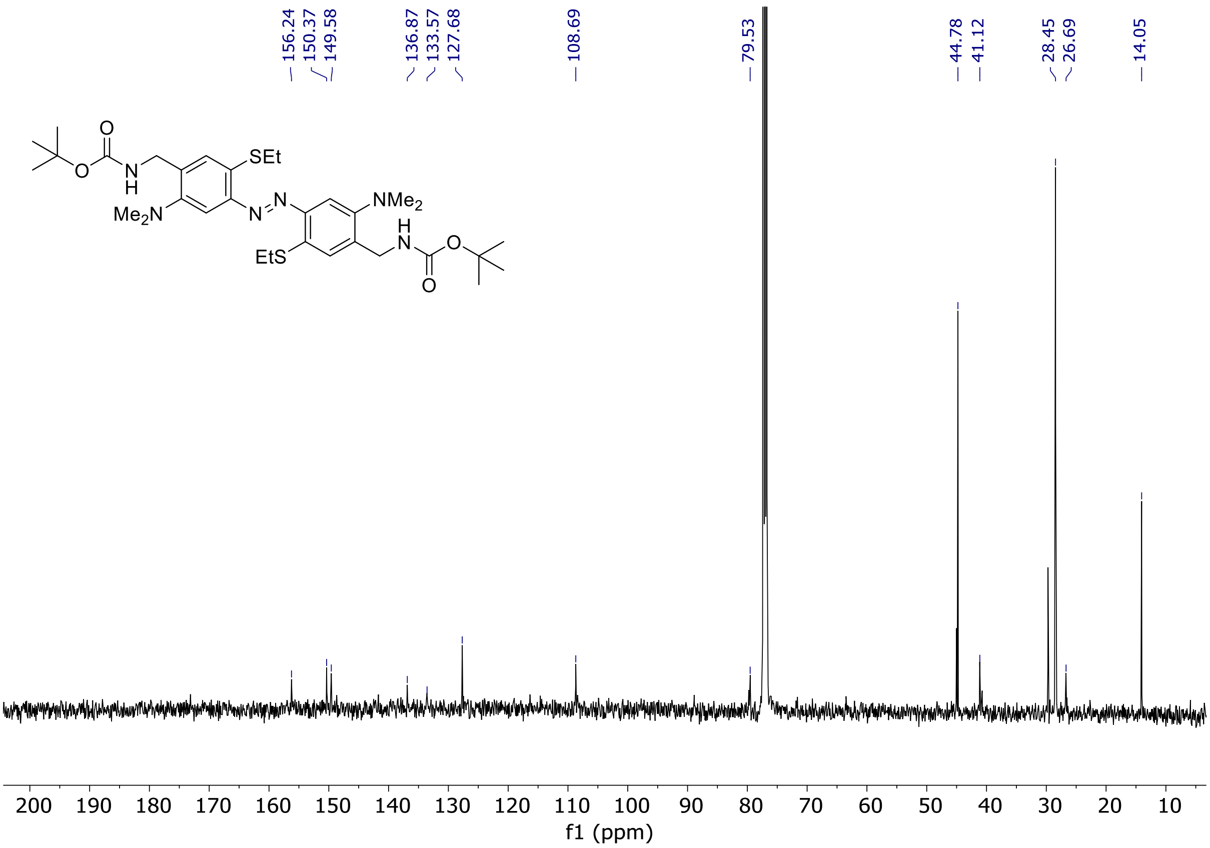

Figure S155. ^{13}C NMR spectrum of **(SEt$_2$),*m*-(NMe$_2$)$_2$**. (Chloroform-*d*, 298 K).

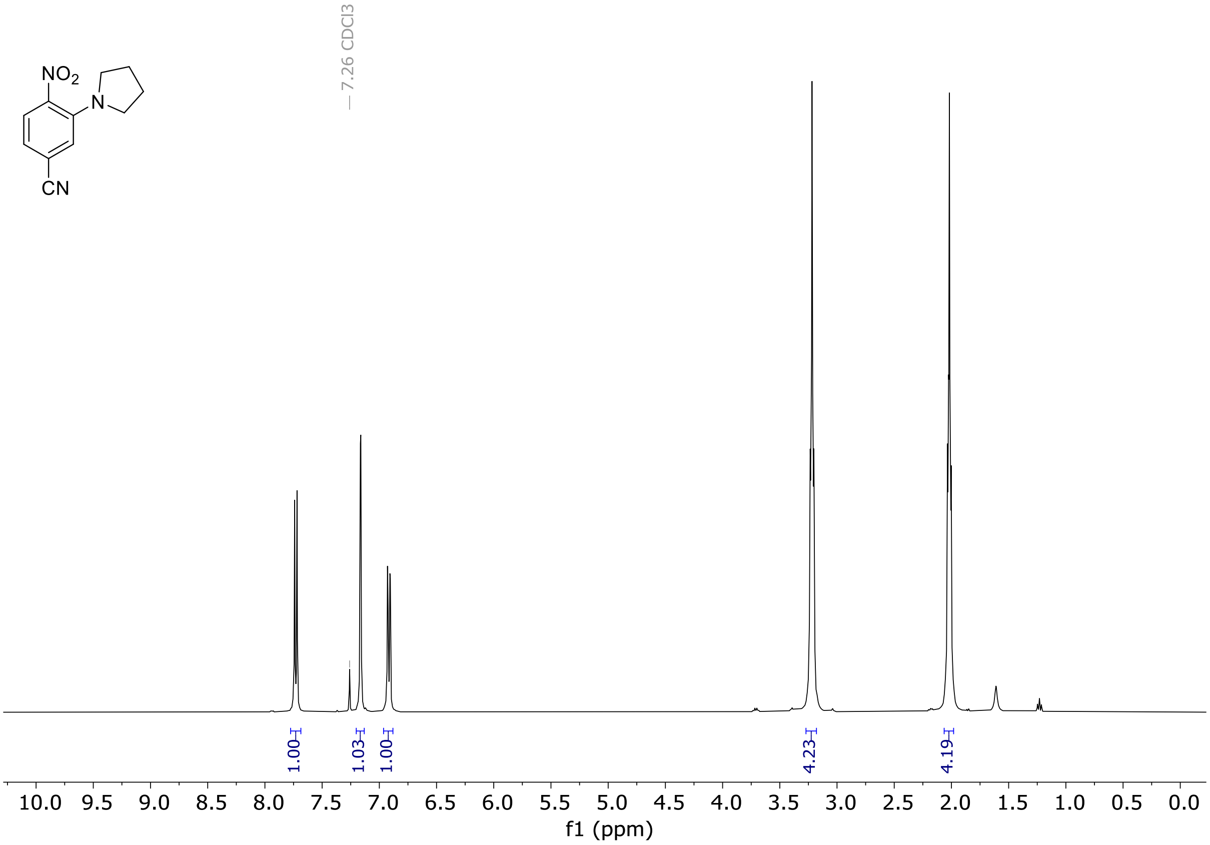

Figure S156. ^{1}H NMR spectrum of **S78** (Chloroform-*d*, 298 K).

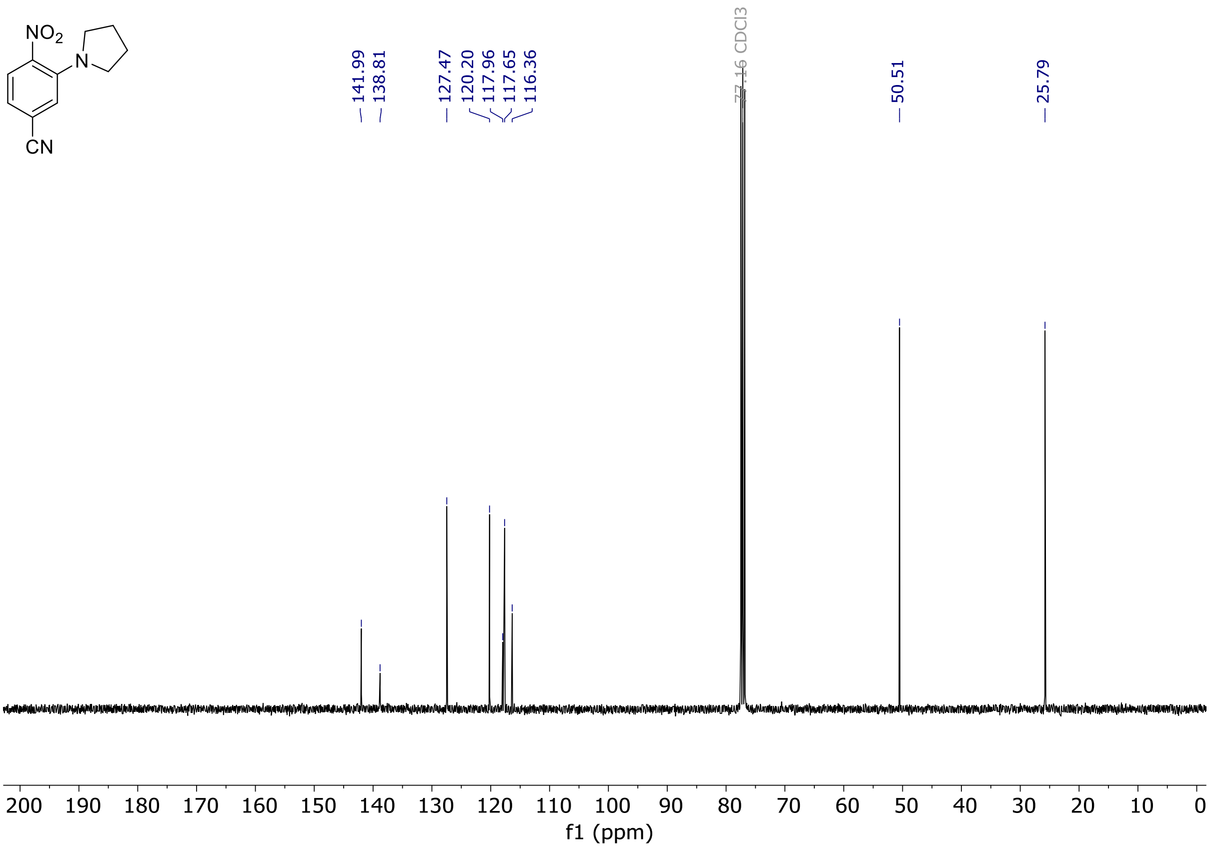

Figure S157. ^{13}C NMR spectrum of **S78** (Chloroform-*d*, 298 K).

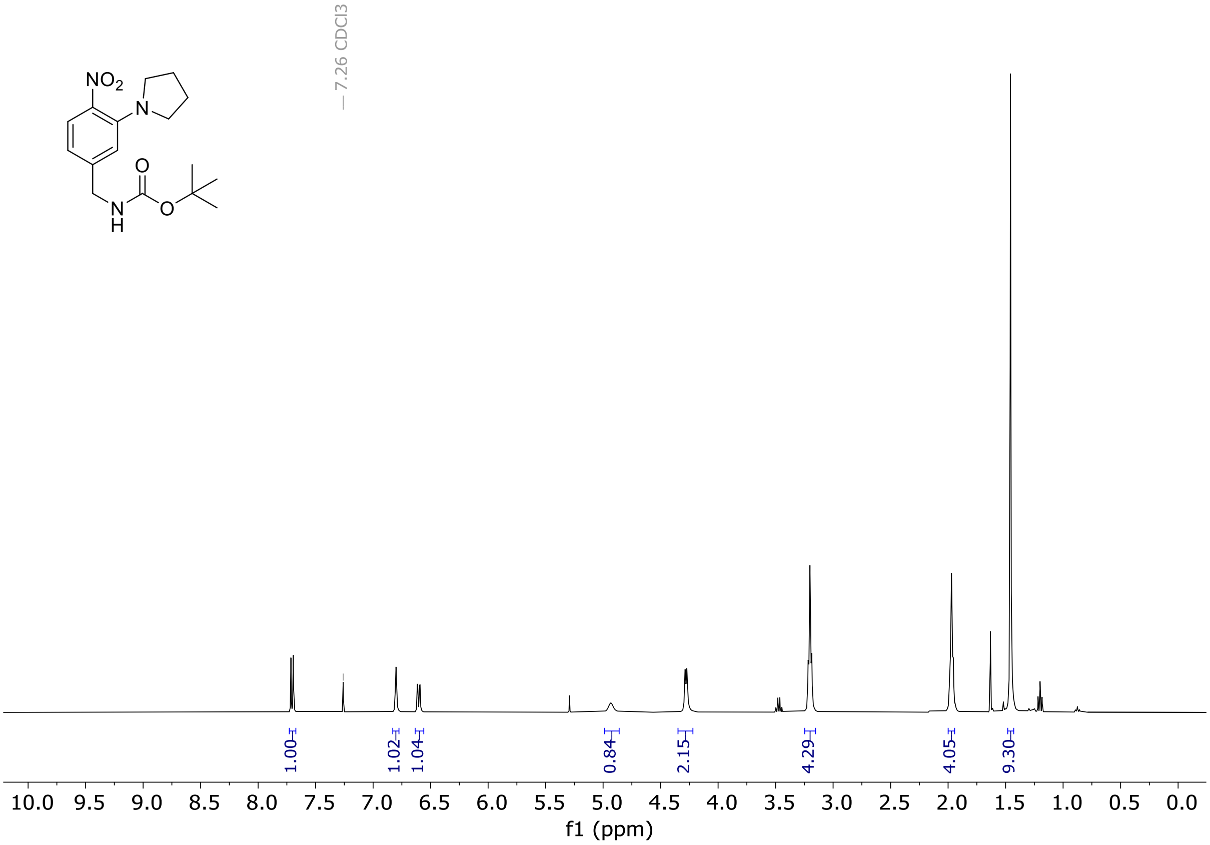

Figure S158. ^{1}H NMR spectrum of **S79** (Chloroform-*d*, 298 K).

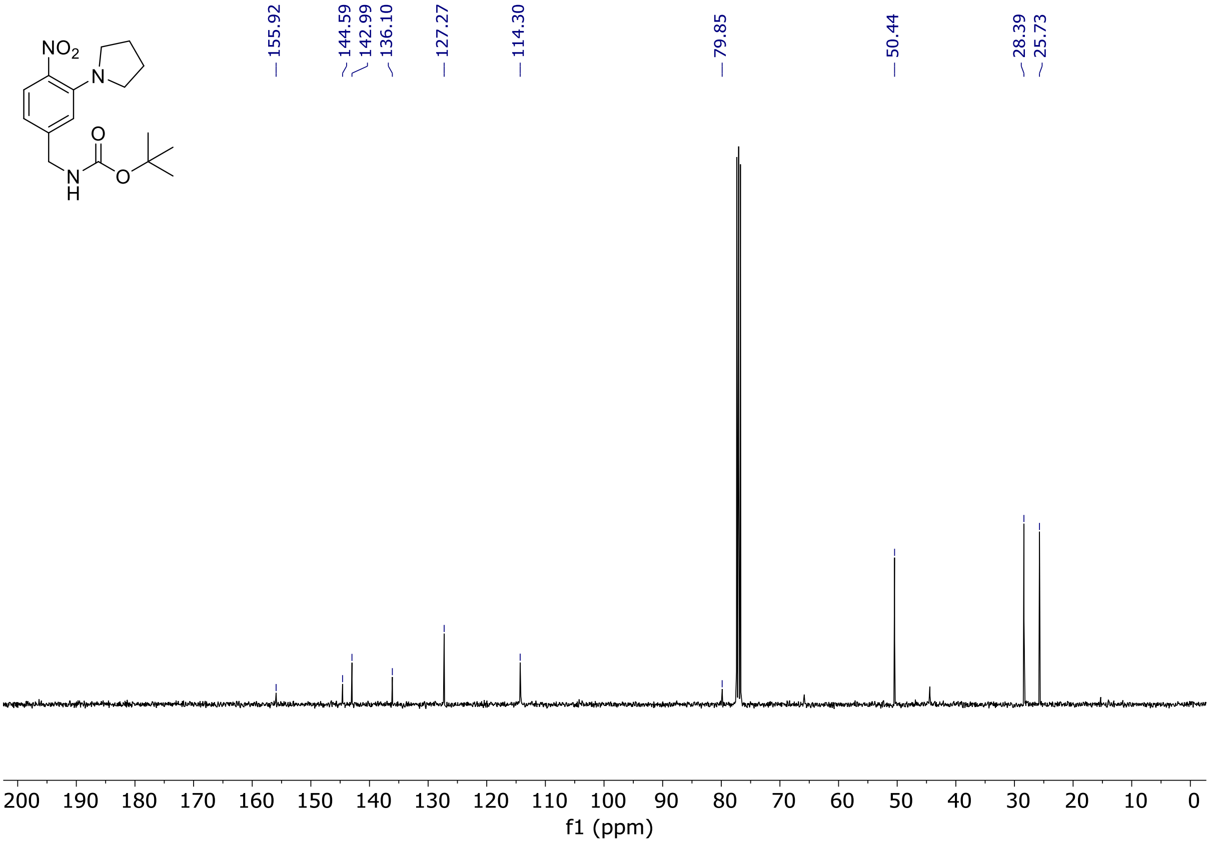

Figure S159. ^{13}C NMR spectrum of **S79** (Chloroform-*d*, 298 K).

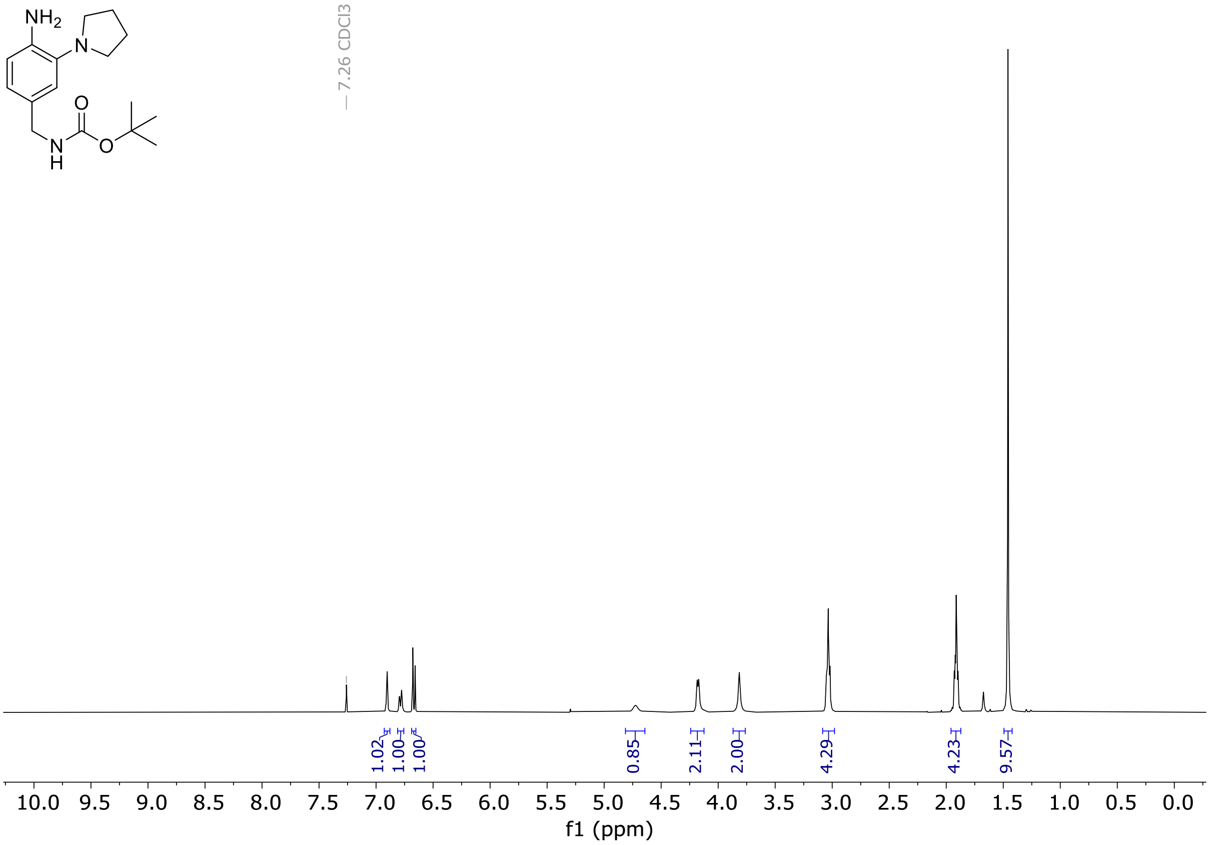

Figure S160. ^{1}H NMR spectrum of **S80** (Chloroform-*d*, 298 K).

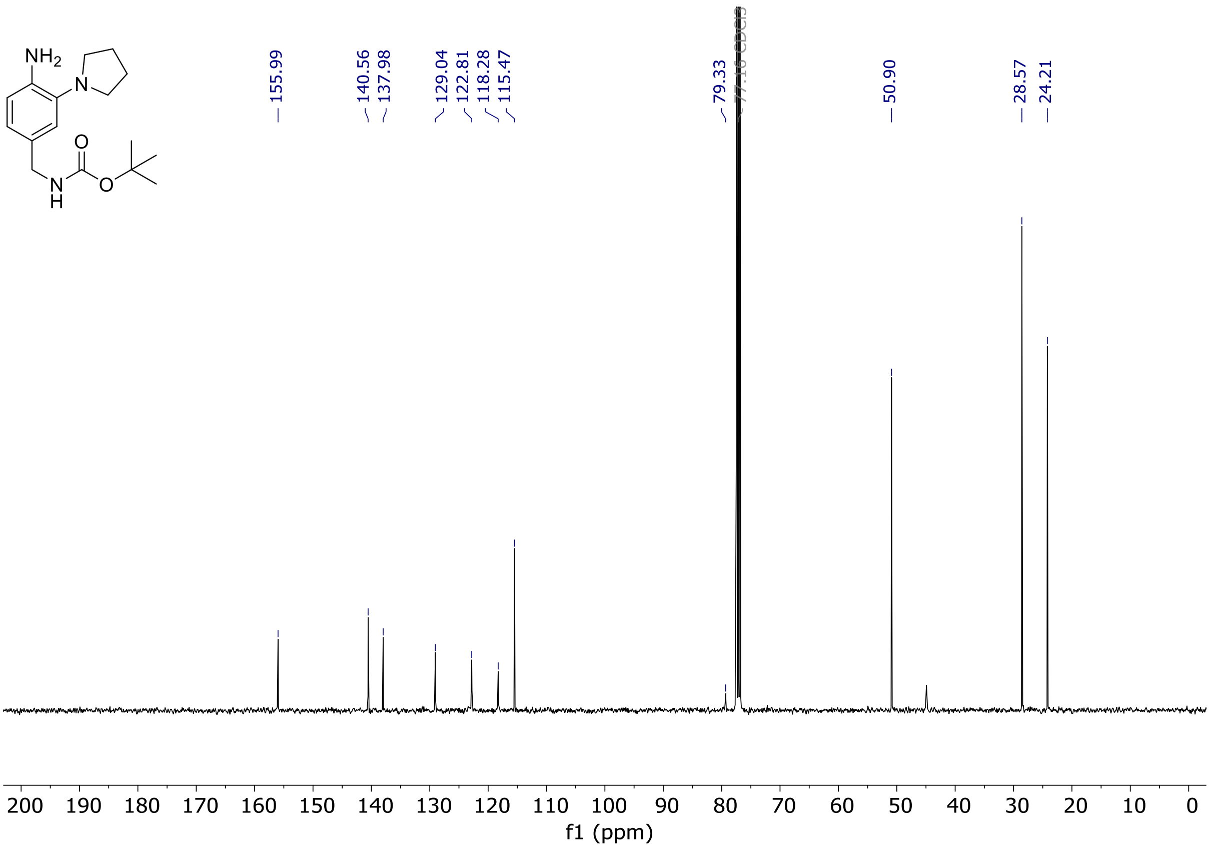

Figure S161. ^{13}C NMR spectrum of **S80** (Chloroform-*d*, 298 K).

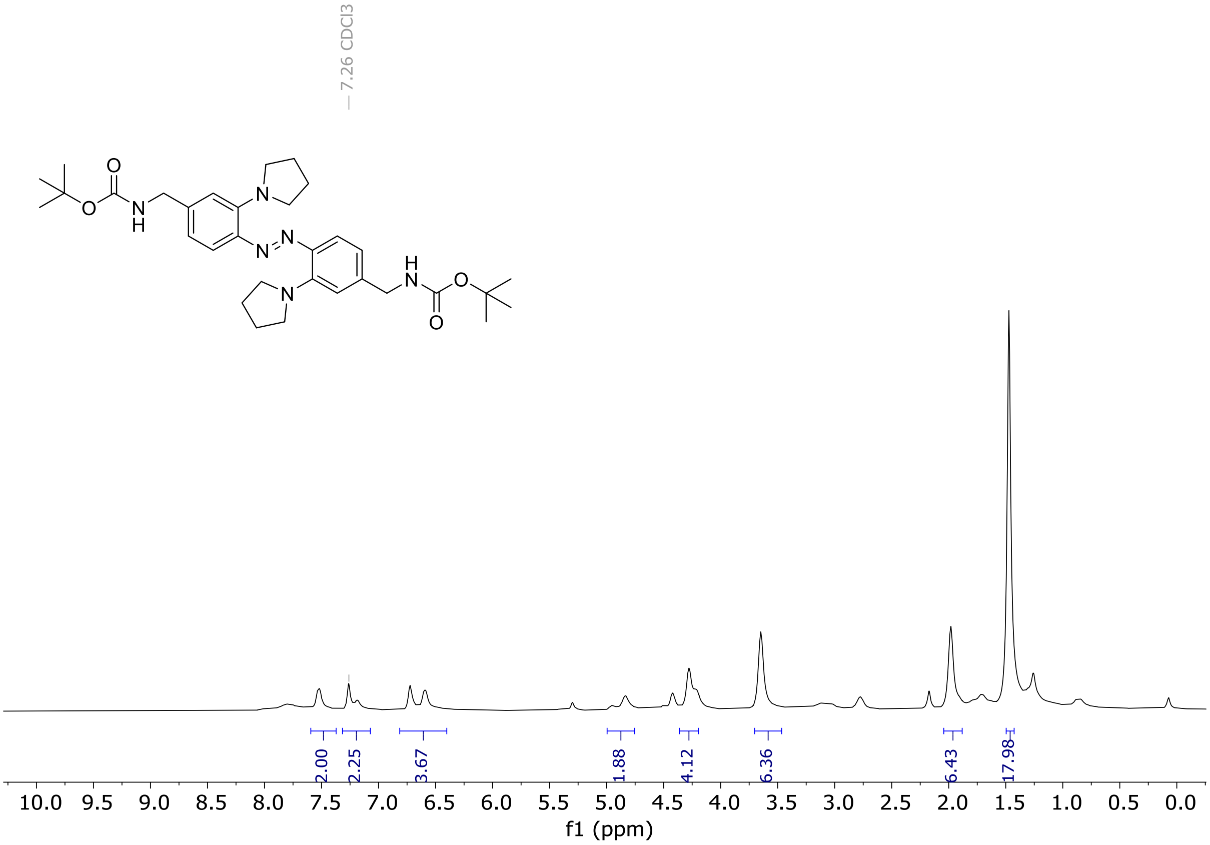

Figure S162. ^{1}H NMR spectrum of **$(N(CH_2)_4)_2$,*m*-H** (Chloroform-*d*, 298 K).

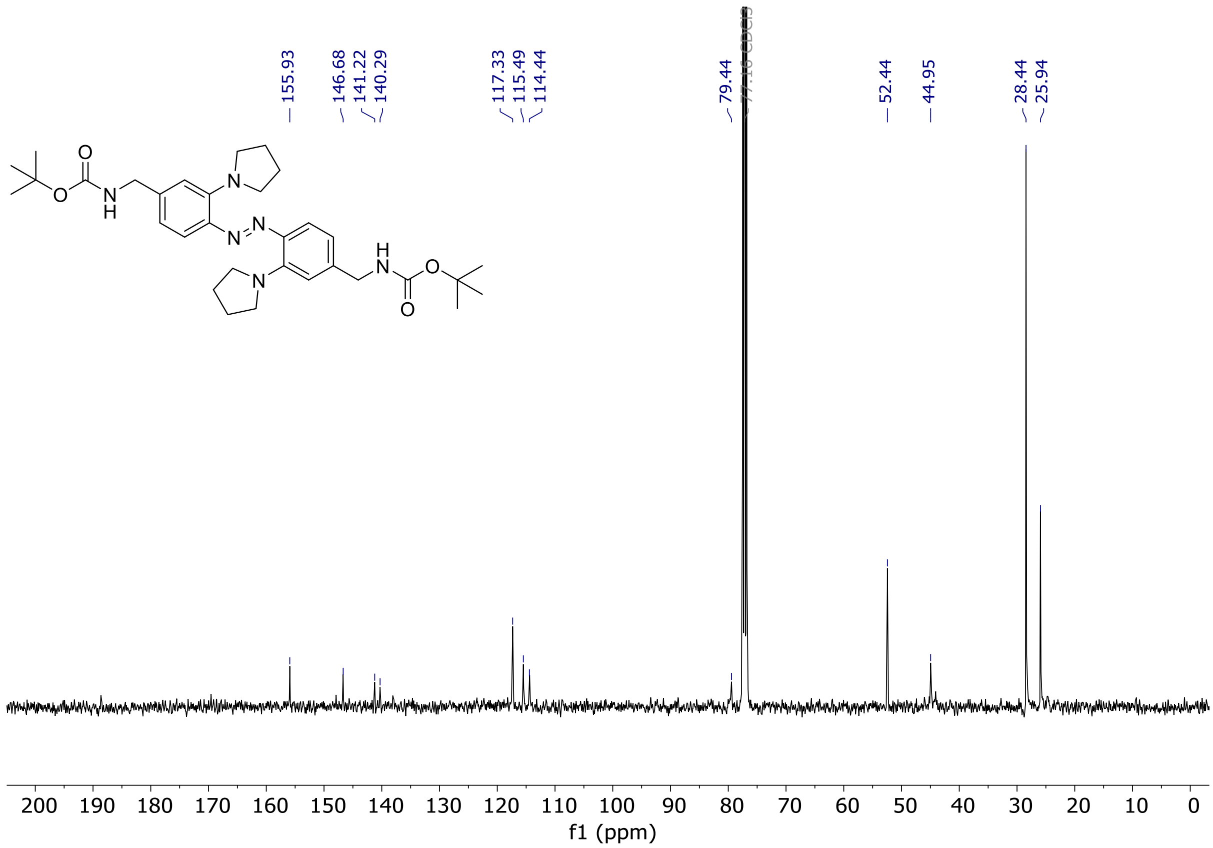

Figure S163. ^{13}C NMR spectrum of **(N(CH$_2$)$_4$)$_2$,*m*-H** (Chloroform-*d*, 298 K).

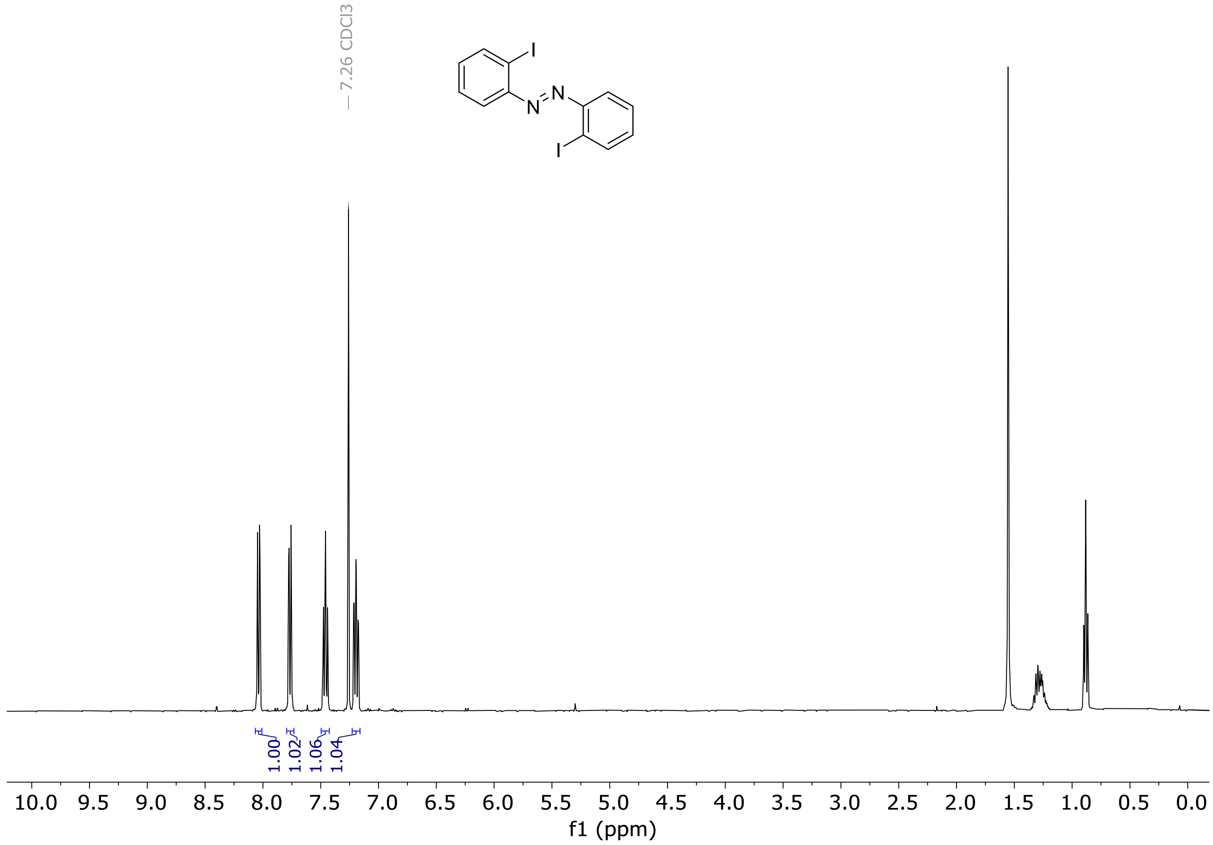

Figure S164. ^{1}H NMR spectrum of **S82** (Chloroform-*d*, 298 K).

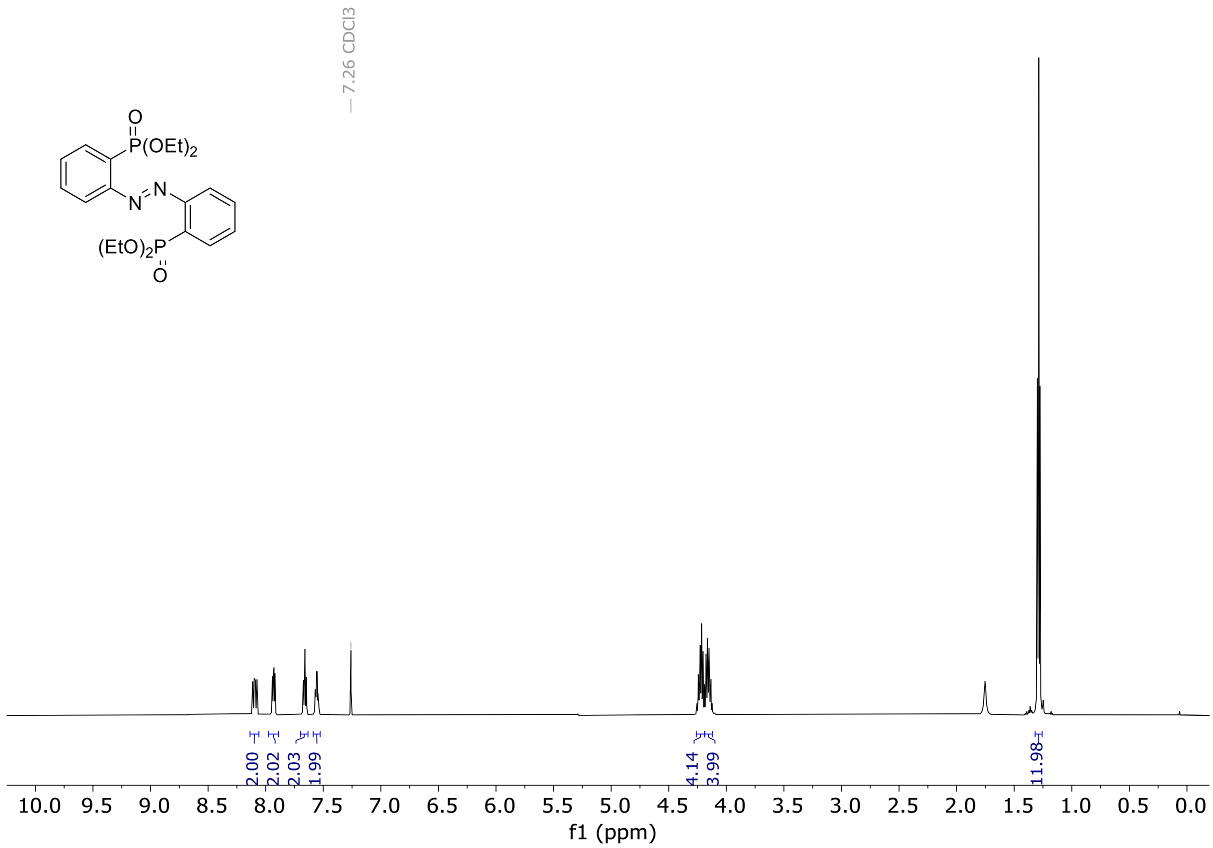

Figure S165. ^{1}H NMR spectrum of **(PO(OEt)$_2$)$_2$,*m*-H** (Chloroform-*d*, 298 K).

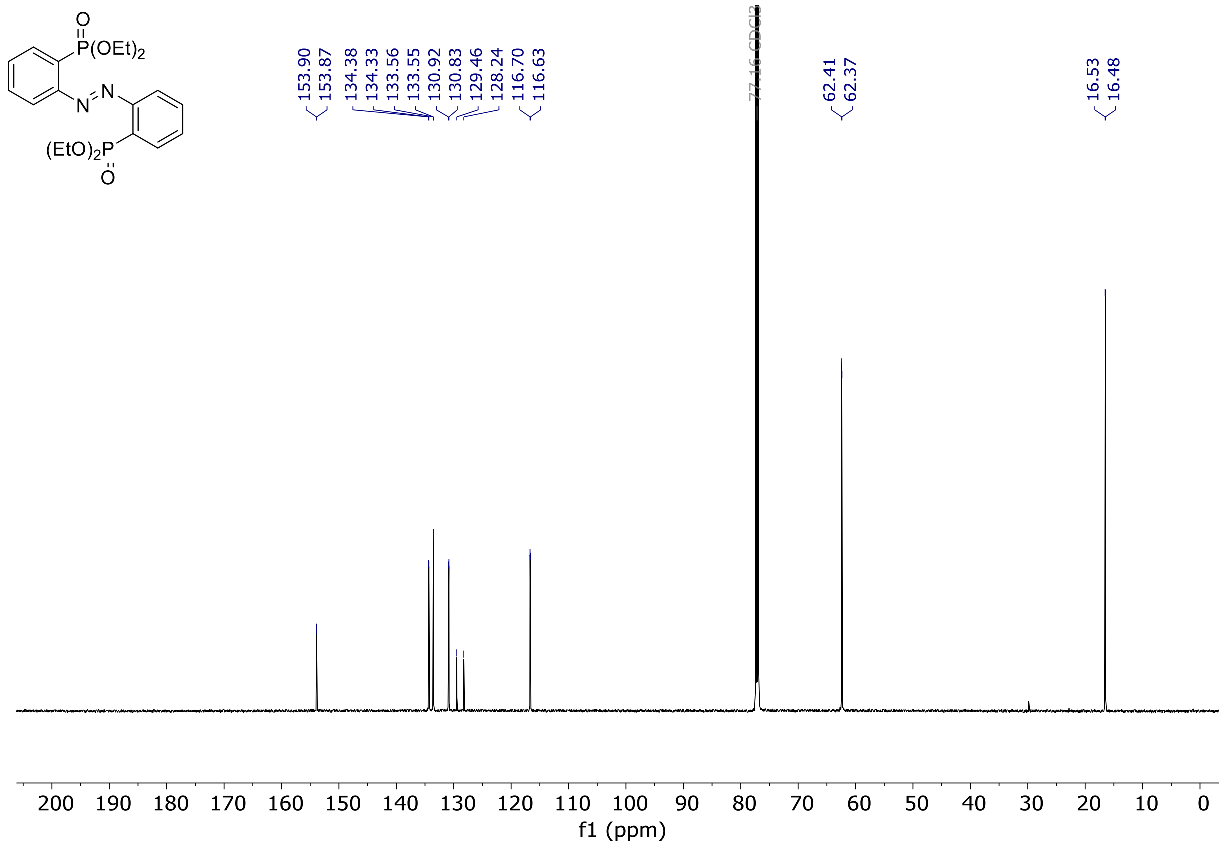

Figure S166. ^{13}C NMR spectrum of **(PO(OEt)$_2$)$_2$,*m*-H** (Chloroform-*d*, 298 K).

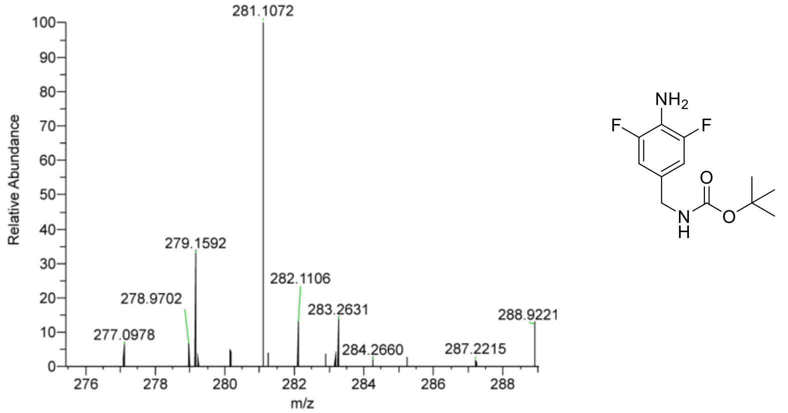

Figure S167. HRMS spectrum of **S5.** HRMS-ESI (m/z) Calculated for $C_{12}H_{16}O_2N_2F_2$ $[M+Na]^+$, 281.1072; found 281.1072.

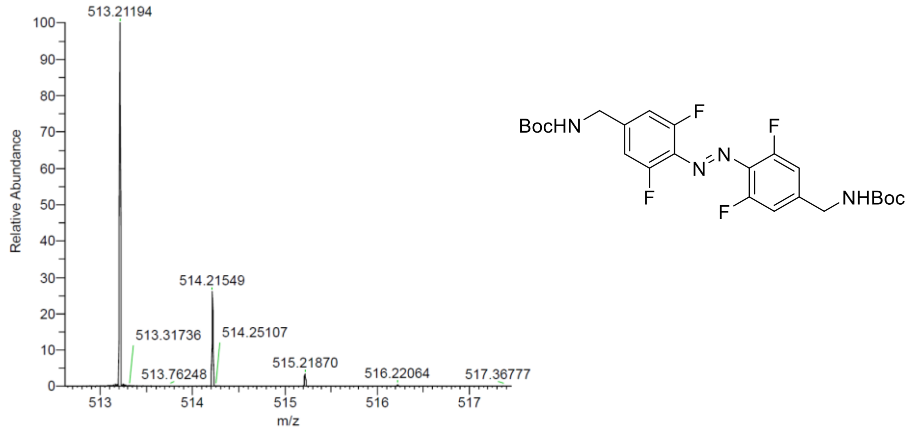

Figure S168. HRMS spectrum of **F_4-NHBoc.** HRMS-ESI (m/z) Calculated for $C_{24}H_{28}F_4N_4O_4$ $[M+H]^+$, 513.2119; found 513.2119.

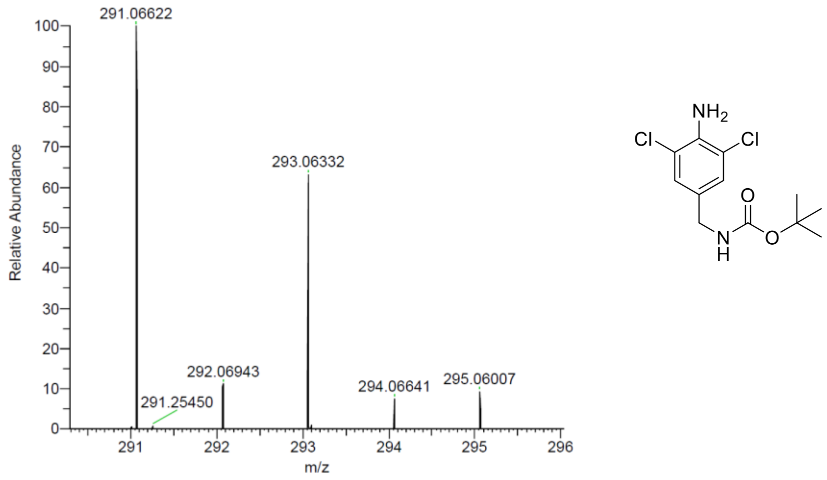

Figure S169. HRMS spectrum of **S10.** HRMS-ESI (m/z) Calculated for $C_{12}H_{16}Cl_2N_2O_2$ $[M+H]^+$, 291.0661; found 291.0662.

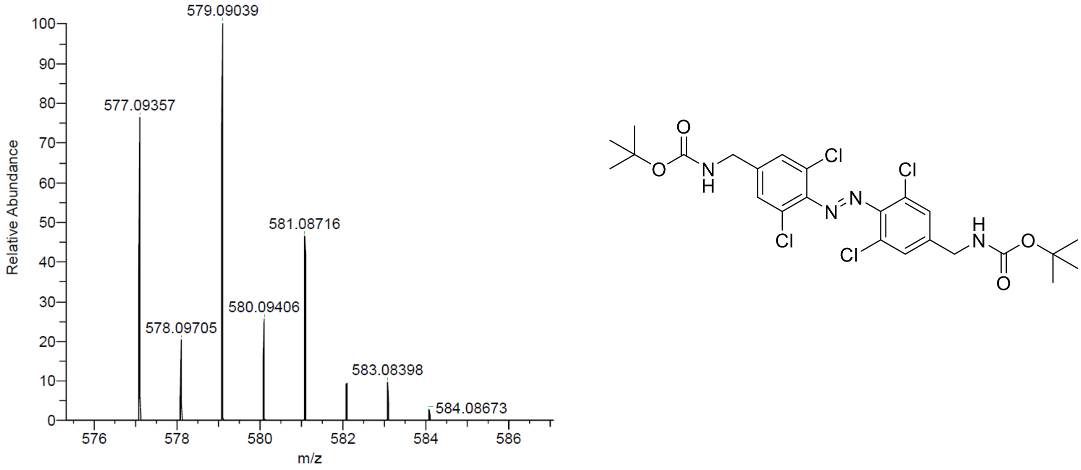

Figure S170. HRMS spectrum of **Cl_4-NHBoc.** HRMS-ESI (m/z) Calculated for $C_{24}H_{28}Cl_4N_4O_4$ $[M+H]^+$, 577.0937; found 577.0936.

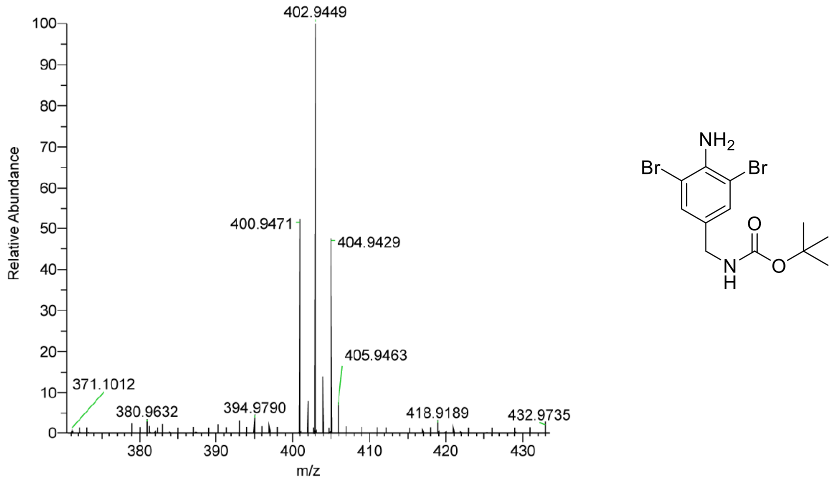

Figure S171. HRMS spectrum of **S14.** HRMS-EI (m/z) Calculated for $C_{12}H_{16}O_2N_2Br_2Na$ $[M+Na]^+$, 402.9450; found 402.9449.

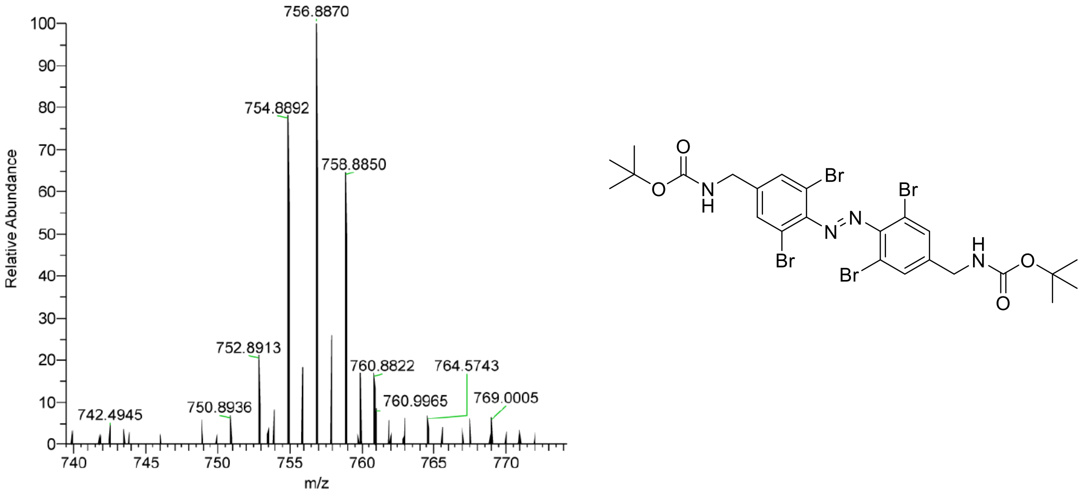

Figure S172. HRMS spectrum of **Br_4-NHBoc.** HRMS-EI (m/z) Calculated for $C_{24}H_{29}O_4N_4Br_4$ $[M+H]^+$, 756.8878; found 756.8870.

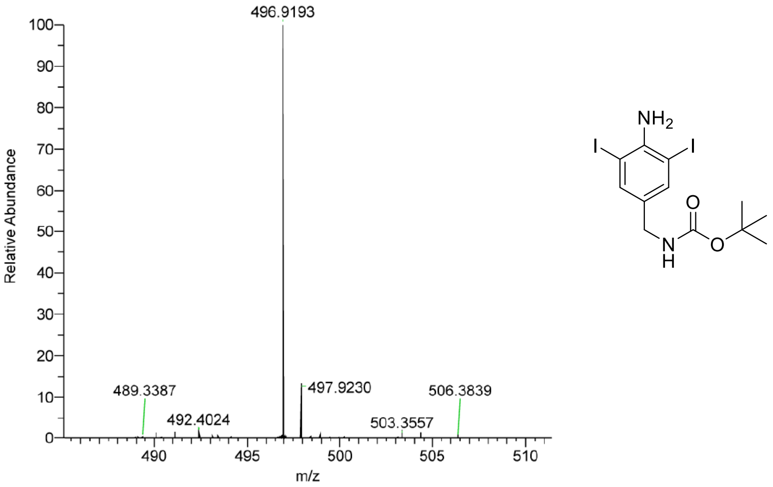

Figure S173. HRMS spectrum of **S17.** HRMS-EI (m/z) Calculated for $C_{12}H_{16}N_2O_6I_2$ $[M+H]^+$, 496.9193; found 496.9193.

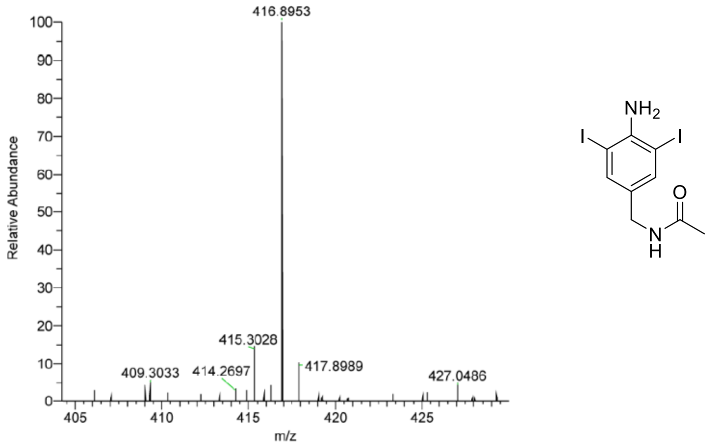

Figure S174. HRMS spectrum of **S18.** HRMS-EI (m/z) Calculated for $C_9H_{11}N_2OI_2$ $[M+H]^+$, 416.8955; found 416.8953.

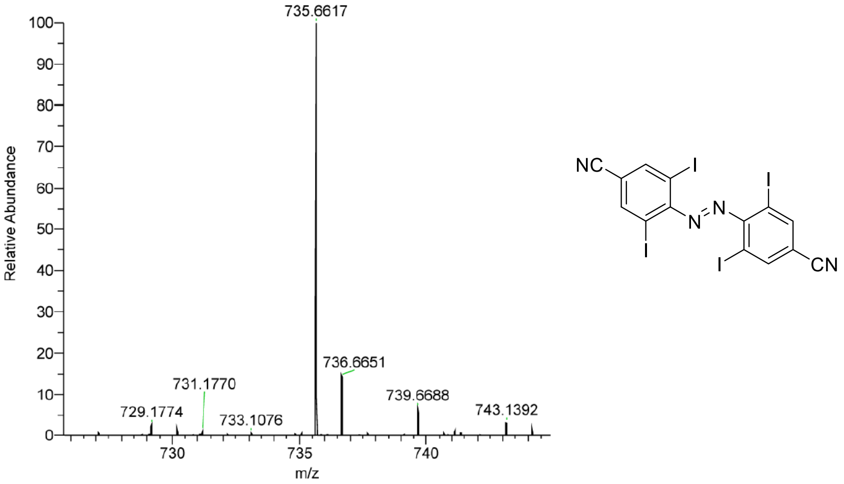

Figure S175. HRMS spectrum of **I_4-CN.** HRMS-EI (m/z) Calculated for $C_{12}H_{16}N_2O_6I_2$ $[M-H]^-$, 735.6620; found 735.6617.

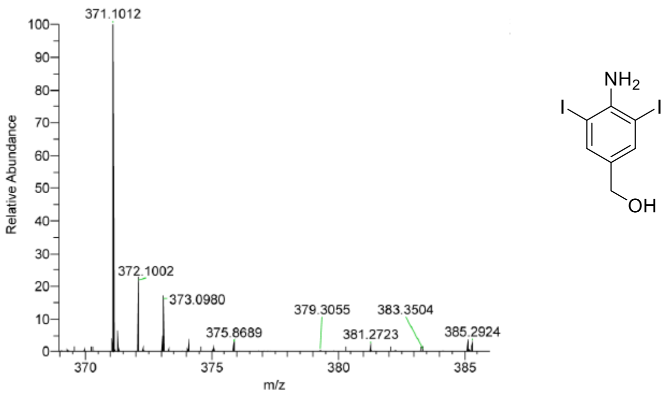

Figure 176. HRMS spectrum of **S22.** HRMS-EI (m/z) Calculated for $C_7H_8NOI_2$ $[M+H]^+$, 375.8690; found 375.8689.

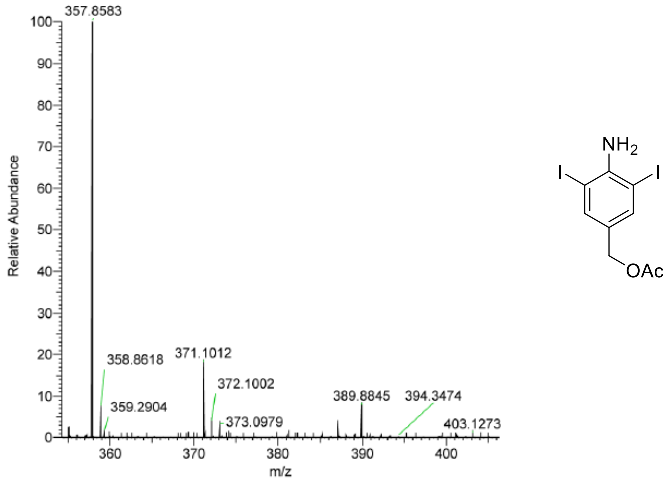

Figure 177. HRMS spectrum of **S23.** HRMS-EI (m/z) Calculated for $C_{17}H_6I_2$ $[M\text{-}OAc^-]^+$, 357.8584; found 357.8583.

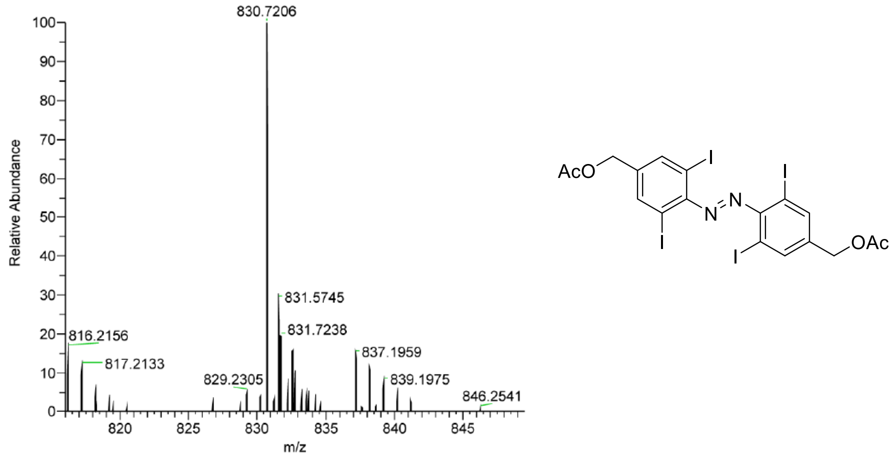

Figure 178. HRMS spectrum of **I_4-OAc.** HRMS-EI (m/z) Calculated for $C_{18}H_{15}N_2O_4I_4$ $[M+H]^+$, 830.7205; found 830.7206.

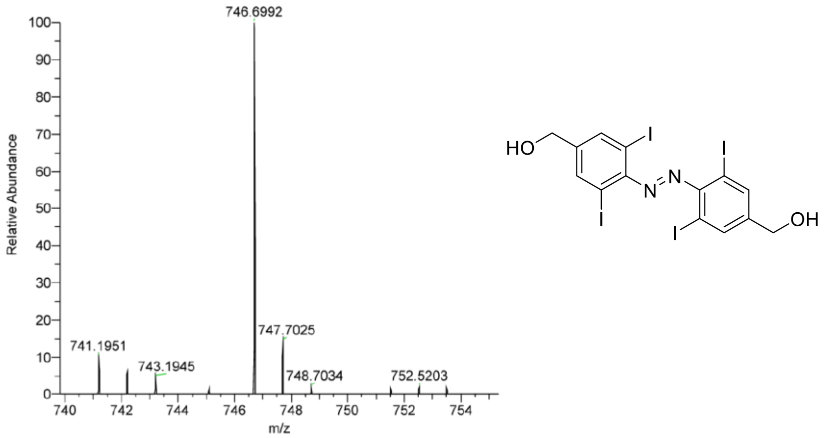

Figure S179. HRMS spectrum of **I_4-OH.** HRMS-EI (m/z) Calculated for $C_{14}H_{11}N_2O_2I_4$ $[M+H]^+$, 746.6994; found 746.6992.

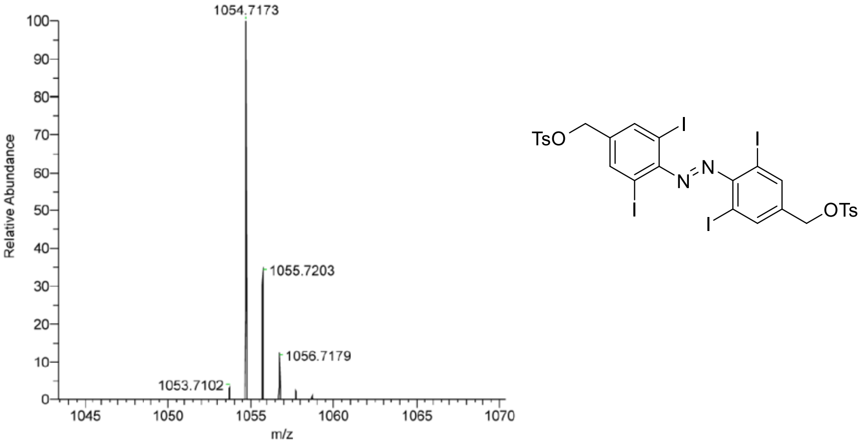

Figure S180. HRMS spectrum of **S25.** HRMS-EI (m/z) Calculated for $C_{28}H_{23}N_2O_6S_2I_4$ $[M+H]^+$, 1054.7171; found 1054.7173.

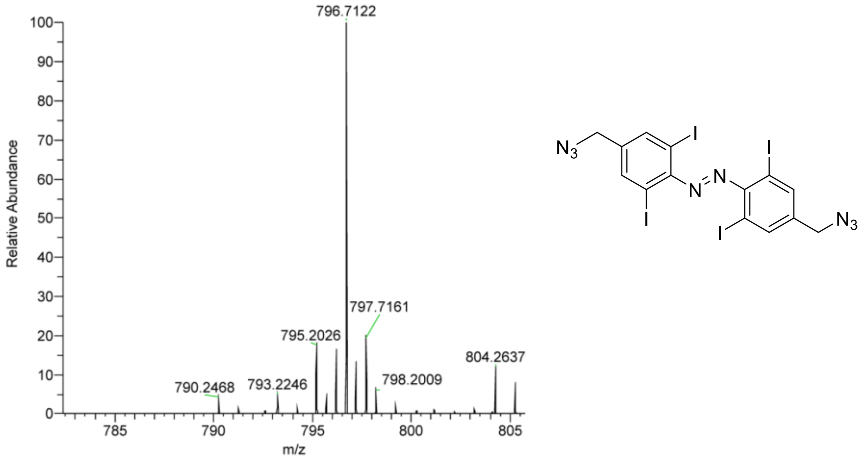

Figure S181. HRMS spectrum of **I_4-N_3.** HRMS-EI (m/z) Calculated for $C_{14}H_9N_8I_4$ $[M+H]^+$, 796.7123; found 796.7122.

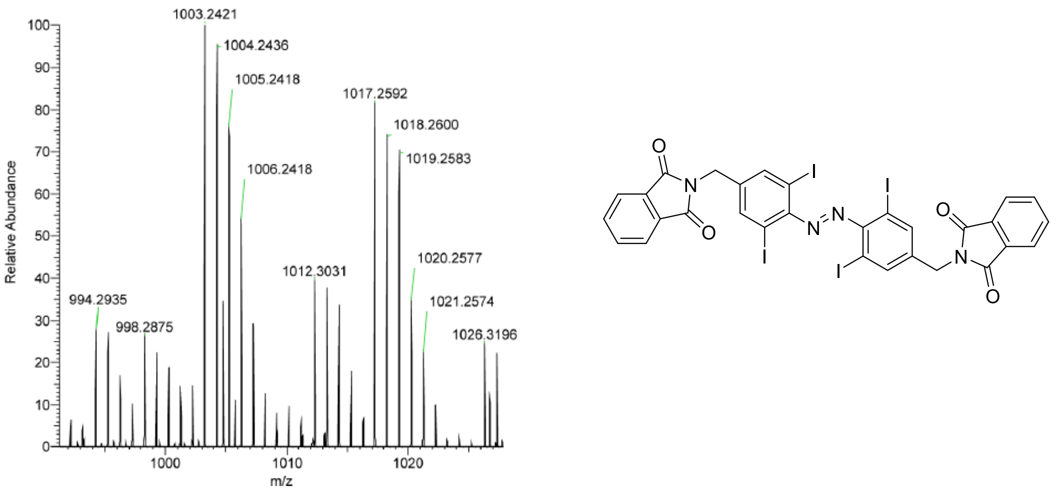

Figure S182. HRMS spectrum of **I_4-NPhth.** HRMS-EI (m/z) Calculated for $C_{30}H_{17}N_4O_4I_4$ $[M+H]^+$, 1004.7423; found 1003.2421.

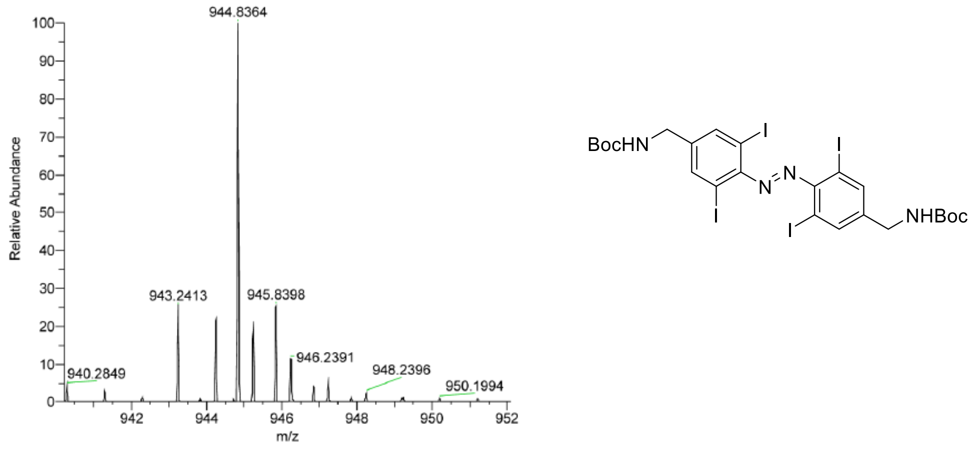

Figure S183. HRMS spectrum of **I_4-NHBoc.** HRMS-EI (m/z) Calculated for $C_{24}H_{29}N_4O_4I_4$ $[M+H]^+$, 944.8362; found 944.8364.

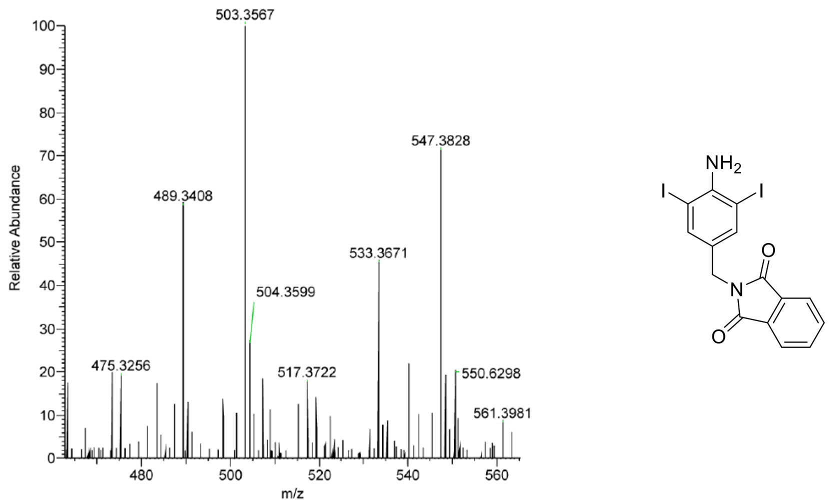

Figure S184. HRMS spectrum of **S28.** HRMS-EI (m/z) Calculated for $C_{15}H_{11}N_2O_2I_2$ $[M+H]^+$, 504.8094; found 503.3567.

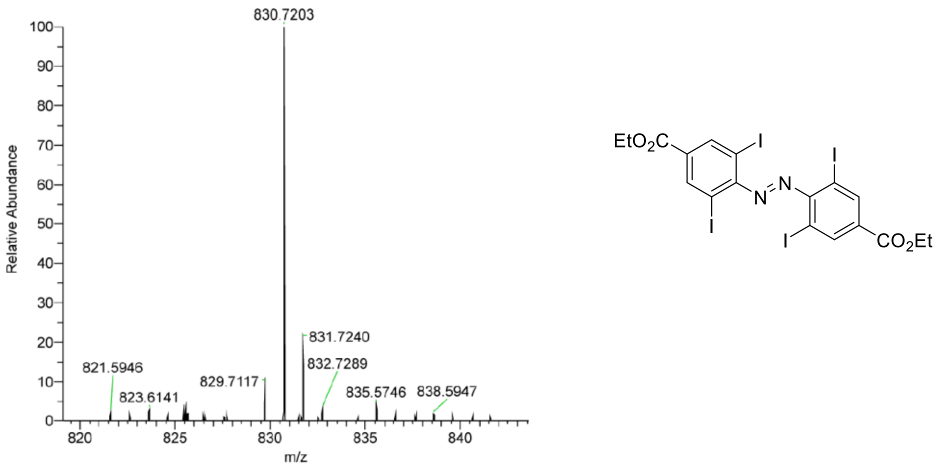

Figure S185. HRMS spectrum of **I_4-Ester.** HRMS-EI (m/z) Calculated for $C_{18}H_{15}N_2O_4I_4$ $[M+H]^+$, 830.7205; found 830.7203.

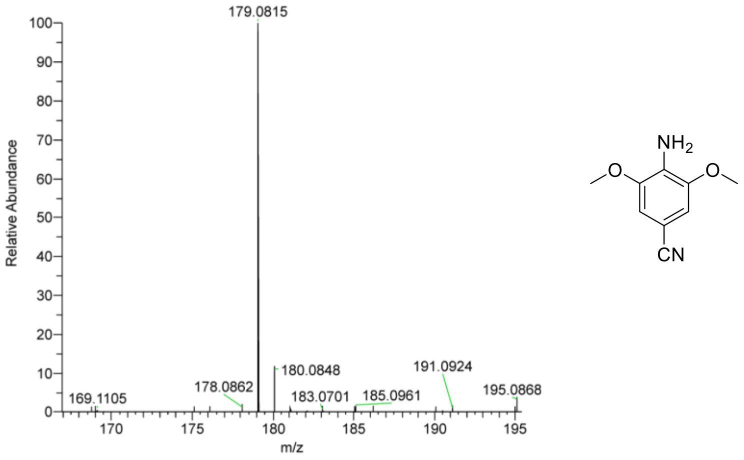

Figure S186. HRMS spectrum of **S33.** HRMS-EI (m/z) Calculated for $C_9H_{11}O_2N_2$ $[M+H]^+$, 179.0815; found 179.0815.

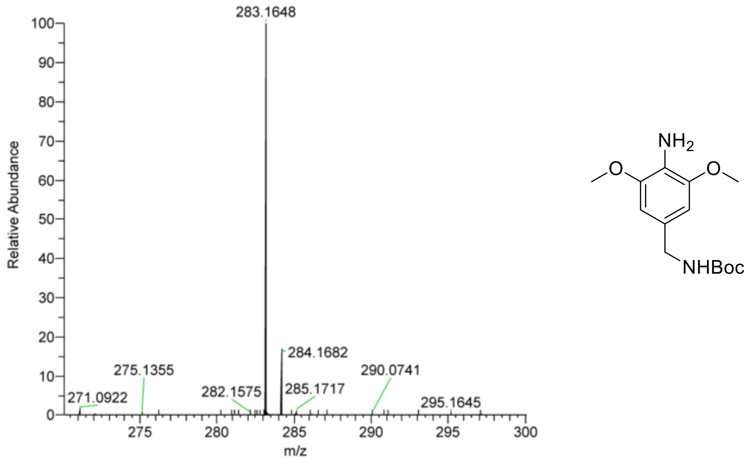

Figure S187. HRMS spectrum of **S35.** HRMS-EI (m/z) Calculated for $C_{14}H_{23}O_4N_2$ $[M+H]^+$, 283.1652; found 283.1648.

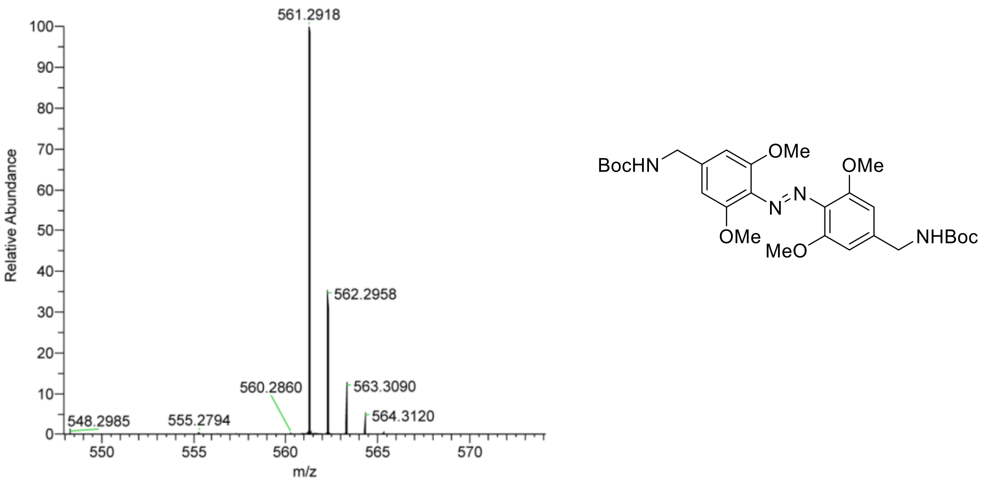

Figure S188. HRMS spectrum of **(OMe)₄-NHBoc.** HRMS-EI (m/z) Calculated for $C_{14}H_{23}O_4N_2$ $[M+H]^+$, 561.2919; found 561.2918.

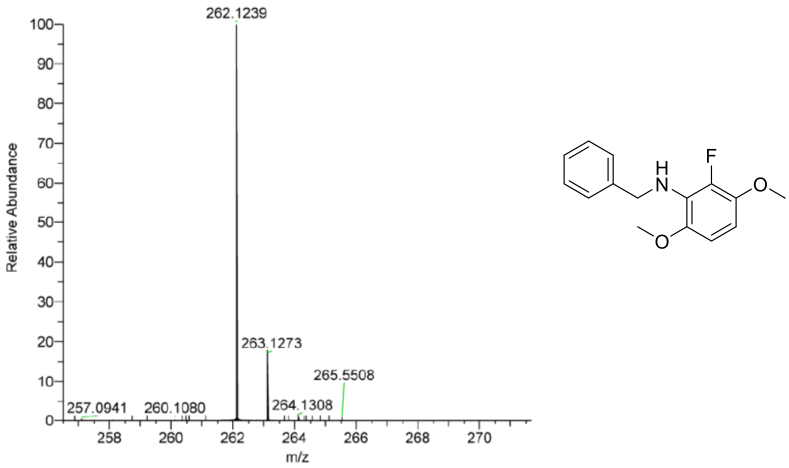

Figure S189. HRMS spectrum of **S38.** HRMS-EI (m/z) Calculated for $C_{15}H_{17}O_2NF$ $[M+H]^+$, 262.1238; found 262.1239

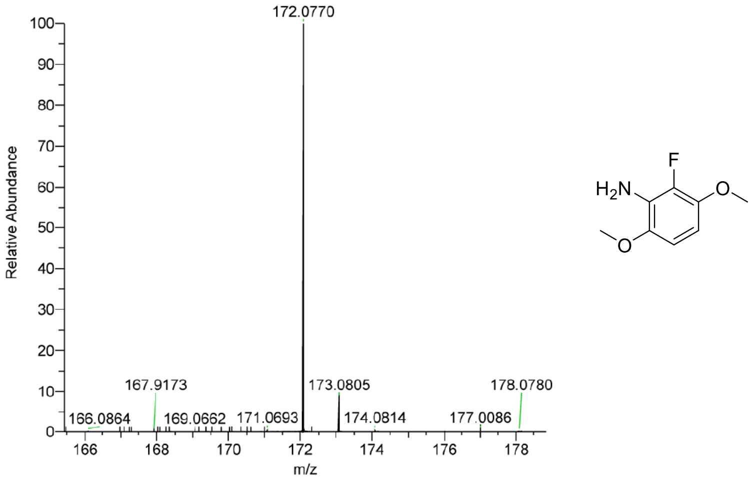

Figure S190. HRMS spectrum of **S39.** HRMS-EI (m/z) Calculated for $C_8H_{11}O_2NF$ $[M+H]^+$, 172.0768; found 172.0770

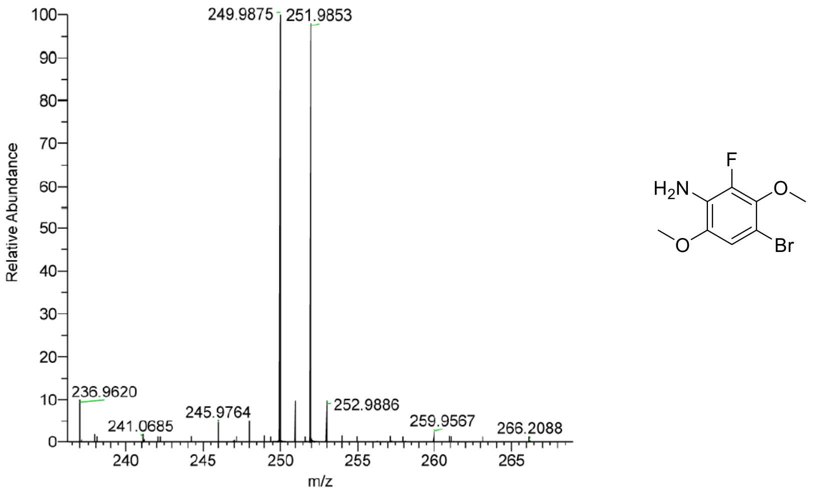

Figure S191. HRMS spectrum of **S40.** HRMS-EI (m/z) Calculated for $C_8H_{10}O_2NBrF$ $[M+H]^+$, 249.9873; found 249.9875.

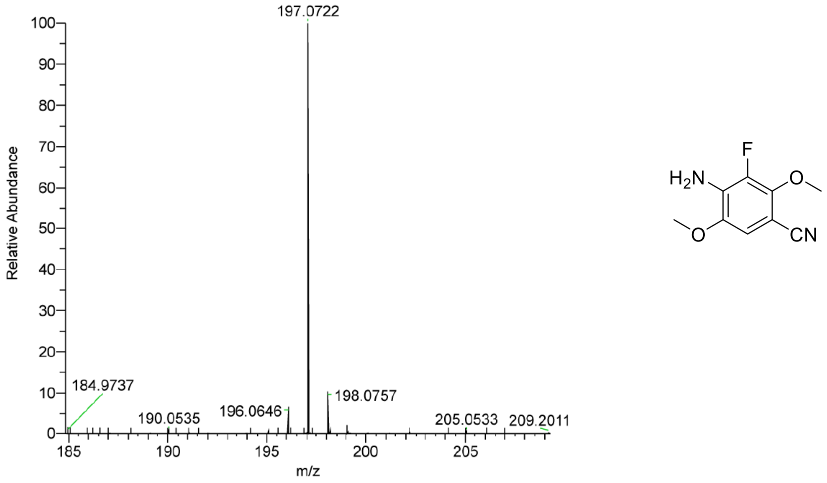

Figure S192. HRMS spectrum of **S41.** HRMS-EI (m/z) Calculated for $C_9H_{10}O_2N_2F$ $[M+H]^+$, 197.0721; found 197.0722.

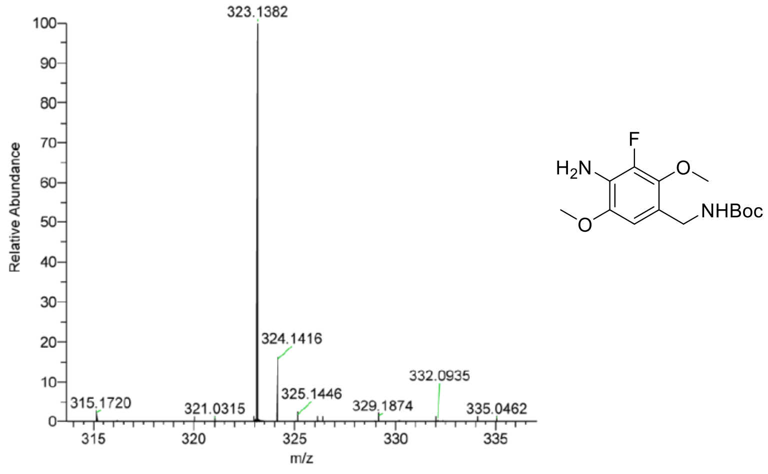

Figure S193. HRMS spectrum of **S42.** HRMS-ESI (m/z) Calculated for $C_{14}H_{21}N_2O_4FNa$ $[M+Na]^+$, 323.1378; found 323.1382.

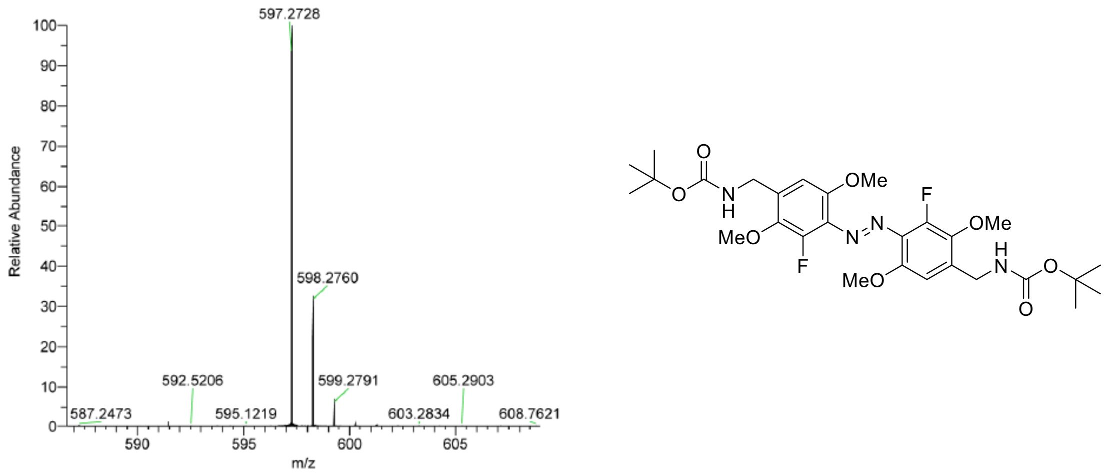

Figure S194. HRMS spectrum of **(OMe)$_2$F$_2$,*m*-OMe$_2$-NHBoc.** HRMS-ESI (m/z) Calculated for $C_{28}H_{39}N_4O_8F_2Na$ $[M+H]^+$, 597.2730; found 597.2728.

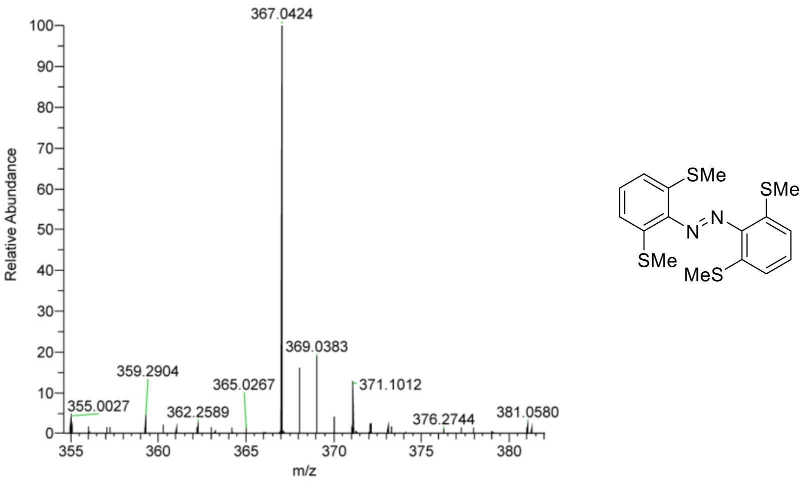

Figure S195. HRMS spectrum of **(SMe$_4$)-H.** HRMS-EI (m/z) Calculated for $C_{16}H_{19}N_2S_4$ $[M+H]^+$, 367.0426; found 367.0424

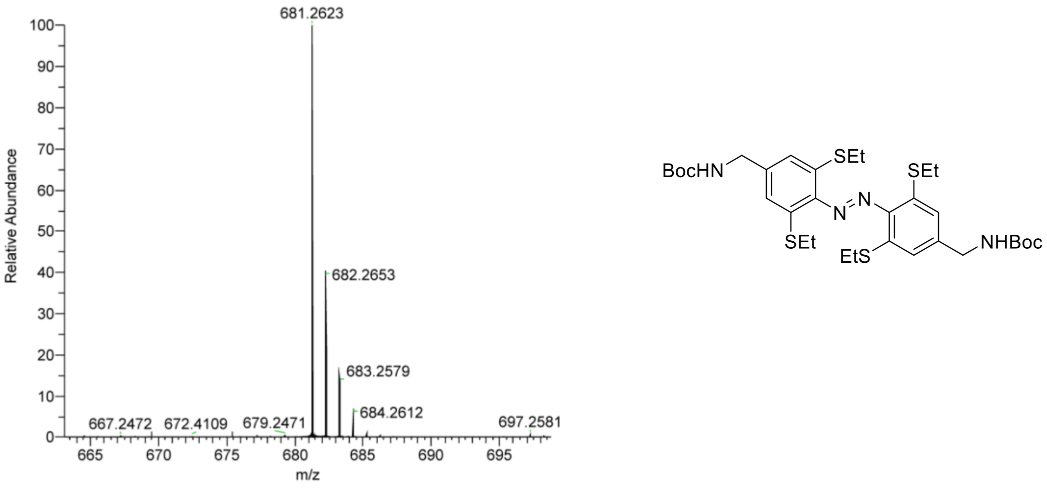

Figure S196. HRMS spectrum of **(SEt)$_4$-NHBoc.** HRMS-EI (m/z) Calculated for $C_{32}H_{49}O_4N_4S_4$ $[M+H]^+$, 681.2631; found 681.2623

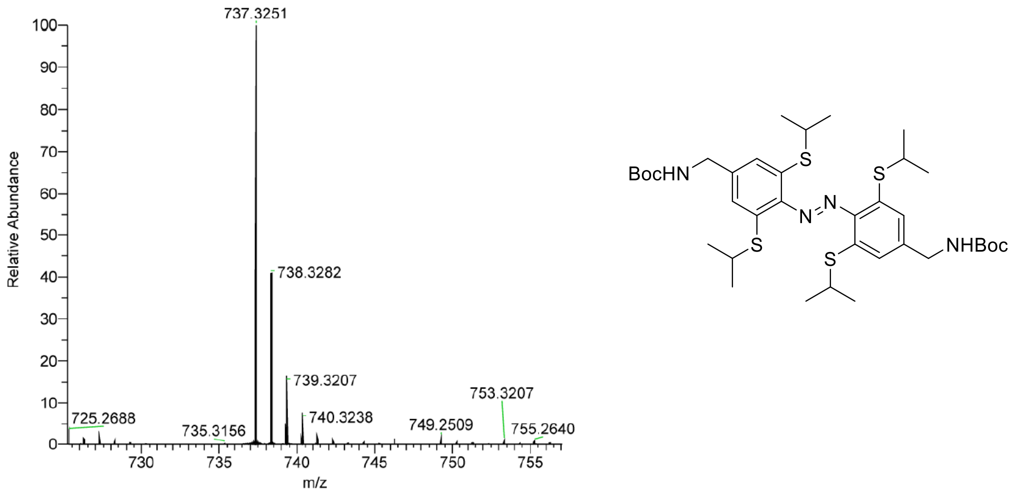

Figure S197. HRMS spectrum of **(S^{i}Pr)$_4$-NHBoc.** HRMS-EI (m/z) Calculated for $C_{36}H_{57}O_4N_4S_4$ $[M+H]^+$, 737.3251; found 737.3257

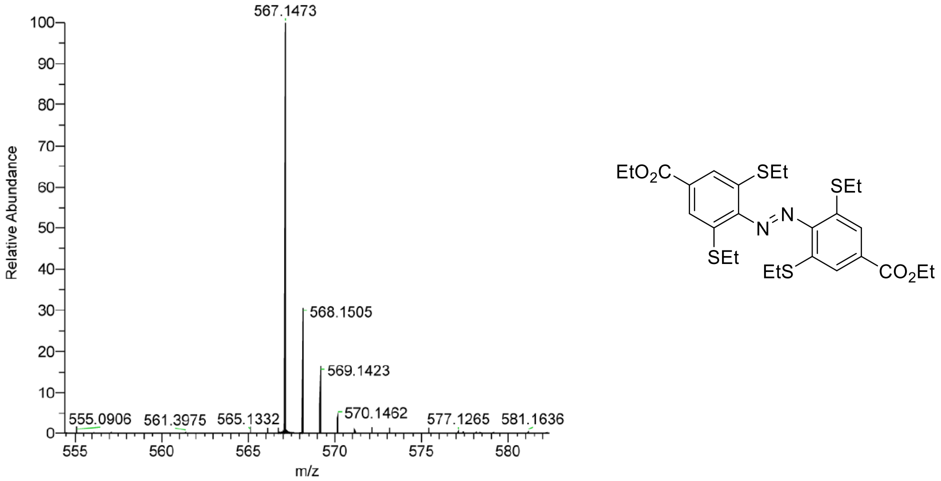

Figure S198. HRMS spectrum of **(SEt)$_4$-ester.** HRMS-EI (m/z) Calculated for $C_{26}H_{35}O_4N_2S_4$ $[M+H]^+$, 567.1474; found 567.1473

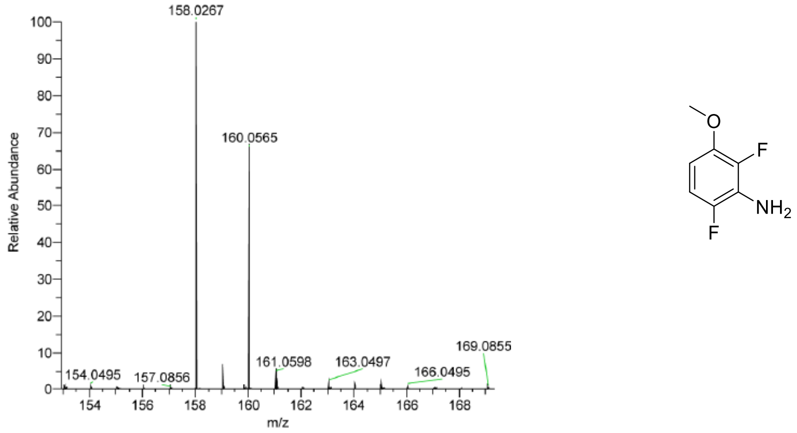

Figure S199. HRMS spectrum of **S47.** HRMS-APCI (m/z) Calculated for $C_7H_8ONF_2$ $[M+H]^+$, 160.0568; found 160.0565.

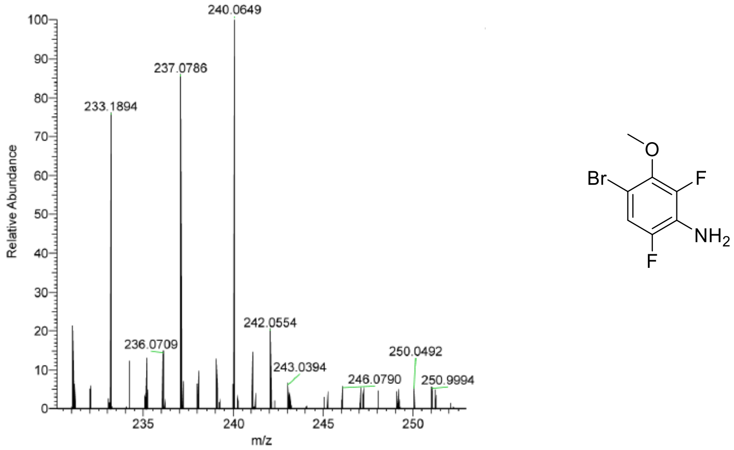

Figure S200. HRMS spectrum of **S48.** HRMS-APCI (m/z) Calculated for $C_7H_6ONF_2Br$ $[M+H]^+$, 239.9653; found 240.0649.

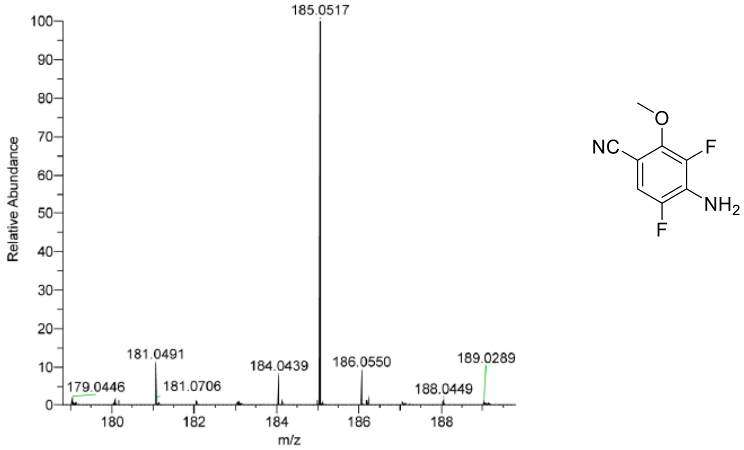

Figure S201. HRMS spectrum of **S49.** HRMS-APCI (m/z) Calculated for $C_8H_7ONF_2$ $[M+H]^+$, 185.0521; found 185.0517.

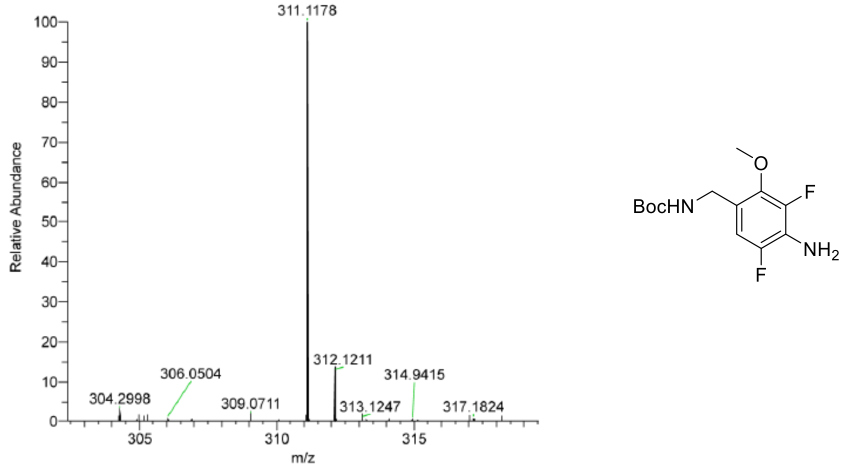

Figure S202. HRMS spectrum of **S50.** HRMS-EI (m/z) Calculated for $C_{13}H_{18}F_2N_2O_2Na$ $[M+Na]^+$, 311.1178; found 311.1178.

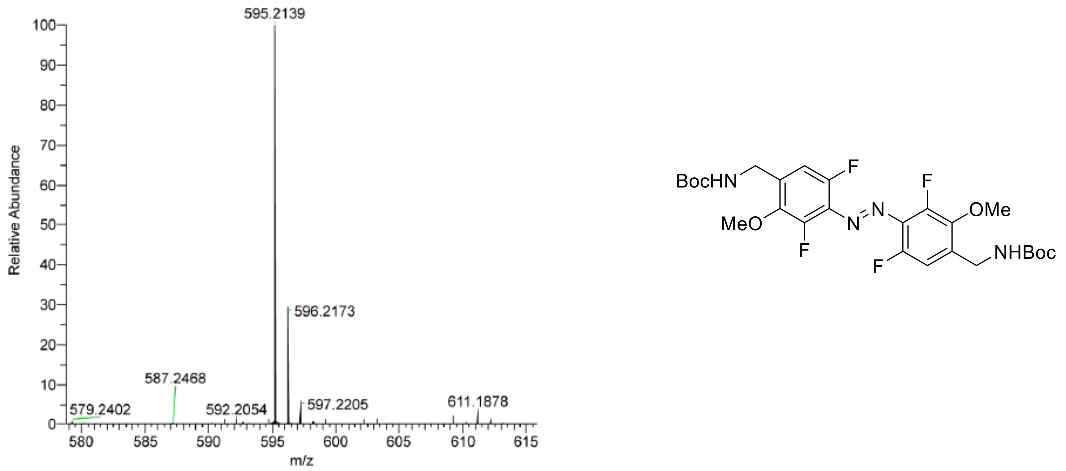

Figure S203. HRMS spectrum of **F$_4$,*m*-OMe$_2$-NHBoc.** HRMS-EI (m/z) Calculated for $C_{26}H_{32}F_4N_4O_6Na$ [M+Na]$^+$, 595.2150; found 595.2139.

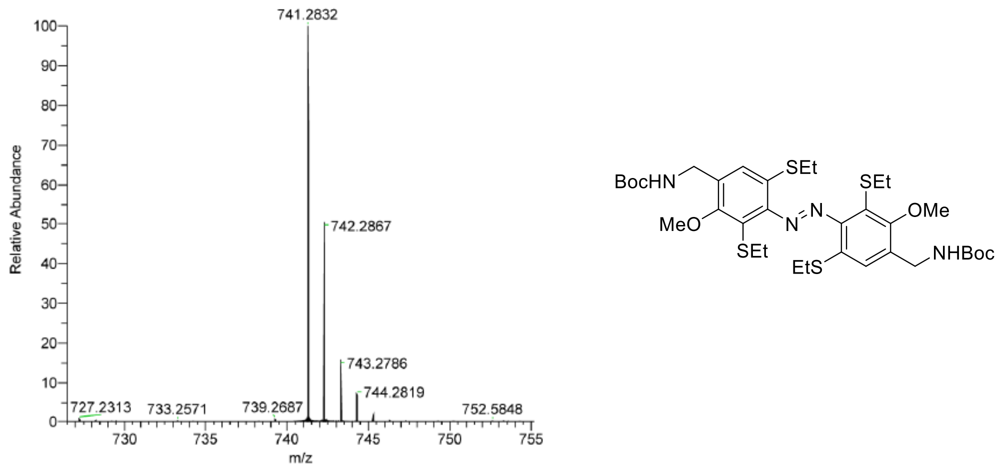

Figure S204. HRMS spectrum of **(SEt)$_4$,*m*-OMe$_2$-NHBoc.** HRMS-EI (m/z) Calculated for $C_{34}H_{53}S_4N_4O_6$ [M+H]$^+$, 741.2842; found 741.2832.

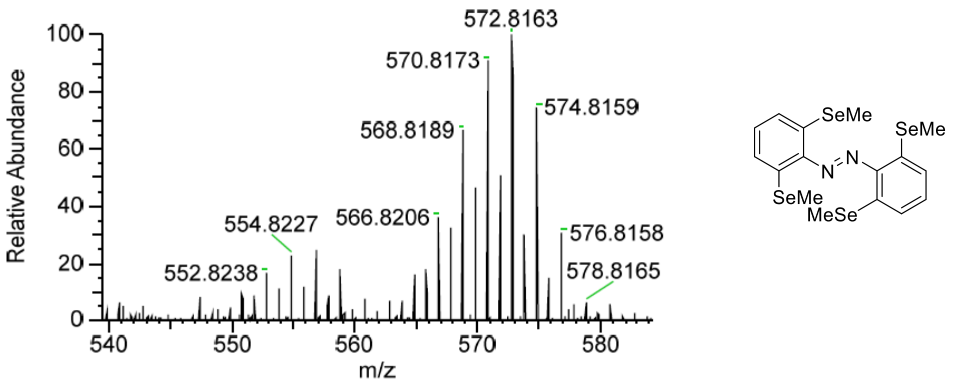

Figure S205. HRMS spectrum of **(SeMe$_4$)-H.** HRMS-EI (m/z) Calculated for $C_{16}H_{19}N_2Se_4$ [M+H]$^+$, 554.8219; found 554.8227.

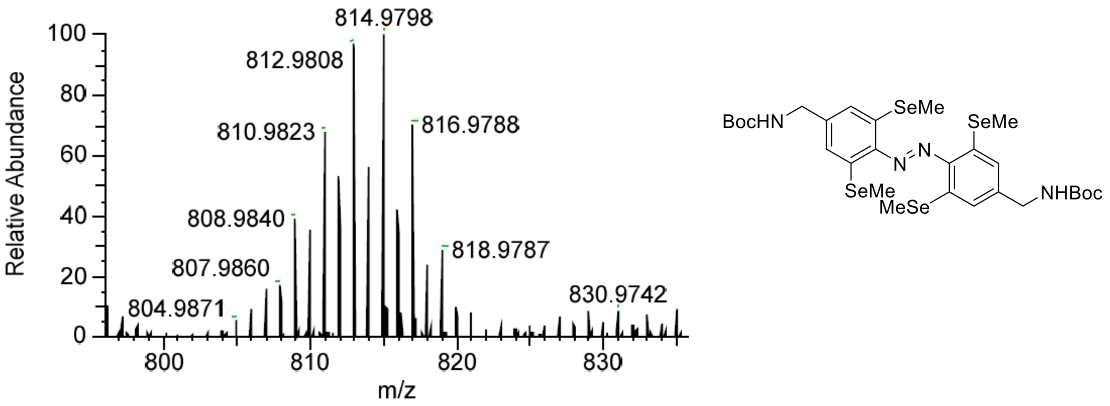

Figure S206. HRMS spectrum of **(SeMe$_4$)-NHBoc.** HRMS-EI (m/z) Calculated for $C_{28}H_{40}N_4O_4Se_4$ $[M+H]^+$, 814.9791; found 814.9798.

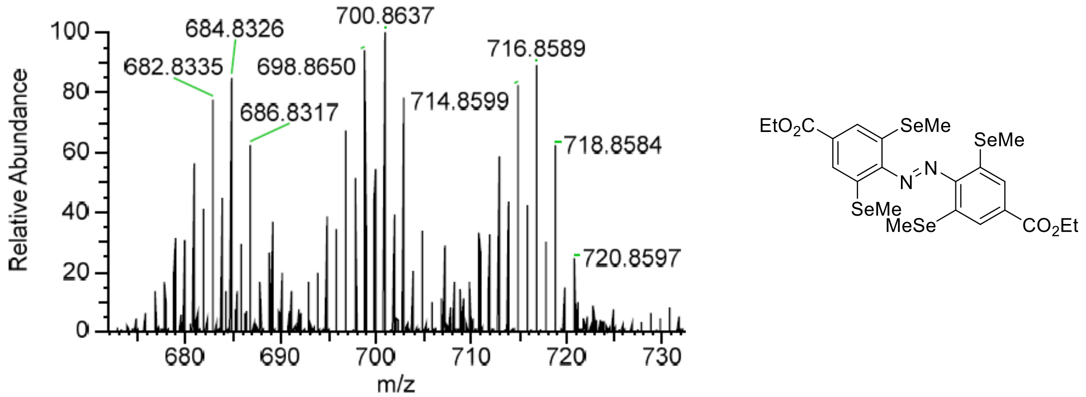

Figure S207. HRMS spectrum of **(SeMe$_4$)-ester.** HRMS-EI (m/z) Calculated for $C_{22}H_{27}N_2O_4Se_4$ $[M+H]^+$, 700.8634; found 700.8637.

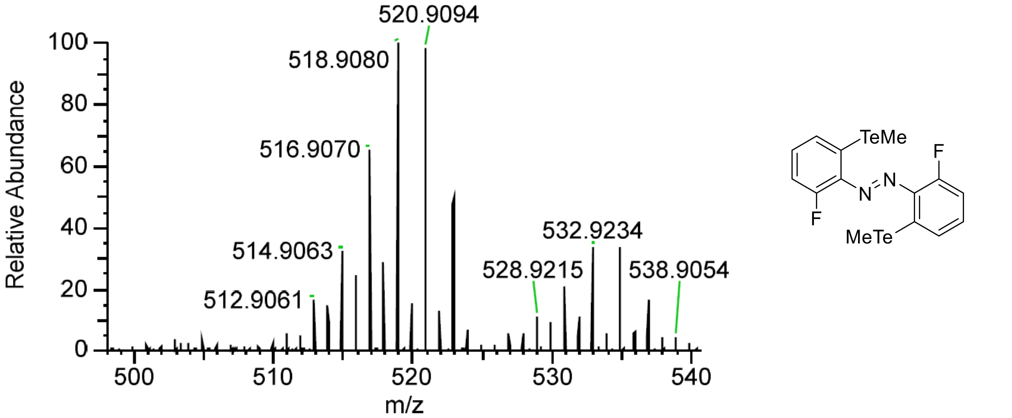

Figure S208. HRMS spectrum of **Te$_2$F$_2$-H.** HRMS-EI (m/z) Calculated for $C_{14}H_{12}N_2F_2Te_2OH$ $[M+O+H]^+$, 520.9097; found 520.9094.

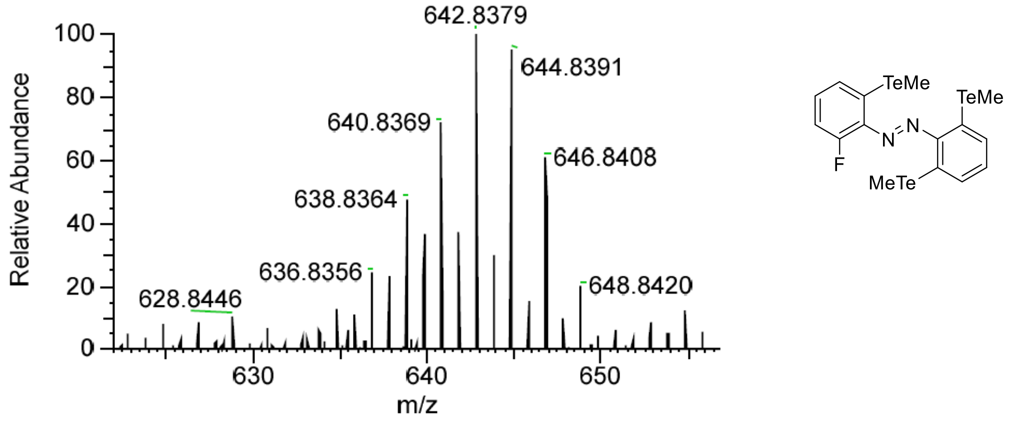

Figure S209. HRMS spectrum of **Te_3F-H.** HRMS-EI (m/z) Calculated for $C_{15}H_{15}N_2FTe_3OH$ $[M+O+H]^+$, 642.8381; found 642.8379.

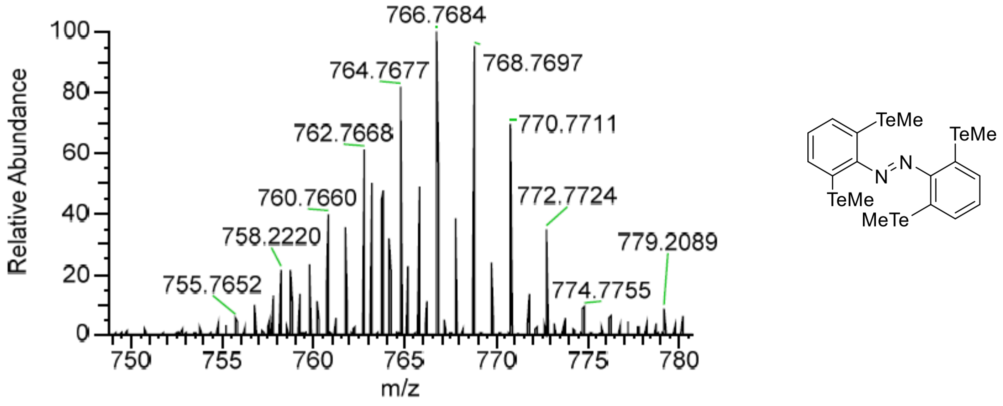

Figure S210. HRMS spectrum of **(TeMe)$_4$-H.** HRMS-EI (m/z) Calculated for $C_{16}H_{18}N_2Te_4OH$ $[M+H+O]^+$, 766.7676; found 766.7682.

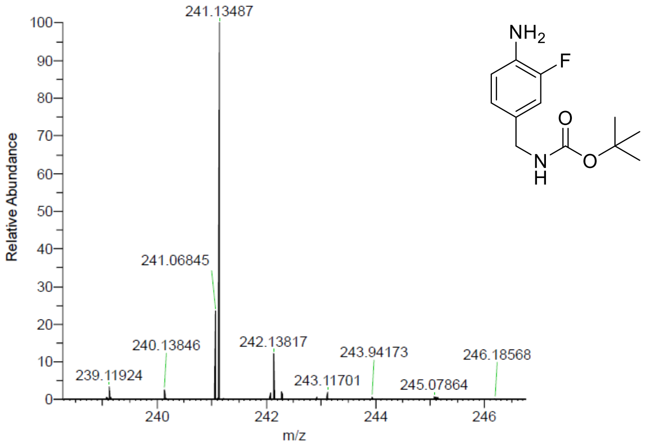

Figure S211. HRMS spectrum of **S53.** HRMS-ESI (m/z) . HRMS-ESI (m/z) Calculated for $C_{12}H_{18}FN_2O_2$ $[M+H]^+$, 241.1347; found 241.1349.

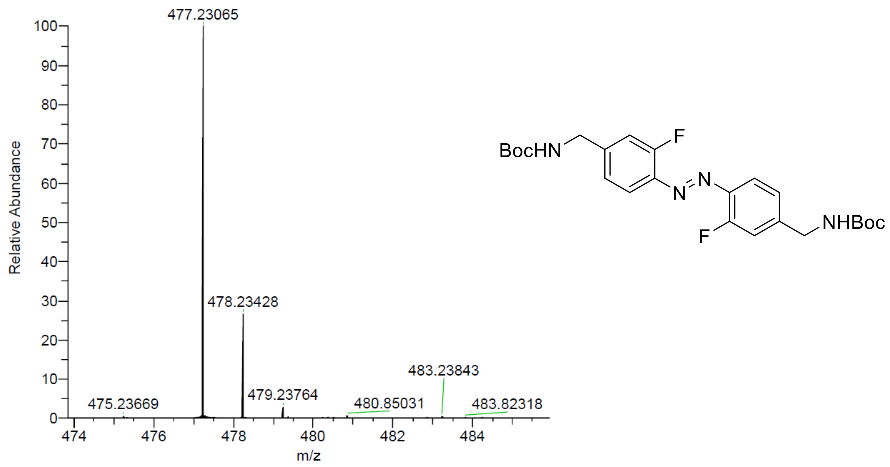

Figure S212. HRMS spectrum of **F_2,*m*-H_2.** HRMS-ESI (m/z) Calculated for $C_{24}H_{30}F_2N_4O_4$ $[M+H]^+$, 477.23079; found 477.23065.

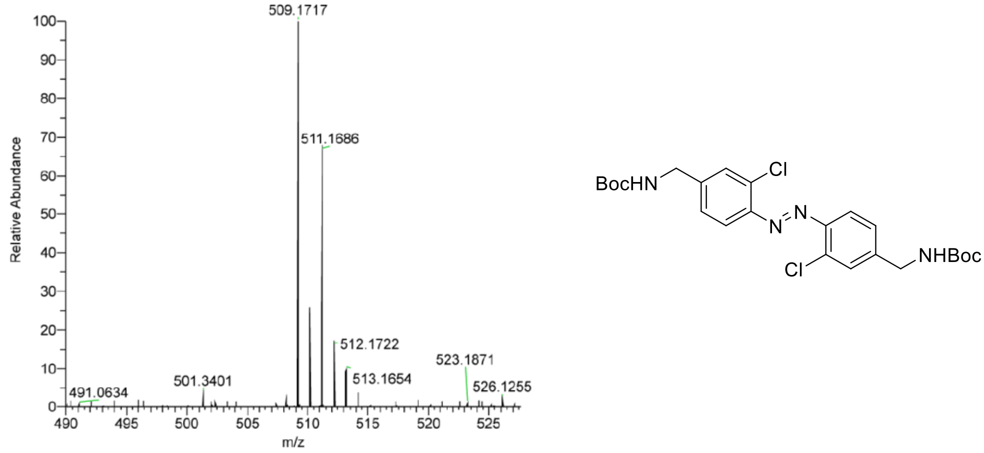

Figure S213. HRMS spectrum of **Cl_2,*m*-H_2.** HRMS-EI (m/z) Calculated for $C_{24}H_{31}O_4N_4Cl_2$ $[M+H]^+$, 509.1717; found 509.1717

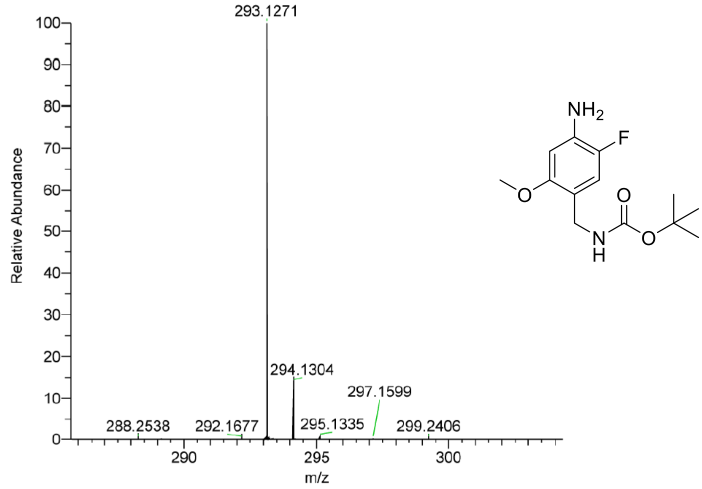

Figure S214. HRMS spectrum of **S59.** HRMS-EI (m/z) Calculated for $C_{13}H_{19}O_3N_2FNa$ $[M+Na]^+$, 293.1272; found 293.1271

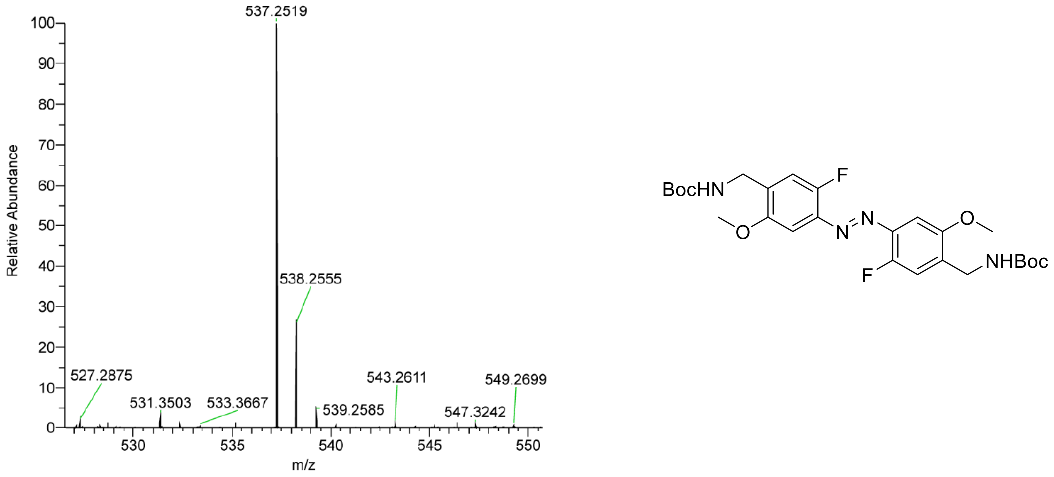

Figure S215. HRMS spectrum of **F_2,*m*-OMe$_2$.** HRMS-EI (m/z) Calculated for $C_{26}H_{35}O_6N_4F_2$ $[M+H]^+$, 537.2519; found 537.2519

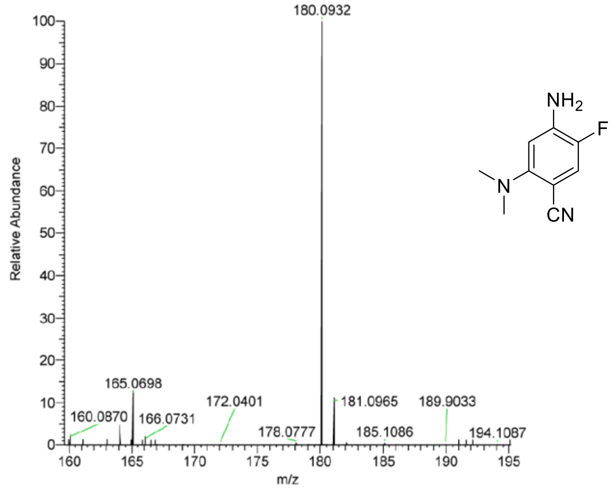

Figure S216. HRMS spectrum of **S61.** HRMS-EI (m/z) Calculated for $C_9H_{11}FN_3$ $[M+H]^+$, 180.0937; found 180.0932

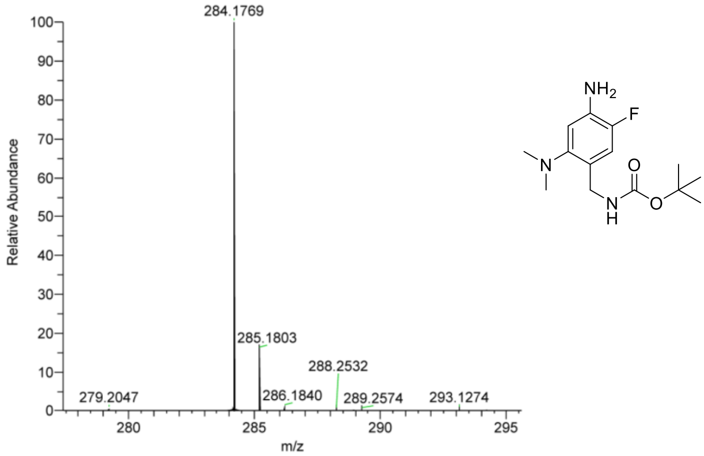

Figure S217. HRMS spectrum of **S70b.** HRMS-EI (m/z) Calculated for $C_{14}H_{23}O_2N_3F$ $[M+H]^+$, 284.1769; found 284.1769

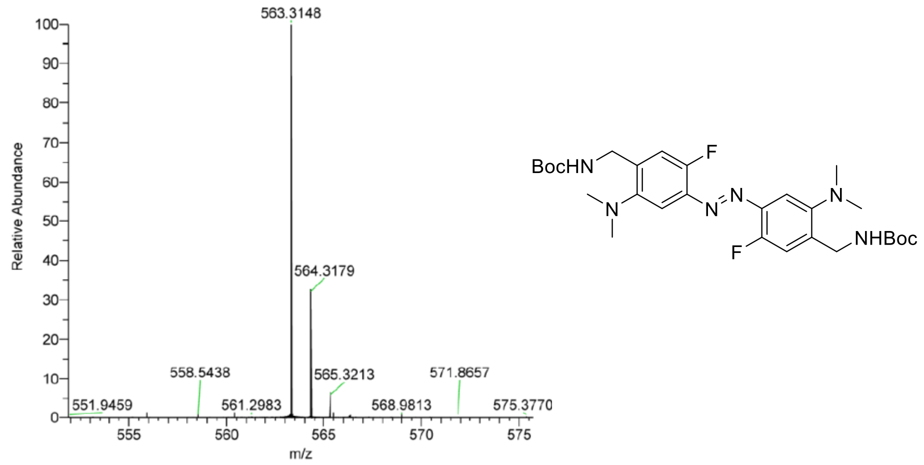

Figure S218. HRMS spectrum of **F_2,*m*-$(NMe_2)_2$.** HRMS-EI (m/z) Calculated for $C_{28}H_{41}O_4N_6F_2$ $[M+H]^+$, 563.3152; found 563.3148

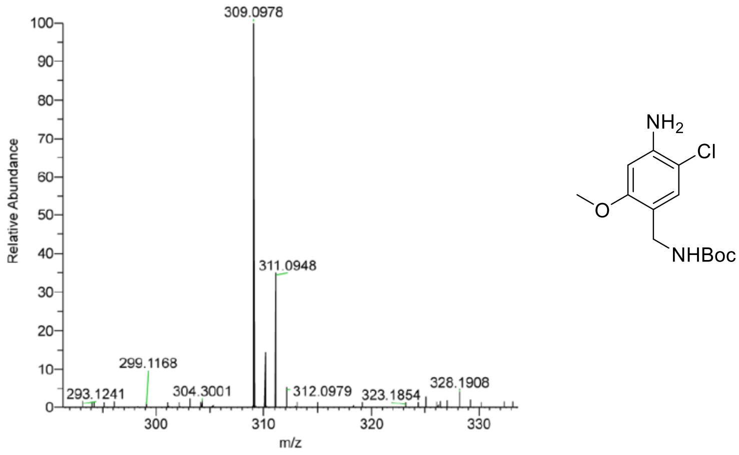

Figure S219. HRMS spectrum of **70c.** HRMS-ESI (m/z) Calculated for $C_{13}H_{19}ClN_2O_3Na$ $[M+Na]^+$, 309.0975; found 309.0978.

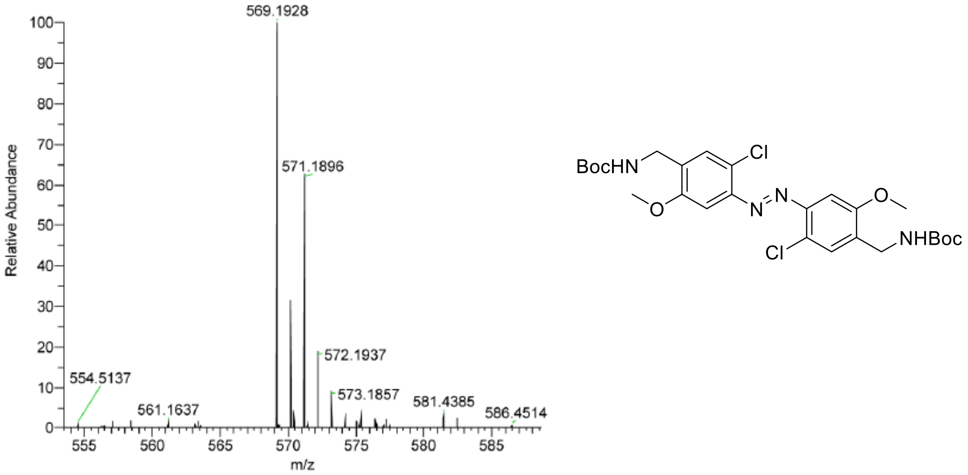

Figure S220. HRMS spectrum of **Cl_2,*m*-OMe_2.** HRMS-ESI (m/z) Calculated for $C_{26}H_{35}Cl_2N_4O_6$ $[M+H]^+$, 569.1928; found 569.1928.

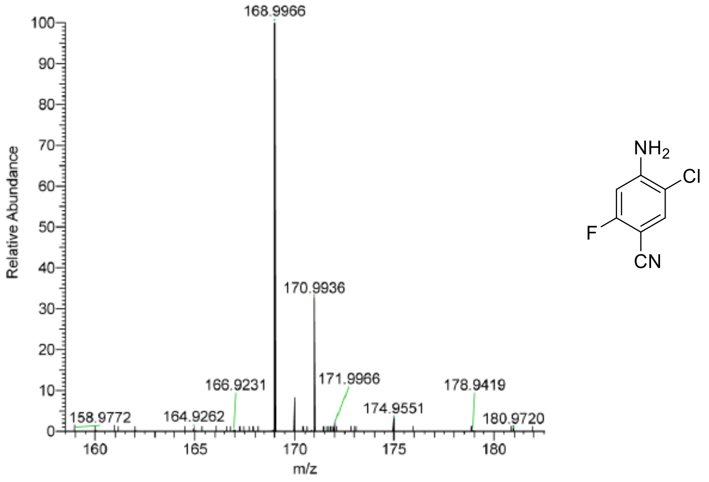

Figure S221. HRMS spectrum of **S67.** HRMS-EI (m/z) Calculated for $C_7H_3N_2FCl$ $[M-H]^-$, 168.9974; found 168.9966

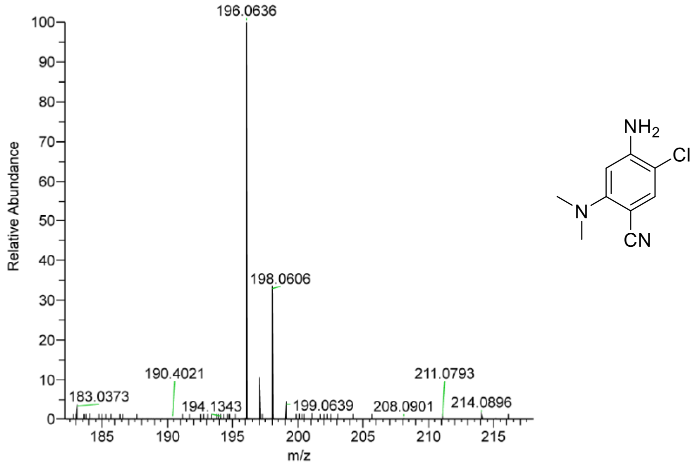

Figure S222. HRMS spectrum of **S68.** HRMS-EI (m/z) Calculated for $C_9H_{11}N_3Cl$ $[M+H]^+$, 196.0636; found 196.0636

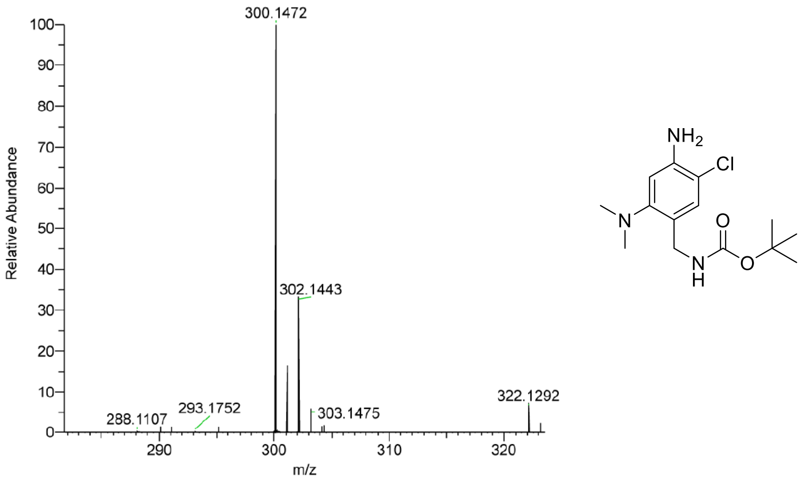

Figure S223. HRMS spectrum of **S70d.** HRMS-EI (m/z) Calculated for $C_{14}H_{23}O_2N_3Cl$ $[M+H]^+$, 300.1473; found 300.1472

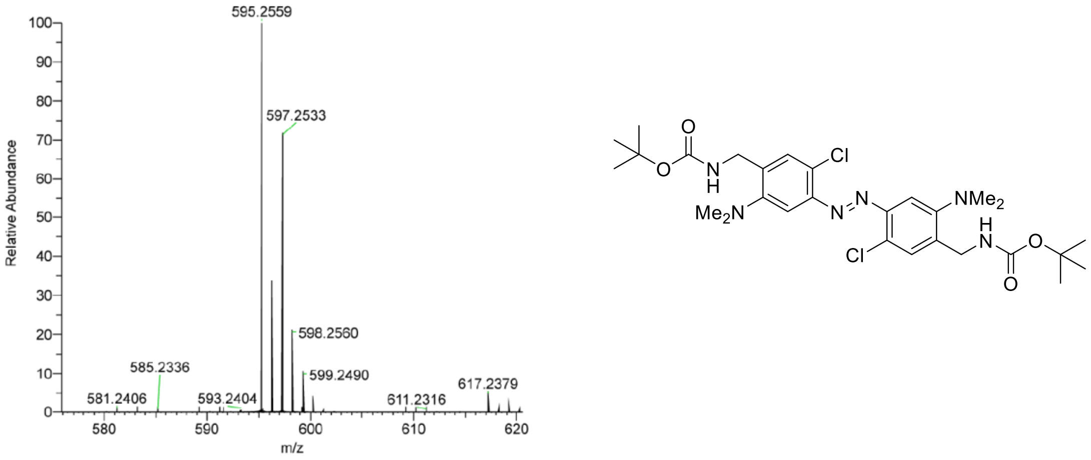

Figure S224. HRMS spectrum of **Cl_2,*m*-(NMe_2)$_2$.** HRMS-EI (m/z) Calculated for $C_{28}H_{41}O_4N_6Cl_2$ $[M+H]^+$, 595.2561; found 595.2559

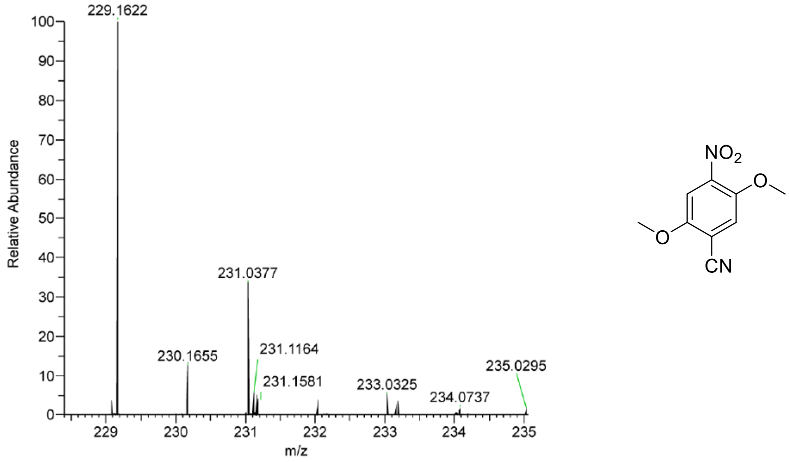

Figure S225. HRMS spectrum of **S74.** HRMS-EI (m/z) Calculated for $C_9H_8O_4N_2Na$ $[M+Na]^+$, 231.0376; found 231.0377.

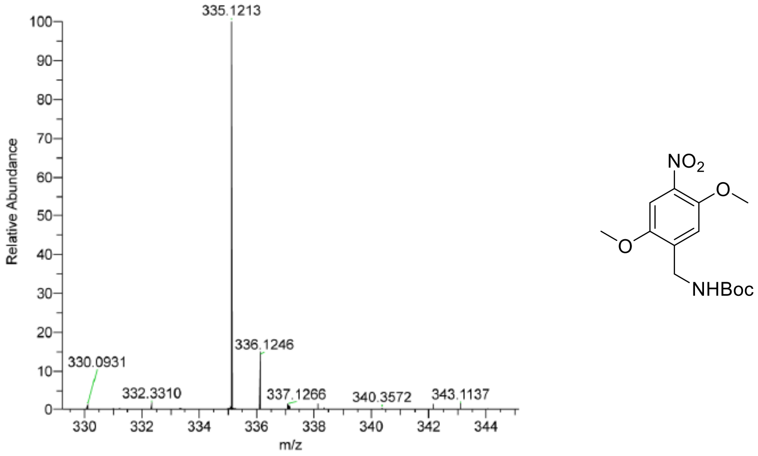

Figure S226. HRMS spectrum of **S75.** HRMS-EI (m/z) Calculated for $C_{14}H_{20}O_6N_2Na$ $[M+Na]^+$, 335.1214; found 335.1213

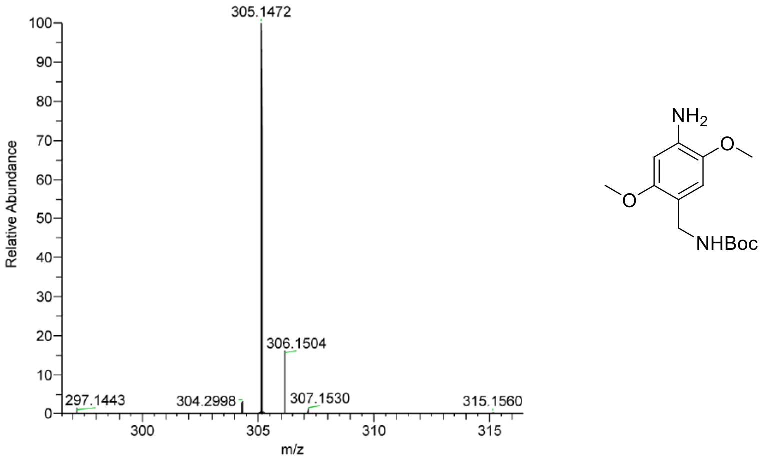

Figure S227. HRMS spectrum of **S76.** HRMS-EI (m/z) Calculated for $C_{14}H_{22}O_4N_2Na$ [M+Na]$^+$, 305.1472; found 305.1472

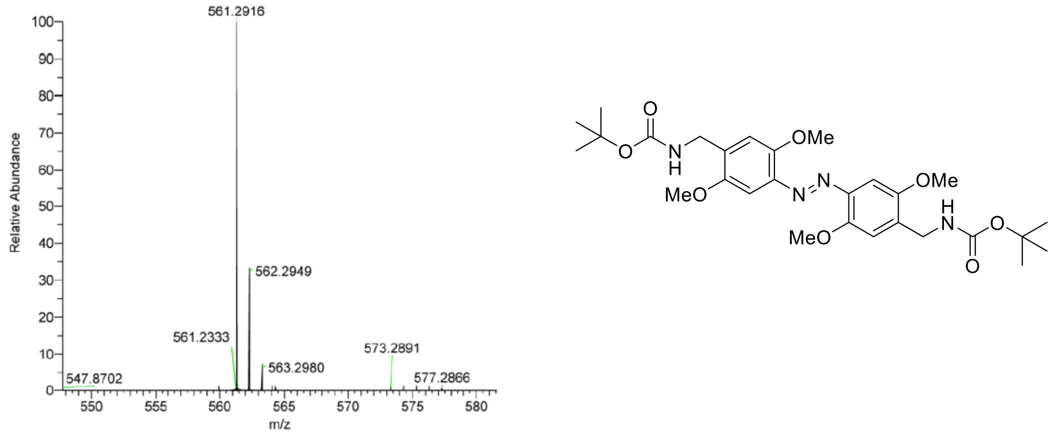

Figure S228. HRMS spectrum of **OMe₂,*m*-OMe.** HRMS-EI (m/z) Calculated for $C_{28}H_{41}O_8N_4$ [M+H]$^+$, 561.2919; found 561.2916

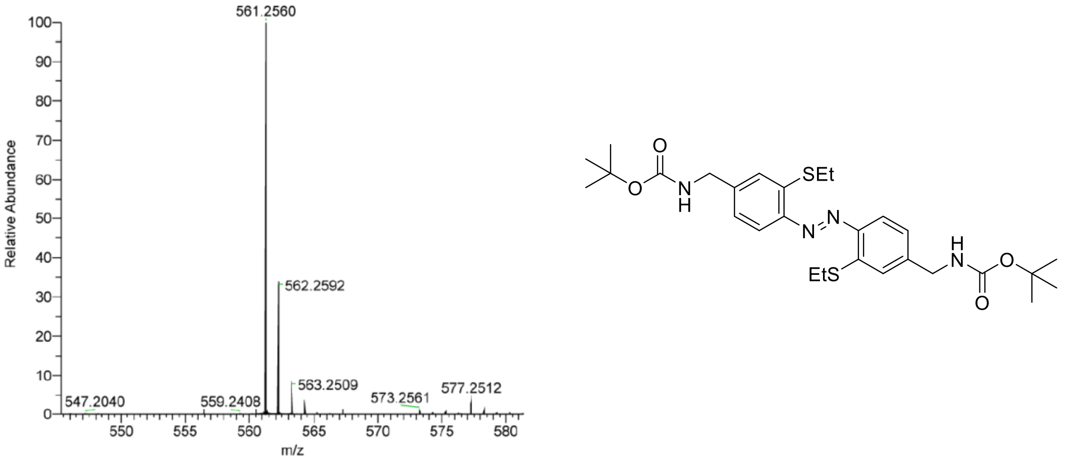

Figure S229. HRMS spectrum of **(SEt)₂,*m*-H₂.** HRMS-EI (m/z) Calculated for $C_{28}H_{41}O_4N_4S_2$ [M+H]$^+$, 561.2564; found 561.2560

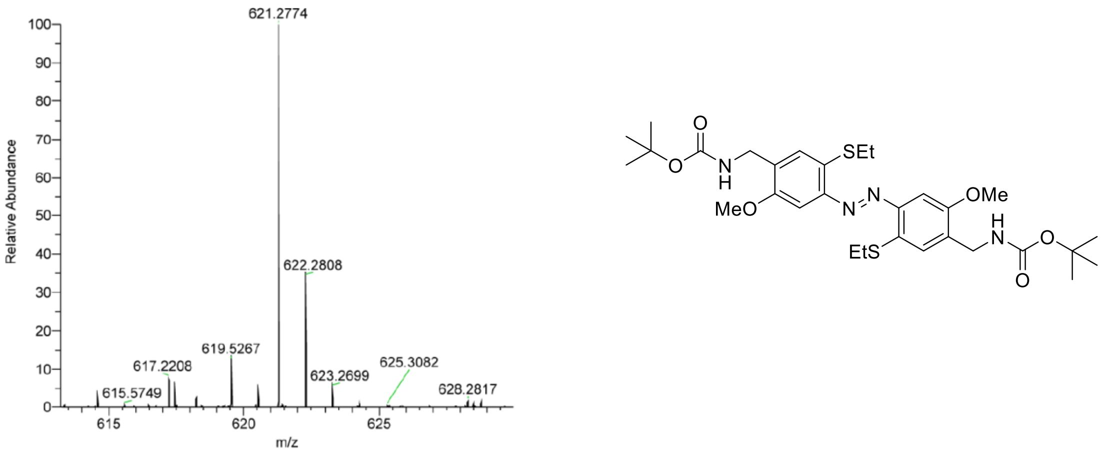

Figure S230. HRMS spectrum of **(SEt)$_2$,*m*-OMe$_2$.** HRMS-EI (m/z) Calculated for $C_{30}H_{45}O_6N_4S_2$ $[M+H]^+$, 625.2775; found 625.2774

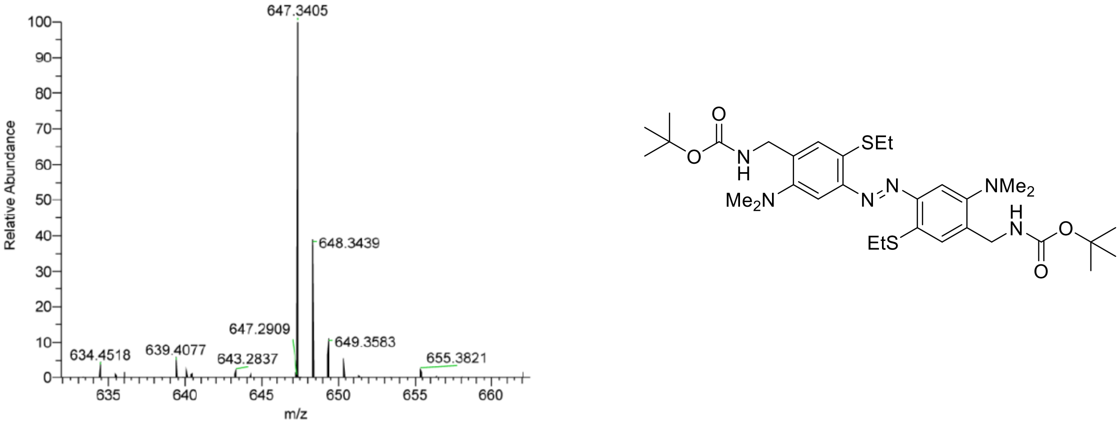

Figure S231. HRMS spectrum of **(SEt$_2$),*m*-(NMe$_2$)$_2$.** HRMS-EI (m/z) Calculated for $C_{32}H_{51}O_4N_6S_2$ $[M+H]^+$, 647.3408; found 647.3405

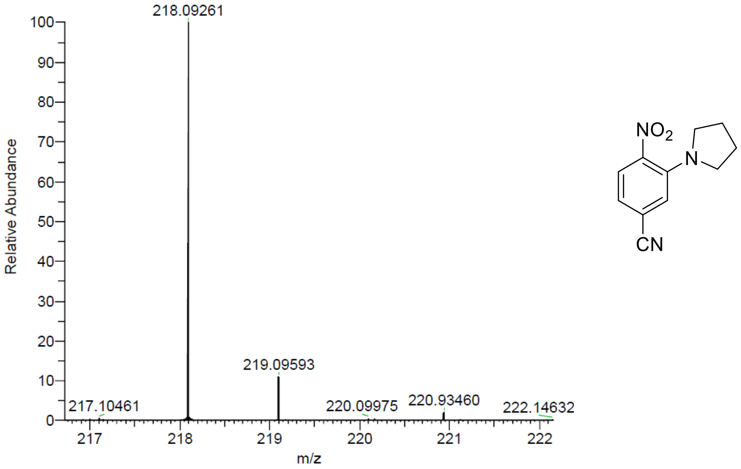

Figure S232. HRMS spectrum of **S78.** HRMS-ESI (m/z) Calculated for $C_{11}H_{11}N_3O_2$ $[M+H]^+$, 218.09240; found 218.09261.

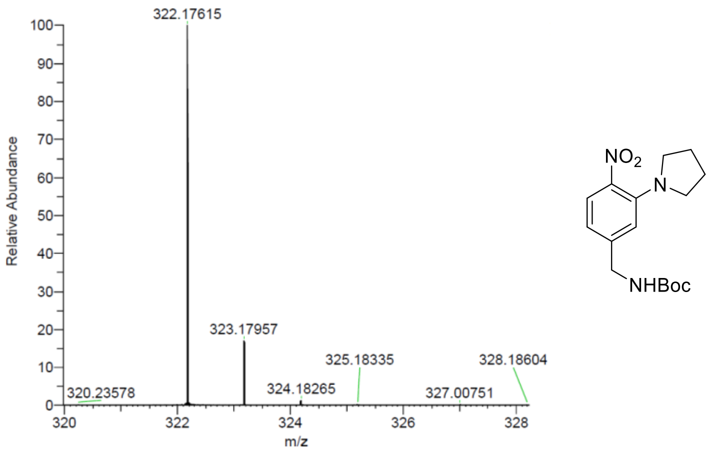

Figure S233. HRMS spectrum of **S79.** HRMS-ESI (m/z) Calculated for $C_{16}H_{23}N_3O_4$ $[M+H]^+$, 322.17613; found 322.17615.

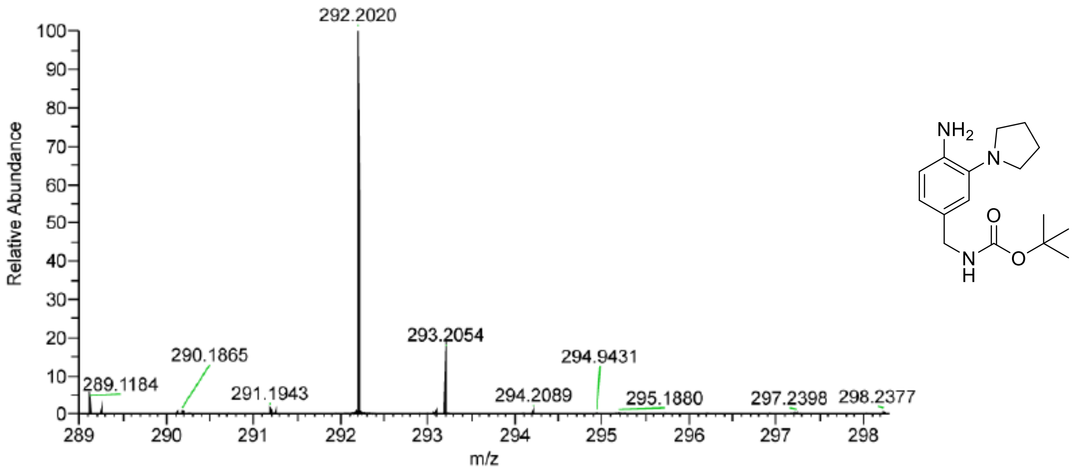

Figure S234. HRMS spectrum of **S80.** HRMS-ESI (m/z) Calculated for $C_{16}H_{25}N_3O_2$ $[M+H]^+$, 292.2020; found 292.2020.

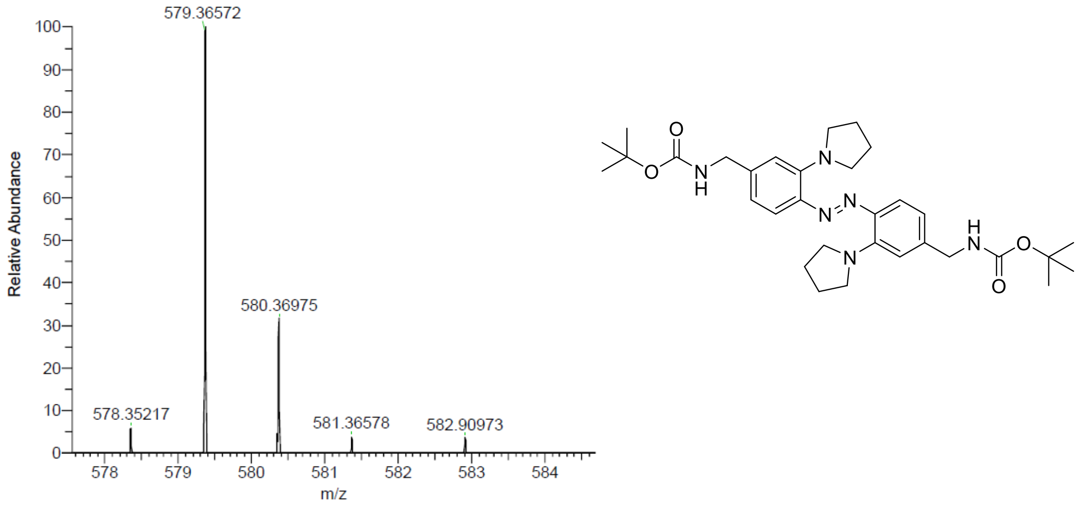

Figure S235. HRMS spectrum of **$(N(CH_2)_4)_2$,*m*-H.** HRMS-EI (m/z) Calculated for $C_{32}H_{47}O_4N_6$ $[M+H]^+$, 579.3653; found 579.3657

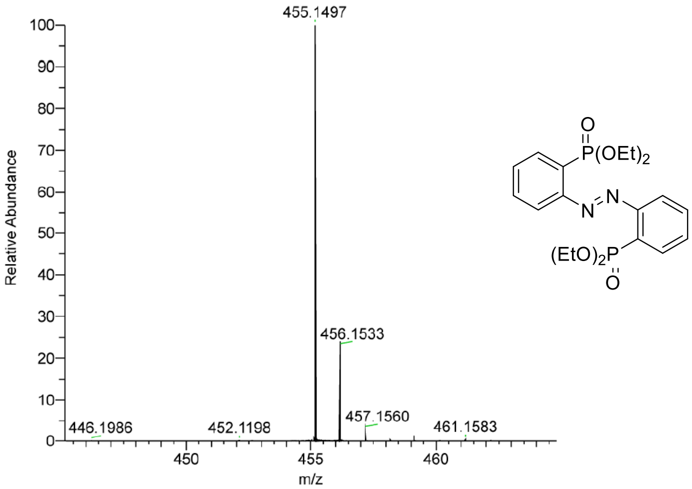

Figure S236. HRMS spectrum of **S82**. HRMS-EI (m/z) Calculated for $C_{20}H_{29}N_2O_6P_2$ $[M+H]^+$, 455.1495; found 455.1497.

3. UV-Vis spectra

All UV-vis spectra were determined in DMSO solution. Extinction coefficients were determined by recording a UV-vis spectra for the *E* isomer at 10, 20, 30, 40 µM in DMSO respectively. The absorbance at the maximum of the π - π* transition of the *E*-isomers was plotted against concentration (Beer-Lambert plot) to determine the molar extinction coefficient ε. For each slow relaxing compound, the *E* isomer sample at 40 µM was irradiated with the appropriate wavelength of light to generate the photo-stationary state, and another spectrum was run. This spectrum was normalised to units of ε and overlaid with the dark (100% *E* isomer) spectrum.

Deconvoluted spectra were prepared using Origin Pro 2020b by fitting each λ_{max} to the "Multiple Peak Fit" function.

Photo-irradiation of liquid samples was carried out using Thorlabs high-power mounted LEDs (models M730L4, (NIR, 730 nm), M660L4 (red, 660 nm) M625L4 (red, 625 nm); M590L4 (Amber, 590 nm) M530L4 (green, 530 nm); in-house custom built set-ups using optical components supplied by Thorlabs, as described in reference [2]

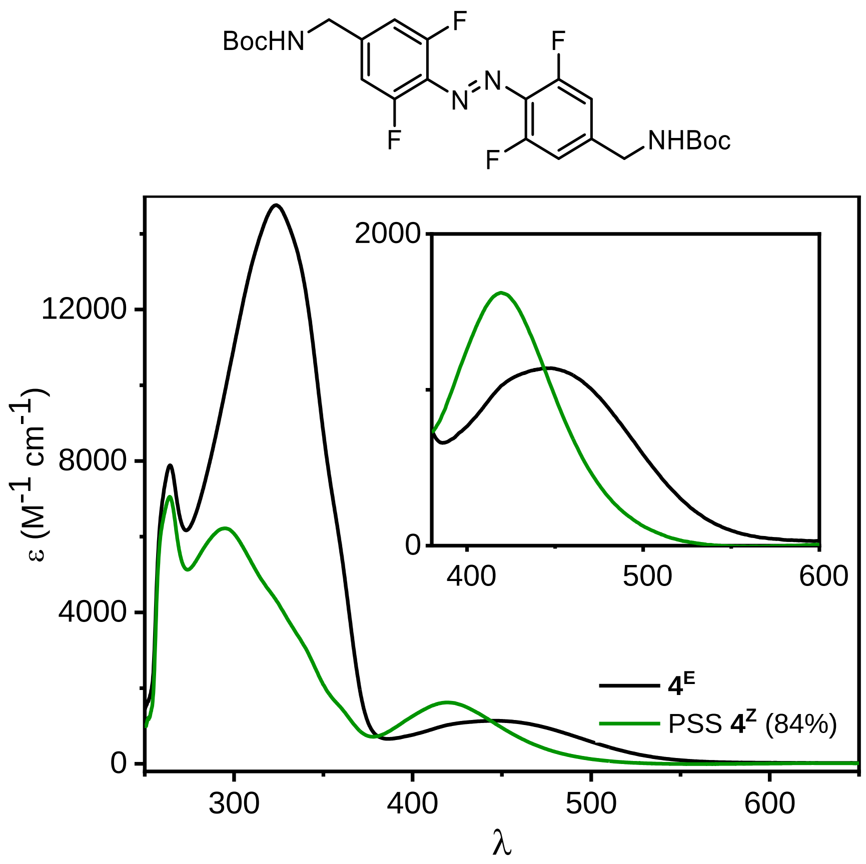

Figure S237. UV-vis Spectrum of **F_4-NHBoc** in the dark (100% *E*) and green (84% *Z*) state.

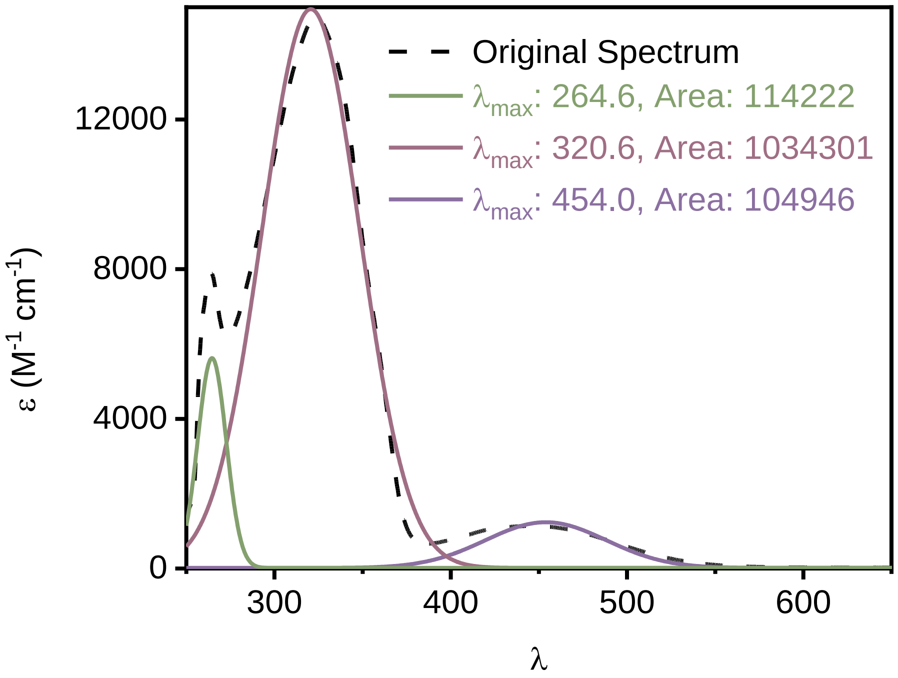

Figure S238. Deconvoluted UV-vis Spectrum of (*E*)-**F_4-NHBoc**

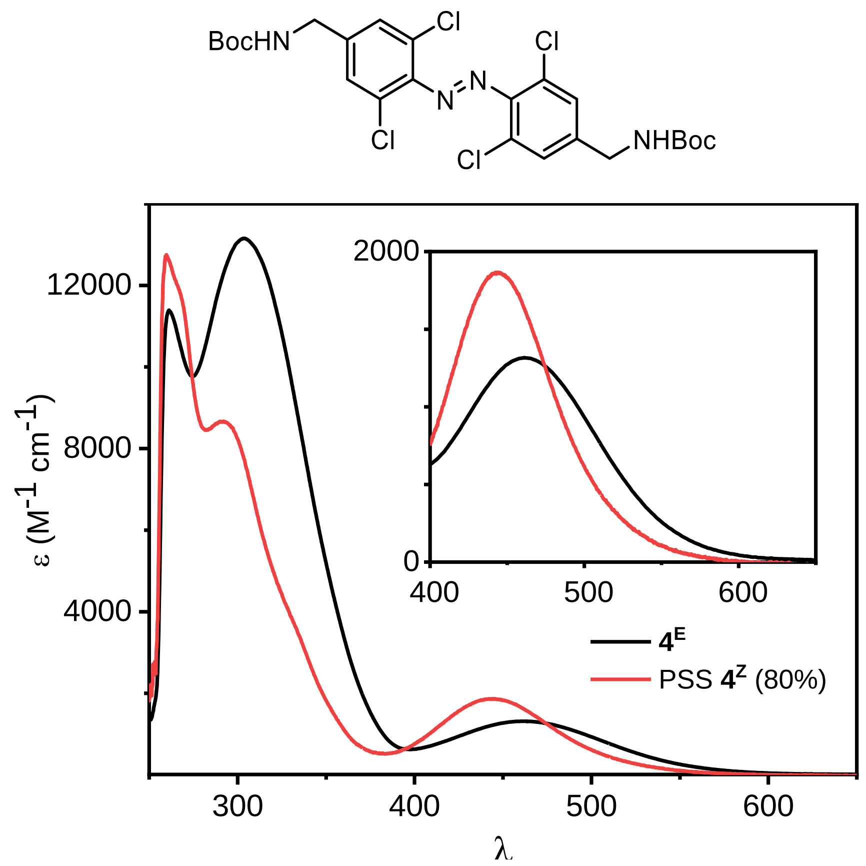

Figure S239. UV-vis Spectrum of **Cl$_4$-NHBoc** in the dark (100% *E*) and red (77% *Z*) state.

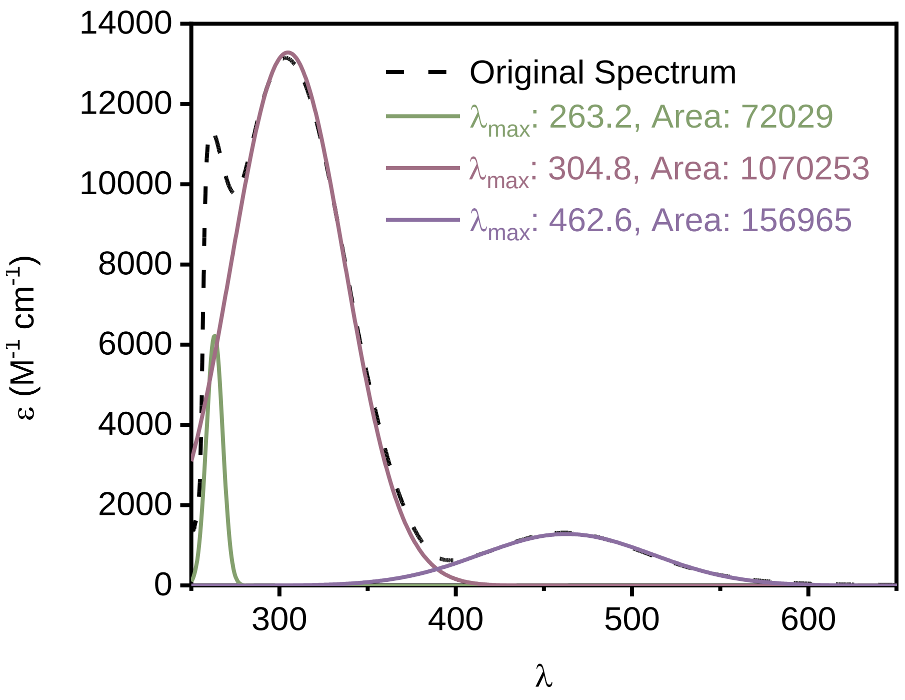

Figure S240. Deconvoluted UV-vis Spectrum of (*E*)-**Cl$_4$-NHBoc**

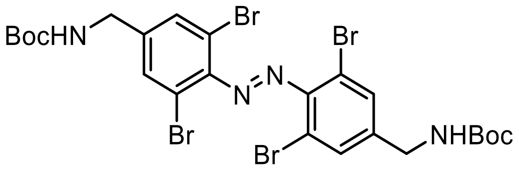

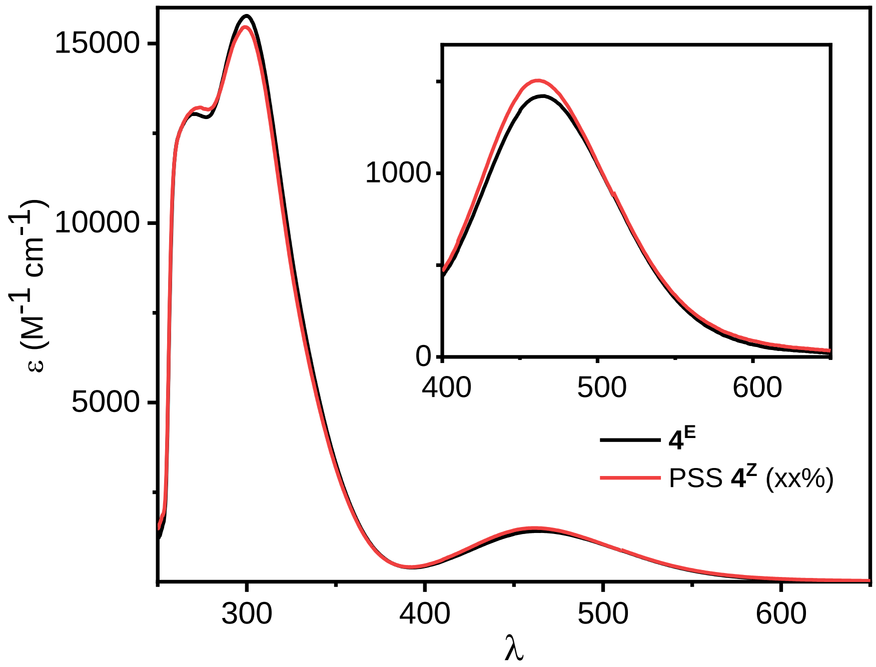

Figure S241. UV-vis Spectrum of **Br$_4$-NHBoc** in the dark (100% *E*) and red (63% Z) state

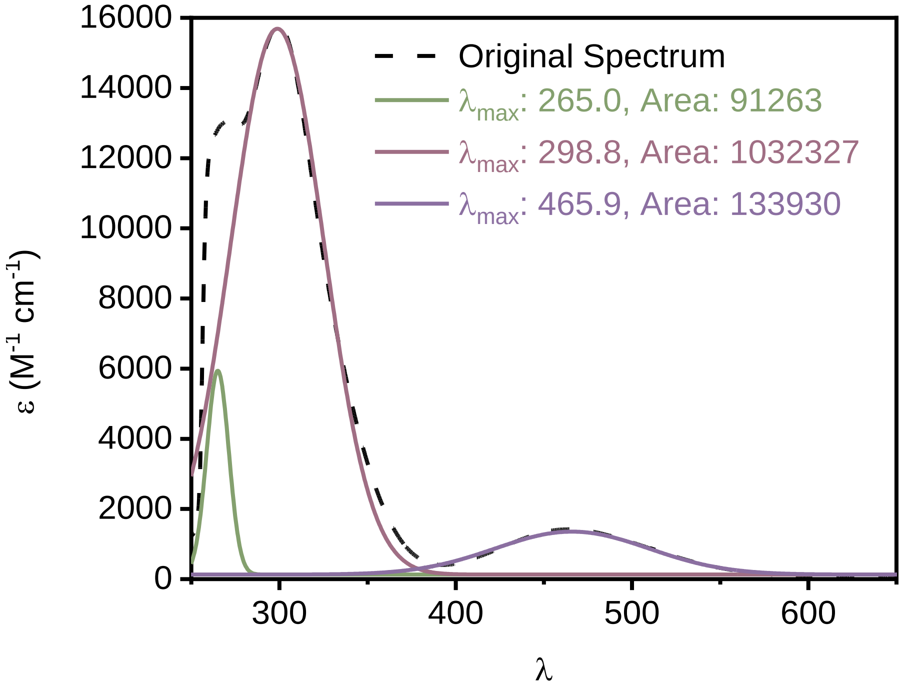

Figure S242. Deconvoluted UV-vis Spectrum of (*E*)-**Br$_4$-NHBoc**

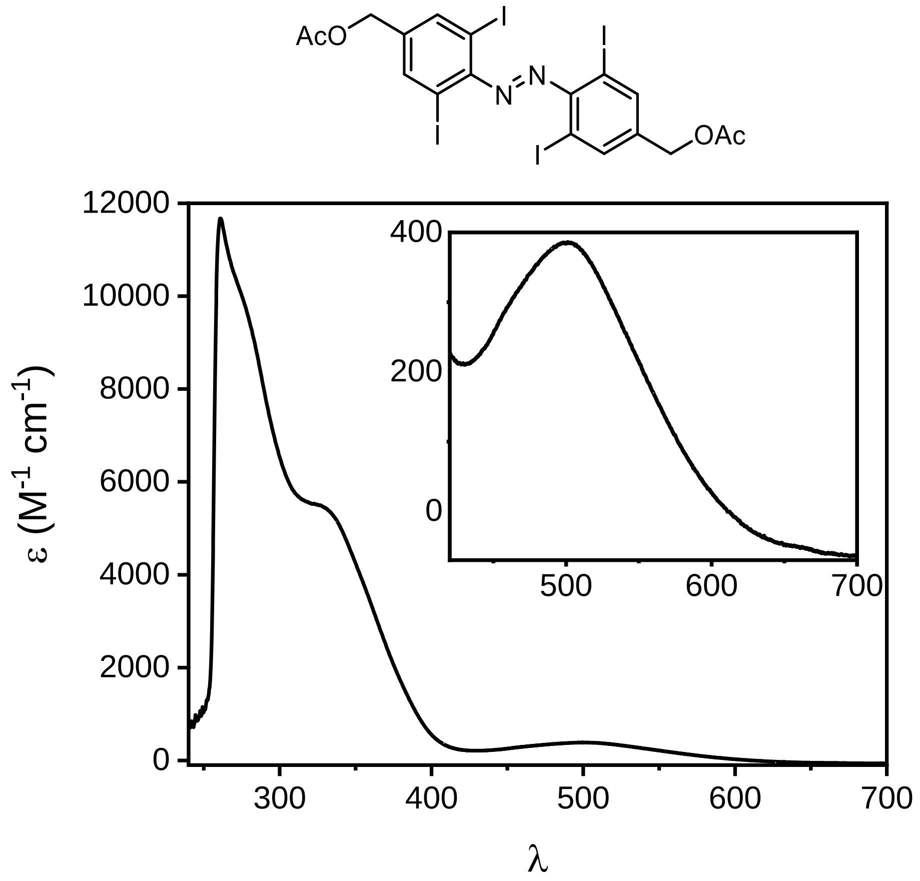

Figure S243. UV-vis Spectrum of **I_4-OAc** in the dark (100% *E*)

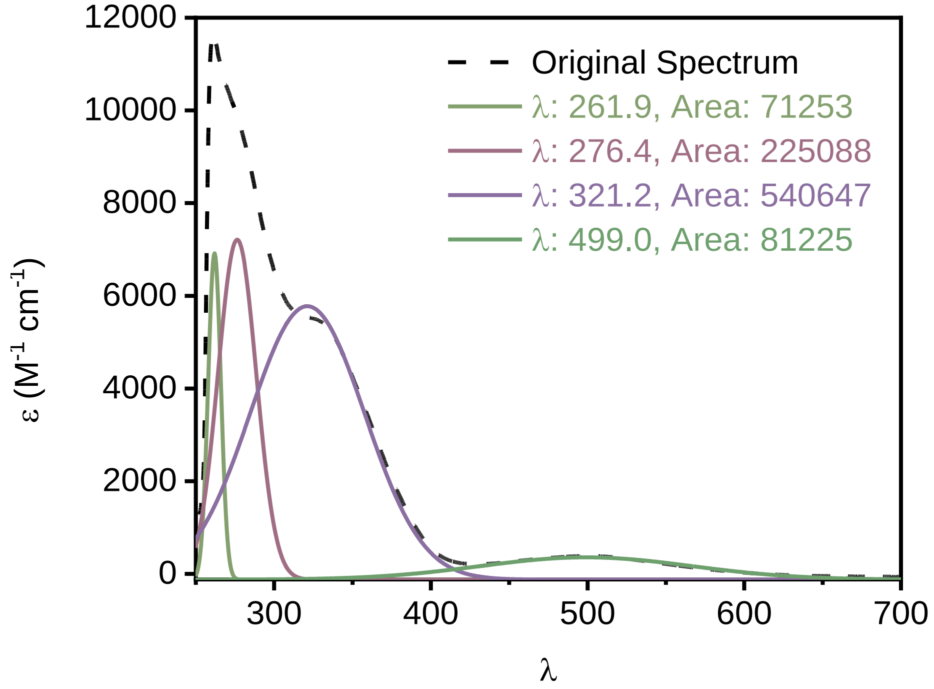

Figure S244. Deconvoluted UV-vis Spectrum of (*E*)-**I_4-OAc**

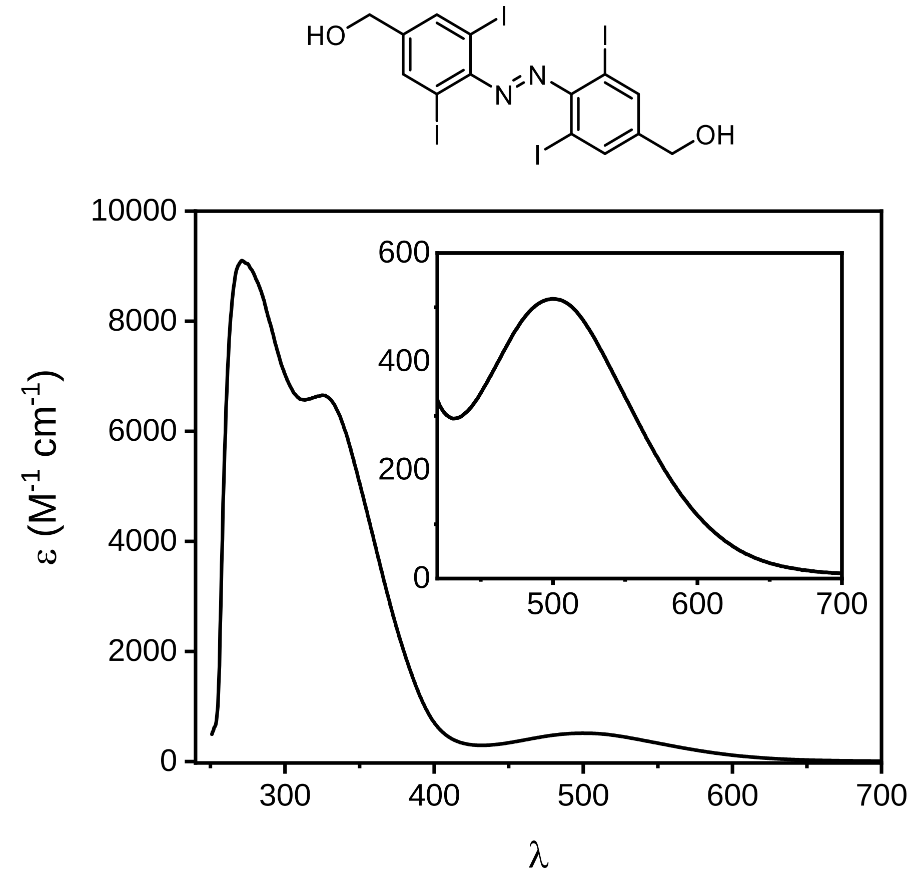

Figure S245. UV-vis Spectrum of **I_4-OH** in the dark (100% *E*)

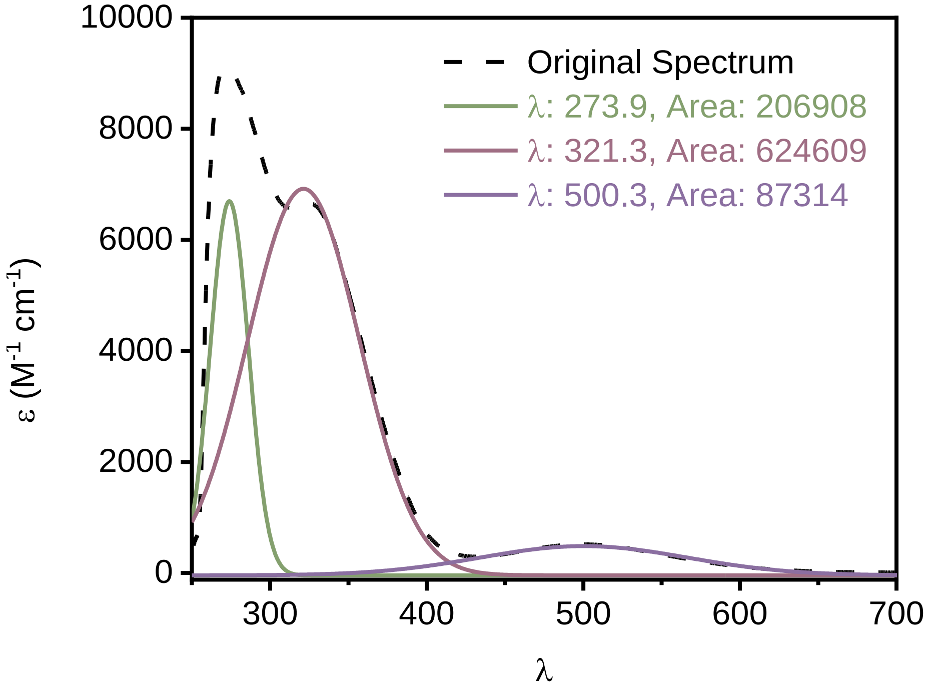

Figure S246. Deconvoluted UV-vis Spectrum of (*E*)-**I_4-OH**

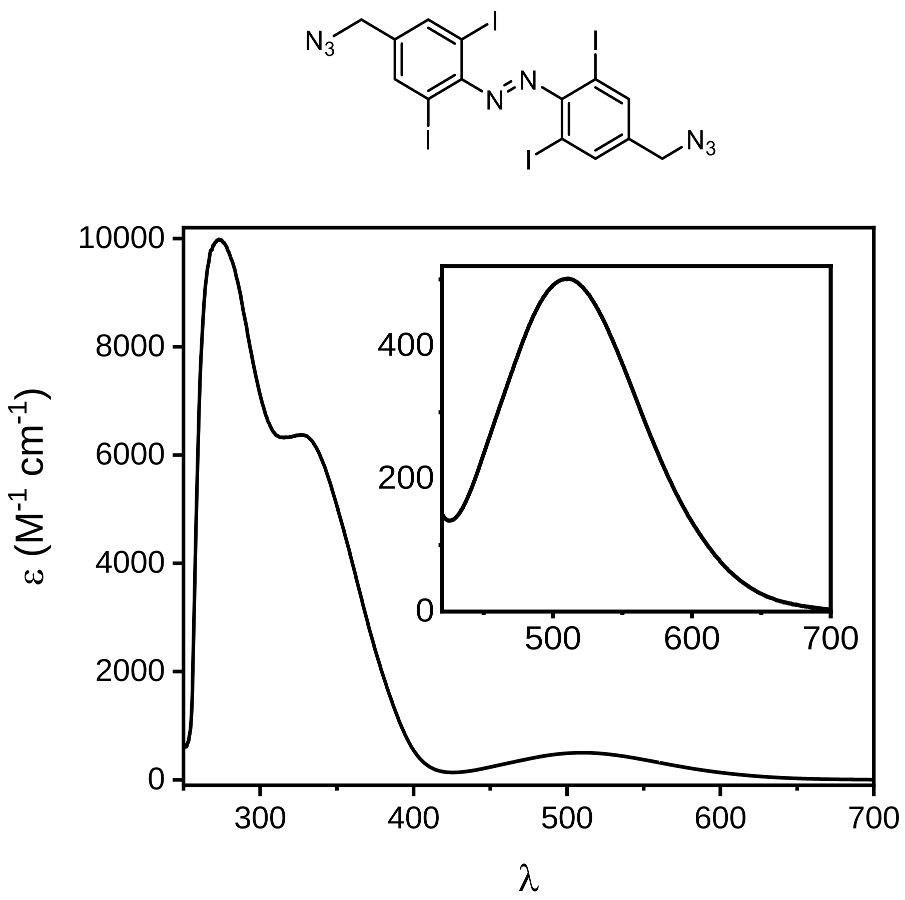

Figure S247. UV-vis Spectrum of **I_4-N_3** in the dark (100% *E*)

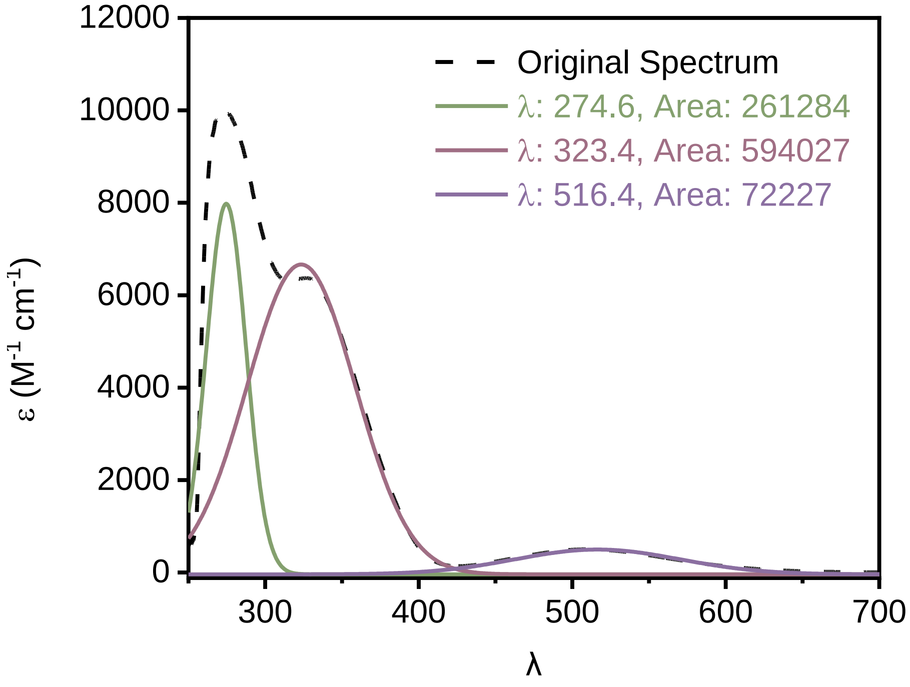

Figure S248. Deconvoluted UV-vis Spectrum of (*E*)-**I_4-N_3**

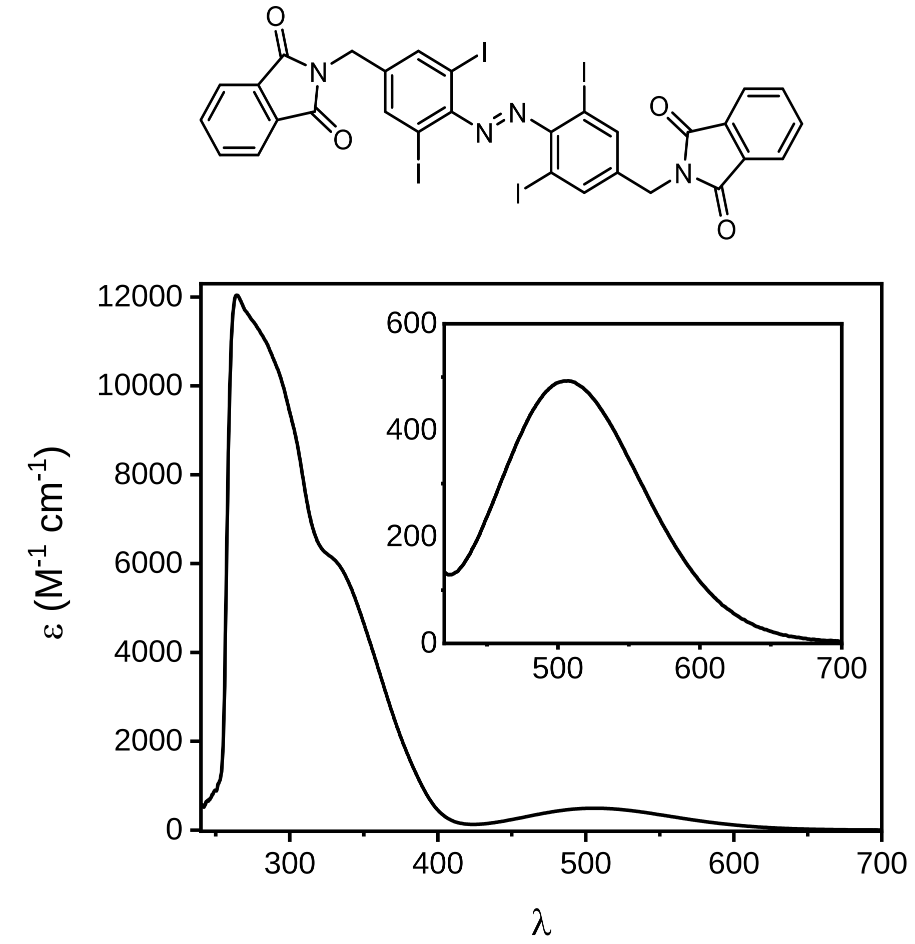

Figure S249. UV-vis Spectrum of **I_4-Phth** in the dark (100% *E*)

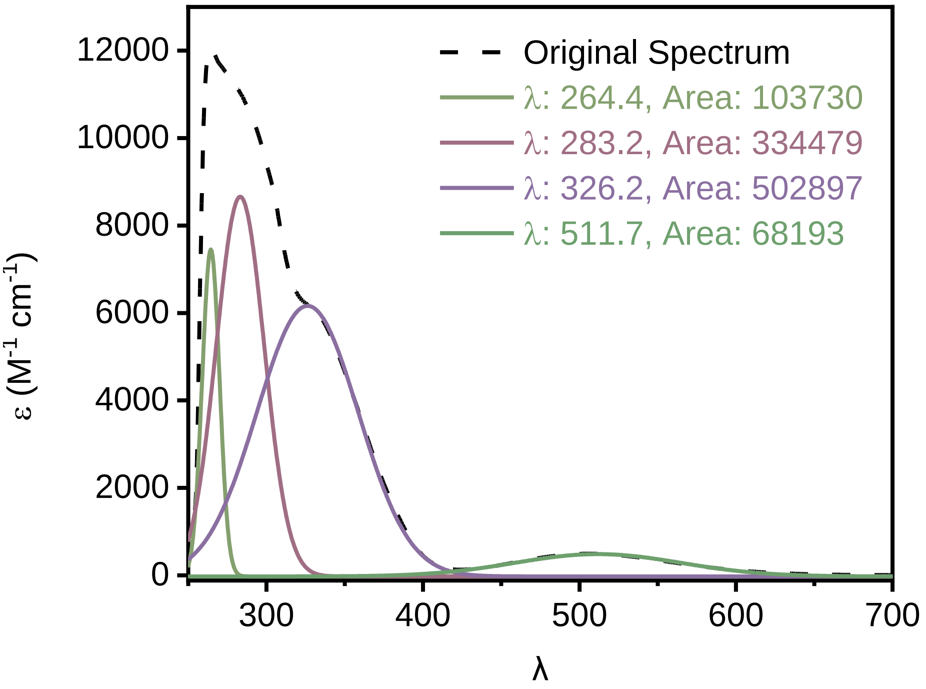

Figure S250. Deconvoluted UV-vis Spectrum of (*E*)-**I_4-Phth**

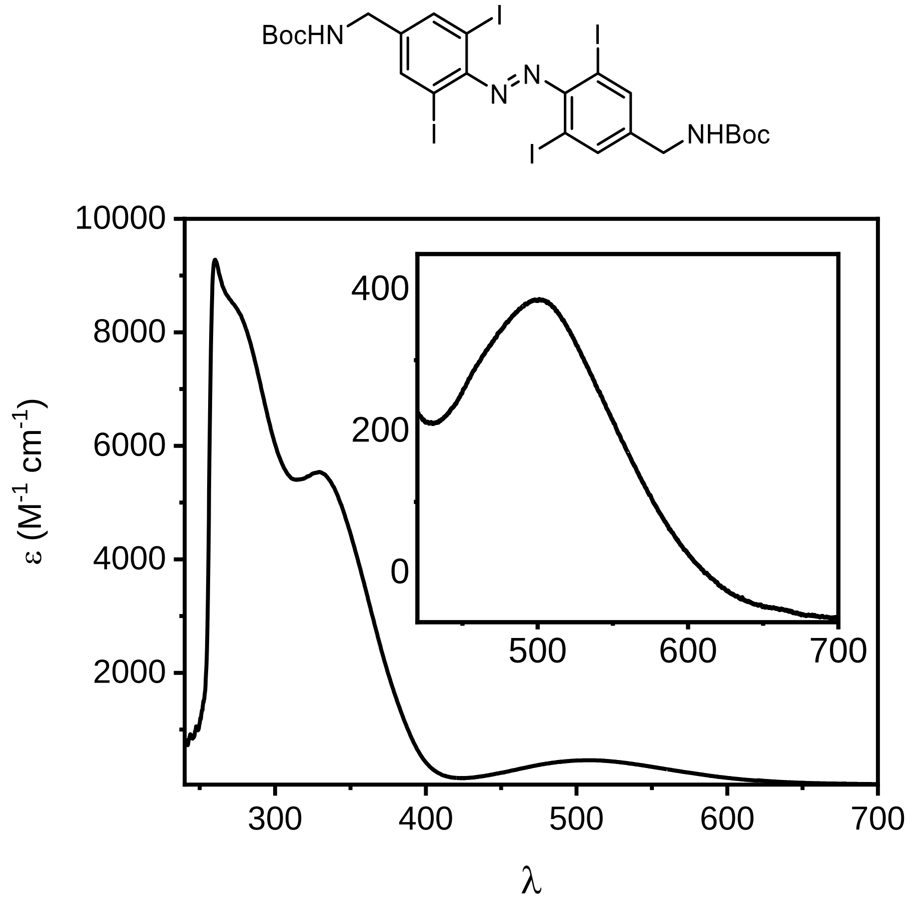

Figure S251. UV-vis Spectrum of **I$_4$-NHBoc** in the dark (100% *E*)

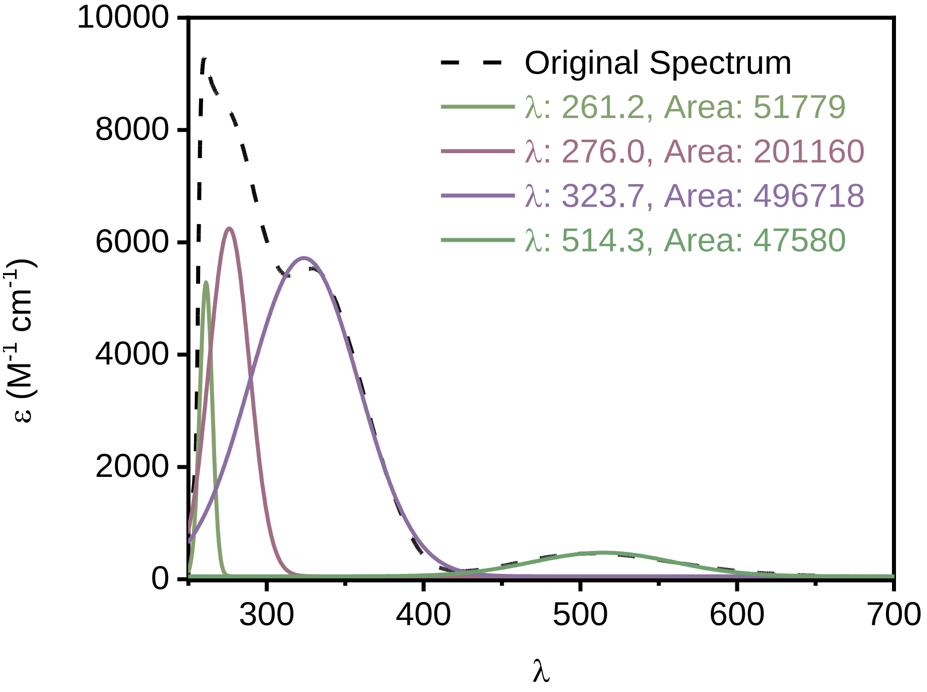

Figure S252. Deconvoluted UV-vis Spectrum of (*E*)-**I$_4$-NHBoc**

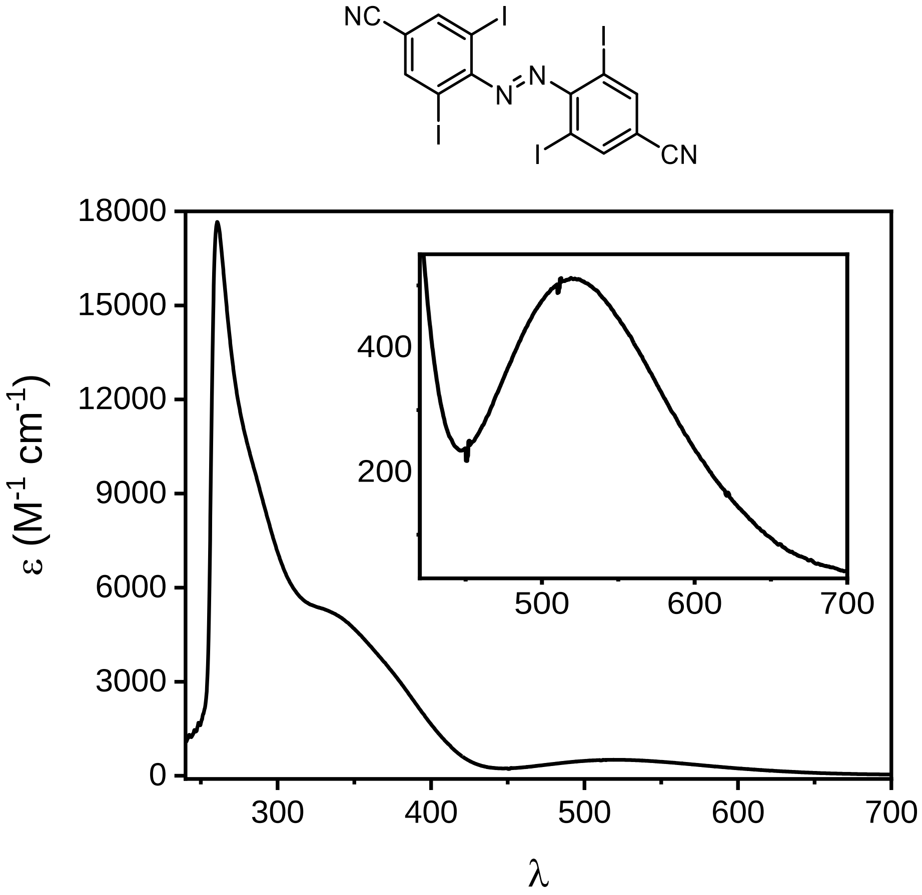

Figure S253. UV-vis Spectrum of **I$_4$-CN** in the dark (100% *E*)

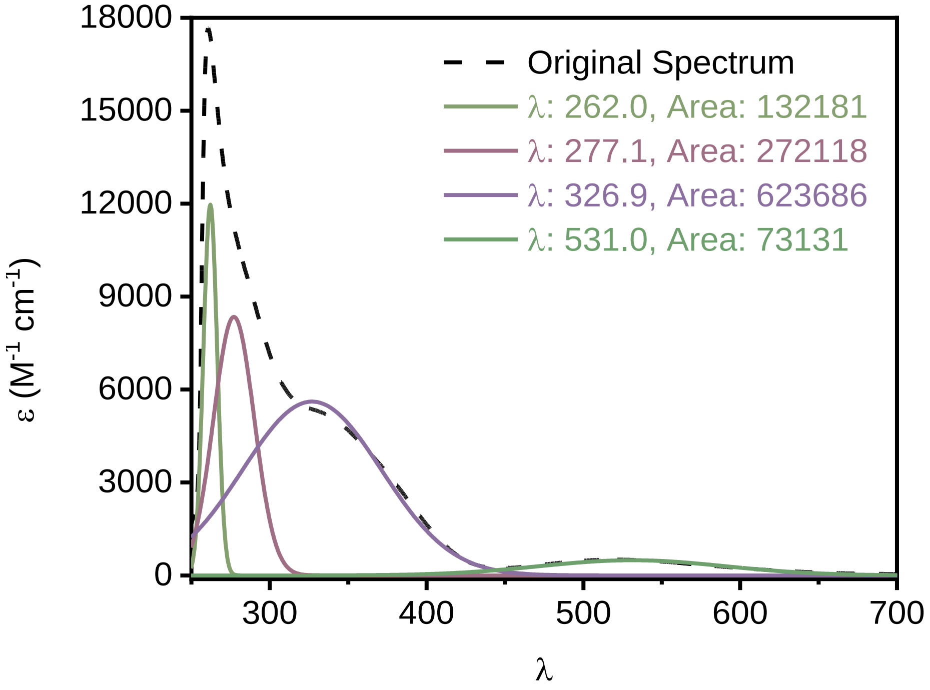

Figure S254. Deconvoluted UV-vis Spectrum of (*E*)-**I$_4$-CN**

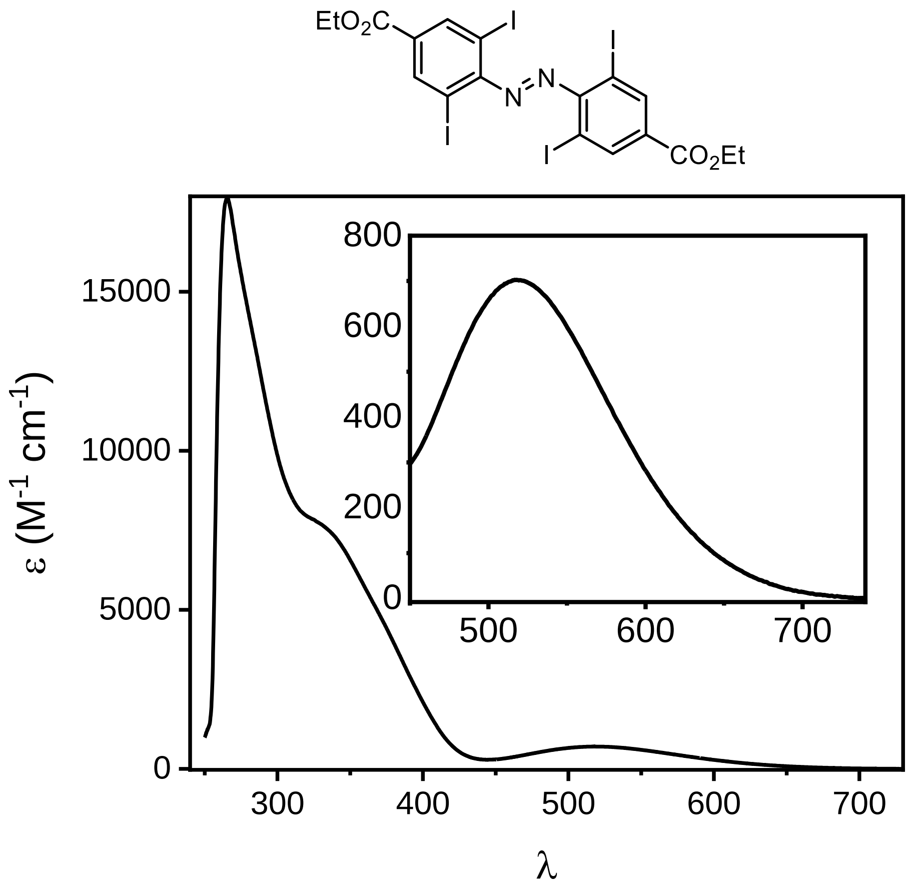

Figure S255. UV-vis Spectrum of **I$_4$-ester** in the dark (100% *E*)

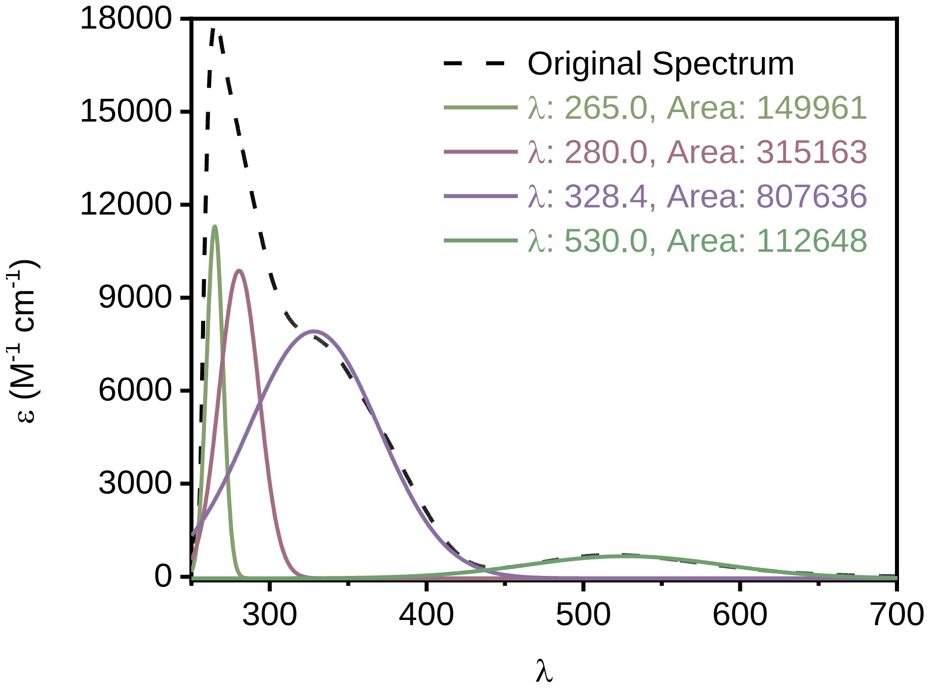

Figure S256. Deconvoluted UV-vis Spectrum of (*E*)-**I$_4$-ester**

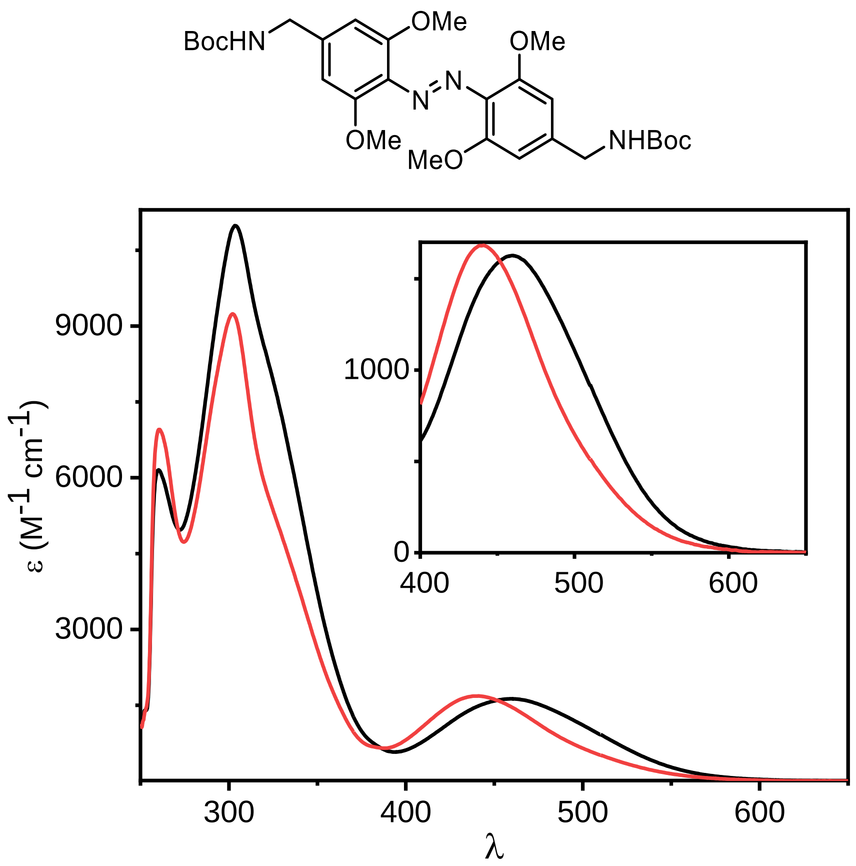

Figure S257. UV-vis Spectrum of **(OMe)$_4$-NHBoc** in the dark (100% *E*) and red (95% *Z*) state.

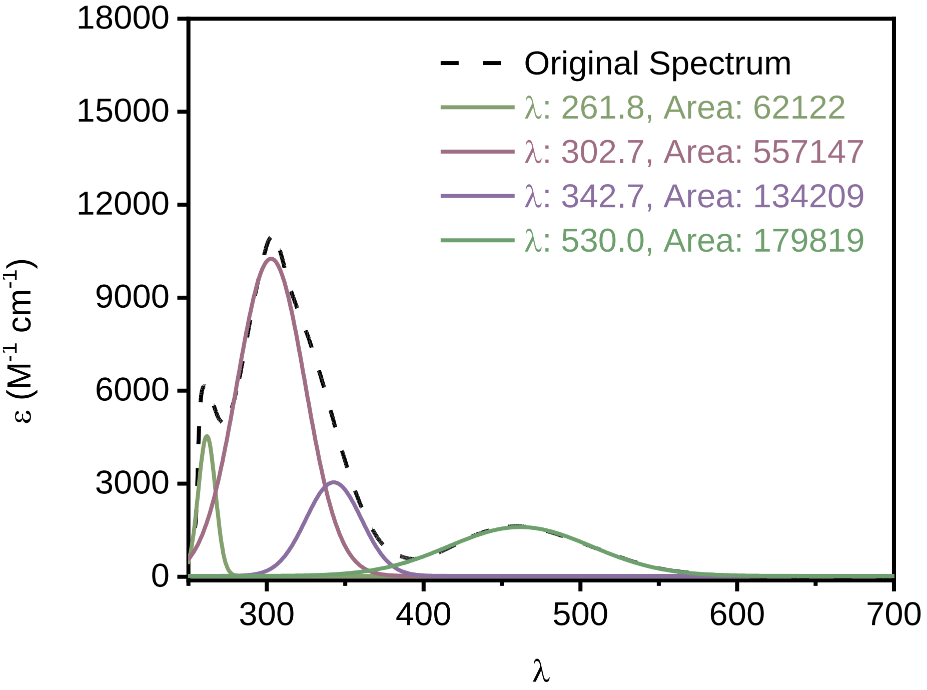

Figure S258. Deconvoluted UV-vis Spectrum of (*E*)-**(OMe)$_4$-NHBoc**

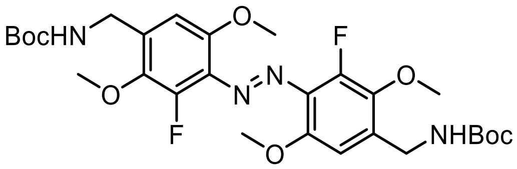

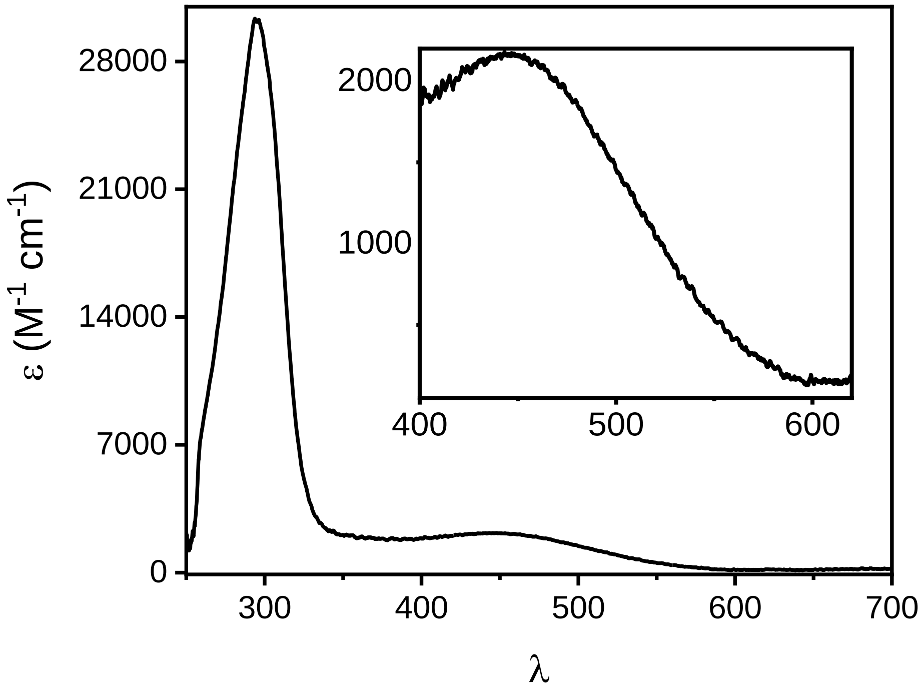

Figure S259. UV-vis Spectrum of **(OMe)$_2$F$_2$,m-OMe$_2$-NHBoc** in the dark (100% *E*)

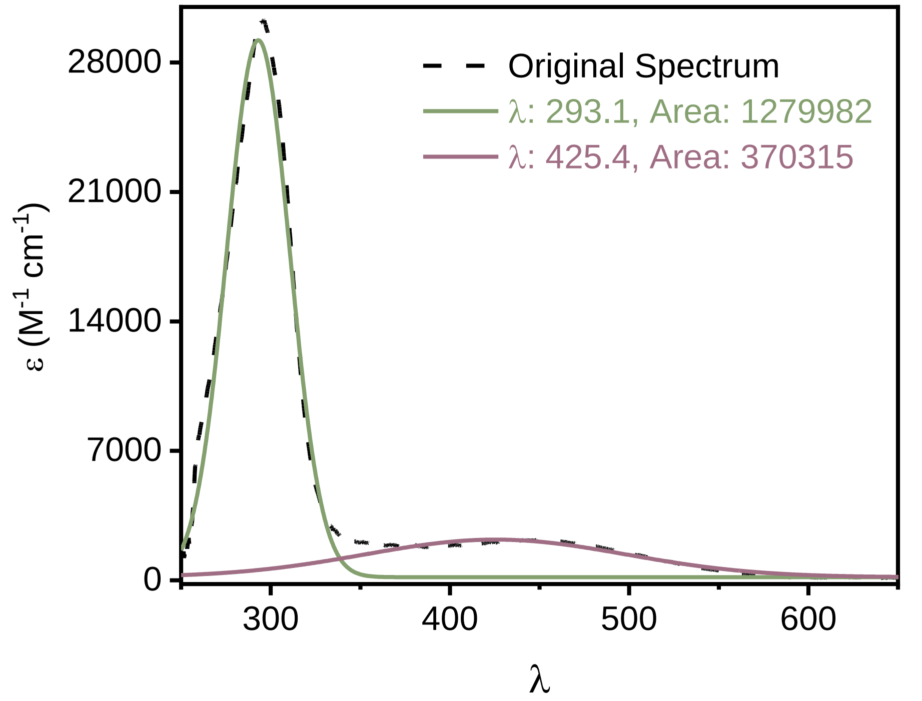

Figure S260. Deconvoluted UV-vis Spectrum of (*E*)-**(OMe)$_2$F$_2$,m-OMe$_2$-NHBoc**

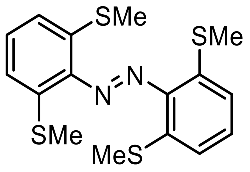

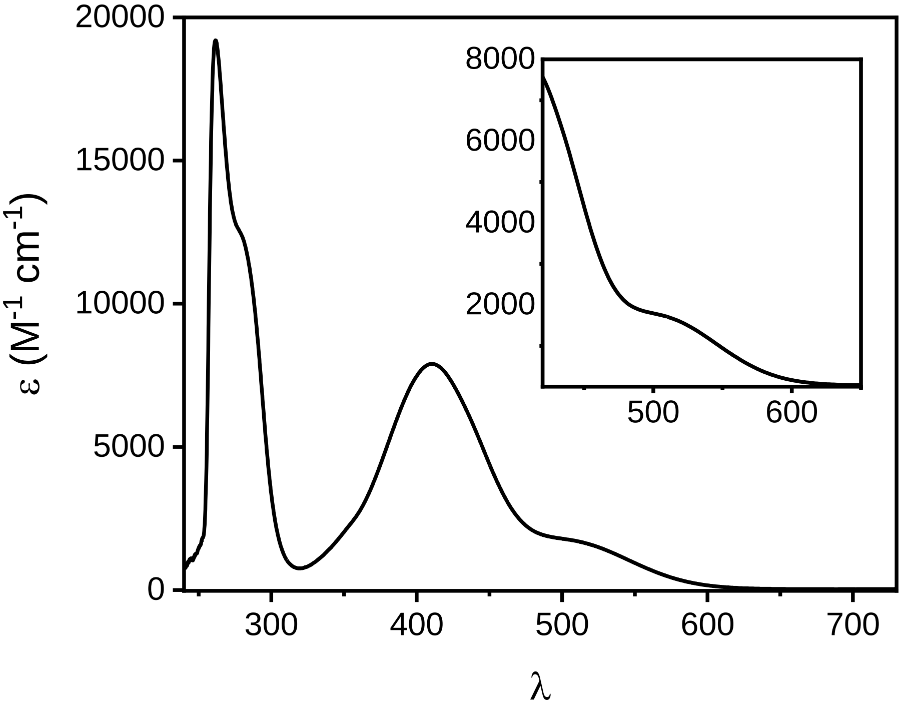

Figure S261. UV-vis Spectrum of **(SMe)$_4$-H** in the dark (100% *E*)

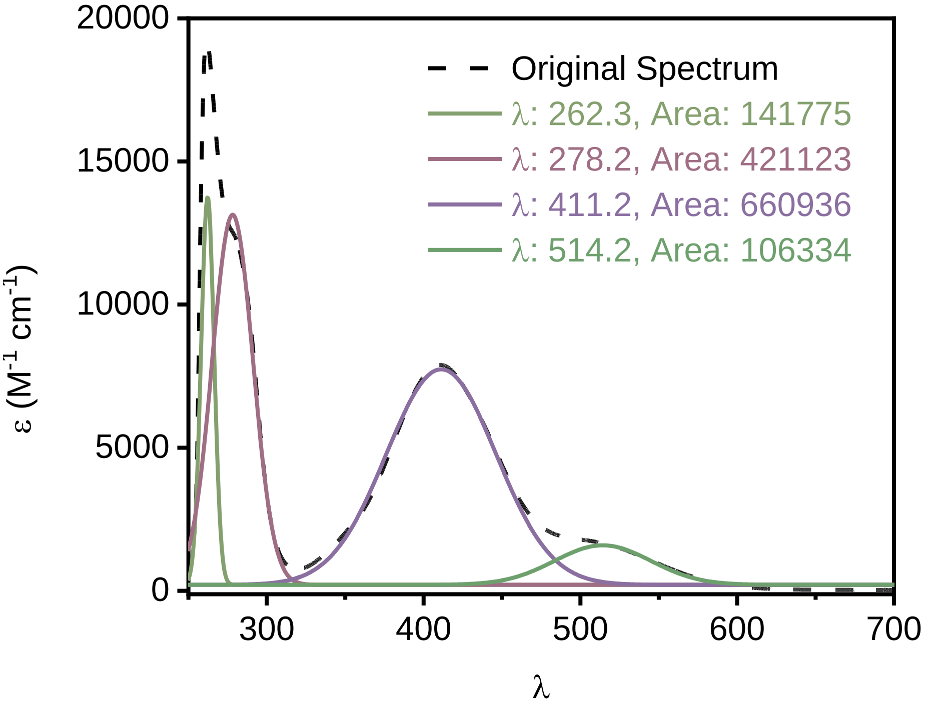

Figure S262. Deconvoluted UV-vis Spectrum of (*E*)-**(SMe)$_4$-H**

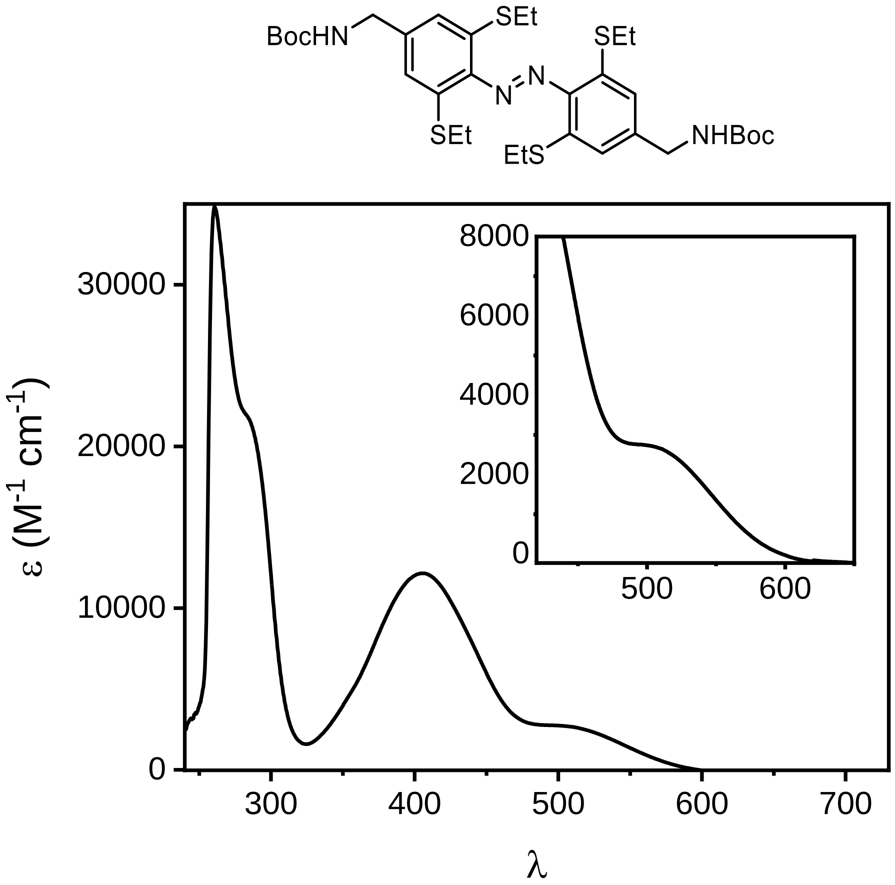

Figure S263. UV-vis Spectrum of **(SEt)$_4$-NHBoc** in the dark (100% *E*)

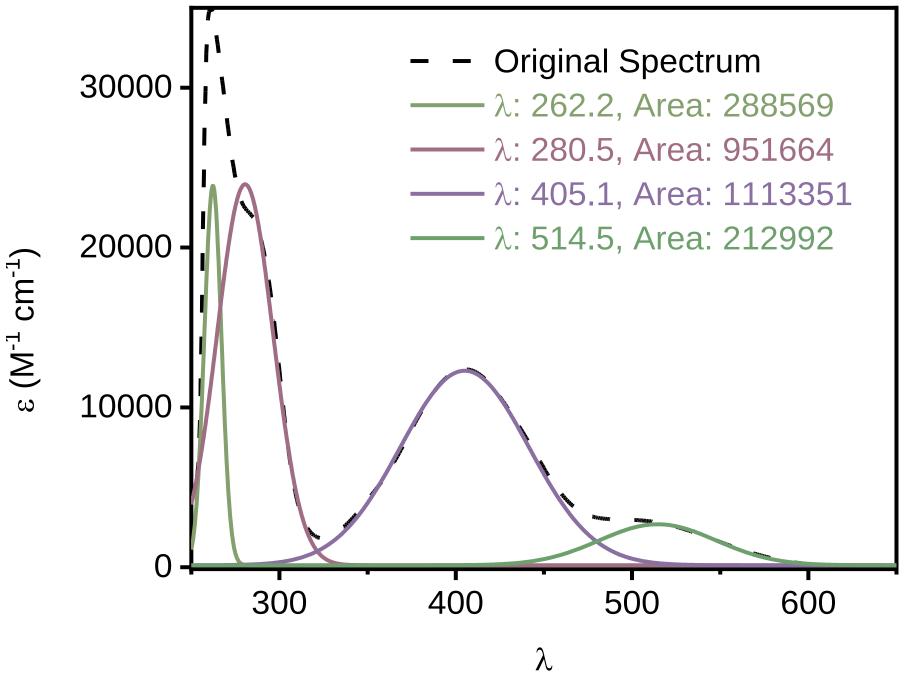

Figure S264. Deconvoluted UV-vis Spectrum of (*E*)-**(SEt)$_4$-NHBoc**

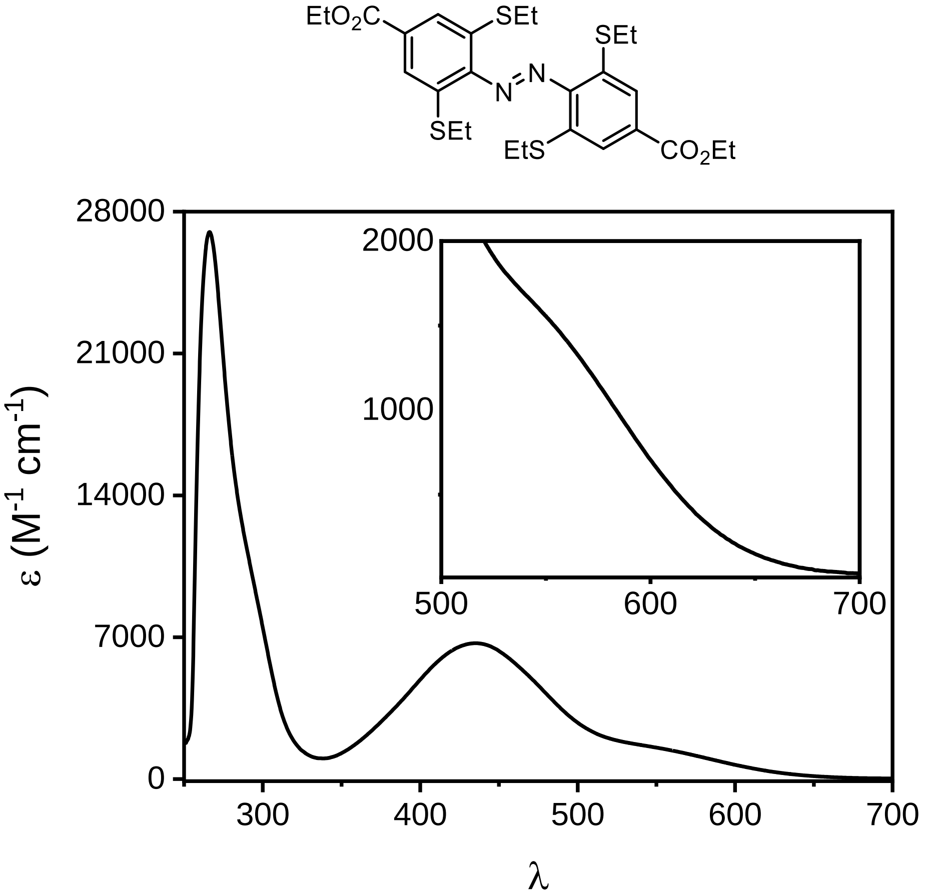

Figure S265. UV-vis Spectrum of **(SEt)$_4$-ester** in the dark (100% *E*)

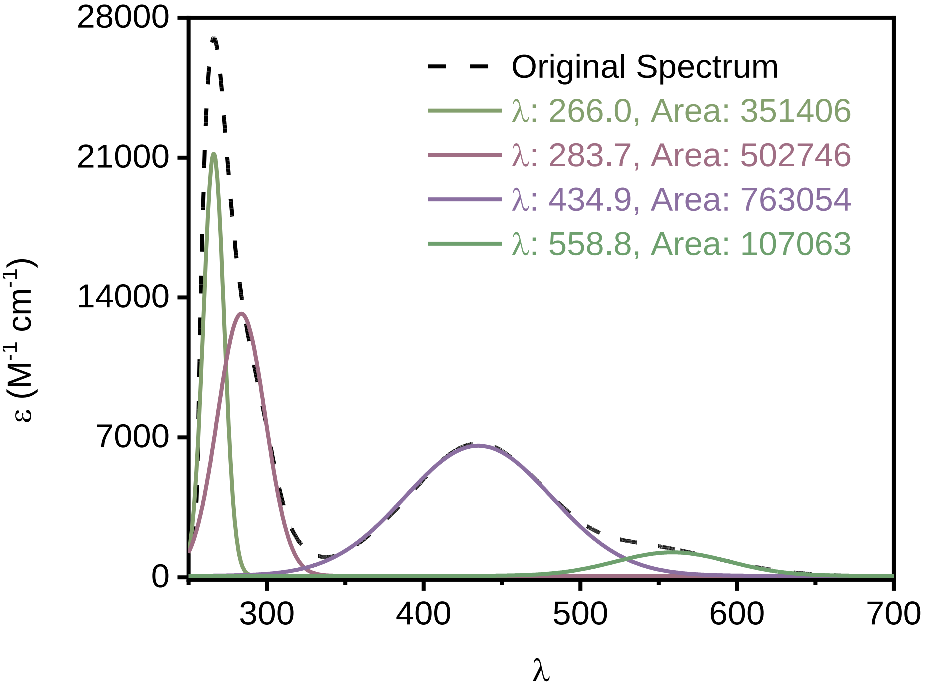

Figure S266. Deconvoluted UV-vis Spectrum of (*E*)-**(SEt)$_4$-ester**

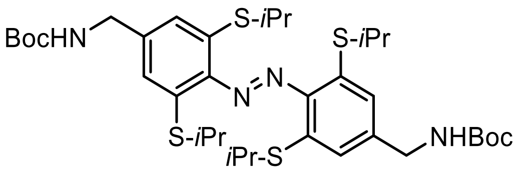

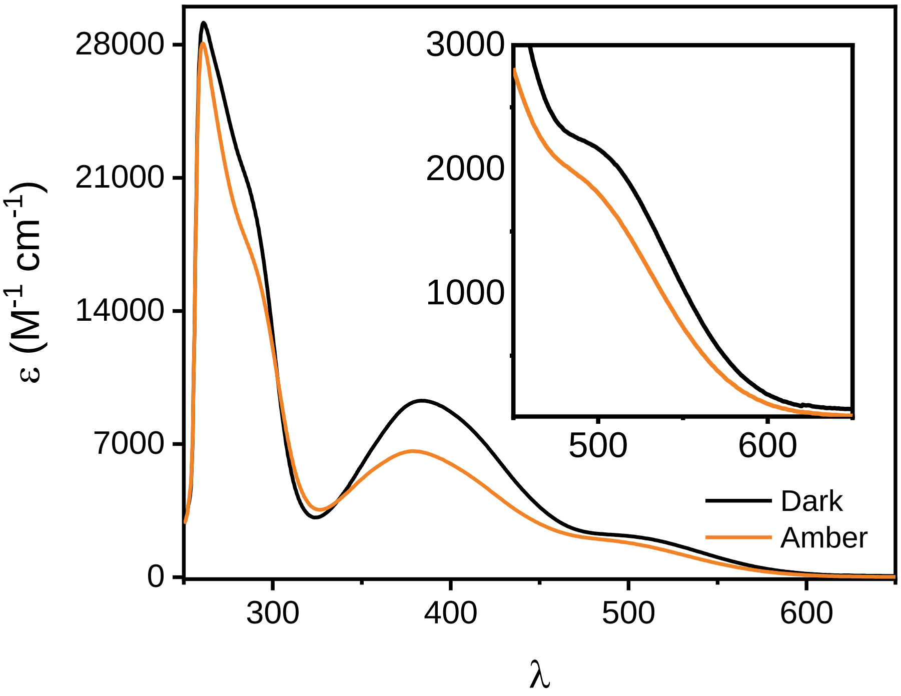

Figure S267. UV-vis Spectrum of **(S^{i}Pr)$_4$-NHBoc** in the dark (100% *E*) and under Amber irradiation

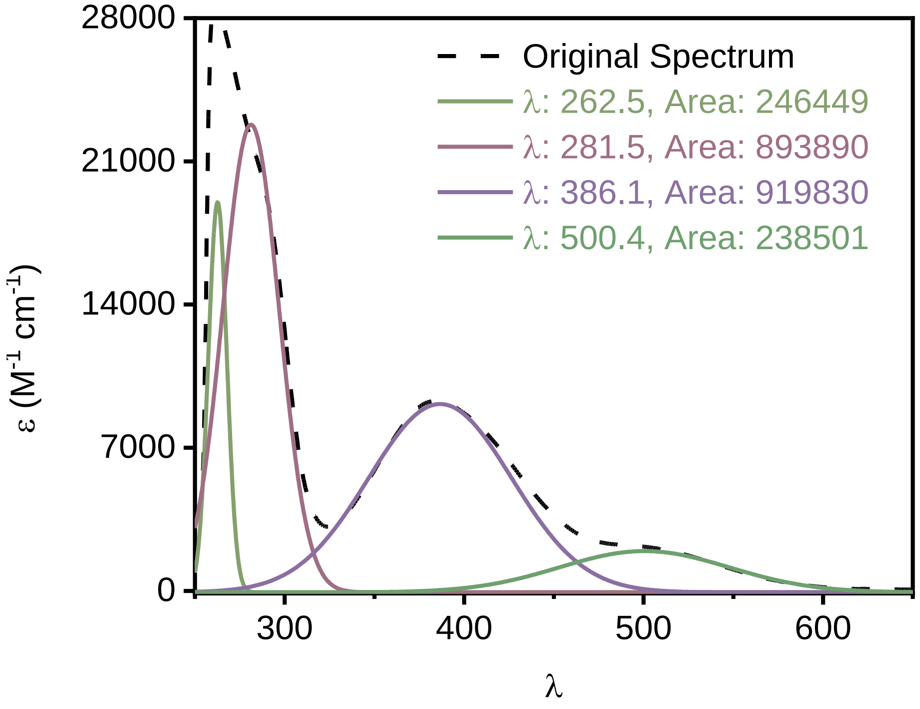

Figure S268. Deconvoluted UV-vis Spectrum of (*E*)-**(S^{i}Pr)$_4$-NHBoc**

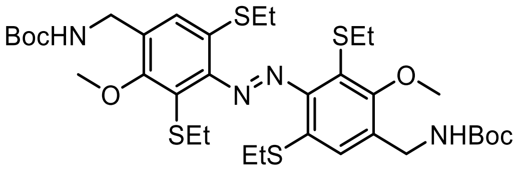

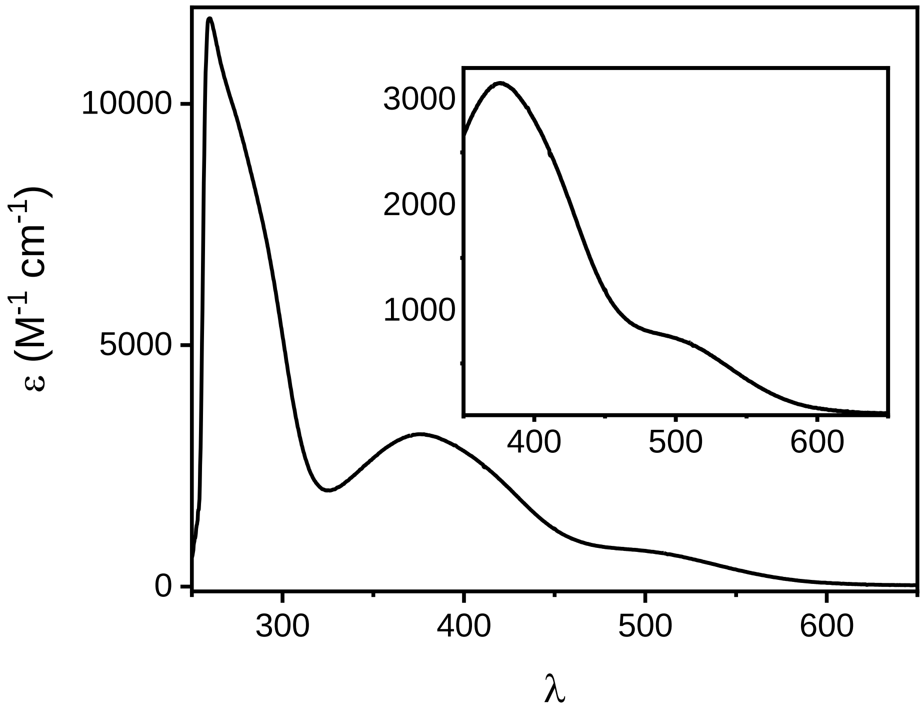

Figure S269. UV-vis Spectrum of **(SEt)$_4$,*m*-OMe$_2$-NHBoc** in the dark (100% *E*)

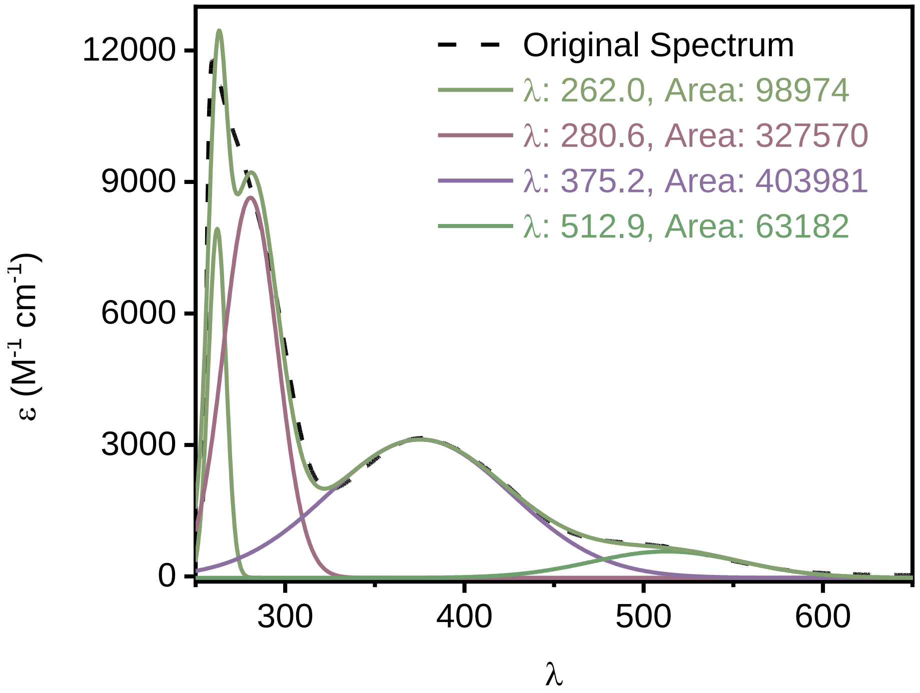

Figure S270. Deconvoluted UV-vis Spectrum of (*E*)-**(SEt)$_4$,*m*-OMe$_2$-NHBoc**

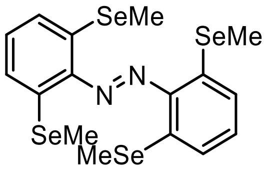

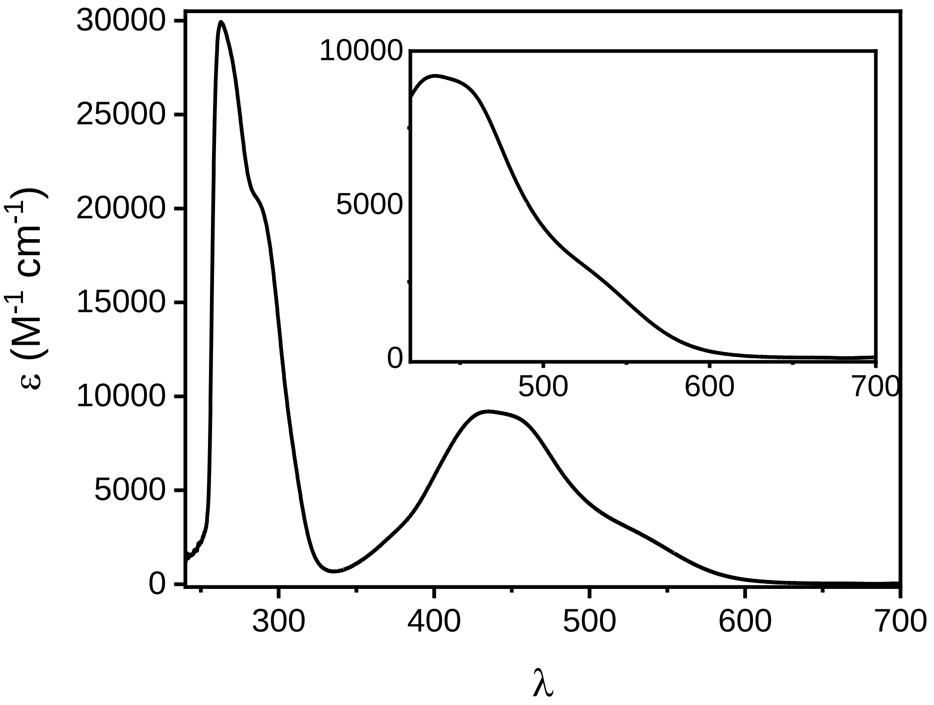

Figure S271. UV-vis Spectrum of **(SeMe)$_4$-H** in the dark (100% *E*)

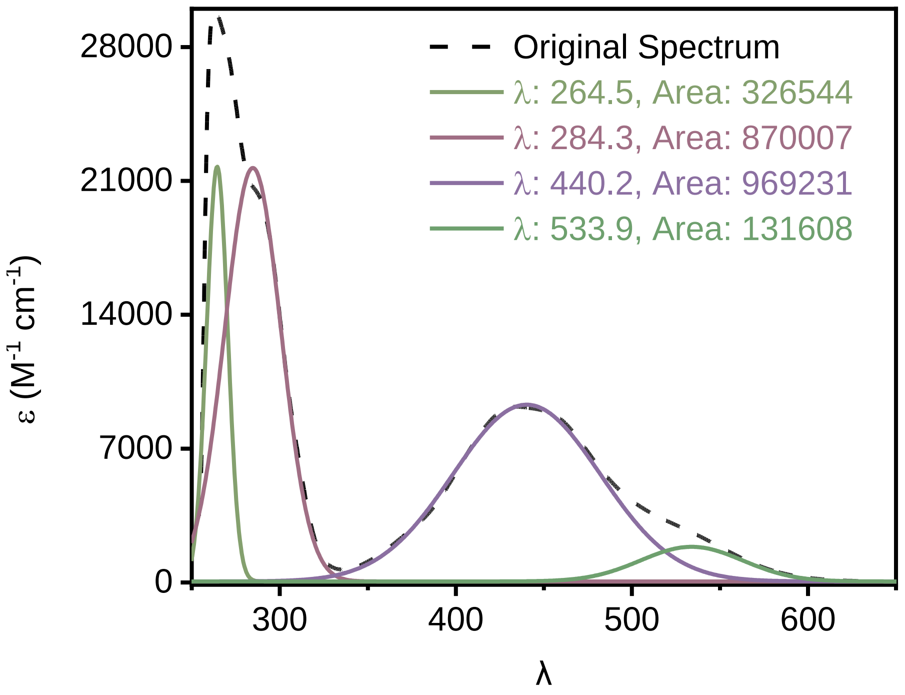

Figure S272. Deconvoluted UV-vis Spectrum of (*E*)-**(SeMe)$_4$-H**

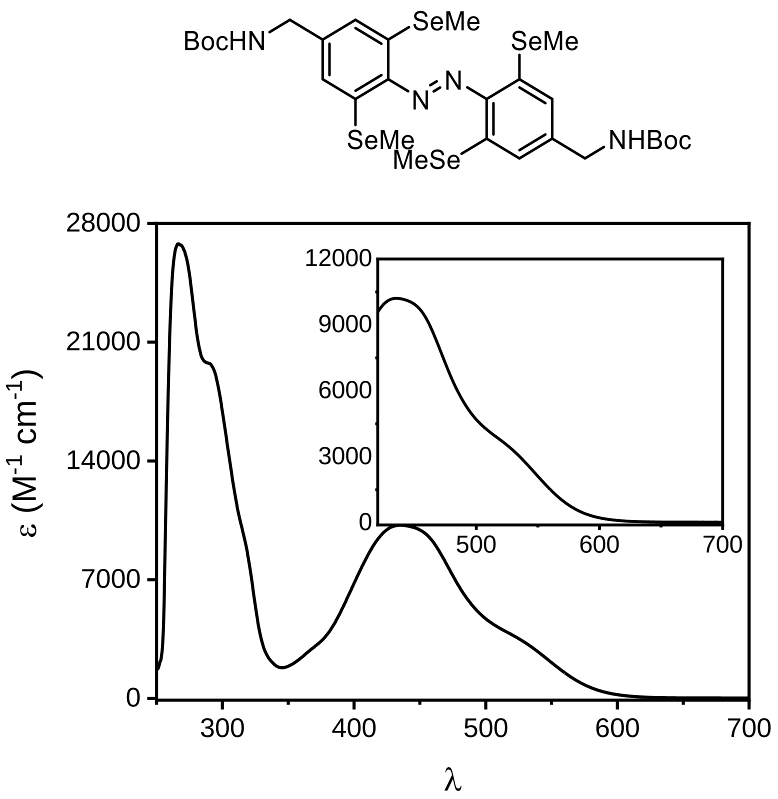

Figure S273. UV-vis Spectrum of **(SeMe)$_4$-NHBoc** in the dark (100% *E*)

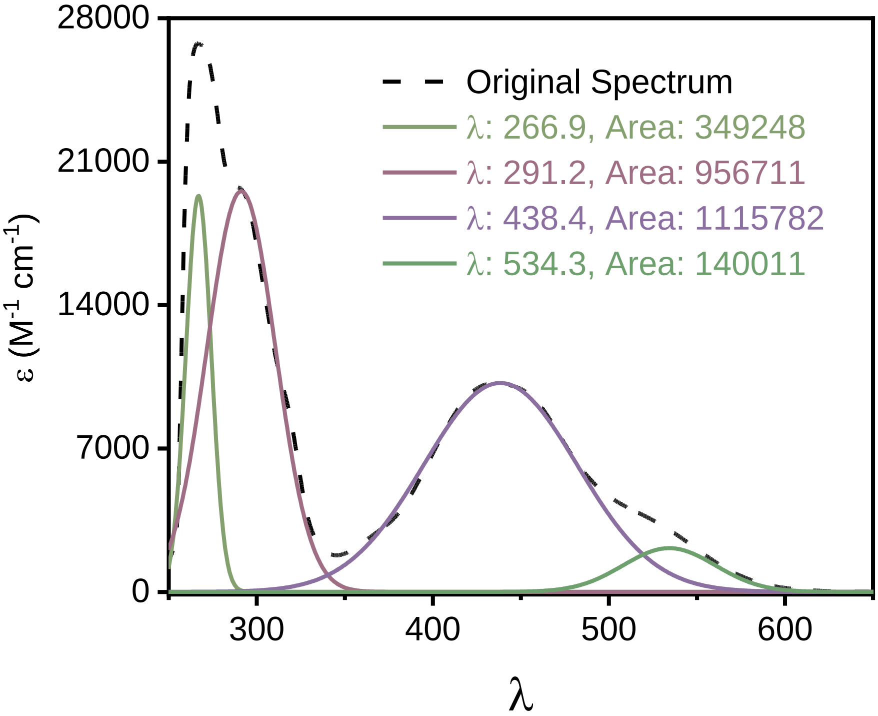

Figure S274. Deconvoluted UV-vis Spectrum of (*E*)-**(SeMe)$_4$-NHBoc**

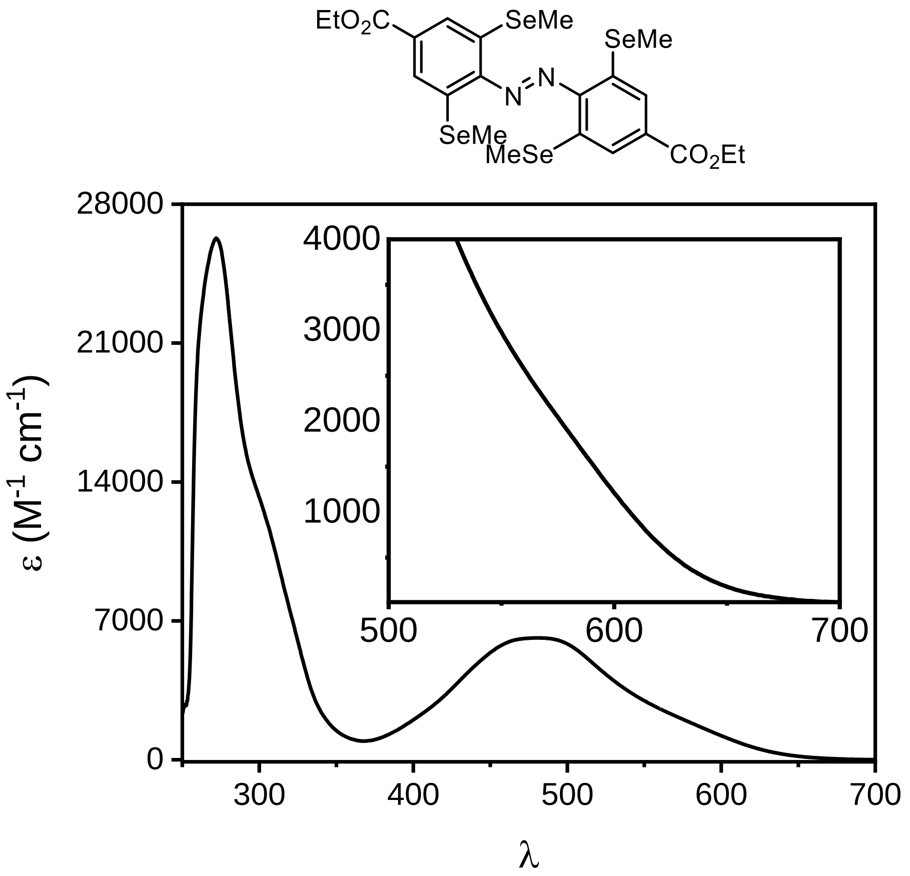

Figure S275. UV-vis Spectrum of **(SeMe)$_4$-ester** in the dark (100% *E*)

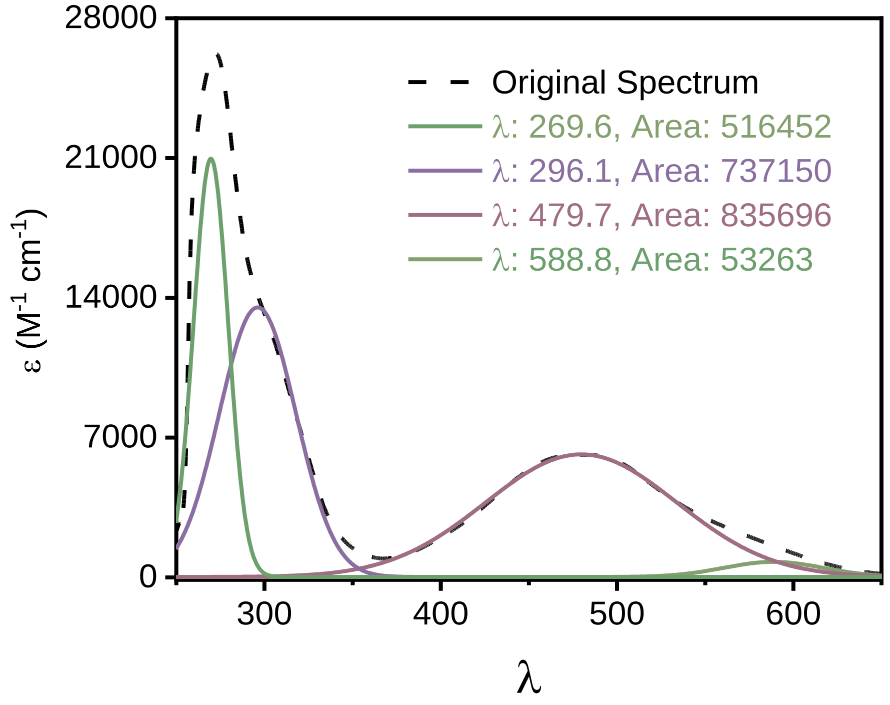

Figure S276. Deconvoluted UV-vis Spectrum of (*E*)-**(SeMe)$_4$-ester**

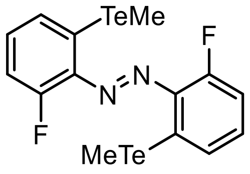

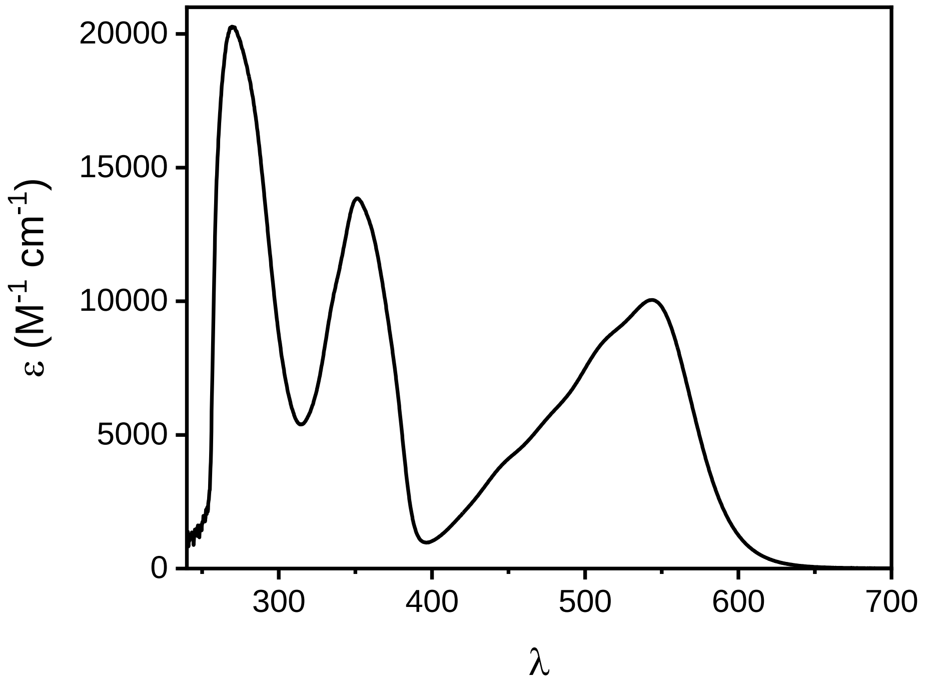

Figure S277. UV-vis Spectrum of $\mathbf{Te_2F_2}$**-H** in the dark (100% *E*)

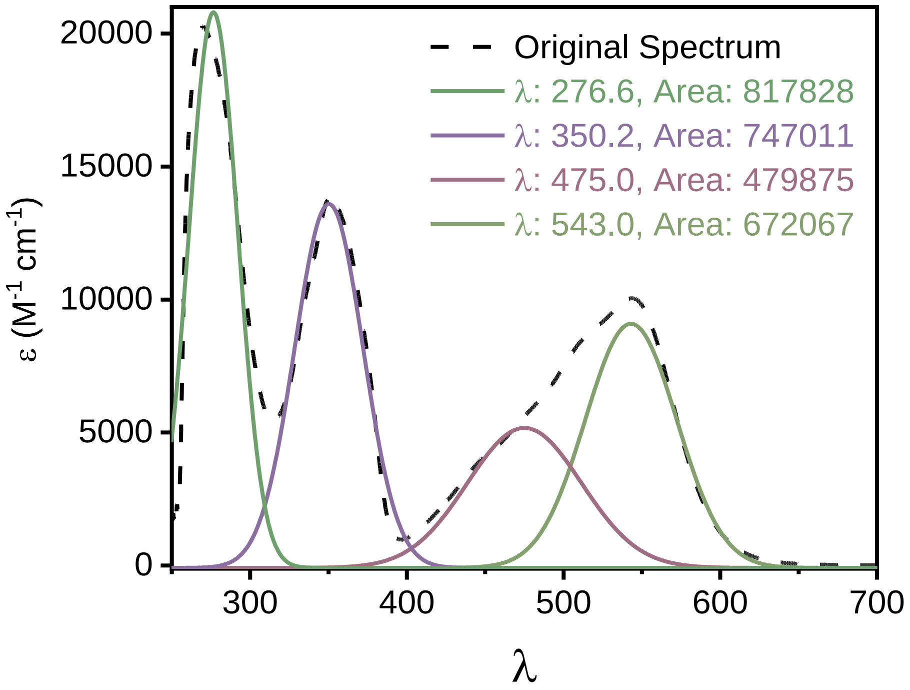

Figure S278. Deconvoluted UV-vis Spectrum of (*E*)-$\mathbf{Te_2F_2}$**-H**

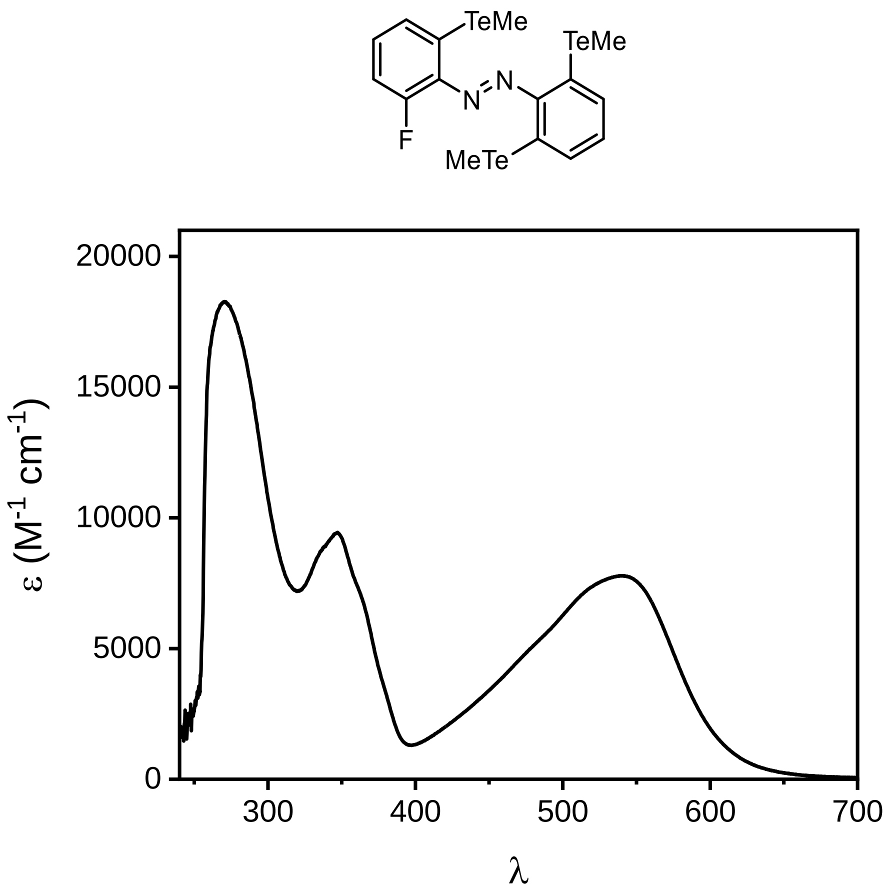

Figure S279. UV-vis Spectrum of **Te_3F-H** in the dark (100% *E*)

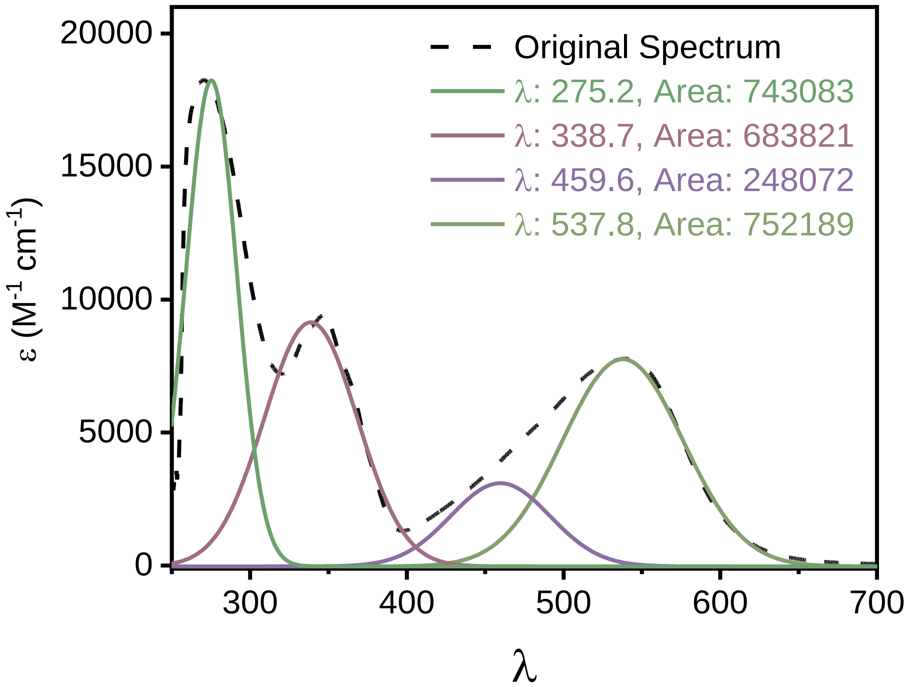

Figure S280. Deconvoluted UV-vis Spectrum of (*E*)-**Te_3F-H**

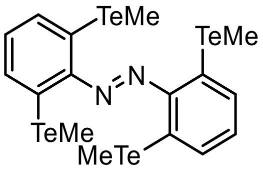

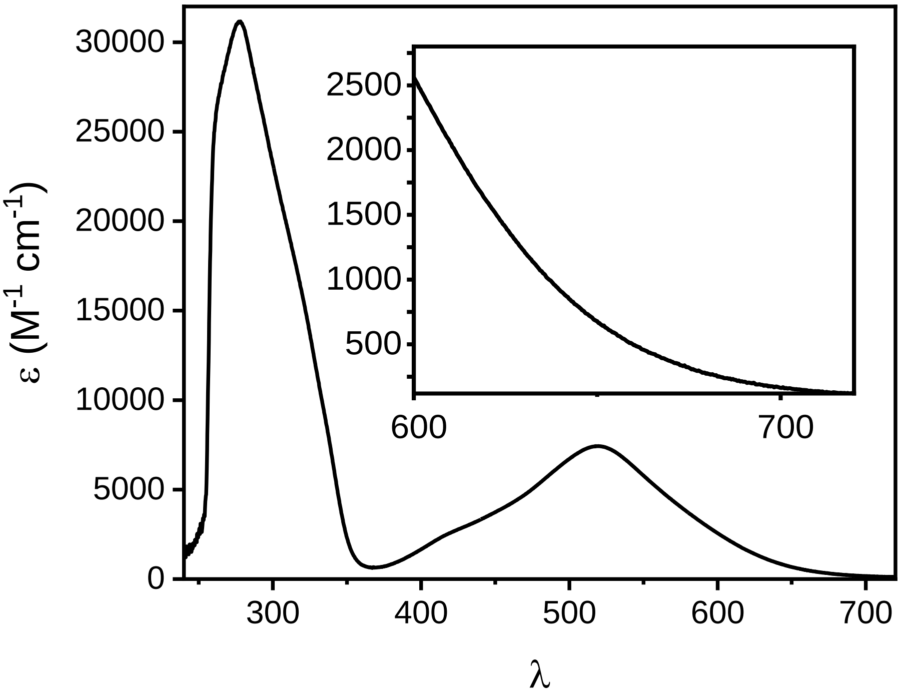

Figure S281. UV-vis Spectrum of **(TeMe)$_4$-H** in the dark (100% *E*)

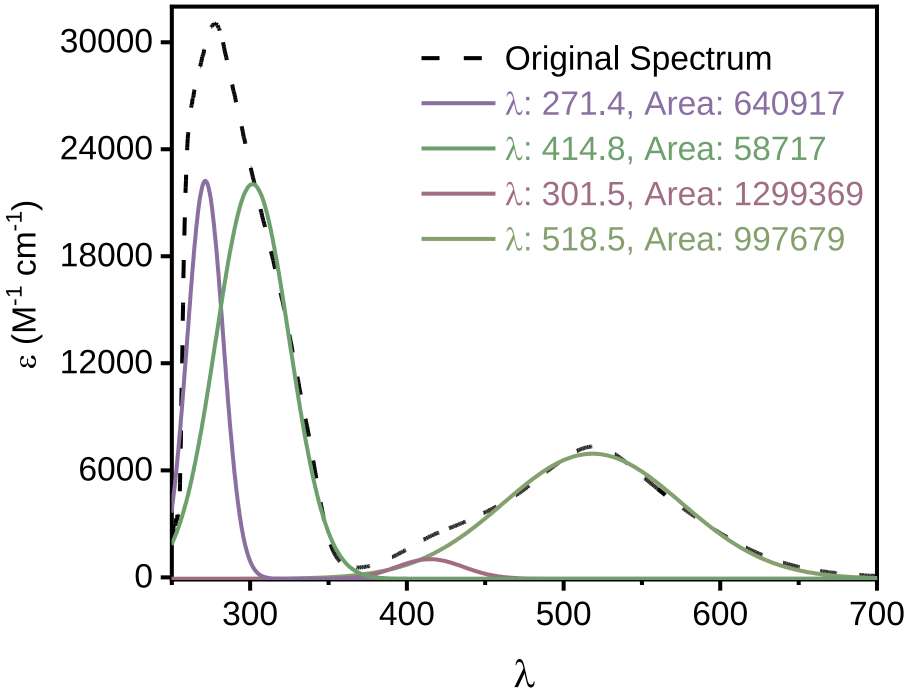

Figure S282. Deconvoluted UV-vis Spectrum of (*E*)-**(TeMe)$_4$-H**

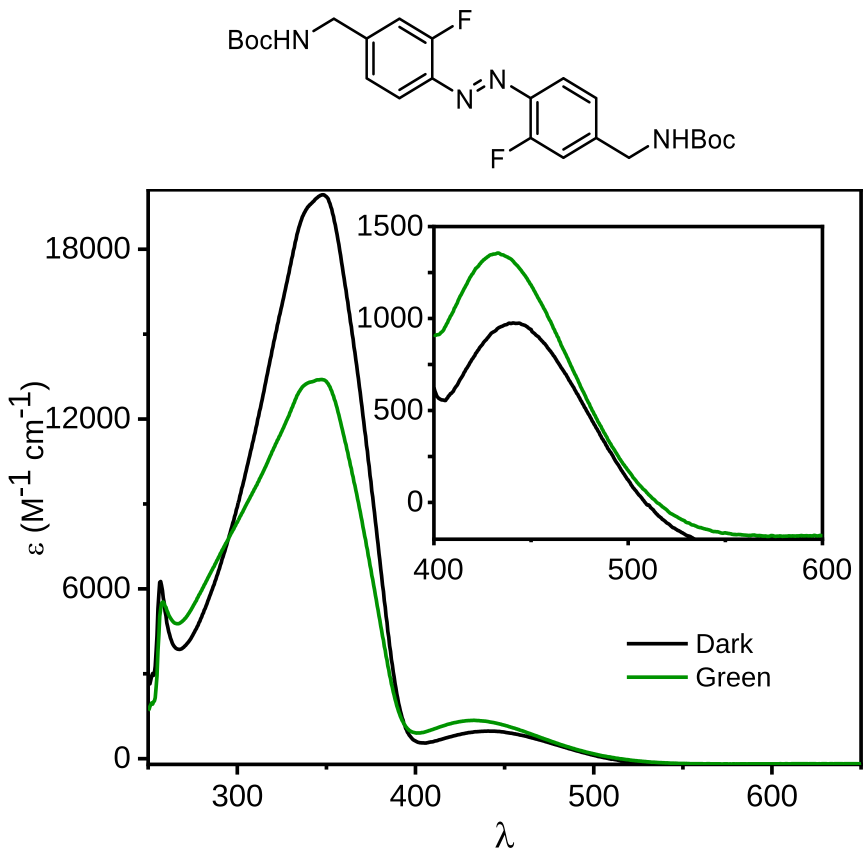

Figure S283. UV-vis Spectrum of **F_2,*m*-H_2** in the dark (100% *E*) and green (50% *Z*) state.

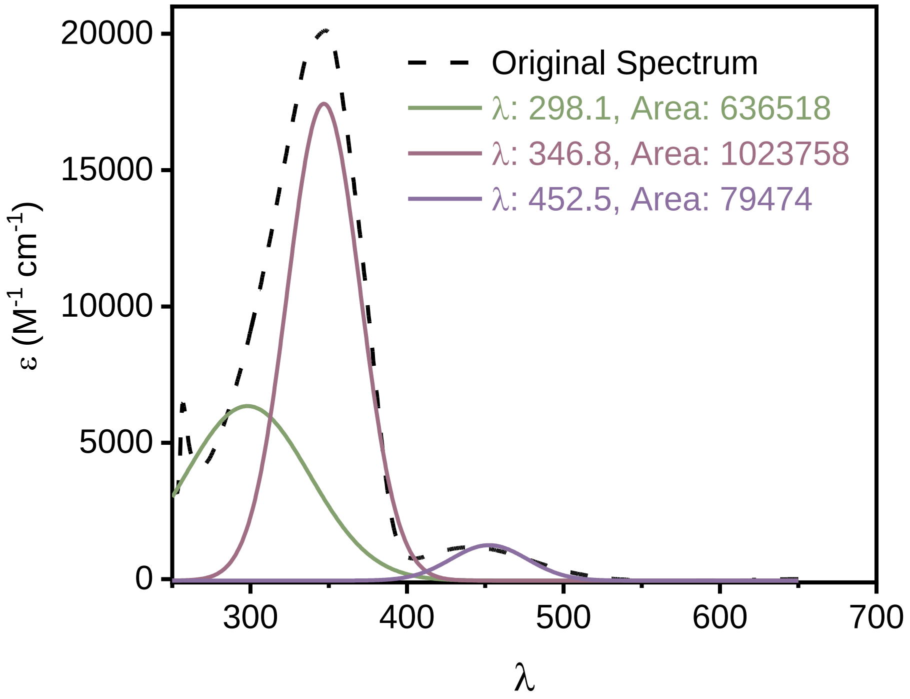

Figure S284. Deconvoluted UV-vis Spectrum of (*E*)-**F_2,*m*-H_2**

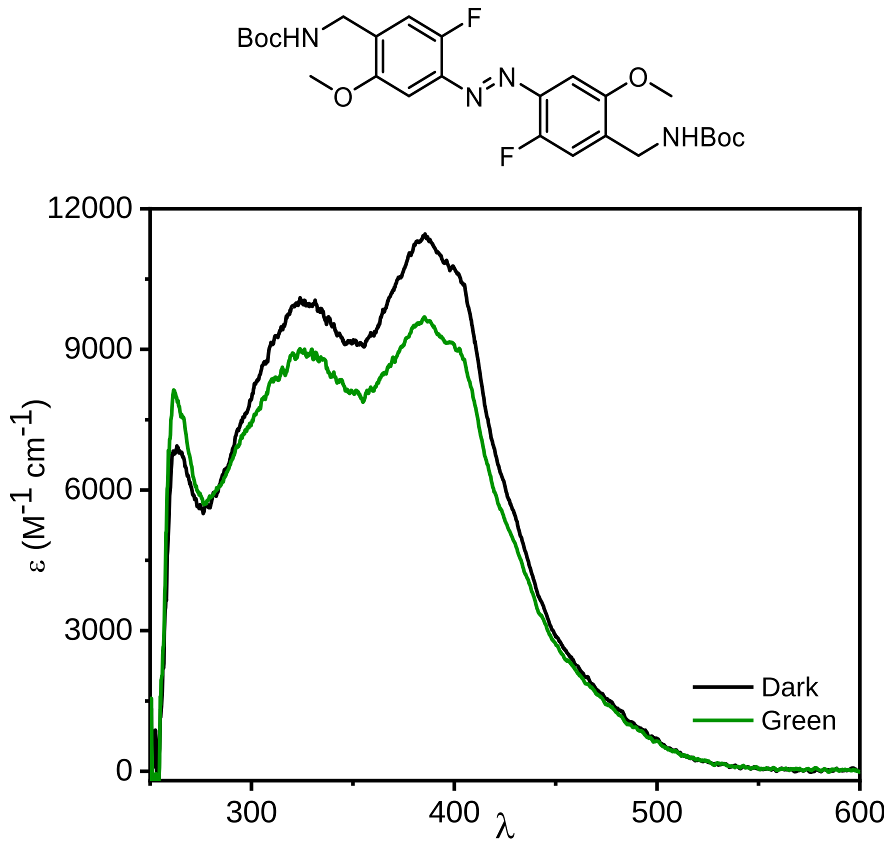

Figure S285. UV-vis Spectrum of **F$_2$,*m*-OMe$_2$** in the dark (100% *E*) and green (50% *Z*) state.

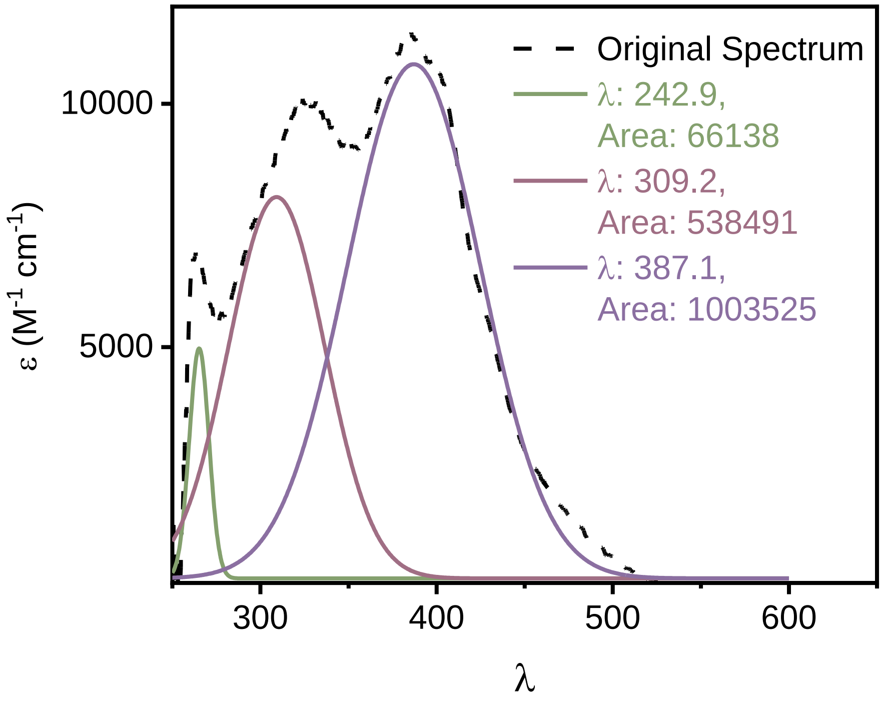

Figure S286. Deconvoluted UV-vis Spectrum of (*E*)-**F$_2$,*m*-OMe$_2$**

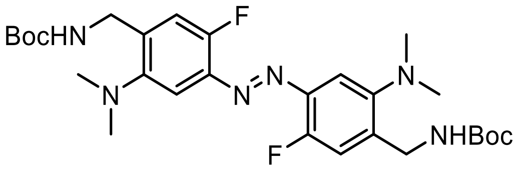

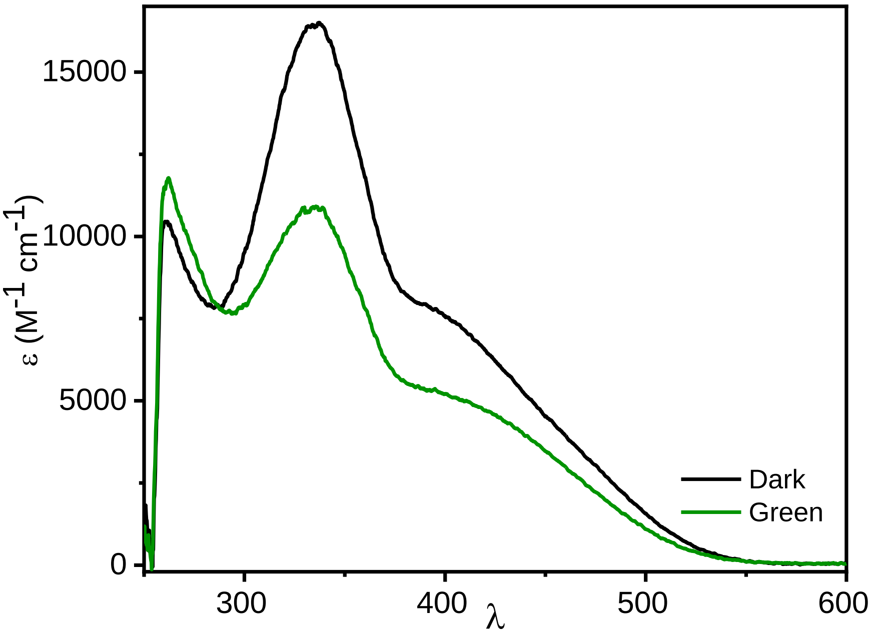

Figure S287. UV-vis Spectrum of **F$_2$,*m*-(NMe$_2$)$_2$** in the dark (100% *E*) and green (48% *Z*) state.

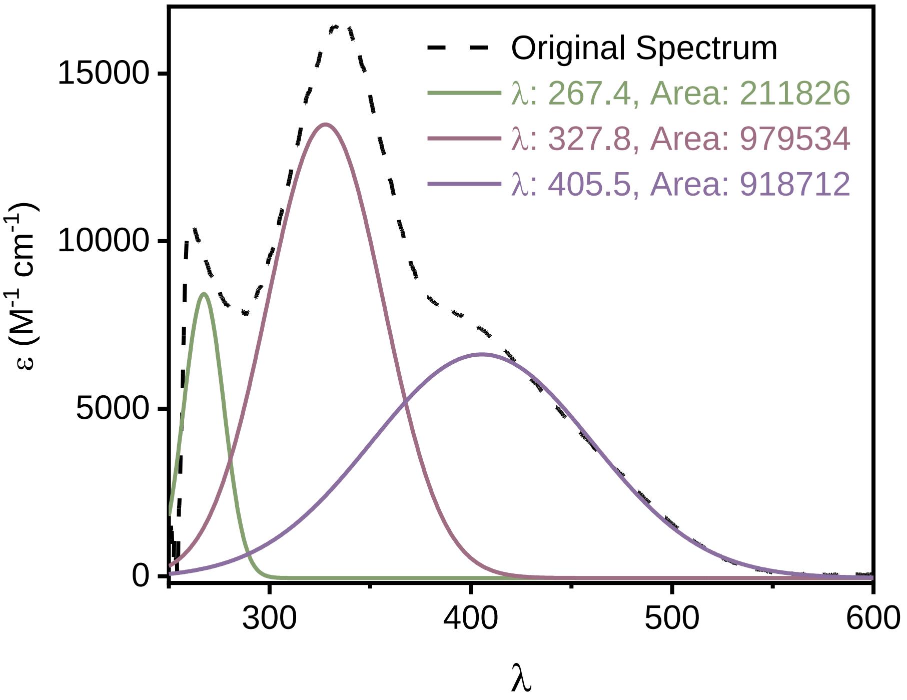

Figure S288. Deconvoluted UV-vis Spectrum of (*E*)-**F$_2$,*m*-(NMe$_2$)$_2$**

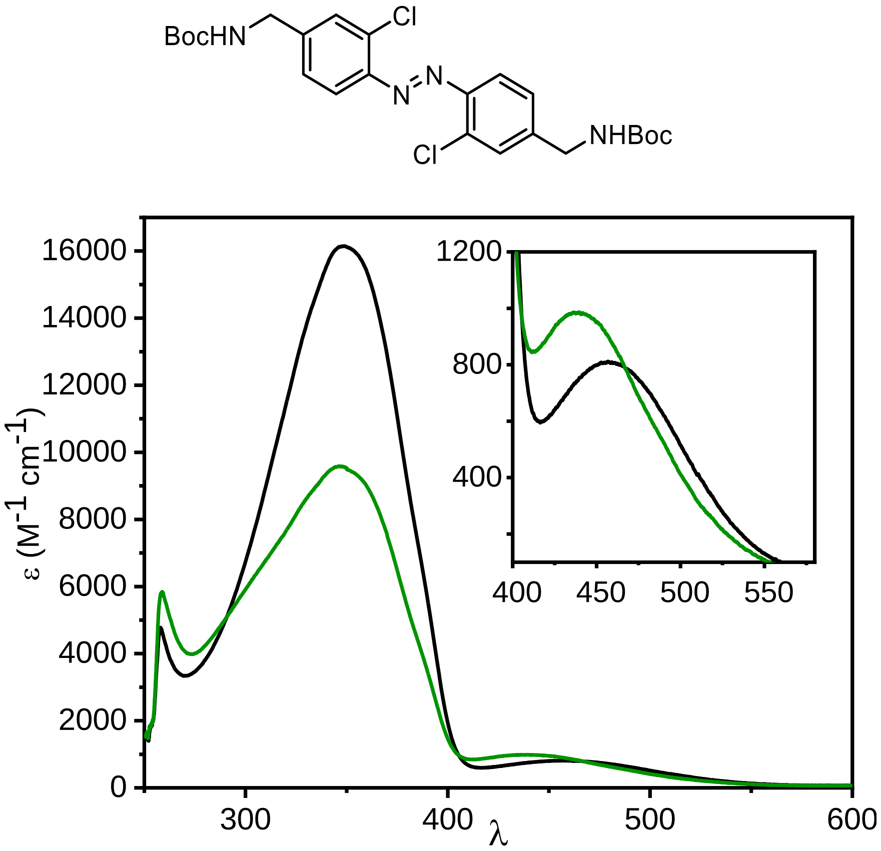

Figure S289. UV-vis Spectrum of **Cl_2,*m*-H_2** in the dark (100% *E*) and green (50% *Z*) state.

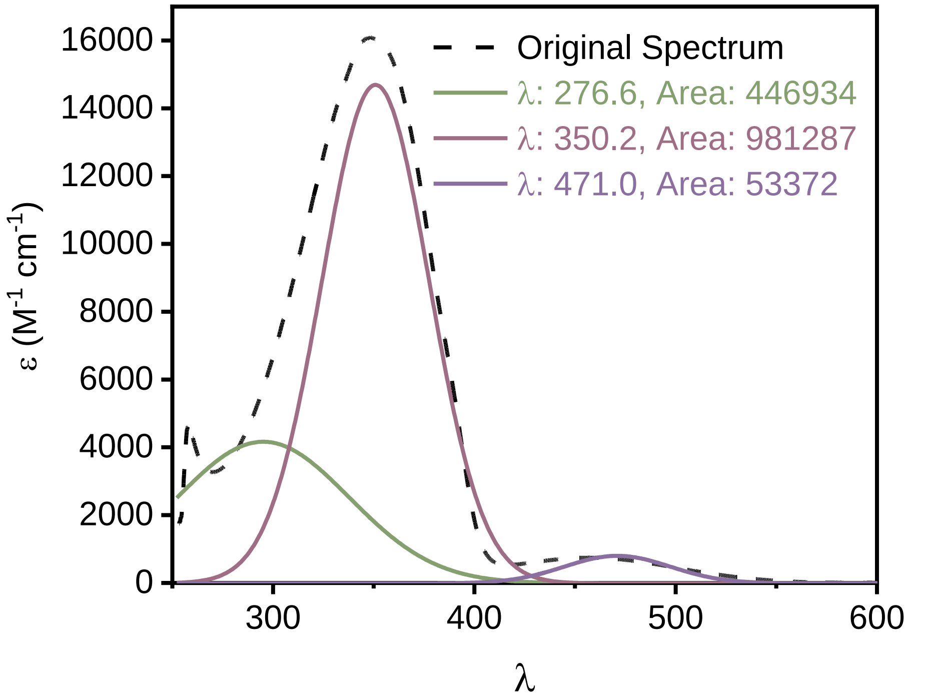

Figure S290. Deconvoluted UV-vis Spectrum of (*E*)-**Cl_2,*m*-H_2**

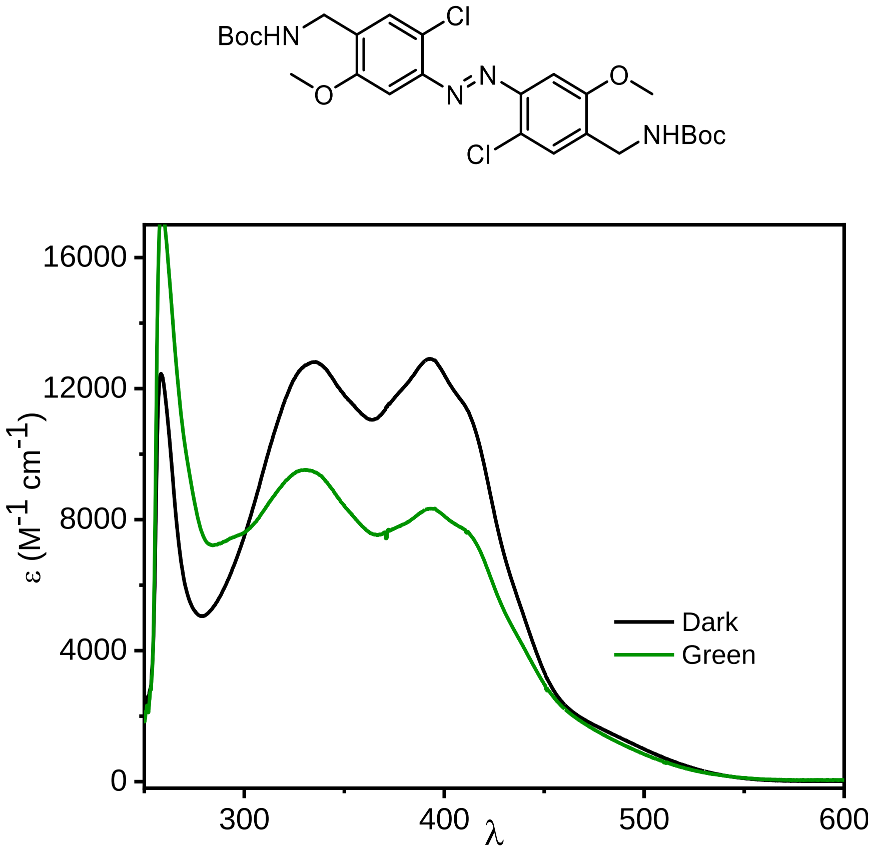

Figure S291. UV-vis Spectrum of **Cl_2,*m*-OMe_2** in the dark (100% *E*) and green (77% *Z*) state.

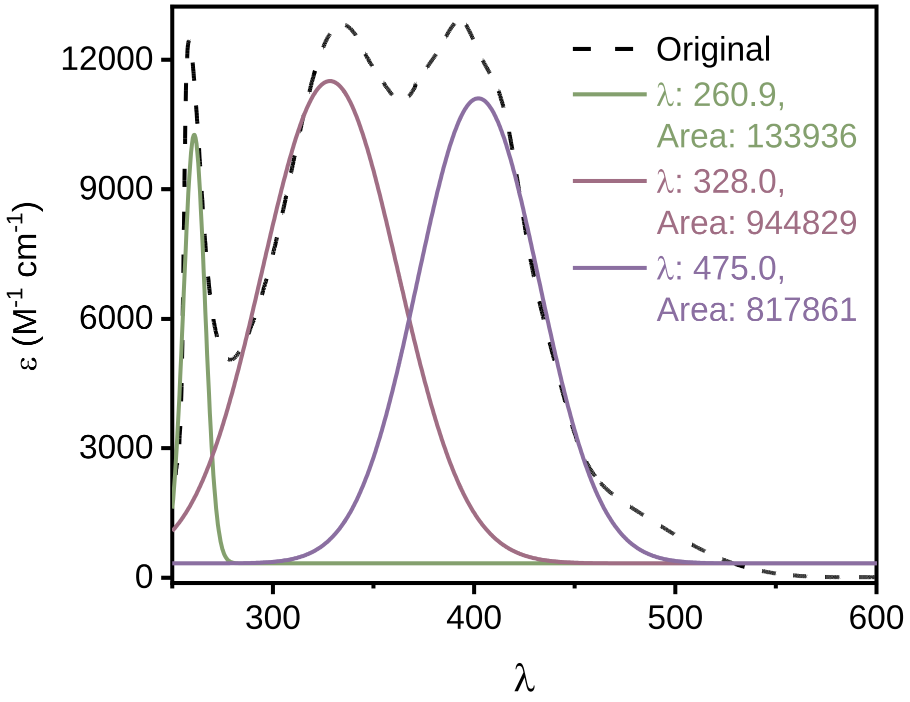

Figure S292. Deconvoluted UV-vis Spectrum of (*E*)-**Cl_2,*m*-OMe_2**

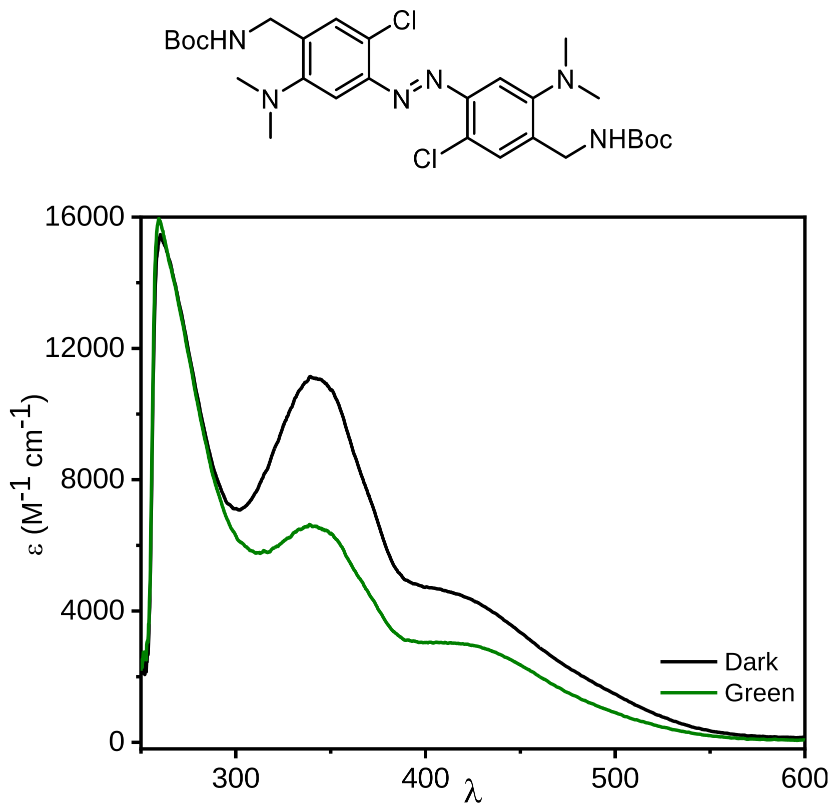

Figure S293. UV-vis Spectrum of **Cl_2,*m*-(NMe_2)$_2$** in the dark (100% *E*) and green (65% Z) state.

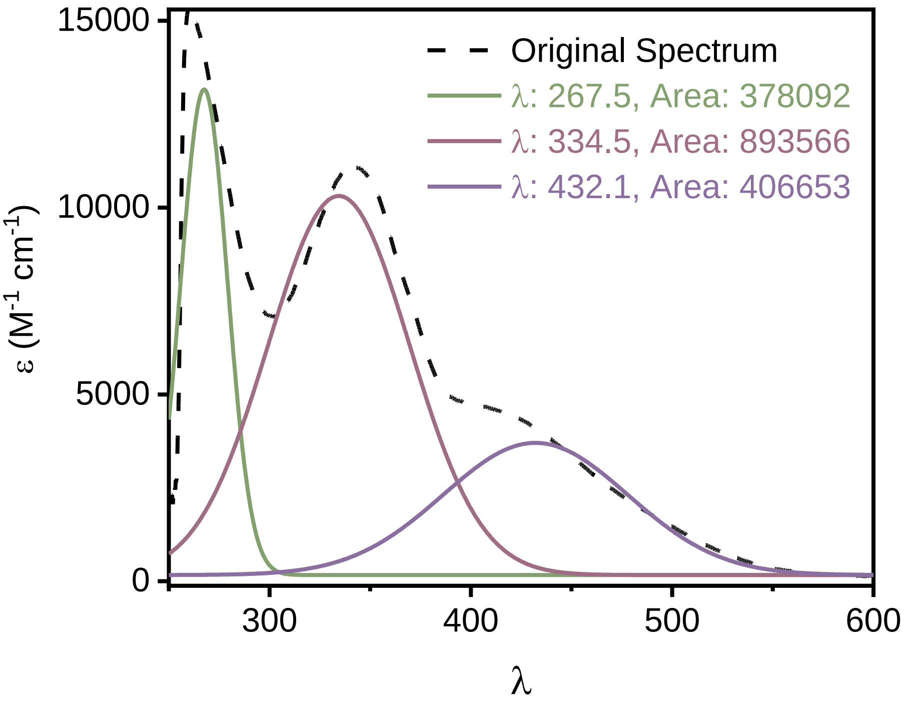

Figure S294. Deconvoluted UV-vis Spectrum of (*E*)-**Cl_2,*m*-(NMe_2)$_2$**

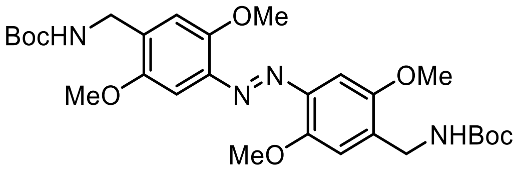

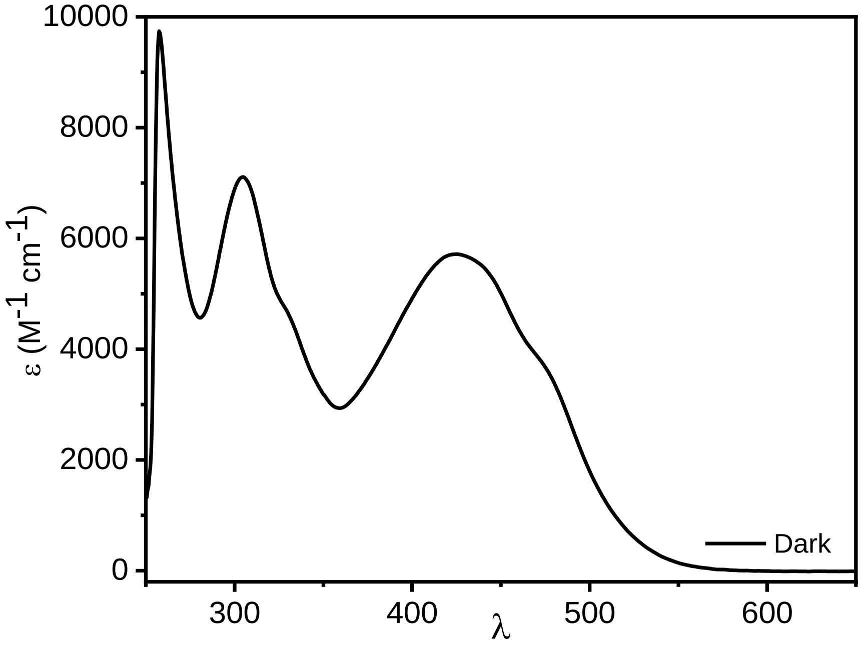

Figure S295. UV-vis Spectrum of **OMe$_2$,*m*-OMe$_2$** in the dark (100% *E*) state

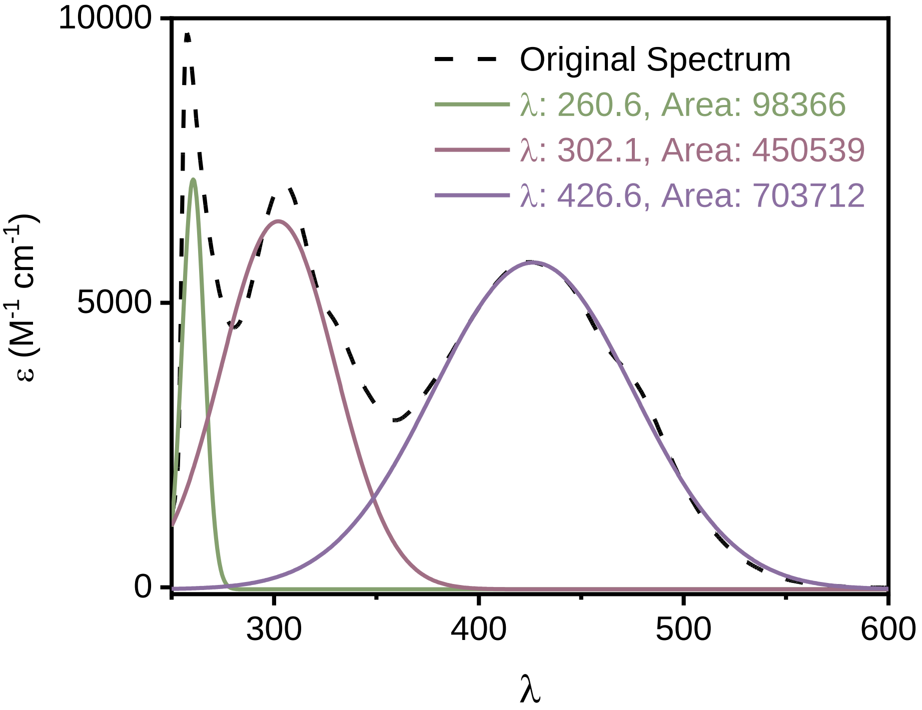

Figure S296. Deconvoluted UV-vis Spectrum of (*E*)-**OMe$_2$,*m*-OMe$_2$**

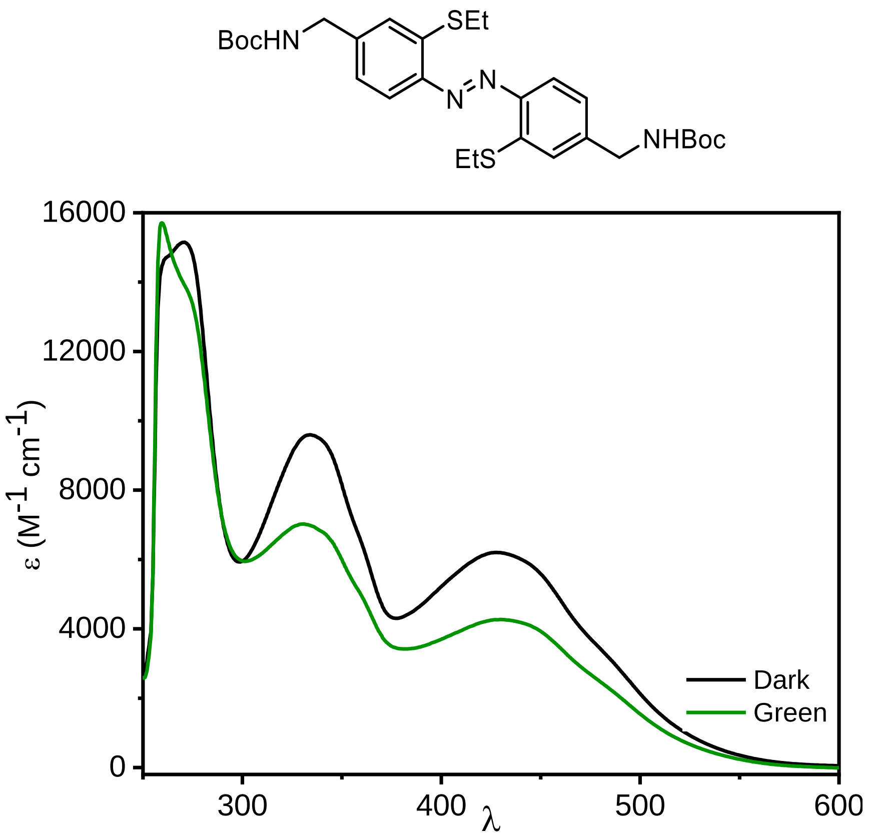

Figure S297. UV-vis Spectrum of **(SEt)$_2$,*m*-H$_2$** in the dark (100% *E*) and green (50% *Z*) state.

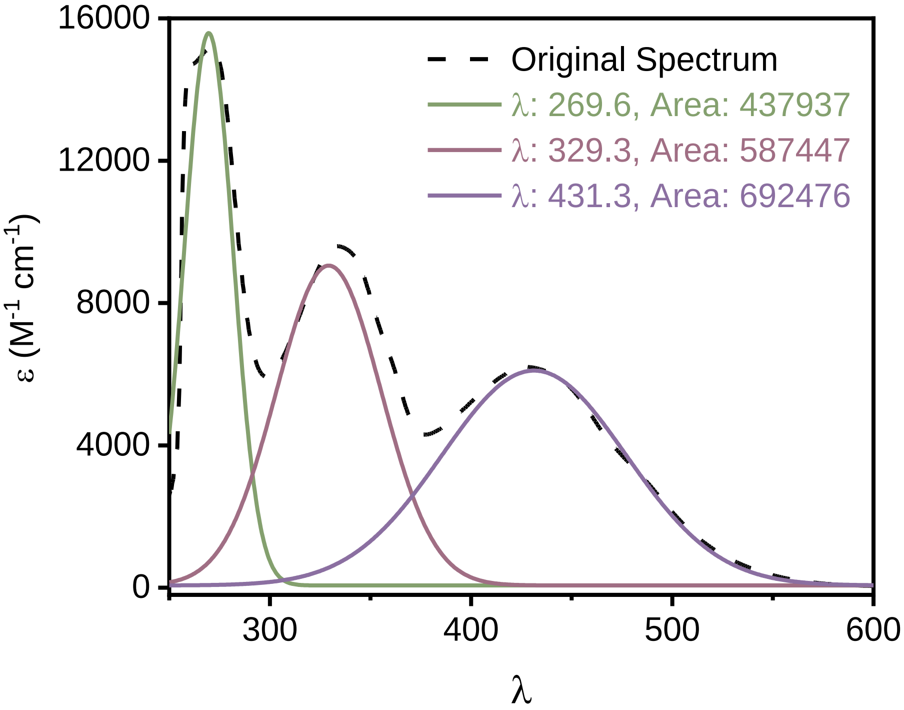

Figure S298. Deconvoluted UV-vis Spectrum of *(E)*-**(SEt)$_2$,*m*-H$_2$**

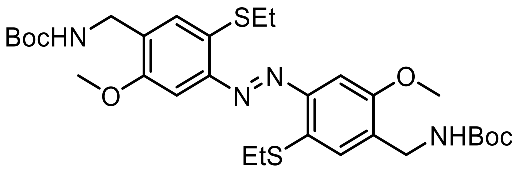

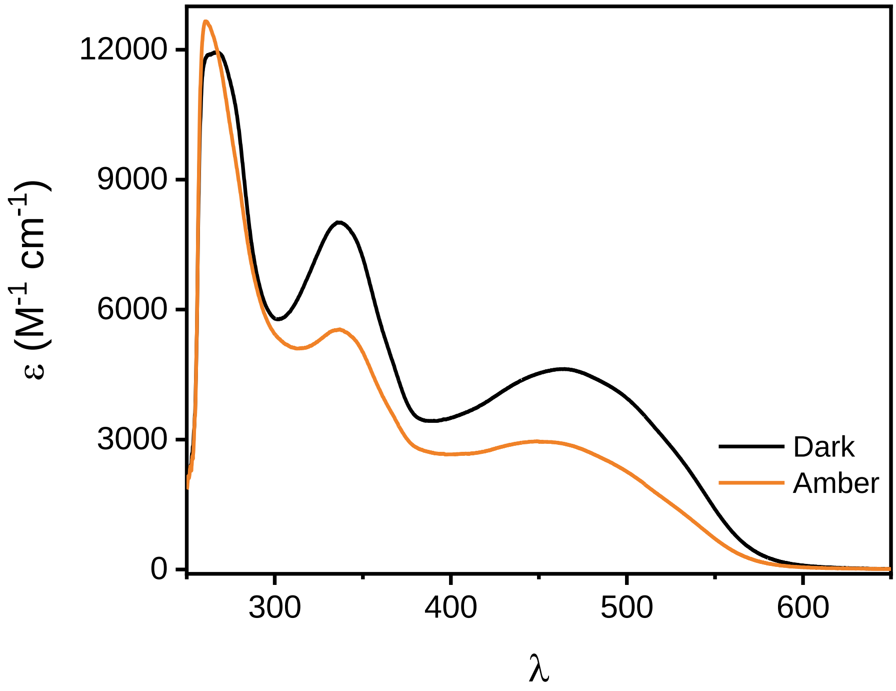

Figure S299. UV-vis Spectrum of **(SEt)$_2$,*m*-OMe$_2$** in the dark (100% *E*) and amber state.

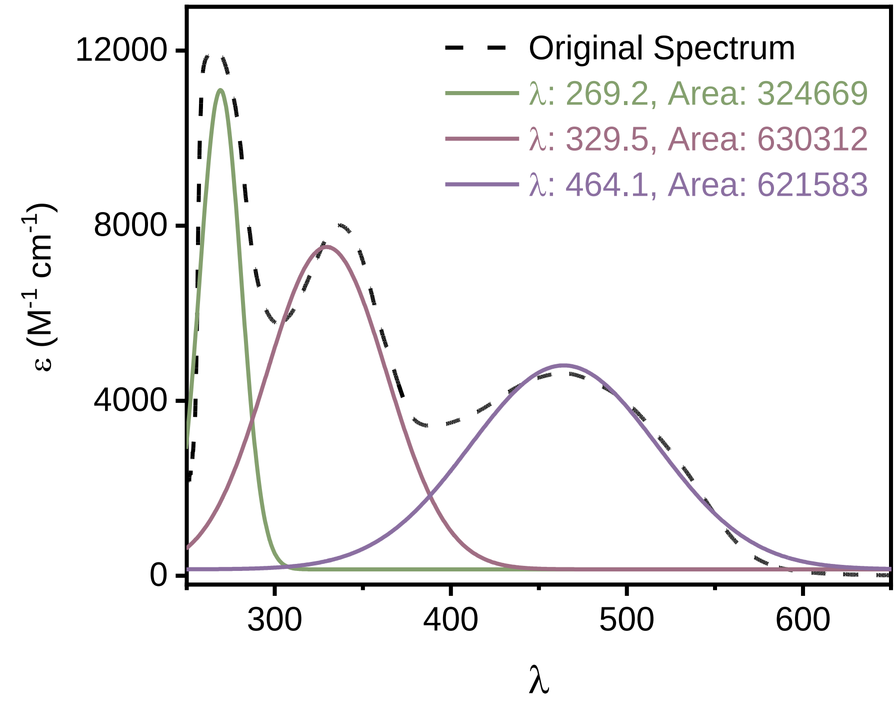

Figure S300. Deconvoluted UV-vis Spectrum of (*E*)-**(SEt)$_2$,*m*-OMe$_2$**

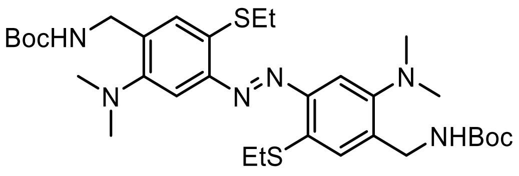

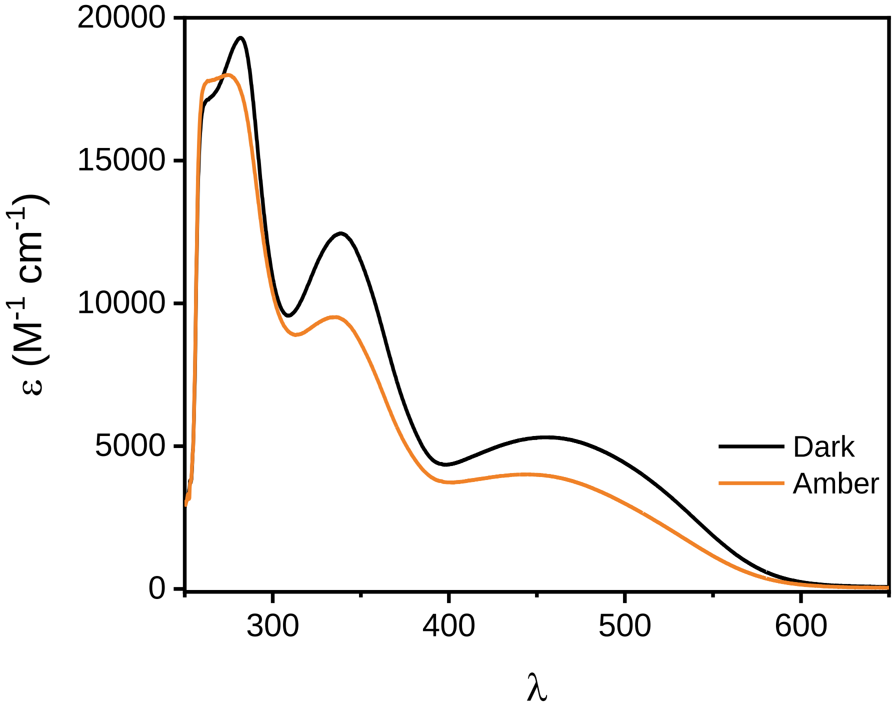

Figure S301. UV-vis Spectrum of **(SEt)$_2$,*m*-(NMe$_2$)$_2$** in the dark (100% *E*) and amber state.

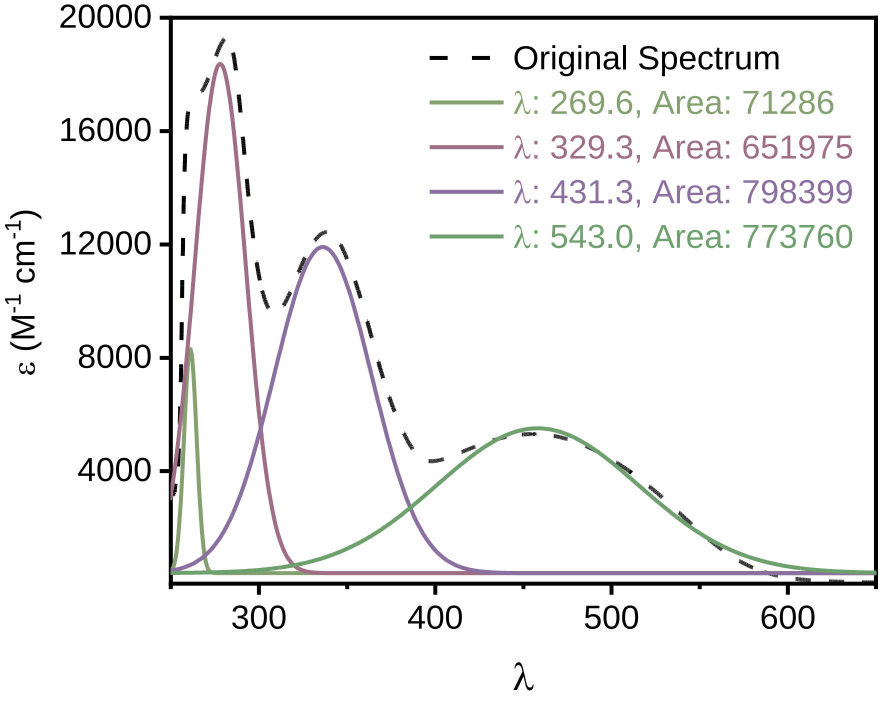

Figure S302. Deconvoluted UV-vis Spectrum of *(E)*-**(SEt)$_2$,*m*-(NMe$_2$)$_2$**

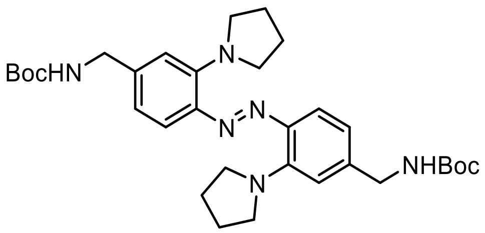

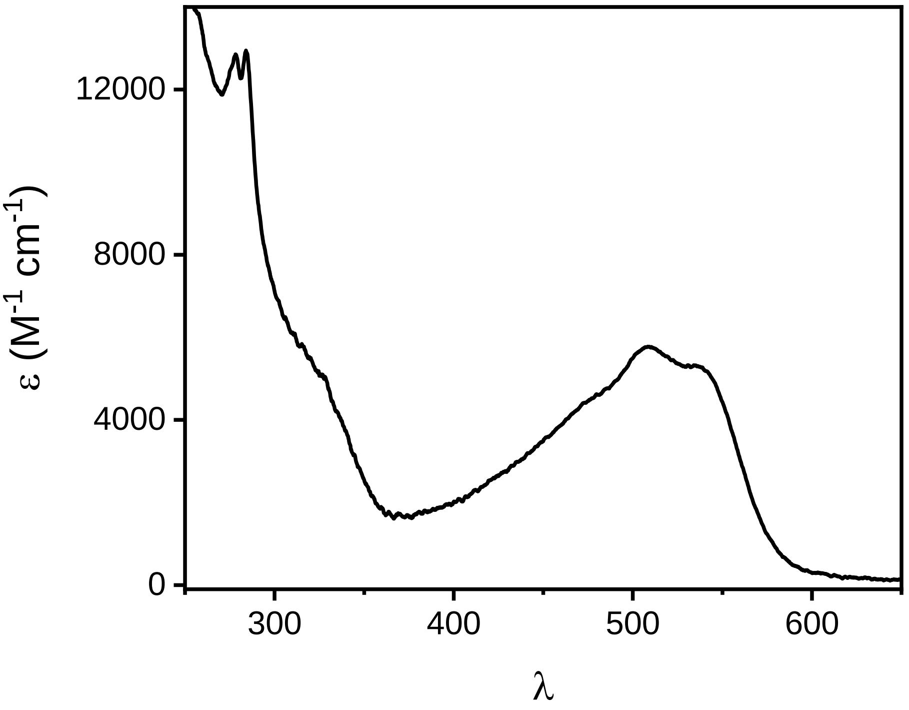

Figure S303. UV-vis Spectrum of **(N(CH$_2$)$_4$)$_2$,*m*-H** in the dark (100% *E*)

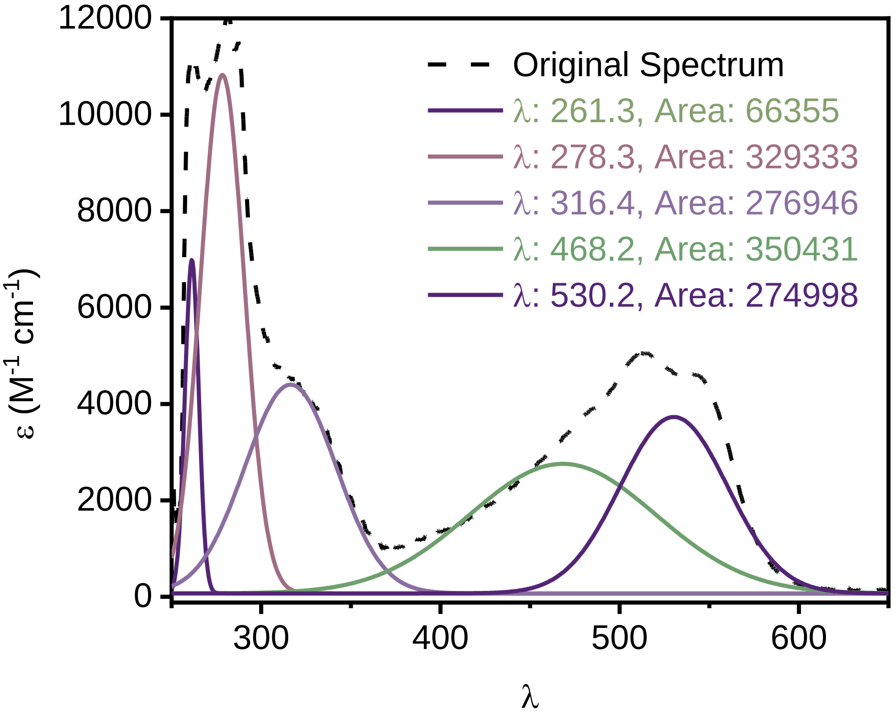

Figure S304. Deconvoluted UV-vis Spectrum of *(E)*-**(N(CH$_2$)$_4$)$_2$,*m*-H**

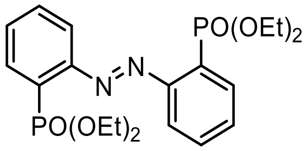

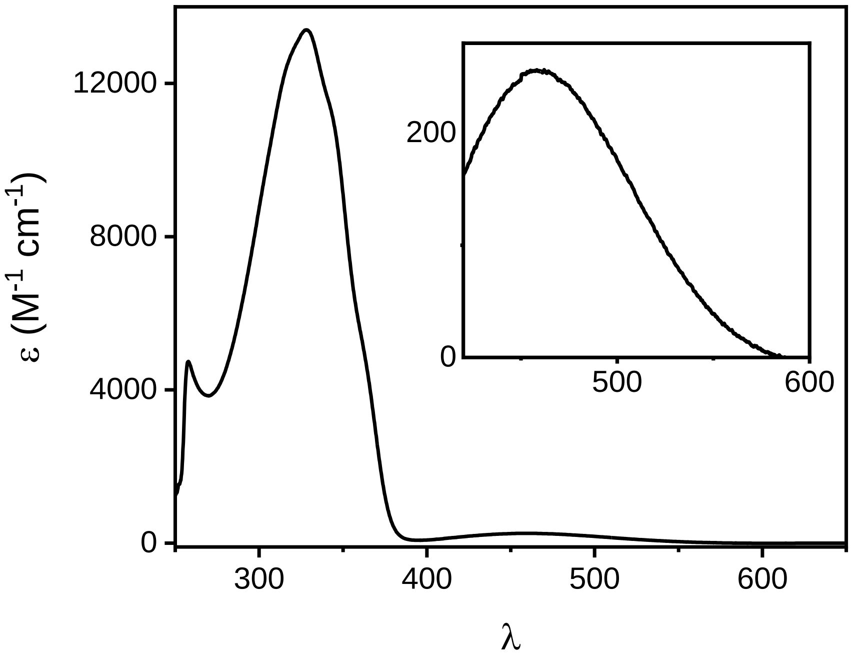

Figure S305. UV-vis Spectrum of **(PO(OEt)$_2$)$_2$,*m*-H** in the dark (100% *E*)

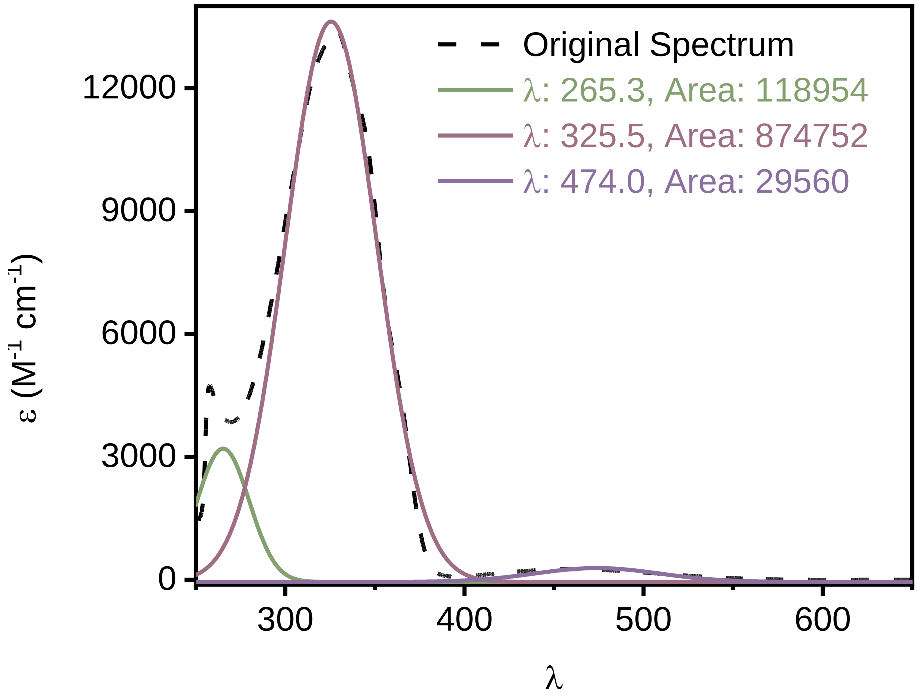

Figure S306. Deconvoluted UV-vis Spectrum of (*E*)-**(PO(OEt)$_2$)$_2$,*m*-H**

4. Photoswitching: PSS and Half-Life Determination

Photo-irradiation of liquid samples was carried out using Thorlabs high-power mounted LEDs (models M730L4, (NIR, 730 nm), M660L4 (red, 660 nm) M625L4 (red, 625 nm); M590L4 (Amber, 590 nm); M530L4 (green, 530 nm). Photo-stationary states (PSS) were determined using ^{1}H NMR spectroscopy. For faster relaxing systems, the %Z isomer was extrapolated for t=0 from 1st order expontial decay plots (equation S1) of ^{1}H NMR spectra at known intervals after irradiation of the sample.

Halflife determination were determined using ^{1}H NMR or UV-Vis spectroscopy. For the F_4 derivative half-life was determined using UV-vis spectroscopy at elevated temperatures and generating an Arrhenius plot. Slow relaxing systems were evaluated using ^{1}H NMR at room temperature and following the %Z isomer by integration over time. Z->E isomerisation for fast relaxing systems were evaluated by UV-Vis spectroscopy by irradiating the sample inside the spectrometer and following a single wavelength for the compound over time (Figure S307). Where detectable, E->Z isomerisation is included in the supporting information.

Data was fitted using Origin Pro using an exponential decay model to determine t_1 (Equation S1). Half-life ($\tau_{1/2}$) was determined using Equation S2

$$y = A_1 e^{\left(\frac{-x}{t1}\right)} \qquad (\mathbf{S1})$$

$$T_{1/2} = t_1 ln2 \qquad (\boldsymbol{S2})$$

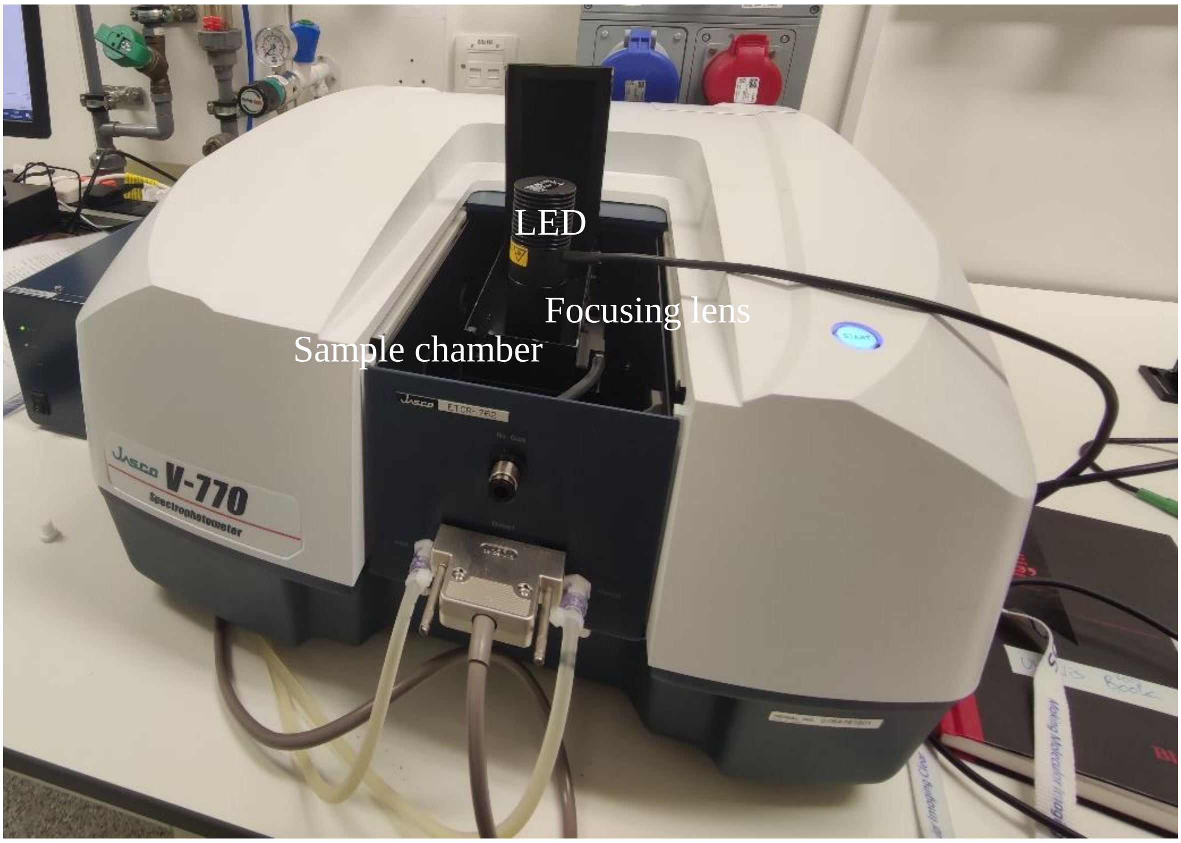

Figure 307: Experimental setup for in-situ switching

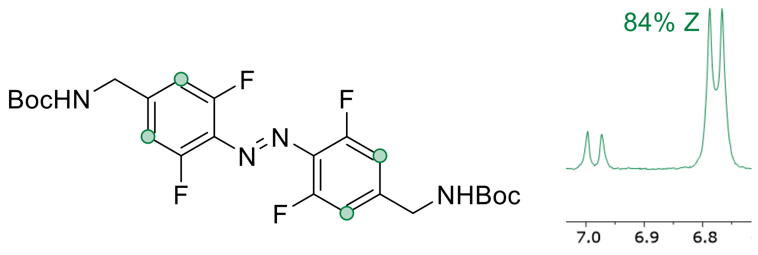

Figure S308. Partial ^{1}H NMR spectrum of **F$_4$-NHBoc** in the green (530 nm) PSS state

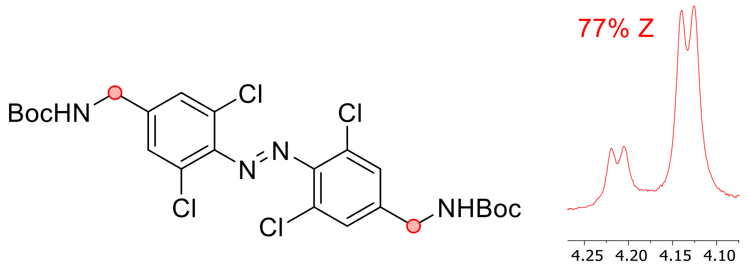

Figure S309. Partial ^{1}H NMR spectrum of **Cl$_4$-NHBoc** in the red (625 nm) PSS state

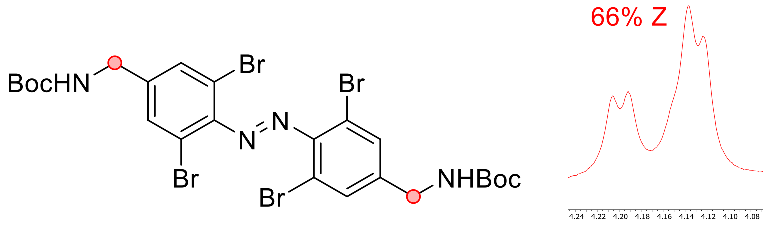

Figure S310. Partial ^{1}H NMR spectrum of **Br$_4$-NHBoc** in the red (625 nm) PSS state

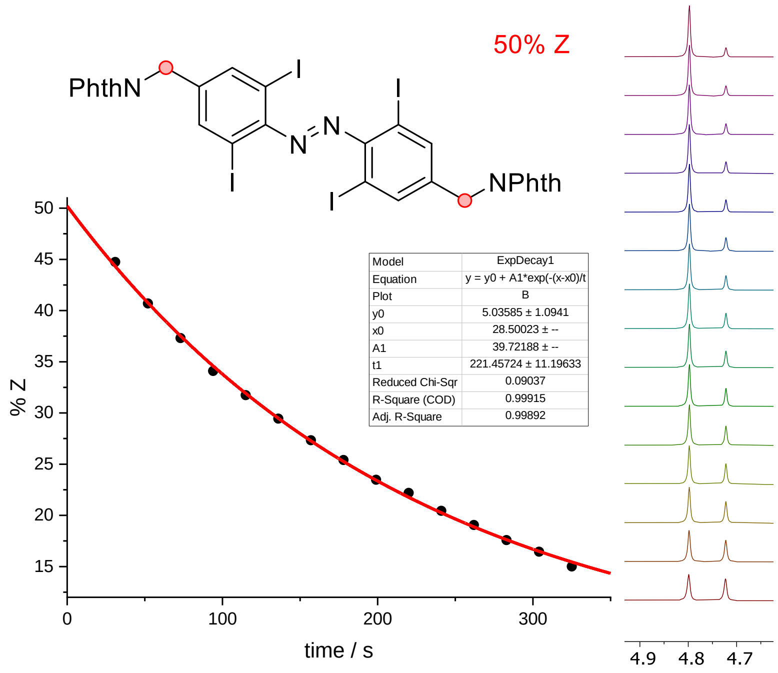

Figure S311. Partial ^{1}H NMR spectra of **I$_4$-Phth** in the red (625 nm) state over time to extrapolate PSS

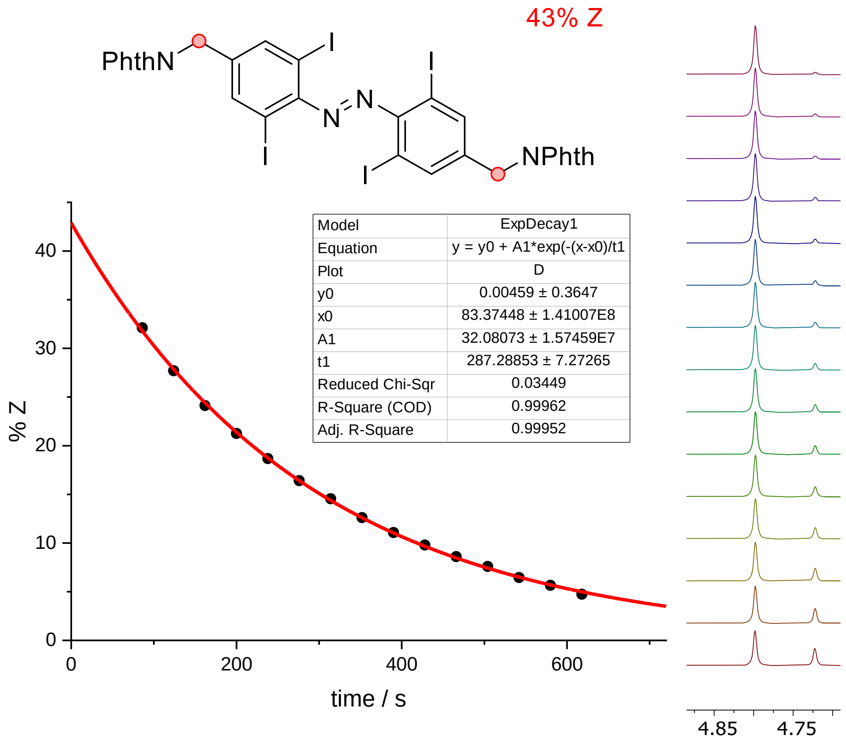

Figure S312. Partial ^{1}H NMR spectra of **I$_4$-Phth** in the deep red (660 nm) state over time to extrapolate PSS

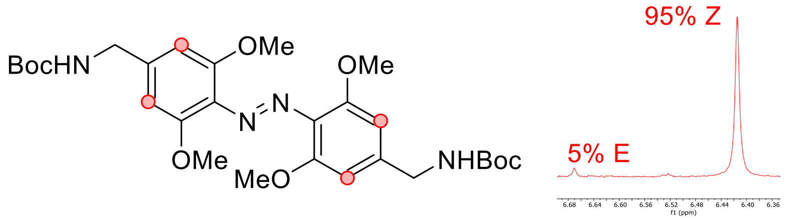

Figure S313. Partial ^{1}H NMR spectrum of **(OMe)$_4$-NHBoc** in the red (625 nm) PSS state

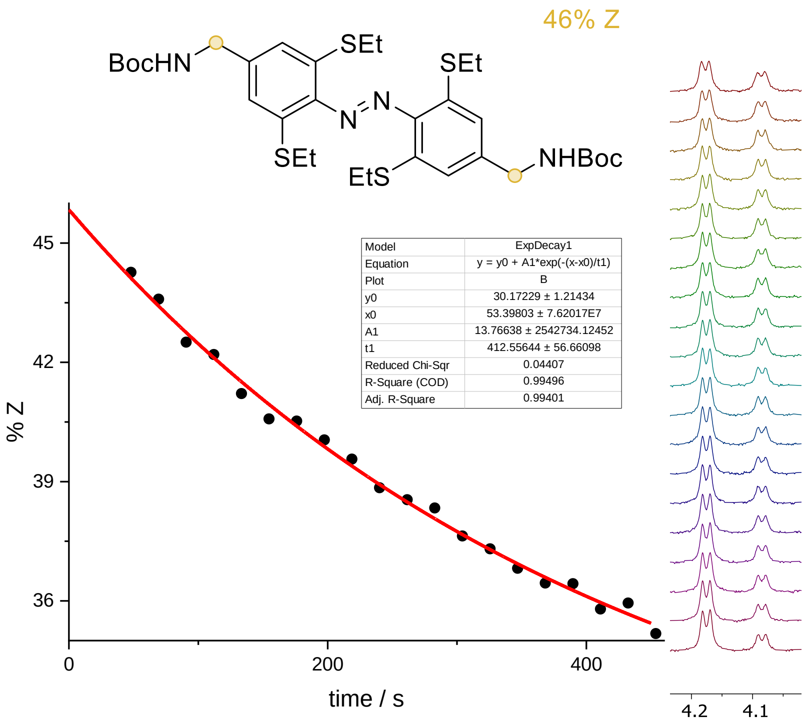

Figure S314. Partial ^{1}H NMR spectra of **(SEt)$_4$-NHBoc** in the amber (590 nm) state over time to extrapolate PSS

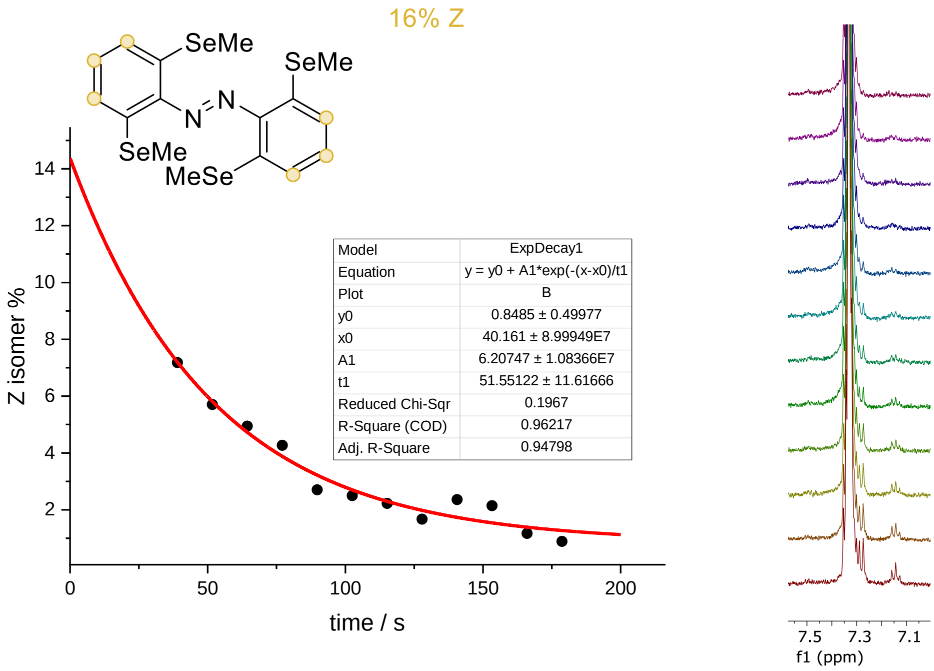

Figure S315. Partial ^{1}H NMR spectra of **(SEt)$_4$-H** in the amber (590 nm) state over time. Lower bounds of PSS determined by extrapolation to be >14%

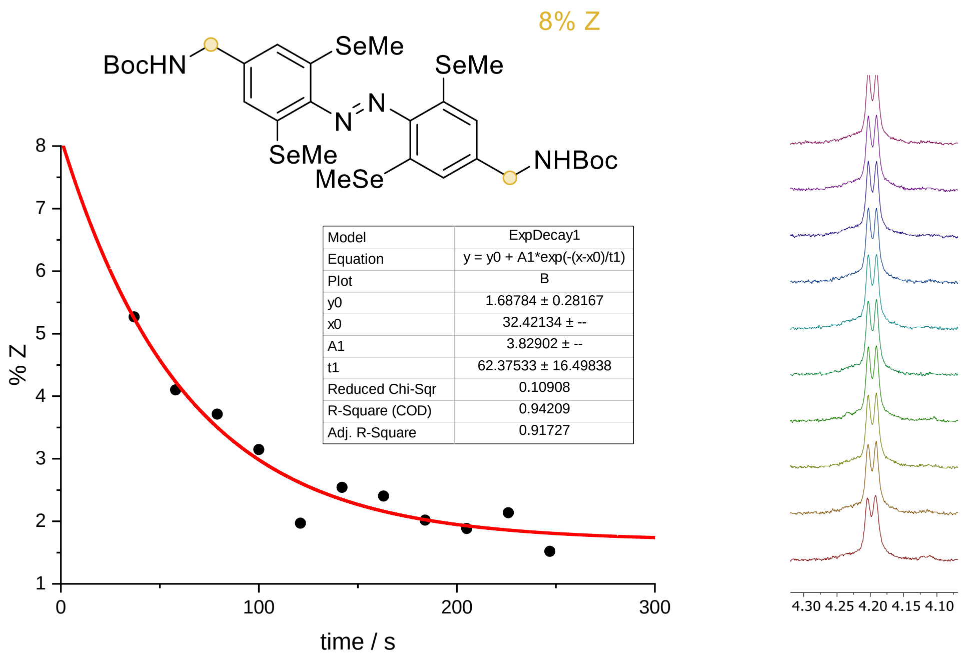

Figure S316. Partial ^{1}H NMR spectra of **(SeMe)$_4$-NHBoc** in the amber (590 nm) state over time. Lower bounds of PSS determined by extrapolation to be >8%

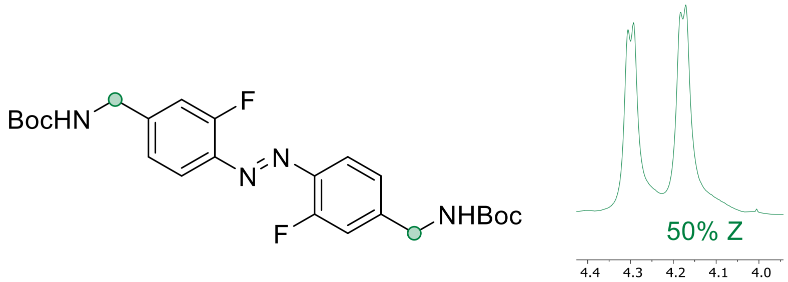

Figure S317. Partial ^{1}H NMR spectrum of **F$_2$,*m*-H$_2$** in the green (530 nm) PSS state

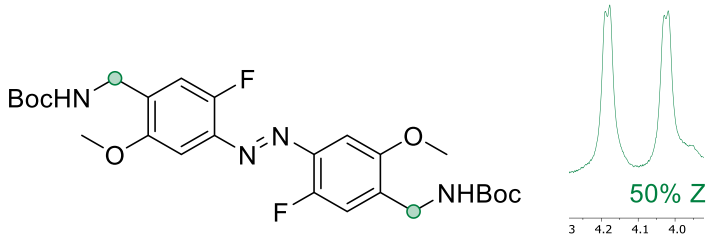

Figure S318. Partial ^{1}H NMR spectrum of **F$_2$,*m*-OMe$_2$** in the green (530 nm) PSS state

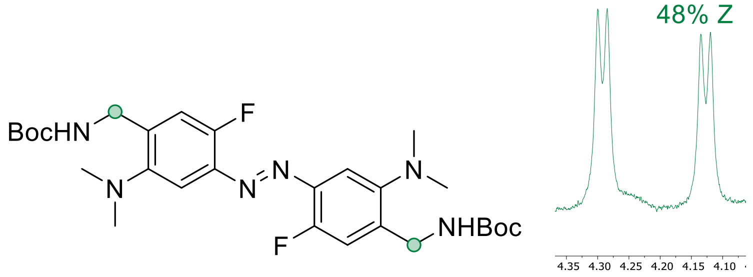

Figure S319. Partial ^{1}H NMR spectrum of **F$_2$,*m*-(NMe$_2$)$_2$** in the green (530 nm) PSS state

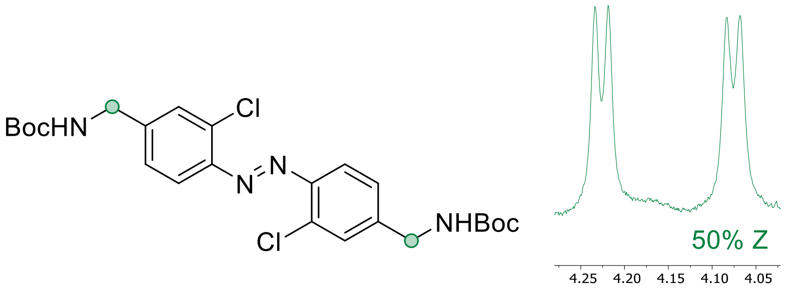

Figure S320. Partial ^{1}H NMR spectrum of **Cl$_2$,*m*-H$_2$** in the green (530 nm) PSS state

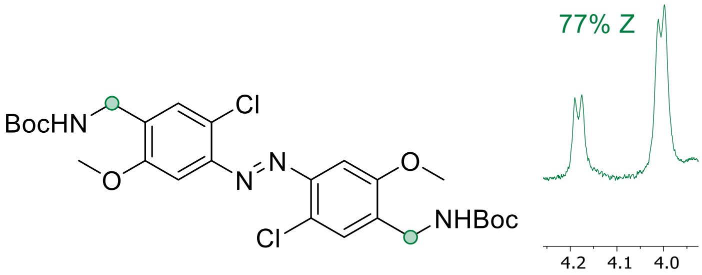

Figure S321. Partial ^{1}H NMR spectrum of **Cl$_2$,*m*-OMe$_2$** in the green (530 nm) PSS state

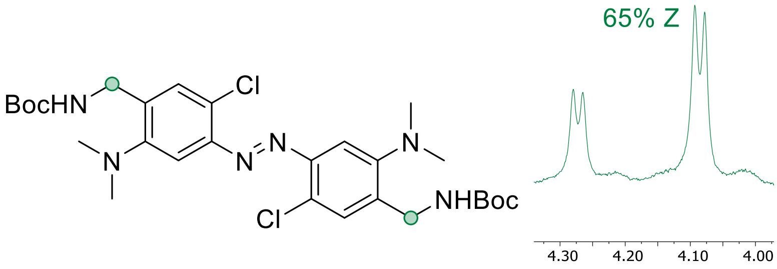

Figure S322. Partial ^{1}H NMR spectrum of **F$_2$,*m*-(NMe$_2$)$_2$** in the green (530 nm) PSS state

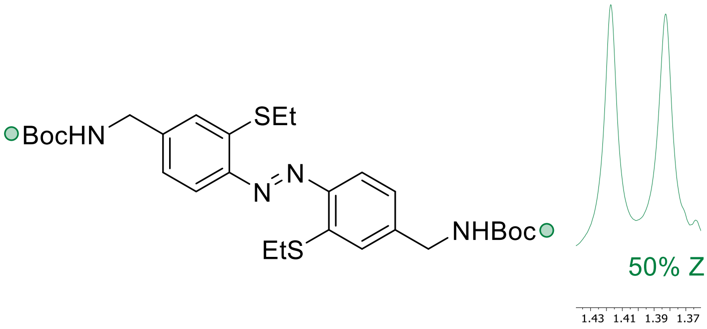

Figure S323. Partial ^{1}H NMR spectrum of **(SEt)$_2$,*m*-H** in the green (530 nm) PSS state

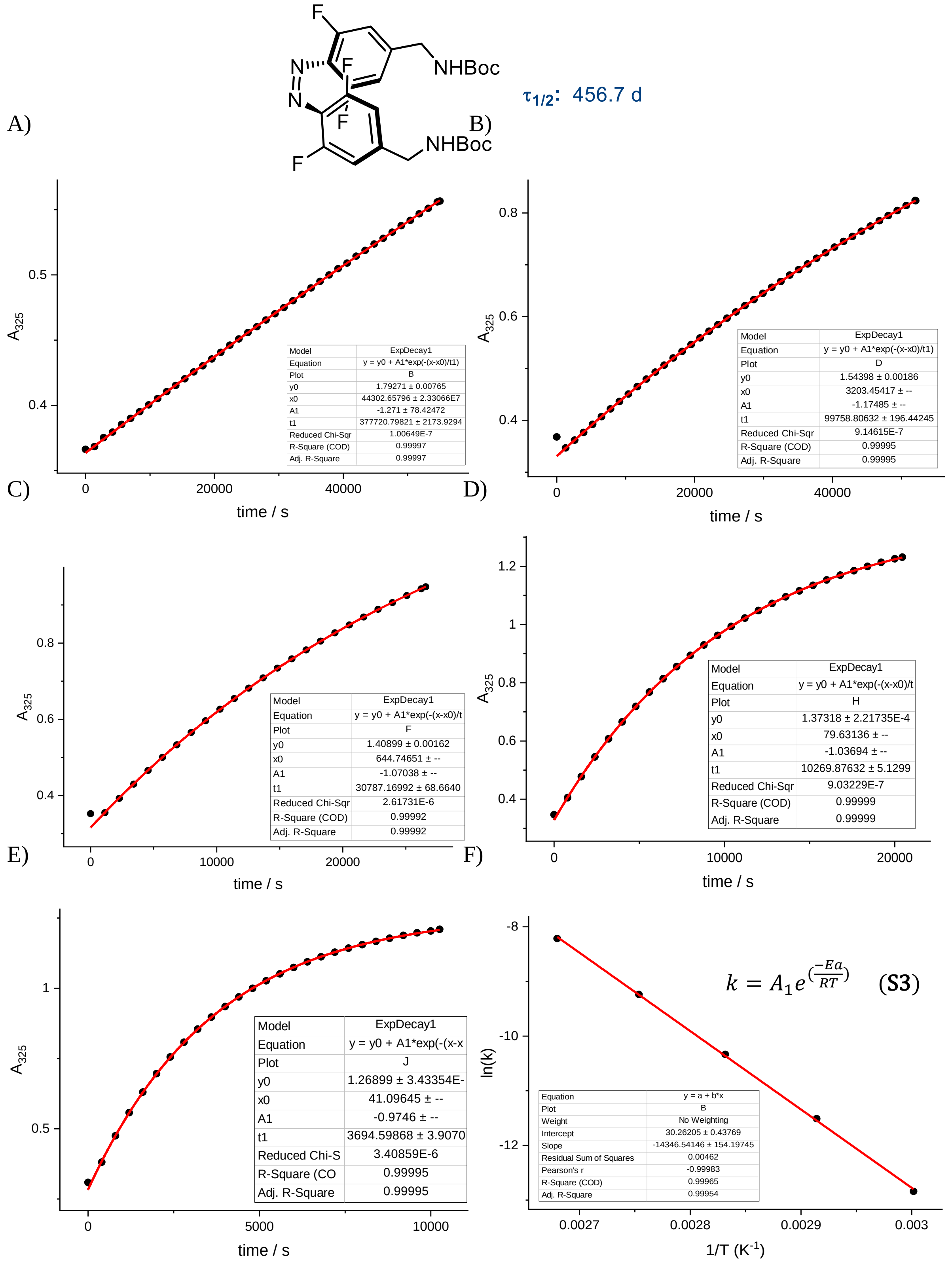

Figure S324. Lifetime plot of (*Z*)-**F$_4$-NHBoc** based on following UV-Vis absorbance over time after irradiation with 530 nm light (rt, DMSO). (A) 100 °C (B) 90 °C (C) 80 °C (D) 70 °C (E) 60 °C (F) Arrhenius Plot using data from A-E. t_1 at T = 298K is calculated using equation **S3**, and half-life is calculated using equation **S2**.

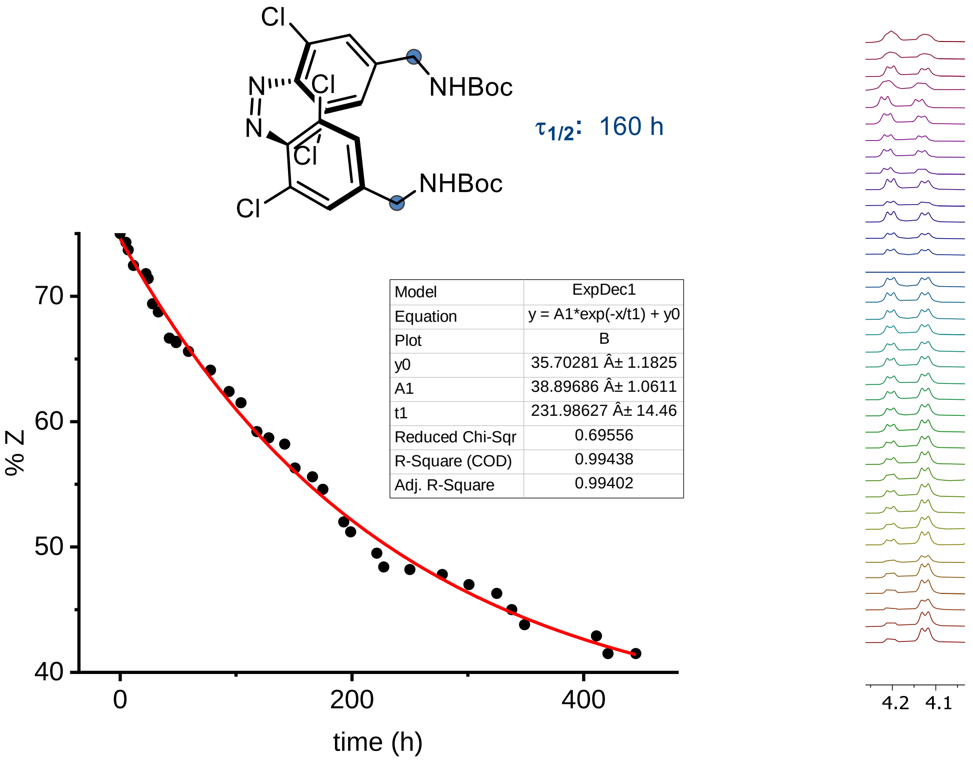

Figure S325. Lifetime plot of (*Z*)-**Cl_4-NHBoc** based on ^{1}H NMR integration of CH_2 protons over time after irradiation with 625 nm light (rt, d6-DMSO).

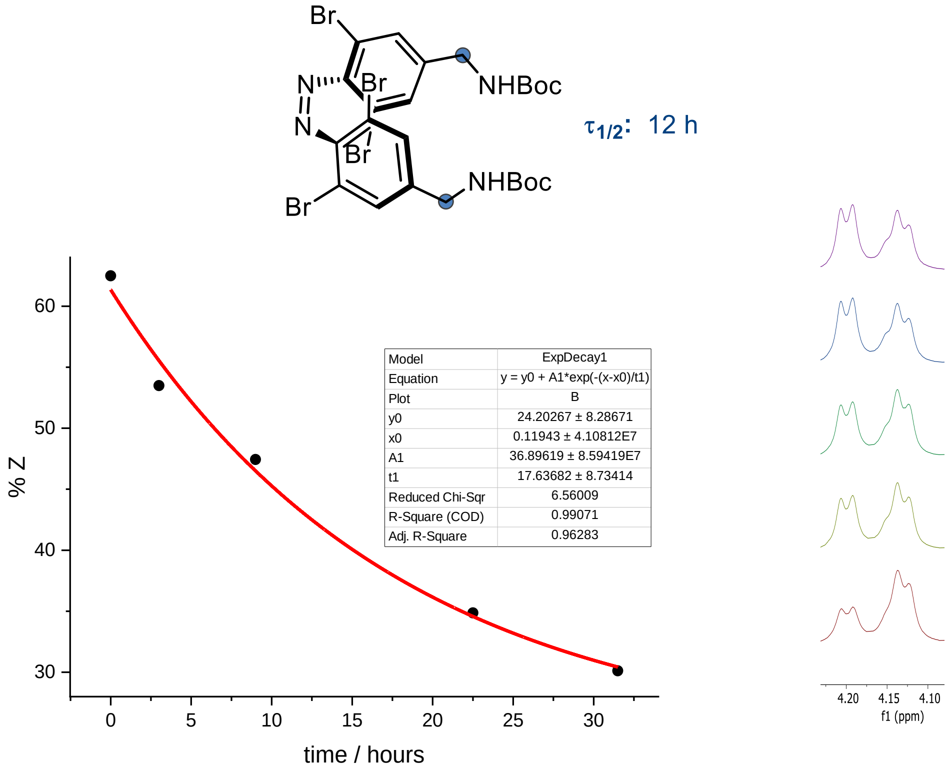

Figure S326. Lifetime plot of (*Z*)-**Br_4-NHBoc** based on ^{1}H NMR integration of CH_2 protons over time after irradiation with 625 nm light (rt, d6-DMSO).

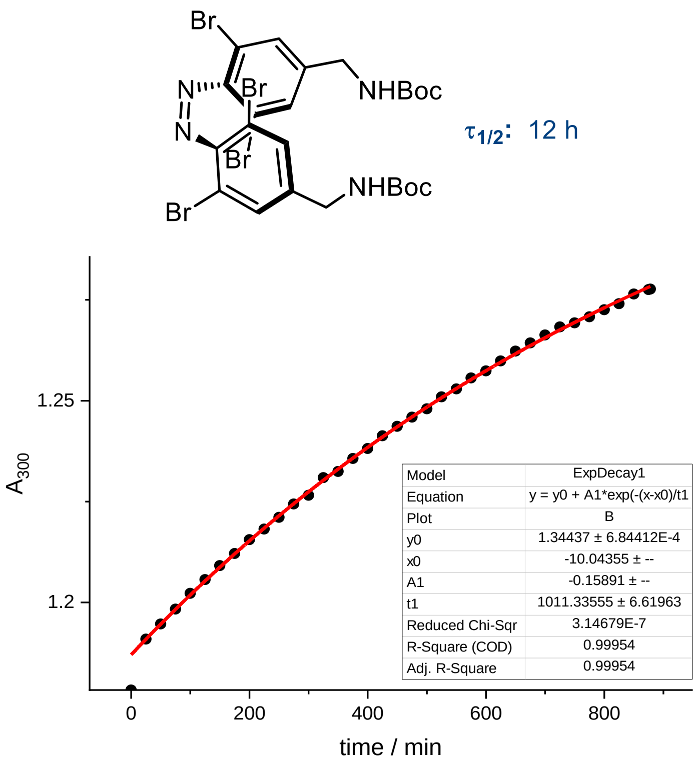

Figure S327. Lifetime plot of (*Z*)-**Br$_4$-NHBoc** based on following UV-Vis absorbance over time after irradiation with 625 nm light (rt, DMSO). Data consistent with NMR half-life data.

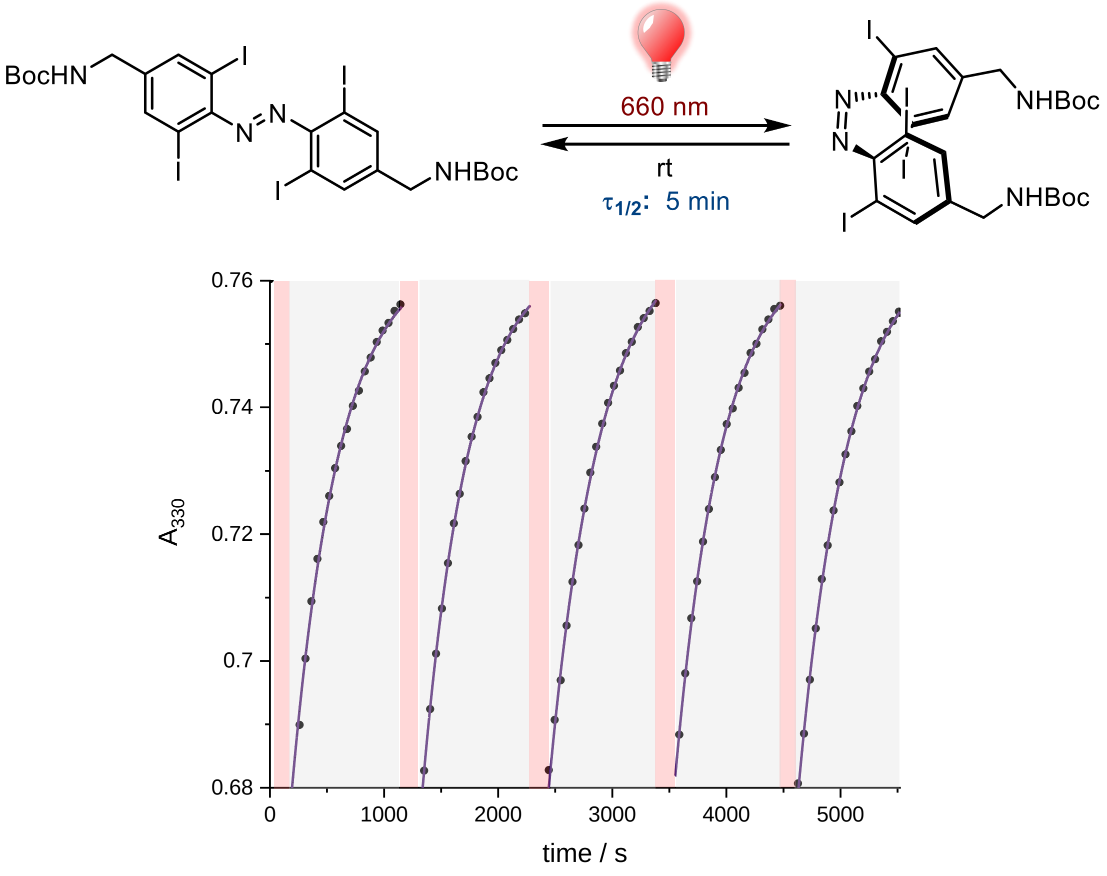

Figure S328. Reversible of switching of **I$_4$-NHBoc** over time after irradiation with 660 nm light (rt, DMSO). Dots : raw data, Line: 1st order Exponential Fit

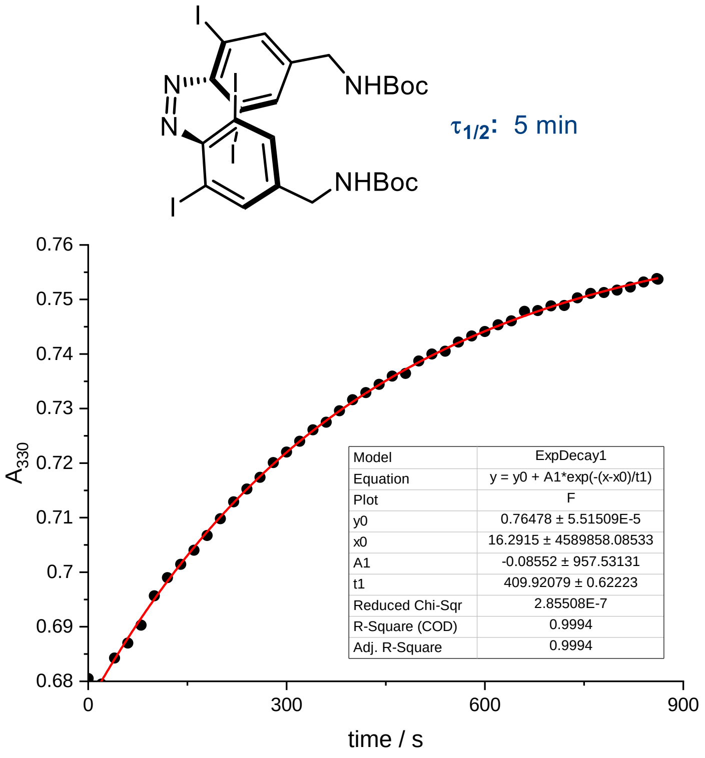

Figure S329. Lifetime plot of (*Z*)-**I$_4$-NHBoc** based on following UV-Vis absorbance over time after irradiation with 660 nm light (rt, DMSO)

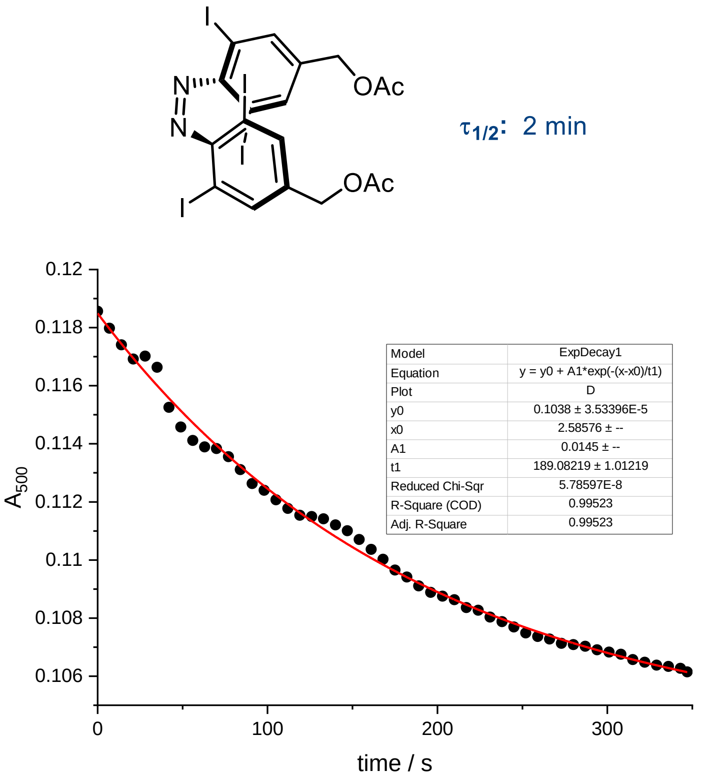

Figure S330. Lifetime plot of (*Z*)-**I$_4$-OAc** based on following UV-Vis absorbance over time after irradiation with 625 nm light (rt, DMSO)

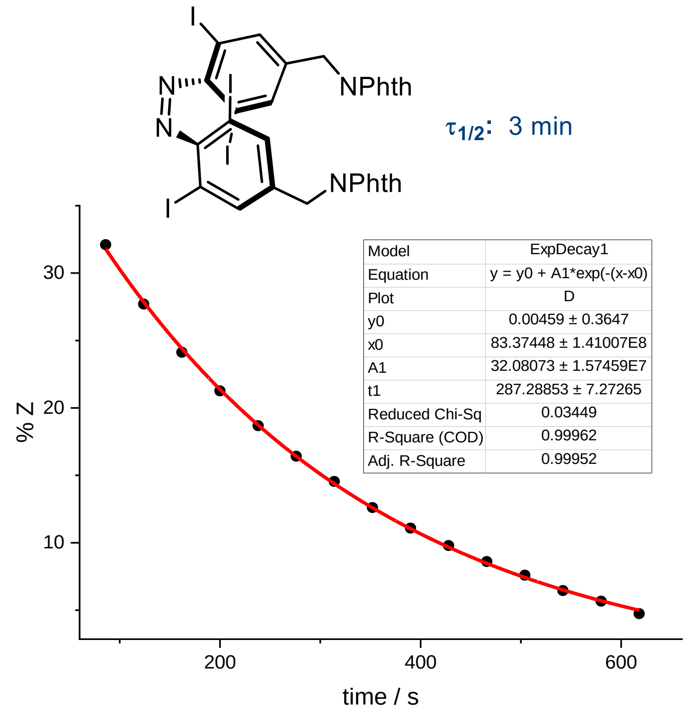

Figure S331. Lifetime plot of (*Z*)-**I$_4$-NPhth** based on ^{1}H NMR integration of CH_2 protons over time after irradiation with 660 nm light (rt, d$_6$-DMSO).

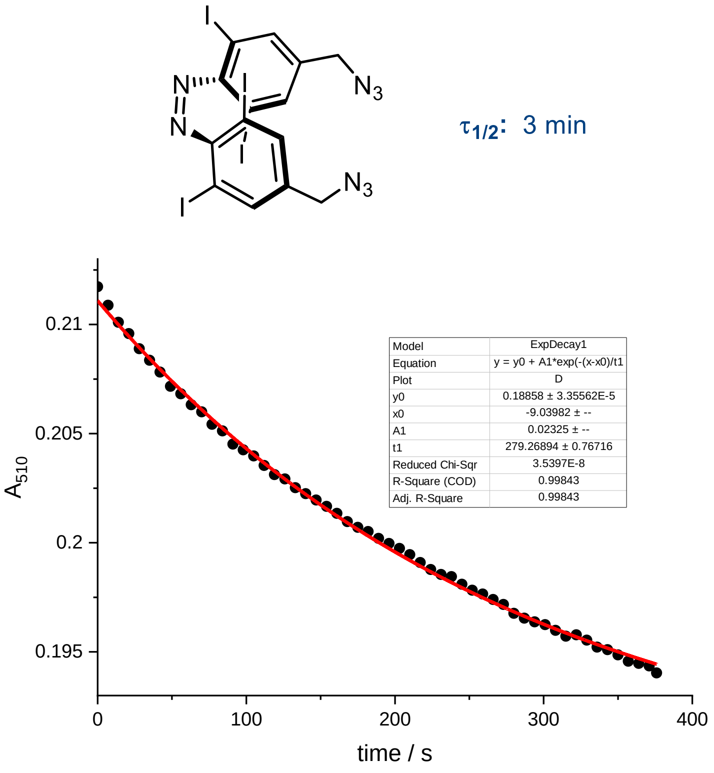

Figure S332. Lifetime plot of (*Z*)-**I$_4$-N$_3$** based on following UV-Vis absorbance over time after irradiation with 625 nm light (rt, DMSO)

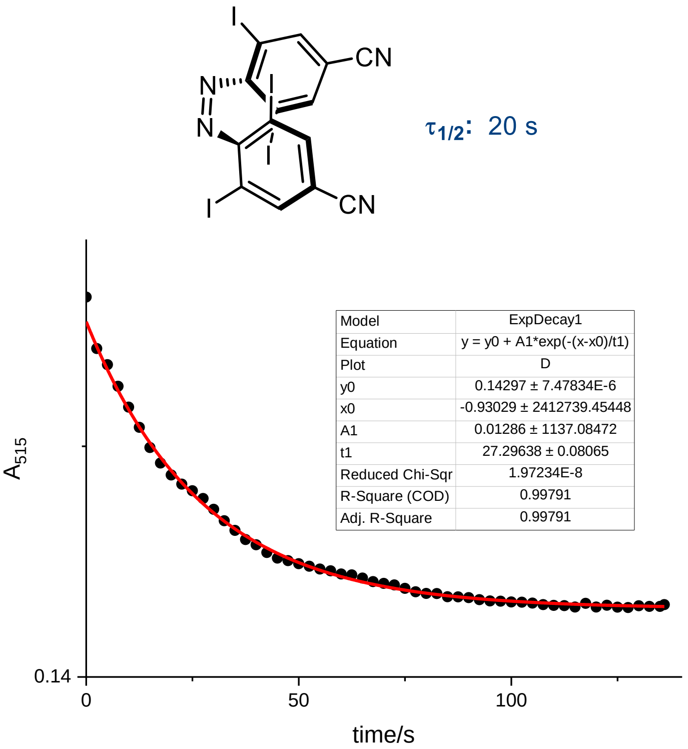

Figure S333. Lifetime plot of (*Z*)-**I$_4$-CN** based on following UV-Vis absorbance over time after irradiation with 625 nm light (rt, DMSO)

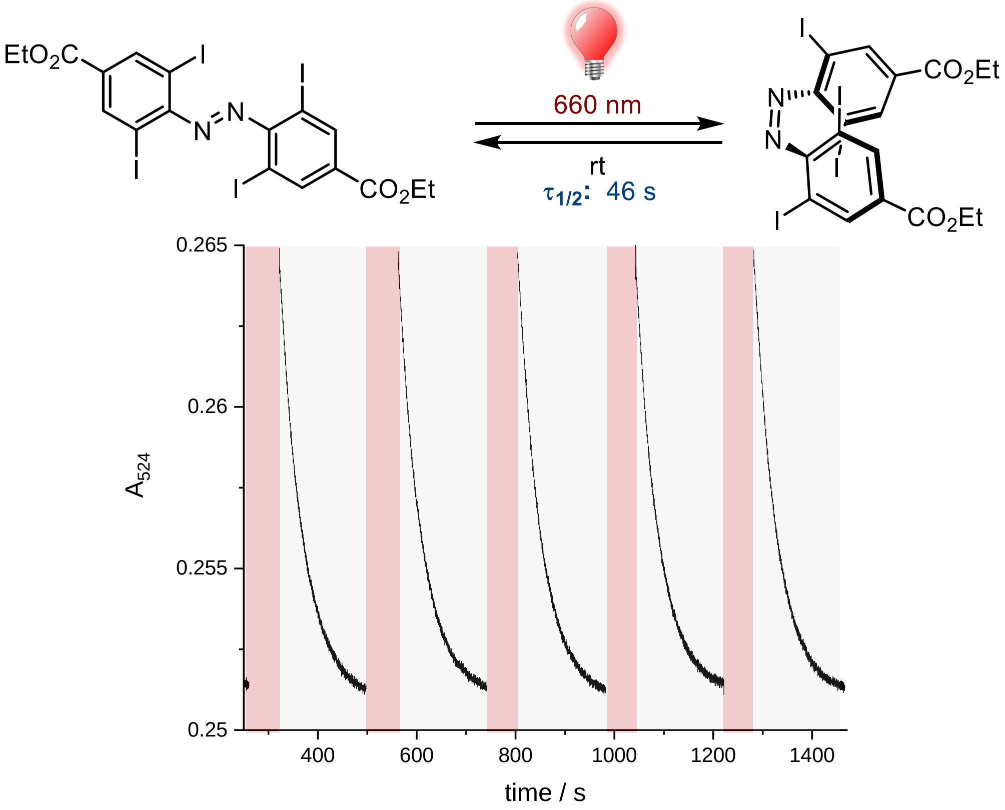

Figure S334. Reversible of switching of **I$_4$-ester** over time after irradiation with 660 nm light (rt, DMSO).

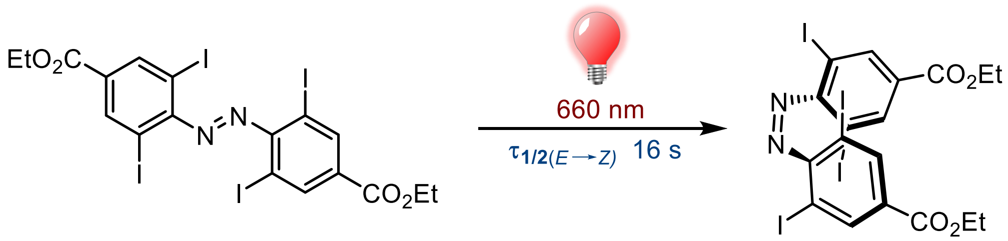

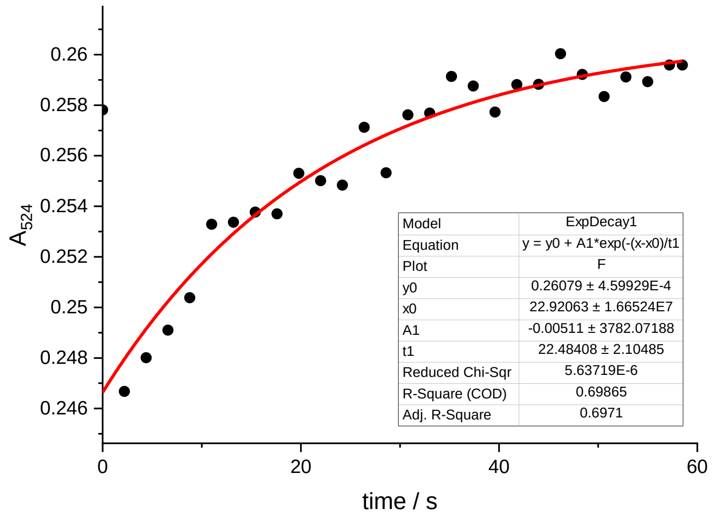

Figure S335. E → Z isomerisation of (*E*)-**I$_4$-ester** under constant irradiation of 660 nm light

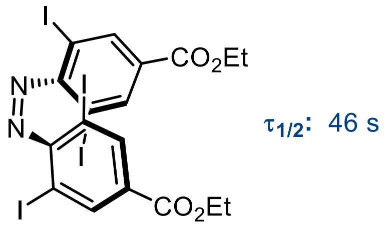

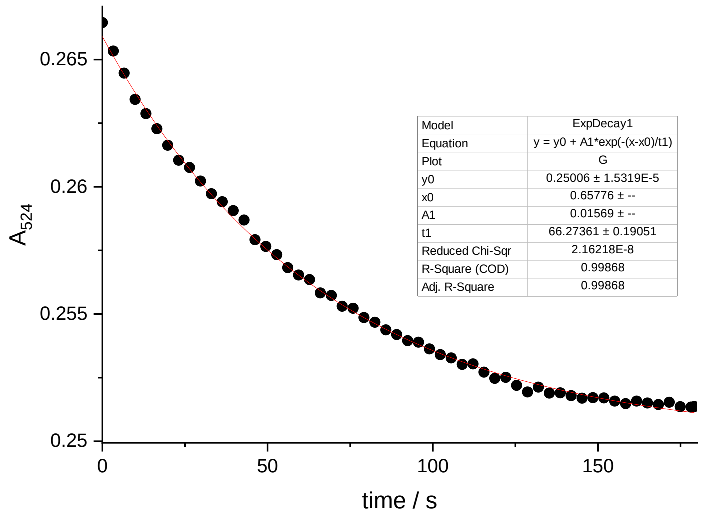

Figure S336. Lifetime plot of (*Z*)-**I$_4$-ester** based on following UV-Vis absorbance over time after irradiation with 660 nm light (rt, DMSO).

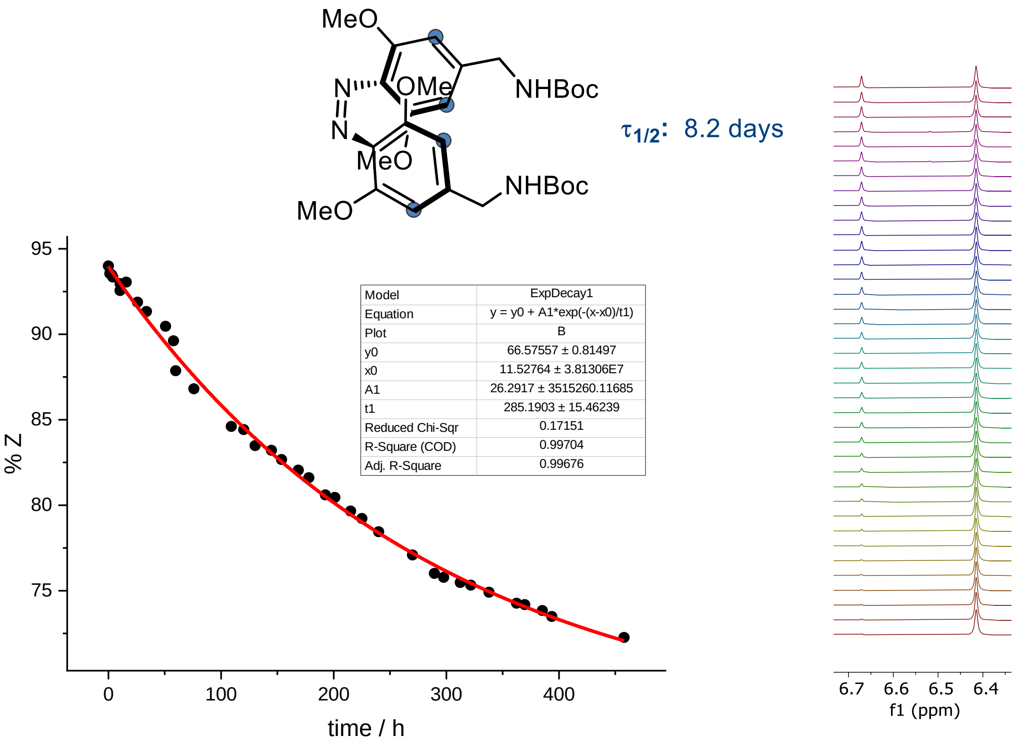

Figure S337. Lifetime plot of (*Z*)-**(OMe)$_4$-NHBoc** based on ^{1}H NMR integration of Ar protons over time after irradiation with 625 nm light (rt, d6-DMSO).

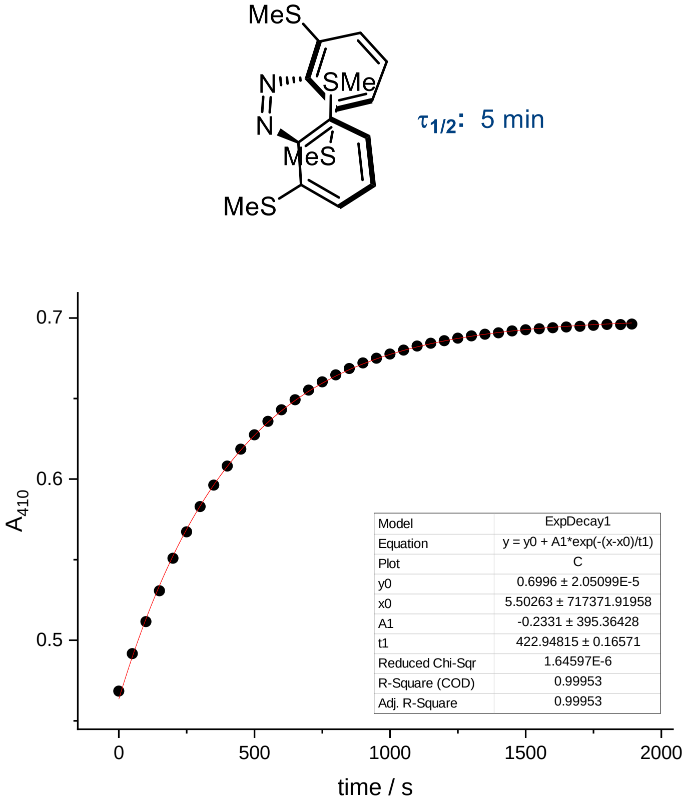

Figure S338. Lifetime plot of (*Z*)-**(SMe)$_4$-H** based on following UV-Vis absorbance over time after irradiation with 590 nm light (rt, DMSO**)**

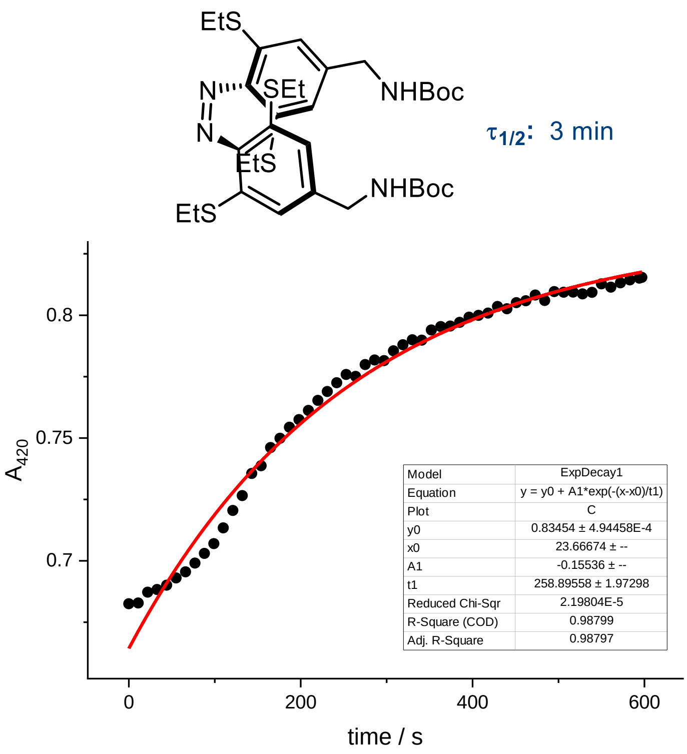

Figure S339. Lifetime plot of (*Z*)-**(SEt)$_4$-NHBoc** based on following UV-Vis absorbance over time after irradiation with 590 nm light (rt, DMSO)

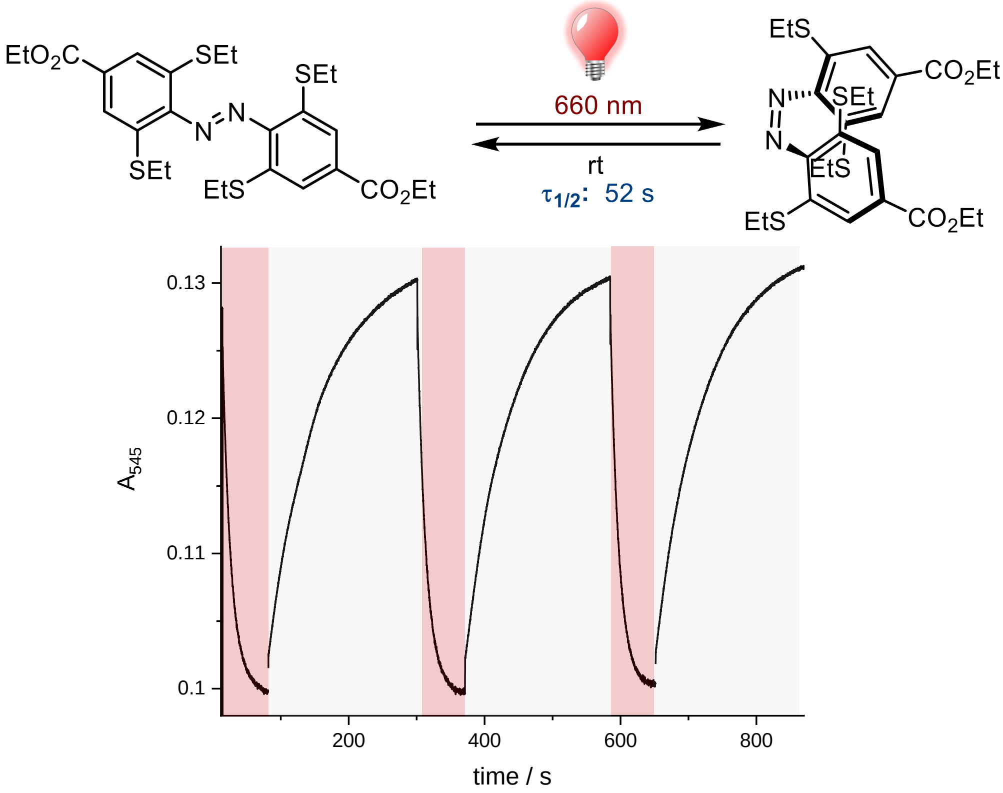

Figure S340. Reversible of switching of **(SEt)$_4$-ester** over time after irradiation with 660 nm light (rt, DMSO).

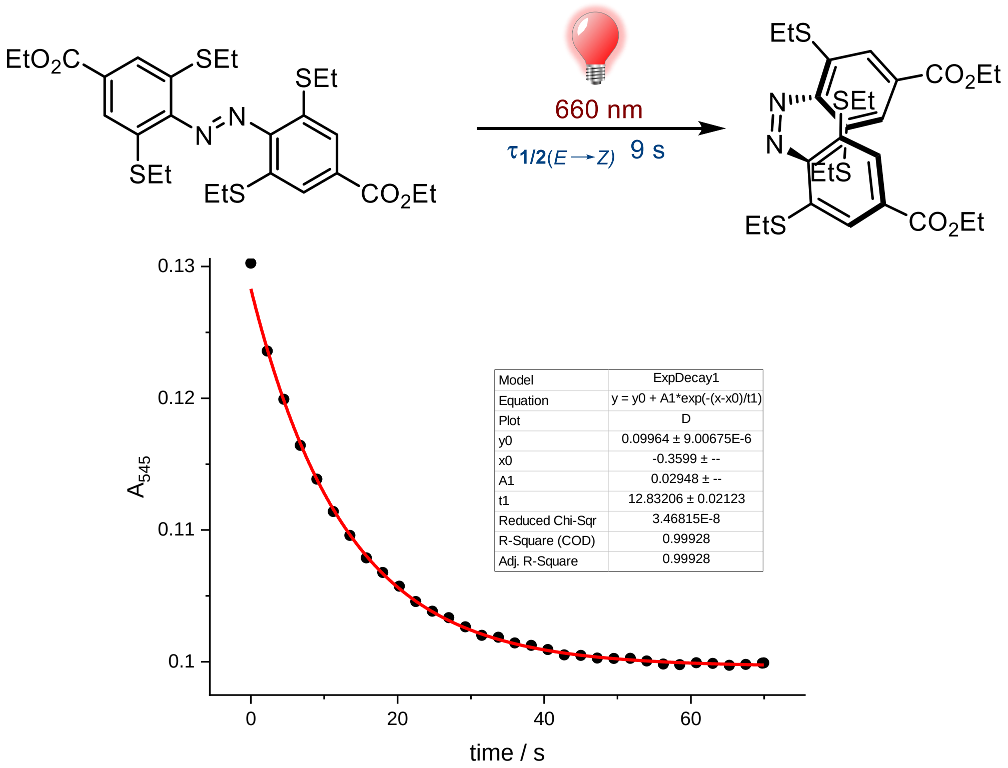

Figure S341. E → Z isomerisation of (*E*)-**(SEt)₄-ester** under constant irradiation of 660 nm light

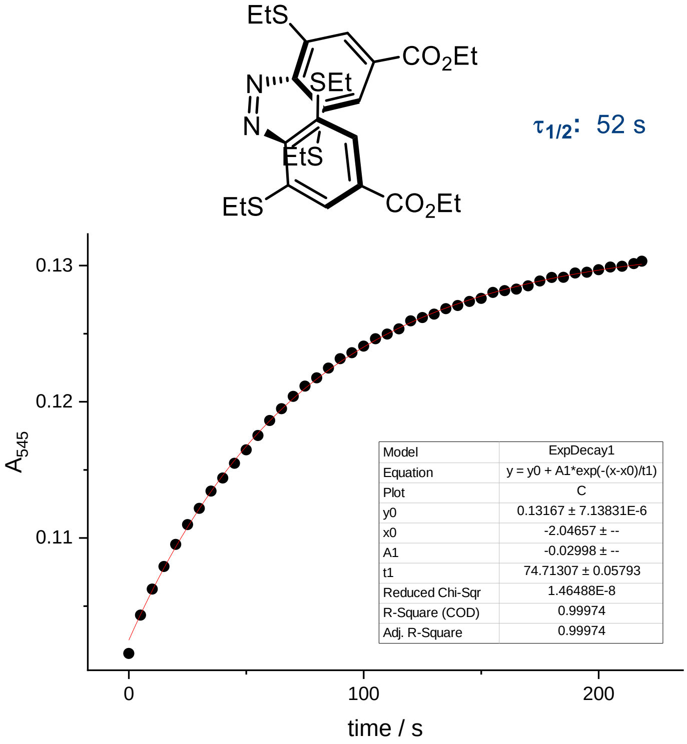

Figure S342. Lifetime plot of (*Z*)-**(SEt)₄-ester** based on following UV-Vis absorbance over time after irradiation with 660 nm light (rt, DMSO**)**

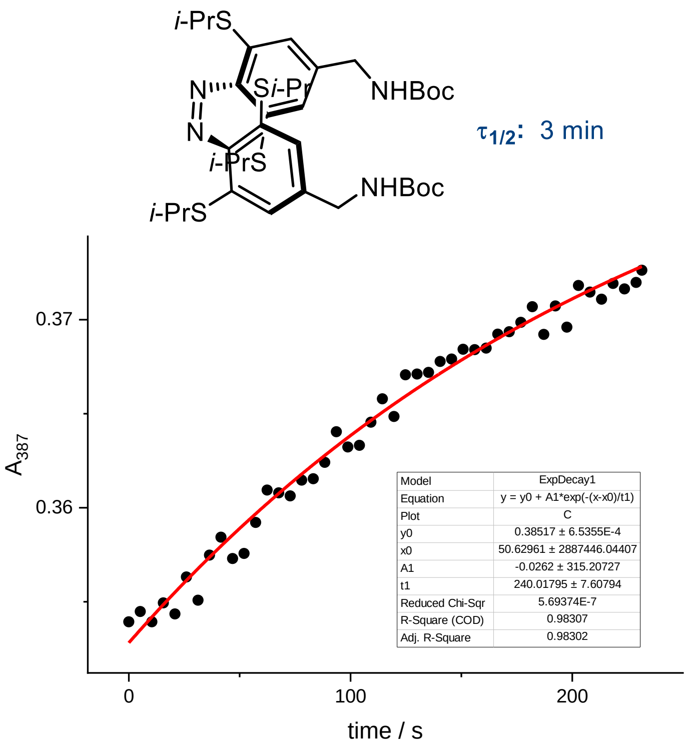

Figure S343. Lifetime plot of (Z)-**(S^{i}Pr)$_4$-NHBoc** based on following UV-Vis absorbance over time after irradiation with 590 nm light (rt, DMSO)

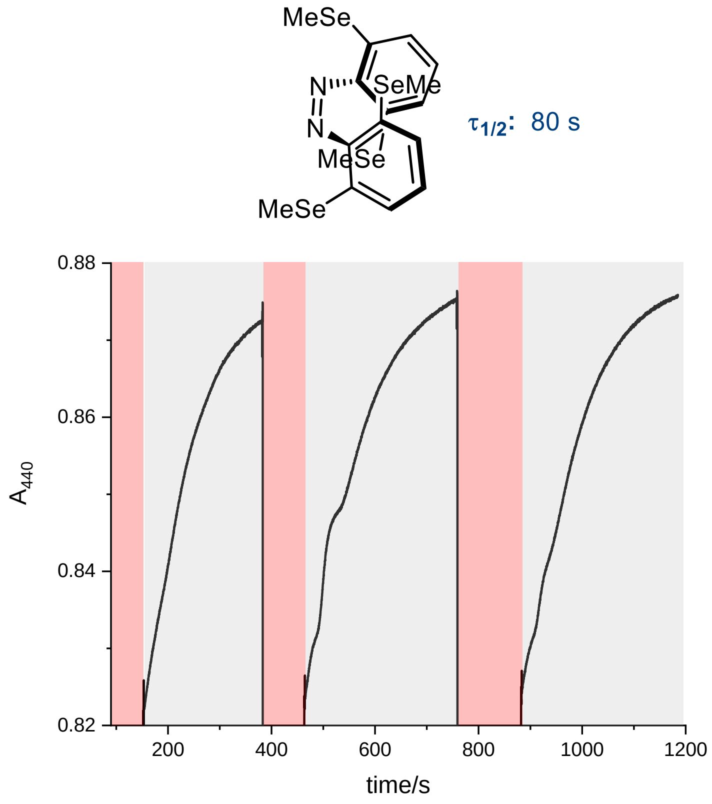

Figure S344. Reversible of switching of **(SeMe)$_4$-H** over time after irradiation with 625 nm light (rt, DMSO).

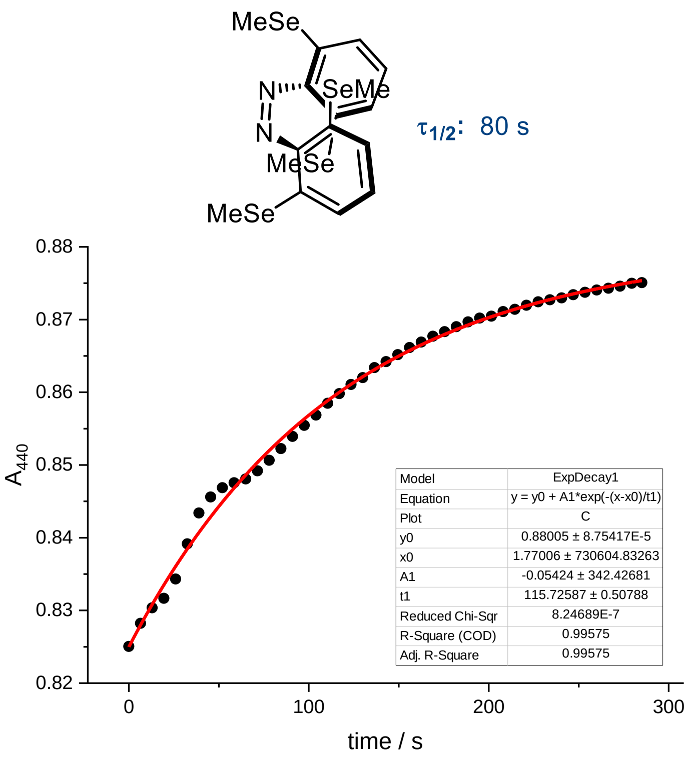

Figure S345. Lifetime plot of (*Z*)-**(SeMe)$_4$-H** based on following UV-Vis absorbance over time after irradiation with 625 nm light (rt, DMSO)

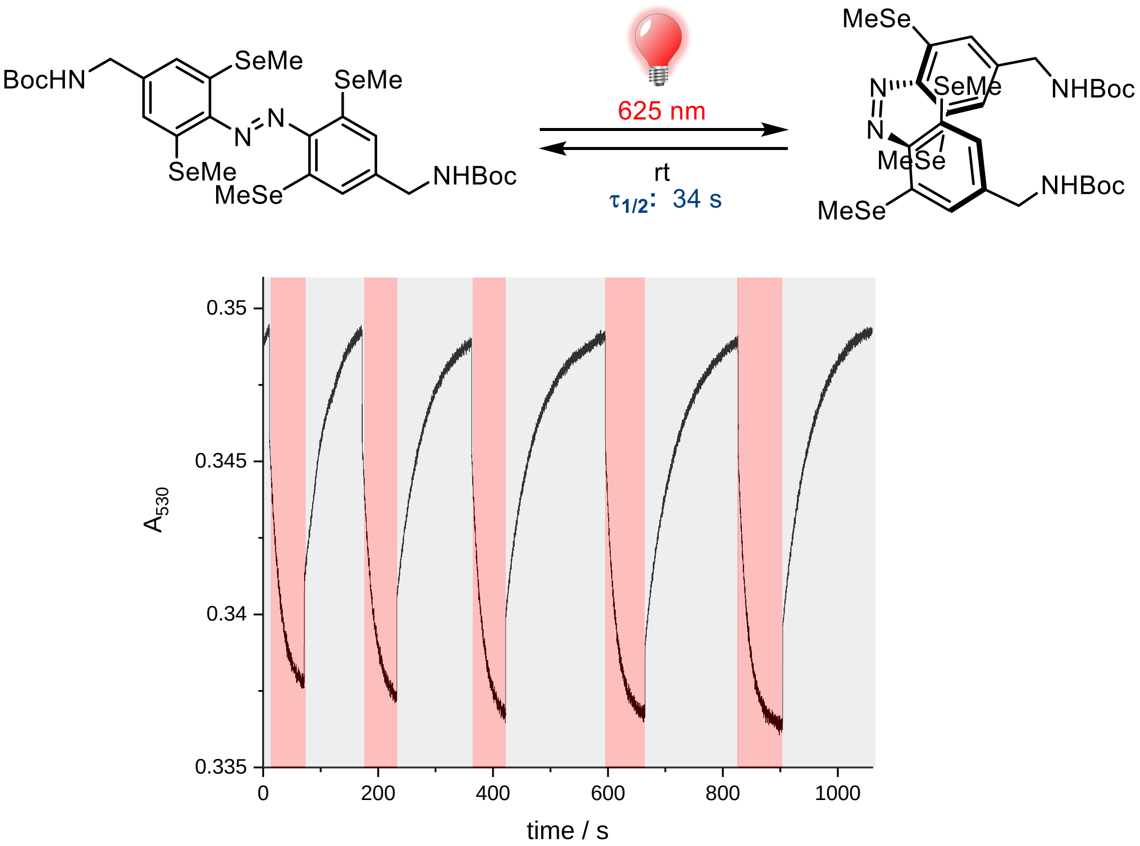

Figure S346. Reversible of switching of **(SeMe)$_4$-NHBoc** over time after irradiation with 625 nm light (rt, DMSO).

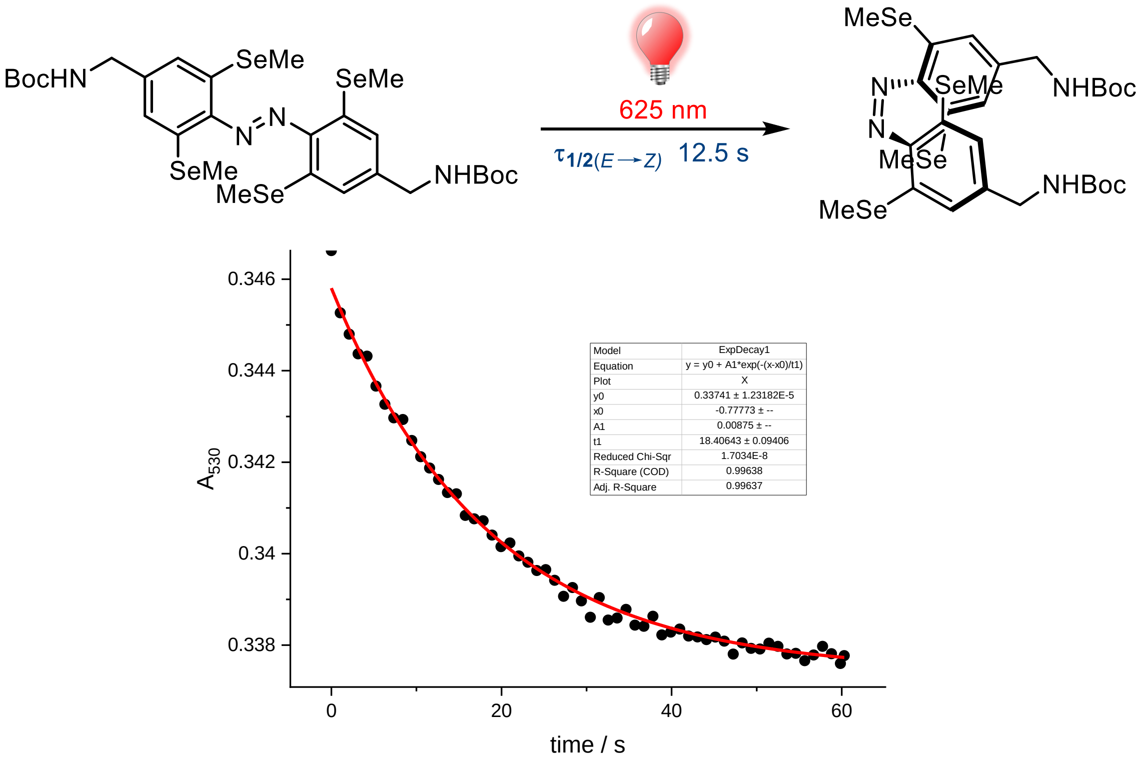

Figure S347. E → Z isomerisation of (*E*)-**(SeMe)$_4$-NHBoc** under constant irradiation of 625 nm light

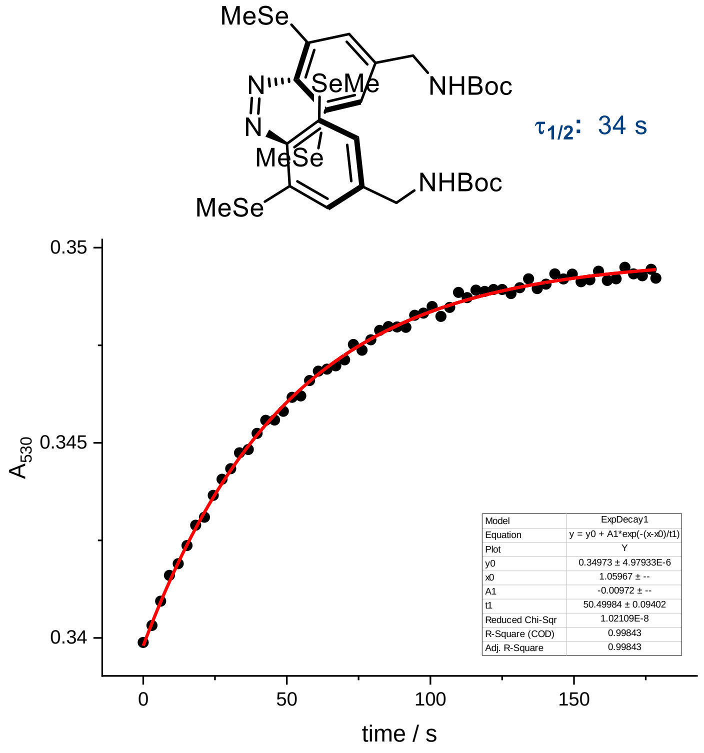

Figure S348. Lifetime plot of (*E*)-**(SeMe)$_4$-NHBoc** based on following UV-Vis absorbance over time after irradiation with 625 nm light (rt, DMSO)

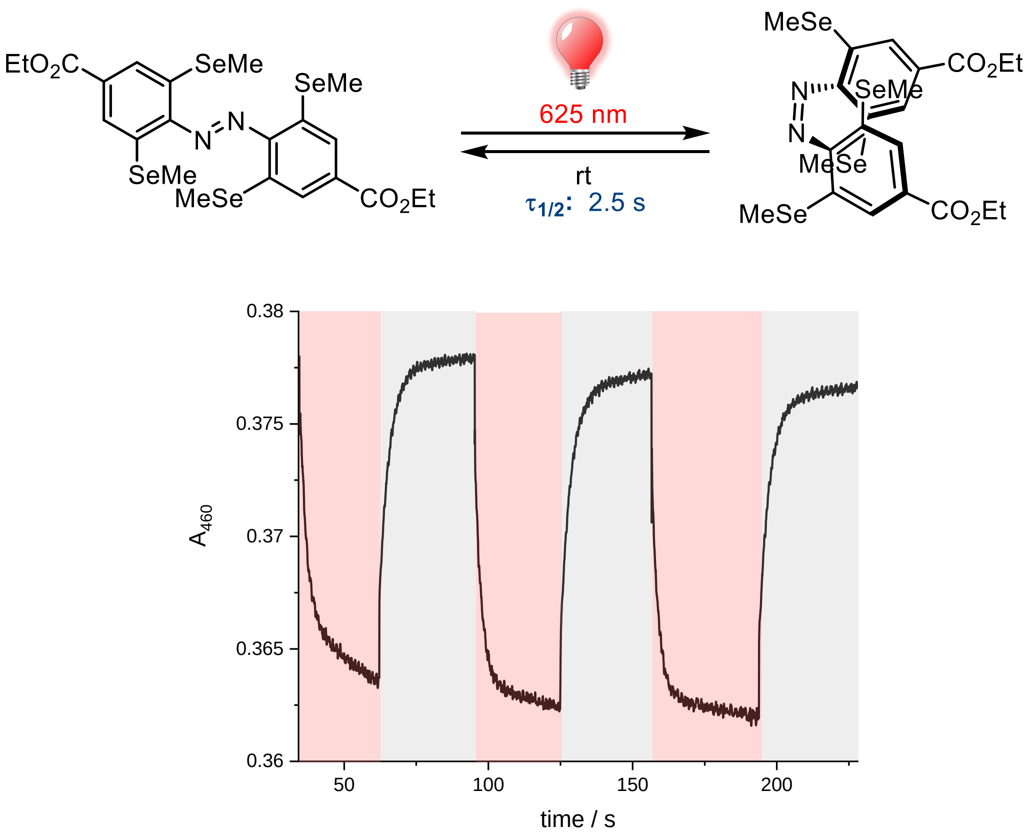

Figure S349. Reversible of switching of **(SeMe)$_4$-ester** over time after irradiation with 660 nm light (rt, DMSO).

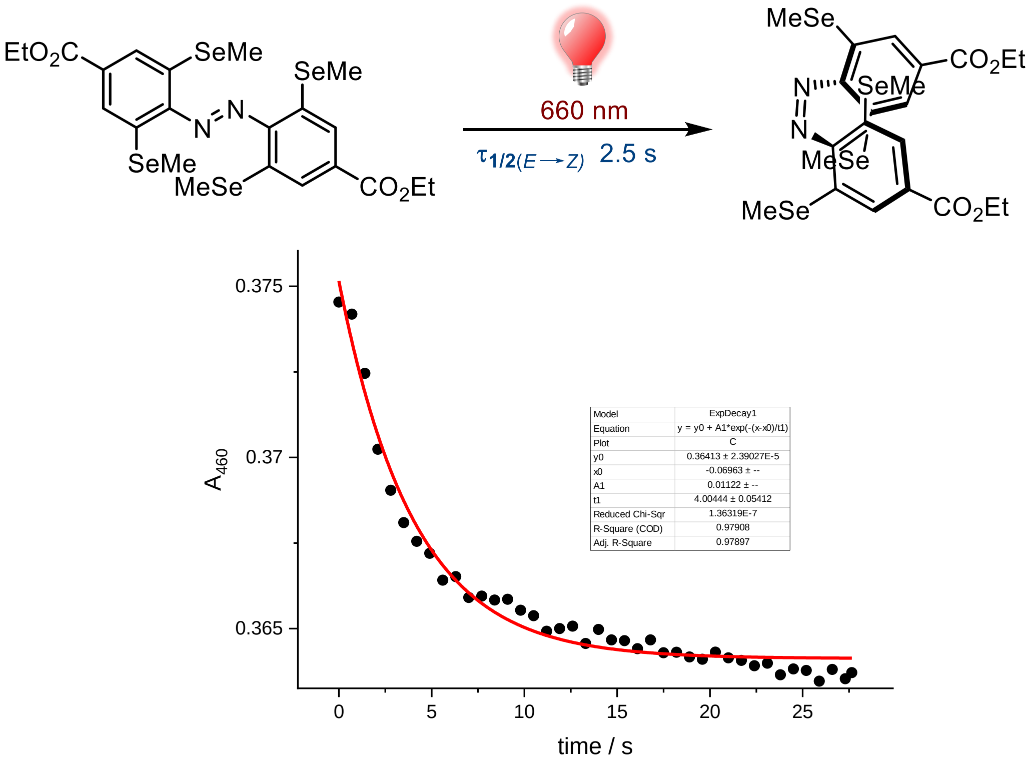

Figure S350. E → Z isomerisation of (*E*)-**(SeMe)$_4$-ester** under constant irradiation of 660 nm light

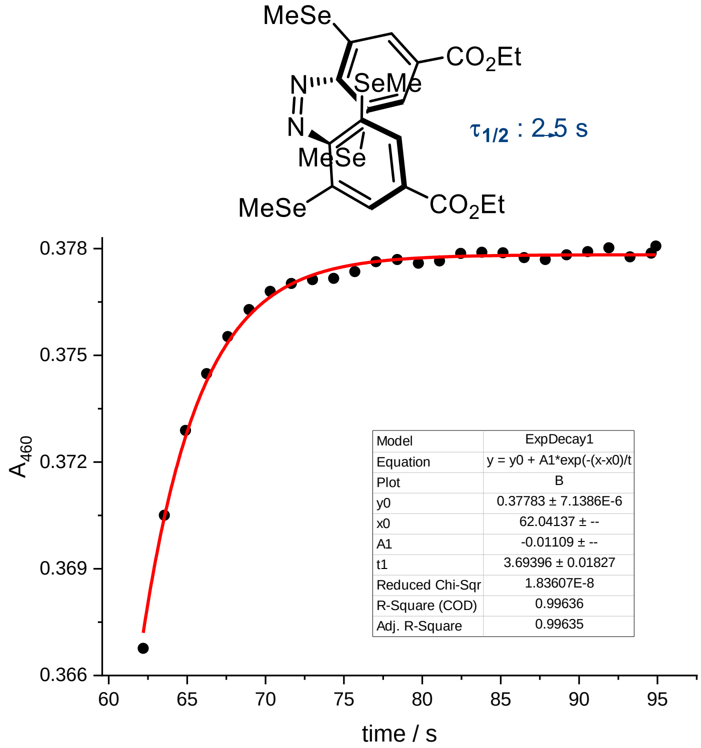

Figure S351. Lifetime plot of (*Z*)-**(SeMe)$_4$-ester** based on following UV-Vis absorbance over time after irradiation with 625 nm light (rt, DMSO)

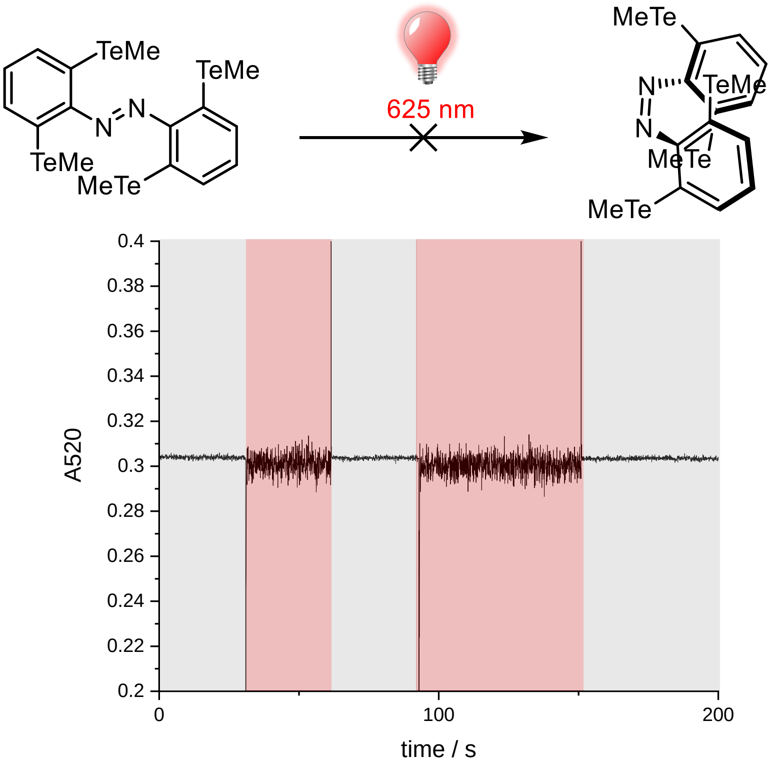

Figure S352. No switching was observed upon irradiation of (*E*)-**(TeMe)$_4$-H** with 660 nm light (rt, DMSO. (Background noise due to incoming light from LEDs during irradiation)

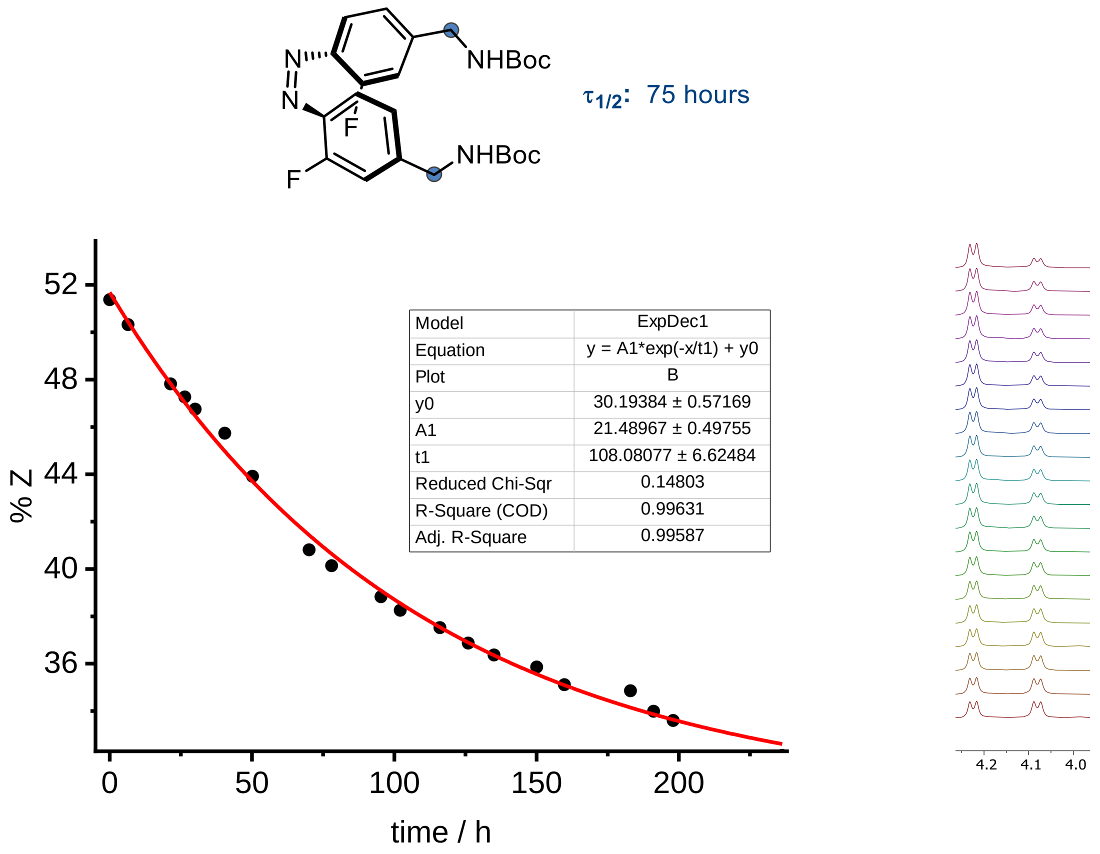

Figure S353. Lifetime plot of (*Z*)-**F$_2$,*m*-H$_2$** based on ^{1}H NMR integration of CH_2 protons over time after irradiation with 530 nm light (rt, d6-DMSO).

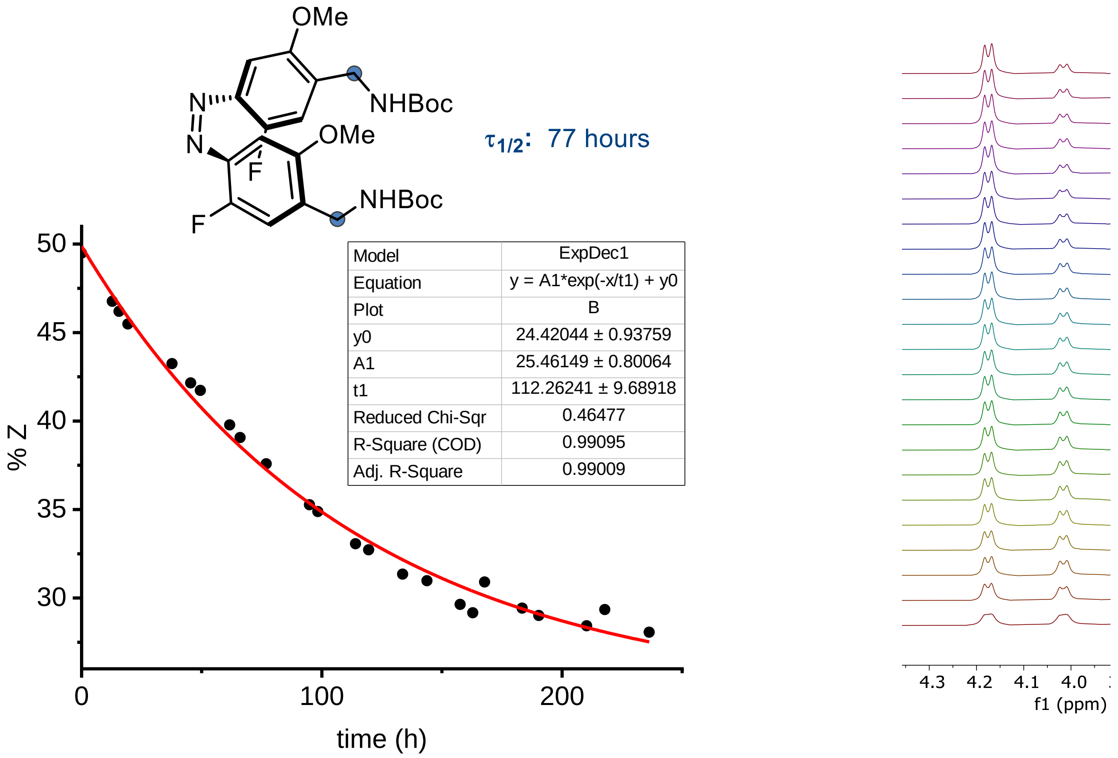

Figure S354. Lifetime plot of (*Z*)-**F$_2$,*m*-OMe$_2$** based on ^{1}H NMR integration of CH_2 protons over time after irradiation with 530 nm light (rt, d6-DMSO).

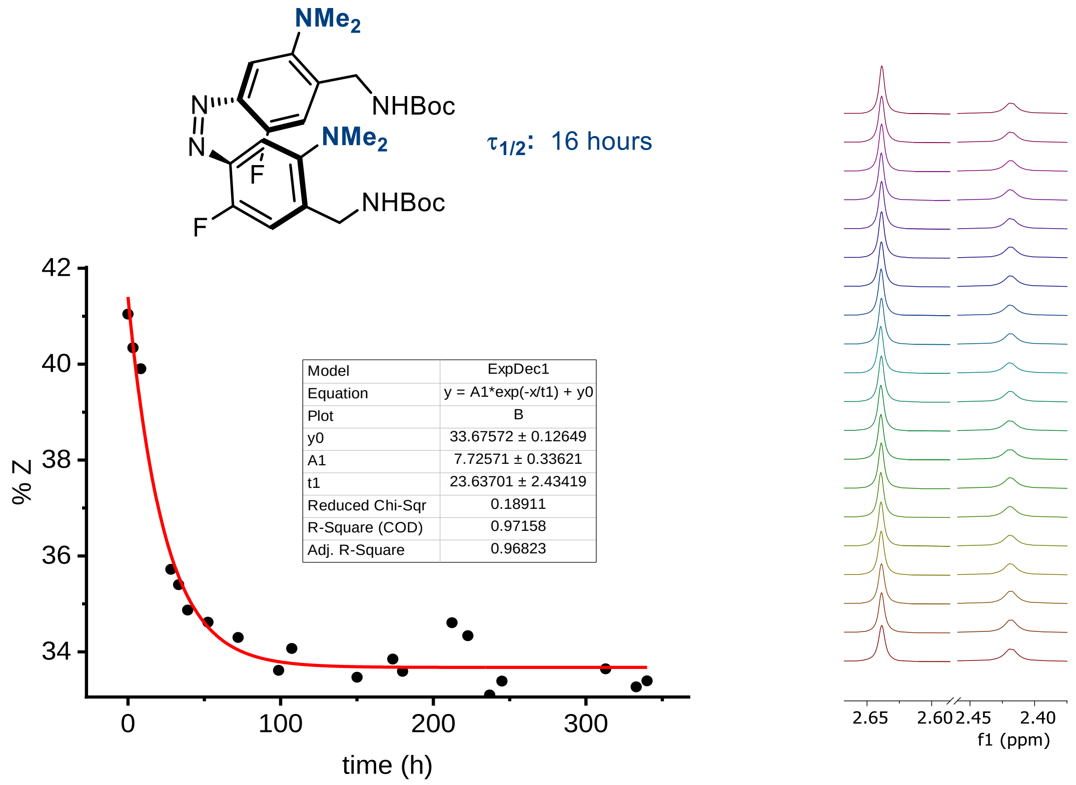

Figure S355. Lifetime plot of (*Z*)-**F$_2$,*m*-(NMe$_2$)$_2$** based on ^{1}H NMR integration of NMe$_2$ protons over time after irradiation with 530 nm light (rt, d6-DMSO).

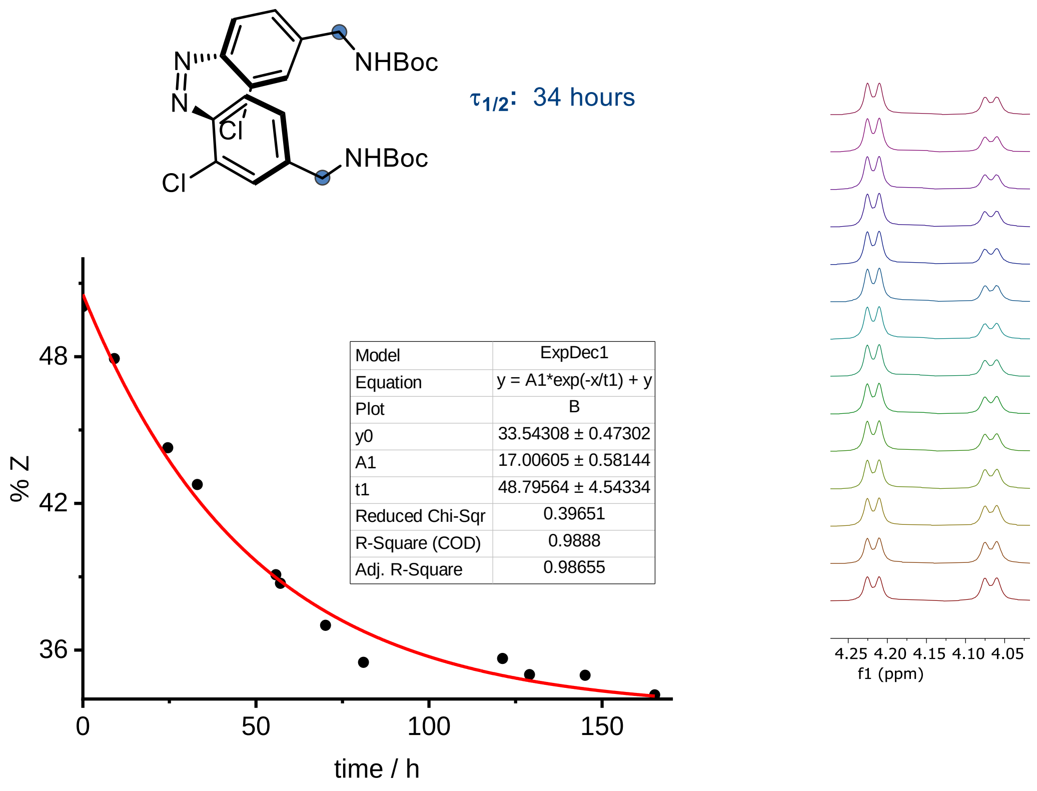

Figure S356. Lifetime plot of (*Z*)-**Cl$_2$,*m*-H$_2$** based on ^{1}H NMR integration of CH$_2$ protons over time after irradiation with 530 nm light (rt, d6-DMSO).

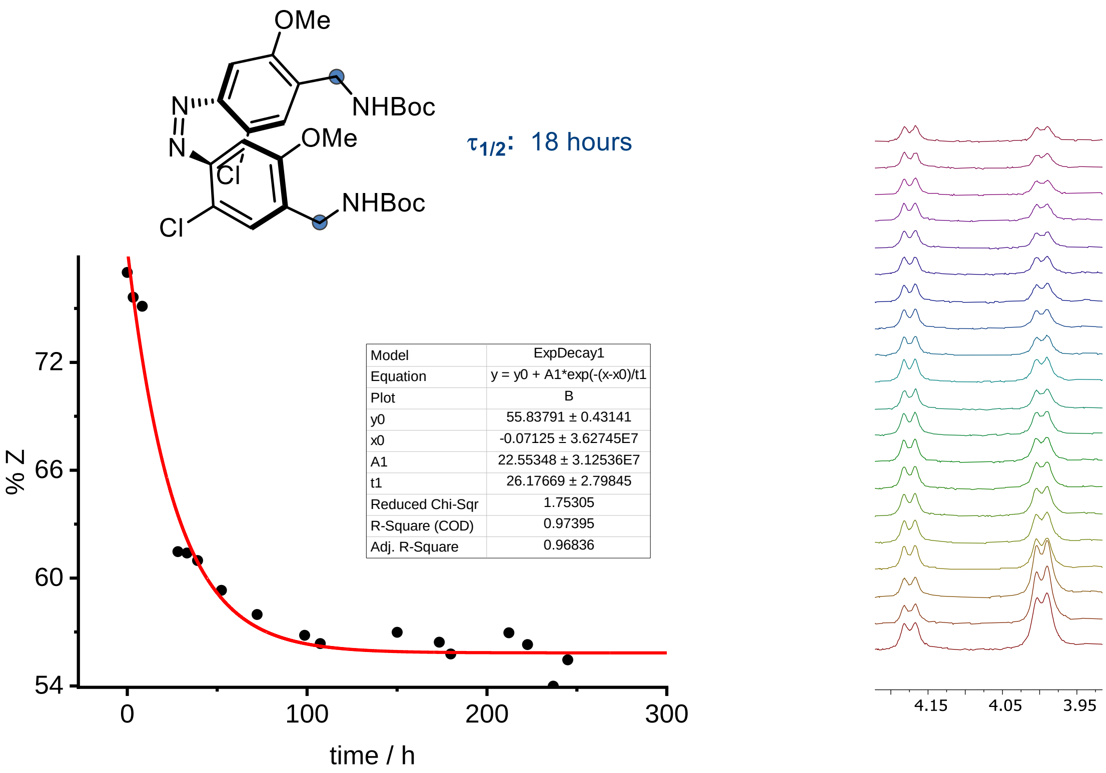

Figure S357. Lifetime plot of (*Z*)-**F$_2$,*m*-OMe$_2$** based on ^{1}H NMR integration of CH_2 protons over time after irradiation with 530 nm light (rt, d6-DMSO).

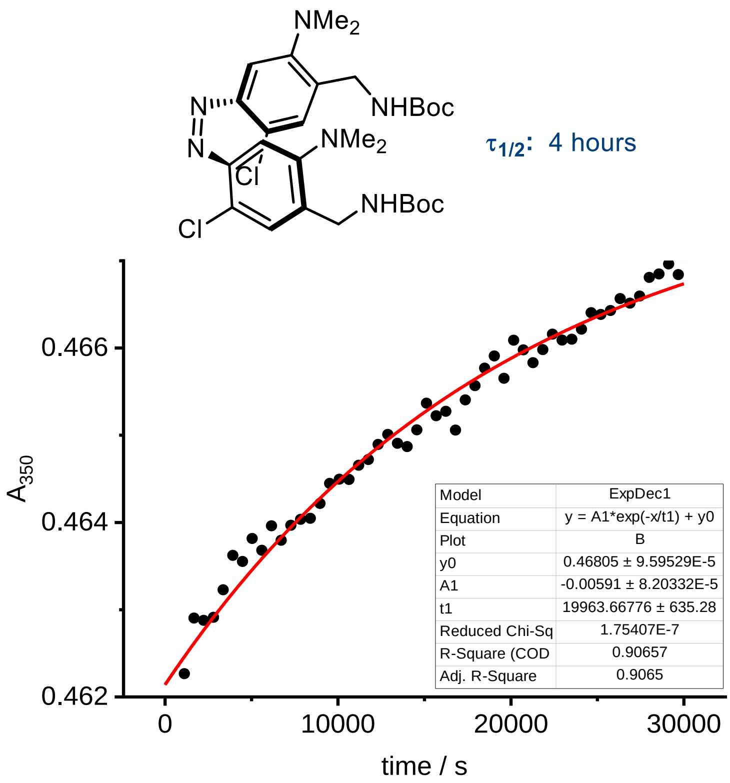

Figure S358. Lifetime plot of (*Z*)-**Cl$_2$,*m*-(NMe$_2$)$_2$** based on following UV-Vis absorbance over time after irradiation with 530 nm light (rt, DMSO).

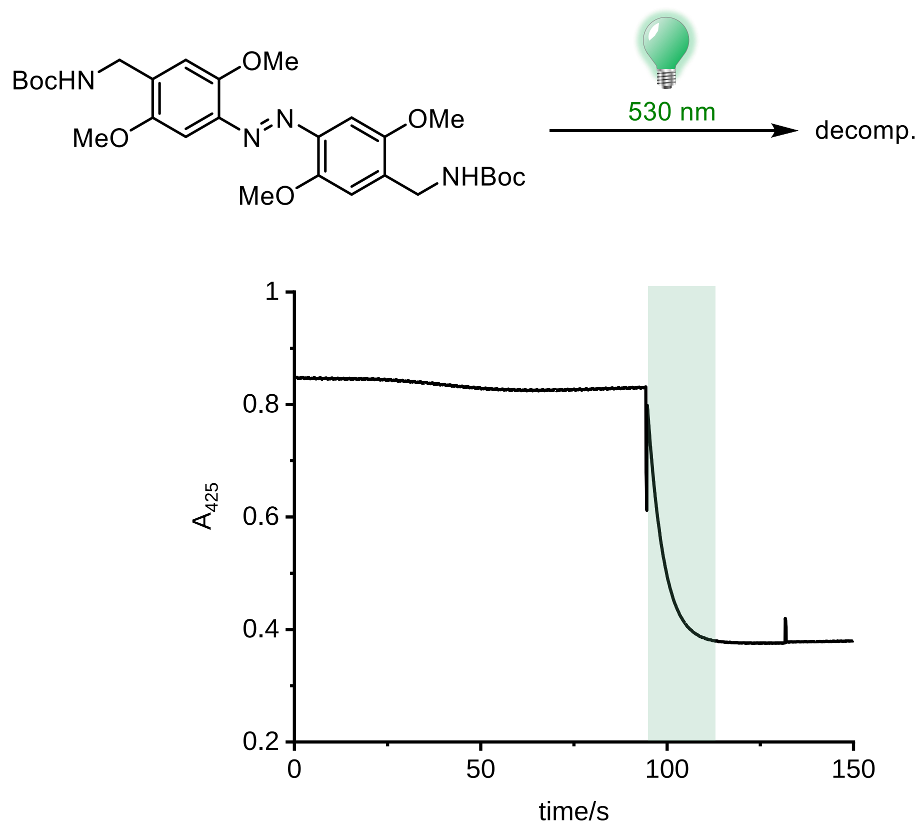

Figure S359. Decomposition of **OMe₂,*m*-OMe₂** over time during irradiation with 530 nm light (rt, DMSO).

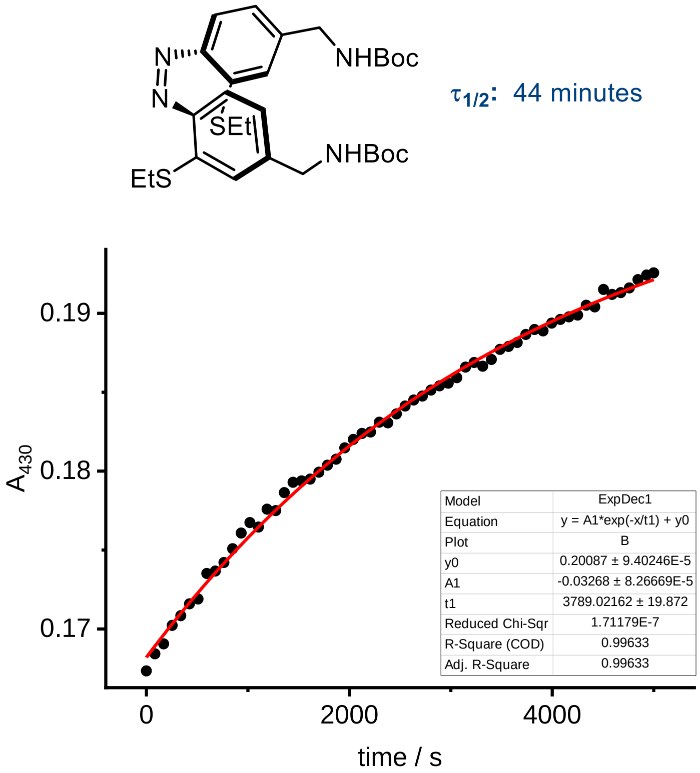

Figure S360. Lifetime plot of (*Z*)-**SEt₂,*m*-H₂** based on following UV-Vis absorbance over time after irradiation with 590 nm light (rt, DMSO).

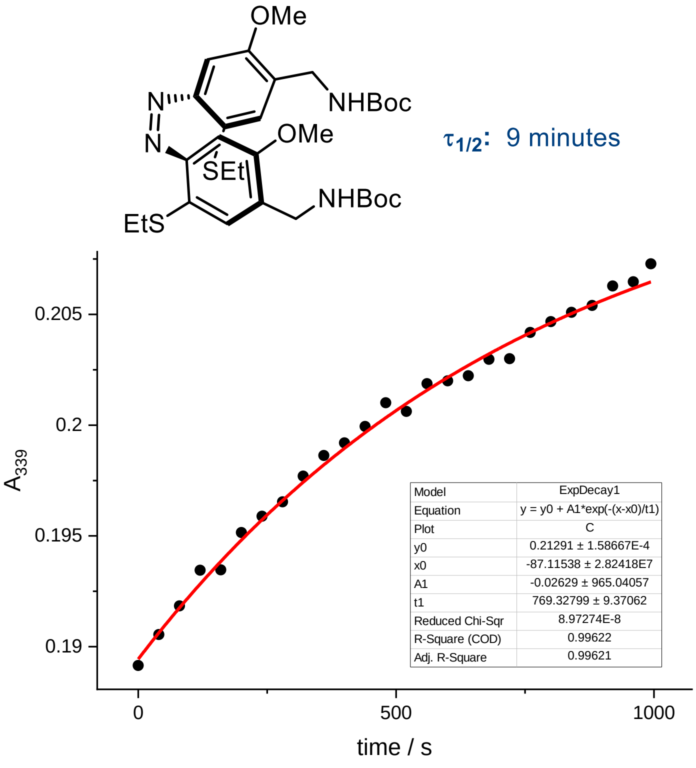

Figure S361. Lifetime plot of (*Z*)-**SEt$_2$,*m*-OMe$_2$** based on following UV-Vis absorbance over time after irradiation with 590 nm light (rt, DMSO).

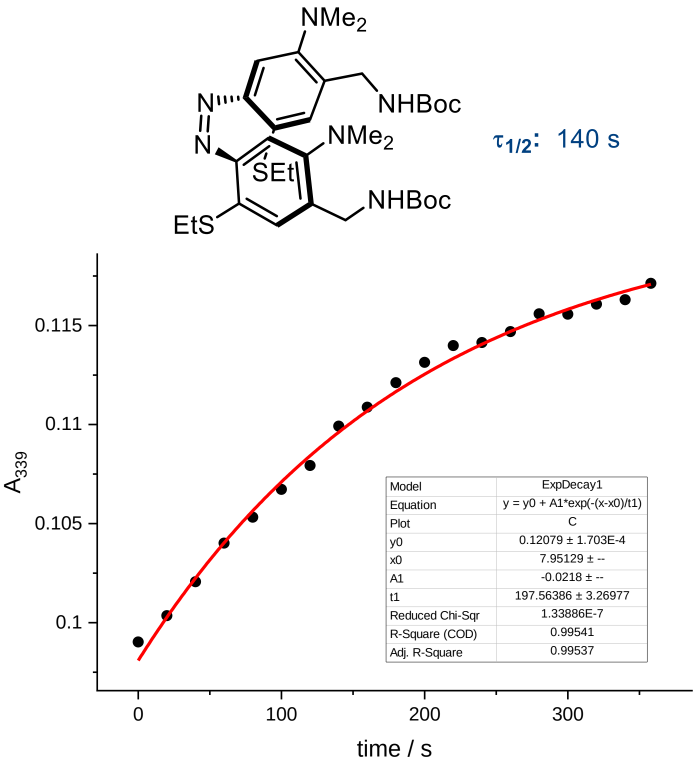

Figure S362. Lifetime plot of (*Z*)-**SEt$_2$,*m*-(NMe$_2$)$_2$**based on following UV-Vis absorbance over time after irradiation with 590 nm light (rt, DMSO).

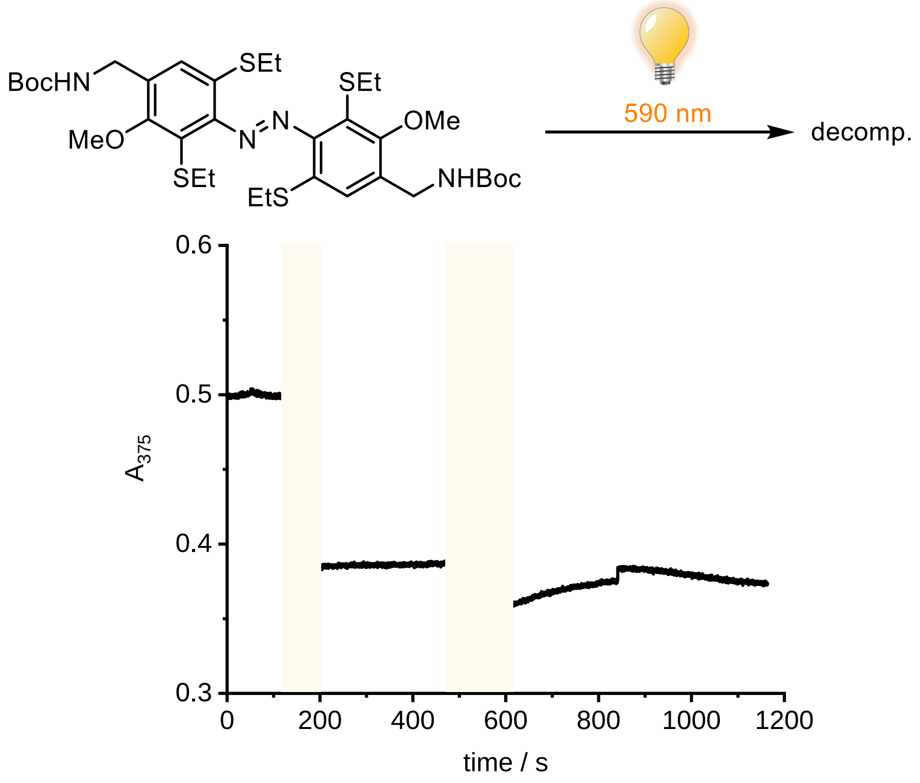

Figure S363. Decomposition of **(SEt)$_4$,*m*-OMe$_2$-NHBoc** over time during irradiation with 590 nm light (rt, DMSO).

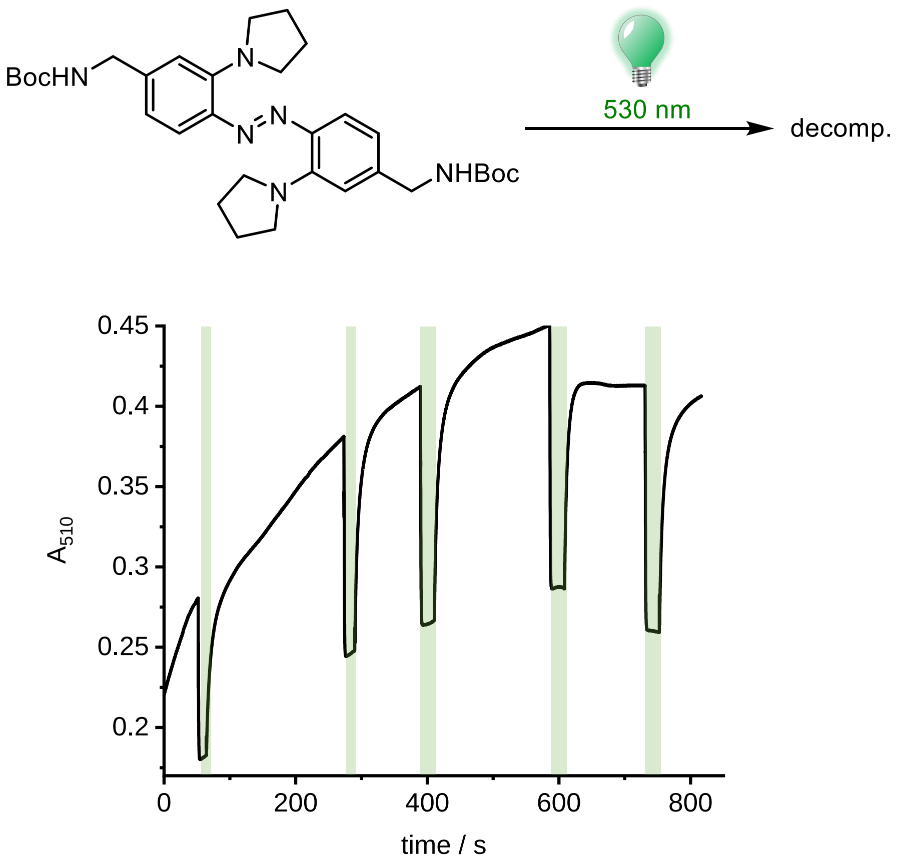

Figure S364. Decomposition of **(N(CH$_2$)$_4$)$_2$,*m*-H** over time during irradiation with 530 nm light (rt, DMSO).

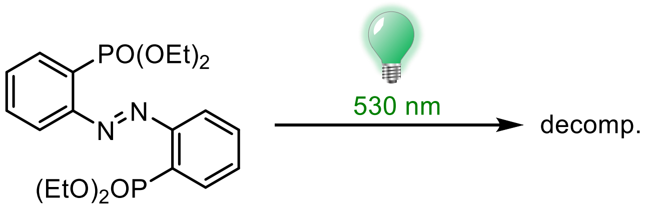

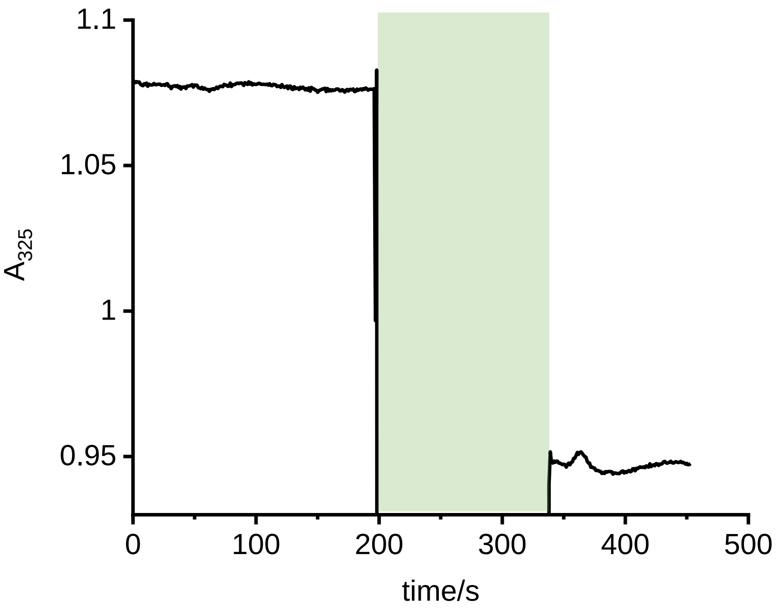

Figure S365. Decomposition of **(PO(OEt)$_2$)$_2$,*m*-H** over time during irradiation with 530 nm light (rt, DMSO).

5. GSH stability studies

A 10 mM reduced glutathione (GSH) solution was prepared in degassed 100 mM sodium phosphate buffer (at least 15 minutes of N_2 bubbled through the buffer solution prior to addition to GSH). The absorption of the solution was measured periodically at 25°C, then fitted to a 1st Order exponential decay.

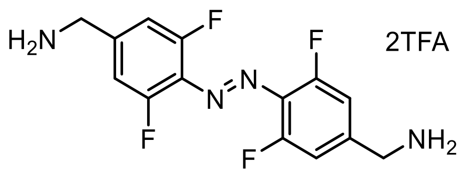

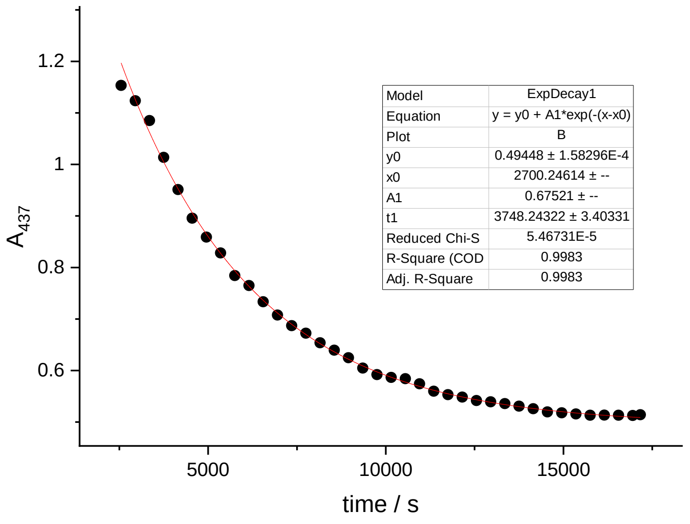

Figure S366. Decomposition of **F_4-CH_2NH_2•TFA** Conditions: 100 mM phosphate buffer containing 10 mM GSH degassed with N_2. T = 298 K.

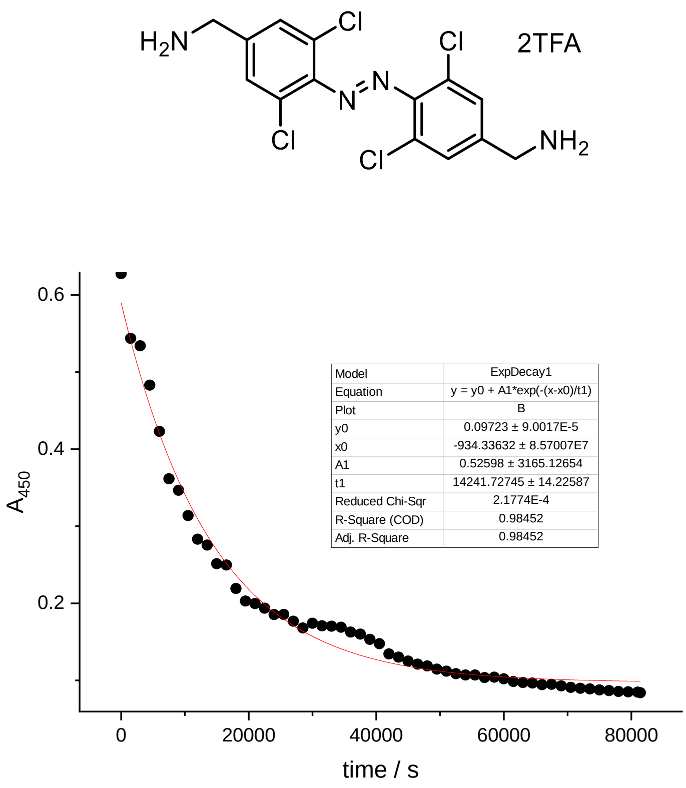

Figure S367. Decomposition of **Cl_4-CH_2NH_2•TFA.** Conditions: 100 mM phosphate buffer containing 10 mM GSH degassed with N_2. T = 298 K.

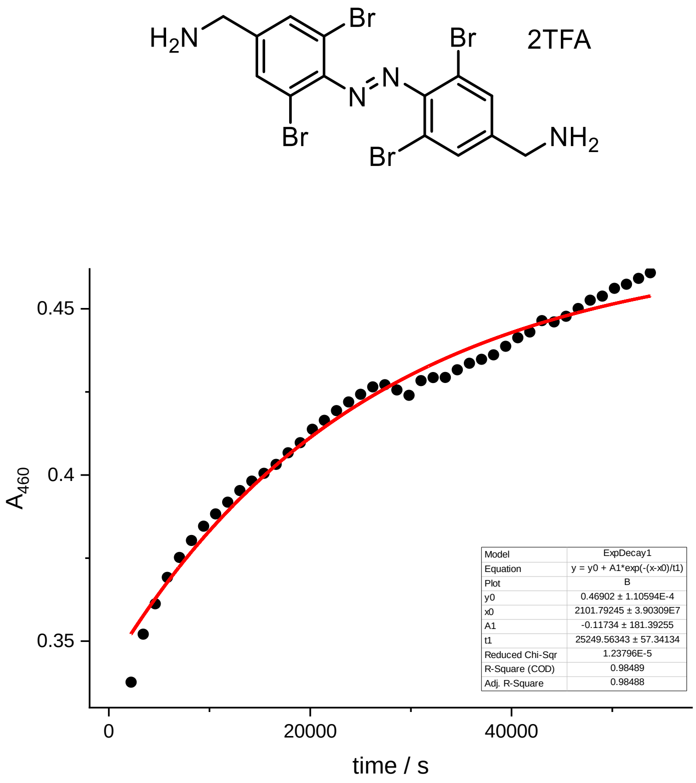

Figure S368. Decomposition of **Br_4-CH_2NH_2•TFA.** Conditions: 100 mM phosphate buffer containing 10 mM GSH degassed with N_2. T = 298 K.

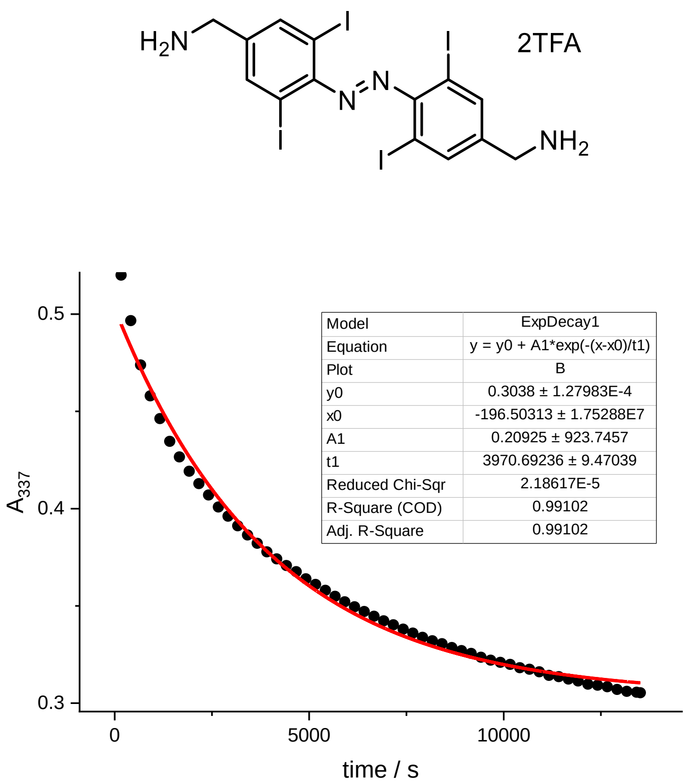

Figure S369. Decomposition of **I_4-CH_2NH_2•TFA.** Conditions: 100 mM phosphate buffer containing 10 mM GSH degassed with N_2. T = 298 K.

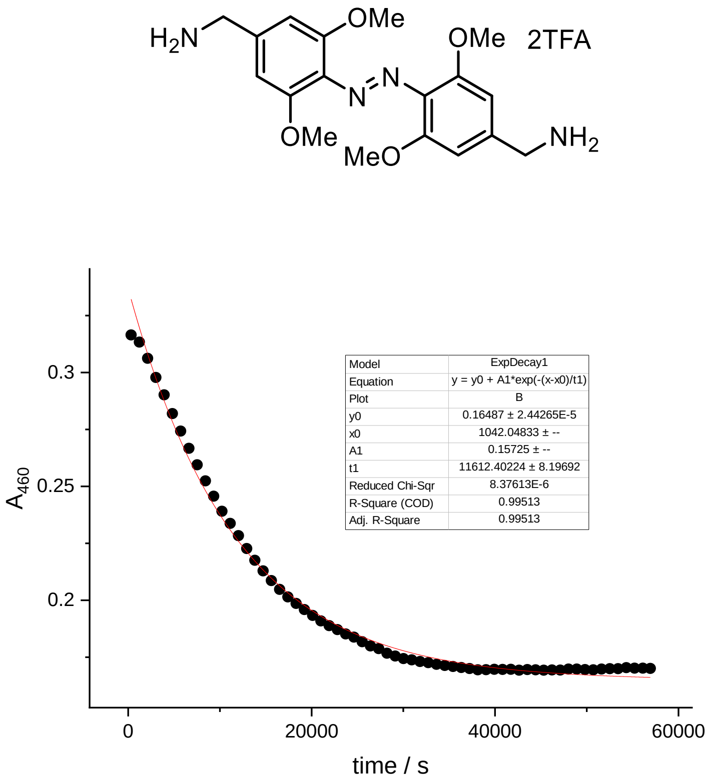

Figure S370. Decomposition of **$(OMe)_4$-CH_2NH_2•TFA.** Conditions: 100 mM phosphate buffer containing 10 mM GSH degassed with N_2. T = 298 K.

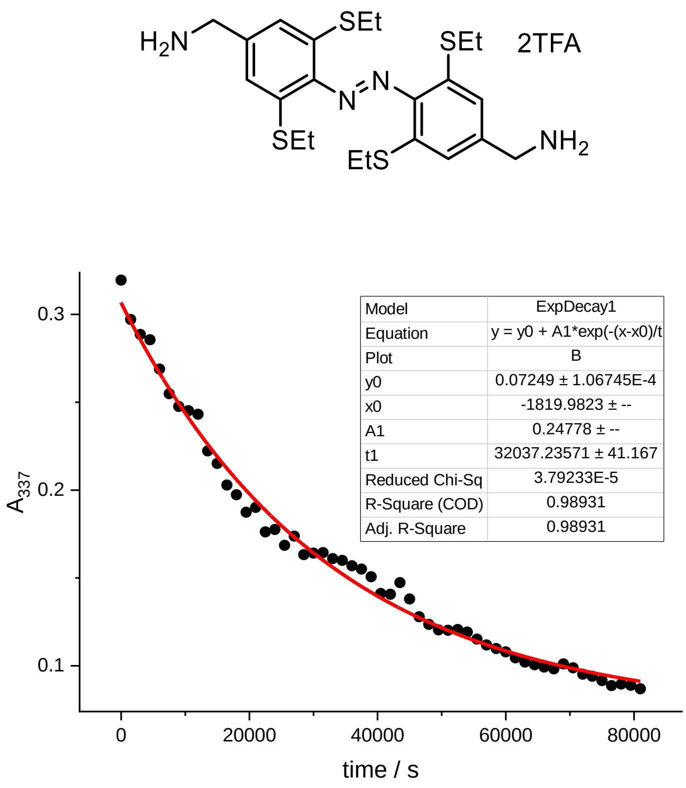

Figure S371. Decomposition of **(SEt)$_4$-CH$_2$NH$_2$•TFA.** Conditions: 100 mM phosphate buffer containing 10 mM GSH degassed with N_2. T = 298 K.

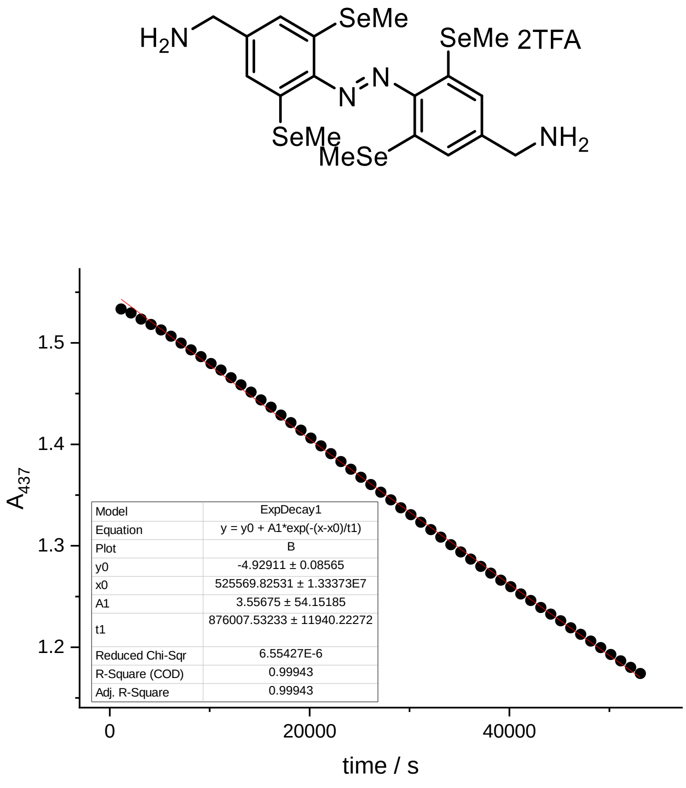

Figure S372. Decomposition of **(SeMe)$_4$-CH$_2$NH$_2$•TFA.** Conditions: 100 mM phosphate buffer containing 10 mM GSH degassed with N_2. T = 298 K.

6. Single crystal X-ray diffraction experiments

Crystals were grown by vapour diffusion. Compounds (2-15 mg) were dissolved in minimal amounts of $CDCl_3$, then filtered over cotton into a small vial. The vial was capped and punctured with a small needle, then placed into a larger vial containing pentane or hexane. The vials were stored in the fridge until suitable crystals formed.

Crystal Structure Determination Deposition Number(s) 2196083-92 contains the supplementary crystallographic data for this paper. These data are provided free of charge by the joint Cambridge Crystallographic Data Centre and Fachinformationszentrum Karlsruhe Access Structures service www.ccdc.cam.ac.uk/structures."

Single-crystal X-ray diffraction data were collected at 150 K on an Oxford Diffraction(Rigaku) SuperNovae or a Rigaku Synergy-DW diffractometer (EP/V028995/1). In all cases, Cu-Kα (λ = 1.54184 Å) radiation was used and the instrument was equipped with a nitrogen gas Oxford Cryosystems Cryostream unit[6]. A suitable crystal was chosen and mounted on a 200 µm MiTeGen loop using perfluoropolyether oil. The CrysAlisPro[7] software was used for data collection and integration. All structures were solved using SuperFlip[8] and refined using full-matrix least-squares refinement within the CRYSTALS[9] suite. All non-hydrogen atoms were refined anisotropically. The hydrogen atoms were positioned at geometrically sensible positions and refined using a riding model.

Substituent 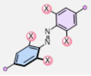	Dihedral (º) 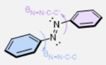		Average N=N-C-C' Angle (º) (normalised)	Coplanarity (º) 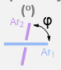	Distance (A) 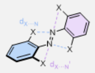		Distance (A) (Average) N···X		N=N (A)	N=N (º)	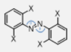	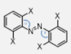	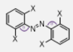	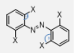	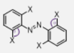
F, ∘ = H	40.7	40.7	40.7	0	2.760, 2.761	2.747, 2.759	2.761	**2.753**	1.229	179.2	124.6, 125.3	120,120	120,120	120,120	120,120
*F, ∘ = Br	3.2	3.2	3.2	0	2.655, 2.655	2.635, 2.635	2.655	**2.635**	1.253	180.0	115.2, 115.2	128.8,128.8	116.3,116.3	119.5,119.5	117.4,117.5
Cl, ∘ = H	79.7	35.2	57.45	44.5	3.229, 2.930	2.990, 2.880	3.080	**2.935**	1.182	180.0	112.9,117.8	120.8,126.7	120.8,115.7	119.9,121.9	119.4,119.1
	126.9	59.3	56.2	67.6	2.962, 3.815	2.916, 3.092	3.389	**3.004**	1.248	176.2	113.6,112.1	124.1, 123.6	118.1,118.0	120.9,120.4	118.9,119.0
	135.0	61.9	53.45	73.1	3.725, 3.096	2.956, 2.928	3.411	**2.942**	1.245	178.0	113.0,112.1	117.9,117.6	124.2,124.3	119.3,118.9	120.7,120.3
Br, ∘ = CH_2NHBoc	45.8	-134.2	45.8	-88.4	3.031, 3.022	3.030, 3.023	3.027	3.027	1.252	175.6	113.8,113.8	125.2,125.2	117.9,117.9	121.8,121.8	120,120
I, ∘ = CH_2N_3	48.6	-135.5	46.55	-86.7	3.170, 3.121	3.183, 3.203	**3.146**	3.193	1.260	174.2	111.6,112.6	123.3,123.0	116.1,116.6	124.6,125.5	122.1,122.5
	50.9	-129.4	50.75	-78.5	3.177, 3.254	3.188, 3.078	3.216	**3.133**	1.215	179.5	112.8,116.2	126.5,123.5	115.4,118.4	123.5,122.4	118.8,120.7
I, ∘= CO_2Et	50.3	-48.8	49.6	-80.9	3.389, 3.439	3.152, 3.222	3.414	**3.187**	1.249	171.6	114.9,112.9	123.9,123.3	116.6,118.4	124.6,123.1	120.5,118.5
OMe, ∘ = Br	29.6	-142	33.8	-67.6	2.584, 2.622	2.678, 2.714	**2.603**	2.696	1.252	179.4	116.5,113.6	127.8,125.3	113.8,115.7	117.0,117.6	115.6,115.9
SMe, ∘ = H	22.4	-150.8	**25.8**	51.8	2.653, 2.686	2.811, 2.814	**2.670**	2.812	**1.268**	**169.9**	115.0,114.1	124.6,124.0	114.6, 115.2	120.8,120.9	116.5,116.2
	152.3	159.5	**24.1**	48.2	2.721, 2.659	2.777, 2.821	**2.690**	2.836	**1.266**	**169.7**	113.5,116.1	124.2,125.9	115.4,113.3	120.8,121.1	115.8,116.2
SEt, ∘ = CO_2Et	1.0	-1.6	**1.3**	0.3	2.666, 2.666	2.813, 2.813	**2.666**	2.813	**1.275**	**180.0**	116.2, 116.2	126.6,126.6	114.0, 114.0	120.2,120.2	117.1,117.1
SeMe, ∘ = H	19.5	159.5	**20.0**	47.0	2.739, 2.710	2.885, 2.894	**2.725**	2.890	**1.265**	**170.5**	114.0,115.8	124.4,125.7	115.4,114.1	120.8,121.0	116.1,116.6
	158.0	152.9	**24.55**	49.1	2.670, 2.705	2.917, 2.911	**2.688**	2.914	**1.266**	**169.9**	115.2,114.2	124.2,124.2	115.4,115.7	120.7,120.3	116.8,116.5
SeMe ∘ = ester	-21.4	160.2	20.6	41.2	2.702, 2.703	2.927, 2.927	2.702	2.927	1.263	175.4	115.1,115.1	125.3,125.3	114.9,114.9	120.6,120.7	117.8,117.8
	-23.2	162.3	20.5	41.0	2.728, 2.728	2.921, 2.921	2.728	2.921	1.261	174.1	115.3,115.3	125.1,125.1	114.8,114.8	120.7,120.7	117.7,117.7
TeMe ∘ H	158.1	157.3	Av: 23.37 (24.45/22.3)	44.6	2.763,2.838	3.096,3.048	2.800	3.072	1.279	174.6	115.8,114.2	124.3,124.0	114.9,115.5	121.7,122.0	119.1,117.1
	22.2	26.7		48.9 a:46.75	2.792,2.834	3.091,3.060	2.813	3.076	1.265	177.9	115.3,114.0	123.8,123.9	115.1,114.9	122.1,122.2	119.1,117.4

Table S3: Summary of key data from crystal structures

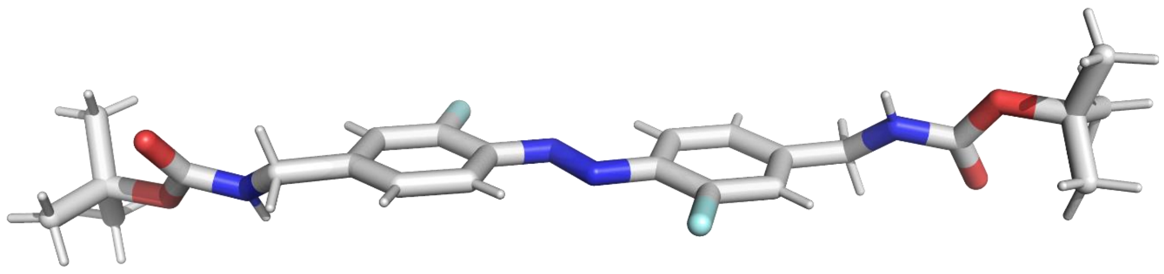

Figure S373. X-ray crystallographic structure of **F_2H_2-NHBoc**. Grey = carbon, blue = nitrogen, white = hydrogen, red = oxygen, sky blue = fluorine

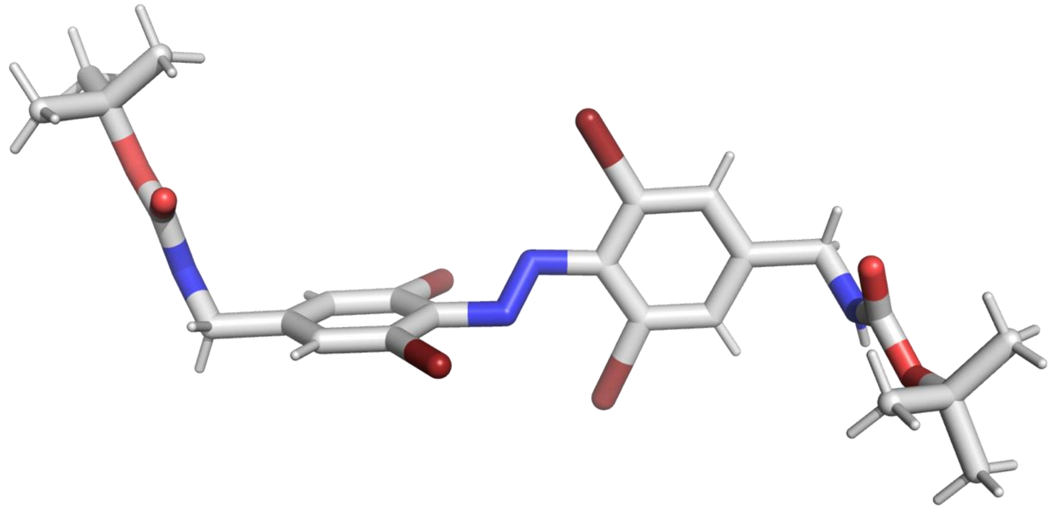

Figure S374. X-ray crystallographic structure of **Br_4-NHBoc**. Grey = carbon, blue = nitrogen, white = hydrogen, red = oxygen, dark red = bromine

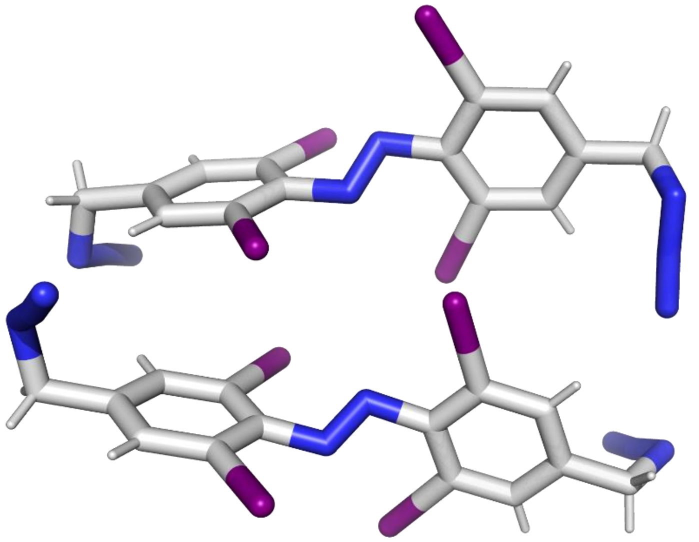

Figure S375. X-ray crystallographic structure of **I_4-N_3**. Grey = carbon, blue = nitrogen, white = hydrogen, red = oxygen, purple = iodine

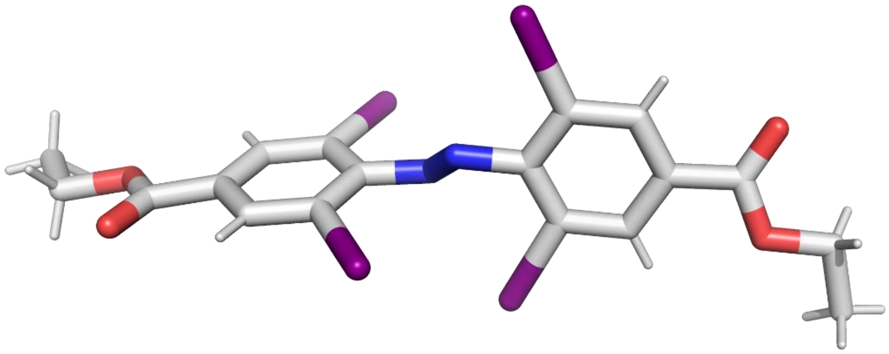

Figure S376. X-ray crystallographic structure of **I_4-Ester**. Grey = carbon, blue = nitrogen, white = hydrogen, red = oxygen, purple = iodine

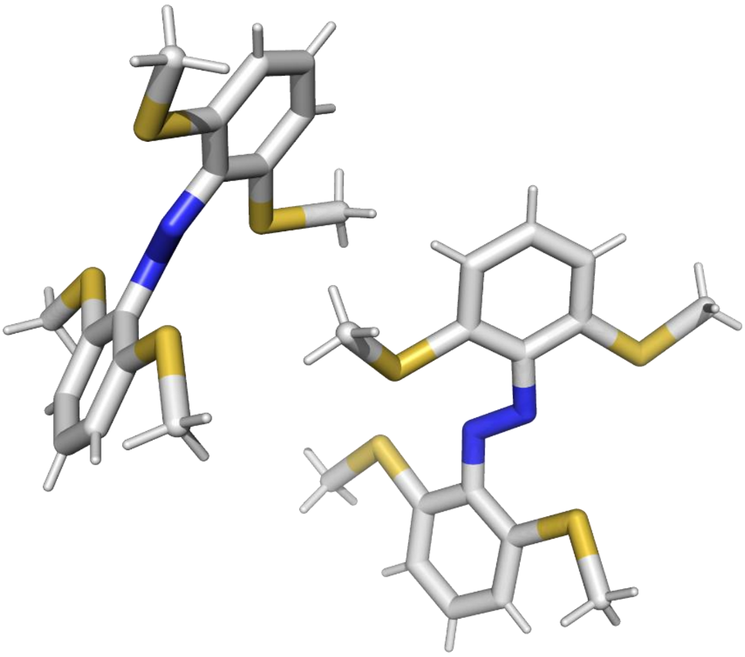

Figure S377. X-ray crystallographic structure of **(SMe_4)-H.** Grey = carbon, blue = nitrogen, white = hydrogen, yellow = sulphur

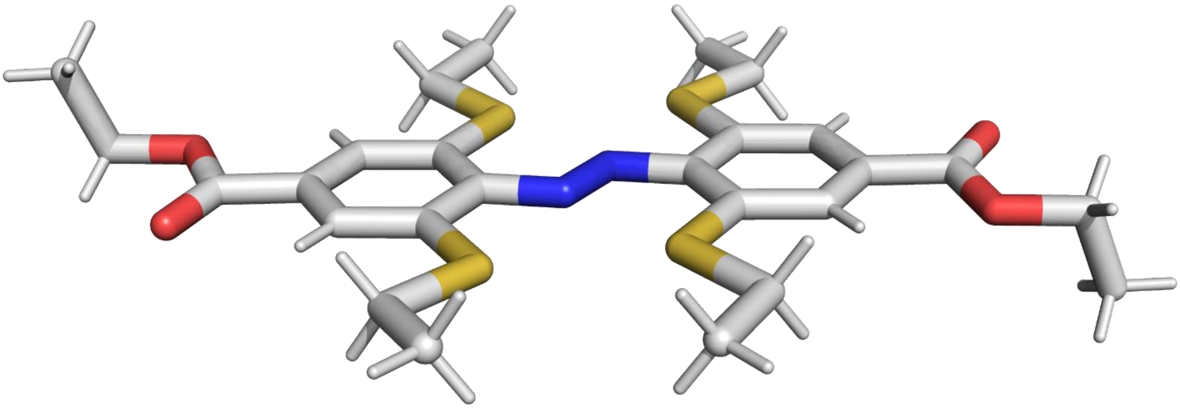

Figure S378. X-ray crystallographic structure of **(SEt_4)-CO_2Et**. Grey = carbon, blue = nitrogen, white = hydrogen, red = oxygen, yellow = sulphur

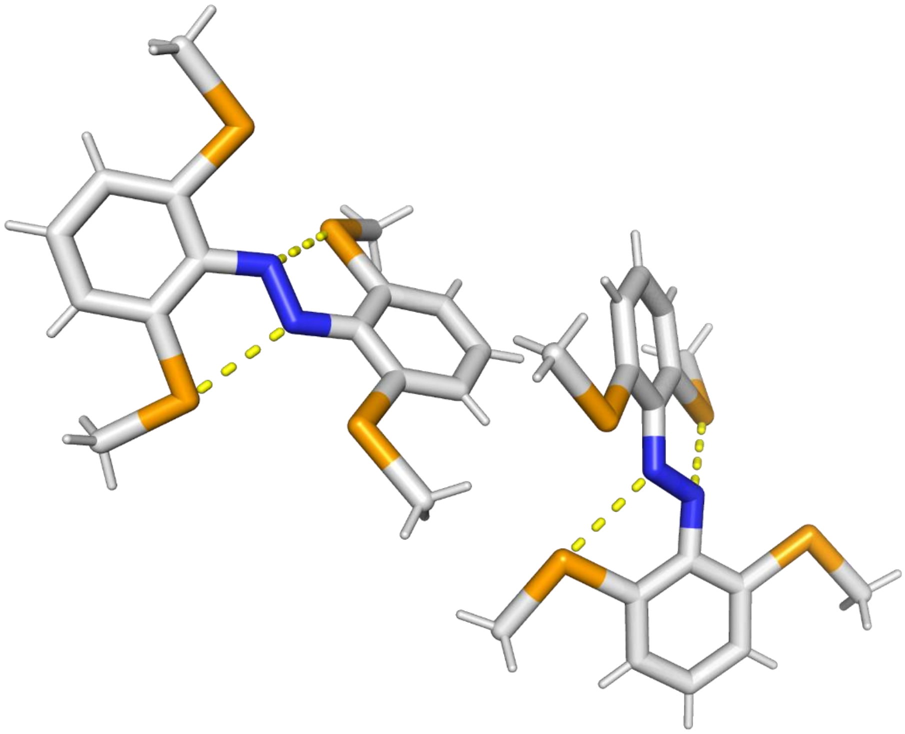

Figure S379. X-ray crystallographic structure of **(SeMe$_4$)-H**. Chalcogen bond interactions shown as dashed lines. Grey = carbon, blue = nitrogen, white = hydrogen, red = oxygen, orange = selenium

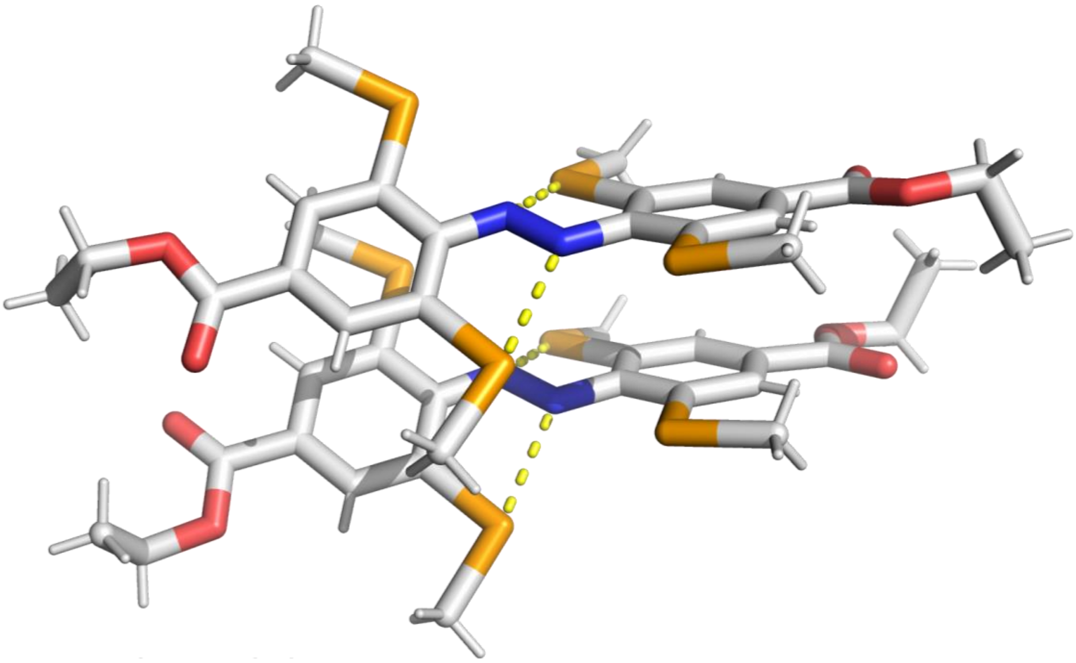

Figure S380. X-ray crystallographic structure of **(SeMe$_4$)-ester.** Chalcogen bond interactions shown as dashed lines. Grey = carbon, blue = nitrogen, white = hydrogen, red = oxygen, orange = selenium

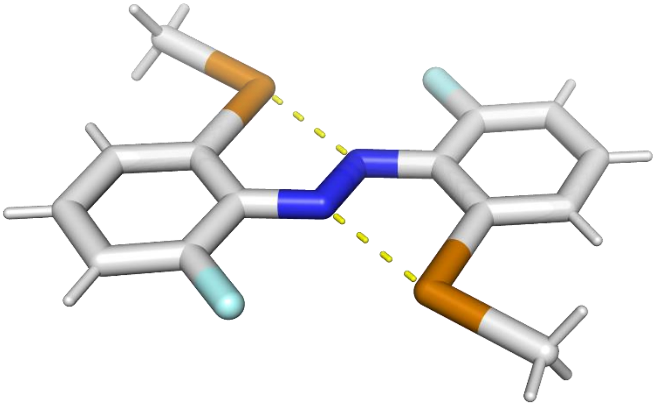

Figure S381. X-ray crystallographic structure of **Te_2H_2-H.** Chalcogen bond interactions shown as dashed lines. Grey = carbon, blue = nitrogen, white = hydrogen, red = oxygen, sky blue = Fluorine, orange = tellurium

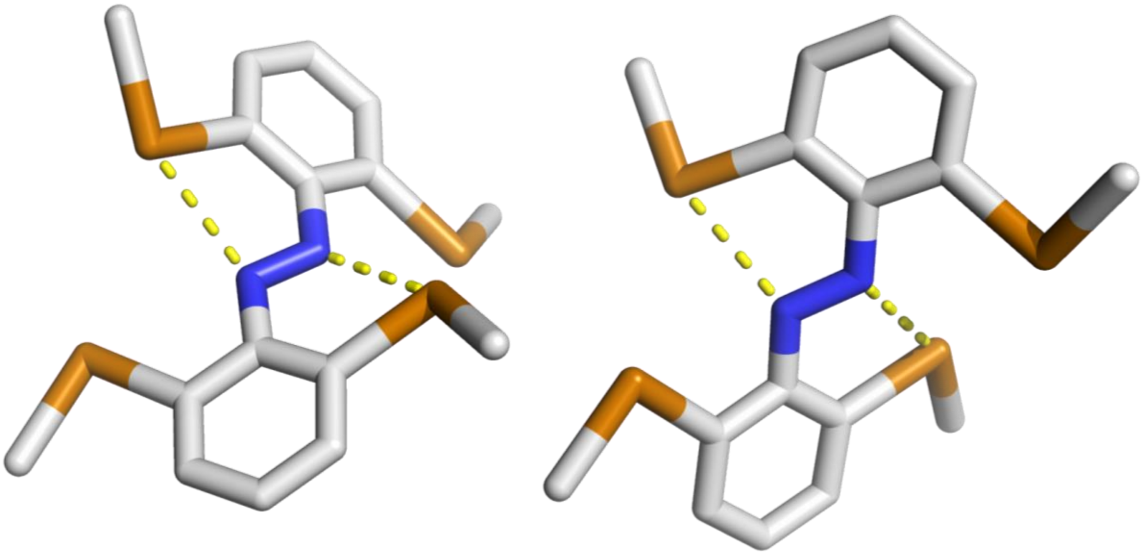

Figure S382. X-ray crystallographic structure of **Te_4-H.** Chalcogen bond interactions shown as dashed lines. Grey = carbon, blue = nitrogen, white = hydrogen, red = oxygen, orange = tellurium

Table S3. Crystal data and structure refinement for **Br_4-NHBoc**.

Empirical formula	C24 H28 Br4 N4 O4	
Formula weight	756.13	
Temperature	150 K	
Wavelength	1.54184 Å	
Crystal system	Monoclinic	
Space group	I2/a	
Unit cell dimensions	a = 20.6359(4) Å	α= 90°.
	b = 5.18090(10) Å	β= 104.528(2)°.
	c = 27.7349(6) Å	γ = 90°.
Volume	2870.40(10) $Å^3$	
Z	4	
Density (calculated)	1.750 Mg/m^3	
Absorption coefficient	7.147 mm^{-1}	
F(000)	1488	
Crystal size	0.31 x 0.05 x 0.01 mm^3	
Theta range for data collection	4.427 to 76.043°.	
Index ranges	-18<=h<=25, -6<=k<=6, -34<=l<=34	
Reflections collected	13970	
Independent reflections	2983 [R(int) = 0.041]	
Completeness to theta = 74.523°	99.6 %	
Absorption correction	Semi-empirical from equivalents	
Max. and min. transmission	0.93 and 0.23	
Refinement method	Full-matrix least-squares on F^2	
Data / restraints / parameters	2978 / 0 / 164	
Goodness-of-fit on F^2	1.1565	
Final R indices [I>2sigma(I)]	R1 = 0.1031, wR2 = 0.0656	
R indices (all data)	R1 = 0.1044, wR2 = 0.0666	
Largest diff. peak and hole	3.23 and -1.12 e.$Å^{-3}$	

Table S4. Crystal data and structure refinement for **I_4-N_3**.

Empirical formula	C14 H8 I4 N8	
Formula weight	795.89	
Temperature	150 K	
Wavelength	1.54184 Å	
Crystal system	Triclinic	
Space group	P -1	
Unit cell dimensions	a = 11.9316(3) Å	α= 73.159(4)°.
	b = 12.2715(5) Å	β= 73.857(3)°.
	c = 15.2444(7) Å	γ = 79.026(3)°.
Volume	2037.41(14) Å^3	
Z	4	
Density (calculated)	2.595 Mg/m^3	
Absorption coefficient	48.188 mm^{-1}	
F(000)	1440	
Crystal size	0.22 x 0.09 x 0.03 mm^3	
Theta range for data collection	3.791 to 76.110°.	
Index ranges	-14<=h<=13, -15<=k<=15, -18<=l<=19	
Reflections collected	20715	
Independent reflections	8417 [R(int) = 0.073]	
Completeness to theta = 73.827°	99.7 %	
Absorption correction	Semi-empirical from equivalents	
Max. and min. transmission	0.24 and 0.01	
Refinement method	Full-matrix least-squares on F^2	
Data / restraints / parameters	8412 / 0 / 469	
Goodness-of-fit on F^2	1.1626	
Final R indices [I>2sigma(I)]	R1 = 0.1252, wR2 = 0.1083	
R indices (all data)	R1 = 0.1306, wR2 = 0.1122	
Largest diff. peak and hole	6.41 and -2.29 e.Å^{-3}	

Table S5. Crystal data and structure refinement for **I_4-ester**.

Empirical formula	C18 H14 I4 N2 O4	
Formula weight	829.94	
Temperature	150 K	
Wavelength	1.54184 Å	
Crystal system	Monoclinic	
Space group	P 21/c	
Unit cell dimensions	a = 14.0017(3) Å	α= 90°.
	b = 8.23620(10) Å	β= 104.636(2)°.
	c = 19.1456(4) Å	γ = 90°.
Volume	2136.24(7) Å^3	
Z	4	
Density (calculated)	2.580 Mg/m^3	
Absorption coefficient	46.060 mm^{-1}	
F(000)	1520	
Crystal size	0.20 x 0.10 x 0.09 mm^3	
Theta range for data collection	5.058 to 76.433°.	
Index ranges	-17<=h<=17, -10<=k<=8, -23<=l<=23	
Reflections collected	18739	
Independent reflections	4444 [R(int) = 0.050]	
Completeness to theta = 74.904°	99.5 %	
Absorption correction	Semi-empirical from equivalents	
Max. and min. transmission	0.02 and 0.00	
Refinement method	Full-matrix least-squares on F^2	
Data / restraints / parameters	4443 / 0 / 253	
Goodness-of-fit on F^2	1.0359	
Final R indices [I>2sigma(I)]	R1 = 0.0694, wR2 = 0.1752	
R indices (all data)	R1 = 0.0716, wR2 = 0.1791	
Largest diff. peak and hole	4.27 and -1.58 e.Å^{-3}	

Table S6. Crystal data and structure refinement for **$(SMe)_4$-H**

Empirical formula	C16 H18 N2 S4	
Formula weight	366.60	
Temperature	150 K	
Wavelength	1.54184 Å	
Crystal system	Monoclinic	
Space group	P 2/n	
Unit cell dimensions	a = 15.1050(2) Å	α= 90°.
	b = 7.88010(10) Å	β= 90.6804(12)°.
	c = 28.9196(3) Å	γ = 90°.
Volume	3442.03(7) $Å^3$	
Z	8	
Density (calculated)	1.415 Mg/m^3	
Absorption coefficient	5.038 mm^{-1}	
F(000)	1535.991	
Crystal size	0.28 x 0.10 x 0.03 mm^3	
Theta range for data collection	3.285 to 76.196°.	
Index ranges	-13<=h<=18, -9<=k<=9, -28<=l<=36	
Reflections collected	24880	
Independent reflections	7138 [R(int) = 0.040]	
Completeness to theta = 74.672°	99.6 %	
Absorption correction	Semi-empirical from equivalents	
Max. and min. transmission	0.86 and 0.46	
Refinement method	Full-matrix least-squares on F^2	
Data / restraints / parameters	7138 / 0 / 397	
Goodness-of-fit on F^2	1.0088	
Final R indices [I>2sigma(I)]	R1 = 0.0418, wR2 = 0.1073	
R indices (all data)	R1 = 0.0486, wR2 = 0.1183	
Largest diff. peak and hole	1.10 and -0.55 e.$Å^{-3}$	

Table S7. Crystal data and structure refinement for **$(SEt)_4$-ester**

Empirical formula	C26 H34 N2 O4 S4	
Formula weight	566.83	
Temperature	150 K	
Wavelength	1.54184 Å	
Crystal system	Triclinic	
Space group	P -1	
Unit cell dimensions	a = 7.2152(2) Å	α= 87.957(3)°.
	b = 8.0923(3) Å	β= 82.964(3)°.
	c = 12.5451(4) Å	γ = 66.841(3)°.
Volume	668.32(4) $Å^3$	
Z	1	
Density (calculated)	1.408 Mg/m^3	
Absorption coefficient	3.561 mm^{-1}	
F(000)	300	
Crystal size	0.25 x 0.09 x 0.02 mm^3	
Theta range for data collection	3.550 to 76.057°.	
Index ranges	-9<=h<=8, -10<=k<=10, -15<=l<=15	
Reflections collected	12330	
Independent reflections	2761 [R(int) = 0.021]	
Completeness to theta = 73.776°	99.7 %	
Absorption correction	Semi-empirical from equivalents	
Max. and min. transmission	0.93 and 0.68	
Refinement method	Full-matrix least-squares on F^2	
Data / restraints / parameters	2761 / 0 / 163	
Goodness-of-fit on F^2	0.9995	
Final R indices [I>2sigma(I)]	R1 = 0.0245, wR2 = 0.0696	
R indices (all data)	R1 = 0.0252, wR2 = 0.0703	
Largest diff. peak and hole	0.40 and -0.27 e.$Å^{-3}$	

Table S8. Crystal data and structure refinement for **$(SeMe)_4$-H**

Empirical formula	C16 H18 N2 Se4	
Formula weight	554.17	
Temperature	150 K	
Wavelength	1.54184 Å	
Crystal system	Monoclinic	
Space group	P 2/n	
Unit cell dimensions	a = 15.5783(2) Å	α= 90°.
	b = 7.94990(10) Å	β= 90.3204(10)°.
	c = 29.5587(3) Å	γ = 90°.
Volume	3660.67(8) Å^3	
Z	8	
Density (calculated)	2.011 Mg/m^3	
Absorption coefficient	9.571 mm^{-1}	
F(000)	2111.991	
Crystal size	0.23 x 0.10 x 0.06 mm^3	
Theta range for data collection	5.299 to 76.285°.	
Index ranges	-18<=h<=19, -9<=k<=6, -32<=l<=36	
Reflections collected	38021	
Independent reflections	7607 [R(int) = 0.034]	
Completeness to theta = 74.759°	99.9 %	
Absorption correction	Semi-empirical from equivalents	
Max. and min. transmission	0.56 and 0.27	
Refinement method	Full-matrix least-squares on F^2	
Data / restraints / parameters	7605 / 0 / 398	
Goodness-of-fit on F^2	1.0394	
Final R indices [I>2sigma(I)]	R1 = 0.0250, wR2 = 0.0614	
R indices (all data)	R1 = 0.0266, wR2 = 0.0625	
Extinction coefficient	7.9(9)	
Largest diff. peak and hole	0.79 and -1.29 e.Å^{-3}	

Table S9. Crystal data and structure refinement for **$(TeMe)_4$-H**

Empirical formula	C16 H18 N2 Te4	
Formula weight	748.73	
Temperature	150 K	
Wavelength	1.54184 Å	
Crystal system	Monoclinic	
Space group	P 21	
Unit cell dimensions	a = 10.73350(10) Å	α= 90°.
	b = 10.57430(10) Å	β= 95.2723(7)°.
	c = 18.02080(10) Å	γ = 90°.
Volume	2036.69(3) $Å^3$	
Z	4	
Density (calculated)	2.442 Mg/m^3	
Absorption coefficient	44.738 mm^{-1}	
F(000)	1344	
Crystal size	0.03 x 0.03 x 0.005 mm^3	
Theta range for data collection	4.136 to 76.409°.	
Index ranges	-13<=h<=13, -13<=k<=12, -22<=l<=22	
Reflections collected	45376	
Independent reflections	8268 [R(int) = 0.036]	
Completeness to theta = 74.117°	99.9 %	
Absorption correction	Semi-empirical from equivalents	
Max. and min. transmission	0.80 and 0.24	
Refinement method	Full-matrix least-squares on F^2	
Data / restraints / parameters	8268 / 1 / 398	
Goodness-of-fit on F^2	0.9937	
Final R indices [I>2sigma(I)]	R1 = 0.0249, wR2 = 0.0600	
R indices (all data)	R1 = 0.0263, wR2 = 0.0606	
Absolute structure parameter	-0.020(4)	
Largest diff. peak and hole	1.15 and -0.56 e.$Å^{-3}$	

Table S10. Crystal data and structure refinement for **$(SeMe)_4$-ester**

Empirical formula	C22 H26.00 N2 O4 Se4	
Formula weight	698.30	
Temperature	150 K	
Wavelength	1.54184 Å	
Crystal system	Orthorhombic	
Space group	P b c n	
Unit cell dimensions	a = 16.0349(3) Å	α= 90°.
	b = 15.8463(4) Å	β= 90°.
	c = 19.0464(4) Å	γ = 90°.
Volume	4839.57(18) Å^3	
Z	8	
Density (calculated)	1.917 Mg/m^3	
Absorption coefficient	7.531 mm^{-1}	
F(000)	2719.987	
Crystal size	0.14 x 0.11 x 0.09 mm^3	
Theta range for data collection	3.922 to 77.501°.	
Index ranges	-20<=h<=10, -18<=k<=19, -23<=l<=23	
Reflections collected	43637	
Independent reflections	5050 [R(int) = 0.050]	
Completeness to theta = 75.176°	99.8 %	
Absorption correction	Semi-empirical from equivalents	
Max. and min. transmission	0.51 and 0.35	
Refinement method	Full-matrix least-squares on F^2	
Data / restraints / parameters	5050 / 0 / 289	
Goodness-of-fit on F^2	1.0194	
Final R indices [I>2sigma(I)]	R1 = 0.0399, wR2 = 0.1076	
R indices (all data)	R1 = 0.0502, wR2 = 0.1190	
Largest diff. peak and hole	1.48 and -0.77 e.Å^{-3}	

Table S12. Crystal data and structure refinement for **Te_2F_2-H.**

Empirical formula	C14 H12 F2 N2 Te2	
Formula weight	501.46	
Temperature	150 K	
Wavelength	0.71073 Å	
Crystal system	Monoclinic	
Space group	$P2_1/n$	
Unit cell dimensions	a = 6.4567(7) Å	α= 90°.
	b = 8.6728(9) Å	β= 92.320(9)°.
	c = 13.3795(13) Å	γ = 90°.
Volume	748.61(13) $Å^3$	
Z	2	
Density (calculated)	2.225 Mg/m^3	
Absorption coefficient	3.909 mm^{-1}	
F(000)	464	
Crystal size	0.15 x 0.10 x 0.08 mm^3	
Theta range for data collection	3.450 to 30.490°.	
Index ranges	-9<=h<=9, -12<=k<=12, -18<=l<=18	
Reflections collected	6642	
Independent reflections	2012 [R(int) = 0.066]	
Completeness to theta = 27.727°	99.8 %	
Absorption correction	Semi-empirical from equivalents	
Max. and min. transmission	0.73 and 0.59	
Refinement method	Full-matrix least-squares on F^2	
Data / restraints / parameters	1580 / 0 / 91	
Goodness-of-fit on F^2	1.0063	
Final R indices [I>2sigma(I)]	R1 = 0.0337, wR2 = 0.0580	
R indices (all data)	R1 = 0.0646, wR2 = 0.0707	
Largest diff. peak and hole	2.27 and -2.26 e.$Å^{-3}$	

Table S13. Crystal data and structure refinement for **F_2,H_2-NHBoc**.

Empirical formula	C24 H30 F2 N4 O4	
Formula weight	476.52	
Temperature	150 K	
Wavelength	1.54184 Å	
Crystal system	Triclinic	
Space group	P -1	
Unit cell dimensions	a = 10.1072(7) Å	α= 94.868(6)°.
	b = 11.4012(8) Å	β= 95.841(6)°.
	c = 11.5402(9) Å	γ = 112.726(7)°.
Volume	1208.99(17) Å^3	
Z	2	
Density (calculated)	1.309 Mg/m^3	
Absorption coefficient	0.841 mm^{-1}	
F(000)	504	
Crystal size	0.26 x 0.05 x 0.02 mm^3	
Theta range for data collection	3.886 to 77.150°.	
Index ranges	-11<=h<=12, -13<=k<=14, -14<=l<=14	
Reflections collected	12314	
Independent reflections	5019 [R(int) = 0.038]	
Completeness to theta = 72.521°	99.5 %	
Absorption correction	Semi-empirical from equivalents	
Max. and min. transmission	0.98 and 0.88	
Refinement method	Full-matrix least-squares on F^2	
Data / restraints / parameters	5012 / 0 / 307	
Goodness-of-fit on F^2	0.9792	
Final R indices [I>2sigma(I)]	R1 = 0.0509, wR2 = 0.1349	
R indices (all data)	R1 = 0.0760, wR2 = 0.1584	
Largest diff. peak and hole	0.37 and -0.33 e.Å^{-3}	

7. References

[1] A. Kerckhoffs, Z. Bo, S. E. Penty, F. Duarte, M. J. Langton, *Organic & Biomolecular Chemistry* **2021**, *19*, 9058-9067.

[2] D. Bléger, J. Schwarz, A. M. Brouwer, S. Hecht, *Journal of the American Chemical Society* **2012**, *134*, 20597-20600.

[3] B. Heinrich, K. Bouazoune, M. Wojcik, U. Bakowsky, O. Vázquez, *Organic & Biomolecular Chemistry* **2019**, *17*, 1827-1833.

[4] A. Kerckhoffs, M. J. Langton, *Chemical Science* **2020**, *11*, 6325-6331.

[5] K. Khosravi, S. Naserifar, *Tetrahedron* **2018**, *74*, 6584-6592.

[6] M. Dong, A. Babalhavaeji, C. V. Collins, K. Jarrah, O. Sadovski, Q. Dai, G. A. Woolley, *Journal of the American Chemical Society* **2017**, *139*, 13483-13486.

[7] Y. Takeda, S. Okumura, S. Minakata, *Synthesis* **2013**, *45*, 1029-1033.

[8] S. M. Walter, S. H. Jungbauer, F. Kniep, S. Schindler, E. Herdtweck, S. M. Huber, *Journal of Fluorine Chemistry* **2013**, *150*, 14-20.

[9] B. Latli, R. Kiesling, S. Aßfalg, M. Chevliakov, M. Hrapchak, S. Campbell, N. Gonnella, C. A. Busacca, C. H. Senanayake, *Journal of Labelled Compounds and Radiopharmaceuticals* **2016**, *59*, 648-656.

[10] A. R. T. A.I. Vogel, B.S. Furnis, A.J. Hannaford, P.W.G Smith, *Vogel's Textbook of Practical Organic Chemistry*, Prentice Hall, **1989**.

[11] S. V. Khansole, S. B. Junne, M. A. Sayyed, Y. B. Vibhute, *Synthetic Communications* **2008**, *38*, 1792-1798.

[12] J. Hirschfeld, A. Buschauer, S. Elz, W. Schunack, M. Ruat, E. Traiffort, J. C. Schwartz, *Journal of Medicinal Chemistry* **1992**, *35*, 2231-2238.

[13] A.-L. Leistner, S. Kirchner, J. Karcher, T. Bantle, M. L. Schulte, P. Gödtel, C. Fengler, Z. L. Pianowski, *Chemistry – A European Journal* **2021**, *27*, 8094-8099.

[14] N. H. Park, E. V. Vinogradova, D. S. Surry, S. L. Buchwald, *Angewandte Chemie International Edition* **2015**, *54*, 8259-8262.

[15] R. Dubey, H. Lee, D.-H. Nam, D. Lim, *Tetrahedron Letters* **2011**, *52*, 6839-6842.

[16] S. T. Gadge, A. Mishra, A. L. Gajengi, N. V. Shahi, B. M. Bhanage, *RSC Advances* **2014**, *4*, 50271-50276.

[17] G. L. A. M. N. P. D. K. J. N. S. D. M. K. Sheetal, Glenmark Pharamceuticals SA, **2016**.

[18] M. A. B. Mostafa, R. M. Bowley, D. T. Racys, M. C. Henry, A. Sutherland, *The Journal of Organic Chemistry* **2017**, *82*, 7529-7537.

[19] C. C. Woodroofe, B. Zhong, X. Lu, R. B. Silverman, *Journal of the Chemical Society, Perkin Transactions 2* **2000**, 55-59.

[20] S. S. Matikonda, J. M. Fairhall, F. Fiedler, S. Sanhajariya, R. A. J. Tucker, S. Hook, A. L. Garden, A. B. Gamble, *Bioconjug Chem* **2018**, *29*, 324-334.

[21] A. A. Estrada, X. Liu, C. Baker-Glenn, A. Beresford, D. J. Burdick, M. Chambers, B. K. Chan, H. Chen, X. Ding, A. G. DiPasquale, S. L. Dominguez, J. Dotson, J. Drummond, M. Flagella, S. Flynn, R. Fuji, A. Gill, J. Gunzner-Toste, S. F. Harris, T. P. Heffron, T. Kleinheinz, D. W. Lee, C. E. Le Pichon, J. P. Lyssikatos, A. D. Medhurst, J. G. Moffat, S. Mukund, K. Nash, K. Scearce-Levie, Z. Sheng, D. G. Shore, T. Tran, N. Trivedi, S. Wang, S. Zhang, X. Zhang, G. Zhao, H. Zhu, Z. K. Sweeney, *Journal of Medicinal Chemistry* **2012**, *55*, 9416-9433.

[22] T. Suzuki, K. Iwaoka, N. Imanishi, Y. Nagakura, K. Miyata, H. Nakahara, M. Ohta, T. Mase, *CHEMICAL & PHARMACEUTICAL BULLETIN* **1999**, *47*, 120-122.

[23] R. L. Mackman, B. A. Katz, J. G. Breitenbucher, H. C. Hui, E. Verner, C. Luong, L. Liu, P. A. Sprengeler, *Journal of Medicinal Chemistry* **2001**, *44*, 3856-3871.

[24] A. Granados, A. Shafir, A. Arrieta, F. P. Cossío, A. Vallribera, *The Journal of Organic Chemistry* **2020**, *85*, 2142-2150.

[25] M. Dong, A. Babalhavaeji, M. J. Hansen, L. Kálmán, G. A. Woolley, *Chemical Communications* **2015**, *51*, 12981-12984.
[26] N. Sakai, S. Asama, S. Anai, T. Konakahara, *Tetrahedron* **2014**, *70*, 2027-2033.
